# Traffic light optimization using non-dominated sorting genetic algorithm (NSGA2)

**DOI:** 10.1038/s41598-023-38884-2

**Published:** 2023-09-20

**Authors:** Samara Soares Leal, Paulo Eduardo M. de Almeida

**Affiliations:** 1 University Center of Belo Horizonte – UniBH, Av. Professor Mário Werneck, 1685, Buritis, Belo Horizonte, MG 30575-180 Brazil; 2 Federal Center of Technological Education of Minas Gerais – CEFET-MG, Av. Amazonas, 7675, Belo Horizonte, MG 30510-000 Brazil

**Keywords:** Computational science, Computer science, Information technology, Scientific data, Software

## Abstract

Traffic congestion is a major concern in urban centers, as it can affect society, the environment, and the economy. There are many studies on the use of computational intelligence (CI) to improve mobility in urban centers. Some of these researches focus on developing strategies for traffic light programming, since traffic coordination is complex due to its many parameters, variables, and dynamic behavior, and also an inefficient traffic control plan can lead to increased delays and contribute to traffic congestion. Although there are many works in the literature on strategies for traffic control, there are still some contributions and gaps to be filled, especially because some studies do not consider the automatic optimization of traffic signals in real time, that is, according to the demand of vehicles on the roads, considering multiple objectives and the use of a network of intersections in their experiments. In addition, some of the proposed models are not independent of simulation to evaluate the solutions of CI algorithms, resulting in a more complex deployment in real situations. In this context, this paper presents a new method to optimize traffic light plan in a network of intersections and in real time, called Active Control of Traffic Signals (ACTS) associated with the Non-Dominated Sorting Genetic Algorithm, considering multiple objectives in the optimization process (minimizing the average delay time and the number of vehicles stops per cycle). To test the applicability of the model, a real dataset of vehicle demand collected by the Company of Transport and Traffic of Belo Horizonte (BHTrans) is loaded into the AIMSUN simulator, then the method is applied and compared with the current traffic control plan used by BHTrans. The results show that the ACTS method reduces the average vehicle delay by almost half compared to the results obtained with the current solution used by BHTrans. In real life, this means less time spent in traffic, which promotes faster traffic flow, reducing traffic congestions.

## Introduction

The large number of vehicles circulating in city centers has been a strong trend in recent years. In Brazil, for example, the vehicle fleet is expected to grow by about 140% over the next 20 years, reaching 95.2 million units^1^. One of the main symptoms of this growth is traffic congestion, which affects the quality of life of the population and causes economic, social and environmental damage.

Due to this increase in the number of vehicles circulating on the roads, one of the main challenges in building a smart city is the implementation of an intelligent mobility plan. In Brazil, the current urban mobility policies are not sufficient to control the problems caused by the increase in traffic congestion in large and medium-sized cities. That's why many studies and important contributions have been made in recent years to develop Intelligent Transport Systems (ITS) in the country. One of the most important areas of ITS is Advanced Travel Management Systems (ATMS). In ATMS, among the different strategies, intelligent or active traffic signal control is highlighted because of its ability to adapt the values of traffic control parameters according to the traffic conditions in real time, reducing travel times, delays and, consequently, improving the traffic network^2^.

Furthermore, several computational intelligence (CI) techniques using evolutionary algorithms have been proposed in the ATMS literature for urban traffic optimization. According to Ohazulike and Brands^3^, in recent years, many researchers have turned their attention to solving multi-objective traffic control problems using evolutionary algorithms because real-world decision processes have many social concerns and thus need to achieve multiple objectives simultaneously. The research approaches of Ohazulike and Brands^3^, Costa et al.^4^, Wang et al.^5^, Zhou et al.^6^ use multi-objective models to solve traffic control problems considering different objectives.

In this context, this paper presents a new method to optimize traffic light plan in a network of intersections and in real time, entitled Active Control of Traffic Signals (ACTS), associated with a CI multi-objective technique called Non-Dominated Sorting Genetic Algorithm (NSGA2), which is an extension and evolution of the Costa et al.^4^, aiming to overcome the limitations observed in the literature and in that work, such as: The lack of a model that optimizes the traffic light control automatically and in real time, that is, according to the demand of vehicles in a network of intersections, and that this model is independent of simulation to evaluate the solutions of the algorithms, and therefore faster and easier to apply in real situations.

Giving this, the general objective of this paper is to present ACTS to dynamically generate optimal traffic signal control plans for different traffic conditions, using the NSGA2 in the optimization process, while satisfying the objectives of minimizing the average vehicle delay time and the number of vehicles stops per cycle. NSGA2 was chosen because it presented good results in Costa et al.^4^ and it’s a widely used genetic algorithm for dealing with multi-objective optimization in many areas.

For the validation of the proposed method, real vehicle flow data from a region of the city of Belo Horizonte, Brazil, collected by the Transport and Traffic Company of Belo Horizonte (BHTrans) were used in the experiments. Since at the time of this research there was no way to perform the entire feedback process of the system in the real world, this traffic data was used for the ACTS experiments in the AIMSUN simulator. Thus, to perform the dynamic operation of the system, at each simulated time interval (i.e., cycle time), the model receives the vehicle flow data generated in AIMSUN and determines a good traffic light schedule for that period that minimizes the objective functions. Therefore, the AIMSUN simulator is only used to perform the urban road traffic simulations and is not used to evaluate the NSGA solution, as will be seen in the next sections.

The experimental results obtained with NSGA2 are compared with the current traffic signal control plan used by BHTrans, and also with results obtained with a mono-objective approach using genetic algorithms (GA), in order to evaluate whether NSGA2 is more efficient in optimizing the operation of traffic signals in a region of intersections.

This paper is organized as follows: Section "Background" describes the background and presents some relevant studies in the field. Section "The Proposed Method" presents the proposed ACTS method. Section "Case Study and Experimental Results" describes the case study, the experiments and analyzes the results. Finally, Section "Discussions" presents some discussion about this work and Section "Conclusion" summarizes the main conclusions, future work and opportunities.

## Background

### Non-dominated sorting genetic algorithm 2 (NSGA2)

Due to the use of an evolutionary technique within ACTS, it is necessary to present general aspects about the NSGA2 method and to discuss the computational representation of the decision variables that will be used in the method, in order to allow a good understanding of the optimization technique implemented in this approach. Also, information about the fitness and objective functions used in the model will be given, and some details about the evolutionary operators used will be presented in Section "The Proposed Method".

Before explaining NSGA2, it’s important to briefly present its mono-objective counterpart GA (Genetic Algorithm). According to Pacheco^7^, GA is a CI algorithm inspired by simplified models of natural evolution. GA process an initial set of possible solutions to a problem (initial population). The elements that constitute this population are designated by individuals (decision variables) that are transformed and evolved over successive generations. Due to the selection mechanism, individuals that constitute a new population tend to be better than individuals from previous generations, that is, they represent a better solution to the problem.

NSGA2 was developed in 2002 by Deb et al.^8^. The method builds a fitness mechanism from the ranking algorithm fast non-dominated sorting and implements the diversity preservation operator crowding distance. The fast non-dominated sorting procedure is responsible for classifying the dominance levels of each solution and then generating bounds for classifying the solutions. The crowding distance procedure is divided into two parts: the density estimation metric and the crowded comparison operator, which guides the algorithm's selection process.

The general procedure of NSGA2 starts with a randomly generated initial population of solutions. Individuals from this population are evaluated and the operators are applied to the solutions, defining the best solutions so far, and parents are selected to generate offspring. Parents are crossed, creating more children, and a mutation operator is applied to them. Finally, the children are evaluated and, together with the parents, integrate the next generation. This process is repeated until some stopping criterion is met, while the global quality of the solutions tends to increase from one generation to the next.

### State-of-the-art

Several works on traffic signal optimization using evolutionary techniques can be found in the literature. Among the studies, some are mentioned in this paper because of their relevance, the way they approached the problem, and their limitations.

Nowadays, GA and its multi-objective counterpart NSGA2 have been used in various approaches to traffic signal optimization. In the work of Kwasnicka et al.^9^, traffic data is obtained from the simulation and serves as input to a multi-objective GA whose goals are to minimize the time lost and to maximize the average speed of vehicles. All traffic signal control plans of the intersections are encoded in a chromosome. A limitation of this work is that the need for simulation to evaluate each solution obtained can make the optimization process slow and totally dependent on computational delays.

Hua et al.^10^ implemented the NSGA2 algorithm for the optimization of a fixed-time multi-objective traffic signal model in an isolated intersection. The authors proposed a method in which the optimized variables are cycle size and green times. The proposed optimization model used three objective functions: average vehicle delay time, average number of stops, and vehicle queue size. In this work, compared to the mono-objective optimization method GA, NSGA2 significantly improved the quality of the solutions obtained.

Costa et al.^4^ used micro-regions of the city of Belo Horizonte to optimize fixed-time control plans with the aim of maximizing the average speed of vehicles and minimizing the variance of their speeds. The authors implemented NSGA2 and MBVL-NSGA2 (Memory-Based Variable-Length Encoding Non-Dominated Sorting Genetic Algorithm 2), which combines the use of NSGA2 with a memory mechanism that eliminates re-evaluation of solutions between generations, and an adaptive neighborhood mechanism to control the sampling density of new solutions. This work used fixed-time traffic control, i.e., there is no feedback system using actual intersection traffic data, and simulation is used extensively to evaluate solutions.

Singh, Tripathi and Arora^11^ presented a GA-based traffic control strategy to promote good performance at an isolated intersection. The developed intelligent system was able to make real-time decisions to modify the green times for a set of traffic lights. In addition, the authors developed an emulator to represent the traffic conditions at an isolated intersection. The emulator was designed to send the traffic data to the algorithm, which then returned the traffic light green time setting that minimized a performance index chosen as the fitness function. Finally, comparing the experimental result obtained by the fixed traffic control with the real-time control model proposed by the authors, the efficiency of the real-time model was proven.

It is clear that various approaches have been proposed to solve the problem of traffic signal optimization. Given this scenario, this paper reports the development of a method to address some limitations existing in these approaches. The ACTS method proposed here will be able to implement real-time feedback on a traffic control system in a network of intersections, taking into account multiple objectives. Moreover, the method does not depend on simulation to evaluate the solutions obtained, which is an advantage of this approach, since this is a serious limitation of previous methods.

## The proposed method

Active control of traffic is a technique that has the ability to dynamically manage recurring and non-recurring congestion based on existing traffic conditions^2^. According to Grant^12^, the active traffic control is a traffic signal control technique that has already been implemented in several European countries and in the United States and several benefits have been observed, such as reduction in congestion and in the emission of pollutants; reduction in the average travel time and in the average delay time of vehicles; the improvement in security; between others.

The use of this technique is also promising in Brazil and although there are not many public policies for mobility and investment culture in active traffic control in the country, some studies have already been made about this strategy. In the work of Loureiro et al.^13^, the performance of traffic management with fixed time plans of the software TRANSYT is compared with the real time control of the SCOOT system, evaluating the indicators of average delay and volume in each approximation of the intersections under study. Meneses et al.^14^, present a discussion on the performance indicators for a real time traffic control system for the city of Fortaleza—Brazil.

Based on the state-of-the-art it can be seen that active control is a promising area to improve urban mobility in Brazil. In this context and to overcome the limitations found in previous works and to explore the opportunities pointed out by the literature review, this paper proposes an active traffic control method—ACTS. This method is associated to NSGA2 to determine, in real time, a good configuration of traffic signal parameters that minimizes the average delay time of vehicles in intersection networks, according to the actual traffic demand obtained at each traffic signal cycle time interval.

In addition, this model does not depend on simulation to evaluate the solutions of CI algorithms, which is an advantage of this work, since this is one of the major problems in the previous approaches, as it makes the search process slow and difficult to adapt and use in the real world.

Since traffic solutions are usually analyzed using traffic simulation models, this paper only uses a traffic simulator called AIMSUN in the experiments to test the model in a network of intersections. Although the experiments reported here are carried out using AIMSUN, the proposed technique can be implemented in a real traffic network by replacing AIMSUN with real-time data obtained from sensors installed in the traffic infrastructure, for example.

**Figure 1:**
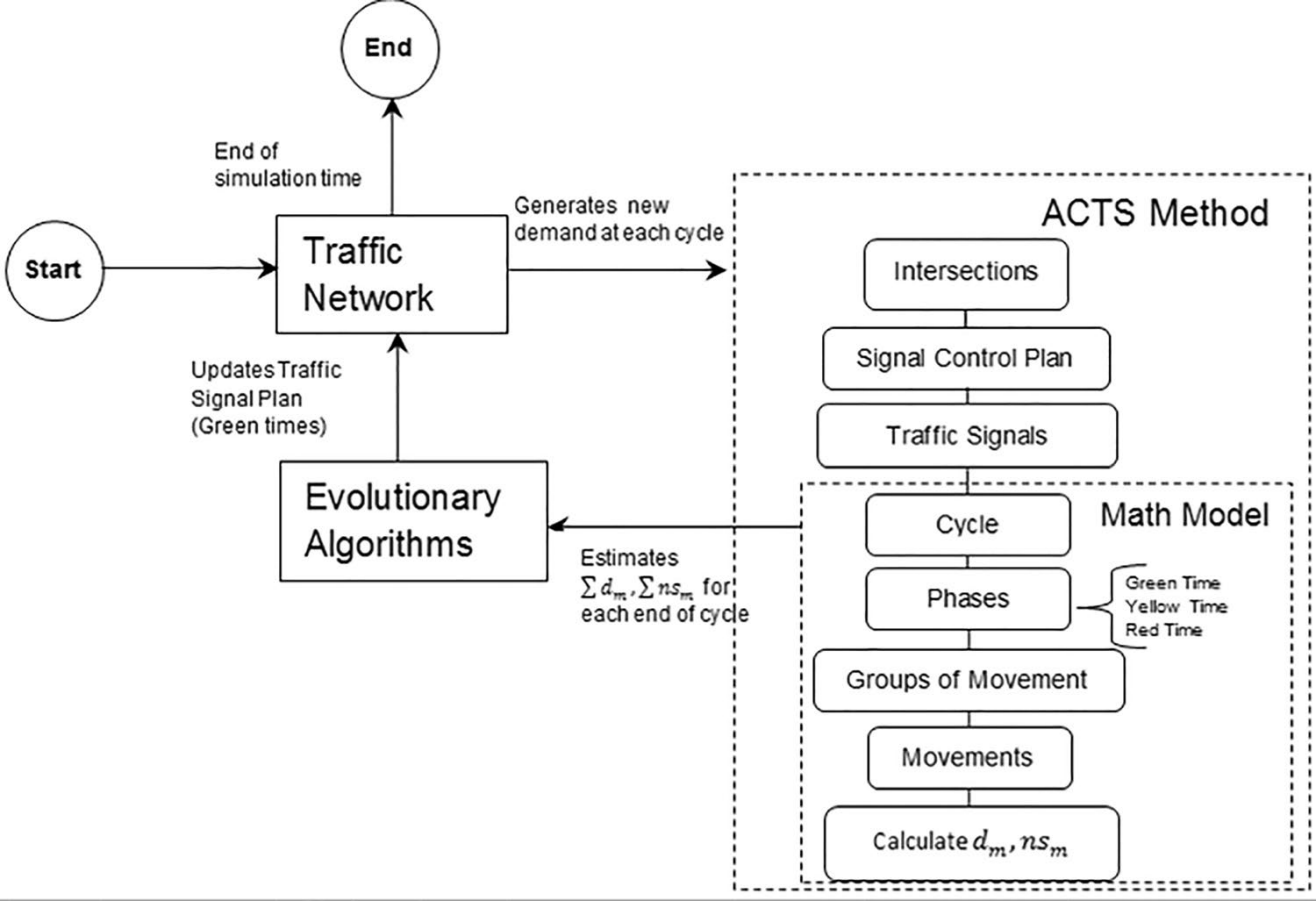
General model of ACTS.

Figure 1 briefly illustrates a general scheme for ACTS, as proposed in this work, that can be used in networked intersections. This scheme consists of three parts: a traffic network to be controlled, a mathematical model to estimate the traffic behavior in the network and an evolutionary algorithm (e.g., GA or NSGA2) to optimize the signal control plans in the network.

The interactions between these parts are described below. They occur at each cycle time:

1. The ACTS method feeds flow data collected from the network under control. In the experiments conducted here, these data are collected from the traffic network drawn and simulated by AIMSUN. The traffic demand data loaded into AIMSUN is based on actual data provided by BHTrans. This traffic network generates instantaneous flow data for the model at every cycle through simulation. However, the proposed method can be easily implemented in a real network by replacing AIMSUN simulations with real-time data collected by sensors installed on a real traffic infrastructure.
2. The traffic network consists of a group of intersections. A traffic signal control plan determines the signal timing, which consists of cycles *c* defined for each intersection. Each cycle is composed of a fixed sequence of phases. Each phase contains a group of non-conflicting movements at each intersection. For each movement of the group, a green time is defined. Thus, the green times *x* of each phase correspond to the sum of the green times of all the movements belonging to a phase. These green times *x* are the decision variables in the optimization model. After generating *x*, the average vehicle delay and the number of vehicles stops at each movement are estimated. The sum of delay and number of vehicles stops in all movements corresponds to the average delay time per cycle,* D*, and the total number of vehicles stops per cycle, *NS*.
3. Thus, at the end of each cycle, the model estimates the value of *D* and *NS*, which are the evaluation functions that drive the optimization process.
4. The estimated values *D* and *NS* are then used to evaluate different optimization solutions generated by evolutionary algorithms (e.g., GA and NSGA2). Once a good solution has been found, ACTS updates the green times *x* of the current signal control plans for all movements m in the network. Then the optimization process recycles: a new cycle time starts; ACTS dynamically collect traffic states and responds by finding another optimal signal control plan to meet the current demand with respect to the defined objective functions.

In summary, ACTS is executed at the end of each cycle time period, repeating the process of feeding the system with new traffic data, generating valid and good solutions by the evolutionary optimizer, and updating the signal control plans back to the network to properly respond to changes in demand.

The next subsection presents the NSGA2 operators, variables and functions used in this paper. For this purpose and for simplification (since the main objective of this paper is the active optimization of traffic lights using a CI multiobjective technique (NSGA2) and not the in-depth study of variations in the behavior of the algorithm), and for the presentation of good results in Costa et al.^4^, the set of computational definitions used was:

### Genetic operators

There are many implementations of genetic operators (selection, crossover and mutation) discussed in the literature to be used with NSGA2^6^. The Tournament Operator was chosen for selection, along with the Uniform Crossing for crossover and the Gaussian method for mutation, as they have shown good performance in previous approaches.

### NSGA2 parameters

After several runs of NSGA2 and observation of its behavior between experiments, a set of parameters was chosen that gave the best convergence rates. For the sake of simplification (since the main objective of this paper is to present a traffic control method that can be applied to a CI technique, and not to study in depth the variations in the behavior of the algorithm used), and because it presented good results in Leal et al.^15^, the set of values used can be seen in Table 1.

**Table 1:** Parameters of NSGA2 for the experiments.

Population size	Generations	Crossover rate	Mutation rate
100	2000	80%	4%

This set of parameters was chosen after using some different combinations of them and observing that this set gave better results and that no significant improvement in the results was observed by changing them. The crossover and mutation percentages are those generally used in the literature, which suggest that the crossover rate is high (more than 70 percent) and the mutation rate is low—around 3 or 5 percent, according to Gaspar-Cunha et al.^16^. The stopping criterion was the maximum number of generations.

### Decision variables

The decision variables correspond to the set of green times for each signal phase, i.e., together they form a signal control plan for the controlled traffic network. For this paper, a real-valued representation has been adopted, where each individual (or solution) consists of a real-valued vector representing the decision variables.

For simplicity, but without loss of generality, in the experiments reported here, a unique cycle time was predefined for all intersections in the network under control. Since a cycle time is the sum of green times and inter-green times (i.e., an equality constraint imposed on the problem), changing the values of a green time would inevitably cause the total time to differ from the predefined cycle time. As this is undesirable, a moveable partition strategy was adopted, as described next.

### Movable partitions strategy

**Figure 2:**
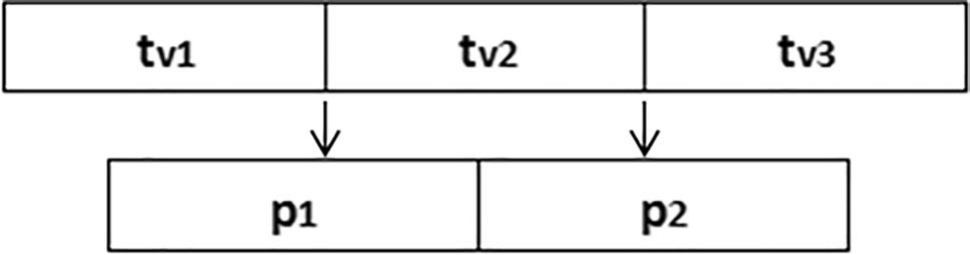
Movable partitions strategy.

In order to deal with the equality constraint discussed above, a strategy of movable partitions was adopted^17^. This strategy uses *N-1* partitions to represent *N* decision variables. To explain the operation of this strategy, consider an example of a single intersection with a control plan consisting of 1 cycle with 3 phases. For each phase, the optimizer would calculate a green time. The number *N* of decision variables would therefore be three. Using this strategy, instead of representing all three solutions of this example (tv1, tv2 and tv3), only *N-1* partitions could be used, i.e., 2 partitions (p1 and p2), as can be seen in Fig. 2. In this way, the objective of the optimization algorithm becomes finding optimal positions of these *N-1* partitions, rather than finding *N* optimal green times. The only concern here is to keep the values of the partitions always different from each other during the optimization process to avoid a corresponding unwanted zero-length green time.

A side effect of using this strategy is a reduction in the dimension of the search space for the optimization algorithm. In fact, if the network has more than one intersection, this reduction is even better, reducing the dimension of the problem.

### Fitness function

As seen in the previous subsection, solutions must be feasible and correctly represent a signal control plan with non-zero green times and predefined cycle times. To avoid infeasible solutions with green times x equal to zero (i.e., the inequality constraint *x* > *0*), repeated partitions must not occur. In other words, partitions occupying the same location at the same time will represent at least one green time x equal to zero. Thus, a penalty function (*PF*) is added to the individual's fitness function (in this paper, the fitness function corresponds to the objective function (*OF*)). Obviously, individuals will avoid forbidden areas due to the low fitness they will reach if they don't respect the imposed constraint. With this strategy, the score S of the individual *x* is given by Eq. (1):

1 $$S\left(x\right)=OF\left(x\right)+kp*PF(x)$$

in which *kp* is the penalty constant applied, and *x* are the green times for the network.

### Objective functions

The average delay time *D* measures the waiting time caused to vehicles by existing traffic signals. The objective function one *D*, given in seconds, can be determined by Eq. (2)^17,18^:

2 $$D(x)=({C.\left(1-p\left(x\right)\right)}^{2}) / (2.\left(1-p\left(x\right).s\right))$$

in which *C* is the cycle time (seconds);* p* is the green fraction (the relationship between green time *x* and cycle time *C*); and *s* is the degree of saturation (the ratio between demand and capacity).

All parameters are calculated from data obtained from sensors in the infrastructure (in our experiments they are provided by the AIMSUN simulator). In addition to the problems caused by the increase in delay time, there is also the increase in the number of vehicles suffering stops per cycle *NS* (number of stops), which also causes large congestion. The objective function number two *NS* can be determined by Eq. (3)^17,18^

3 $$NS\left(x\right)= \frac{q*S}{S-q}* \frac{C-x}{3600},$$

in which *q* is the is the flow (vehicles per hour); *S* is the saturation flow (vehicles per hour); *C* is the cycle time (seconds) and *x* is the green time (seconds).

This problem has a multi-objective optimization formulation according to Eq. (4), i.e., it is intended to satisfy the two objective functions simultaneously:

4 $$minimize\left\{\begin{array}{c} D\left(x\right)=Average Delay Time;\\ NS\left(x\right)=Number of Stops.\end{array}\right.$$

Note the multi-objective nature of the problem in Eq. 4. The two functions are not correlated, as minimizing the average delay time of vehicles crossing a network does not imply reducing the number of vehicles stopping per cycle.

The delay was chosen because it is one of the most used function in traffic light optimization studies (it can be seen in my literature review) since delay is basically the time that vehicles lose waiting at traffic lights. Delay also influences in travel times and queue size functions. The number of stops was chosen in this paper because it’s highly influenced by the saturation flow, which is the period that has the most impact when using traffic control plan strategies.

## Case study and experimental results

The experiments carried out in this paper are presented in the following subsections.

### Case study

**Figure 3:**
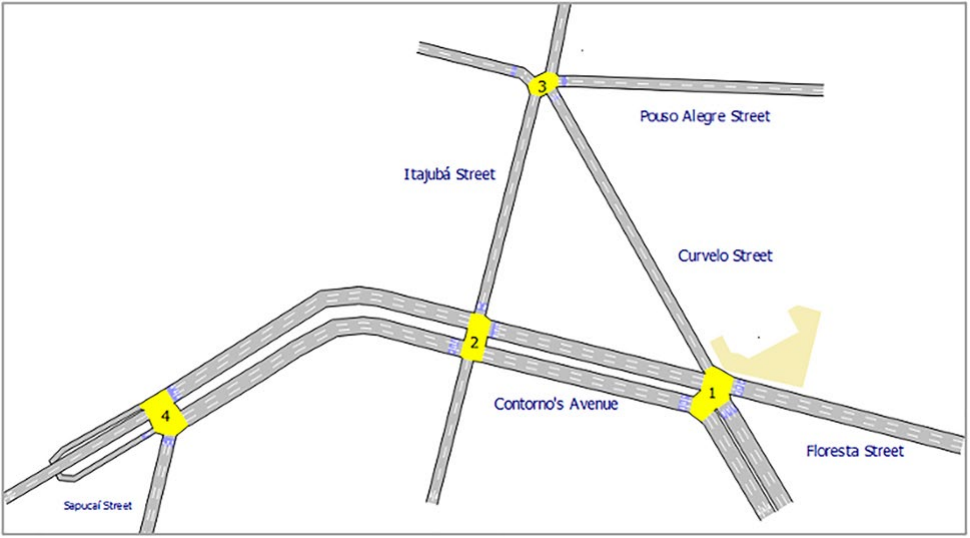
Traffic network reproduced in AIMSUN.

The traffic network reproduced in AIMSUN corresponds to a region of the Floresta neighborhood, near the center of Belo Horizonte, in Brazil, a capital city with about 4 million inhabitants and a huge fleet of more than 1.2 million private vehicles. The study area consists of four signalized intersections, as shown in Fig. 3 and Table 2.

**Table 2:** Characteristics of the intersections in the Floresta region.

ID	Intersections	Number of movements	Number of phases
1	Contorno and Curvelo	7	3
2	Contorno and Itajubá	6	2
3 4	Pouso Alegre and Curvelo Contorno and Sapucaí	4 5	2 2

**Figure 4:**
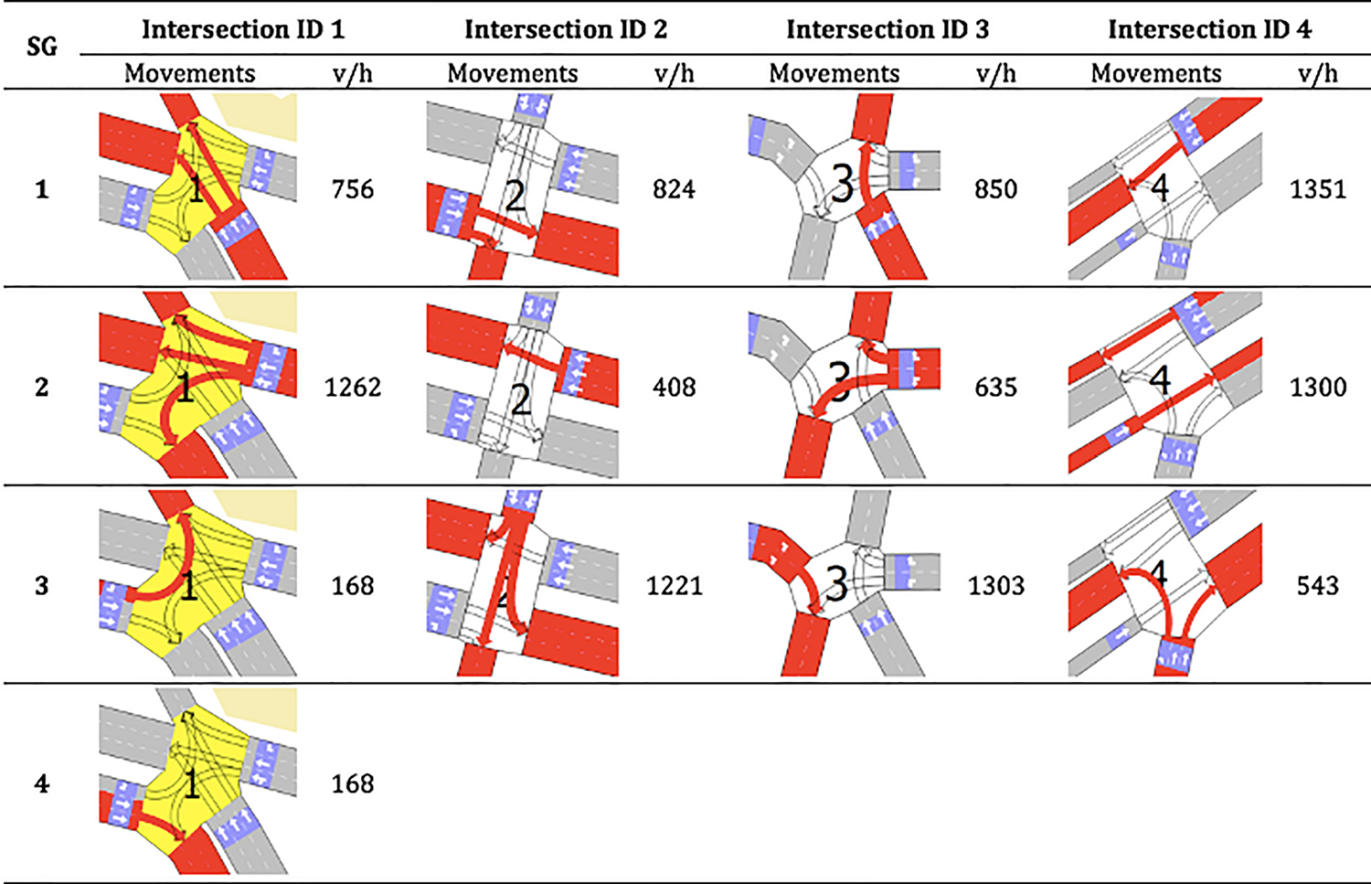
Characteristics of the movements in Floresta region.

The ACTS model aims to optimize the green times x for all phases in the traffic signals at the four intersections described in Table 2. Figure 4 shows the average flow of movements (given in vehicles per hour, v/h) of the intersections shown in Table 2. In Fig. 4, all movements controlled by each signal group (SG) are marked in red color.

Table 3 shows the movements allowed in each of the phases of the traffic signals at each intersection in Table 2. Each phase consists of a group of movements that can be activated in simultaneous splits because they represent non-conflicting movements of the semaphore groups shown in Fig. 4. In other words, movements in a given phase can share the same green time within a cycle time without risk of vehicle collision.

**Table 3:** Characteristics of the phases in Floresta region.

Phase	Intersection ID 1	Intersection ID 2	Intersection ID 3	Intersection ID 4
	Movements	Movements	Movements	Movements
1	1, 4	1, 2	1, 3	1, 2
2	2	3	2	3
3	3, 4			

BHTrans provided vehicle flow data for each movement for the four junctions in the region, from 08:00 to 10:00, at 5 min intervals, for a period of one month. These data were loaded into AIMSUN. The period used for the simulation is 2 h and at each end of the cycle time (i.e., 90 s) the simulator receives a new signal control plan from ACTS.

The implementation proposed in this article is written in Python (version 2.7.5). The AIMSUN simulator (version AIMSUN Next 8.2.2) has an Application Programming Interface (API) for Python, which facilitates the development of algorithms and their integration into simulations.

### Experimental results

**Figure 5:**
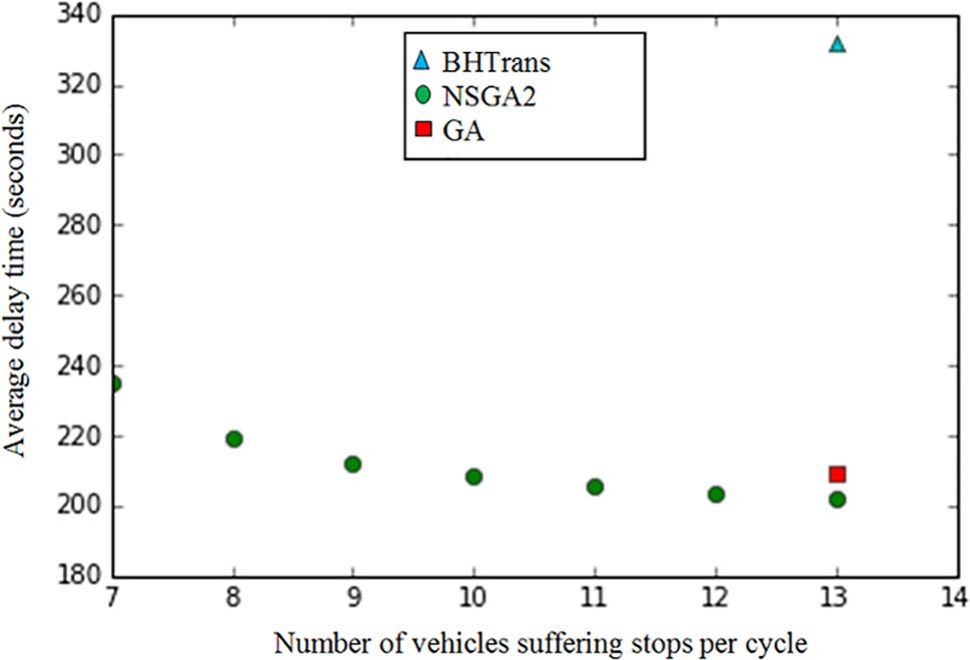
Comparison between NSGA2 and BHTrans solutions.

As mentioned above, the total simulation time was set to two hours, as this is more than enough time for the network to reach steady state. At each end of the cycle time (i.e., 90 s), the simulator receives a new signal control plan from the optimizer. It would be impractical to show here all the efficient solution sets generated by NSGA2, i.e., all 80 signal control plans generated by the algorithm. Therefore, this experiment was carried out by focusing on a signal control plan at a specific simulation interval as an example. So, for this signal control plan, the average vehicle delay *D* and the number of vehicles stops per cycle *NS* are calculated for all movements of the four intersections in the region at this time period. The result of the experiment is shown in Fig. 5.

Figure 5 shows the efficient set of solutions generated by NSGA2 (the average non-dominated solution frontier over 30 runs of the algorithm to satisfy the central limit theorem), compared with the solution obtained by the current traffic signal control plan used by the BHTrans transport company in the actual network and also with the best solution obtained by a mono-objective GA.

On this graph, it can be observed that:

- The best solution found by GA has the following values in the objective functions: D(x*) = *209* s and NS(x*) = *13*.
- A solution selected for comparison, from the set of NSGA2 solutions, has the following values in the objective functions: D(x*) = *203* s and NS(x*) = *13*. D represents the minimum average delay time solution obtained by NSGA2 and NS the number of stops.
- The solution chosen by NSGA2 was able to improve the average delay time by only *3%* and the number of vehicles suffering stops per cycle was 13, i.e., *520* vehicles suffering stops per hour in the whole controlled network.
- The NSGA algorithm was also able to find *7* efficient solutions, as shown in Fig. 5.

From this efficient solution set obtained, it is possible to see the advantages of a multi-objective approach compared to a mono-objective one, as it provides different non-dominated solutions to the problem. The multi-objective approach allows the traffic engineer to choose the desired signal control plan among the different optimal solutions obtained by the algorithm, depending on the situation and the objective to be prioritized at a given time.

When evaluating the current traffic signal control plan used by BHTrans, the average delay time found was *332* s and the number of vehicles stops per cycle was *13*. This shows that both NSGA2 was able to find better solutions to the problem, generating efficient signal control plans that resulted in more interesting values of the objective functions. Comparing the results with the BHTrans control plan, it can be seen that by using ACTS and NSGA2 optimized results in the network signal control plan, a reduction of about *39%* in the average delay time of vehicles can be achieved. This means that vehicles move through the network with almost half the average delay time and this can lead to a significant reduction in congestion on the network.

The ACTS method presented in this paper is not compared with other multi-objective approaches because, to the authors' knowledge, there is no other research that addresses this problem with an ACTS approach using the same set of objective functions.

## Discussions

Within this study, the ACTS model was proposed and implemented, which uses a mathematical model to estimate the average delay times and the number of vehicles stops per cycle. The well-known NSGA2 algorithm was used to find a set of signal control plans that optimize the chosen objective functions. NSGA2 was compared with the current fixed-time signal control plan used by BHTrans and with a mono-objective GA approach using micro-simulation, in order to find out if NSGA2 is efficient in optimizing the traffic signal timing for a region of the city of Belo Horizonte (Brazil). NSGA2 found a number of efficient solutions and reduced the average delay of vehicles in the region by almost half compared to the results obtained using the current signal control plan used by BHTrans.

Compared to some existing studies, this experimental result is a good improvement and contribution to the literature. For example, in the work of Costa et al.^4^, the authors were able to improve the traffic condition in 20 percent by their proposed traffic optimization model using a fixed time traffic control approach, and in the study of Leal et al.^15^, a reduction of 47 percent of the total average delay time of vehicles in the studied network was found, considering only one objective function in the optimization process.

In the technological context, this research has also contributed by proposing a model that is easy to apply in practice, since it does not depend on simulation to evaluate the solutions to the problem, which is an advantage of this work, since this is one of the major problems in approaches because it makes the search process slow and difficult to adapt and use in the real world.

Moreover, as reiterated by^2^, an important strategy used by most European countries to combat congestion is active traffic management. As shown in the literature review, active control is also a promising area to improve urban mobility in Brazil, but there is still a lack of public policies for mobility and investment culture in the country. Thus, another purpose of this work is to show the benefits of active control in reducing delays with real data from traffic of a Brazilian city and to start a discussion with BHTrans and other governmental organizations about future investments in this field. Active control strategy works to provide reliable trips, reduce recurring and nonrecurring congestion, and provide enhanced information to drivers.

Based on advances in technology and traffic management experience, this strategy works to make the best use of existing capacity and provide additional capacity during periods of congestion or incidents^19^.

## Conclusion

Finally, analysis of these results showed that CI techniques can be used to implement ACTS systems to provide more efficient traffic control. In addition, it was observed from the experiments that the increase in vehicle demand leads to a longer delay time using the BHTrans traffic solution. The analysis of these results demonstrates the ability to generalize and apply the model in the process of optimizing traffic light schedules, presenting better traffic control strategies.

This optimization of the traffic lights plans results in a greater fluidity of vehicles and, consequently, in the reduction of congestion and all the consequences provided by it in people’s lives. In addition, efficient traffic signal control can optimize the road infrastructure as a whole, avoiding the construction of heavy infrastructure in the region (such as viaducts, for example) and contributing to the reduction of pollutant emissions into the atmosphere.

For future studies, it is intended to use the proposed model with other intersection networks, to study other operators and parameter set values of NSGA2 in order to make improvements in the results, and also to test the ACTS model with other CI techniques.

### Supplementary Information

Supplementary Information.

## Data Availability

All data generated or analyzed during this study are included in this published article [and its supplementary information files].
