# Supplementary material for: Traffic light optimization using non-dominated sorting genetic algorithm (NSGA2)
Source: Sci Rep. 2023 Sep 20;13:15550. doi: 10.1038/s41598-023-38884-2 (PMC10511403; doi:10.1038/s41598-023-38884-2)
Supplement: Supplementary file 1 — Supplementary Information. [file 41598_2023_38884_MOESM1_ESM.zip › dadosBHTrans/calibrac+î-oa+î▌Æo do modelo/58160003.pdf]

# Genetic Algorithm for the Calibration of Vehicle Performance Models of Microscopic Traffic Simulators

André Luiz Cunha, José Elievam Bessa Jr., and José Reynaldo Setti

Universidade de São Paulo, São Carlos School of Engineering
Dept. of Transport Engineering
Av. Trabalhador São-carlense, 400 – S. Carlos, SP, Brazil – 13566-590
{acunha,jebessa}@sc.usp.br, jrasetti@usp.br

**Abstract.** A genetic algorithm was used to search for optimum calibration parameter values for the vehicle performance models used by two well-known microscopic traffic simulation models, CORSIM and Integration. The mean absolute error ratio between simulated and empirical performance curves was used as the objective function. Empirical data was obtained using differential GPS transponders installed on trucks travelling on divided highways in Brazil. Optimal parameter values were found for the "average" truck for each truck class and for each vehicle in the sample. The results clearly show the feasibility of the proposed approach. The simulation models calibrated to represent Brazilian trucks individually provided average errors of 2.2%. Average errors around 5.0% were found when using the average truck class parameters.

**Keywords:** Genetic algorithm, performance curve, traffic simulation.

## 1 Introduction

Microscopic traffic simulation models have become a very useful tool for planning, design and operation of transport systems. The greatest advantage of using such tools is the ability to evaluate alternatives prior to their implementation. However, the adequate use of simulation models requires calibration of the model parameters to ensure a proper representation of the real traffic behavior [1].

The accuracy of the results provided by a simulation model is highly dependent upon its calibration parameters [2]. The values adopted for those parameters are used by the model's internal logic to govern the movement of vehicles and driver behavior. The vehicle performance model is one of the logics used to represent the movement of vehicles and thus its travel speed along the modeled network. It is especially important in links where heavy vehicles climb steeper grades and other situations where the available engine power is fully utilized.

Most users simply use the default values for the vehicle performance calibration parameters. The accuracy of simulations involving networks in mountainous or hilly terrain with traffic containing a significant fraction of heavy vehicles can,

L. Seabra Lopes et al. (Eds.): EPIA 2009, LNAI 5816, pp. 3–14, 2009.
© Springer-Verlag Berlin Heidelberg 2009

however, be compromised and could be improved by a properly conducted calibration of the performance model for the typical trucks in the traffic stream. The calibration of such models, however, is not a simple task given the complexity of the models and the difficulties in obtaining optimal parameter values empirically. This paper proposes a genetic algorithm approach for this task and presents an application of the proposed approach to two widely different traffic simulation models based on truck performance data collected on Brazilian highways.

## 2 Traffic Simulation Models

Traffic simulation models are efficient and powerful tools for the operational analysis of traffic streams. Their main advantage is to allow for the evaluation of alternatives prior to their implementation or under conditions that would be very difficult to observe in the real world, without submitting the users to any inconveniences that such tests might cause [1,2]. Simulation models are now widely used for the planning, design and management of highway networks and intelligent transportation systems. For instance, in the development of procedures within the Highway Capacity Manual, traffic flow computer simulation was extensively used [3,4]. The use of simulation, however, requires an extensive knowledge about the model itself and its limitations, as well as an excellent understanding of the traffic flow theories upon which the model is based [5]. Two microscopic simulation models were used in this study: CORSIM, one of the most used traffic simulators, and Integration, which uses a more sophisticated vehicle performance model. The next section briefly discusses the relevant aspects of them.

### 2.1 CORSIM

CORSIM (CORridor SIMulation), developed by US FHWA in the late 1970s, consists of two integrated microscopic simulators, NETSIM, which simulates traffic flow on arterials and roads with interrupted flow, and FRESIM, which simulates freeways and other roads with uninterrupted flow [6]. It is one of models most used by practitioners and researchers, due to its ability to simulate all types of road, from freeways to local streets, subjected to all types of control, in addition to having a friendly user interface and high-quality graphical representation of the simulated traffic flow [7,8].

CORSIM is an stochastic model that assigns random attributes to drivers, vehicles and decision-making processes, allowing for a realistic representation of the road network [6]. Driver behavior is defined by random attributes (varying degrees of aggressiveness); vehicle performance (acceleration and speed) is randomly defined in a similar fashion. Vehicle movement in the network is based on the Pitt car-following behavior [9], which is based on the distance headway and speed differential between the leading vehicle and follower. The model updates position, speed and acceleration for all vehicles in the network every second.

Thus, vehicular locomotion is determined by the car-following and acceleration behavior.

CORSIM's heavy vehicle performance model is based on average accelerations that may be achieved based on the vehicle's instantaneous speed. The performance model adopted assumes fixed acceleration values for speeds ranging from 0 to 110 ft/s (120 km/h), for four truck types (light, medium, heavy and extra-heavy). The user manual does not explain how the default values were obtained. However, these values are stored in RT173 (*Maximum Acceleration Table*) and may be modified by the user to better reflect the performance of the trucks being simulated.

The calibration of this performance model requires the collection of speed and acceleration data for trucks representative of the four truck types. Compared to Integration's, this approach is less sophisticated, but is computationally less intensive – something that made sense at the time the model was originally developed.

### 2.2 Integration

Integration is a model capable of simulating from freeways to local roads using the same logic. Created by Michel Van Aerde in the late 1980s, Integration is not as widely used as CORSIM but is widely recognized as a very sophisticated model capable of modelling a wide range of traffic conditions [10]. While Integration is a microscopic simulation model, its car-following model parameters can be calibrated macroscopically, from speed-flow data [11]. Vehicle movement along the links is controlled by three logics: car-following, lane-changing and acceleration (as a function of vehicle performance). Vehicle position, speed and acceleration are updated every 0.1 s.

Integration stores parameters for its vehicle performance model in a file called `maxi_acc.dat`. The logic calculates acceleration from the resultant of the forces acting on the vehicle and assumes that engine power is constant. The acceleration $a$ is

$$a = \frac{F - R}{m} (\mathrm{m/s^2}), \tag{1}$$

where $F$ is the tractive force (N), $R$ is the total resistance (N) and $m$ is the vehicle mass (kg). The tractive force $F$ (N) produced by the engine is

$$F = \min \begin{cases} 3600\,\eta\,P/V \\ W_{ta}\,\mu \end{cases} (\mathrm{N}), \tag{2}$$

where $\eta$ is the transmission efficiency ($0 \leq \eta \leq 1$); $P$ is the nominal engine power (kW); $V$, the vehicle speed; $W_{ta}$, the weight on the tractive axles (N); and $\mu$ the tyre-pavement friction. The total resistance $R$ is the sum of three components – drag ($R_a$), rolling resistance ($R_r$) and grade resistance ($R_g$):

$$\begin{aligned} R &= R_a + R_r + R_g \text{ (N)}, \quad \text{with} \\ R_a &= c_1\,C_D\,C_h\,A\,V^2 \text{ (N)} \\ R_r &= C_r\,(c_2\,V + c_3)\,W/1000 \text{ (N)} \\ R_g &= W\,i \text{ (N)} \end{aligned} \tag{3}$$

where $c_1$ is constant and equal to 0.047285; $c_2$ and $c_3$ are constants reflecting the tyre characteristics; $C_D$ is the drag coefficient; $C_h$ is the correction due to altitude; $A$ is the cross-section area (m$^2$); $V$, the speed (km/h); $C_r$ is the rolling resistance coefficient; and $i$ is the grade (m/m).

Due to the number of parameters involved, calibration of this model is more complicated than CORSIM's. The complexity of the calibration process and the number of calibration parameters involved suggests that a genetic algorithm would be an efficient approach to the calibration of these models, perhaps with an edge over other possible approaches, as the literature suggested [1,2,12,13]. The next sections present a brief review on the use of genetic algorithms for calibration of simulation models.

## 3 Calibration of Traffic Simulation Models Using Genetic Algorithm

Calibration is the process through which the user fine tunes model parameters, compares simulation results to empirical data and verifies the ability of the simulation model to represent adequately the observed traffic stream [2,12,14]. Model developers provide default values for model parameters that reflect the data used in the validation of the software. Users applying the model to other regions should ideally recalibrate model parameters to improve the representation of local vehicle and driver peculiarities by the simulation.

Genetic algorithm (GA), which were first created by Holland [15], is a search method based on the principles of evolution and natural selection. One of its advantages is to search for solutions from multiple points, increasing the probability of finding a global, instead of a local, optimum [1]. Thus, it is particularly useful for problems with a complex search space – as in the case of traffic flow simulation. GAs have been successfully applied to many aspects of transportation engineering: traffic flow simulation modelling [1,2,12,13,16,17], traffic signal timing [18] and even infrastructure maintenance planning [19].

GAs simulate the process of natural evolution, with the possible solutions (in this case, the set of calibration parameter values) representing individuals in a population. An initial population is randomly generated, with the individual level of adaptation to the environment (the quality of the solution) measured by a fitness function. Individuals are represented by strings – called chromosomes – which represent the individual characteristics, genes. Each gene represents one of the calibration parameters.

The search consists in creating successive generations of individuals. At each new generation, new individuals are created from the previous generation using selection and reproduction processes to exchange genetic material. One of these processes, elitism, consists in selecting the individuals who are best adapted to the environment for reproduction, to assure that the best traits are propagated to their descendants. Other genetic operators, predation and mutation, are used to introduce variability (new solutions) into the gene pool. The former consists in replacing the less adapted individuals in the population by new chromosomes,

randomly generated. Mutation is used to randomly modify some of the genes in a population.

A literature survey shows that most of the previous applications of GA to traffic flow simulation models have focused on the calibration of car-following parameters [9,13,16,20,21]. Schultz and Rilett [16], however, emphasize that the use of default parameters for the performance model might affect the quality of the simulation results for networks with a large number of trucks, as it is the case in Brazil. The need for a method to calibrate the vehicle performance models used by CORSIM and Integration was identified. The next section explains the adopted approach.

## 4 The Proposed Approach

The proposed approach consisted in using a GA to find the best values for the parameters of CORSIM and Integration performance models using empirical performance data collected on divided highways. The accuracy of the simulation was evaluated comparing empirical and simulated performance curves (speed vs. distance travelled along a grade). Initially, the GA was used to find parameter sets to best represent the performance each truck observed during the data collection. This step was the validation of the process. Subsequently, the GA was modified to obtain parameter sets representative of the performance of typical vehicles for four classes: light, medium, heavy and extra-heavy trucks.

### 4.1 Field Data Collection

Data for the observed performance curves were collected on a divided highway in the state of São Paulo, Brazil. Total truck mass, nominal engine power, as well as axle number and configuration data were collected at a mobile weigh station. With the agreement of the driver, a differential GPS receiver was installed in the truck to collect kinematic speed and position data. The trucks were monitored along a section of approximately 10 km. The GPS data provided vertical and horizontal alignment and truck speed at 1-s intervals. From this data, performance curves were constructed for all vehicles in the sample.

Trucks were grouped in 4 classes according to the number of axles: light (two-axle rigid trucks), medium (three-axle rigid trucks), heavy (five- and six-axle articulated trucks) and extra-heavy (seven- to nine-axle articulated trucks). The sample consisted of 62 trucks (light: 5; medium: 13; heavy: 22; extra-heavy: 22) travelling on grades varying from 0.6% to 5.2%, although most of the observations were made on 1.8% (22 trucks), 2.9% (13 trucks) and 3.4% (14 trucks) grades.

### 4.2 Genetic Algorithm

The genetic algorithm was coded in Microsoft Excel's Visual Basic for Application. Figure 1 shows a simplified flowchart of the GA's main components. In the next sections the main features of the GA are explained.

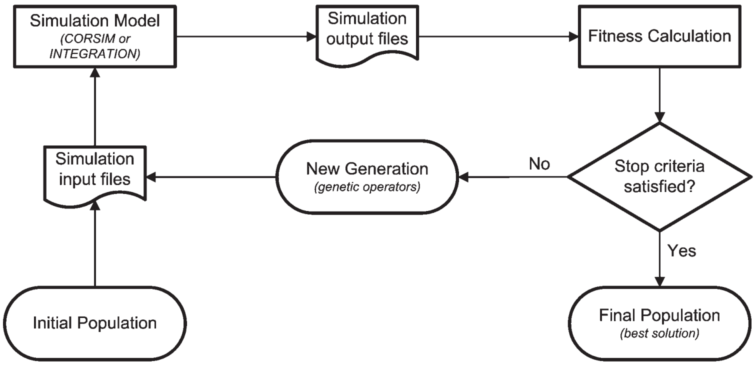


**Fig. 1.** Simplified flowchart of the genetic algorithm used

**Initial Population.** An initial population of 40 individuals was used. The population size was chosen after a sensitivy analysis using light trucks, whose results suggested that the best combination would be 40 individuals and 50 generations.

Each individual in the population is a string (chromosome) containing model parameter values (genes). Parameter values for the initial population were generated randomly, within predefined search space limits. For CORSIM, the genes are the acceleration for 10 ft/s speed intervals, from 0 to 110 ft/s, and search spaces are shown in Table 1. The search spaces were established assuming that the upper and lower limits should follow a monotone decrescent function. Special care was also taken to avoid acceleration ranges that could result in the truck not being able to climb a steep grade.

**Table 1.** Search space for CORSIM calibration parameters

| | Speed (ft/s) | | | | | | | | | | |
|---|---|---|---|---|---|---|---|---|---|---|---|
| Acceleration (ft/s$^2$) | 0 | 10 | 20 | 40 | 50 | 60 | 70 | 80 | 90 | 100 | 110 |
| Minimum | 2 | 1.5 | 1 | 0 | 0 | 0 | 0 | 0 | 0 | -1 | -1 |
| Maximum | 20 | 15 | 10 | 6 | 4 | 3.5 | 3 | 2.5 | 2 | 1.5 | 1.5 |

The chromosome used for Integration's calibration was made of 8 genes, containing the parameters listed in Table 2, which also lists the search spaces for each truck class. The search spaces for mass $W$ and nominal engine power $P$ were defined as the confidence interval ($\alpha = 5\%$) for the sample mean, for each class. Search spaces for the other parameters were defined from the literature [11]. An additional parameter, $\eta_2$, was included in the chromosome to represent driver aggressiveness and is a multiplier of the nominal power.

**Simulation Input Files.** The next step is the preparation of the input files for the simulation of each chromosome. For CORSIM, a `.trf` file is created, in

**Table 2.** Search spaces for Integration calibration parameters

| | Truck class | | | |
|---|---|---|---|---|
| Parameter | Light | Medium | Heavy | Extra-heavy |
| $W$ (kg) | 5,998 – 12,510 | 19,222 – 23,464 | 42,164 – 44,074 | 58,844 – 59,401 |
| $P$ (kW) | 95 – 110 | 115 – 158 | 252 – 264 | 281 – 292 |
| $\eta$ | 0.7000 – 0.9000 | 0.7000 – 0.9000 | 0.7000 – 0.9000 | 0.7000 – 0.9000 |
| $C_D$ | 0.5000 – 12.000 | 0.5000 – 12.000 | 0.5000 – 12.000 | 0.5000 – 1.2000 |
| $c_2$ | 0.0100 – 0.0500 | 0.0100 – 0.0500 | 0.0100 – 0.0500 | 0.0100 – 0.0500 |
| $c_3$ | 20.000 – 90.000 | 2.0000 – 90.000 | 2.0000 – 90.000 | 2.0000 – 9.0000 |
| $C_r$ | 10.000 – 20.000 | 1.0000 – 20.000 | 1.0000 – 20.000 | 1.0000 – 2.0000 |
| $\eta_2$ | 0.5000 – 15.000 | 0.5000 – 15.000 | 0.5000 – 15.000 | 0.5000 – 1.5000 |

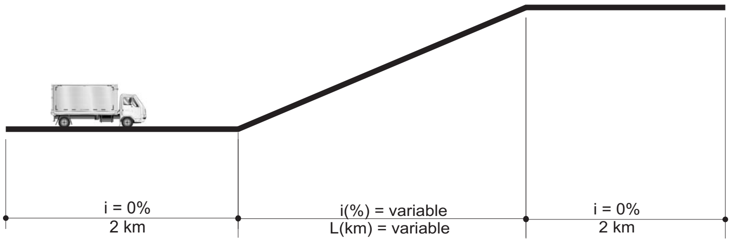


**Fig. 2.** Vertical alignment of the simulated road network

which the line corresponding to RT173 contains the parameter values for that individual. For Integration, a `maxi_acc.dat` file is created for each individual in the population, containing the corresponding parameter values.

**Simulation Runs.** In this step, the model is called to run the simulation for each chromosome in the population. The simulation experiments used an hipotetical road section composed of three segments, as shown in Figure 2: an initial 2-km level segment, an intermediate segment with variable grade and length, and a final 2-km level segment. The initial segment was used to adjust the vehicle speed to the initial speed of the observed truck. This assured that both the simulated and observed performance curves had the same entry speed. The grade magnitude for the second segment was set to the observed grade magnitude. The simulated traffic flow was set to 10 veic/h to avoid interaction between vehicles and the consequent effects of the car-following logic on the acceleration. The simulation time used was 30 min and just one type of truck was used in each simulation. The simulation results are then used in the next step.

**Simulation Output Files.** The simulation results are used to create performance curves, which were built using instantaneous speeds at every 100 m along the grade. The data were extracted from the simulation output files, `.tsd` for CORSIM and `detector.out` for Integration. At the end of this step, the observed and simulated performance curves were available to be compared.

**Fitness Calculation.** In this step, the quality of the solution provided by each chromosome is evaluated using a fitness function which, in this case, was the mean absolute error ratio (*MAER*). The fitness function measures the chromosome's ability to represent faithfully the behavior of all trucks in a class and is given by:

$$MAER_{jk} = \frac{1}{N_k} \sum_{l=1}^{N_k} IMAER_{jkl} \tag{4}$$

in which $MAER_{jk}$ is the average *MAER* for the $k$-th truck class using chromosome $j$; and $N_k$ is the number of trucks in the $k$-th truck class. $IMAER_{jkl}$ is a measure of the discrepance between the simulated and observed speeds, which are determined at every 100 m along the grade:

$$IMAER_{jkl} = \frac{1}{m} \sum_{i=0}^{m} \left| \frac{VSIM_{kl}(i) - VOBS_{kl}(i)}{VOBS_{kl}(i)} \right| \tag{5}$$

in which $IMAER_{jkl}$ is the *MAER* for $l$-th truck in the $k$-th truck class using chromosome $j$; $VSIM_{kl}(i)$ is the simulated speed at the $i$-th station along the grade for the $l$-th truck in the $k$-th class; $VOBS_{kl}(i)$ is the observed speed at the $i$-th station along the grade for the $l$-th truck in the $k$-th class; and $m$ is the number of speed observation stations along the grade. The values of the observed speed used to calculate *IMAER* were "raw" — i.e., they were not filtered to remove any observational noise.

The smaller $MAER_{jk}$ is the better the quality of the solution provided by chromosome $j$; the greater $MAER_{jk}$ is the poorer the solution provided by this parameter set.

**Stop Criteria.** Two stop criteria were adopted for the GA used to find the calibration parameters for truck classes: $MAER \leq 3.0\%$ or 50 generations. For the calibration for individual trucks in the sample, the criteria were $MAER \leq 0.1\%$ or 10 generations. Once one of these criteria is reached, the best solution is provided by the chromosome with the smallest *MAER*.

**New Generation.** If the stop criteria is not satisfied, a new generation is created from the current population, through the use of genetic operators. Different strategies were adopted for each model. The one used for CORSIM was more aggressive, given the paucity of information about its performance logic and how the default values were found [16]. For Integration, however, a less agressive strategy was utilized because ranges of values for the parameters used in its performance model can be easily found in the literature [11].

For CORSIM, at the end of each generation, *predation* is used to cull from the gene pool that half of the population with the highest *MAER*. These least fit individuals are replaced by randomly generated chromosomes. Next, the best fit individual of that generation – the chromosome with the smallest *MAER* – is selected (*elitism*) to exchange genetic material with the remainder of the

population (*crossover*). The chromosomes thus created are then subjected to *mutation*, which is a random change in one of the genes; the mutation rate used was 30%. The resulting population is the new generation, which is subjected to all steps until one of the stop criteria is satisfied.

For Integration, elitism was used to select the chromosome to crossover genetic material with the remainder of the population. The main difference is that predation and mutation are applied only after five generations. The mutation rate used was 10% and the predation rate, 30%. The new generation is made of the chromosomes thus obtained and the process repeats until one of the stop criteria is met.

## 5 Analysis of the Results

Initially, the GA was used to obtain calibration parameters for each truck observed, to validate the proposed approach. The calibration results are illustrated in Figure 3, which shows the simulated (GA individual) and "raw" observed performance curves (GPS) for one of the heavy trucks in the sample. Also shown in the figure is the simulated performance curve using the calibration parameters obtained for the heavy truck class (GA category), which are discussed next.

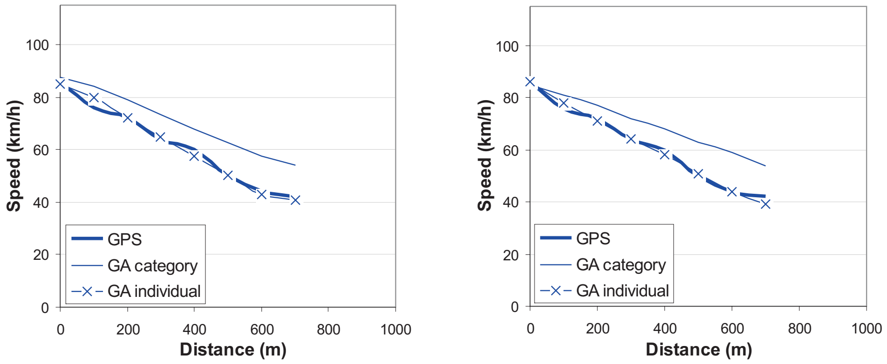


**Fig. 3.** Simulated and unfiltered observed performance curves for one of the heavy trucks in the sample (CORSIM on the left; Integration on the right)

The histograms in Figure 4 show the distribution of $MAER$ found in the calibration of each model. $MAER$ was greater than 5% in only 7 of the 62 trucks observed for the CORSIM calibration; for Integration, 5 trucks had $MAER$ greater than 5%, which clearly demonstrates the efficiency of the proposed approach.

### 5.1 Calibration for Truck Classes

Once the proposed approach was tested and validated, the GA was used to find calibration parameter sets for typical trucks that would represent each of

the four truck classes adopted. The results for this are summarized in Figure 5, which shows the improvement obtained with the use of the new parameter values to replace the default values. It can be easily noticed that the default values could not simulate the performance of Brazilian trucks adequately and that the models recalibrated using the GA are able to provide better results. The recalibrated parameters are shown in Tables 3 and 4, which show the best results (least $MAER$) produced by the GA. The last generation, however, had several individuals that provided solutions only marginally worse than the best, for both CORSIM and Integration.

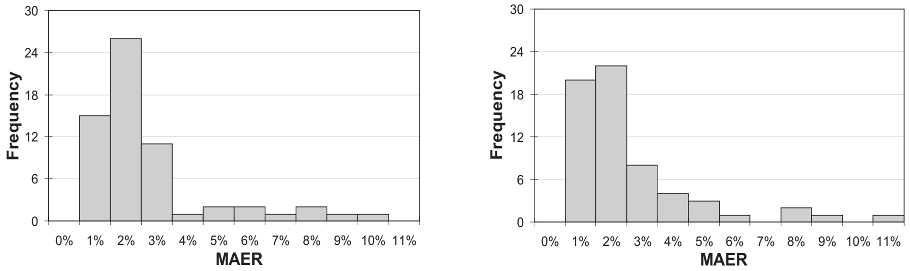


**Fig. 4.** Mean absolute error ratio distributions found in the calibration of the models (CORSIM on the left; Integration on the right)

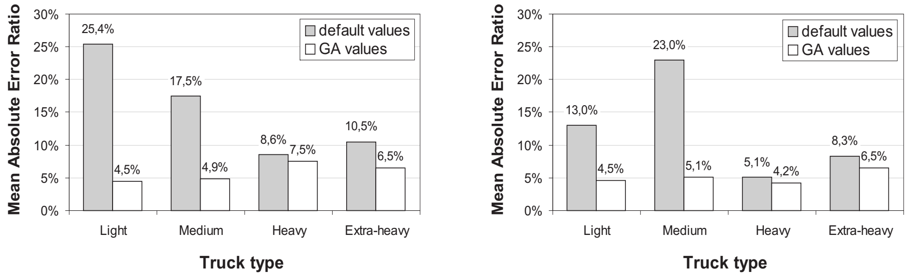


**Fig. 5.** Mean absolute error ratios disaggregated by truck class, before and after the calibration of the models (CORSIM on the left; Integration on the right)

**Table 3.** New values found for Integration's performance model parameters by the GA

| Truck class | $W$ (kg) | $P$ (kW) | $\eta$ | $C_D$ | $c_2$ | $c_3$ | $C_r$ | $\eta_2$ |
|---|---|---|---|---|---|---|---|---|
| Ligth | 11,383 | 106.1 | 0.7486 | 0.6632 | 0.0150 | 3.1638 | 1.6049 | 1.0372 |
| Medium | 21,974 | 142.5 | 0.7227 | 0.7584 | 0.0212 | 2.5301 | 1.3168 | 0.7153 |
| Heavy | 43,954 | 253.8 | 0.7515 | 0.8217 | 0.0108 | 5.1679 | 1.8076 | 1.1992 |
| Extra-heavy | 59,076 | 284.9 | 0.8062 | 0.8210 | 0.0426 | 3.6663 | 1.2803 | 0.7507 |

**Table 4.** New values of acceleration (ft/s$^2$) found for CORSIM by the GA

| | Truck class | | | |
|---|---|---|---|---|
| Speed (ft/s) | Light | Medium | Heavy | Extra-heavy |
| 0 | 12.56 | 12.95 | 11.76 | 16.29 |
| 10 | 10.25 | 4.32 | 6.36 | 1.68 |
| 20 | 5.76 | 1.94 | 3.11 | 1.60 |
| 30 | 3.09 | 0.65 | 2.30 | 0.75 |
| 40 | 2.24 | 0.35 | 1.79 | 0.50 |
| 50 | 1.20 | 0.35 | 0.84 | 0.48 |
| 60 | 0.73 | 0.28 | 0.54 | 0.35 |
| 70 | 0.31 | 0.23 | 0.46 | 0.06 |
| 80 | 0.27 | 0.02 | 0.45 | 0.00 |
| 90 | 0.15 | 0.02 | 0.06 | 0.01 |
| 100 | −0.05 | 0.01 | 0.02 | −0.84 |
| 110 | −0.13 | −0.10 | −0.10 | −0.97 |

## 6 Concluding Remarks

This research has shown that a GA can be used to calibrate the performance models used by two traffic flow simulators, CORSIM and Integration. Specifically, new parameters were found for Brazilian trucks using empirical speed data collected with a differential GPS. Furthermore, the GA was able to find parameters to represent the average performance of trucks in four classes: light, medium, heavy and extra-heavy. When using truck class parameters found by the GA, the differences between observed and simulated speeds, measured as the mean absolute error ratio, for trucks climbing grades from 0.6% to 5.2% ranged from 4.5% (light trucks) to 7.5% (heavy trucks) for CORSIM. For Integration, the differences ranged from 4.2% (heavy trucks) to 6.4% (extra-heavy trucks).

The vehicle performance models used in CORSIM and Integration, while very different, proved capable to represent the climbing performance of most trucks in the sample with high fidelity. When using parameters found by the GA for individual trucks, the difference between simulated and observed speeds was greater than 5% for only 7 of the 62 trucks for CORSIM and 5 out of 62, for Integration. The average $MAER$ was 2.2%.

**Acknowledgments.** The research reported in this paper was funded by CNPq, the Brazilian National Council for Scientific and Technological Development. The support given by Centrovias Sistemas Rodoviários S.A. and its personnel during the data collection is also gratefully acknowledged.

## References

1. Kim, K.-O., Rilett, L.R.: Genetic-Algorithm-Based Approach for Calibrating Microscopic Simulation Models. In: 2001 IEEE Intelligent Transportation Systems Conference Proceedings, pp. 698–704. IEEE Press, New York (2001)

2. Ma, T., Abdulhai, B.: Genetic Algorithm-Based Optimization Approach and Generic Tools for Calibrating Traffic Microscopic Simulation Parameters. Transp. Res. Rec. 1800, 6–15 (2001)
3. Elefteriadou, L., Torbic, D., Webster, N.: Development of Passenger Car Equivalents for Freeways, Two-Lane Highways, and Arterials. Transp. Res. Rec. 1572, 51–58 (1997)
4. Harwood, D., May, A., Anderson, I., Leiman, L., Archilla, R.: Capacity and Quality of Service of Two-Lane Highways. NCHRP, Transportation Research Board (1999)
5. Halati, A., Lieu, H., Walker, S.: CORSIM – Corridor Traffic Simulation Model. In: Traffic Congestion and Traffic Safety in the 21st Century, pp. 570–576. ASCE, Chicago (1997)
6. CORSIM User's Guide. Federal Highway Administration, U.S. Department of Transportation, Washington, D.C. (2001)
7. Owen, L.E., Zhang, Y., Rao, L., McHale, G.: Traffic Flow Simulation Using CORSIM. In: Proc. of the 2000 Winter Simulation Conference, pp. 1143–1147 (2000)
8. Milam, R.T., Choa, F.: Recommended Guidelines for the Calibration and Validation of Traffic Simulation Models. In: 8th TRB Conference on the Application of Transportation Planning Methods, pp. 178–187. Transportation Research Board, Washington (2002)
9. Rakha, H., Crowther, B.: Comparison and Calibration of FRESIM and INTEGRATION Steady-state Car-following Behavior. Transp. Res. A 37, 1–27 (2003)
10. May, A.D.: Traffic Management from Theory to Practice: Past, Present, Future. Transp. Res. Rec. 1457, 1–14 (1994)
11. Demarchi, S.H.: Heavy Vehicle Effects on Capacity and Level of Service of Divided Highways (in Portuguese). Ph.D. thesis, Univ. de São Paulo, Brazil (2000)
12. Egami, C.Y., Setti, J.R., Rilett, L.R.: Calibration of a Two-Lane Highway Traffic Simulator Using Genetic Algorithm (in Portuguese). Transportes 12, 5–14 (2004)
13. Cheu, R., et al.: Calibration of FRESIM for Singapore Expressway Using Genetic Algorithm. J. Transp. Engg. 124, 526–535 (1998)
14. Hellinga, B.R.: Requirements for the Calibration of Traffic Simulation Models. In: Proc. of the Canadian Society for Civil Engineering, vol. IVb, pp. 211–222 (1998)
15. Goldberg, D.E.: Genetic Algorithms in Search, Optimization and Machine Learning. Addison-Wesley, Reading (1989)
16. Schultz, G.G., Rilett, L.R.: Calibration of Distribution of Commercial Motor Vehicle in CORSIM. Transp. Res. Rec. 1934, 246–255 (2005)
17. Araújo, J.J., Setti, J.R.: Analysis of Heavy-Vehicle Impacts on a Bridge Using Microsimulation (in Portuguese). In: Transporte em Transformação XII, pp. 23–42. Positiva, Brasília (2008)
18. Teklu, F., Sumalee, A., Watling, D.: A Genetic Algorithm Approach for Optimizing Traffic Control Signals Considering Routing. Computer-Aided Civil and Infrastructure Engineering 22, 31–43 (2007)
19. Liu, C., Hammad, A., Itoh, Y.: Maintenance Strategy Optimization of Bridge Decks Using Genetic Algorithm. J. Transp. Engg. 123, 91–100 (1997)
20. Payne, H.J., et al.: Calibration of FRESIM to Achieve Desired Capacities. Transp. Res. Rec. 1591, 23–30 (1997)
21. Chundury, S., Wolshon, B.: Evaluation of CORSIM Car-Following Model by Using Global Positioning System Field Data. Transp. Res. Rec. 1710, 114–121 (2000)
