# Supplementary material for: Traffic light optimization using non-dominated sorting genetic algorithm (NSGA2)
Source: Sci Rep. 2023 Sep 20;13:15550. doi: 10.1038/s41598-023-38884-2 (PMC10511403; doi:10.1038/s41598-023-38884-2)
Supplement: Supplementary file 1 — Supplementary Information. [file 41598_2023_38884_MOESM1_ESM.zip › dadosBHTrans/calibrac+î-oa+î▌Æo do modelo/Arquivos/AG-Micro/Instrucoes_AG_AIMSUN_by Elievam.pdf]

UNIVERSIDADE DE SÃO PAULO
ESCOLA DE ENGENHARIA DE SÃO CARLOS

Departamento de Transportes
Avenida Trabalhador Sãocarlense, 400
13566-590 – São Carlos, SP
Fone: (16) 3373-9601
Fax: (16) 3373-9602

Algoritmo genético para calibração do simulador AIMSUN

Instruções para utilização

Autor: MSc. José Elievam Bessa Jr.
Orientador: Prof. Dr. José Reynaldo Setti
Instituição: Universidade de São Paulo
Escola de Engenharia de São Carlos
Laboratório de Simulação de Sistemas de Transportes

São Carlos, abril de 2013

1. Introdução

Este documento tem como meta fornecer instruções para utilização de um Algoritmo Genético (AG) desenvolvido com o intuito de calibrar o simulador AIMSUN. O AG foi criado no Laboratório de Simulação de Sistemas de Transportes (LabSim) do Departamento de Engenharia de Transportes da Escola de Engenharia de São Carlos (STT-EESC-USP). O projeto foi capitaneado pelo aluno de doutorado José Elievam Bessa Júnior em parceria com outros estudantes e seu orientador, Prof. José Reynaldo Setti. A utilização do AG em trabalhos acadêmicos é permitida, desde que o LabSim seja mencionado como desenvolvedor do algoritmo.

2. Arquivos de entrada do AG

O AG é composto dos seguintes arquivos, que são detalhados em seções subsequentes:

- **AG_AIMSUN_v4.0.py**: código que consiste no AG para calibração do AIMSUN (programado em Python);
- **principal.txt**: contém as principais informações para funcionamento do algoritmo, como o número de gerações e o tamanho da população;
- **travel_time.txt**: contém dados sobre o tempo de viagem observado em campo, que servirá para cálculo da função de aptidão (*fitness*) de cada solução (cromossomo) do AG.
- **group_section.txt**: descrição dos IDs das seções que foram agrupadas para formar, por exemplo, corredores viários.
- **parametros.txt**: indica quais parâmetros do AIMSUN serão calibrados e quais valores *default* serão usados caso não sejam escolhidos para calibração;
- **dist_default.txt**: determina os valores *default* de parâmetros relacionados com as características veiculares e que são baseados em distribuições de frequência. Para cada parâmetro, devem ser informados a média, o desvio padrão e os valores máximo e mínimo da distribuição de frequência correspondente.
- **parametros_calibracao - descricao.xls**: planilha que descreve as siglas dos parâmetros do AIMSUN possíveis de serem calibrados. Muita atenção na descrição desses parâmetros, como o tempo de reação variável (por veículos) e as distribuições de parâmetros das características veiculares, como a aceleração máxima.
- **Execute-AG.bat**: Arquivo executável para chamar o AG e executá-lo.

Os nomes desses arquivos não devem ser alterados (exceto o arquivo *.xls e *.bat), assim como o conteúdo das duas primeiras linhas dos arquivos *.txt. A primeira coluna dos arquivos “principal.txt” e “pa-

râmetros.txt” também não devem ser alteradas. Todos esses documentos devem ser colados no diretório onde se encontra o arquivo de entrada *.ang do AIMSUN com a rede viária a ser calibrada. Todas as escolhas da rede devem ser feitas previamente, como, por exemplo, quais os modelos de *car-following* e de mudança de faixas devem ser calibrados, e quais tipos de veículos serão utilizados.

2.1. AG_AIMSUN_v4.0.py

O AG foi codificado em Python e, para isso, a versão 2.6.2 deve ser instalada (http://www.python.org/download/releases/2.6.2/). O arquivo de instalação do Python chama-se “python-2.6.2.msi” (instalar no diretório “C:/Python26”). Além disso, deve-se abrir esse código ou no Bloco de Notas ou no editor instalado junto com o Python (chamado “IDLE (Python GUI)”) e modificar as linhas **diretorio = "C:/Elievam/AG_AIMSUN_PYTHON/"** e **network = "Rede.ang"** para o diretório onde se encontra o arquivo a ser calibrado e a rede viária.

2.2. principal.txt:

A primeira coluna desse arquivo indica algumas informações importantes:

- **Experiment_ID**: código identificador do experimento, presente no arquivo *.ang;
- **Veh_types_IDs[car,bus,truck]**: IDs dos tipos de veículos a serem calibrados (máximo = 3);
- **Tipo_fitness**: se o valor é igual a 1, será usado como *fitness* o inverso da média dos erros percentuais entre os tempos de viagem observados em campo e simulados de cada corredor ou seção viária; se o valor é igual a 2, o *fitness* será o inverso do somatório das diferenças ao quadrado dos tempos de viagem observados em campo e simulados. Nos dois tipos de *fitness*, é calculada a média dos erros de cada corredor/seção das replicações (veja Equações 1 e 2);
- **Erro_min(%)**: caso o “Tipo_fitness” seja igual a 1, um critério de parada adicional pode ser usado, à ser determinado neste campo como um erro percentual mínimo obtido.
- **Replicacao_ID**: código identificador da replicação a ser utilizada no AG, informada no arquivo *.ang;
- **Replicacoes**: informa as sementes a serem usadas nas replicações, que indica o número de replicações desejadas;
- **Populacao**: tamanho da população (é o total de cromossomos, que representam soluções);
- **Geracoes**: número máximo de gerações, que serve como critério de parada do AG;
- **Taxa_mutacao**: porcentagem dos genes da população que sofrerão mutação quando for desejado inserir diversidade;

- **Taxa_predacao**: porcentagem dos indivíduos da população que serão predados (os piores) quando for desejado inserir diversidade;
- **Diversidade**: indica que será inserida diversidade (mutação e predação) se a geração corrente for múltipla de "Diversidade";

$$F(I) = \frac{1}{D}, \tag{1}$$

$$D = \frac{1}{M} \cdot \sum_{i=1}^{M} EMR(i), \tag{2}$$

podendo os desvios (ou erros) serem representados ou pela diferença média das medidas de desempenho:

$$EMR(i) = \frac{1}{N} \cdot \sum_{j=1}^{N} \left| \frac{OBS(i,j) - SIM(i,j)}{SIM(i,j)} \right|. \tag{3}$$

ou pelo somatório do quadrado dessas diferenças:

$$EMR(i) = \sum_{j=1}^{N} \left(OBS(i,j) - SIM(i,j) \right)^2, \tag{4}$$

em que $F(I)$ é o *fitness* do cromossomo I; M é o total de replicações com diferentes sementes de números aleatórios; e N é o total de seções ou trechos (que são conjunto de seções) observados em campo e simulados com o AIMSUN. $OBS(i,j)$ e $SIM(i,j)$ representam uma medida de desempenho (tempo de viagem) observada e simulada de uma seção j, respectivamente.

2.3. travel_time.txt:

A primeira coluna desse arquivo não é utilizada pelo AG, é apenas informativo para o usuário. A segunda coluna mostra os tempos de viagem (em segundos) observados em campo para cada corredor (que é um grupo de seções) ou para cada seção. A ordem das linhas não é relevante para o AG. As duas primeiras linhas desse arquivo não devem ser alteradas.

2.4. group_section.txt

Cada linha desse arquivo representa um grupo de seções, como um corredor viário. Em cada grupo, devem ser informados os IDs das seções que representam esse corredor viário. Se não houver grupos de seções, mas deseja-se avaliar cada seção individualmente, haverá apenas uma coluna em "IDs das secoes" com o ID de cada seção avaliada. A primeira coluna é apenas informativo para o usuário. A ordem das linhas desse arquivo deve ser a mesma do arquivo "travel_time.txt".

2.5. dist_default.txt

O arquivo dist_default.txt determina os valores *default* de parâmetros relacionados com as características veiculares e que são baseados em distribuições de frequência. Como citado anteriormente, para cada pa-

râmetro, devem ser informados a média, o desvio padrão e os valores máximo e mínimo da distribuição de frequência correspondente.

Para diminuir o esforço de calibração, somente a média da distribuição será passível de ajuste pelo AG. Os demais (desvio padrão, mínimo e máximo) são obtidos de acordo com a seguinte lógica, pensada com o intuito de manter o coeficiente de variação *default*:

```
se media_default ≠ 0, então:
    desvio = desvio_default * (media / media_default)
senão:
    desvio = desvio_default


se mínimo_default < (media - 2 * desvio):
    mínimo = mínimo_default
senão:
    mínimo = média - 2 * desvio


se máximo_default > (media + 2 * desvio):
    máximo = máximo_default
senão:
    máximo = média + 2 * desvio

se mínimo < 0:
    mínimo = 0
```

2.6. parametros.txt / parametros_calibracao - descricao.xls

O arquivo “parametros.txt” contém 4 colunas:

- **Parametro**: contém as siglas de todos os parâmetros do AIMSUN que podem ser calibrados; as descrições desses parâmetros encontram-se no arquivo “parametros_calibracao - descricao.xls”;
- **valor default**: esse valor será usado no referido parâmetro do AIMSUN caso ele não seja escolhido para calibração pelo AG;
- **calibracao [sim = 1; nao = 0]**: identificador que indica se o parâmetro será calibrado pelo AG. Caso o valor seja 1, o parâmetro será calibrado. Caso seja 0, será usado o valor presente na coluna “valor default”;
- **range_minimo**: valor mínimo do intervalo de busca do parâmetro do AIMSUN, caso ele tenha sido escolhido para calibração; e
- **range_maximo**: valor máximo do intervalo de busca do parâmetro do AIMSUN, caso ele tenha sido escolhido para calibração.

3. Executar o AG

Após determinar valores nos arquivos do AG, deve-se editar o arquivo “Execute-AG.bat”, salvar e clicar duas vezes nele para rodar o AG. Um arquivo chamado “geracoes.txt” será produzido com as seguintes colunas:

- geracao e individuo: indica a geração e o indivíduo testado;
- Valores dos parâmetros de calibração de determinado cromossomo (ver siglas dos parâmetros na linha 1);
- Valores dos tempos de viagem de cada corredor ou seção; na linha 1, esses valores possuem o padrão MOEsim*i*rep*k*. Nesse padrão, MOEsim significa o tempo de viagem do corredor ou seção *i* informado no arquivo “travel_time.txt”; e rep representa a replicação *k*.

Aparentemente, o AIMSUN pode gerar valores infactíveis para o tempo de viagem (com valores menores do que zero). Nesses casos, o tempo de viagem de determinado corredor viário ou seção receberá o valor 10000 como penalização, para o cromossomo ser eliminado da população no decorrer do processo de calibração.

Deve-se abrir o arquivo “geracoes.txt” e substituir todos os pontos por vírgula (caminho: Editar >> Substituir, no Bloco de Notas). Para manipular esses resultados do AG, exportá-los para o Microsoft Excel (2007 em diante) seguindo o seguinte caminho: Dados >> De Texto >> encontrar “geracoes.txt” >> Delimitado >> Tabulação e Espaço >> Concluir.

Outras coisas que devem ser feitas:

- Não se esquecer de mudar o diretório, nas linhas 11 e 12 do código; e
- Não esquecer de gerar os arquivos, tal como abaixo:

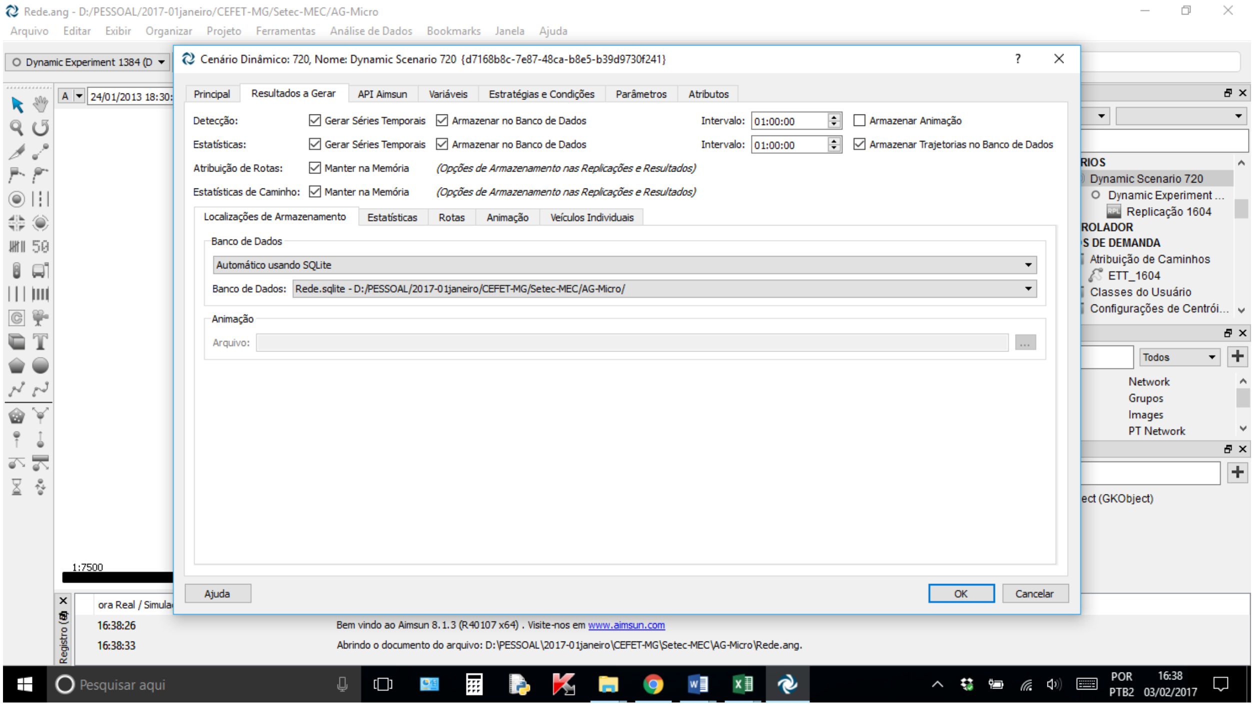
Cenário Dinâmico: 720, Nome: Dynamic Scenario 720
Principal
Resultados a Gerar
API Aimsun
Variáveis
Estratégias e Condições
Parâmetros
Atributos
Detecção:
Estatísticas:
Atribuição de Rotas:
Estatísticas de Caminho:
Gerar Séries Temporais
Armazenar no Banco de Dados
Manter na Memória
Intervalo: 01:00:00
Armazenar Animação
Armazenar Trajetorias no Banco de Dados
Localizações de Armazenamento
Banco de Dados
Automático usando SQLite
Banco de Dados: Rede.sqlite - D:/PESSOAL/2017-01janeiro/CEFET-MG/Setec-MEC/AG-Micro/
Animação
Arquivo:
Ajuda
OK
Cancelar

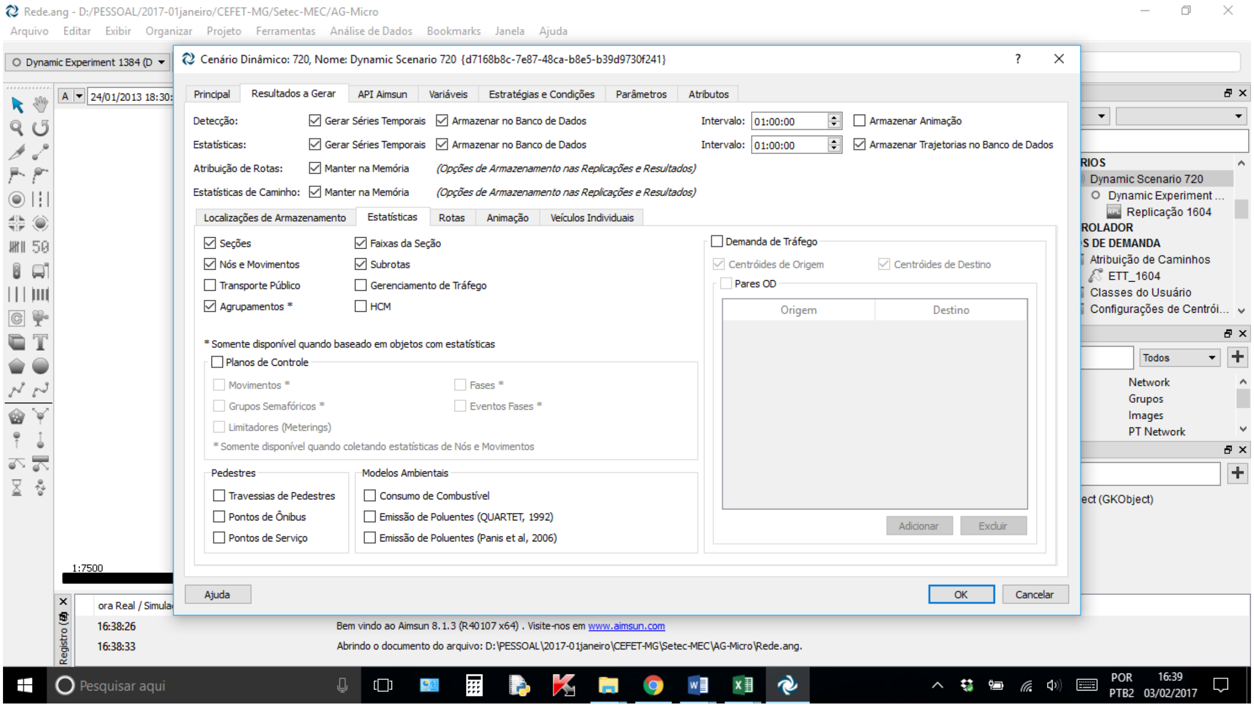
Cenário Dinâmico: 720, Nome: Dynamic Scenario 720
Estatísticas
Seções
Faixas da Seção
Nós e Movimentos
Subrotas
Transporte Público
Gerenciamento de Tráfego
Agrupamentos *
HCM
* Somente disponível quando baseado em objetos com estatísticas
Planos de Controle
Demanda de Tráfego
Centróides de Origem
Centróides de Destino
Pares OD
Origem
Destino
Adicionar
Excluir
Pedestres
Travessias de Pedestres
Pontos de Ônibus
Pontos de Serviço
Modelos Ambientais
Consumo de Combustível
Emissão de Poluentes (QUARTET, 1992)
Emissão de Poluentes (Panis et al, 2006)
Ajuda
OK
Cancelar

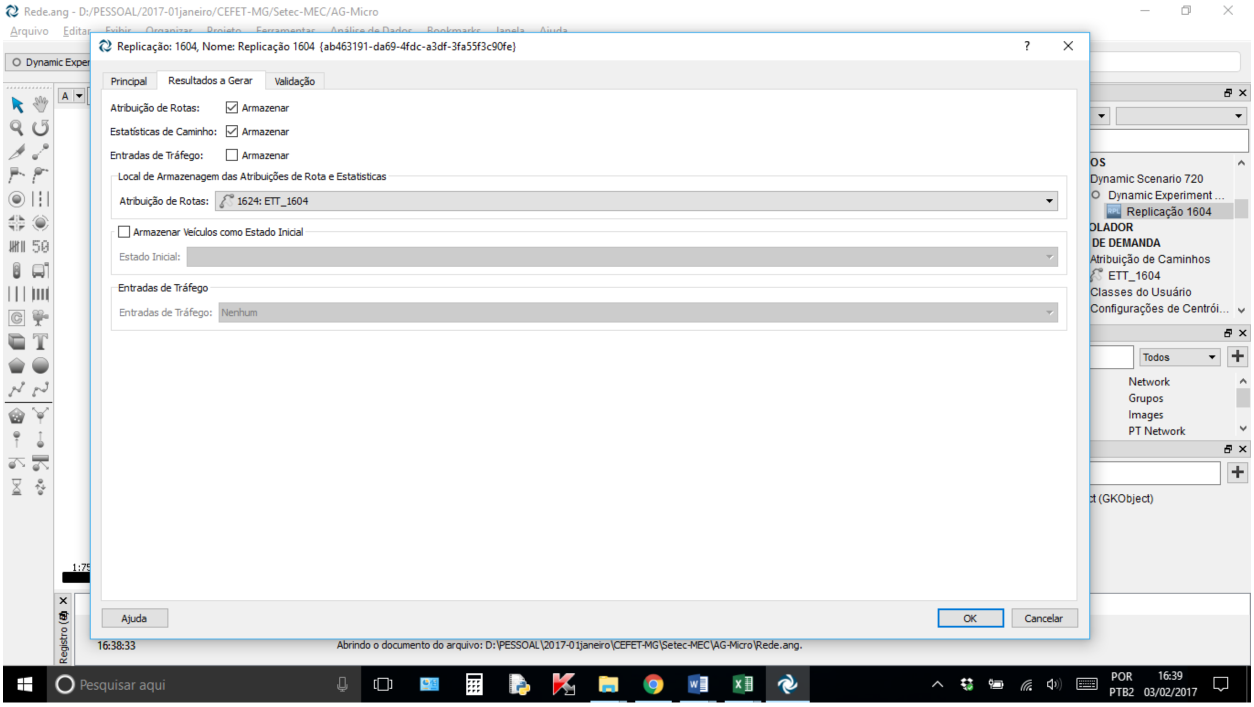

4. Contato

O AG encontra-se em fase de testes. Para reportar erros, tirar dúvidas sobre a utilização do algoritmo, e saber mais sobre como funciona os operadores do AG (mutação, predação, seleção e crossover), entrar em contato pelo e-mail: elievamjr@gmail.com ou pelo telefone (31) 97305-9297.
