# Supplementary material for: Traffic light optimization using non-dominated sorting genetic algorithm (NSGA2)
Source: Sci Rep. 2023 Sep 20;13:15550. doi: 10.1038/s41598-023-38884-2 (PMC10511403; doi:10.1038/s41598-023-38884-2)
Supplement: Supplementary file 1 — Supplementary Information. [file 41598_2023_38884_MOESM1_ESM.zip › dadosBHTrans/calibrac+î-oa+î▌Æo do modelo/Calibrac+î-oa+î▌Æo de submodelos comportamentais do simulador Aimsun para Belo Horizonte.pdf]

Calibração de submodelos comportamentais do simulador Aimsun para Belo Horizonte

Vinícius de Magalhães[1]; Warley Silva de Oliveira[2]; Bruna Castro Souza Maciel[3]; José Elievam Bessa Júnior[4]; Maria Eduarda Vieira Moura[5]; José Maurício Pinto Júnior[6]

[1] Empresa de Transportes e Trânsito de Belo Horizonte - BHTRANS, Diretoria de Sistema Viário - DSV, - Gerência de Simulação de Tráfego e Programação Semafórica - GESIT, Av Engenheiro Carlos Goulart, 900, Buritis, CEP 30455-902, Tel.: 31 3314-7832, viniciusm@pbh.gov.br; [2] BHTRANS, DSV, GESIT, warleyoliveira.eng@gmail.com; [3] BHTRANS, DSV, GESIT, brunacsmaciel@gmail.com; [4] Centro Federal de Educação Tecnológica de Minas Gerais – CEFET-MG, Departamento de Engenharia de Transportes – DET, Av. Amazonas 5.253, Nova Suíça, Belo Horizonte, MG, CEP 30.421-169, Tel.: 31 3319-7107, elievamjr@gmail.com; [5] CEFET-MG, DET, mariaeduardav.moura@hotmail.com; [6] BHTRANS, DSV, GESIT, jmjunior@pbh.gov.br.

SINÓPSE

Este trabalho teve como meta calibrar e validar parâmetros de submodelos comportamentais do simulador de tráfego Aimsun para modelagem microscópica de Belo Horizonte. A calibração e a validação a partir de um Algoritmo Genético apresentaram valores da função objetivo melhores do que usando os valores *default* do simulador.

PALAVRAS-CHAVES

Microssimulação, Calibração, Algoritmo Genético

1 INTRODUÇÃO

Simuladores de tráfego são muito usados em pesquisas e no meio técnico para modelar e analisar o sistema viário. Isso porque permite representar uma rede viária com várias características e, principalmente, pode retratar o comportamento individual dos usuários (Hollander e Liu, 2008). Com os simuladores, o usuário pode propor novos cenários alternativos, operações modificadas e analisar as atitudes que ainda poderão ser tomadas.

O simulador Aimsun é utilizado pela Empresa de Transporte e Trânsito de Belo Horizonte S/A – BHTRANS, principalmente para auxiliar na elaboração de programações semafóricas na Gerência de Simulação de Tráfego e Programação Semafórica – GESIT. Por essa razão, o objetivo geral deste trabalho foi calibrar submodelos comportamentais do simulador AIMSUN para que ele possa modelar adequadamente redes de tráfego em Belo Horizonte.

2 REVISÃO DA LITERATURA

Os modelos de simulação de tráfego são importantes ferramentas para realizar estudos acerca da operação dos sistemas viários. Seu objetivo é proporcionar uma realidade artificial e simulação do sistema real, avaliando mudanças antes de sua implantação (Cunha et al., 2009).

O *Advanced Interactive Microscopic Simulator for Urban or Non-urban Networks* (Aimsun) foi originalmente desenvolvido como programa de pesquisa na Universidade da Catalonia (UPC) e está agora na oitava versão comercial sob comando da empresa *Transport Simulation Systems* (TSS). Permite representar desde uma única faixa de ônibus até uma região inteira. O AIMSUN destaca-se pela atribuição de tráfego estático e dinâmico com simulação mesoscópica, microscópica, macroscópica e híbrida no mesmo software (TSS, 2015).

Um dos pontos mais importantes para realizar uma adequada modelagem do tráfego é a calibração de simuladores de tráfego, que é o processo pelo qual o usuário ajusta os parâmetros

do modelo, compara os resultados da simulação com dados empíricos e verifica a capacidade do modelo de simulação em representar adequadamente o fluxo de tráfego observado (Cunha, 2009). Alguns dessas medidas de desempenho são: tempo de viagem, velocidade, atrasos e tamanhos de filas. Segundo Kim e Rilett (2001), pela complexidade dos modelos de simulação de tráfego e o número de parâmetros envolvidos, o processo utilizado é geralmente heurístico, no intuito de buscar valores de parâmetros que aperfeiçoam uma ou mais medidas de ajuste. Afirma ainda que a calibração de parâmetros de um modelo de simulação pode ser considerada como um problema de otimização em que se busca o conjunto de parâmetros que maximize uma função objetivo.

Os métodos utilizados são, basicamente, de dois tipos: realizados de forma manual ou automática. Sendo o primeiro empregado há mais tempo, em que os parâmetros são ajustados pelo próprio usuário do modelo, que conduz as simulações, extrai os resultados do simulador e faz o tratamento para análise. Todo o processo é repetido até a adequação dos resultados. No segundo método todo o processo é realizado de forma automática por meio de um programa computacional, sendo o Algoritmo Genético (AG) um dos principais (Egami 2006).

O Algoritmo Genético (AG) foi inicialmente desenvolvido nas décadas de 1960 e 1970 por John Holland, mas foi publicado somente no ano de 1975 (Júnior, 2002). Os AG's são ferramentas de busca e otimização baseada nos mecanismos da seleção natural e da genética, no qual indivíduos mais aptos têm mais oportunidades de serem reproduzidos (Cunha e Setti, 2006).

A ideia do AG foi inspirada na genética e na evolução dos seres vivos. Em que a sobrevivência de indivíduos (Soluções) é proporcional ao grau de adaptação do ambiente (fitness), que é um ranking de uma função objetivo aplicada a cada indivíduo. Os indivíduos mais bem adaptados transmitem seu material genético aos seus filhos (por meio de um cruzamento, ou crossover), gerando uma nova geração. Além desse, dois outros operadores genéticos são também usados como a mutação (mudança do gene) e a predação (elimina indivíduos menos adaptados) (Cunha e Setti, 2006; Santos *et. al*., 2016).

3 COLETA DE DADOS DE TRÁFEGO

No presente estudo foi utilizada uma rede base que engloba toda a Região Metropolitana de Belo Horizonte (RMBH), construída pela BHTRANS e vem sendo atualizada ao longo dos anos por meio de várias iniciativas, como projetos de pesquisa e extensão com universidades, como o CEFET-MG.

Para realizar a microssimulação, foi escolhida a rede viária apresentada na Figura 1. A partir de um estudo anterior (Santos *et al*., 2016), foi realizada a classificação viária da rede base e foram definidos parâmetros da função de atraso de Akçelik usados na macrossimulação. Na rede viária, as avenidas do Contorno, Afonso Pena e Getúlio Vargas estão classificadas como Arterial, a av. Professor Moraes, rua Cláudio Manoel e trecho da av. Bernardo Guimarães como Coletora e o restante do sistema viário como Local.

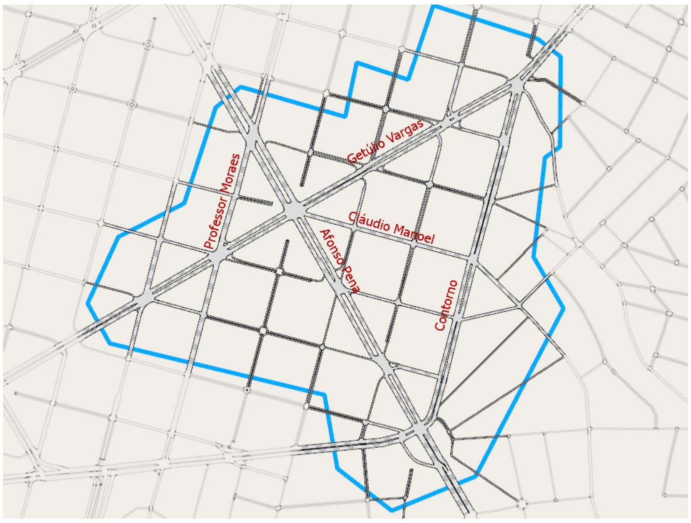

Figura 1: Mapa da área de estudo

A demanda de tráfego utilizada foi através de uma matriz OD, gerada a partir da Pesquisa OD 2012 (Batista, 2012) realizada pela Agência de Desenvolvimento da Região Metropolitana de Belo Horizonte – Agência RMBH, órgão vinculado ao Governo do Estado de Minas Gerais, o trabalho é resultado da parceria entre as Secretarias de Gestão Metropolitana – SEGEM e de Transportes e Obras Públicas – SETOP. Para geração da matriz da área de estudo foi utilizada a ferramenta do Aimsun “Static OD Traversal” que permite a criação de uma matriz de atravessamento para subredes. O cenário simulado foi o pico manhã, definido para o horário de 7 h às 8h.

A área de estudo está localizada na região centro-sul de Belo Horizonte e assim como no entorno próximo possui uma ocupação mista, residencial e comercial, fato que promove um equilíbrio nos deslocamentos de modo que as linhas de desejos criadas à partir da matriz origem e destino das viagens individuais motorizadas mostram demanda nos dois sentidos dos sistema viário principal, não se caracterizando como um sistema pendulares. O mapa com as linhas de desejos é apresentado na figura 2.

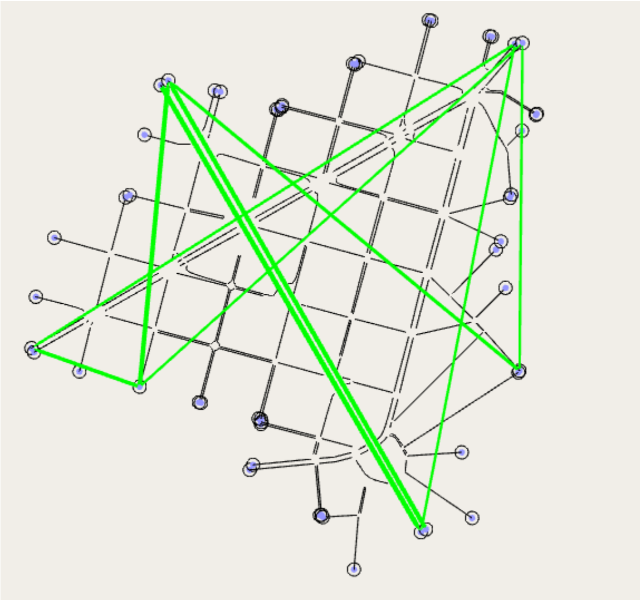

Figura 2: Linhas de desejo conforme matriz OD.

As principais linhas de transporte público gerenciada pela BHTRANS também foram lançadas na rede de simulação. Foram levantados itinerários, frequências, pontos de paradas e definido tempo médio de embarque e desembarque.

Foi realizado, ainda, um ajuste da matriz de atravessamento através da ferramenta do Aimsun "Static OD Adjustment Scenario", a partir de com dados de contagem volumétrica. Foram obtidos dados do CIT (Central Inteligente de Tráfego da BHTRANS) que conta com 31 sensores na área de estudo e, além disso, para validação foram utilizados dados de pesquisa de tráfego em três interseções. As pesquisas de tráfego possuem dados direcionais em interseções enquanto que os dados dos sensores são relativos a um determinado trecho de via. A figura 3 mostra os locais dos sensores e o ajuste obtido considerando a medida de desempenho *GEH*, que relaciona diferenças absolutas e relativas como se segue:

$$GEH = \sqrt{\frac{2(M-C)^2}{M+C}}$$

em que:
M = Fluxo Alocado;
C = Fluxo Observado.

O GEH foi menor que 5 em 86% da amostra, ou seja 26 de 30 sensores apresentou GEH inferior a 5.

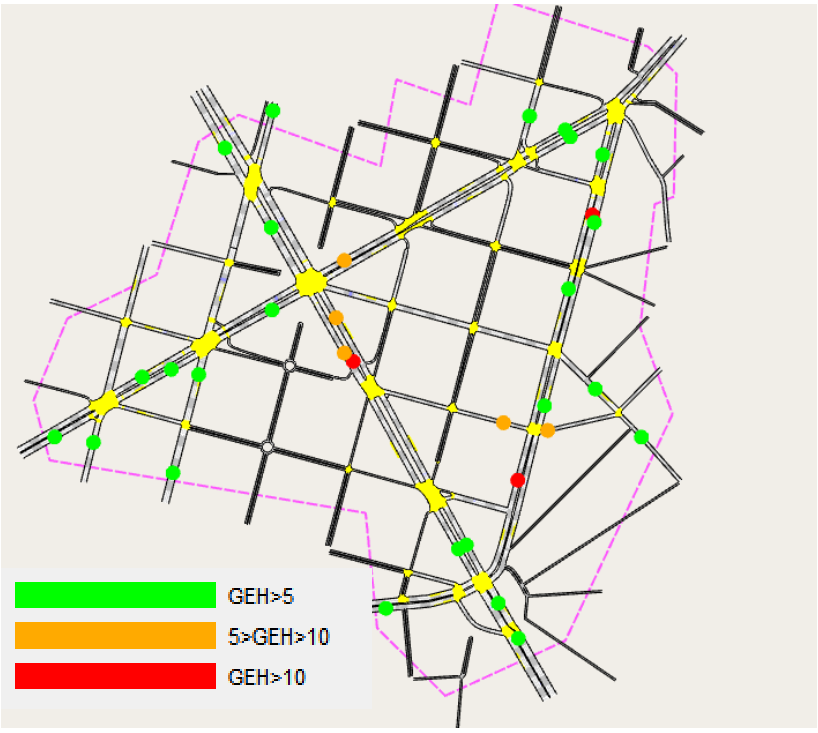

Figura 3: Resultado ajuste Matriz - GEH

O processo de calibração dos submodelos comportamentais, apresentado em seções subsequentes, foi realizado utilizando-se dados de tempo de viagem. Para garantir uma calibração adequada, foi realizada uma comparação a partir de dois conjuntos de dados. O primeiro conjunto de dados foi usado para a calibração dos parâmetros do modelo, enquanto o segundo foi utilizado para validação dos parâmetros obtidos na calibração.

Para a coleta dos tempos de viagem, foi utilizado o Método das Placas, que, conforme mencionado no Manual de Estudos de Tráfego (DNIT, 2006), consiste na anotação, na entrada e saída do trecho analisado, da placa e hora de passagem dos veículos. Posteriormente, no escritório, as chapas são “casadas” entre entradas e saídas, obtendo-se o tempo de viagem de cada veículo que entrou e saiu pelos locais observados.

Foram realizadas pesquisas em sete trechos selecionados dentro da área de estudo, procurou-se pesquisar o sistema viário principal onde acontecem a maior parte das viagens. Os trechos pesquisados estão destacados na Figura . A figura 5 mostra um gráfico de Box Plot para a coleta de calibração. Com a retirada dos *outliers*, foram obtidos os valores médios dos tempos de viagem de cada corredor viário, que foram usados para avaliar a qualidade dos conjuntos de parâmetros testados no processo de calibração e validação do Aimsun. Os tempos médios obtidos e utilizados na calibração e validação da rede de simulação são apresentados na Tabela 1.

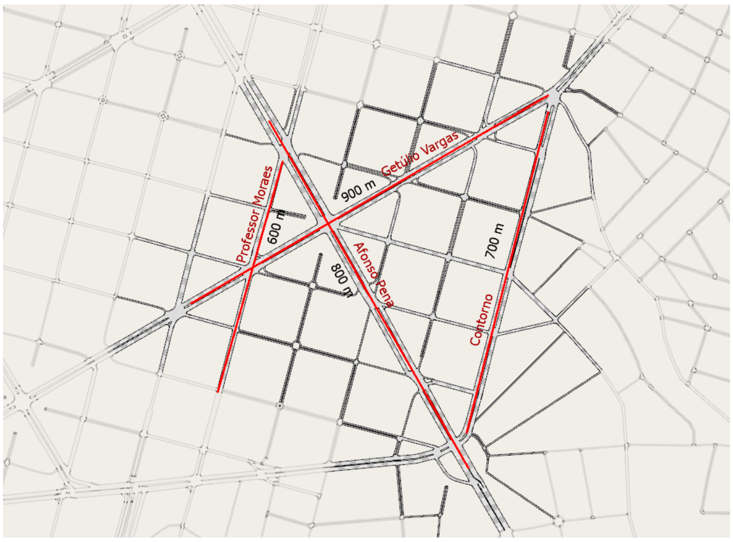

Figura 4: Trechos pesquisados

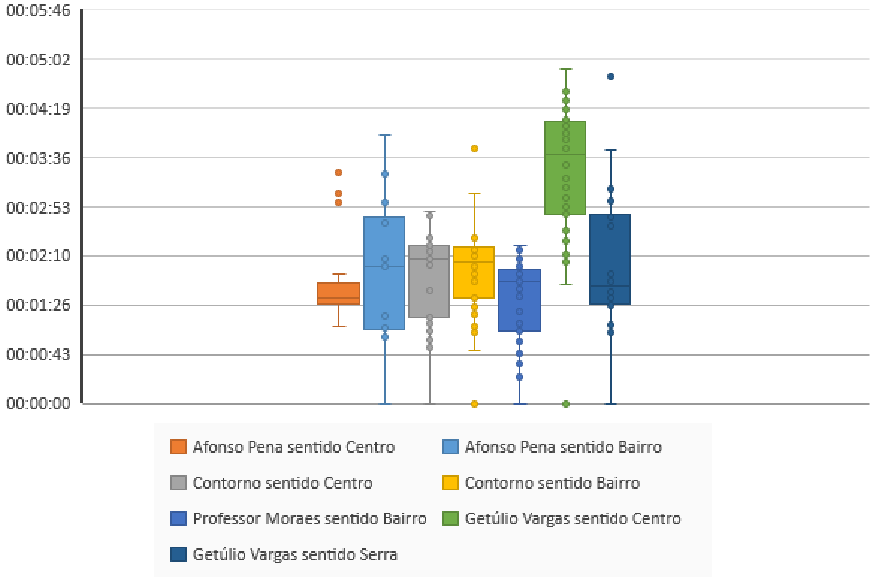

Figura 5: Tempo de viagem para o processo de calibração (pesquisa 1 - calibração)

Tabela 1: Valores de tempo médio obtido nas pesquisas

Trecho	Data P1	Tempo Viagem P1 (s)	Data P2	Tempo Viagem P2 (s)
Afonso Pena (Rodoviária / Mangabeiras)	04/04/2017	123	18/04/2017	114
Afonso Pena (Mangabeiras/Rodoviária)	04/04/2017	84	18/04/2017	103
Contorno (Savassi/Serra)	05/04/2017	122	19/04/2017	113
Contorno (Serra/Savassi)	05/04/2017	115	19/04/2017	111
Professor Moraes	05/04/2017	95	20/04/2017	78
Getúlio Vargas (Savassi/Serra)	06/04/2017	122	20/04/2017	108
Getúlio Vargas (Serra/Savassi)	06/04/2017	208	20/04/2017	149

4 ALGORITMO GENÉTICO UTILIZADO E DISCUSSÃO DOS RESULTADOS

O algoritmo genético (AG) é um processo iterativo que tem como base a teoria da evolução. Inicialmente, o AG produz uma população aleatória, gerado a partir de um espaço de busca, definido como múltiplos indivíduos derivados de parâmetros para futura calibração e soluções de problema. São feitas várias iterações e cada uma apresenta uma população inicial diferente da anterior, devido a função *fitness* avaliar a adaptabilidade do indivíduo no sistema, a qualidade da solução apresentada. Os indivíduos mais bem adaptados permanecem na população e transferem as características às próximas gerações, enquanto que os menos adaptados tendem a desaparecer. (Bessa Jr. e Setti, 2012; Goldberg, 1989).

Alguns operadores genéticos são essenciais na formação de novas populações, como *crossover*, *mutação* e *predação*. Sendo o *crossover* o operador que mescla partes importantes de outros indivíduos considerados bem adaptáveis. A *mutação* introduz a diversidade genética para produzir gerações diferentes a partir de valores aleatórios que substituem cromossomos. Por fim, a *predação* serve para eliminar os menos adaptáveis (pior solução) e inserir diversidade genética também, substituindo os indivíduos predados por novos (Bessa Jr. e Setti, 2012; Goldberg, 1989).

Tabela 2: Valores *default* dos parâmetros de calibração

Parâmetro	Categoria	Valor default	Espaço de busca	
			Mínimo	Máximo
Número de veículos	Car-following	4	1	6
Distância máxima entre veículos	Car-following	100,00	50,00	150,00
Diferença máxima de velocidade	Car-following	50,00	25,00	75,00
Tempo de reação	Tempo de reação	0,75	0,50	2,00
Tempo de reação no “Pare”	Tempo de reação	1,35	0,70	3,00
Tempo de reação no semáforo	Tempo de reação	1,35	0,70	3,00
Máxima velocidade desejada para os automóveis	Características veiculares	110,00	50,00	180,00
Máxima aceleração para os automóveis	Características veiculares	3,00	2,00	4,00
Aceitação da velocidade para os automóveis	Características veiculares	1,10	0,50	2,00
Mínima distância entre veículos para os automóveis	Características veiculares	1,00	0,50	2,00

Nesse trabalho, o AG desenvolvido buscou obter parâmetros comportamentais do Aimsun de modo que a medida de desempenho escolhida, o tempo de viagem em corredores viários, tivessem os valores simulados e observados bem próximos. O espaço de busca e os valores *default* dos parâmetros escolhidos para calibração pode ser visto na Tabela 2, realizado com base em estudos anteriores (Figueiredo *et al*., 2014; Giuffrè *et al*., 2015).

A população foi composta por 20 indivíduos e o critério de parada do algoritmo foi o número máximo de gerações igual a 20, com cinco replicações usando-se diferentes sementes de números aleatórios. Foi considerado um *crossover* com critério de seleção do tipo elitismo e taxas de diversidade (predação e mutação), respectivamente, iguais a 30% e 20%, sendo aplicadas a cada 2 gerações. A função *fitness* (objetivo) foi o erro normalizado absoluto médio entre os tempos de viagem dos corredores, por sentido, observados e simulados, conforme mostra a equação:

$$MANE = \frac{1}{N}\sum_{i=1}^{n}\frac{|x_i - y_i|}{x_i}$$

em que:

n = total de sensores;
y_i = i-ésimo tempo de viagem obtido na simulação; e
x_i = i-ésimo tempo de viagem obtido em campo.

Usando os parâmetros *default* dos parâmetros de calibração (Tabela 2), foi encontrado um valor de *MANE* de 0,32 para a rede microssimulada. A aplicação da melhor solução encontrada com o AG (Tabela 3) na microssimulação de tráfego com o AIMSUN proporcionou um valor de *MANE* de 0,20, ou 38% menor do que quando usado os valores *default* dos parâmetros.

Foi realizado um processo de validação ao testar a melhor solução encontrada na etapa de calibração com base nos tempos de viagem encontrados na segunda coleta de dados. O valor de *MANE* encontrado foi de 0,13, valor 54% menor que o encontrado a partir dos parâmetros *default*, que foi igual a 0,28.

Tabela 3: Melhor conjunto de parâmetros encontrados pelo AG

**Parâmetro**	**Valor default**
Número de veículos	1
Distância máxima entre veículos	138,88
Diferença máxima de velocidade	34,27
Tempo de reação	0,64
Tempo de reação no “Pare”	0,84
Tempo de reação no semáforo	1,25
Máxima velocidade desejada para os automóveis	74,47
Máxima aceleração para os automóveis	3,65
Aceitação da velocidade para os automóveis	0,64
Mínima distância entre veículos para os automóveis	0,56

Com relação aos resultados dos parâmetros calibrados, considera-se que os valores obtidos são mais agressivos, como é o caso dos tempos de reação e da mínima distância entre os veículos, da aceitação da velocidade e da máxima velocidade desejada (todos menores do que os valores *default*). A máxima aceleração encontrada foi maior do que o parâmetro *default*, valor que pode não estar associado ao veículo, mas sim ao motorista (devendo, portanto,

ser usado com ressalta). Os parâmetros do modelo de *car-following*, de um modo geral, também indicam uma maior agressividade do motorista brasileiro.

5 CONCLUSÕES

Este artigo teve como meta desenvolver um AG para estimar parâmetros de calibração de submodelos comportamentais para realizar simulações microscópicas de tráfego com o AIMSUN. Os resultados da aplicação do AG mostraram uma melhoria da função *fitness MANE* tanto na etapa de calibração como na etapa da validação em relação aos resultados encontrados com os parâmetros *default*.

No intuito de melhorar os resultados obtidos, seria importante obter mais dados de tráfego em outros corredores viários dentro da RMBH. Recomenda-se a aplicação do método proposto com o AG em outras áreas de Belo Horizonte para realizar a calibração dos parâmetros, assim como em outras cidades tanto do porte de Belo Horizonte como de médio porte.

Seria interessante aplicar o AG desenvolvido para outros simuladores, para meio urbano, buscando comparar os resultados da aplicação do AG para o Aimsun com outros simuladores. Ainda como recomendação, poderiam ser desenvolvidos experimentos para aplicar o AG para modelos específicos (*car-following*, por exemplo, isoladamente), ou desenvolver experimentos em campo para estimar parâmetros do Aimsun, a fim de diminuir a problemática a ser resolvida pelo AG.

6 REFERÊNCIAS

Batista, A. M. (2012) *Pesquisa Origem e Destino 2011-2012*. Governo do Estado de Minas Gerais.

Bessa Jr., J. E. e J. R. Setti (2011) *Derivation of ATS and PTSF Functions for Two-lane, Rural Highways in Brazil.* 6th International Symposium on Highway Capacity and Quality of Service, Procedia Social and Behavioral Sciences, n. 16, p. 282–292, Stockholm, Sweden.

Bethonico, F. C. (2016) *Calibração de simuladores microscópicos de tráfego através de medidas macroscópicas.* Tese (Mestrado em Ciências) – Escola de Engenharia de São Carlos, Universidade de São Paulo, São Carlos – SP.

Cunha, A. L. B. N; Bessa Jr. J. E. e Setti, J. R. (2009) *Genetic Algorithm for the Calibration of Vehicle Performance Models of Microscopic Traffic Simulators.* Progress in Artificial Intelligence 5816: 3–14.

DNIT/IPR. (2006) Manual de Estudos de Tráfego. Publicação 723 IPR – Instituto de Pesquisa Rodoviária. 384 p. Rio de Janeiro/RJ.

EGAMI, C. Y. (2006) *Adaptação do HCM-2000 para determinação do nível de serviço em rodovias de pista simples sem faixas adicionais no Brasil.* 2006. Tese (Doutorado em Transportes) - Escola de Engenharia de São Carlos, Universidade de São Paulo, São Carlos.

Figueiredo, M.; Seco, A.; Silva, A. B. (2014) *Calibration of microsimulation models – The effect of calibration parameters errors in the models' performance.* 17th Meeting of the EURO Working Group on Transportation, n. 3, p. 962–971, Sevilla, Spain.

Giuffrè, O. *et al*. (2015) *Developing passenger car equivalents for freeways by microsimulation.* 18th Euro Working Group on Transportation, n. 10, p. 93–102, Delft, The Netherlands.

Goldberg, D. E. (1989) *Genetic algorithms in search, optimization and machine learning*, Addison-Wesley, Reading, Massachussets.

Hollander, Y. e R. Liu (2008) The principles of calibrating traffic microsimulation models. Transportation, Springer Science and Business Media, LLC, v. 35, p. 347-362. Published online: 15 January 2008.

Kim, Kyu-Ok, and L. R. Rilett (2001) *Genetic-algorithm based approach for calibrating microscopic simulation models.* Intelligent Transportation Systems, 2001. Proceedings. 2001 IEEE. IEEE.

Portugal, L. S. (2005) *Simulação de Tráfego: Conceitos e Técnicas de Modelagem.* 197 p. ed. Interciência. ISBN: 85-719-3124-0, 97-885-719-312-44.

Santos, G. H. G.; J. E. Bessa Jr.; H. F. Pimenta; V. Magalhães (2016) *Calibração e validação de funções de atraso usadas no simulador Aimsun por meio de um algoritmo genético.* XXX ANPET Congresso de Pesquisa e Ensino de Transportes, Anais..., Rio de Janeiro – RJ.

Soares Filho, S. (1974) *Um Simulador de Tráfego Urbano.* Tese (Mestrado em Ciências) – Faculdade de Engenharia de Campinas, Universidade Estadual de Campinas, Campinas, SP.

TSS (2015) *AIMSUN User's Manual – Version 8.1*. Transport Simulation System – TSS.

AGRADECIMENTOS

Os autores agradecem o apoio do Setec-MEC/CNPq pelo suporte financeiro sob a forma de auxílio a Projeto de Extensão. Agradecem, ainda, à FAPEMIG e ao CEFET-MG pelo auxílio para participação no congresso. Os autores também agradecem a GESIT/BHTrans pela parceria no Projeto de Extensão que originou este trabalho.
