# Supplementary material for: Traffic light optimization using non-dominated sorting genetic algorithm (NSGA2)
Source: Sci Rep. 2023 Sep 20;13:15550. doi: 10.1038/s41598-023-38884-2 (PMC10511403; doi:10.1038/s41598-023-38884-2)
Supplement: Supplementary file 1 — Supplementary Information. [file 41598_2023_38884_MOESM1_ESM.zip › dadosBHTrans/calibrac+î-oa+î▌Æo do modelo/CALIBRAC+î-oA+î▌ÆO E VALIDAC+î-oA+î▌ÆO DO SIMULADOR TRANSMODELER PARA MODELAGEM DE CORREDORES VIA+î-üRIOS EM CIDADES DE GRANDE PORT.pdf]

CALIBRAÇÃO E VALIDAÇÃO DO SIMULADOR TRANSMODELER PARA MODELAGEM DE CORREDORES VIÁRIOS EM CIDADES DE GRANDE PORTE

José Elievam Bessa Júnior
Centro Federal de Educação Tecnológica de Minas Gerais
Departamento de Engenharia de Transportes
André Luis Medeiros
Alessandro Macêdo de Araújo
Janailson Queiroz Sousa
Transitar Engenharia Ltda.
Vinícius de Magalhães
Empresa de Transportes e Trânsito de Belo Horizonte
Gerência de Estudos de Circulação e Projetos
Warley Silva de Oliveira
Centro Federal de Educação Tecnológica de Minas Gerais
Departamento de Engenharia de Transportes

RESUMO
Este artigo teve como meta comparar os resultados da calibração e validação de parâmetros de submodelos comportamentais do TransModeler para duas cidades brasileiras de grande porte. Foram obtidos dados de tempos de viagem em um corredor viário de cada cidade, a fim de aplicar um Algoritmo Genético (AG) para calibrar o modelo de simulação de tráfego. O AG foi aplicado duas vezes, para os dados coletados em cada cidade. A validação foi realizada de duas formas. Primeiro, foram testados os parâmetros calibrados para a mesma rede viária, mas para dados de tráfego obtidos em outro período. Depois, os parâmetros foram usados na rede viária de outra cidade. No primeiro processo de validação, os tempos de viagem simulados estiveram próximos dos valores obtidos em campo. No entanto, no segundo processo de validação, ocorreu o inverso. Isso pode indicar que os motoristas possuem comportamentos diferentes entre as duas cidades.

ABSTRACT
This paper aimed to compare the results of a calibration and validation of TransModeler's behavioral submodels parameters for two Brazilian large cities. Travel time data were collected in an urban corridor of each city in order to apply a Genetic Algorithm (GA) to calibrate the traffic simulation model. The GA was applied twice, for the traffic data collected in each city. The validation step was made in two ways. First, the calibrated parameters were tested for the same traffic network using traffic data obtained in another time. After that, the parameters were used on the traffic network from the other city. In the first validation procedure, the simulated travel time data were close to the field travel time data. However, in the second validation procedure, the opposite happened. This might indicate that the driver behavior between the cities are different.

1. INTRODUÇÃO
Os modelos de simulação de tráfego têm sido bastante utilizados por pesquisadores e técnicos para realizar análises de desempenho de sistemas de transportes. Para realizar essa modelagem, os microssimuladores de tráfego buscam representar vários aspectos no sistema, como o desempenho veicular e o comportamento dos motoristas. No que diz respeito ao segundo aspecto, os simuladores se valem de submodelos como o de escolha de rota, o de mudança de faixas e o de *car-following* (Toledo *et al.*, 2005; Zhang *et al.*, 2006; Hollander e Liu, 2008). Muitos desses submodelos incluem um grande número de parâmetros que precisam ser calibrados para as condições locais onde serão aplicados (FHWA, 2004; Hollander e Liu, 2008). Isso porque os parâmetros que são fornecidos pelo simulador (*default*) representam as condições de onde os simuladores foram desenvolvidos.

O Algoritmo Genético (AG) é um método de busca e otimização baseado na teoria da evolução que usa uma população de soluções, ao contrário de outras técnicas que partem de uma única solução e buscam melhorá-la. As aplicações de AG's aumentam as chances de alcançar um ótimo global, diminuindo, portanto, as chances da melhor solução encontrada ser um ótimo local (Tomassini, 1995; Hellinga, 1998; Kim e Rilett, 2001; Ma e Abdulhai, 2002).

A técnica do AG vem sendo aplicada em várias áreas de transportes no Brasil, como em transportes público (Galvão *et al*., 2014; Prata, 2016), em Logística (Isler *et al*., 2012; Martins e Silva, 2017), em problemas de roteirização (Novaes *et al*., 2011) e no transporte aéreo (Gomes e Gualda, 2011). O AG é também um dos métodos que mais tem sido utilizados para calibrar simuladores de tráfego, um problema considerado complexo. As aplicações já são bastante disseminadas, com estudos sendo realizados no ajuste de modelos de desempenho de caminhões em rodovias (Bessa Jr. *et al*., 2008; Cunha *et al*., 2009), para microssimulação de rodovias de pista simples (Mon-Ma, 2008; Bessa Jr. e Setti, 2015; Bessa Jr. *et al*., 2017), para meio urbano (Medeiros *et al*., 2013; Magalhães *et al*., 2017) e até em macrossimulações (Santos *et al*., 2016).

No entanto, no que diz respeito às aplicações de AG para calibrar e validar simuladores de tráfego, os ajustes têm sido realizados apenas usando-se dados obtidos num mesmo local. Dessa forma, são coletados dados para calibração na mesma rede viária em que são coletados dados para a validação, mas em outro período. Caso essa validação fosse realizada para outra rede viária, seria possível afirmar que os motoristas desses dois locais possuem comportamentos semelhantes, evitando refazer o ajuste dos parâmetros do simulador e, por consequência, economizando recursos.

Dessa forma, foi traçado o objetivo geral deste trabalho, que foi calibrar e validar um microssimulador de tráfego, o TransModeler (Caliper, 2017), para corredores viários localizados em duas grandes cidades brasileiras, Belo Horizonte e Fortaleza. A seção seguinte apresenta detalhes sobre a coleta de dados e a modelagem dos corredores viários selecionados.

2. COLETA DE DADOS E MODELAGEM DOS CORREDORES VIÁRIOS

Neste trabalho, foram coletados dados de tráfego em dois corredores viários, um em Belo Horizonte e outro em Fortaleza, cidades consideradas metrópoles brasileiras e de porte semelhantes. Na Figura 1, é apresentada a Av. do Contorno, em Belo Horizonte, modelada no TransModeler. Nela, é possível observar as vias transversais, incluindo duas importantes avenidas do município, a Av. Getúlio Vargas e a Av. Afonso Pena. Na Figura 2, é apresentada a Av. Pontes Vieira, em Fortaleza, também inserida no TransModeler com vias importantes para a cidade, como a Av. Barão de Studart.

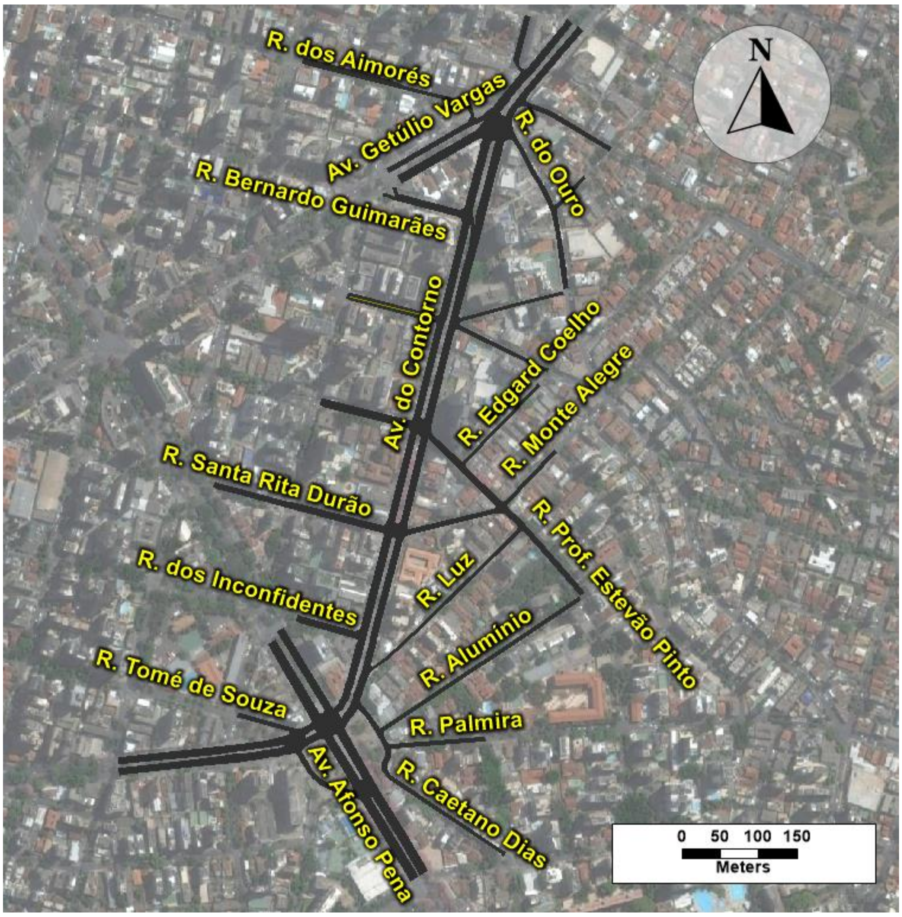

Figura 1: Av. do Contorno modelada no TransModeler

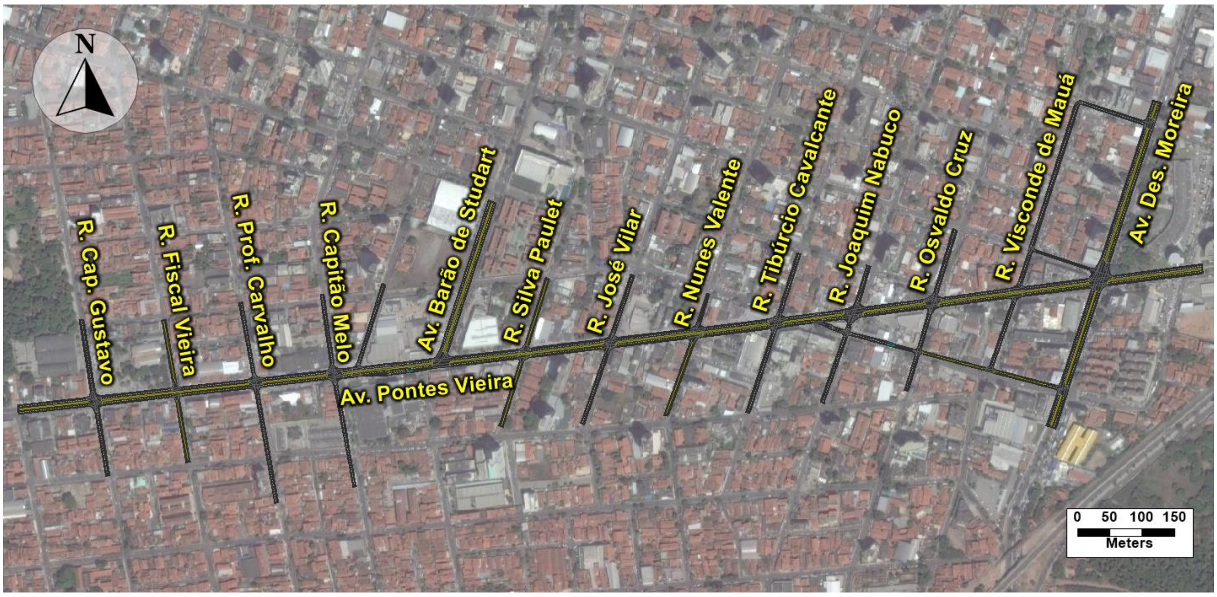

Figura 2: Av. Pontes Vieira modelada no TransModeler

Nos dois corredores viários, foram obtidas amostras de tempos de viagem dos veículos, em ambos os sentidos e em dois dias distintos. No caso de Belo Horizonte, as informações foram coletadas entre as avenidas Afonso Pena e Getúlio Vargas nos dias 5 e 19 de abril de 2017, durante o pico da manhã, entre 7:30 h e 8:30 h. Na pesquisa realizada em Fortaleza, os dados

de tráfego foram coletados no dia 19 de outubro de 2016, entre 7:00 h e 8:00 h, e no dia 26 de outubro de 2016, das 8:00 h às 9:00 h. Na Tabela 1, observa-se os valores médios dos tempos de viagem de cada corredor viário. Os corredores viários selecionados estão localizados próximos às regiões centrais de suas respectivas cidades e possuem um entorno similar, com uma ocupação que promove uma alta demanda por viagens.

Tabela 1: Valores de tempo de viagem médios obtidos nas pesquisas

Corredor viário	Sentido	Dados da calibração		Dados da validação	
		Data da coleta	Tempo de viagem (s)	Data da coleta	Tempo de viagem (s)
Av. do Contorno	Sul-Norte	05/04/2017	122	19/04/2017	132
Av. do Contorno	Norte-Sul	05/04/2017	113	19/04/2017	129
Av. Pontes Vieira	Oeste-Leste	19/10/2016	266	26/10/2016	292
Av. Pontes Vieira	Leste-Oeste	19/10/2016	260	26/10/2016	277

Para a coleta dos tempos de viagem, foi utilizado o Método das Placas, que, conforme mencionado no Manual de Estudos de Tráfego (DNIT, 2006), consiste na anotação, na entrada e saída do trecho analisado, da placa e da hora de passagem dos veículos. Posteriormente, as placas registradas nas entradas e saídas dos segmentos são comparadas, obtendo-se o tempo de viagem de cada veículo, por sentido.

O processo de calibração dos submodelos comportamentais, apresentado em seções subsequentes, foi realizado utilizando-se os dados de tempo de viagem médios apresentados. Apesar da utilização dessa medida de desempenho ser influenciada por diferentes submodelos comportamentais ao mesmo tempo, ela foi escolhida por ser uma das mais simples de ser obtidas em campo. O primeiro conjunto de dados foi usado para a calibração dos parâmetros do modelo, enquanto que o segundo foi utilizado para validação dos parâmetros obtidos na calibração de cada modelo de simulação de tráfego.

3. MÉTODO DE CALIBRAÇÃO PROPOSTO

O AG é um processo iterativo que tem como base a teoria da evolução. Inicialmente, o AG produz uma população de soluções aleatória, gerada a partir de um espaço de busca. A população inicial evolui surgindo outras gerações de indivíduos que tem, como base, a função *fitness* (objetivo), que representa o grau de qualidade da solução testada. Os indivíduos (cromossomos) mais bem adaptados permanecem na população e transferem suas características às próximas gerações, enquanto que os menos adaptados tendem a desaparecer (Goldberg, 1989).

Os operadores genéticos são essenciais na formação de novas populações, como *crossover*, *mutação* e *predação*. No *crossover*, são mescladas partes importantes de outros indivíduos considerados bem adaptados para formar os indivíduos da geração seguinte. A *mutação* introduz diversidade genética ao substituir partes dos cromossomos selecionados aleatoriamente. Por fim, a *predação* serve para eliminar os menos adaptáveis (piores soluções) e também insere diversidade genética, substituindo os indivíduos predados por novos com base no espaço de busca estabelecido pelo usuário (Goldberg, 1989; Bessa Jr *et al.*, 2017).

Neste trabalho, o AG desenvolvido buscou obter valores dos parâmetros comportamentais do TransModeler de modo que a medida de desempenho escolhida, o tempo médio de viagem em

corredores viários, tivessem os valores simulados e observados bem próximos. O espaço de busca e os valores *default* dos parâmetros escolhidos para calibração – que são aqueles que mais afetaram os tempos de viagem simulados – podem ser vistos na Tabela 2. Como há pouca informação sobre os valores aceitáveis dos parâmetros de calibração na literatura, os espaços de busca dos parâmetros foram estabelecidos como mais ou menos 50% em torno do valor *default*.

Tabela 2: Valores *default* dos parâmetros de calibração

Parâmetro	Categoria	Valor *default*	Espaço de busca	
			Mínimo	Máximo
Alpha (*accelerating*)	*Car-following model*	3,81	1,91	5,72
Beta (*accelerating*)		-1,67	-0,84	-2,51
Gamma (*accelerating*)		-0,89	-0,45	-1,34
Alpha (*decelerating*)		4,65	2,33	6,98
Beta (*decelerating*)		1,08	0,54	1,62
Gamma (*decelerating*)		1,65	0,83	2,48
Constant	*Lane changing - Discretionary (DLC) - Neighboring Lane Model - Lane Choice Utility Function*	-3,300	-1,6500	-4,9500
Path Influence Factor		-0,200	-0,1000	-0,3000
Minimum Speed Gain		0,0984	0,0500	0,1500
Average Speed Gain		0,1640	0,0800	0,2500
Slow Vehicle in Passing Lane		-0,7500	-0,3800	-1,1300
Lane Not Connected		-3,0000	-1,5000	-4,5000
Same Direction as Previous DLC		0,7500	0,3800	1,1300
Minimum (*Discretionary/Lead*)	*Gap acceptance model for lane changing - Target Lane Gap Acceptance model*	1,00	0,50	1,50
Minimum (*Discretionary/Lag*)		1,50	0,75	2,25
Minimum (*Mandatory/Lead*)		1,00	0,50	1,50
Minimum (*Mandatory/Lag*)		1,50	0,75	2,25

A população foi composta por 20 indivíduos e o critério de parada do algoritmo foi o número máximo de gerações igual a 50. Foi considerado um *crossover* com critério de seleção do tipo elitismo e taxas de diversidade (predação e mutação), respectivamente, iguais a 30% e 20%, sendo aplicadas a cada geração. Para a etapa de calibração, foi utilizada somente uma replicação, conforme realizado em Medeiros *et al.* (2013). Na etapa de validação, foram realizadas 30 replicações, com diferentes sementes de números aleatórios. A função *fitness* (objetivo) foi o erro normalizado absoluto médio (*MANE*) entre os tempos de viagem dos corredores, por sentido, observados e simulados, conforme mostra a equação:

$$MANE = \frac{1}{N}\sum_{i=1}^{n}\frac{|y_i - x_i|}{x_i} \quad (1)$$

em que:

N = total de corredores viários, por sentido;
y_i = i-ésimo tempo de viagem médio obtido na simulação; e
x_i = i-ésimo tempo de viagem médio obtido em campo.

A função objetivo *MANE* foi escolhida por indicar um erro médio em termos absolutos, sem considerar se há desvios sistemáticos, sendo constantemente aplicados em estudos de calibração de simuladores de tráfego (Hollander e Liu, 2008).

4. RESULTADOS

Os resultados da função objetivo *MANE*, com a aplicação do AG, estão resumidos na Figura 3. A partir dos dados coletados para calibração do corredor viário da Av. do Contorno (Tabela 1), e usando-se os parâmetros de calibração *default* do TransModeler (Tabela 2), foi encontrado um valor de *MANE* de 0,276 para a rede microssimulada. A aplicação da melhor solução encontrada com o AG (Tabela 3) na microssimulação de tráfego com o TransModeler proporcionou um valor de *MANE* de 0,111, ou 60% menor do que quando usado os valores *default* dos parâmetros. Para os dados coletados na Av. Pontes Vieira, usando-se os parâmetros *default* do TransModeler, o AG encontrou um *MANE* igual 0,093. Com os parâmetros obtidos com a aplicação do AG, o valor da função *fitness* caiu para 0,052, ou 44% melhor do que quando foram usados os parâmetros fornecidos com o simulador.

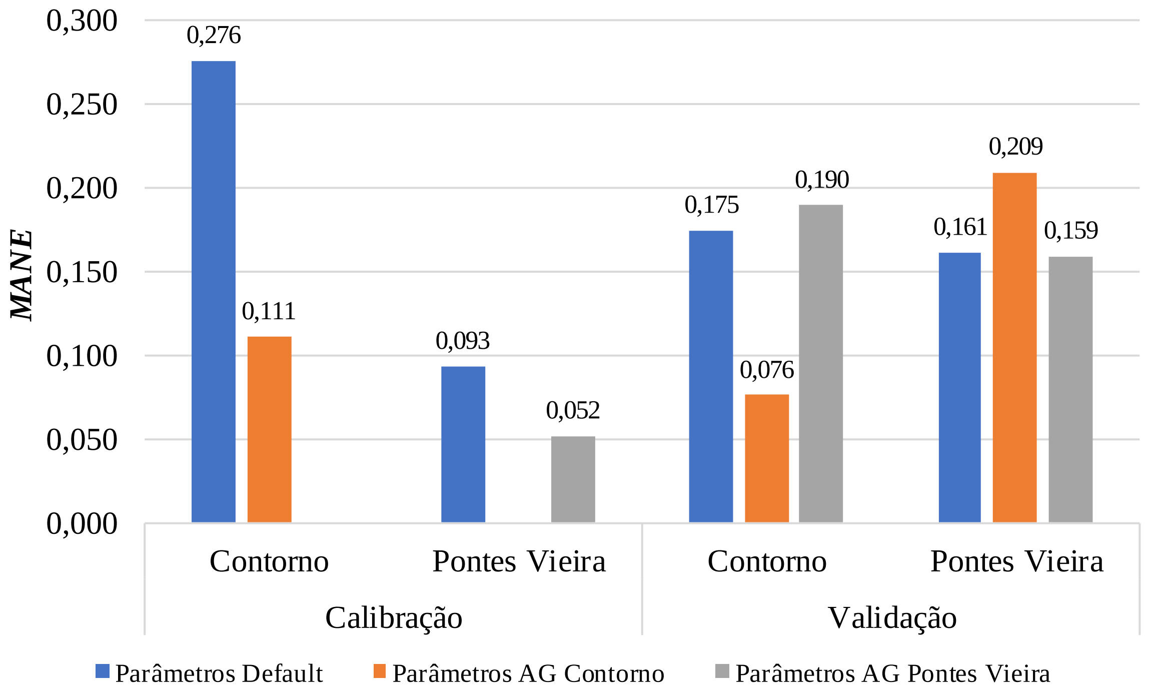

Figura 3: valores de *MANE* encontrados com a aplicação do AG e com parâmetros *default*

Os parâmetros calibrados para Fortaleza foram os que tiveram os parâmetros mais próximos dos valores *default*. Foi o caso do *Alpha*, *Beta* e *Gamma* (*accelerating*); *Constant*; *Path Influence Factor*; *Minimum Speed Gain*; e *Minimum* (*Discretionary/Lead* e *Lag*; *Mandatory/Lead*). Em alguns casos, os parâmetros encontrados para Fortaleza e Belo Horizonte seguiram a mesma tendência, ou aumentaram ou diminuíram, juntos, como ocorreu com o parâmetro *Average Speed Gain*. Situação inversa – ou seja, quando um parâmetro obtido para uma cidade foi maior do que o *default*, enquanto para outra cidade ocorreu o inverso – somente ocorreu para os seguintes parâmetros: *Alpha*, *Beta* e *Gamma* (*decelerating*) e *Lane Not Connected*. Os demais parâmetros, tanto para Fortaleza quanto para Belo Horizonte, tiveram seus valores próximos ao *default*; é o caso do *Minimum* (*Discretionary/Lead* e *Manda-*

tory/Lag). A Figura 4 ilustra melhor essas diferenças entre os parâmetros calibrados e os parâmetros *default*.

Tabela 3: Melhor conjunto de parâmetros encontrados pelo AG

Parâmetro	Valor *default*	Calibração	
		Belo Horizonte	Fortaleza
Alpha (*accelerating*)	3,81	3,03	3,89
Beta (*accelerating*)	-1,67	-1,21	-1,61
Gamma (*accelerating*)	-0,89	-1,15	-0,84
Alpha (*decelerating*)	4,65	4,79	4,50
Beta (*decelerating*)	1,08	0,65	1,22
Gamma (*decelerating*)	1,65	2,40	1,64
Constant	-3,300	-1,8884	-3,9327
Path Influence Factor	-0,200	-0,1364	-0,2084
Minimum Speed Gain	0,0984	0,1037	0,1148
Average Speed Gain	0,1640	0,1149	0,1271
Slow Vehicle in Passing Lane	-0,7500	-0,7467	-0,9228
Lane Not Connected	-3,0000	-3,3499	-2,1381
Same Direction as Previous DLC	0,7500	0,8018	0,6300
Minimum (*Discretionary/Lead*)	1,00	1,00	1,00
Minimum (*Discretionary/Lag*)	1,50	1,79	1,40
Minimum (*Mandatory/Lead*)	1,00	0,52	1,19
Minimum (*Mandatory/Lag*)	1,50	1,39	1,51

Esses resultados sugerem que a calibração realizada em Fortaleza forneceu, de maneira geral, parâmetros próximos aos valores *default*. Sabe-se que cada parâmetro tem um impacto diferente na modelagem em relação aos demais, refletindo nos dados de saída. O quão próximo a calibração de Fortaleza está do conjunto de parâmetros *default* somente podem ser mensurados ao se analisar os tempos de viagem, o que é apresentado logo a seguir. De uma maneira geral, para aqueles parâmetros que diferem significativamente dos valores *default*, há uma tendência de ter valores mais agressivos do que os parâmetros fornecidos com o simulador.

Concluída a etapa de calibração, os parâmetros obtidos da melhor solução foram testados em duas circunstâncias. Na primeira, foram aplicados para o mesmo local de calibração, mas em períodos distintos. Na segunda, foram usados os parâmetros na simulação de outra rede viária, diferente daquela usada no processo de calibração, com o intuito de avaliar se os comportamentos dos motoristas nas duas cidades estudadas são próximos.

Usando-se os parâmetros da terceira coluna da Tabela 3, encontrados para Belo Horizonte em 05/04/2017 (conforme Tabela 1), e aplicando-os para a mesma rede viária modelada com dados de um dia diferente, obtidos em 19/04/2017, foi encontrado um valor de *MANE* de 0,076. Para esse segundo conjunto de dados e com os parâmetros *default*, o valor da função objetivo ficou em 0,175, ou 57% maior do que quando foram usados os parâmetros obtidos com o AG.

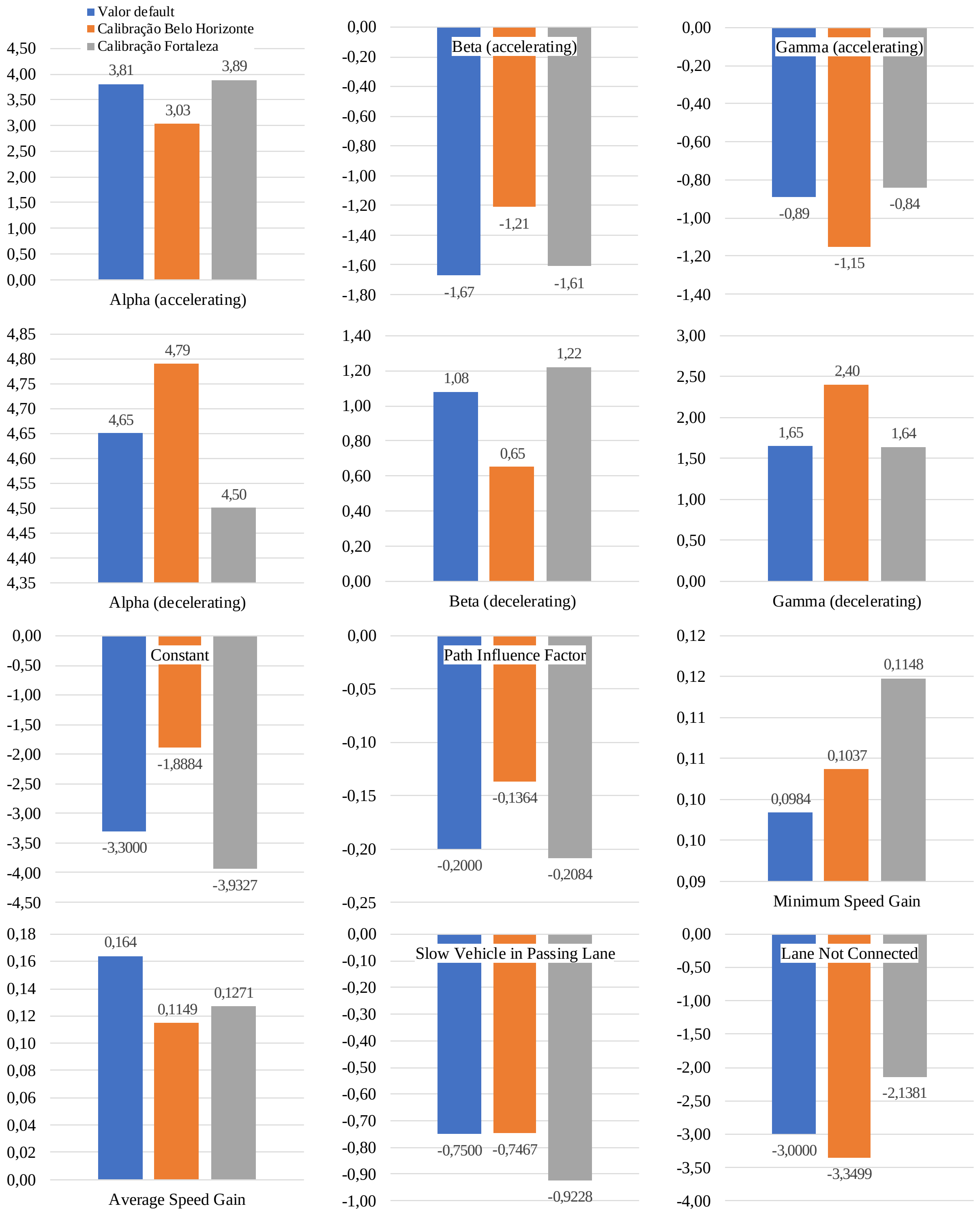

Figura 4: comparação entre os parâmetros calibrados e os parâmetros *default*

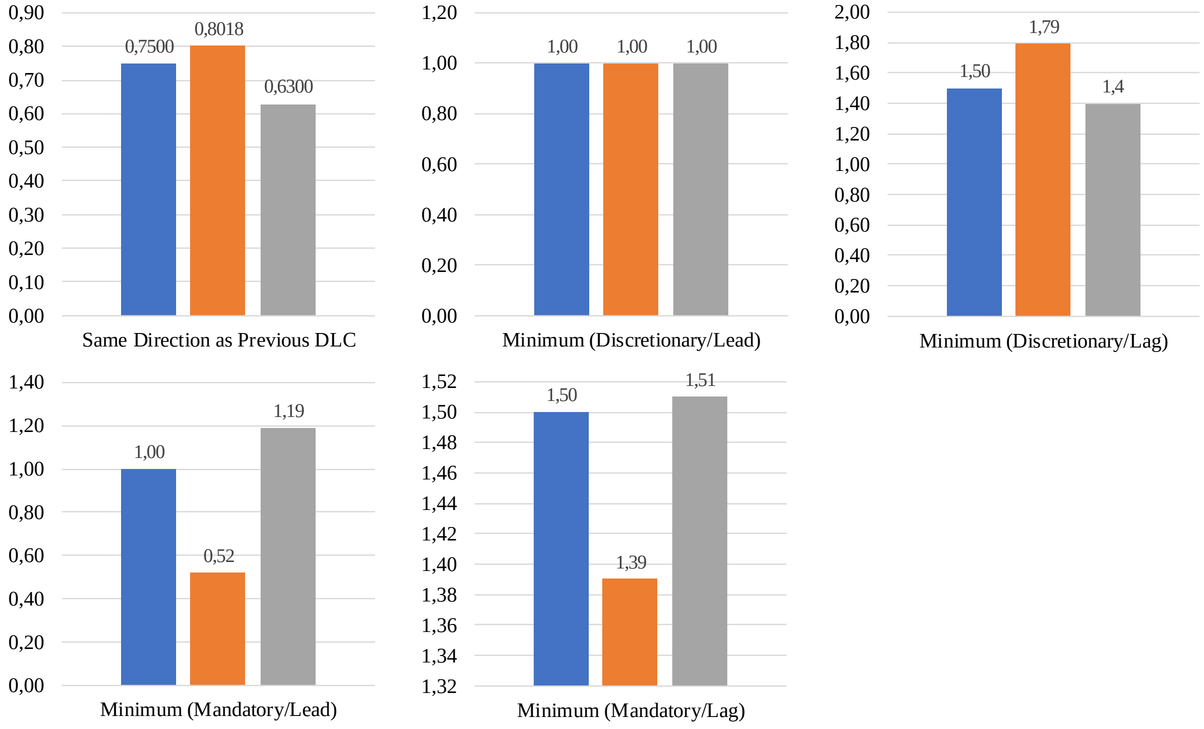

Figura 4 (cont.): comparação entre os parâmetros calibrados e os parâmetros *default*

Para o corredor da Av. Pontes Vieira, quando os parâmetros calibrados foram aplicados para outro período, foi encontrado um *MANE* de 0,159 para o segundo conjunto de dados (26/10/2016, conforme Tabela 1), quando foram usados os parâmetros obtidos com o AG no processo de calibração (com dados de 19/10/2016). Para o conjunto de dados da validação, mas usando os parâmetros *default*, o valor da função objetivo foi de 0,161, praticamente o mesmo valor encontrado com os parâmetros calibrados. Uma razão para isso pode ser o espaço de busca dos parâmetros, determinados como mais ou menos 50% em torno do valor *default*. Essa escolha pode ter sido insuficiente para determinar a diferença comportamental dos motoristas e veículos brasileiros, mas foi necessária para diminuir a chance de o AG encontrar valores irreais para alguns parâmetros. Outro motivo pode ser devido à utilização de dados referentes à hora de pico de apenas um dia para cada etapa (calibração e validação), pois a própria aleatoriedade inerente ao fenômeno pode ter causado variações no tráfego de tal forma que os parâmetros calibrados não se adequaram tão bem ao cenário de validação. O tamanho da amostra também é considerado pequeno, o que incorpora um grau de limitação elevado no ajuste dos parâmetros comportamentais.

Com os parâmetros calibrados para a Av. do Contorno aplicados para o segundo conjunto de dados da rede viária da Av. Pontes Vieira, o valor de *MANE* encontrado foi igual a 0,209. Como dito antes, usando-se parâmetros encontrados na fase de calibração da Av. Pontes Vieira, a função objetivo foi em torno de 0,159, ou 30% melhor do que usando os parâmetros obtidos a partir dos dados de Belo Horizonte.

De maneira análoga, os parâmetros encontrados a partir dos dados da Av. Pontes Vieira foram aplicados na rede viária da Av. do Contorno (segundo conjunto de dados, de 19/04/2017). O resultado foi um *MANE* de 0,190. A partir dos parâmetros *default* do simulador, o *MANE* en-

contrado foi igual a 0,175, cerca de 9% melhor do que se usando os parâmetros encontrados para a Av. Pontes Vieira.

O fato dos resultados de *MANE* com a calibração para a Av. Pontes Vieira estarem próximos do que quando se usam os parâmetros de calibração *default* é explicado pelos parâmetros estarem próximos, conforme visto na Tabela 3. As diferenças encontradas entre esses parâmetros parecem não ter sido grandes à ponto de fornecer resultados simulados dos tempos de viagem próximos aos observados em campo. Situação inversa ocorreu para o conjunto de parâmetros calibrados para Belo Horizonte, que apresentou bons resultados de validação quando aplicados para dados coletados na cidade, mas o mesmo não ocorreu com os parâmetros calibrados para o tráfego de Fortaleza.

5. CONSIDERAÇÕES FINAIS

Este trabalho teve como meta comparar os resultados da calibração e validação de parâmetros do microssimulador TransModeler para duas cidades de grande porte brasileiras, Belo Horizonte e Fortaleza. Foram simulados dois corredores viários, um de cada cidade: a Av. do Contorno, na região centro-sul de Belo Horizonte; e a Av. Pontes Vieira, localizada próxima a região central de Fortaleza. Para avaliar a eficiência das soluções testadas, realizadas por meio de um AG, foram obtidos tempos de viagem nos corredores viários, por sentido.

Diferentemente do que se observa, geralmente, em outros trabalhos acerca do ajuste de parâmetros de submodelos de simuladores de tráfego, a validação foi realizada tanto para a mesma rede viária, mas para dados de tráfego obtidos em outro período (mais comum), quanto para outro corredor viário (em outra cidade de porte similar). Os resultados obtidos mostraram que a calibração foi eficiente. No primeiro processo de validação, pelo menos para a calibração para a Av. do Contorno, os valores do tempo de viagem simulados estiveram mais próximos dos valores obtidos em campo, em comparação com os resultados quando foram usados os parâmetros *default*. Com a outra validação, aplicando-se para uma cidade os parâmetros obtidos da calibração no outro município, os tempos de viagem obtidos não estiveram próximos dos dados de campo, o que pode indicar que os motoristas possuem comportamentos diferentes entre as cidades.

Para trabalhos futuros, recomenda-se coleta de alguns parâmetros dos submodelos comportamentais do TransModeler em campo, fazendo com que diminuam o número de parâmetros de calibração, ocasionando num problema de otimização mais simples para o AG resolver. Outra melhoria seria a escolha de uma função *fitness* que não fosse baseada apenas no tempo de viagem, que é influenciada por mais de um submodelo comportamental ao mesmo tempo. Uma maneira de fazer isso seria a escolha de experimentos de calibração que possam isolar os submodelos comportamentais e, caso possível, os próprios parâmetros dos submodelos. O aumento do conjunto de dados poderia, ainda, ajudar na obtenção de melhores resultados de calibração e de validação.

Seria interessante, também, avaliar o espaço de busca dos parâmetros escolhidos para calibração. A escolha de um espaço de busca em torno dos valores *default* pode ter inibido uma maior melhora da calibração realizada para a Av. do Contorno, assim como pode ter contribuído para que o erro de validação da Av. Pontes Vieira fornecesse um *MANE* semelhante quando foram usados os parâmetros *default*. A alteração do espaço de busca, que pode ser feita a partir de uma revisão bibliográfica mais minuciosa, também pode influenciar bastante nos resul-

tados da validação para uma cidade diferente da que foi usada na calibração. O aumento do espaço de busca tem que ser realizado com cuidado, uma vez que o AG pode buscar valores irreais dos parâmetros na tentativa de aproximar as medidas de desempenho simuladas e calibradas que compõem a função objetivo (*MANE*).

Recomenda-se, ainda, aplicar esse método de calibração para outros corredores de Belo Horizonte e Fortaleza. Tanto os parâmetros calibrados com o AG como o próprio método de ajuste dos parâmetros podem ser testados para cidades de grande porte, objeto deste artigo, ou mesmo para municípios de pequeno e médio portes.

Agradecimentos
Os autores agradecem ao CEFET-MG pelo auxílio para participação no congresso.

REFERÊNCIAS

Bessa Jr., J. E.; F. A. A Lima; A. L. B. N. Cunha e J. R. Setti (2008) Calibração do modelo de desempenho do simulador Integration através de um algoritmo genético. XXII ANPET Congresso de Pesquisa e Ensino em Transportes, *Anais*..., Fortaleza-CE, CD-ROM.

Bessa Jr., J. E. e J. R. Setti (2015) Avaliação de medidas de desempenho para rodovias de pista simples obtidas a partir de relações fluxo-velocidade. XXIX Congresso Pesquisa e Ensino em Transportes, *Anais*..., Ouro Preto - MG.

Bessa Jr., J. E.; J. R. Setti e S. S. Washburn (2017) Evaluation of Models to Estimate Percent Time Spent Following on Two-Lane Highways. *Journal of Transportation Engineering, Part A: Systems*, v. 143, p. 04017010-1-04017010-9.

Caliper (2017) *TransModeler Traffic Simulation Software - TransModeler Overview*. Disponível em: < http://www.caliper.com/transmodeler/default.htm>. Acesso em: 11/07/2017.

Cunha, A. L. B. N.; J. E. Bessa Júnior e J. R. Setti (2009) Genetic Algorithm for the Calibration of Vehicle Performance Models of Microscopic Traffic Simulators. *Fourteenth Portuguese Conference on Artificial Intelligence*. LNAI Proceedings. Heildeberg: Springer, 2009. v. 5816. p. 3-14.

DNIT (2006) *Manual de Estudos de Tráfego*. Departamento Nacional de Infra-estrutura de Transportes. Publicação IPR-723.

FHWA (2004) *Traffic Analysis Toolbox Volume III: guidelines for applying traffic microsimulation modeling software*. Federal Highway Administration (FHWA), jul 2004.

Galvão, M. L.; M. V. Lamar e P. W. G. Taco (2014) Desenho automático de mapas octalineares de rede de transporte público utilizando algoritmo genético. *Transportes*, v. 22, n. 1, p. 21-30.

Goldberg, D. E. (1989) *Genetic algorithms in search, optimization and machine learning*, Addison-Wesley, Reading, Massachussets.

Gomes, W. P.; N. D. F. Gualda (2011) Modelagem integrada do problema de programação de tripulantes de aeronaves. *Transportes*, v. 19, n. 1, p. 23-32.

Hellinga, B. (1998) Requirements for the validation and calibration of traffic simulation models. *Proceedings of Canadian Society for civil engineering*, p. 211-222.

Hollander, Y. e R. Liu (2008) The principles of calibrating traffic microsimulation models. *Transportation*, Springer Science and Business Media, LLC, v. 35, p. 347-362. Published online: 15 January 2008.

Isler, C. A.; A. C. Bonassa e C. B. Cunha (2012) Algoritmo genético para resolução do problema de p-medianas capacitado associado à distribuição de peças automotivas. *Transportes*, v. 20, n. 2, p. 5-14.

Kim, K. e R. L. Rilett (2001) Genetic algorithm based approach for calibration microscopic simulation models. *IEEE Intelligent Transportation Systems Conference Proceedings*, Oakland, CA, USA, p. 698–704.

Ma, T. e B. Abdulhai (2002) Genetic Algorithm-based Optimization Approach and generic tool for calibration traffic microscopic simulation parameters. *Transportation Research Record 1800*, TRB, National Research Council, Washington, D.C., p. 6-15.

Magalhães, V.; W. S. Oliveira; B. C. S. Maciel; J. E. Bessa Jr.; M. E. V. Moura, e J. M. Pinto Jr. (2017) Calibração de submodelos comportamentais do simulador Aimsun para Belo Horizonte. ANTP, 21º Congresso Brasileiro de Transporte e Trânsito, São Paulo - SP.

Martins, L. C. e G. P. Silva (2017) Aplicação de um algoritmo genético ao problema de rodízio de tripulações do sistema de transporte público urbano. *Transportes*, v. 25, n. 1, p. 31-40.

Medeiros, A. L.; M. M. Castro Neto; C. F. G. Loureiro e J. E. Bessa Jr. (2013) Calibração de redes viárias urbanas microssimuladas com o uso de algoritmos genéticos. XXVII ANPET Congresso de Pesquisa e Ensino em Transportes, *Anais...*, Belém-PA, 2013.

Mon-Ma, M. L. (2008) *Adaptação do HCM-2000 para rodovias de pista simples com faixas adicionais típicas do Estado de São Paulo*. 162p. Tese (Doutorado) EESC-USP, São Carlos.

Novaes, A. G.; P. J. Burin; E. T. Bez e B. Scholz-Reiter (2011) Roteirização dinâmica de veículos usando simulação e algoritmo genético. *Transportes*, v. 24, n. 1, p. 19-30.

Prata, B. A. (2016) Um algoritmo genético multiobjetivo para a programação integrada de veículos e tripulações. *Transportes*, v. 19, n. 3, p. 85-92.

Santos, G. H. G.; J. E Bessa Jr.; H. F. Pimenta e V. Magalhães (2016) Calibração e Validação de Funções de Atraso Usadas no Simulador Aimsun por Meio de um Algoritmo Genético. XXX ANPET Congresso de Pesquisa e Ensino em Transportes, *Anais...*, Rio de Janeiro - RJ.

Toledo, T.; C. F. Choudhury; M. E. Ben-Akiva (2005) Lane-changing model with explicit target lane choice. *Transportation Research Record 1934*, p. 157–165.

Tomassini, M. (1995) A survey of genetic algorithms. *Annual Reviews of Computational Physics 1995*, World Scientific, p. 87-118. Disponível em: <http://citeseerx.ist.psu.edu/viewdoc/summary?doi=10.1.1.55.5988> (Acesso em: 20 Nov 2008).

Zhang, M. H.; J. Ma; H. Dong (2006) Calibration of departure time and route choice parameters in microsimulation with macro measurements and genetic algorithm. *Proceedings of the 85th TRB Annual Meeting*, Washington, D.C.

Endereço para contato:
Prof. Dr. José Elievam Bessa Júnior (elievamjr@gmail.com)

Centro Federal de Educação Tecnológica de Minas Gerais – CEFET-MG, Departamento de Engenharia de Transportes - DET

Av. Amazonas 5.253, Nova Suíça, Belo Horizonte, MG, 30.421-169

MSc. André Luis Medeiros (andremed_1@yahoo.com.br)
Eng. Alessandro Macêdo de Araújo (alessandro.mac.araujo@gmail.com)
MSc. Janailson Queiroz Sousa (janailsonqs@gmail.com)
Transitar Engenharia Ltda.
Av. Humberto Monte, n° 2929, sala 519s, Pici, Fortaleza, CE, 60.440-593

Eng. Vinícius de Magalhães (viniciusm@pbh.gov.br)

Empresa de Transportes e Trânsito de Belo Horizonte - BHTrans, Gerência de Estudos de Circulação e Projetos – GECIP

Av. Eng. Carlos Goulart, 900, Buritis, Belo Horizonte, MG, 30.455-902

Warley Silva de Oliveira (warleyoliveira.eng@gmail.com)

Centro Federal de Educação Tecnológica de Minas Gerais – CEFET-MG, Departamento de Engenharia de Transportes - DET

Av. Amazonas 5.253, Nova Suíça, Belo Horizonte, MG, 30.421-169
