# Supplementary material for: Traffic light optimization using non-dominated sorting genetic algorithm (NSGA2)
Source: Sci Rep. 2023 Sep 20;13:15550. doi: 10.1038/s41598-023-38884-2 (PMC10511403; doi:10.1038/s41598-023-38884-2)
Supplement: Supplementary file 1 — Supplementary Information. [file 41598_2023_38884_MOESM1_ESM.zip › dadosBHTrans/calibrac+î-oa+î▌Æo do modelo/Santos et al %5b2016%5d_v-final.docx]

**CALIBRAÇÃO E VALIDAÇÃO DE FUNÇÕES DE ATRASO USADAS NO
SIMULADOR AIMSUN POR MEIO DE UM ALGORITMO GENÉTICO**

**Gustavo Henrique Gomes Santos**

**José Elievam Bessa Júnior**

**Heron Fonseca Pimenta**

Centro Federal de Educação Tecnológica de Minas Gerais

Departamento de Engenharia de Transportes

**Vinícius de Magalhães**

Empresa de Transportes e Trânsito de Belo Horizonte

Gerência de Simulação de Tráfego e Programação Semafórica

**RESUMO**

Em redes de tráfego microscópicas, é comum a utilização de algoritmos de busca e de otimização para calibrar modelos comportamentais e de desempenho dos simuladores, como os Algoritmos Genéticos (AG’s). Na literatura, não têm sido encontradas aplicações de AG’s em simulações macroscópicas. A meta deste trabalho foi desenvolver um AG para calibrar e validar uma função de atraso (Akçelik) para modelar macroscopicamente o município de Belo Horizonte no simulador AIMSUN. Dados de tráfego também foram usados com o intuito de estimar capacidade, velocidade de fluxo livre e o parâmetro *J* da função de Akçelik para vias arteriais. Para outros tipos de vias (coletoras, locais e expressa), essas informações foram encontradas com o AG. A aplicação do algoritmo proporcionou um aumento da função *fitness* do AG (que compara os volumes simulados e observados em campo) em relação aos valores obtidos com parâmetros sugeridos na literatura.

**ABSTRACT**

In microscopic traffic networks it is common using search and optimization algorithms to calibrate behavior and performance models of simulators, like Genetic Algorithms (GA’s). In the literature GA’s applications have not been found in macroscopic simulations. The goal of this work was to develop a GA to calibrate and validate a volume delay function (Akçelik) to simulate macroscopically Belo Horizonte in AIMSUN. Traffic data were also used aiming to estimate capacity, free flow speed and the parameter *J* of Akçelik function for arterial roads. For other road types (collectors, locals and expressways) these data were found by GA. The algorithm application provided higher values of the GA’s fitness function (that compares simulated and observed traffic volumes) than the values obtained with suggested parameters in the literature.

**1. INTRODUÇÃO**

A simulação de tráfego tem sido bastante usada no meio acadêmico e por empresas públicas e privadas para solucionar problemas em redes urbanas e rurais. O surgimento dos computadores pessoais possibilitou o desenvolvimento de novos softwares de simulação capazes de gerar projetos mais complexos, acessíveis e cheios de alternativas (Hellinga, 1998).

Um simulador de tráfego consegue replicar situações complexas, enquanto métodos numéricos e analíticos não conseguem, em alguns casos, resolver os problemas adequadamente. Consegue também gerar, em um curto intervalo de tempo, diferentes cenários virtuais, demonstrando-os visualmente e sem a necessidade de testá-los na realidade, gerando economia de tempo e de recursos.

A maior parte dos simuladores de tráfego foram desenvolvidos, no princípio, para modelar redes microscópicas, aqueles em que os dados dos veículos (como velocidade e aceleração) são detalhados individualmente e atualizadas periodicamente. A modelagem macroscópica de tráfego é usualmente relacionada com a dinâmica dos fluidos, em que os veículos possuem características similares e fazem sua escolha de rota baseada numa função de custo, que pode ser relacionada, por exemplo, com a capacidade das vias. A simulação mesoscópica consiste numa modelagem com grau de complexidade intermediária entre a simulação micro a macroscópica; consiste em agrupar, em pelotões, veículos com características semelhantes (tamanho, localização, velocidade, aceleração) e podem ser explicados, por exemplo, por meio de modelos de tráfego como a relação fluxo-velocidade (Pursula, 1999; Portugal, 2005; Barceló, 2010).

De acordo com o tipo, as simulações de tráfego eram desenvolvidas separadamente. Com o passar dos anos, os softwares passaram a realizar simulações híbridas, em que mais de um tipo de simulação podem ser realizados ao mesmo tempo (TSS, 2015). Uma aplicação da simulação híbrida, por exemplo, consiste em estimar ou ajustar matrizes Origem-Destino (OD) e realizar alocações de tráfego de uma rede viária. Com isso, são atribuídos fluxos de tráfego de atravessamento de uma subrede menor, dados que são necessários para realização de simulações microscópicas.

É comum calibrar e validar os simuladores manualmente, muitas vezes usando técnicas do tipo “tentativa e erro”. Geralmente as aplicações se restringem a certos contextos específicos, não podendo ser expandidos para outros pontos da cidade sem que o processo tenha que ser totalmente reiniciado. É necessário, portanto, uma ferramenta que realize os ajustes nos parâmetros do simulador de maneira lógica e automática, tanto para redes microssimuladas, quanto para redes meso e macrossimuladas.

Para redes macroscópicas, os simuladores, a partir da determinação de zonas de tráfego, de centroides e de contagens em campo, estima a matriz OD de viagens com base em uma matriz inicial (semente). Em outra abordagem, os simuladores podem atribuir uma matriz OD sem a necessidade de ajuste, muitas vezes oriundas de pesquisas socioeconômicas feitas pelos órgãos gestores. Com a matriz OD obtida, é realizada a alocação das viagens na rede viária, que possuem seus custos estimados por funções de atraso, importante para a escolha das rotas pelos motoristas. É comum que essas funções sejam usadas de acordo com o padrão fornecido pelos simuladores ou com valores sugeridos na literatura, sem a calibração dos modelos a partir de dados obtidos em campo.

Em redes microscópicas, a calibração e a validação dos simuladores geralmente têm sido feitas para ajuste de submodelos comportamentais como os de *car-following*, de aceitação de brechas, de ultrapassagem e de modelos de desempenho. O Algoritmo Genético (AG) é uma ferramenta de busca e de otimização largamente usada para calibração de redes microscópicas, como é o caso dos simuladores Paramics (Ma e Abdulhai, 2002; Ma *et al*., 2007),
VISSIM (Park e Qi, 2005; Medeiros *et al*., 2013), TWOPAS (Mon-Ma, 2008; Bessa Jr e Setti, 2011) e Integration (Bessa Jr. *et al*., 2008).

Na literatura, não foram encontradas aplicações de algoritmos de busca e otimização, como o AG, para calibrar e validar funções de atraso. Com base nisso, a meta deste trabalho foi desenvolver um AG para calibrar e validar uma função de atraso – de Akçelik – usada para modelar macroscopicamente o município de Belo Horizonte no AIMSUN.

**2. ALOCAÇÃO DE TRÁFEGO NO AIMSUN**

O simulador de tráfego AIMSUN foi escolhido por ser amplamente utilizado por analistas da Empresa de Transportes e Trânsito de Belo Horizonte (BHTrans), órgão da prefeitura responsável pelo gerenciamento e fiscalização do sistema de transportes e de trânsito de Belo Horizonte, onde o trabalho foi desenvolvido.

O AIMSUN foi criado em 1997 em Barcelona, Espanha. O software foi o instrumento de um programa de pesquisa da Universidade de Catalunha (UPC) que se iniciou em 1989. Inicialmente, sua proposta era abordar apenas simulações microscópicas, como sugerido por sua sigla, que significa *Advanced Interactive Microscopic Simulator for Urban and Non-Urban Networks*. Desde então, foram incluídas várias funcionalidades no software, como os modelos meso e macroscópicos (TSS, 2016).

No AIMSUN, para realizar alocações de viagens de uma matriz OD estaticamente, é preciso determinar uma função de custo para os *links* da rede viária. No simulador, é possível usar um dos três tipos de funções de custo: i) *Volume Delay Function – VDF*; ii) *Turn Penalty Function - TPF*; e iii) *Junction Delay Function – JDF* (TSS, 2015). Existem, ainda, cinco diferentes tipos de métodos para realizar uma alocação de tráfego estática: i) Método “tudo ou nada”; ii) Incremental; iii) MAS; iv) Frank e Wolfe; e v) Estocástica.

Neste trabalho, foi adotado o método de alocação de tráfego Frank e Wolfe. O método baseia-se no conceito do Equilíbrio do Usuário, que assume que os viajantes tentam minimizar o tempo de viagem individual ao escolher a rota que aparenta ser a mais curta. O método é formulado a partir do princípio de Wardrop, que parte do pressuposto de que o tempo de viagem de todas as rotas utilizadas são iguais ou menores que o tempo de qualquer outra rota entre um par OD (Barceló, 2010). No caso da função de custo, escolheu-se a do tipo VDF, mas modificado em relação ao usado como padrão pelo AIMSUN, como discutido na seção a seguir.

**3. A FUNÇÃO DE ATRASO VDF**

As funções de atraso VDF são relações matemáticas usadas na etapa de alocação de tráfego do modelo quatro etapas de previsão de demanda. Essas funções representam o fator de atraso em relação à obtida em fluxo livre (Andrade *et al*.*,* 2015). A VDF representa o tempo total de viagem em um determinado trecho de via. É função da capacidade de veículos suportado por essa via e do volume de veículos transitando nela num determinado espaço de tempo, ambos dados em veículos por hora:

(1)

em que:

*t*0 = tempo de viagem em fluxo livre (h/km);

*v* = fluxo de tráfego (veic/h); e

*c* = capacidade da via (veic/h).

A relação entre esses valores na função sempre aparece pela razão *v*/*c*. As funções de atraso utilizam um fator de majoração do tempo de viagem que um indivíduo teria ao percorrer um trecho viário. O valor de *t*(*v*) é tanto maior quanto mais congestionado for o trecho. Ela cresce exponencialmente em condições congestionadas quando a demanda de tráfego é maior que a capacidade da via, quando . Esse entendimento é natural, visto que, no mundo real, demora-se mais tempo para percorrer um caminho dentro da cidade durante as horas de pico, quando há uma quantidade muito grande de veículos nas ruas.

De acordo com Huntsinger e Rouphail (2011), a indústria de softwares de simulação viária reconhece a necessidade de ajustes locais e de melhorias nas funções de atraso, visto que a maioria desses softwares apresenta ferramentas de calibração ajustáveis pelo usuário, como ocorre também com o AIMSUN.

Segundo Spiess (1989), diferentes tipos de funções de atraso foram utilizados no passado. A mais antiga e utilizada delas é a função de atraso do *Bureau of Public Roads –* BPR (BPR, 1964). Ela foi proposta na segunda edição do *Highway Capacity Manual* (HCM), de 1965, e possui formato parabólico:

(2)

sendo *α* e *β* constantes de calibração cujos valores são baseados nas características da via. Machado e Ribeiro (2003) utilizaram a função BPR para calibrar o trecho da ponte Rio-Niterói a partir de dados empíricos. Os valores das constantes que encontraram para *α* e *β* foram de 0,21 e 3,82, respectivamente.

Spiess (1989) faz algumas ressalvas em relação ao uso da função BPR. Ele afirma que seus valores tendem ao infinito segundo uma assíntota vertical para valores de supersaturação, quando . Além disso, a função ainda pode apresentar sensibilidade praticamente nula em baixos níveis de tráfego.

Andrade *et al*. (2015) ainda citam outras três funções de atraso – Cônica, de Akçelik e Logística – como as principais encontradas na literatura e incorporadas à maioria dos modelos de demanda existentes no mercado.

Dowling and Skabardonis (2006) afirmam que a função de atraso de Akçelik (Akçelik, 1991) tende a se aproximar mais dos resultados previstos analiticamente através da teoria de filas, especialmente nas situações de supersaturação das vias. A função de atraso de Akçelik tem a seguinte formulação:

(3)

em que:

*t*Akç = tempo total de viagem (h/km);

*T* = duração do intervalo de análise (h); e

*J* = coeficiente de calibração.

Segundo Akçelik (1991), o parâmetro *J* é dado pelo produto de dois outros parâmetros: *k* – que depende da aleatoriedade do processo de chegadas e de atendimento numa via, onde formam-se filas – e *p*, a quantidade de elementos que podem provocar atrasos em um trecho, como o número de interseções por unidade de distância. Um valor apropriado para *k* em um lugar com semáforos isolados é igual a 0,6, enquanto que *k* é igual 0,3 em locais com semáforos coordenados. Para rotatórias e interseções não semaforizadas, *k* igual a 1,0 é apropriado.

Como ponto positivo da função de atraso de Akçelik, ressalta-se a utilização de apenas um parâmetro de calibração (*J*), se a capacidade e a *FFS* forem obtidas em campo. Portanto, a curva do tipo Akçelik parece ser mais adequada do que a função BPR para a integrar o método de alocação de tráfego, justificando-se a escolha neste trabalho. No AIMSUN, a função BPR é padrão, mas a versão do simulador usada neste artigo – versão 8.1.3 integrado com *Application Programming Interface* (API) – permite programar (em Python) a função VDF com o modelo de Akçelik.

**4. COLETA E TRATAMENTO DE DADOS DE TRÁFEGO**

Para estimar a capacidade viária, a velocidade de fluxo livre e o parâmetro *J* de algumas vias e, ainda, validar a alocação de tráfego na rede, foram obtidos dados de tráfego proveniente de 130 sensores instalados pela cidade de Belo Horizonte. Cada sensor – que possui a limitação de ser usado na fiscalização eletrônica de excesso de velocidade ou de avanço semafórico – é capaz de identificar três tipos de veículos: leve (automóvel), motocicleta e pesado (caminhão ou ônibus).

Cada sensor consegue determinar a hora exata, a velocidade, e a faixa onde cada veículo passou. Foram considerados os dados de todos os veículos que passaram por esses sensores durante as terças, quartas e quintas-feiras do mês de agosto de 2015, considerados dias típicos, com picos na parte da manhã e no fim da tarde. A Figura 1 apresenta a localização dos sensores espalhados pela cidade.


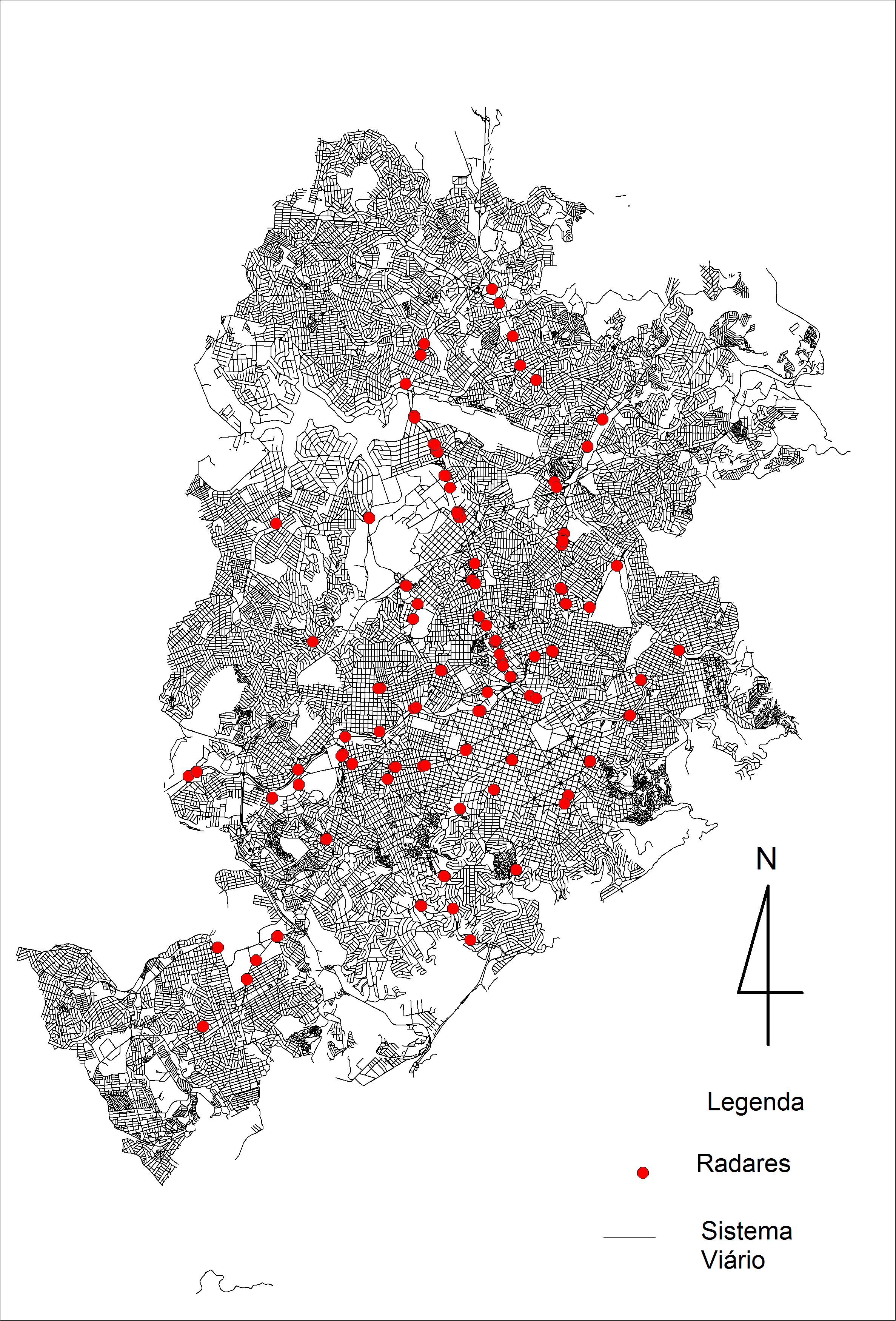


**Figura 1**: Localização dos sensores de tráfego

Os dados de cada um dos 130 sensores foram agregados em intervalos de 15 minutos. Foi utilizado um volume equivalente de 0,33 UCP (Unidade de Carro de Passeio) para motocicletas e 2,5 UCP para veículos pesados, padrão usado pela BHTrans. Foram, assim, calculados os fluxos de veículos equivalentes das correntes de tráfego de cada sensor.

A partir dos dados de tráfego dos sensores, foi usado o software *SPD_CAL,* desenvolvido por Van Aerde M. e Rakha H. (1995) para obter o modelo fluxo-velocidade-densidade de Van Aerde. Esse software utiliza um método heurístico iterativo para calibrar as curvas ao obter quatro parâmetros: *FFS*, velocidade na capacidade, capacidade e densidade de congestionamento (Figura 2).

**Figura 2**: Exemplo do modelo de Van Aerde obtido a partir de dados de tráfego

Segundo Rakha H. e Crowther B. (2002), o modelo de tráfego de Van Aerde é uma junção de dois outros modelos mais simplificados. O mais simples deles, o modelo de Greenshields, requer dois parâmetros de calibração: a velocidade de fluxo livre e a capacidade ou a densidade de congestionamento. O segundo, o modelo de *car-following* de Pipe, requer três parâmetros de calibração: a velocidade de fluxo livre, a densidade de congestionamento e um fator de sensibilidade do motorista. O modelo de Van Aerde demonstra um nível maior de liberdade para refletir diferentes comportamentos de tráfego dentre as diversas características do sistema. O modelo consegue retratar bem desde vias lentas, como as locais, às vias expressas, de fluxo elevado.

Dos 130 sensores de tráfego estudados, somente 20 apresentaram uma dispersão dos pontos adequada para obtenção do Modelo de Van Aerde, chegando na capacidade. Os sensores estavam posicionados, principalmente, nas avenidas Amazonas, Antônio Carlos e Cristiano Machado, consideradas do tipo arterial. Em muitos casos, os dados possuíam uma dispersão semelhantes.

Akçelik (1991) sugere valores de *J* de acordo com o tipo de via, como mostra a Tabela 1. O trabalho não apresenta uma formulação para encontrar *J* com base em variáveis do tráfego obtidas em campo, como a *FFS* e a capacidade, para vias urbanas. No entanto, é possível estimar o formato da função de Akçelik pela relação fluxo-velocidade com base na premissa de que as curvas devem ser semelhantes antes de atingirem a capacidade. Com os valores de *FFS* e capacidade encontrados com o modelo de Van Aerde, o formato da função de Akçelik é determinado pelo parâmetro *J*, encontrado por “tentativa e erro”. Um exemplo dessa aplicação pode também ser visto na Figura 2. Foram determinados dois subtipos de funções para as vias arteriais:

- Arterial 1: capacidade = 1300 cpe/h.faixa, *FFS* = 55 km/h e *J* = 0,5; e
- Arterial 2: capacidade = 850 cpe/h.faixa, *FFS* = 50 km/h e *J* = 1,0.

Como não havia sensores instalados nos outros tipos de vias – expressa, coletoras e locais – foi aplicado um AG para encontrar os parâmetros *J* e os valores da capacidade e da velocidade de fluxo livre.

**5. APLICAÇÃO DO AG E ALOCAÇÃO DE TRÁFEGO**

A rede simulada foi georreferenciada e conta com características diversas, tais como: hierarquia viária, número de faixas, conversões permitidas e proibidas, velocidade máxima permitida, largura da faixa, capacidade, presença ou ausência de acostamento e linhas de ônibus. A rede modelada conta com 36201 interseções e 16695 km de extensão; parte dessa rede foi modelada pela BHTrans, mas 35% do total foi modelada durante o desenvolvimento deste trabalho. Também foram adicionadas vias principais de cidades da Região Metropolitana de Belo Horizonte (RMBH) para representarem o fluxo de veículos entre essas regiões e a capital mineira.

Pode-se dizer que é praticamente impossível gerar um modelo de alocação de tráfego no qual todos os tempos de viagem entre os pares OD sejam perfeitamente iguais. Portanto, para alocar o tráfego estaticamente no AIMSUN, deve ser informado qual é o critério de parada do algoritmo de alocação: se um número máximo de iterações ou um “*gap* relativo” mínimo, que representa a diferença média dos tempos de deslocamento entre os pares OD. Neste trabalho, foram adotadas ou 50 iterações ou um *gap* relativo mínimo de 3% como critério de parada.

**5.1. Matriz OD**

A matriz OD é uma estimativa do volume e das características dos deslocamentos realizados pela população de uma região entre áreas de geração e de atração de viagens durante um determinado intervalo de tempo. A Matriz OD da RMBH foi construída a partir de uma pesquisa socioeconômica realizada através de uma parceria entre as Secretarias de Gestão Metropolitana (SEGEM) e de Transportes e Obras Públicas (SETOP) em 2012.

O objetivo principal da Pesquisa Origem e Destino – Pesquisa OD – foi identificar as necessidades de mobilidade da população da RMBH e conjugar tais necessidades com as características socioeconômicas desta população. Isto se tornou possível por meio do cruzamento de dados domiciliares, individuais e de trajetos coletados na referida pesquisa. Foram entrevistados cerca de 1% dos moradores de domicílios localizadas nos trinta e quatro municípios, totalizando 39958 questionários, sendo 19360 deles em Belo Horizonte (Batista, 2012). Essa matriz OD obtida foi integralmente modelada no AIMSUN sem ajustes.

**5.2. Aplicação do AG**

Para realizar a calibração e a validação do Modelo de Akçelik, foi utilizado um AG com características semelhantes aos de outras aplicações em redes microssimuladas para calibrar parâmetros de modelos comportamentais (Bessa Jr. e Setti, 2011; Moreno *et al*., 2014; Bessa Jr. e Setti, 2015) e de modelos de desempenho (Bessa Jr. *et al*., 2008).

O método consiste em produzir, aleatoriamente, uma população inicial de indivíduos (soluções) que evolui ao longo de gerações com base no seu grau de adaptação ao meio (função *fitness*). Para a população evoluir, é necessário que os melhores indivíduos (mais bem adaptados) transmitam seu material genético aos seus filhos (por meio do cruzamento, ou *crossover*). Além disso, outros operadores genéticos são usados, como a mutação de seus genes (parte de um indivíduo) e a predação (ou eliminação predatória) daqueles indivíduos menos adaptados ao meio que dão lugar a outros produzidos aleatoriamente. Maiores detalhes sobre o uso de AG’s para calibrar simuladores de tráfego podem ser obtidas em Bessa Jr. (2015).

Neste trabalho, o AG desenvolvido buscava encontrar uma solução (indivíduo) cujo os valores de *J*, *FFS* e capacidade das funções de atraso produzissem volumes de tráfego simulados próximos daqueles encontrados nos 130 sensores estudados, para um determinado período de tempo. Como o AG é uma técnica que exige simular a mesma rede muitas vezes (com diferentes soluções), a rede viária usada na calibração foi reduzida para ser simulada mais rapidamente, levando em conta a subrede próxima do encontro entre a Av. D. Pedro II e o Anel Rodoviário de Belo Horizonte (Figura 3). O espaço de busca dos parâmetros pode ser visto na Tabela 2, estabelecido com base nos parâmetros sugeridos por Akçelik (1991) mostrados na Tabela 1.


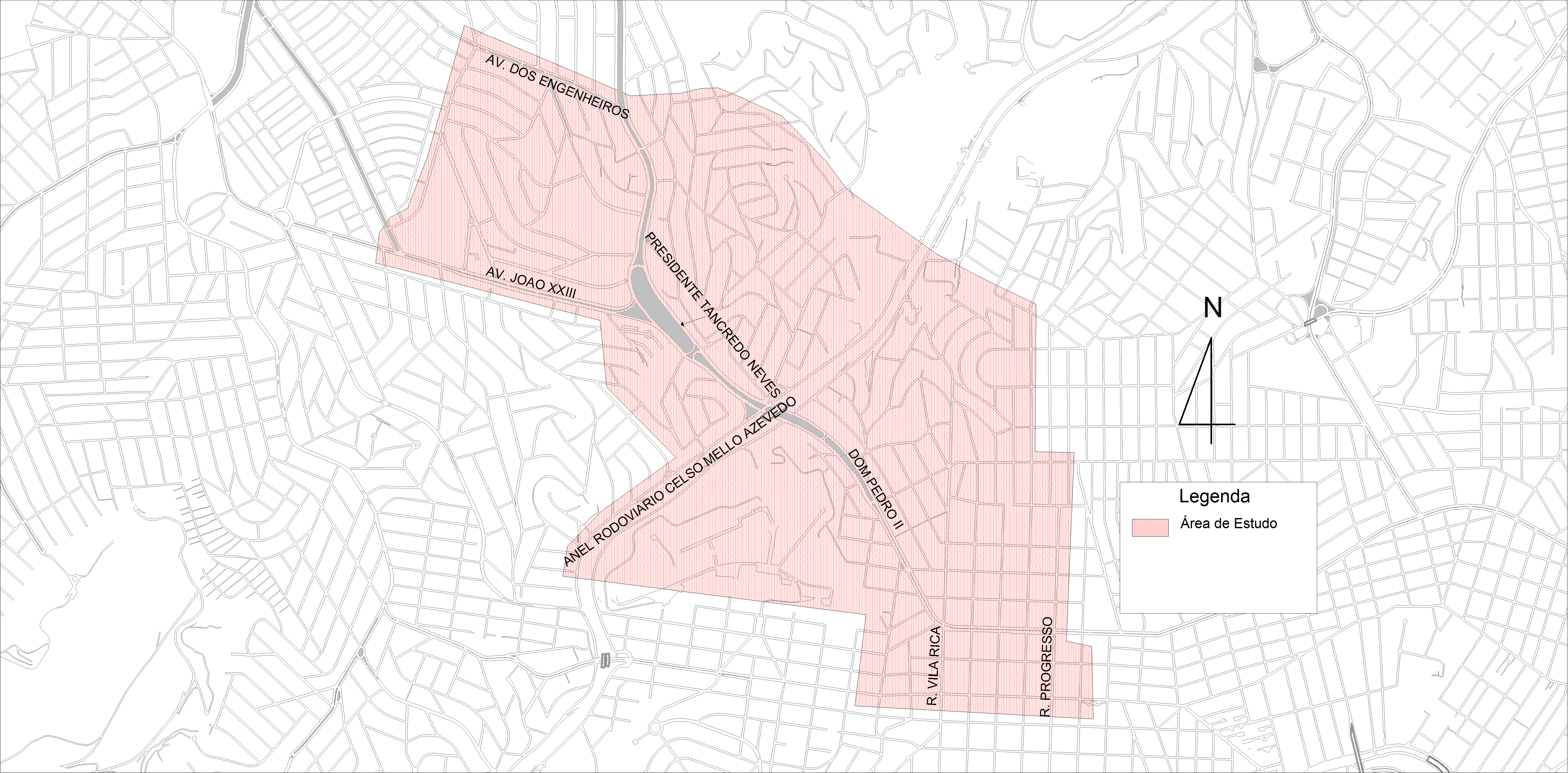


**Figura 3**: Parte do município de Belo Horizonte e subrede usada na aplicação do AG

**Tabela 1**: Valores *default* dos parâmetros de calibração

| Tipo de via | Capacidade (cpe/h.faixa) |  | *FFS* (km/h) |  | *J* |
| --- | --- | --- | --- | --- | --- |
| Expressa | 1800 |  | 100 |  | 0,20 |
| Coletora 1 | 900 |  | 60 |  | 0,80 |
| Coletora 2 | 900 |  | 60 |  | 0,80 |
| Via Local 1 | 600 |  | 40 |  | 1,60 |
| Via Local 2 | 600 |  | 40 |  | 1,60 |

**Tabela 2**: Espaço de busca dos parâmetros de calibração do AG

| Tipo de via | Capacidade (cpe/h.faixa) | |  | *FFS* (km/h) | |  | *J* | |
| --- | --- | --- | --- | --- | --- | --- | --- | --- |
|  | mínimo | máximo |  | mínimo | máximo |  | mínimo | máximo |
| Expressa | 1000 | 2000 |  | 60 | 120 |  | 0,10 | 0,60 |
| Coletora 1 | 700 | 1100 |  | 40 | 70 |  | 0,60 | 1,20 |
| Coletora 2 | 700 | 1100 |  | 40 | 70 |  | 0,60 | 1,20 |
| Via Local 1 | 400 | 800 |  | 30 | 50 |  | 1,20 | 2,00 |
| Via Local 2 | 400 | 800 |  | 30 | 50 |  | 1,20 | 2,00 |

A população foi composta por 20 indivíduos e o critério de parada do algoritmo foi o número máximo de gerações igual a 50. Foi considerado um *crossover* com critério de seleção do tipo *elitismo* e taxas de diversidade (predação e mutação), respectivamente, iguais a 30% e 20%, sendo aplicadas a cada 2 gerações. A função *fitness* foi a raiz do erro quadrático médio entre os volumes de tráfego observados e simulados, conforme mostra a equação:

(4)

em que:

*n =* total de sensores;

*y*i = i-ésimo volume de tráfego simulado; e

*x*i = i-ésimo volume de tráfego observado em campo.

A escolha pelo *RMSE* justifica-se por ela penalizar mais as grandes diferenças entre os volumes observados e simulados, ao contrário de outras funções *fitness* como o coeficiente de correlação – que indica proporcionalidade e não igualdade – e o erro médio percentual – que trata erros grandes, de valores absolutos diferentes e iguais em termos percentuais, da mesma forma (Hollander e Liu, 2008). A simulação da rede foi conduzida considerando-se o horário de pico da manhã, entre 7h e 8h. A matriz OD foi ajustada para os dados do deslocamento da população referentes a esse período.

Usando os parâmetros *default* da função VDF (Tabela 1), foi encontrado um valor de *RMSE* de 462 para a subrede alocada. Esse valor é considerado bom (baixo) e foi obtido pelo simulador por se tratar de uma rede viária relativamente pequena, de 111 km e 301 interseções. A escolha por essa rede justifica-se por conter todos os tipos de vias a serem calibrados e com dados de campo numa quantidade suficiente para fazer uma avaliação da alocação de tráfego.

A aplicação da melhor solução encontrada com o AG (Tabela 3) na alocação de tráfego com o AIMSUN proporcionou um valor de *RMSE* de 366, ou 21% menor do que quando usado os valores *default* dos parâmetros. Portanto, embora o uso dos parâmetros *default* tenha proporcionado um valor baixo para a função *fitness*, a aplicação do AG permitiu melhorar ainda mais a alocação do tráfego, com a obtenção de volumes de tráfego atribuídos mais próximos dos volumes de tráfego observados.

**Tabela 3**: Melhor solução encontrada pelo AG

| Tipo de via | Capacidade (cpe/h.faixa) |  | *FFS* (km/h) |  | *J* |
| --- | --- | --- | --- | --- | --- |
| Expressa | 1780 |  | 87 |  | 0,44 |
| Coletora 1 | 1090 |  | 62 |  | 0,79 |
| Coletora 2 | 800 |  | 47 |  | 1,11 |
| Via Local 1 | 440 |  | 32 |  | 1,30 |
| Via Local 2 | 510 |  | 38 |  | 1,70 |

Com relação aos resultados dos parâmetros calibrados, observa-se uma coerência entre os valores de *FFS*, capacidade e os valores de *J*. Quanto maior foram os dois primeiros, menor foi o último. Vale a pena observar também os valores encontrados para as vias coletoras e vias locais, com dois subtipos cada. Foram obtidos parâmetros com valores diferentes dentro de cada hierarquia viária, o que suporta a hipótese de que era mesmo necessário encontrar mais de uma função VDF para cada tipo de via.

Usando os parâmetros *default* da Tabela 1 para alocar o tráfego de toda a RMBH no AIMSUN, o valor de *RMSE* obtido foi de 1390. Foram usados os parâmetros calibrados e obtido um valor de *RMSE* igual a 1279, ou 8% menor do que quando foram usados os valores *default*. Os valores de *RMSE* encontrados na etapa de validação foram maiores do que aqueles encontrados na etapa de calibração. Isso ocorre porque a rede de tráfego é bem maior na etapa de validação, o que configura como um problema mais complexo a ser solucionado. Mesmo assim, a melhora de 8% na função *fitness* com os valores calibrados sugere que o método cumpriu a meta de proporcionar uma alocação de tráfego mais eficiente do que quando se usa parâmetros sugeridos na literatura.

**6. CONSIDERAÇÕES FINAIS**

Este artigo teve como meta desenvolver um AG para estimar parâmetros de calibração da função de atraso de Akçelik para alocar o tráfego com o AIMSUN. Esse tipo de aplicação de AG não foi encontrado na literatura, embora seja comum o uso de AG’s para ajustar parâmetros comportamentais (*car-following*, aceitação de brechas e de ultrapassagens) e de modelos de desempenho em redes microssimuladas.

Os resultados da aplicação do AG mostraram uma melhoria da função *fitness* *RMSE* tanto na etapa de calibração como na etapa da validação em relação aos resultados encontrados com os parâmetros *default*. Os níveis de melhorias em cada etapa foram diferentes, o que ocorre por se tratar de redes viárias de tamanho e complexidade distintos. Recomenda-se a análise de outras funções *fitness* com o objetivo de continuar a análise dos parâmetros de calibração obtidos com a aplicação do AG. Hollander e Liu (2008) apresentaram uma lista de funções *fitness* que são candidatas para realizar a análise proposta.

No intuito de melhorar os resultados obtidos, seria importante obter mais dados de tráfego próximos a capacidade dentro da RMBH, se possível com dados que não sejam provenientes de fiscalização eletrônica, muito influenciados pelo comportamento do usuário ao visualizar o equipamento. Recomenda-se a aplicação do método proposto com o AG em outras áreas de Belo Horizonte para realizar a calibração dos parâmetros, assim como em outras cidades tanto do porte de Belo Horizonte como de médio porte.

Seria interessante aplicar o AG desenvolvido para calibrar outras funções VDF e comparar os resultados com os provenientes da aplicação do AG para calibrar a função de Akçelik. Recomenda-se, também, a fim de obter resultados mais confiáveis, avaliar se a Matriz OD obtida das pesquisas socioeconômicas não precisa ser ajustada e reavaliada. Poderia ser avaliado, também, outro método de alocação de tráfego estática, como o Método “tudo ou nada” ou o Incremental.

**Agradecimentos**

Os autores agradecem o apoio do Setec-MEC/CNPq pelo suporte financeiro sob a forma de auxílio a Projeto de Extensão. Agradecem, ainda, à FAPEMIG e ao CEFET-MG pelo auxílio para participação no congresso. Os autores também agradecem a GESIT/BHTrans pela parceria no Projeto de Extensão que originou este trabalho.

**REFERÊNCIAS**

Akçelik, R. (1991) Travel time functions for transport planning purposes: Davidson's function, its time-dependent. *Australian Road Research*, v. 21, n.3, p. 49–59.

Andrade, G. R.; J. R. Setti e A. C. C. Ferraz (2015) Compatibilidade entre funções de atraso e o HCM em rodovias Paulistas. *Transportes*, v. 23, n. 3, p. 81-89.

Barceló, J. (2010) *Fundamentals of Traffic Simulation*. Department of Statistics & Operations Research Universitat Politècnica de Catalunya, Barcelona.

Batista, A. M. (2012) *Pesquisa Origem e Destino 2011-2012*. Governo do Estado de Minas Gerais.

Bessa Jr., J. E. (2015) Medidas de desempenho para avaliação da qualidade de serviço em rodovias de pista simples brasileiras. 128p. Tese (Doutorado) EESC-USP, São Carlos.

Bessa Jr., J. E.; F. A. A Lima; A. L. B. N. Cunha e J. R. Setti (2008) Calibração do modelo de desempenho do simulador Integration através de um algoritmo genético. XXII ANPET Congresso de Pesquisa e Ensino em Transportes, *Anais...*, Fortaleza-CE, CD-ROM.

Bessa Jr., J. E. e J. R. Setti (2011) Derivation of ATS and PTSF Functions for Two-lane, Rural Highways in Brazil.6th International Symposium on Highway Capacity and Quality of Service, *Procedia Social and Behavioral Sciences*, n. 16, p. 282–292, Stockholm, Sweden.

Bessa Jr., J. E. e J. R. Setti (2015) Avaliação de medidas de desempenho para rodovias de pista simples obtidas a partir de relações fluxo-velocidade. XXIX Congresso Pesquisa e Ensino em Transportes, *Anais...*, Ouro Preto - MG.

BPR (1964) *Traffic Assignment Manual*. U.S. Department of Commerce, Urban Planning division, Washington, D.C., EUA

Dowling, R. e A. Skabardonis (2006) *Urban Arterial Speed-Flow Equations for Travel Demand Models*. Innovations in Travel Demand Modeling: Summary of a Conference. Transportation Research Board.

Hellinga, B. (1998) Requirements for the validation and calibration of traffic simulation models. *Proceedings of Canadian Society for civil engineering*, p. 211-222.

Hollander, Y. e R. Liu (2008) The principles of calibrating traffic microsimulation models. *Transportation*, Springer Science and Business Media, LLC, v. 35, p. 347-362. Published online: 15 January 2008.

Ma, T. e B. Abdulhai (2002) Genetic Algorithm-based Optimization Approach and generic tool for calibration traffic microscopic simulation parameters. *Transportation Research Record 1800*,TRB, National Research Council, Washington, D.C., p. 6-15.

Ma, J.; H. Donge H. M. Zhang (2007) Calibration of microsimulation with heuristic optimization methods. *Transportation Research Record 1999*,TRB, National Research Council, Washington, D.C., p. 208-217.

Machado, O. F. e P. C. M. Ribeiro (2003) Calibração da Função de Capacidade do BPR para uma Via Expressa Brasileira. *Anais do I Rio de Transportes*, Congresso de Ensino e Pesquisa do Estado do Rio de Janeiro, Rio de Janeiro, v. 1, p. 1-12.

Medeiros, A. L.; M. M. Castro Neto; C. F. G. Loureiro e J. E. Bessa Jr. (2013) Calibração de redes viárias urbanas microssimuladas com o uso de algoritmos genéticos. XXVII ANPET Congresso de Pesquisa e Ensino em Transportes, *Anais...*, Belém-PA, 2013.

Mon-Ma, M. L. (2008) *Adaptação do HCM-2000 para rodovias de pista simples com faixas adicionais típicas do Estado de São Paulo*.162p. Tese (Doutorado) EESC-USP, São Carlos.

Moreno, A. T.; C. Llorca; J. E Bessa Júnior; A. Garcia e D. K. Hale (2014) Evaluación de medidas de comportamiento del tráfico considerando las zonas de adelantamiento de carreteras convencionales mediante microsimulación. *Carreteras*, v. 198, p. 15-29.

Park, B. e H. Qi (2005) Development and evaluation of a procedure for the calibration of simulation models. *Transportation Research Record 1934*,TRB, National Research Council, Washington, D.C., p. 208-217.

Portugal, L. S. (2005) *Simulação de tráfego: Conceitos e técnicas de modelagem*. Editora Interciência, Rio de Janeiro.

Pursula, M. (1999) Simulation of Traffic Systems - An Overview. *Journal of Geographic Information and Decision Analysis*, v. 3, n. 1, p. 1-8.

Rakha H. e B. Crowther (2002) Comparison of Greenshields, Pipes, and Van Aerde Car-following and Traffic Stream Models. *Transportation Research Record*, n. 1802, p. 248-262

TSS (2016) *A brief history of TSS*. Transport Simulation System - TSS. Disponível em: <https://www.
AIMSUN.com/wp/?page_id=6076>. Acesso em: 15/07/2016.

TSS (2015) *AIMSUN User’s Manual – Version 8.1*. Transport Simulation System – TSS.

Van Aerde M. e Rakha H. (1995) Multivariate Calibration of Single-Regime Speed-Flow-Density Relationships. *Proceedings of Vehicle Navigation and Information Conference,* IEEE, Piscataway NJ, USA, p. 334-341.

*Endereço para contato:*

Gustavo Henrique Gomes Santos (gustavohgsantos@gmail.com)

Prof. Dr. José Elievam Bessa Júnior (elievamjr@gmail.com)

Heron Fonseca Pimenta (heronfonseca_93@hotmail.com)

Centro Federal de Educação Tecnológica de Minas Gerais – CEFET-MG, Departamento de Engenharia de Transportes - DET

Av. Amazonas 5.253, Nova Suiça, Belo Horizonte, MG, 30.421-169

Eng. Vinícius de Magalhães (viniciusm@pbh.gov.br)

Empresa de Transportes e Trânsito de Belo Horizonte - BHTrans, Gerência de Simulação de Tráfego e Programação Semafórica - GESIT

Av. Eng. Carlos Goulart, 900, Buritis, Belo Horizonte, MG, 30.455-902
