# Supplementary material for: Traffic light optimization using non-dominated sorting genetic algorithm (NSGA2)
Source: Sci Rep. 2023 Sep 20;13:15550. doi: 10.1038/s41598-023-38884-2 (PMC10511403; doi:10.1038/s41598-023-38884-2)
Supplement: Supplementary file 1 — Supplementary Information. [file 41598_2023_38884_MOESM1_ESM.zip › dadosBHTrans/dados]

Sistema de Controle de Tráfego Urbano OPTIMUS

INTENSIDADE DE 4 PONTOS DE MEDIDA
DADOS DE 5 MINUTOS

PONTO DE MEDIDA 1:PM 0401003 (Pouso Alegre)
PONTO DE MEDIDA 2:PM 0401004 (Pouso Alegre)
PONTO DE MEDIDA 3:PM 0401006 (Curvelo)
PONTO DE MEDIDA 4:PM 0402001 (Itajubá)
DESDE:14/05/2015 00:00
ATÉ:15/05/201500:00

INTENSIDADE / 5 MINUTOS

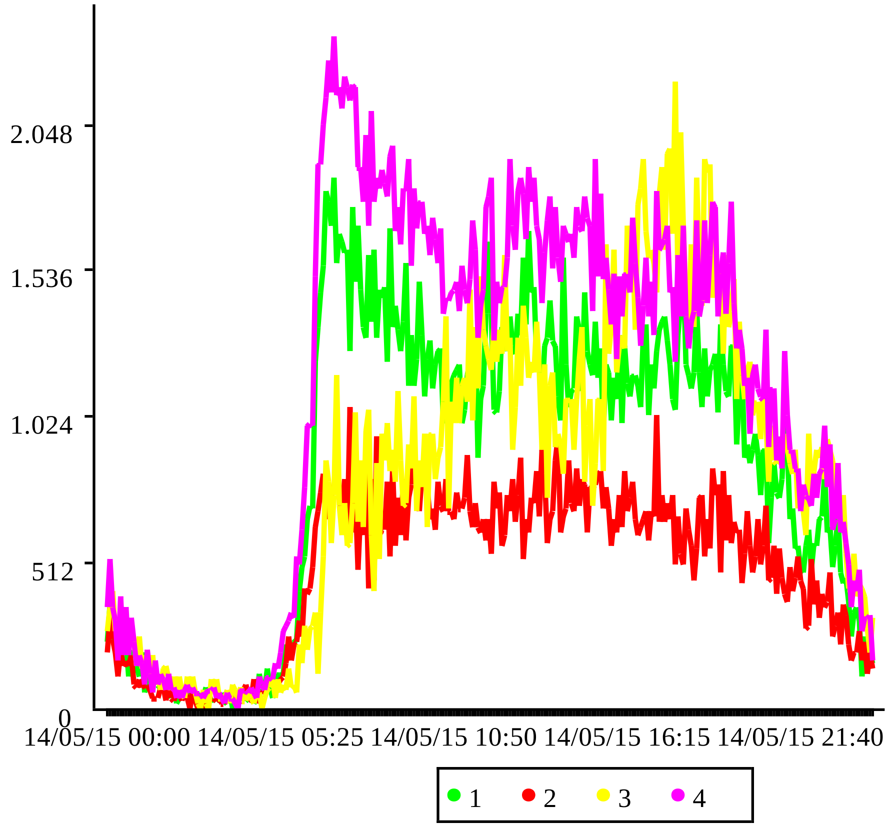

5 MINUTOS	INTENSIDADE			
	PM 0401003	PM 0401004	PM 0401006	PM 0402001
14/05/15 00:00	228	192	264	348
14/05/15 00:05	360	312	348	516
14/05/15 00:10	240	240	408	348
14/05/15 00:15	240	192	264	300
14/05/15 00:20	216	108	264	168
14/05/15 00:25	252	204	288	384
14/05/15 00:30	264	144	312	180
14/05/15 00:35	228	168	240	348
14/05/15 00:40	108	144	276	180
14/05/15 00:45	204	180	288	312
14/05/15 00:50	180	84	228	228
14/05/15 00:55	144	72	144	144
14/05/15 01:00	108	96	252	180
14/05/15 01:05	192	72	132	156
14/05/15 01:10	48	84	180	84

Sistema de Controle de Tráfego Urbano OPTIMUS

5 MINUTOS	INTENSIDADE			
	PM 0401003	PM 0401004	PM 0401006	PM 0402001
14/05/15 01:15	108	84	144	204
14/05/15 01:20	144	60	108	120
14/05/15 01:25	60	36	180	48
14/05/15 01:30	144	36	84	168
14/05/15 01:35	84	36	108	84
14/05/15 01:40	72	72	60	120
14/05/15 01:45	84	72	132	96
14/05/15 01:50	36	24	144	60
14/05/15 01:55	60	108	96	120
14/05/15 02:00	36	36	60	60
14/05/15 02:05	72	24	108	72
14/05/15 02:10	12	48	36	36
14/05/15 02:15	36	48	108	60
14/05/15 02:20	36	24	48	36
14/05/15 02:25	48	60	36	60
14/05/15 02:30	84	36	108	84
14/05/15 02:35	48	0	60	48
14/05/15 02:40	48	60	108	72
14/05/15 02:45	24	24	48	48
14/05/15 02:50	24	0	12	48
14/05/15 02:55	48	36	0	48
14/05/15 03:00	36	24	60	36
14/05/15 03:05	72	0	12	48
14/05/15 03:10	36	24	0	60
14/05/15 03:15	24	36	96	60
14/05/15 03:20	36	36	72	72
14/05/15 03:25	24	24	96	36
14/05/15 03:30	36	36	36	48
14/05/15 03:35	48	12	36	36
14/05/15 03:40	24	12	36	12
14/05/15 03:45	12	36	60	48
14/05/15 03:50	48	36	12	36
14/05/15 03:55	0	48	84	36
14/05/15 04:00	12	12	60	12
14/05/15 04:05	24	12	12	0
14/05/15 04:10	60	24	72	60
14/05/15 04:15	48	60	24	60
14/05/15 04:20	24	84	24	60
14/05/15 04:25	36	24	48	48
14/05/15 04:30	48	36	36	72
14/05/15 04:35	12	96	12	48
14/05/15 04:40	36	12	48	36
14/05/15 04:45	120	48	48	108
14/05/15 04:50	72	48	0	84
14/05/15 04:55	60	60	36	60
14/05/15 05:00	132	84	60	108
14/05/15 05:05	72	84	72	108
14/05/15 05:10	36	96	96	96
14/05/15 05:15	84	84	36	156
14/05/15 05:20	120	84	96	132
14/05/15 05:25	120	96	48	192
14/05/15 05:30	120	108	84	264
14/05/15 05:35	216	156	60	288
14/05/15 05:40	180	252	132	300
14/05/15 05:45	204	168	72	336
14/05/15 05:50	228	216	84	312
14/05/15 05:55	240	228	48	528
14/05/15 06:00	408	300	216	504

Sistema de Controle de Tráfego Urbano OPTIMUS

5 MINUTOS	INTENSIDADE			
	PM 0401003	PM 0401004	PM 0401006	PM 0402001
14/05/15 06:05	492	252	156	624
14/05/15 06:10	528	420	288	768
14/05/15 06:15	660	396	204	984
14/05/15 06:20	708	420	276	996
14/05/15 06:25	696	492	288	984
14/05/15 06:30	1212	636	336	1512
14/05/15 06:35	1332	684	120	1908
14/05/15 06:40	1452	756	336	1908
14/05/15 06:45	1548	816	528	2040
14/05/15 06:50	1812	660	864	2136
14/05/15 06:55	1776	756	792	2268
14/05/15 07:00	1692	744	576	2160
14/05/15 07:05	1860	768	744	2352
14/05/15 07:10	1560	840	1164	2148
14/05/15 07:15	1656	732	708	2172
14/05/15 07:20	1632	672	600	2100
14/05/15 07:25	1596	804	720	2208
14/05/15 07:30	1608	600	564	2172
14/05/15 07:35	1248	1056	576	2124
14/05/15 07:40	1752	612	720	2184
14/05/15 07:45	1488	756	1032	2172
14/05/15 07:50	1692	480	648	1896
14/05/15 07:55	1464	696	864	1896
14/05/15 08:00	1332	600	636	1776
14/05/15 08:05	1296	840	972	2004
14/05/15 08:10	1584	420	1044	1692
14/05/15 08:15	1356	804	600	2088
14/05/15 08:20	1608	444	408	1776
14/05/15 08:25	1296	948	852	1860
14/05/15 08:30	1464	600	516	1824
14/05/15 08:35	1440	624	960	1884
14/05/15 08:40	1476	624	864	1836
14/05/15 08:45	1212	792	996	1788
14/05/15 08:50	1680	528	828	1932
14/05/15 08:55	1332	840	900	1968
14/05/15 09:00	1404	564	792	1668
14/05/15 09:05	1320	732	1104	1752
14/05/15 09:10	1248	600	864	1620
14/05/15 09:15	1356	804	708	1824
14/05/15 09:20	1560	588	720	1812
14/05/15 09:25	1128	732	924	1920
14/05/15 09:30	1308	780	840	1548
14/05/15 09:35	1128	888	1092	1824
14/05/15 09:40	1224	804	684	1680
14/05/15 09:45	1488	684	864	1776
14/05/15 09:50	1308	816	768	1776
14/05/15 09:55	1092	840	960	1656
14/05/15 10:00	1224	768	636	1692
14/05/15 10:05	1284	660	960	1584
14/05/15 10:10	1116	696	960	1716
14/05/15 10:15	1200	624	804	1644
14/05/15 10:20	1248	792	876	1560
14/05/15 10:25	1260	708	912	1680
14/05/15 10:30	996	684	1068	1380
14/05/15 10:35	1056	804	1368	1440
14/05/15 10:40	972	684	696	1428
14/05/15 10:45	1140	684	1068	1452
14/05/15 10:50	1164	660	996	1464

Sistema de Controle de Tráfego Urbano OPTIMUS

5 MINUTOS	INTENSIDADE			
	PM 040100 3	PM 040100 4	PM 040100 6	PM 040200 1
14/05/15 10:55	1188	756	1152	1488
14/05/15 11:00	1200	696	996	1392
14/05/15 11:05	996	696	1128	1548
14/05/15 11:10	1044	732	1152	1464
14/05/15 11:15	1164	888	1068	1416
14/05/15 11:20	1212	684	1464	1500
14/05/15 11:25	1272	636	1008	1704
14/05/15 11:30	1320	720	1332	1608
14/05/15 11:35	876	636	1116	1296
14/05/15 11:40	1080	612	1512	1404
14/05/15 11:45	1128	660	1320	1464
14/05/15 11:50	1380	588	1272	1752
14/05/15 11:55	1632	660	1224	1788
14/05/15 12:00	1380	540	1188	1860
14/05/15 12:05	1044	792	1356	1284
14/05/15 12:10	1032	696	1212	1488
14/05/15 12:15	1104	756	1320	1416
14/05/15 12:20	1332	564	1236	1476
14/05/15 12:25	1320	600	1584	1476
14/05/15 12:30	1248	744	1248	1572
14/05/15 12:35	1368	684	1296	1920
14/05/15 12:40	1272	804	900	1692
14/05/15 12:45	1236	648	1056	1608
14/05/15 12:50	1380	744	1296	1812
14/05/15 12:55	1308	876	1128	1860
14/05/15 13:00	1572	516	1404	1800
14/05/15 13:05	1344	660	1296	1644
14/05/15 13:10	1668	612	1152	1896
14/05/15 13:15	1452	732	1212	1776
14/05/15 13:20	1476	720	1176	1860
14/05/15 13:25	1224	828	1356	1692
14/05/15 13:30	1212	672	1176	1644
14/05/15 13:35	1080	900	900	1416
14/05/15 13:40	1272	816	1200	1596
14/05/15 13:45	1296	576	732	1704
14/05/15 13:50	1428	660	1128	1788
14/05/15 13:55	1284	684	1176	1536
14/05/15 14:00	1272	864	912	1752
14/05/15 14:05	1092	948	960	1572
14/05/15 14:10	1008	612	924	1488
14/05/15 14:15	1572	672	816	1692
14/05/15 14:20	1200	696	1080	1656
14/05/15 14:25	1056	864	1080	1632
14/05/15 14:30	1104	720	1080	1656
14/05/15 14:35	1116	684	900	1572
14/05/15 14:40	1368	840	1080	1752
14/05/15 14:45	1320	708	1224	1692
14/05/15 14:50	1104	804	1332	1668
14/05/15 14:55	1452	780	792	1788
14/05/15 15:00	1248	612	984	1716
14/05/15 15:05	1200	768	1080	1692
14/05/15 15:10	1164	768	708	1392
14/05/15 15:15	1356	780	852	1920
14/05/15 15:20	1152	816	1080	1512
14/05/15 15:25	1260	828	1080	1800
14/05/15 15:30	972	696	828	1500
14/05/15 15:35	1200	768	1620	1572
14/05/15 15:40	1176	696	1236	1464

Sistema de Controle de Tráfego Urbano OPTIMUS

5 MINUTOS	INTENSIDADE			
	PM 040100 3	PM 040100 4	PM 040100 6	PM 040200 1
14/05/15 15:45	1008	564	1344	1344
14/05/15 15:50	1152	660	1608	1524
14/05/15 15:55	1080	612	1176	1224
14/05/15 16:00	1308	744	1272	1512
14/05/15 16:05	996	636	1308	1368
14/05/15 16:10	1272	828	1260	1524
14/05/15 16:15	1140	684	1692	1524
14/05/15 16:20	1092	732	1548	1452
14/05/15 16:25	1164	792	1620	1716
14/05/15 16:30	1164	660	1320	1560
14/05/15 16:35	1128	600	1764	1440
14/05/15 16:40	1056	636	1824	1272
14/05/15 16:45	1308	660	1920	1404
14/05/15 16:50	1344	684	1668	1572
14/05/15 16:55	1020	588	1572	1368
14/05/15 17:00	1200	684	1608	1488
14/05/15 17:05	1116	672	1560	1308
14/05/15 17:10	1248	1020	1452	1812
14/05/15 17:15	1320	648	1800	1608
14/05/15 17:20	1356	744	1896	1620
14/05/15 17:25	1368	648	1608	1644
14/05/15 17:30	1284	720	1944	1692
14/05/15 17:35	1200	660	1956	1452
14/05/15 17:40	1080	744	1656	1476
14/05/15 17:45	1044	504	2196	1212
14/05/15 17:50	1308	672	1464	1584
14/05/15 17:55	1416	540	2016	1368
14/05/15 18:00	1536	504	1692	1692
14/05/15 18:05	1200	696	1428	1368
14/05/15 18:10	1164	624	1320	1260
14/05/15 18:15	1116	564	1620	1356
14/05/15 18:20	1176	444	1332	1392
14/05/15 18:25	1404	552	1860	1704
14/05/15 18:30	1176	732	1560	1368
14/05/15 18:35	1056	744	1404	1428
14/05/15 18:40	1260	528	1920	1704
14/05/15 18:45	1092	660	1872	1416
14/05/15 18:50	1188	552	1908	1584
14/05/15 18:55	1200	840	1440	1776
14/05/15 19:00	1236	744	1560	1752
14/05/15 19:05	1032	780	1512	1368
14/05/15 19:10	1344	468	1548	1524
14/05/15 19:15	1200	828	1236	1596
14/05/15 19:20	1104	588	1584	1380
14/05/15 19:25	1092	744	1332	1560
14/05/15 19:30	1272	576	1392	1776
14/05/15 19:35	1272	648	1500	1416
14/05/15 19:40	924	612	1080	1260
14/05/15 19:45	1080	624	1356	1320
14/05/15 19:50	1176	432	1164	1260
14/05/15 19:55	876	540	1080	1128
14/05/15 20:00	924	684	1188	1152
14/05/15 20:05	852	564	1212	960
14/05/15 20:10	900	468	1020	1164
14/05/15 20:15	960	528	1068	1200
14/05/15 20:20	888	660	1068	1116
14/05/15 20:25	744	504	936	1092
14/05/15 20:30	900	564	1104	1080

Sistema de Controle de Tráfego Urbano OPTIMUS

5 MINUTOS	INTENSIDADE			
	P M 0401003	P M 0401004	P M 0401006	P M 0402001
14/05/15 20:35	900	708	1008	1320
14/05/15 20:40	576	444	792	912
14/05/15 20:45	732	456	984	1116
14/05/15 20:50	852	564	864	1116
14/05/15 20:55	756	396	852	864
14/05/15 21:00	732	552	876	948
14/05/15 21:05	804	456	876	840
14/05/15 21:10	1008	396	960	1248
14/05/15 21:15	912	372	912	1020
14/05/15 21:20	660	492	852	900
14/05/15 21:25	696	408	816	900
14/05/15 21:30	552	456	900	840
14/05/15 21:35	564	528	696	840
14/05/15 21:40	480	444	756	684
14/05/15 21:45	468	420	684	780
14/05/15 21:50	564	276	600	744
14/05/15 21:55	624	288	960	732
14/05/15 22:00	480	516	756	708
14/05/15 22:05	576	360	828	816
14/05/15 22:10	564	444	900	732
14/05/15 22:15	660	312	876	816
14/05/15 22:20	672	396	912	840
14/05/15 22:25	816	348	804	984
14/05/15 22:30	612	372	936	768
14/05/15 22:35	636	468	900	924
14/05/15 22:40	492	252	864	624
14/05/15 22:45	564	288	744	660
14/05/15 22:50	672	336	804	852
14/05/15 22:55	468	216	612	612
14/05/15 23:00	432	360	744	648
14/05/15 23:05	456	324	540	576
14/05/15 23:10	372	216	420	504
14/05/15 23:15	252	168	420	348
14/05/15 23:20	336	192	540	444
14/05/15 23:25	348	192	384	408
14/05/15 23:30	300	264	432	480
14/05/15 23:35	108	168	420	264
14/05/15 23:40	252	228	384	312
14/05/15 23:45	240	120	276	312
14/05/15 23:50	192	192	192	324
14/05/15 23:55	156	132	312	168
