# Supplementary material for: Traffic light optimization using non-dominated sorting genetic algorithm (NSGA2)
Source: Sci Rep. 2023 Sep 20;13:15550. doi: 10.1038/s41598-023-38884-2 (PMC10511403; doi:10.1038/s41598-023-38884-2)
Supplement: Supplementary file 1 — Supplementary Information. [file 41598_2023_38884_MOESM1_ESM.zip › dadosBHTrans/dados BHTrans 2019/22_05_2019/dados2.pdf]

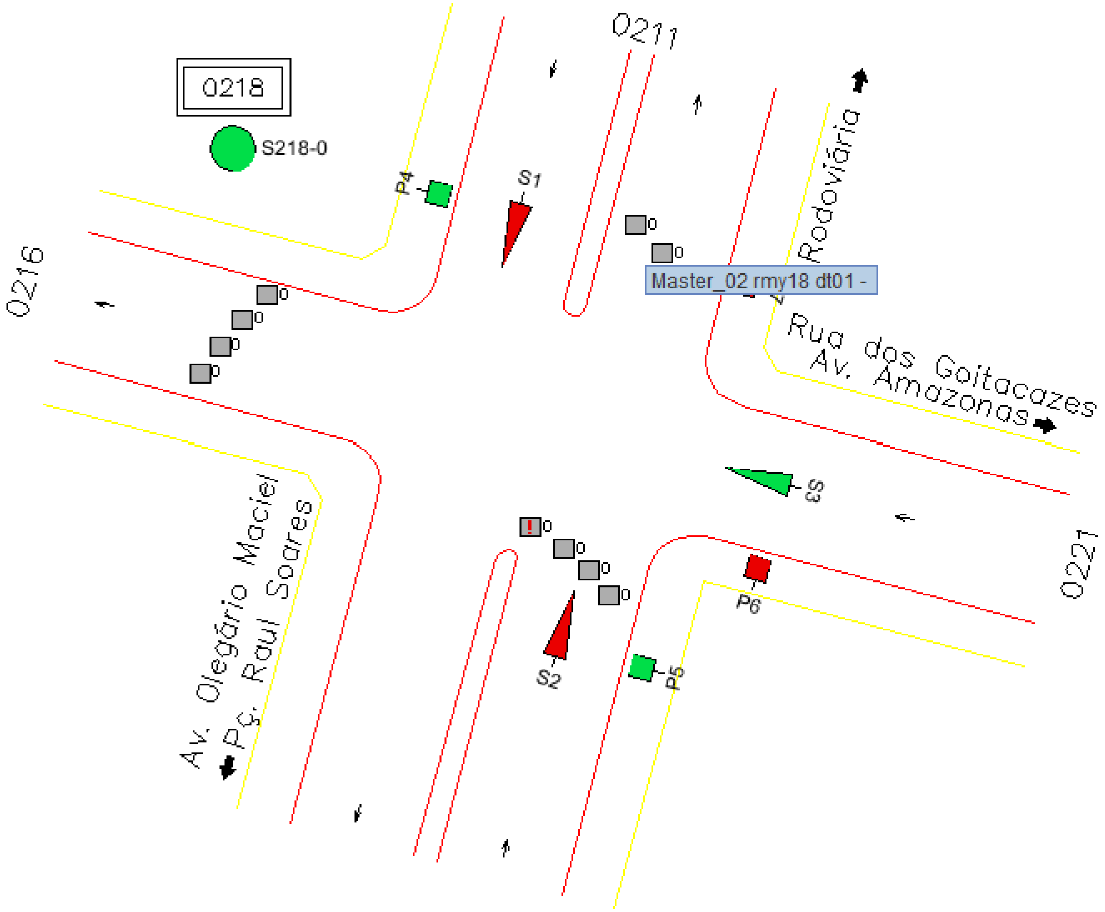
0218
S218-0
0211
0216
0221
P4
S1
S2
S3
P5
P6
Master_02 rmy18 dt01 -
Rodoviária
Rua dos Goitacazes
Av. Amazonas
Av. Olegário Maciel
Pç. Raul Soares

S2:3-6

S3:7-10

S4:1-3, S3:4-6

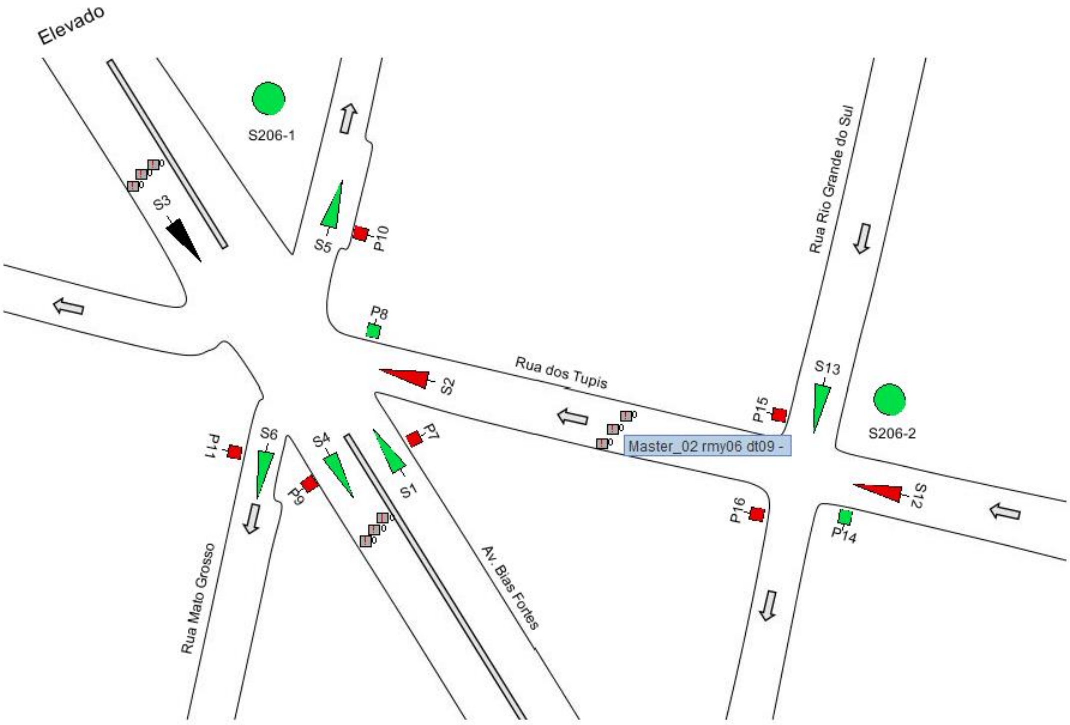
Elevado
S206-1
S3
S5
P10
P8
Rua dos Tupis
S2
Rua Rio Grande do Sul
S13
S206-2
P15
Master_02 rmy06 dt09 -
S6
S4
P7
P11
P9
S1
P16
S12
P14
Rua Mato Grosso
Av. Bias Fortes

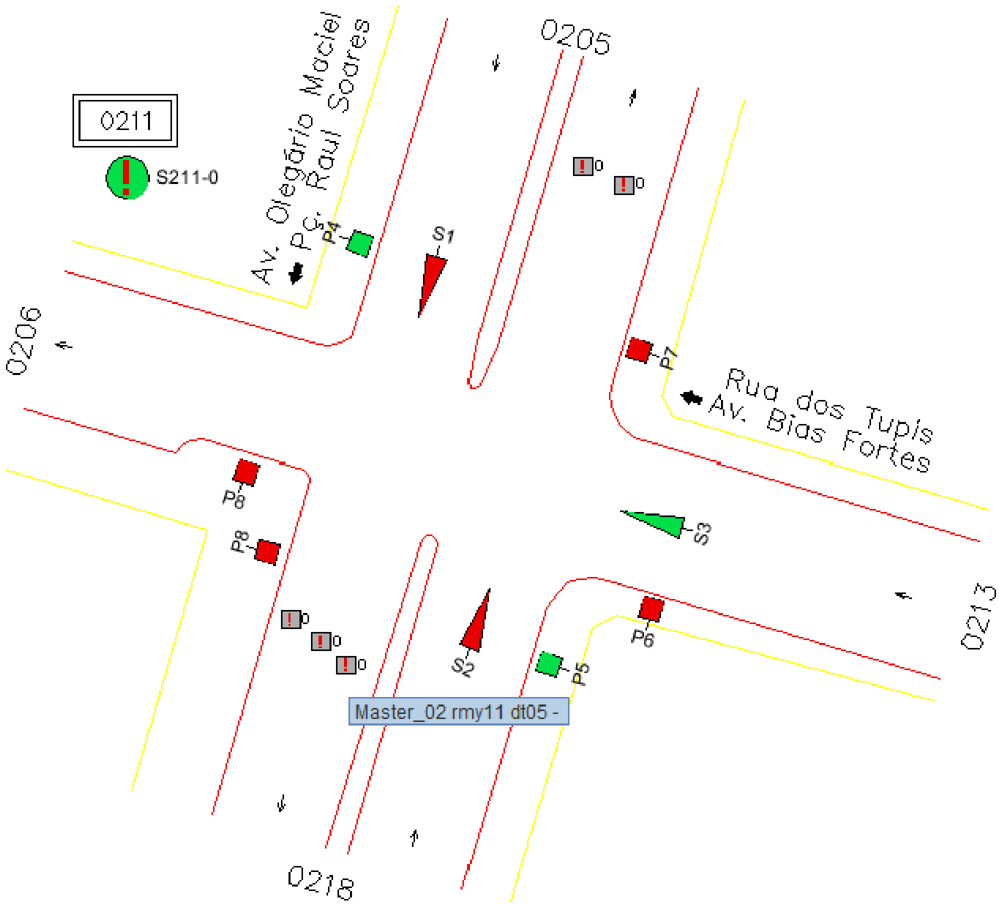
0211
S211-0
Av. Olegário Maciel
Pç. Raul Soares
0205
S1
P4
0206
P7
Rua dos Tupis
Av. Bias Fortes
P8
P8
S3
P6
0213
S2
P5
Master_02 rmy11 dt05 -
0218

S1:3-5, S2:1-2

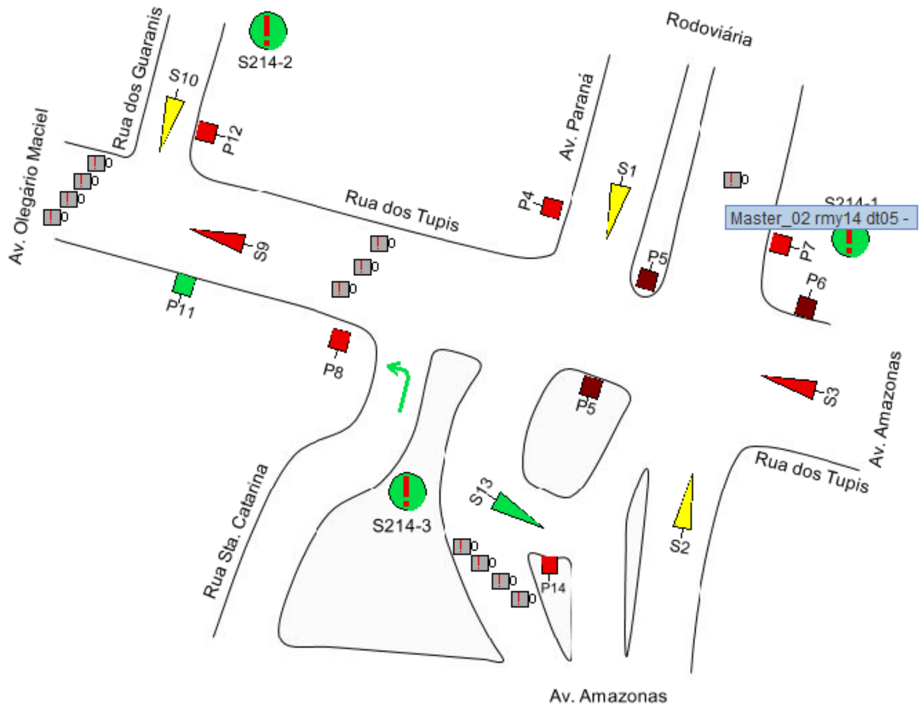

S13:1-4, S9:6-8, S10:9-12, S2:5

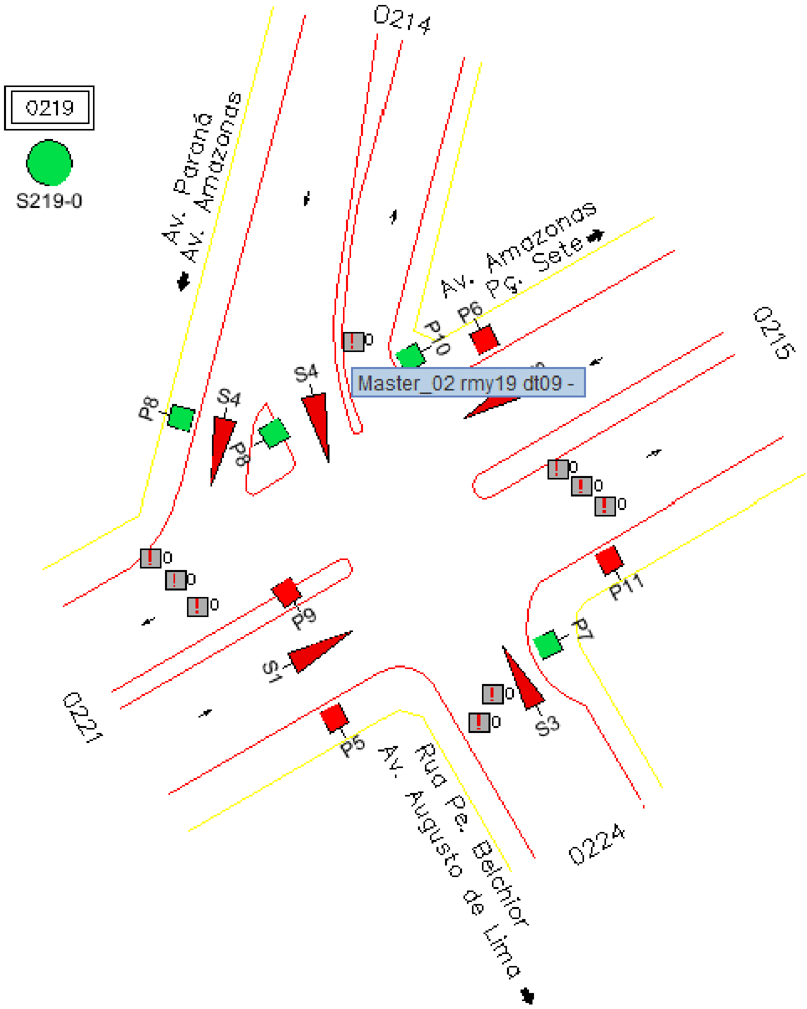

S1:1-3, S3:4-5, S2:6-8,

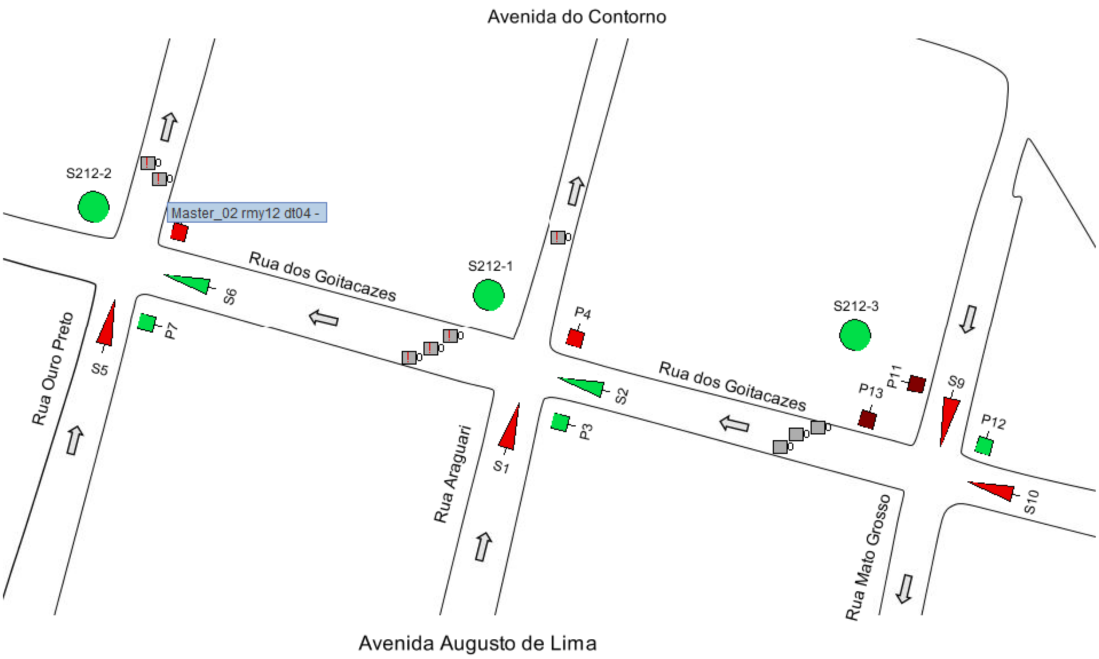

S1:6, S2:6-8 S6:1-3

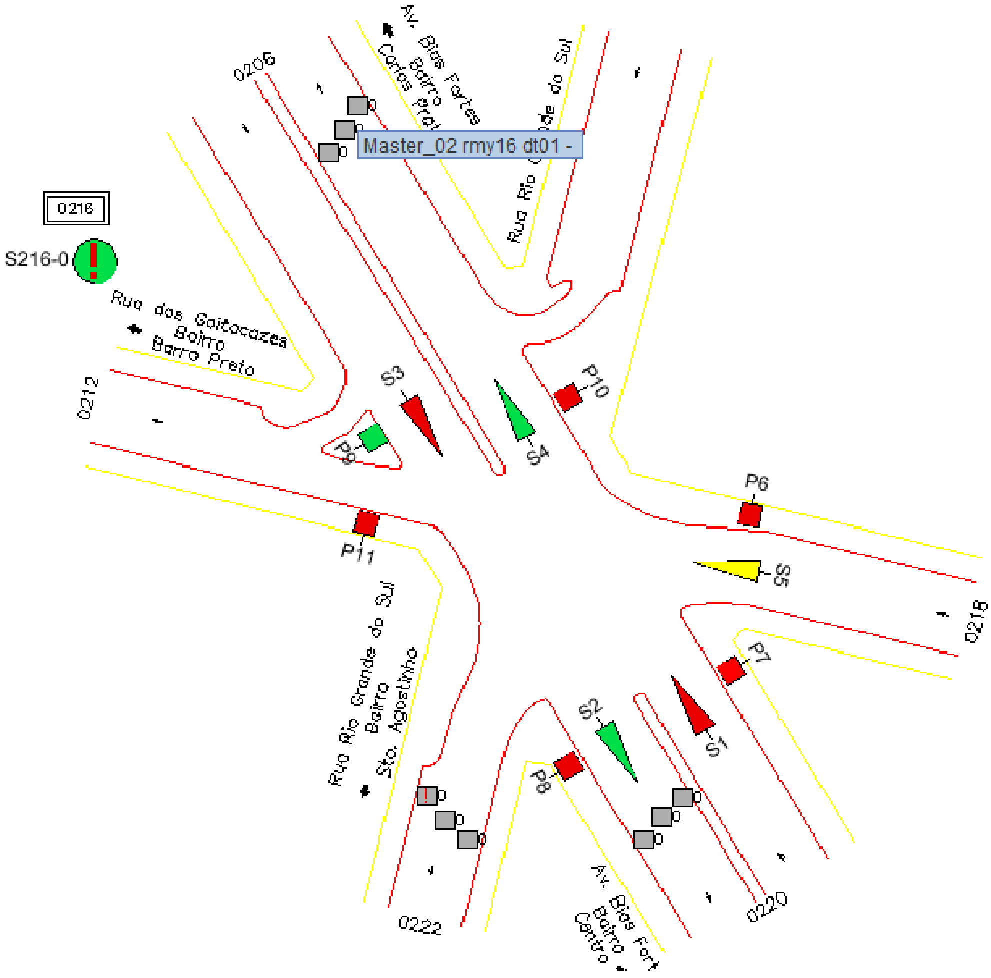

S2:4-6, S5:7-9, S4:1-3

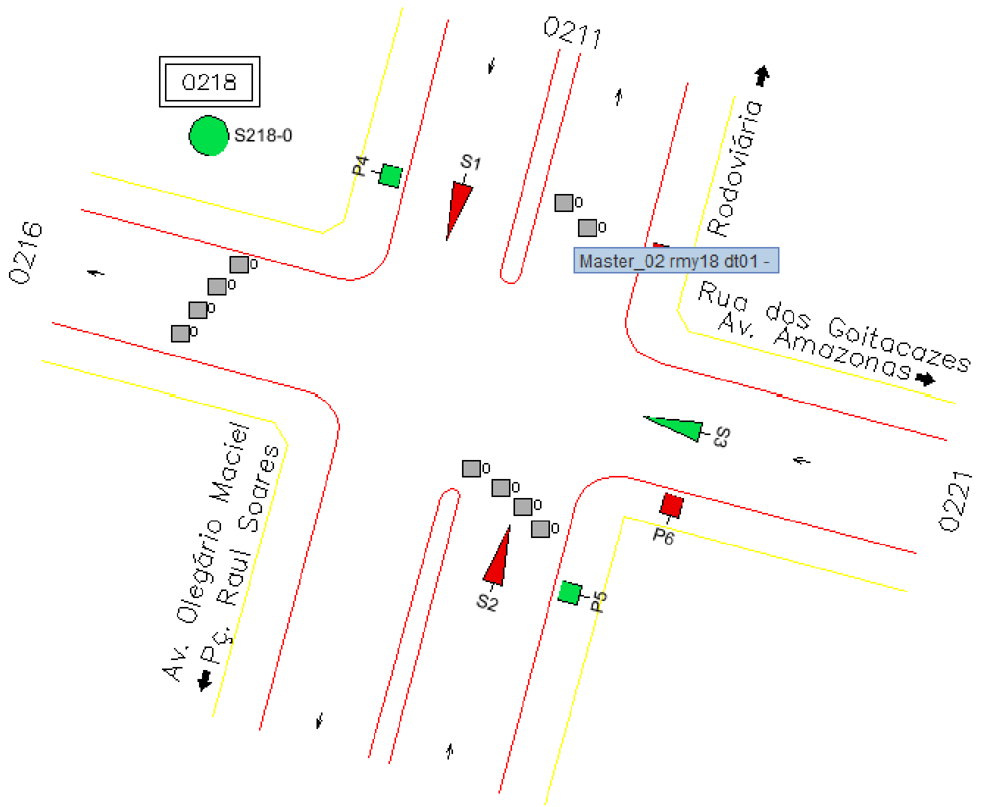
0218
S218-0
0211
0216
0221
S1
S2
S3
P4
P5
P6
Master_02 rmy18 dt01 -
Rodoviária
Rua dos Goitacazes
Av. Amazonas
Av. Olegário Maciel
Pç. Raul Soares

S2:3-6, S3:7-10

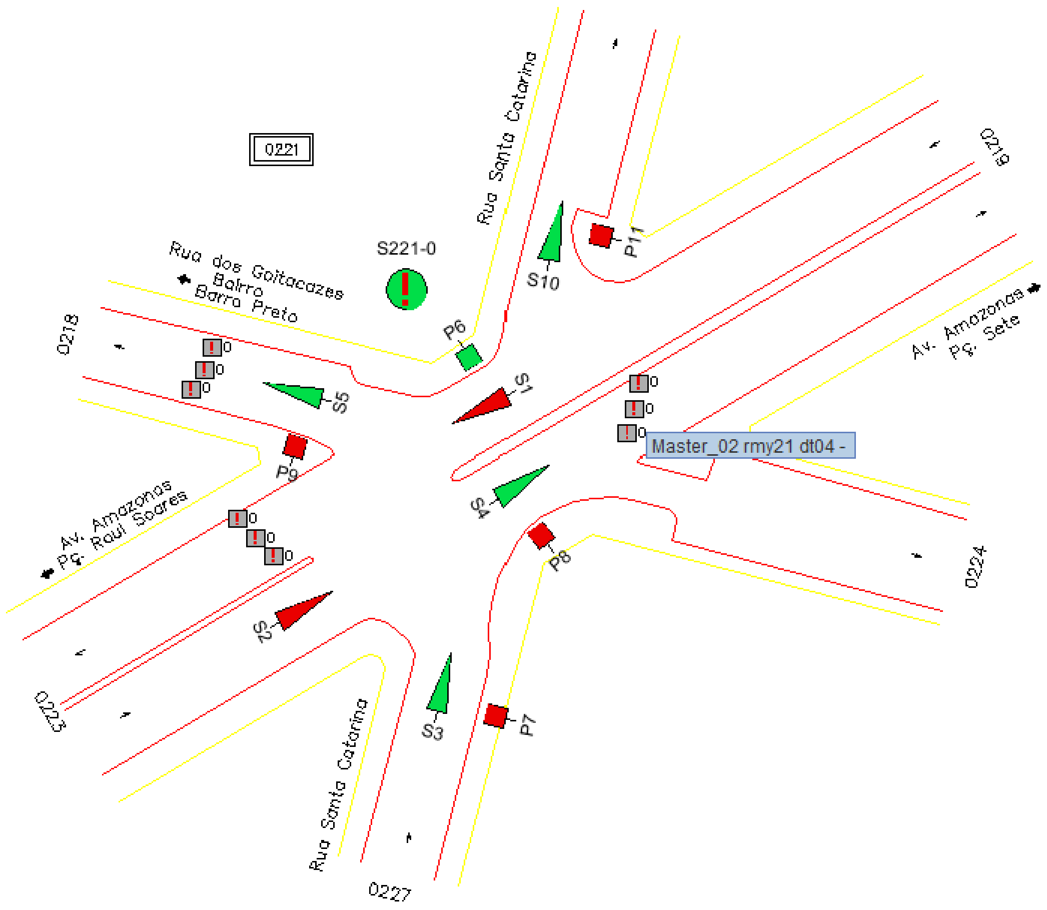

0221
S221-0
Rua Santa Catarina
Rua dos Goitacazes
Bairro
Barra Preto
0218
0219
Av. Amazonas
Pç. Sete
Av. Amazonas
Pç. Raul Soares
0224
0223
Rua Santa Catarina
0227
S1
S2
S3
S4
S5
S10
P6
P7
P8
P9
P11
Master_02 rmy21 dt04 -

S1:7-9, S5:1-3

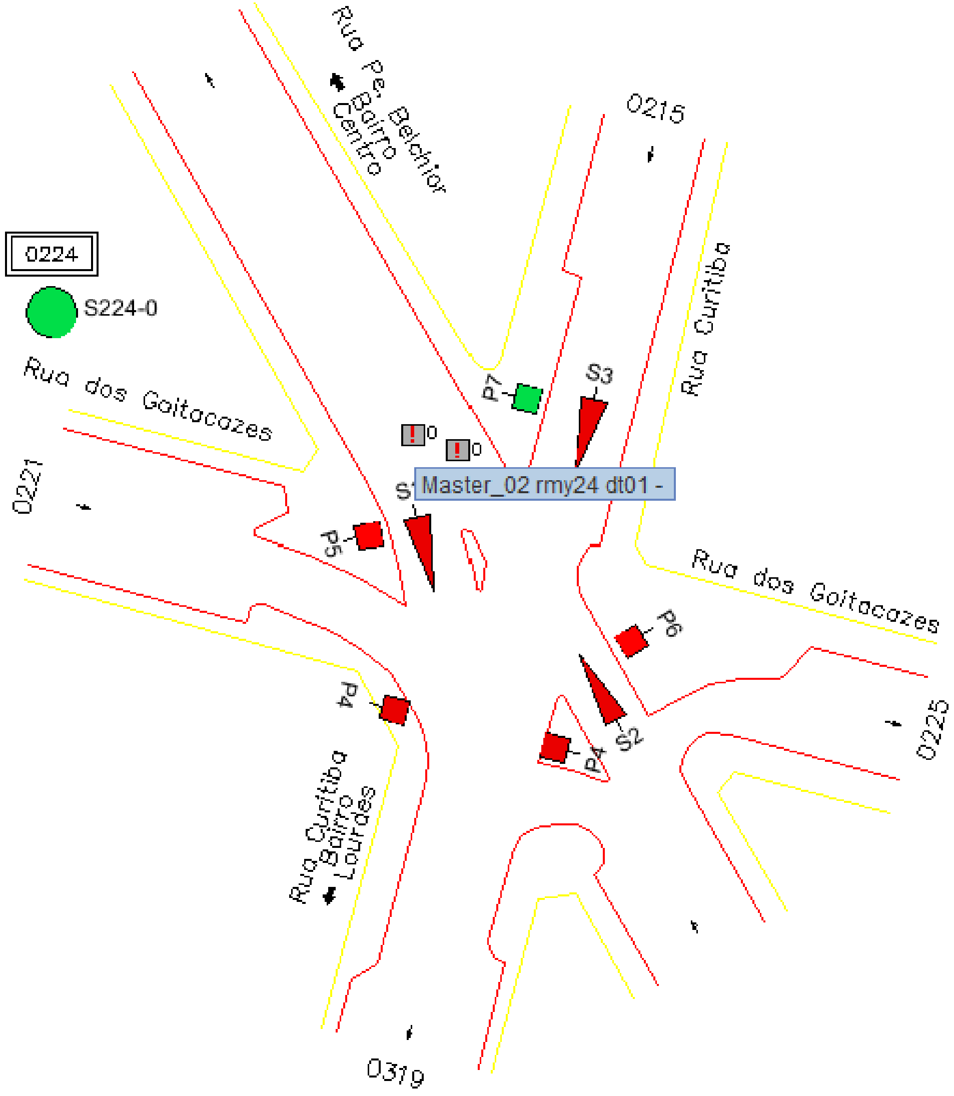
0224
S224-0
Rua dos Goitacazes
Rua Pe. Belchior
Bairro
Centro
0215
Rua Curitiba
S3
P7
Master_02 rmy24 dt01 -
0221
S1
P5
Rua dos Goitacazes
P6
P4
S2
P4
0225
Rua Curitiba
Bairro
Lourdes
0319

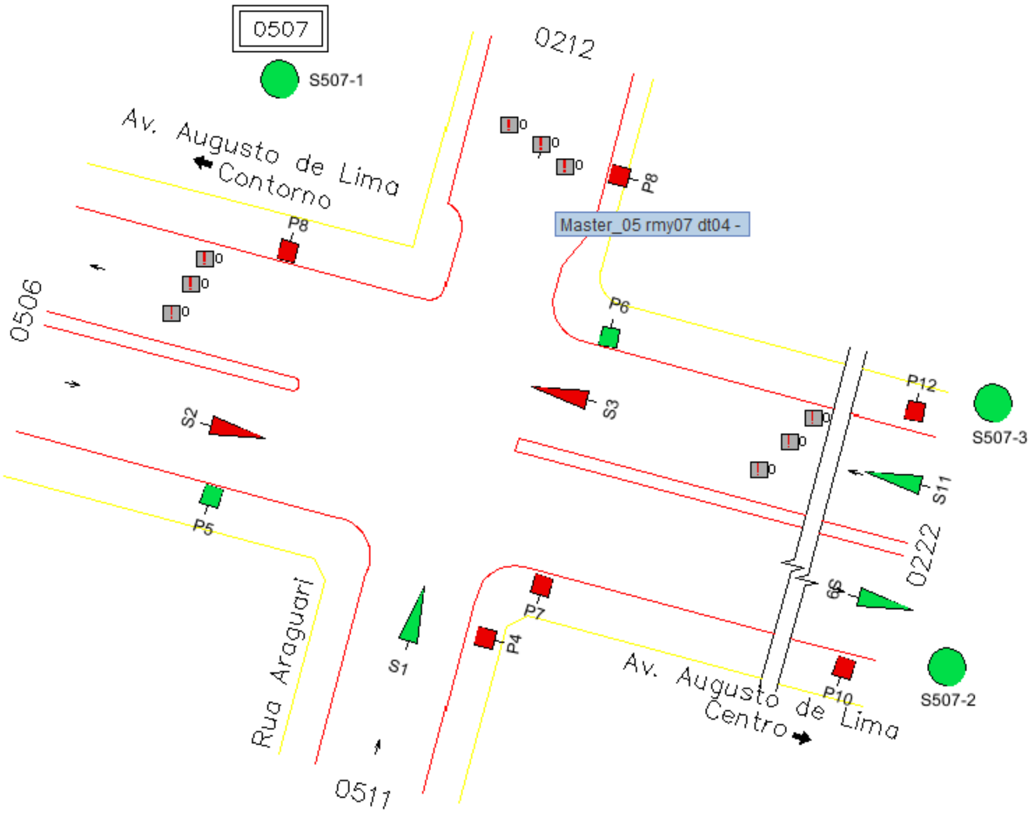
0507
S507-1
0212
Av. Augusto de Lima
Contorno
P8
Master_05 rmy07 dt04 -
0506
P6
P12
S507-3
S2
S3
S11
P5
0222
S9
P7
Rua Araguari
S1
P4
Av. Augusto de Lima
Centro
P10
S507-2
0511

S11:7-9, S3:1-3

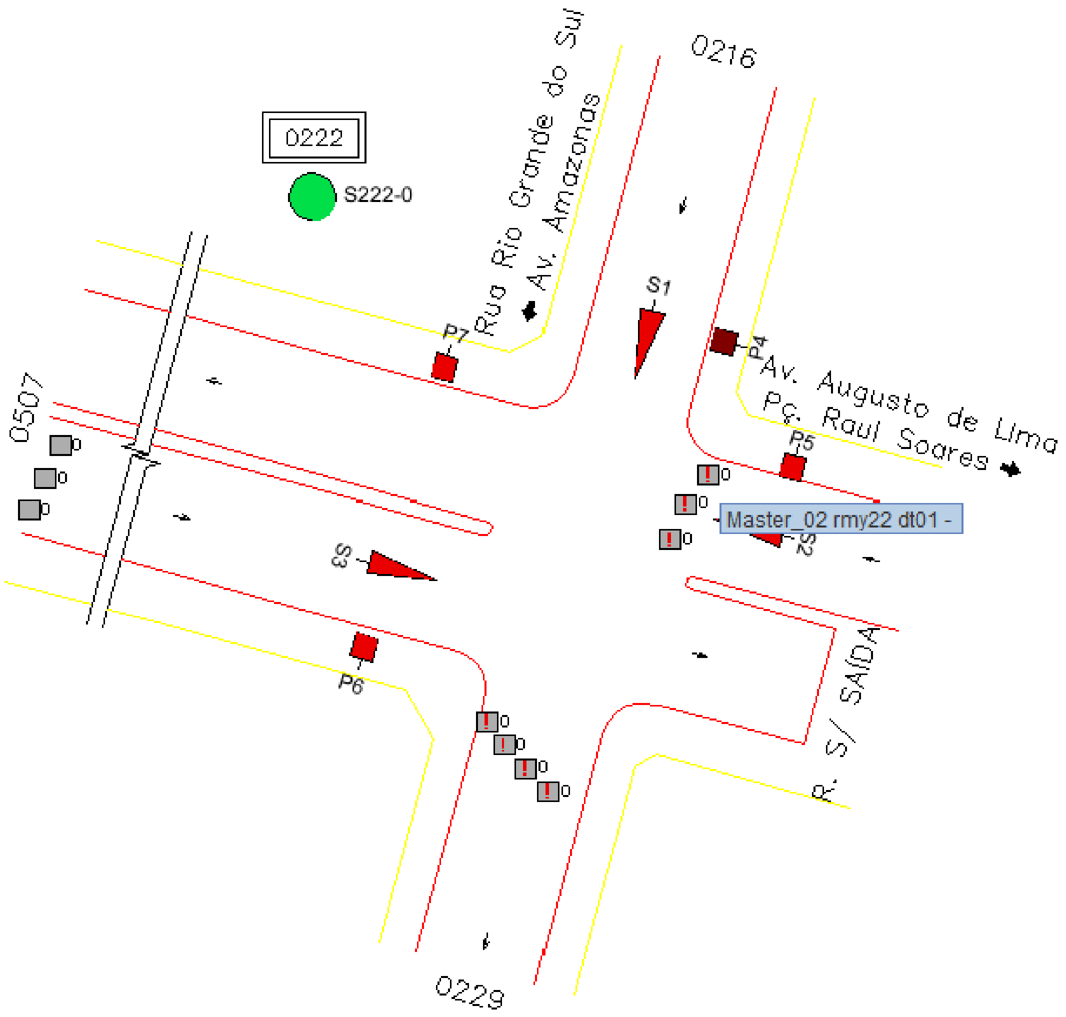

0222
S222-0
Rua Rio Grande do Sul
Av. Amazonas
0216
S1
P4
Av. Augusto de Lima
Pç. Raul Soares
P5
P7
0507
S3
Master_02 rmy22 dt01 -
S2
P6
R. S/ SAÍDA
0229

S1:4-7, S3:8-10

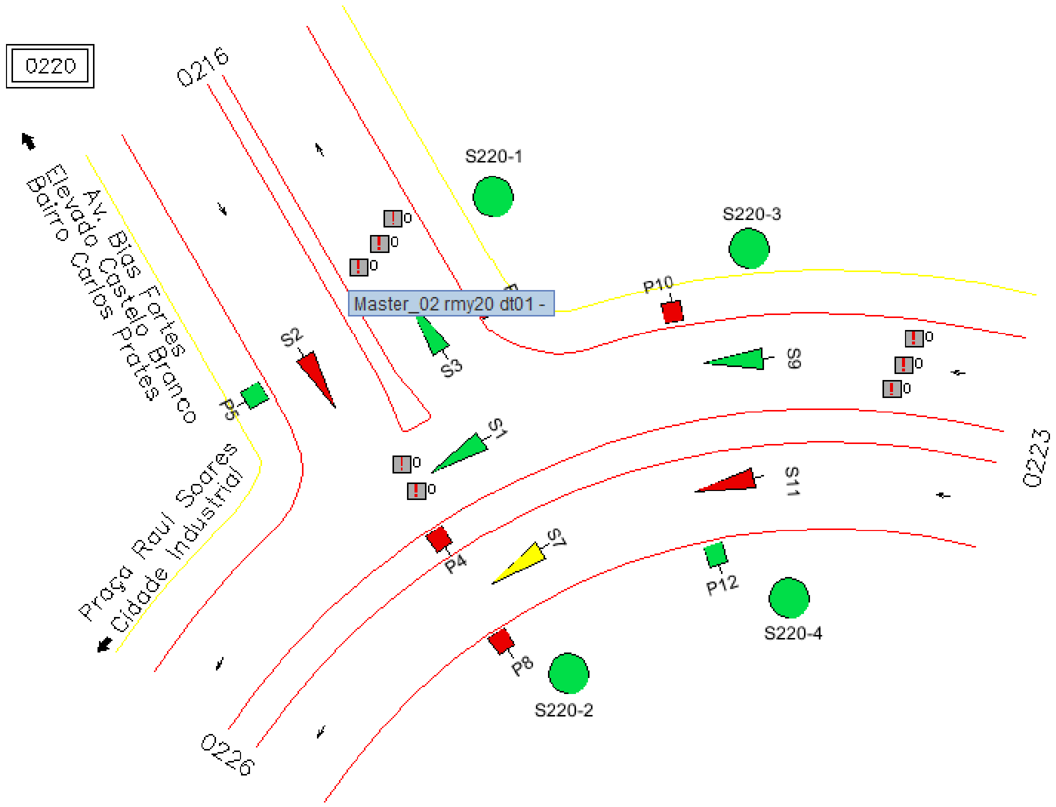
0220
0216
S220-1
S220-3
Av. Bias Fortes
Elevado Castelo Branco
Bairro Carlos Prates
Master_02 rmy20 dt01 -
P10
S2
S3
S9
P5
S1
S11
0223
Praça Raul Soares
Cidade Industrial
P4
S7
P12
S220-4
P8
S220-2
0226

S9:4-6, S1:7-8

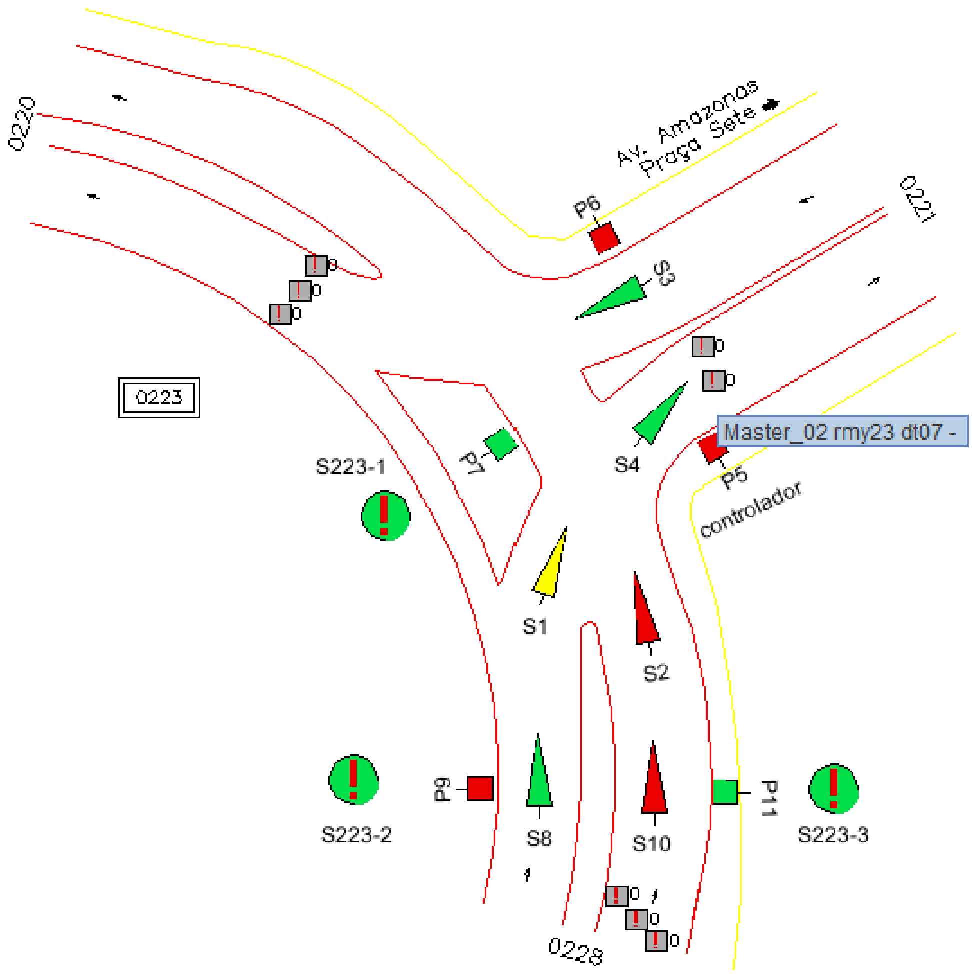
0220
Av. Amazonas
Praça Sete
P6
0221
S3
0223
S4
Master_02 rmy23 dt07 -
P7
P5
S223-1
controlador
S1
S2
S223-2
P9
S8
S10
P11
S223-3
0228

S10:1-3, S3:4-6

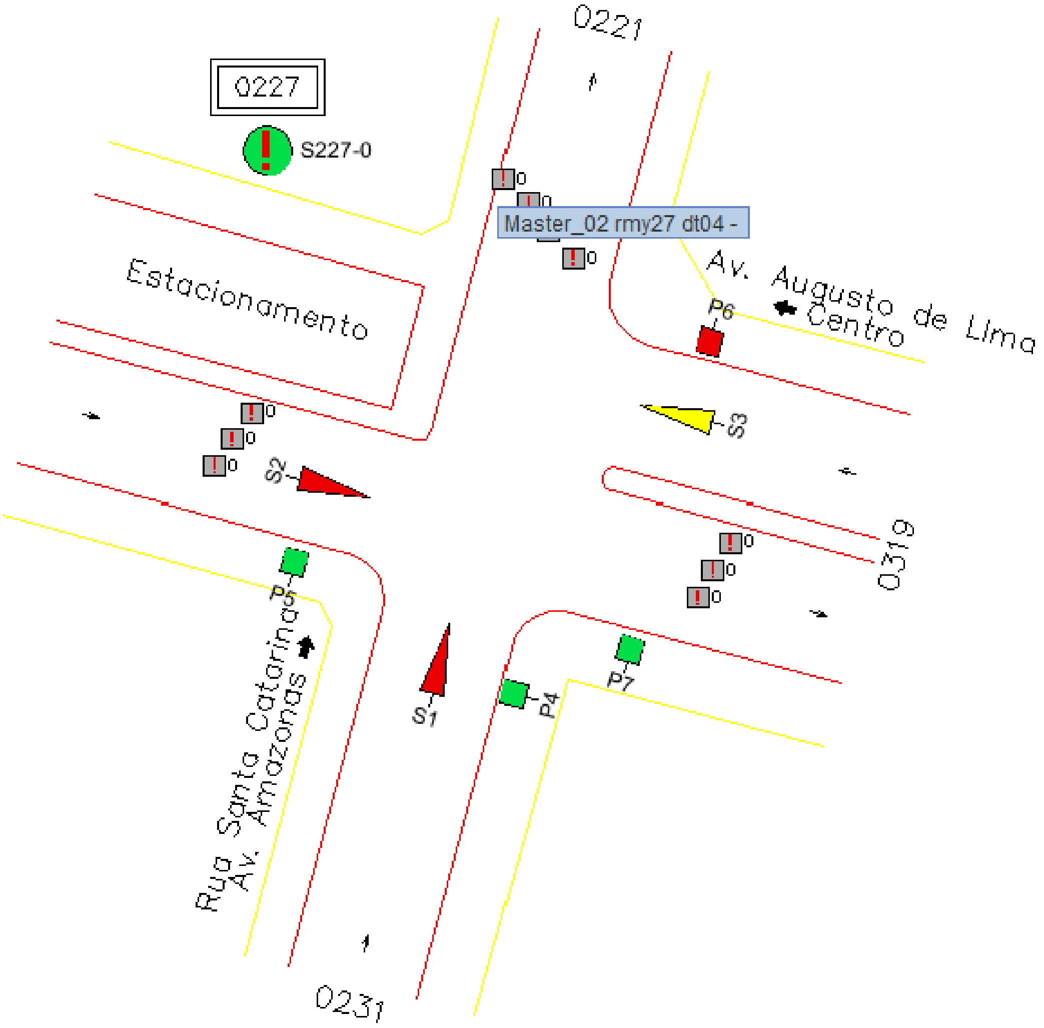
0221
0227
S227-0
Master_02 rmy27 dt04 -
Estacionamento
Av. Augusto de Lima
Centro
P6
S3
S2
0319
P5
P7
P4
S1
Rua Santa Catarina
Av. Amazonas
0231

S2:8-10, S2:1-3,

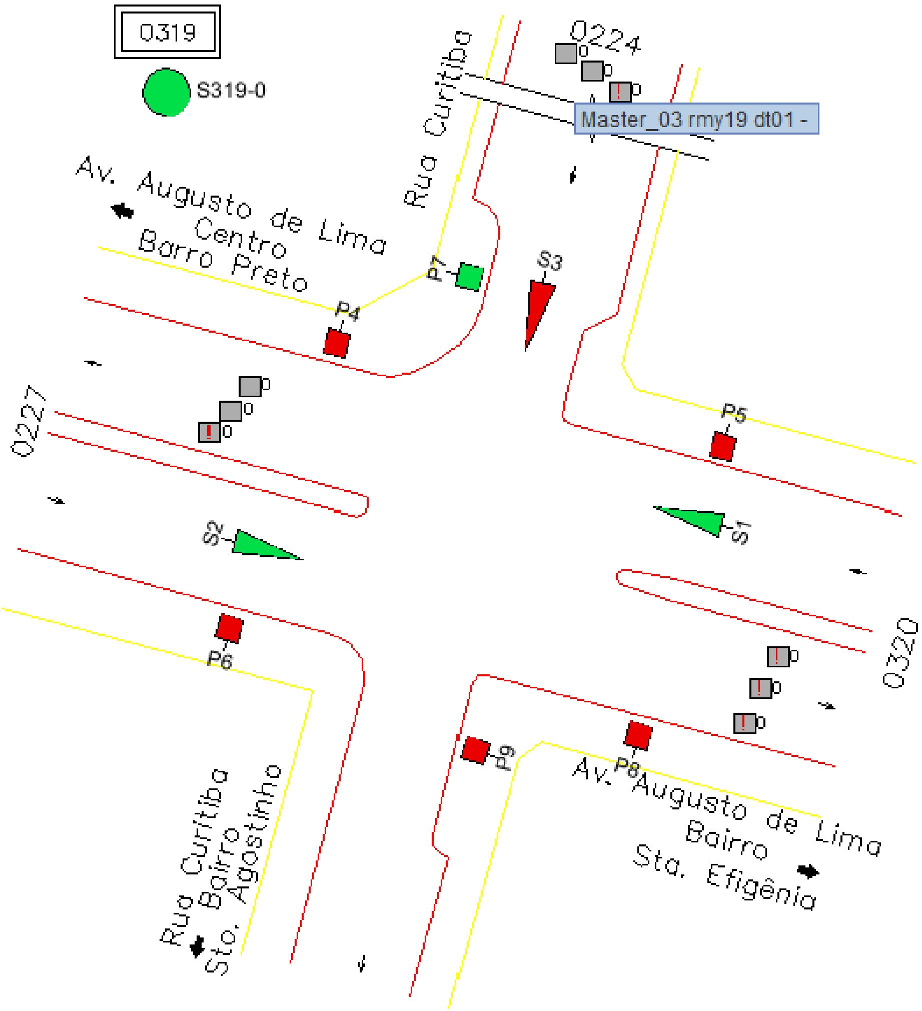
0319
S319-0
0224
Master_03 rmy19 dt01 -
Rua Curitiba
Av. Augusto de Lima
Centro
Barro Preto
P7
P4
S3
0227
P5
S2
S1
P6
0320
P9
P8
Av. Augusto de Lima
Bairro
Sta. Efigênia
Rua Curitiba
Bairro
Sto. Agostinho

S2:4-6, S1:7-9,

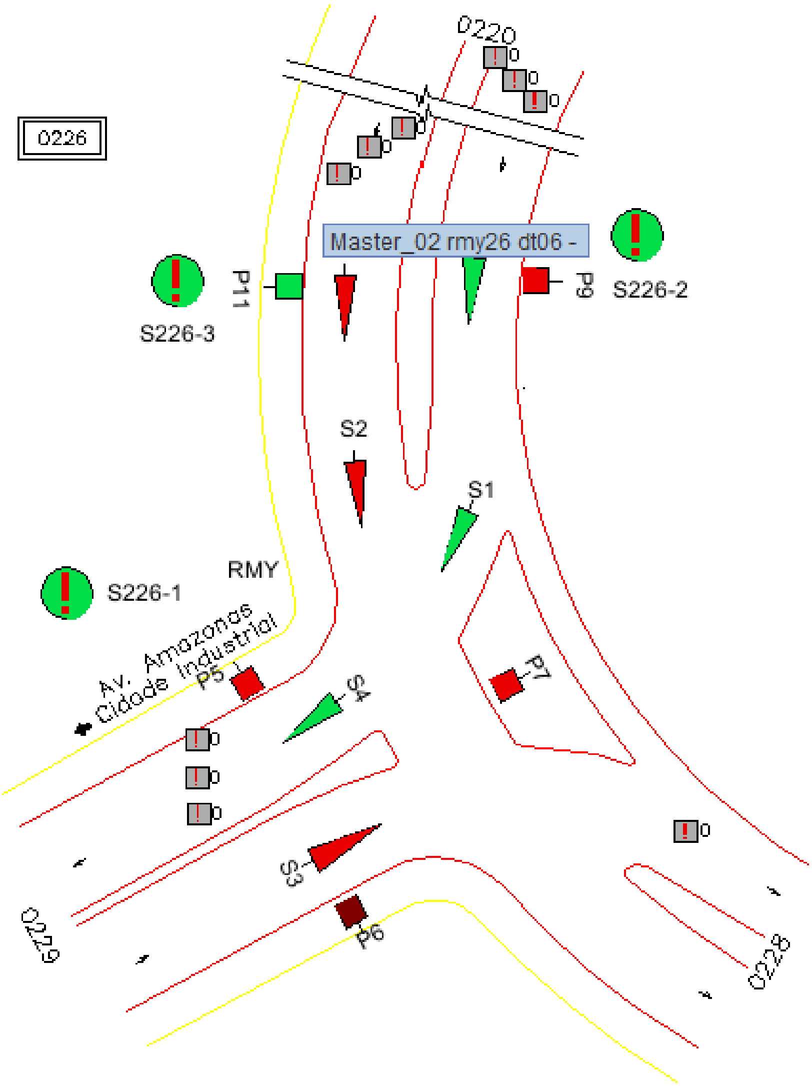

S8:1-3, S3:7, S4:8-10,

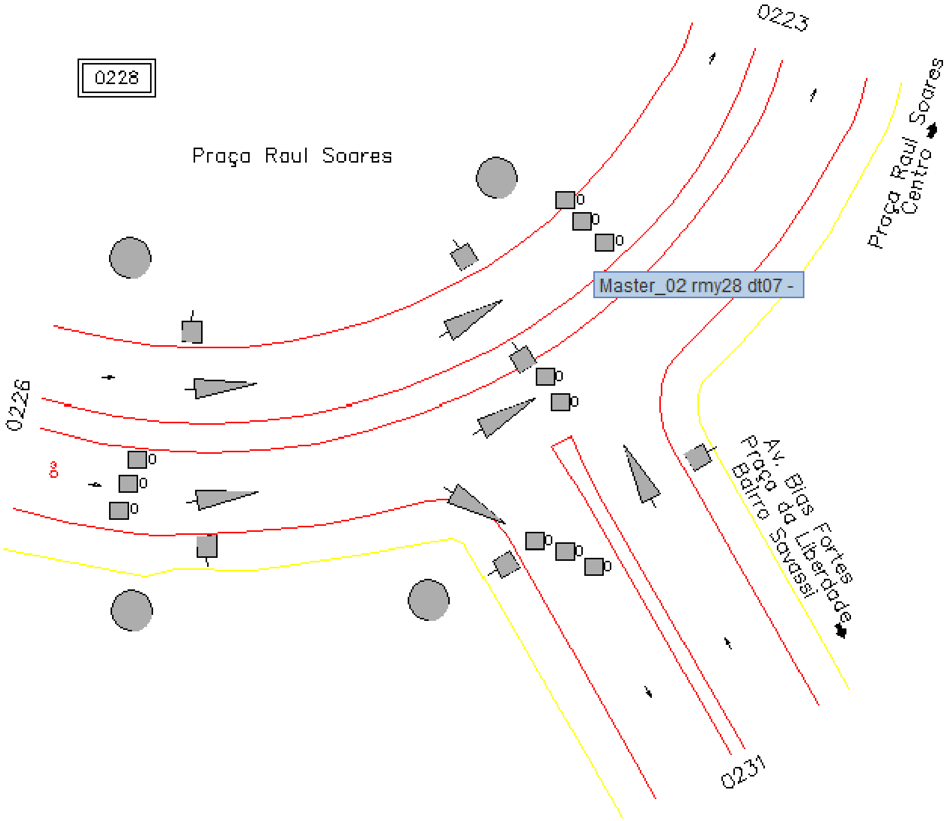

S2:10-11, S3:1-3, S9:4-6

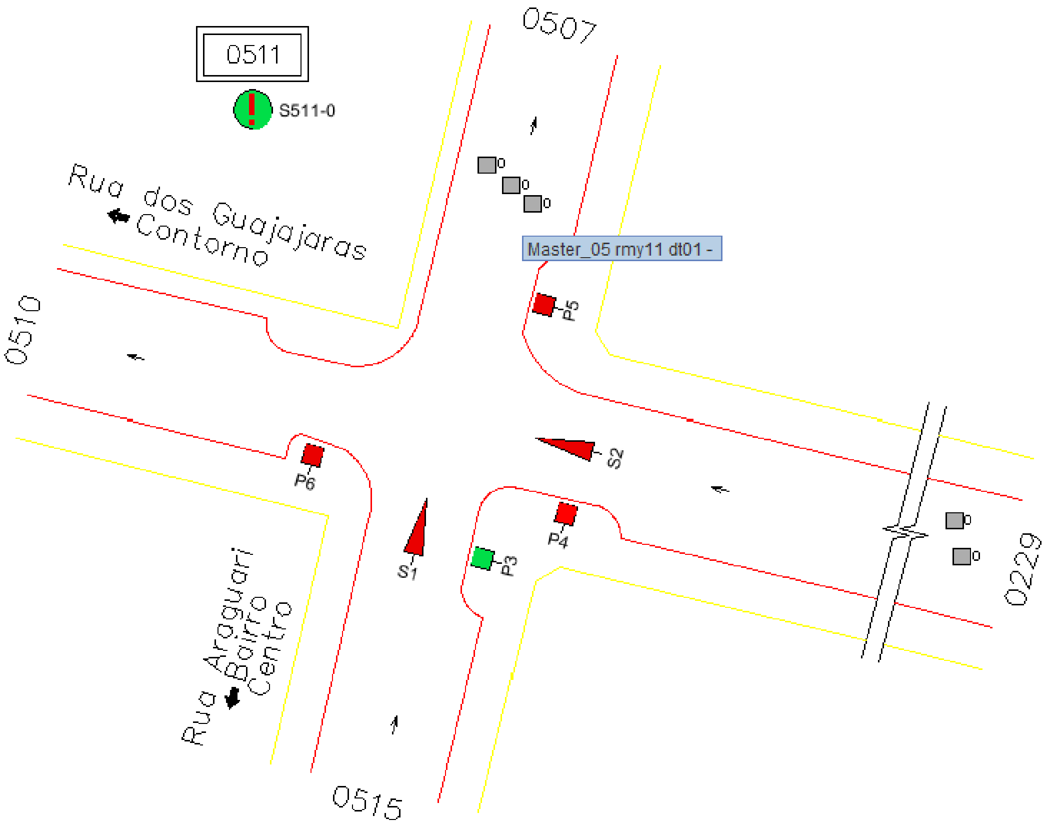

0511
S511-0
0507
Rua dos Guajajaras
Contorno
Master_05 rmy11 dt01 -
P5
0510
P6
S2
S1
P3
P4
0229
Rua Araguari
Bairro
Centro
0515

S2:4-5,

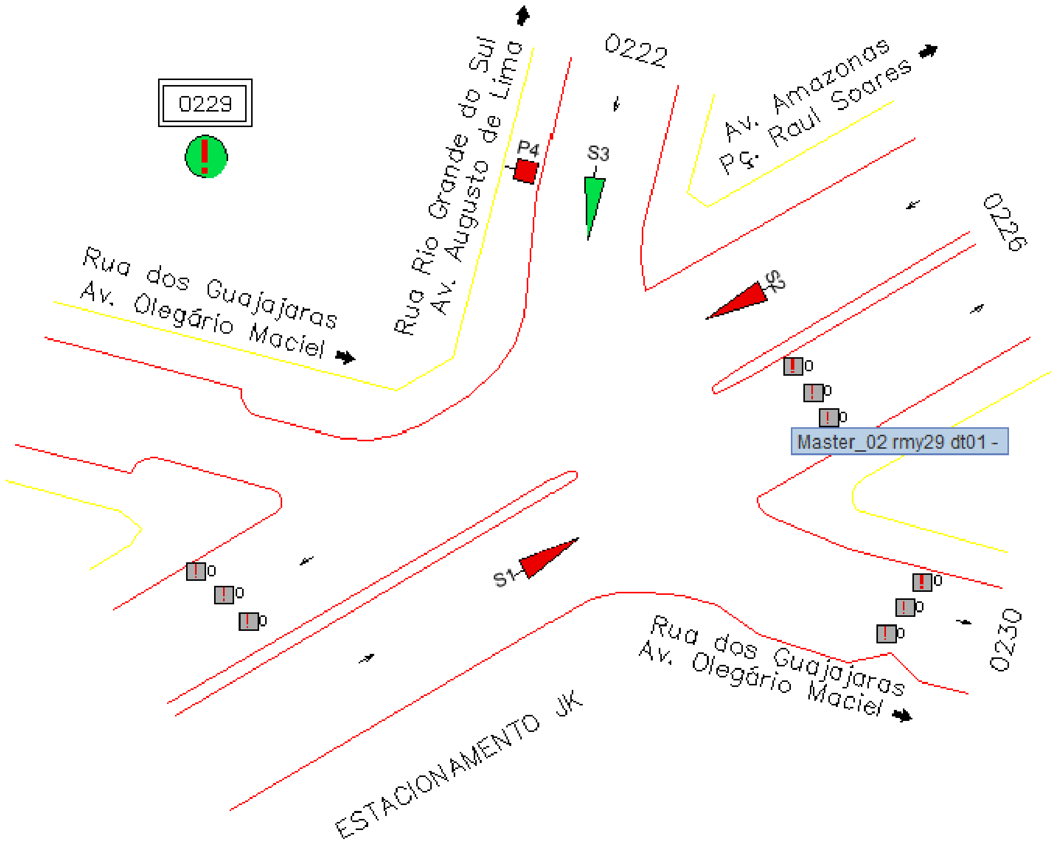
0229
0222
Rua Rio Grande do Sul
Av. Augusto de Lima
P4
S3
Av. Amazonas
Pç. Raul Soares
0226
Rua dos Guajajaras
Av. Olegário Maciel
S2
Master_02 rmy29 dt01 -
S1
Rua dos Guajajaras
Av. Olegário Maciel
0230
ESTACIONAMENTO JK

S3:4-6, S2:7-9

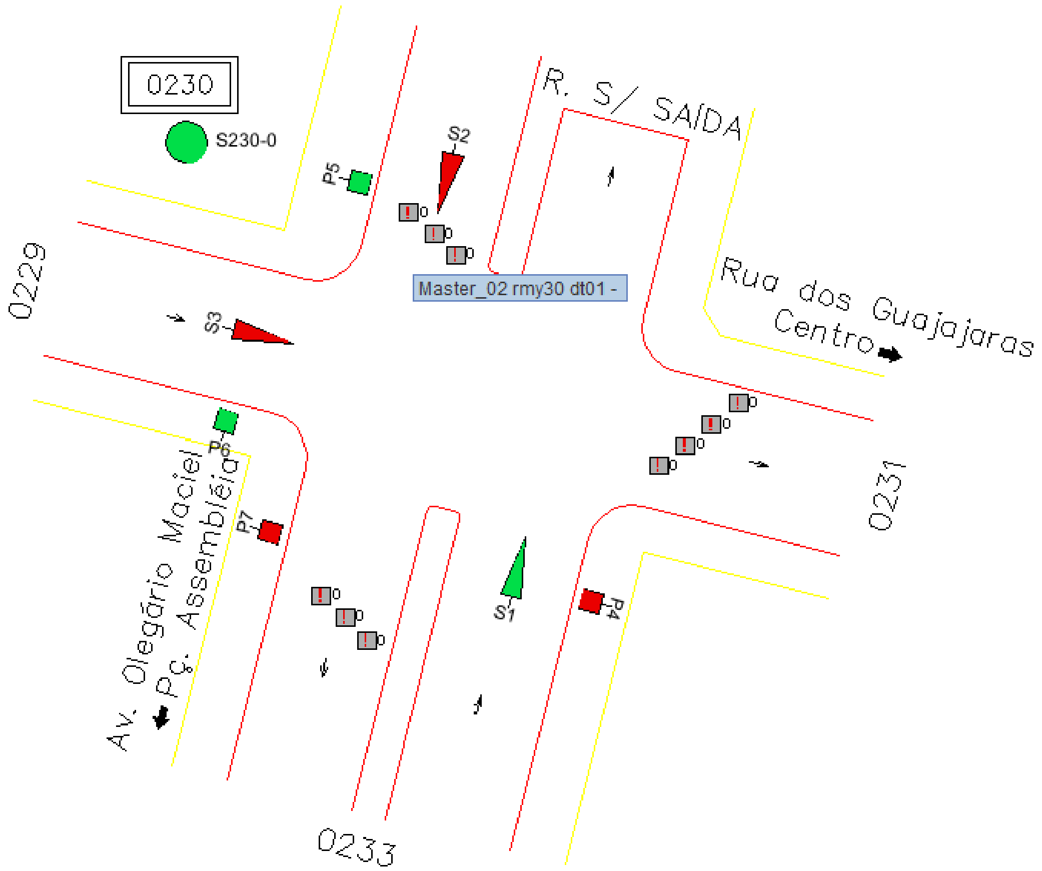
0230
S230-0
R. S/ SAÍDA
S2
P5
Master_02 rmy30 dt01 -
0229
S3
Rua dos Guajajaras
Centro
P6
P7
Av. Olegário Maciel
Pç. Assembléia
S1
P4
0231
0233

S3:4-7, S2:8-10

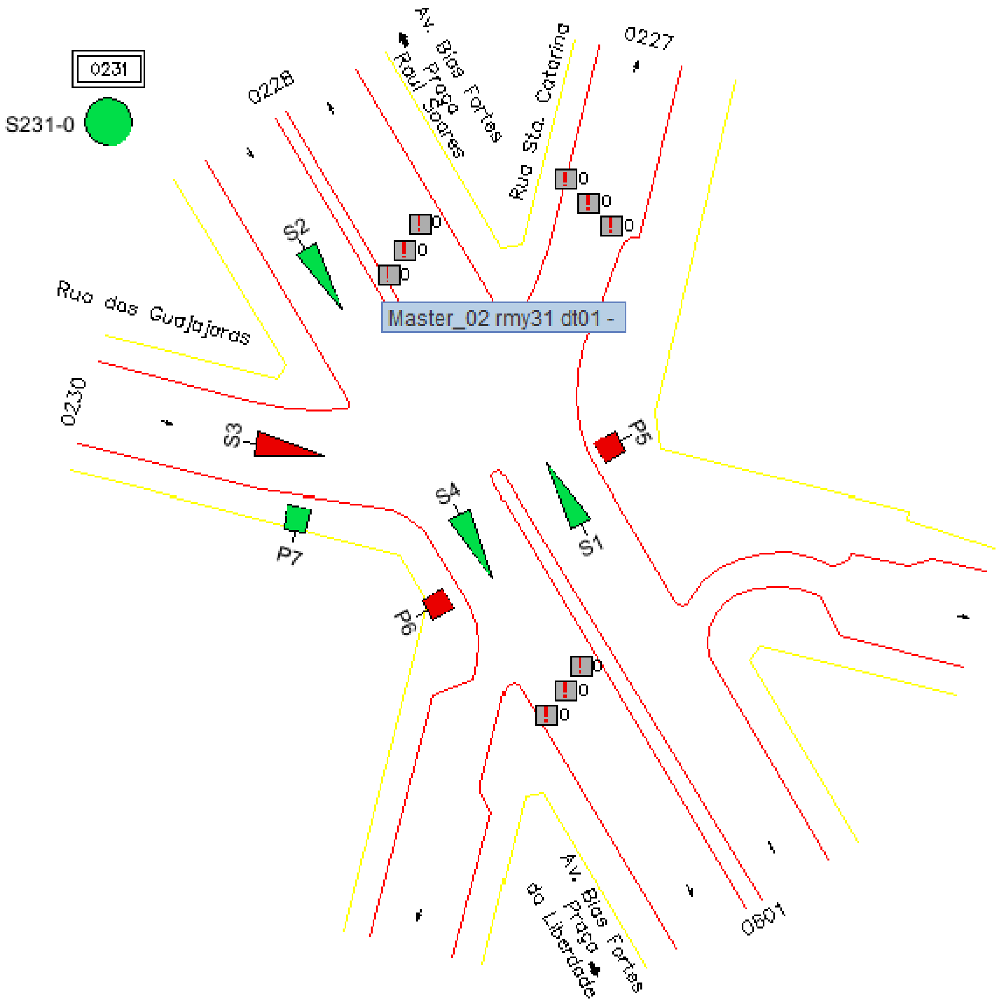

S3:4-6, S4:7-9, S1:1-3

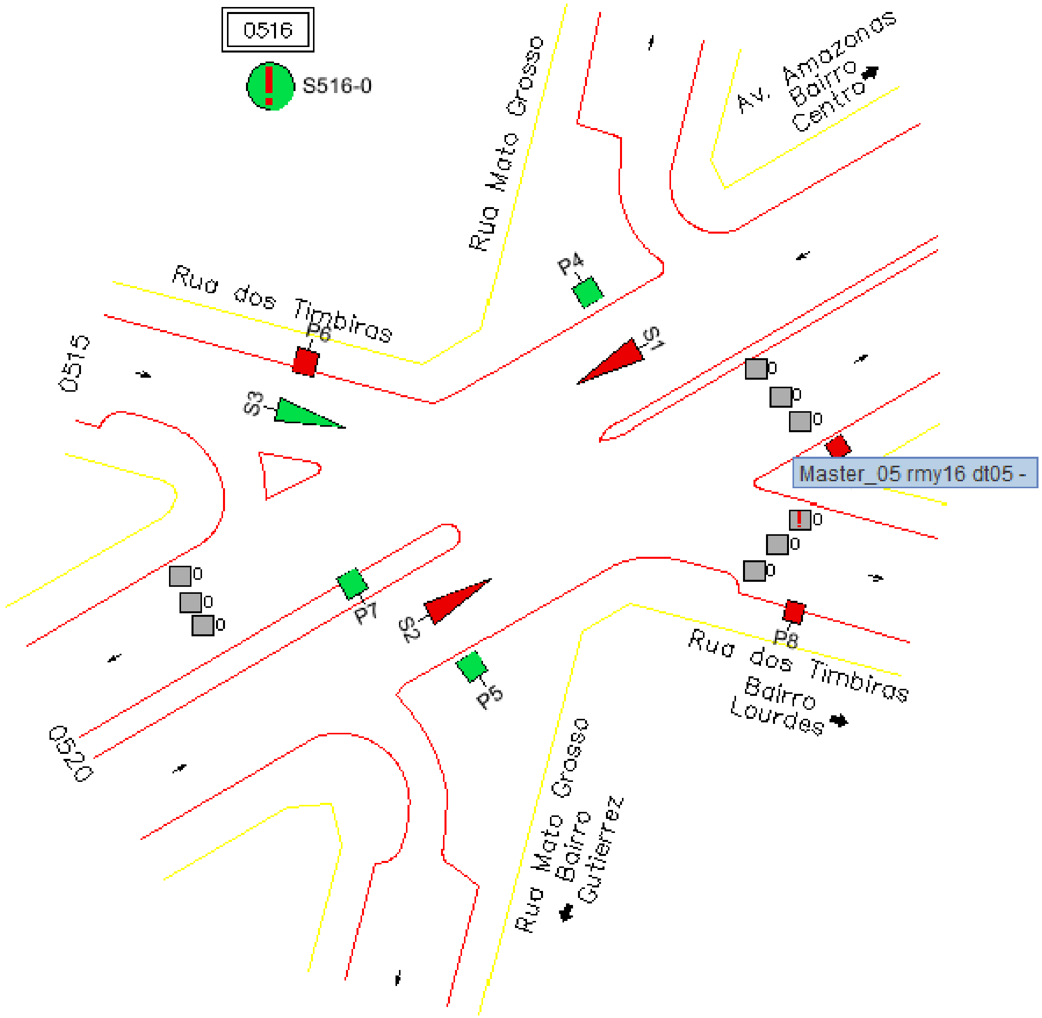
0516
S516-0
Rua Mato Grosso
Av. Amazonas Bairro Centro
Rua dos Timbiras
0515
P4
P6
S1
S3
Master_05 rmy16 dt05 -
P7
S2
P5
P8
Rua dos Timbiras Bairro Lourdes
0520
Rua Mato Grosso Bairro Gutierrez

S3:7-9, S1:1-3

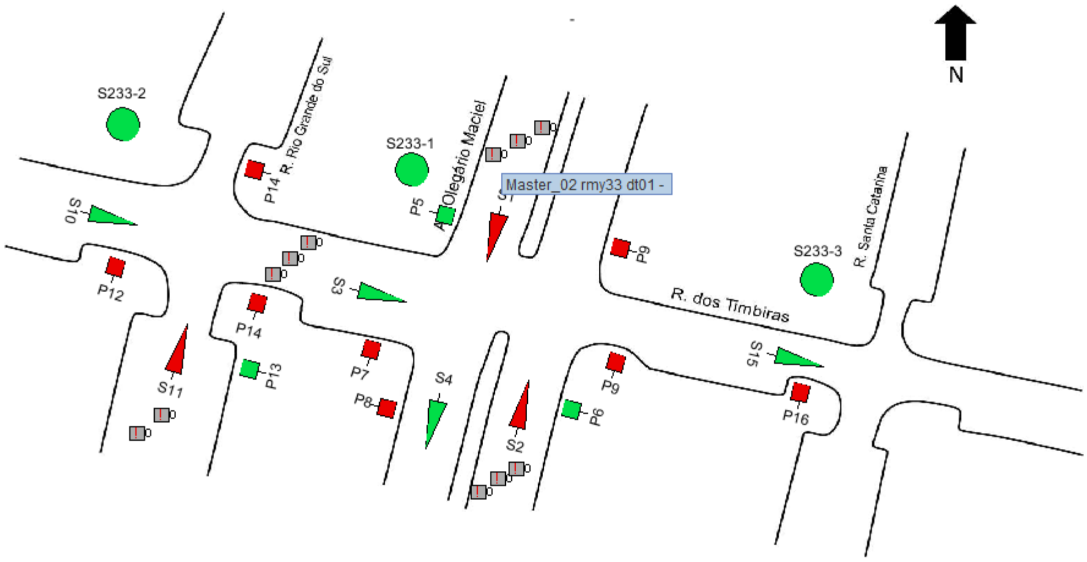

S2:4-6, S11:7-8, S3:9-11,

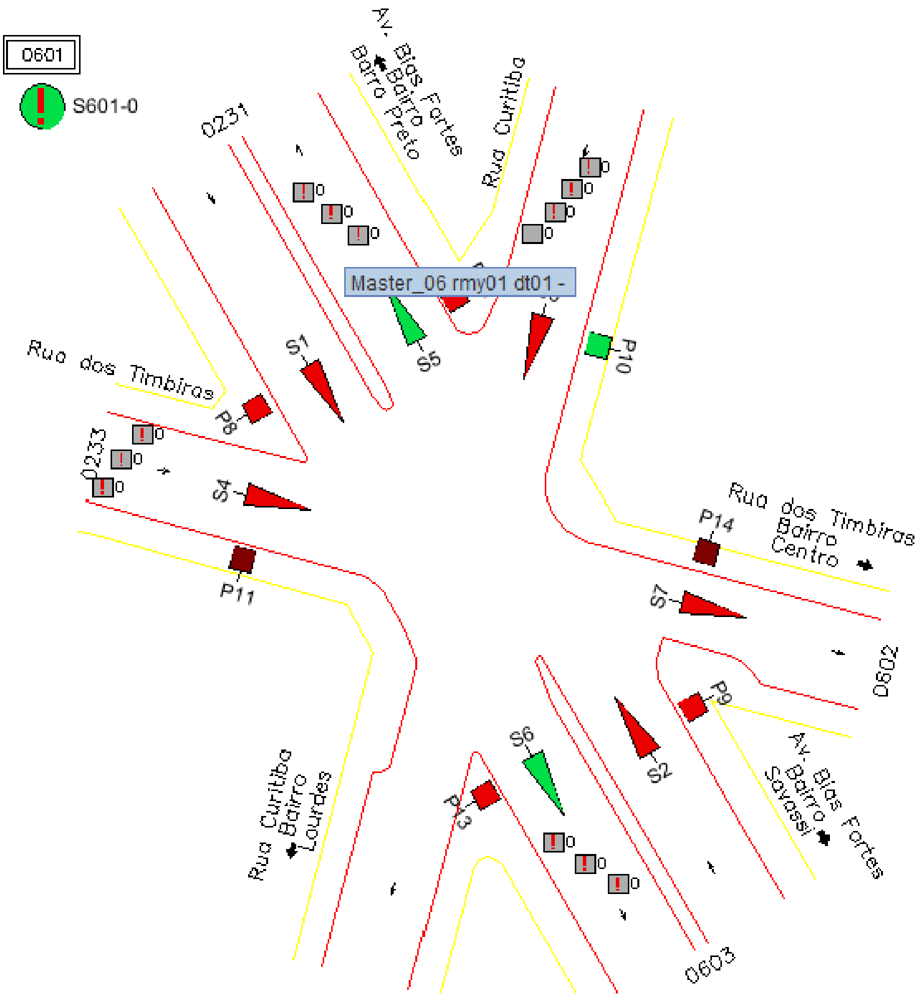

S3:10-13, S6:4-6, S4:7-9

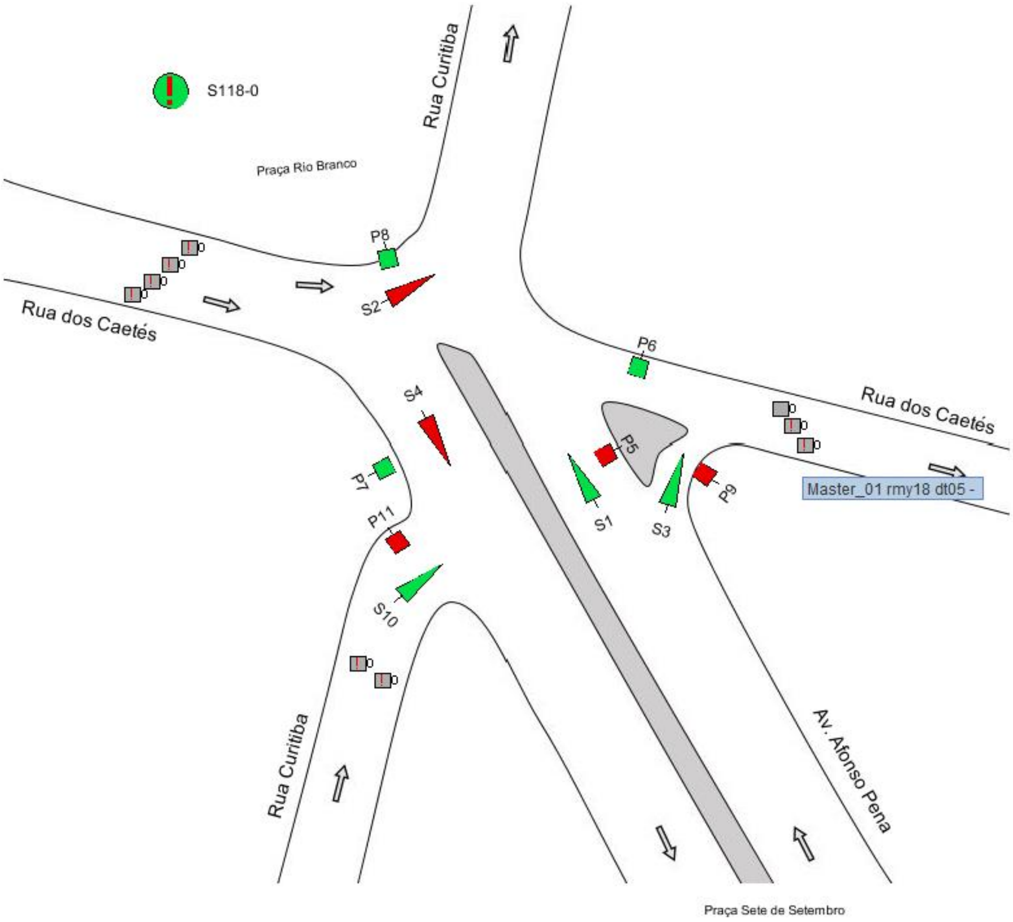
S118-0
Rua Curitiba
Praça Rio Branco
P8
S2
Rua dos Caetés
P6
S4
P5
Rua dos Caetés
P7
P9
Master_01 rmy18 dt05 -
S1
S3
P11
S10
Av. Afonso Pena
Rua Curitiba
Praça Sete de Setembro

S10:8-9, S2:1-4,

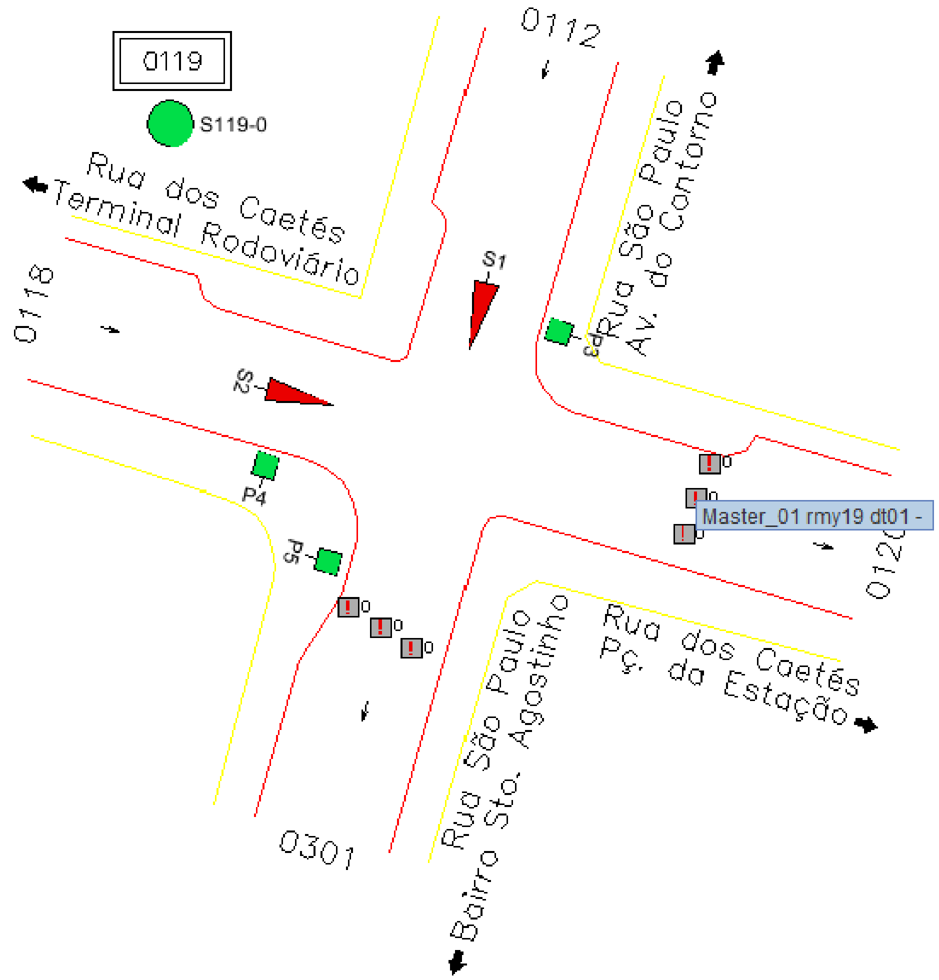
0119
S119-0
Rua dos Caetés
Terminal Rodoviário
0112
Rua São Paulo
Av. do Contorno
S1
S2
0118
P4
P5
Master_01 rmy19 dt01 -
Rua dos Caetés
Pç. da Estação
Rua São Paulo
Sto. Agostinho
Bairro
0301

S1:4-6,

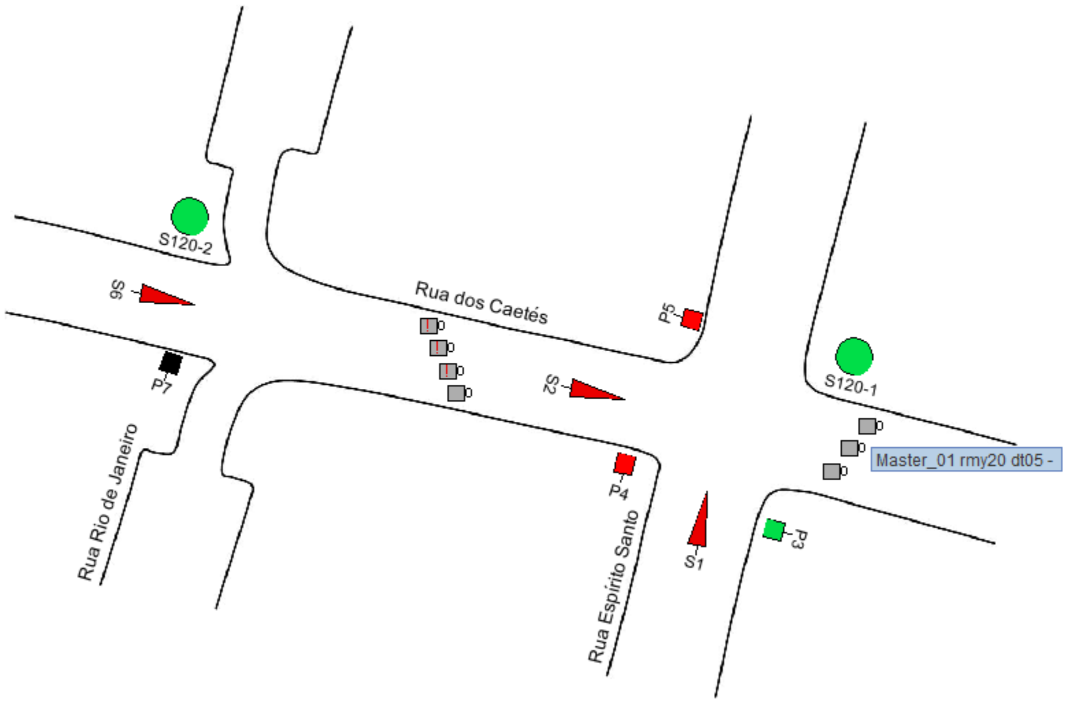
S120-2
S6
Rua dos Caetés
P5
P7
S2
S120-1
Master_01 rmy20 dt05 -
Rua Rio de Janeiro
P4
P3
S1
Rua Espírito Santo

S2:1-4

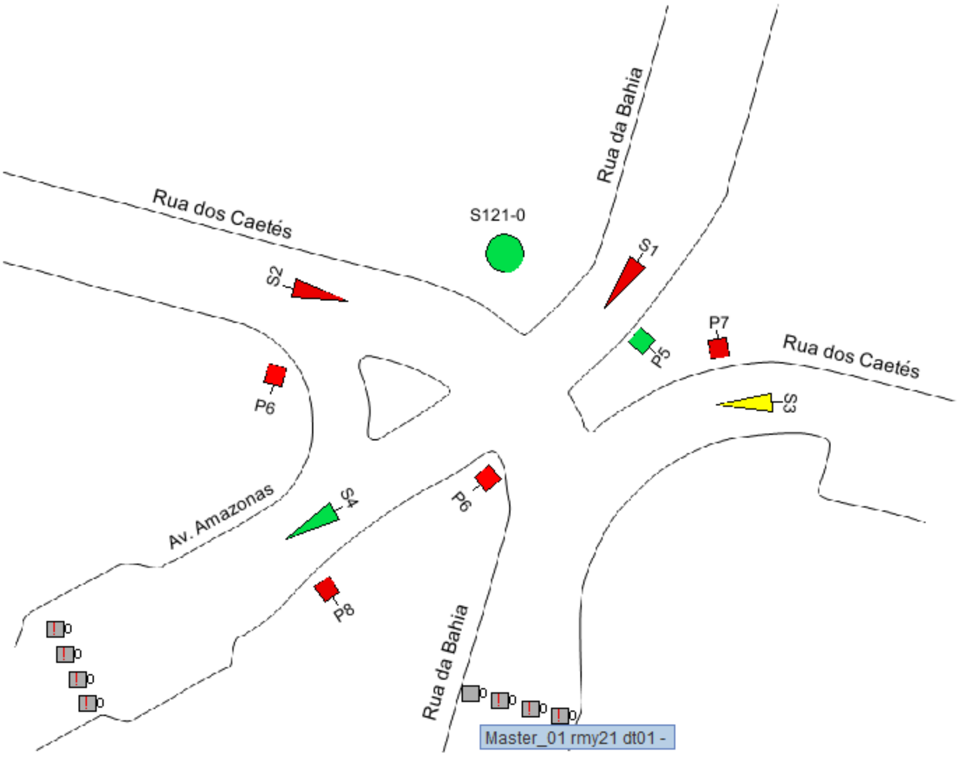
Rua da Bahia
Rua dos Caetés
S121-0
S1
S2
P5
P7
Rua dos Caetés
P6
S3
Av. Amazonas
S4
P6
P8
Rua da Bahia
Master_01 rmy21 dt01 -

S4:5-8

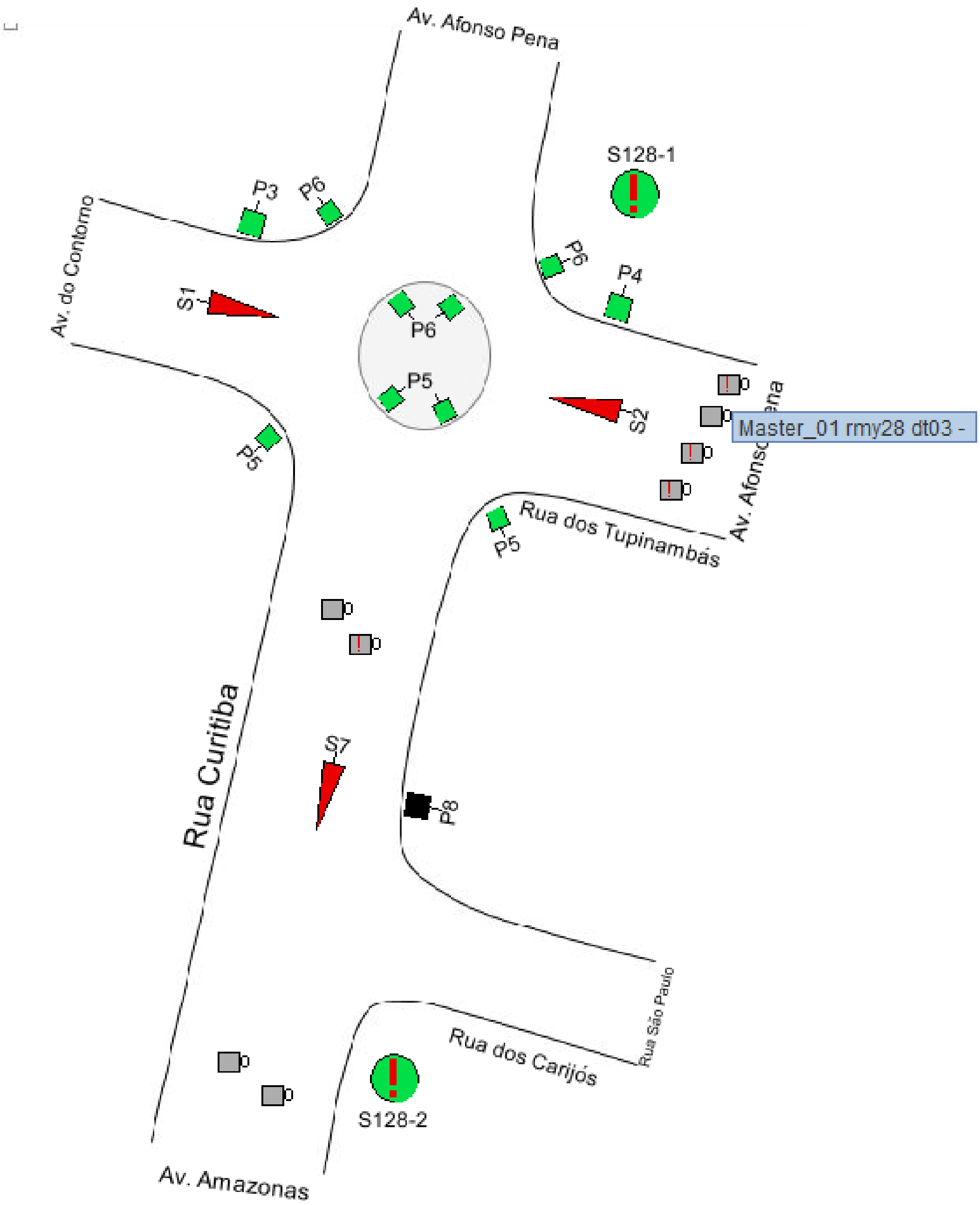

Av. Afonso Pena
S128-1
P3
P6
P6
P4
Av. do Contorno
S1
P6
P5
S2
Master_01 rmy28 dt03 -
P5
P5
Rua dos Tupinambás
Rua Curitiba
S7
P8
Rua São Paulo
Rua dos Carijós
S128-2
Av. Amazonas

S7:7-8, S7-:1-2

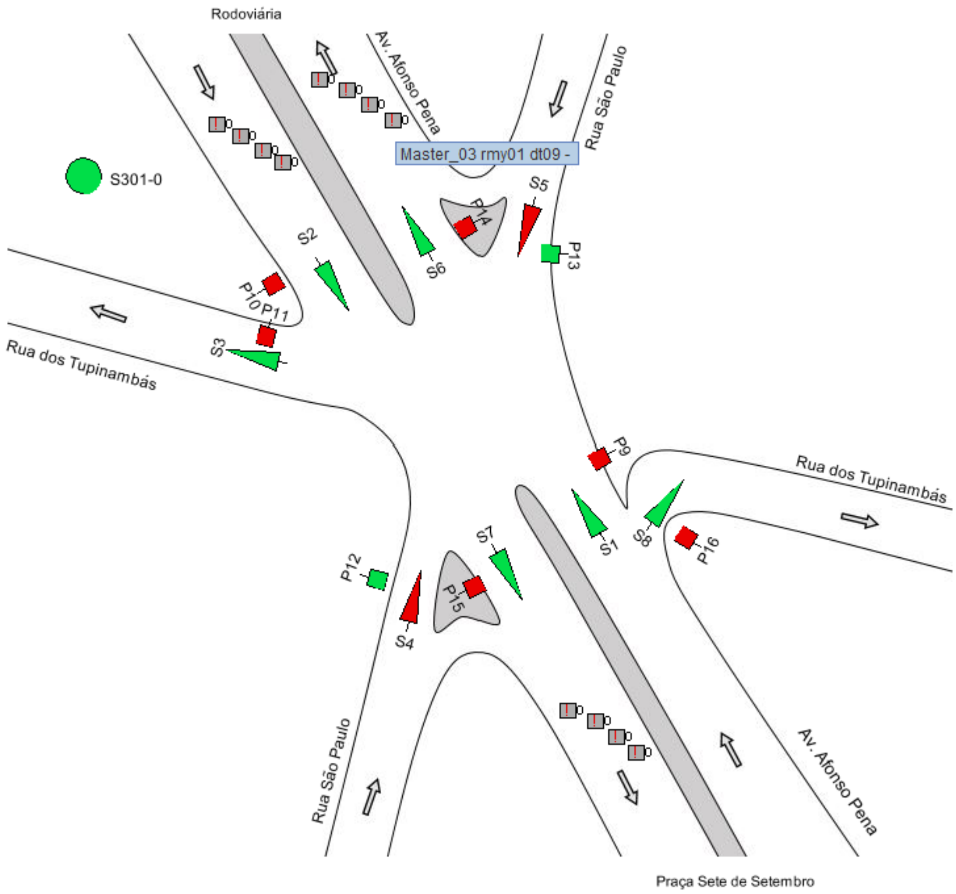
Rodoviária
Av. Afonso Pena
Rua São Paulo
Master_03 rmy01 dt09 -
S301-0
S2
S6
S5
P14
P13
P10
P11
S3
Rua dos Tupinambás
P9
Rua dos Tupinambás
S1
S8
P16
S7
P12
P15
S4
Rua São Paulo
Av. Afonso Pena
Praça Sete de Setembro

S7:1-4, S2:5-8

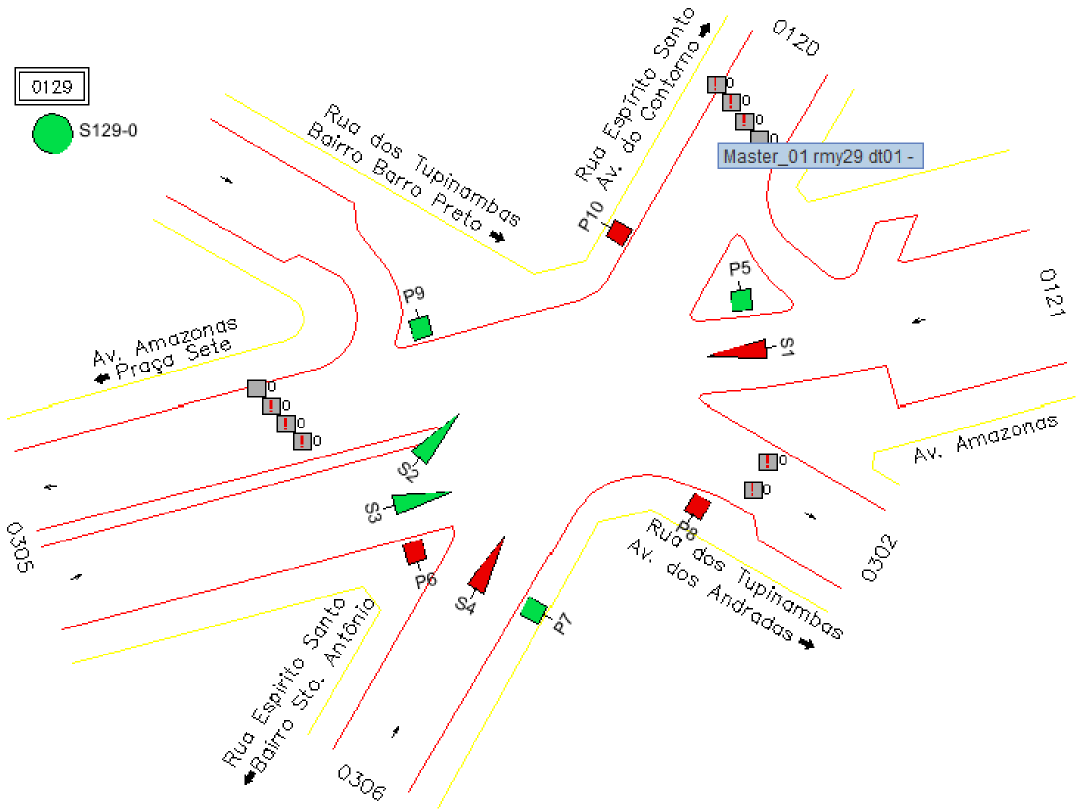
0129
S129-0
Rua dos Tupinambas
Bairro Barro Preto
Rua Espírito Santo
Av. do Contorno
0120
Master_01 rmy29 dt01 -
P10
P9
P5
S1
0121
Av. Amazonas
Praça Sete
S2
S3
0305
Av. Amazonas
P8
Rua dos Tupinambas
Av. dos Andradas
0302
P6
S4
P7
Rua Espírito Santo
Bairro Sto. Antônio
0306

S3:5-6, S1:7-10

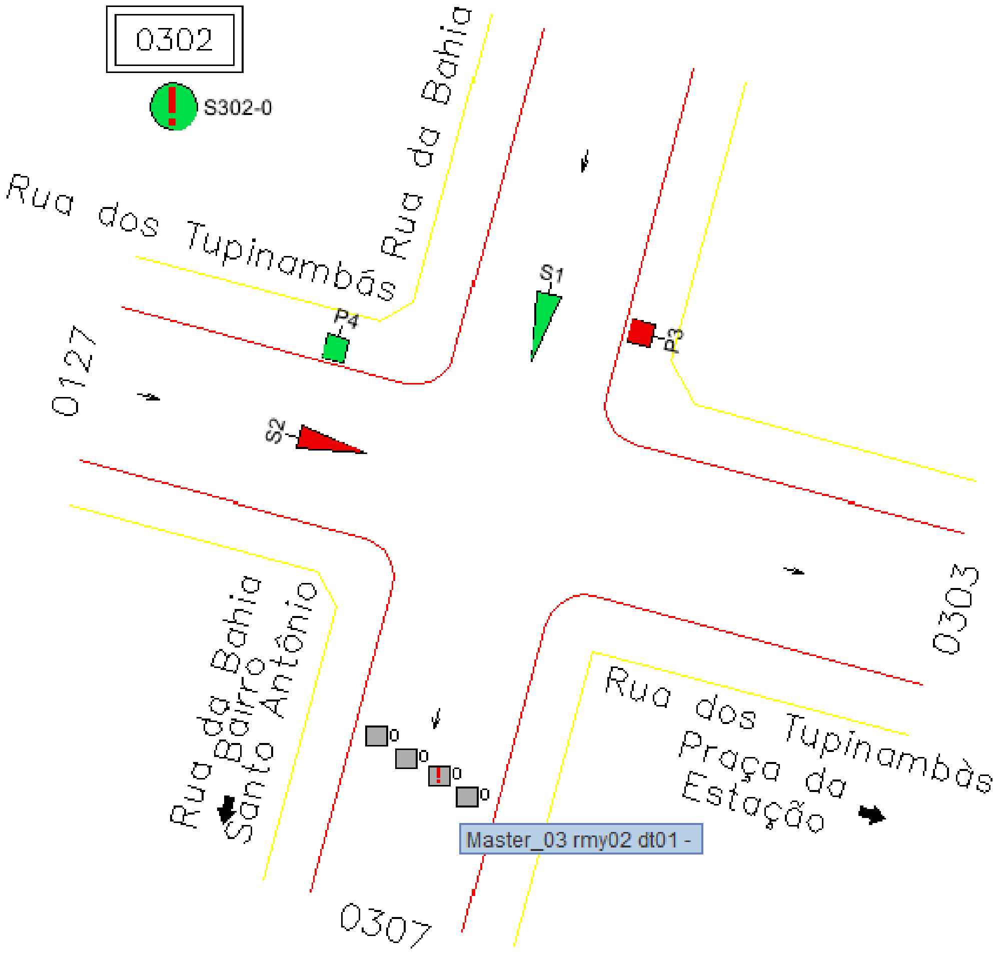
0302
S302-0
Rua dos Tupinambás
Rua da Bahia
S1
P4
P3
0127
S2
0303
Rua da Bahia
Bairro
Santo Antônio
Rua dos Tupinambàs
Praça da
Estação
Master_03 rmy02 dt01 -
0307

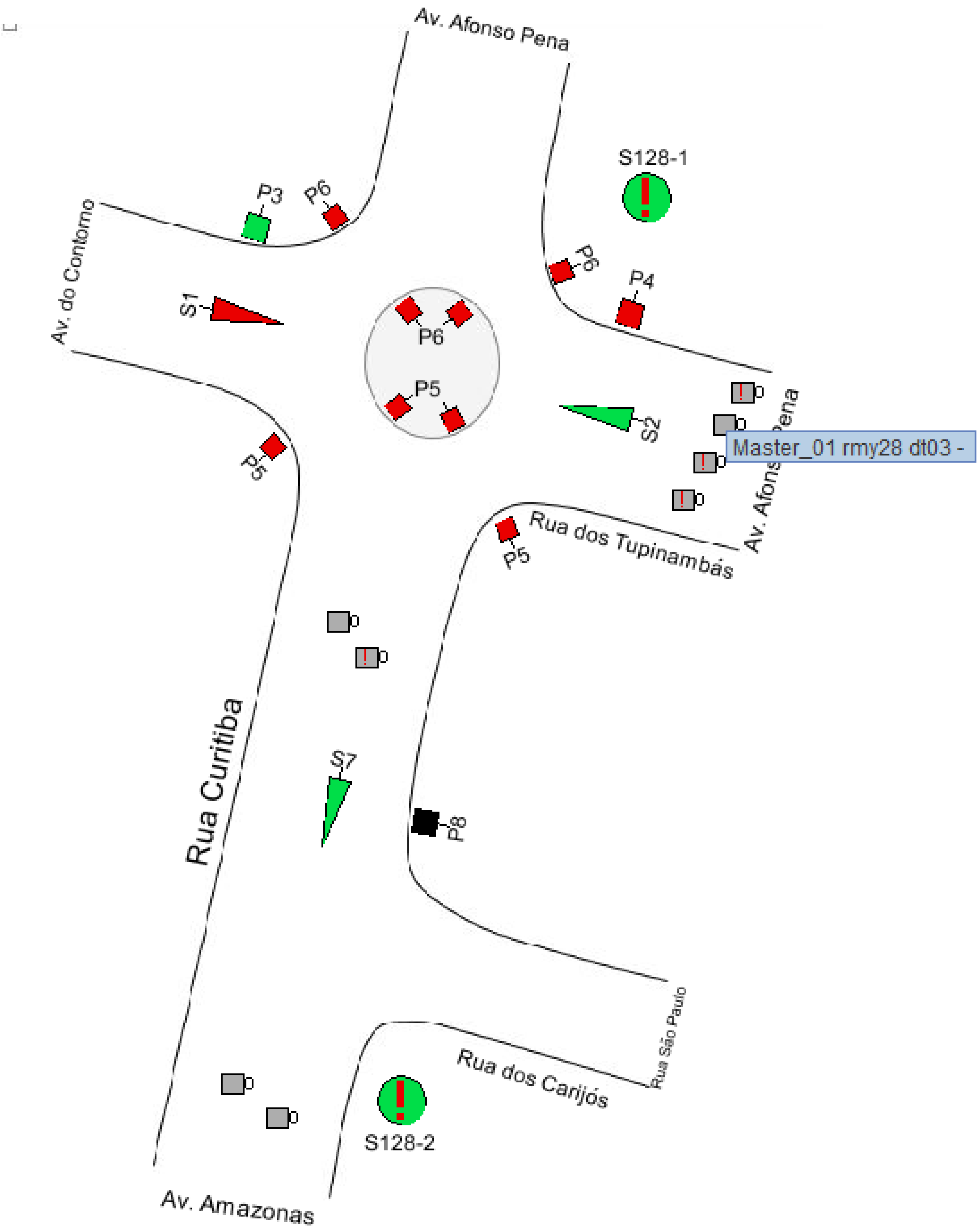

Av. Afonso Pena
S128-1
P3
P6
Av. do Contorno
S1
P6
P4
P6
P5
S2
Master_01 rmy28 dt03 -
Av. Afonso Pena
P5
P5
Rua dos Tupinambás
Rua Curitiba
S7
P8
Rua São Paulo
Rua dos Carijós
S128-2
Av. Amazonas

S7:7-8, S7-:1-2

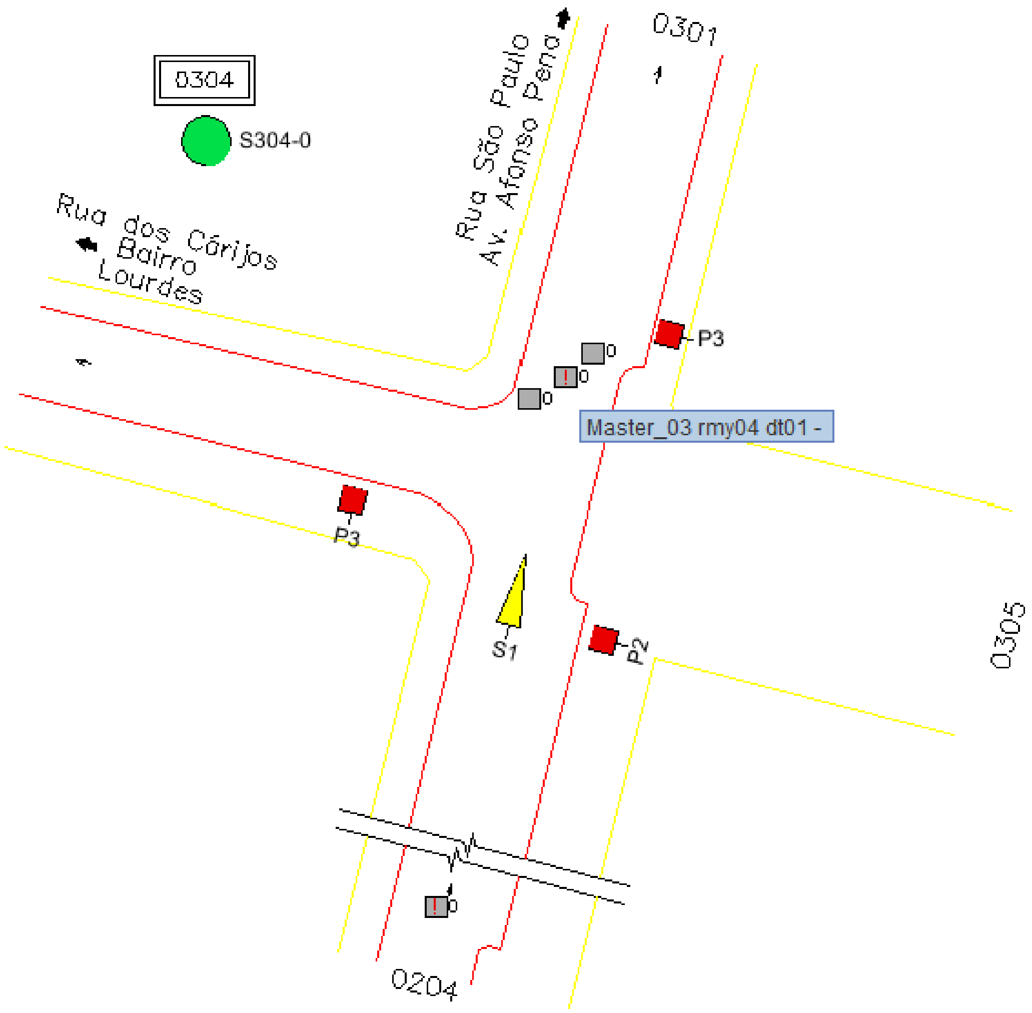
0304
S304-0
Rua dos Córijos
Bairro Lourdes
Rua São Paulo
Av. Afonso Pena
0301
P3
Master_03 rmy04 dt01 -
P3
S1
P2
0305
0204

S1:4

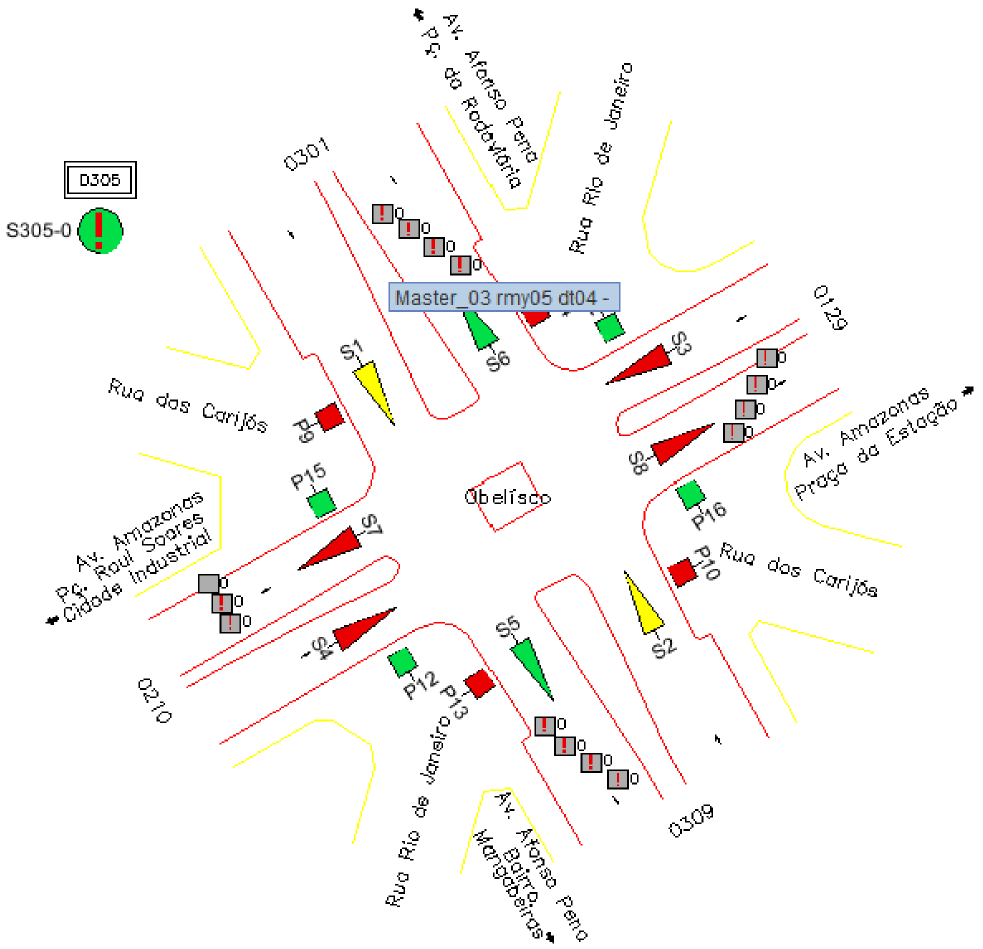

S8:8-11, S5:12-15, S7:1-3,

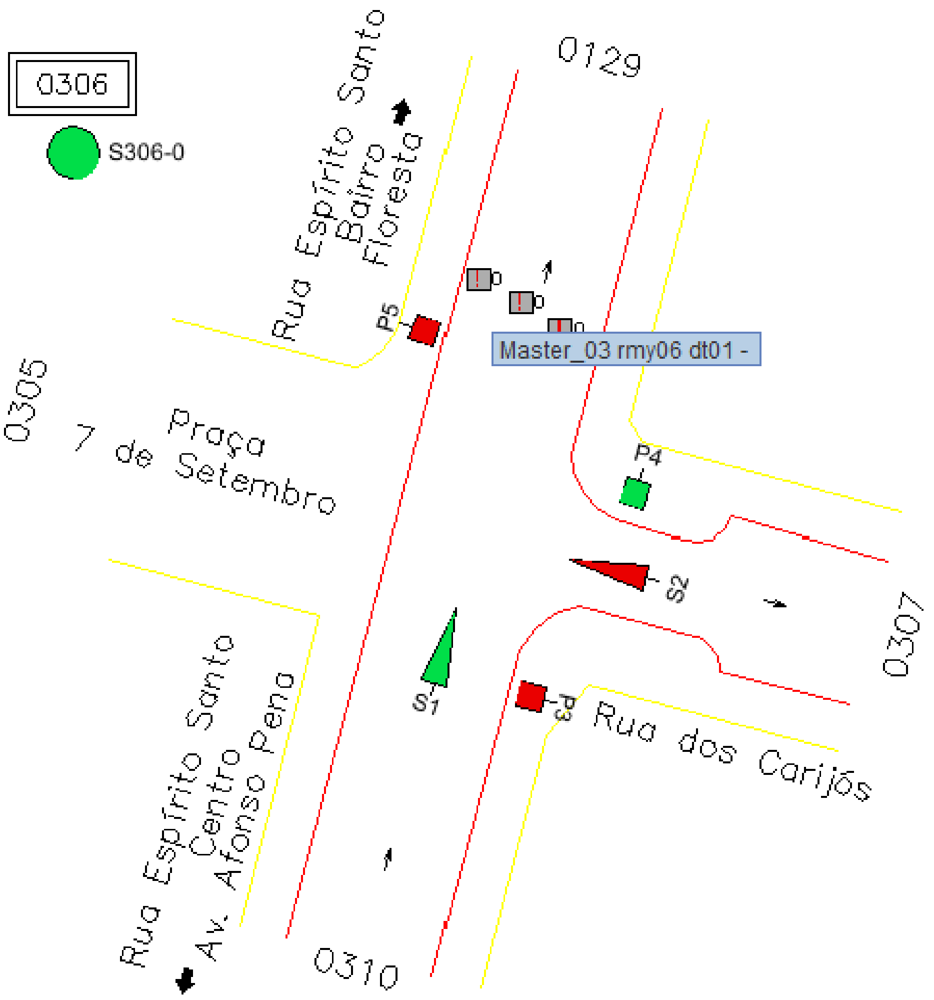
0306
S306-0
Rua Espírito Santo
Bairro
Floresta
0129
P5
Master_03 rmy06 dt01 -
0305
Praça
7 de Setembro
P4
S2
0307
S1
P3
Rua dos Carijós
Rua Espírito Santo
Centro
Av. Afonso Pena
0310

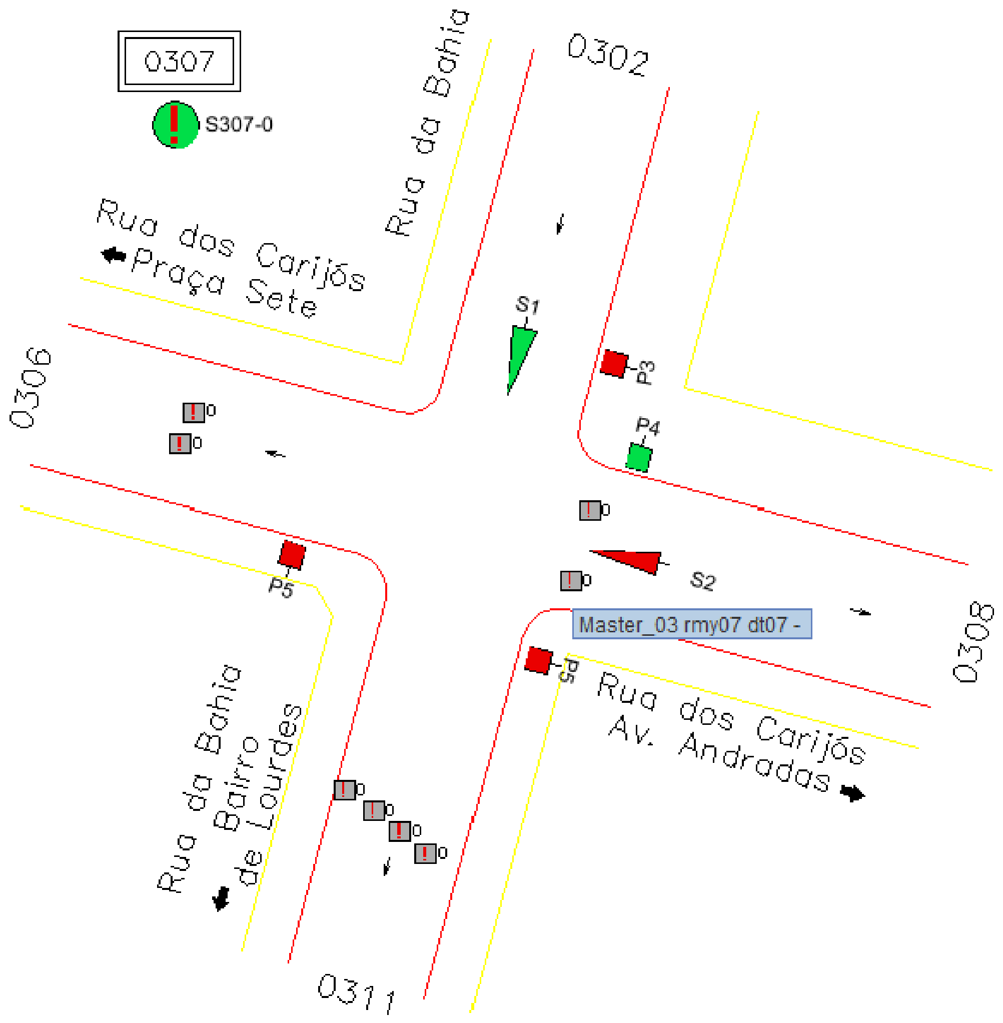
0307
S307-0
Rua da Bahia
0302
Rua dos Carijós
Praça Sete
S1
P3
P4
0306
P5
S2
Master_03 rmy07 dt07 -
0308
P5
Rua dos Carijós
Av. Andradas
Rua da Bahia
Bairro de Lourdes
0311

S1:1-4, S2:5-6

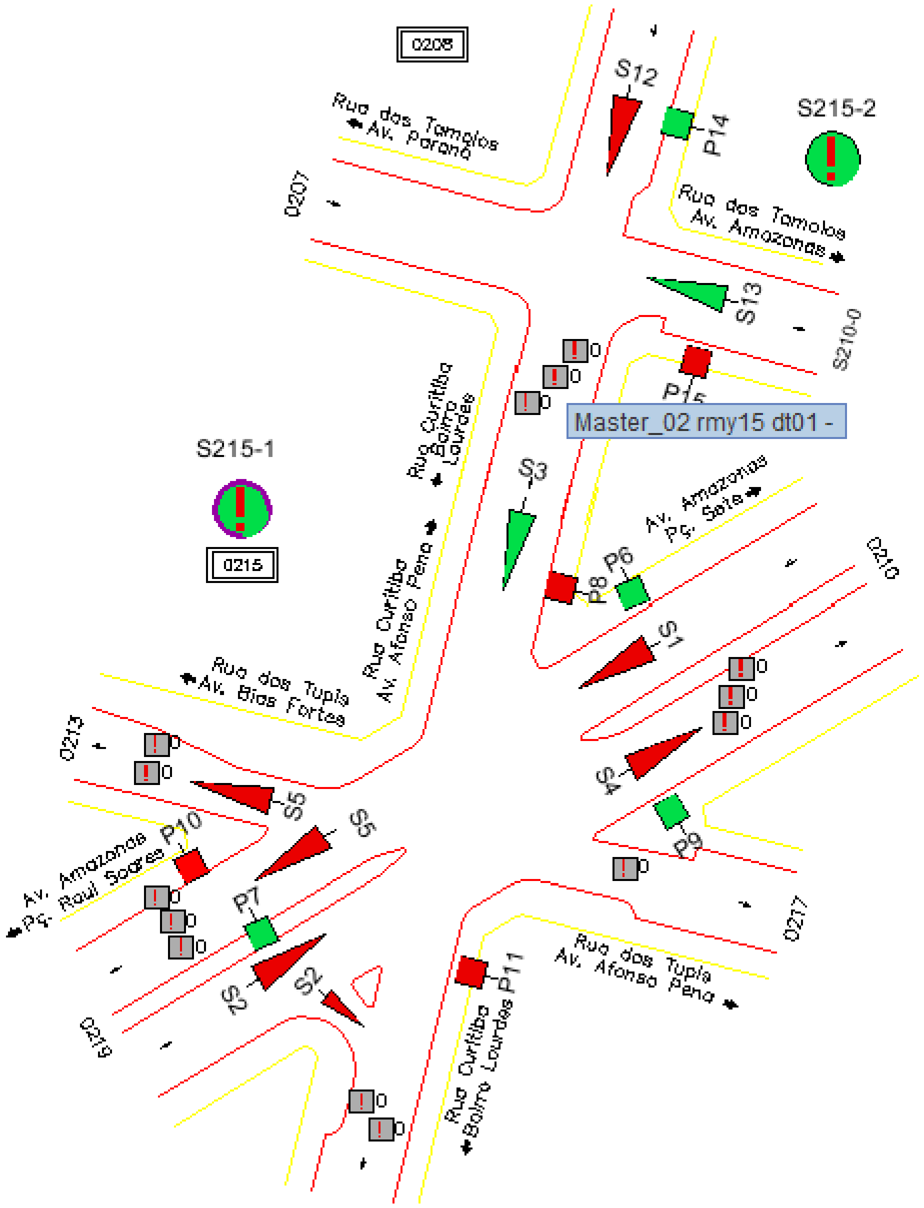

S4:4-6, S5-:7, S2:8-9, S5:10-12, S5:13-14,

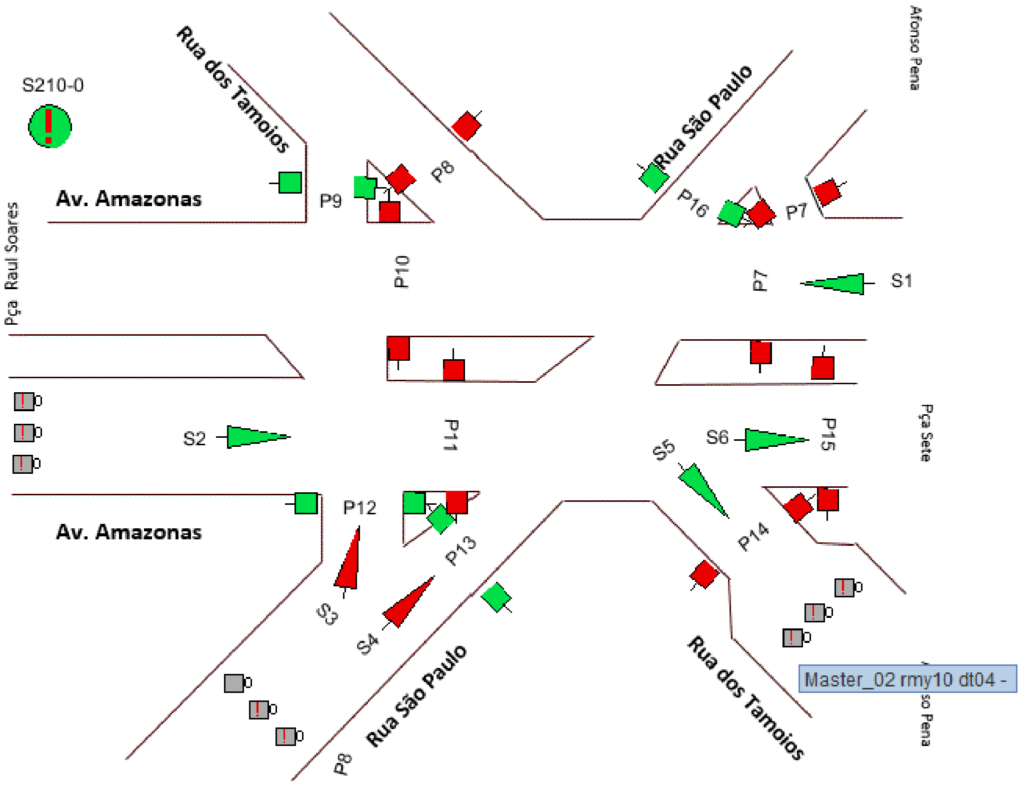
S210-0
Rua dos Tamoios
Rua São Paulo
Afonso Pena
Av. Amazonas
P9
P8
P16
P7
P10
P7
S1
Pça Raul Soares
P11
S2
S5
S6
P15
Pça Sete
P12
P13
P14
Av. Amazonas
S3
S4
Rua São Paulo
Rua dos Tamoios
Master_02 rmy10 dt04 -
P8

S4:7-9, S2:1-3,

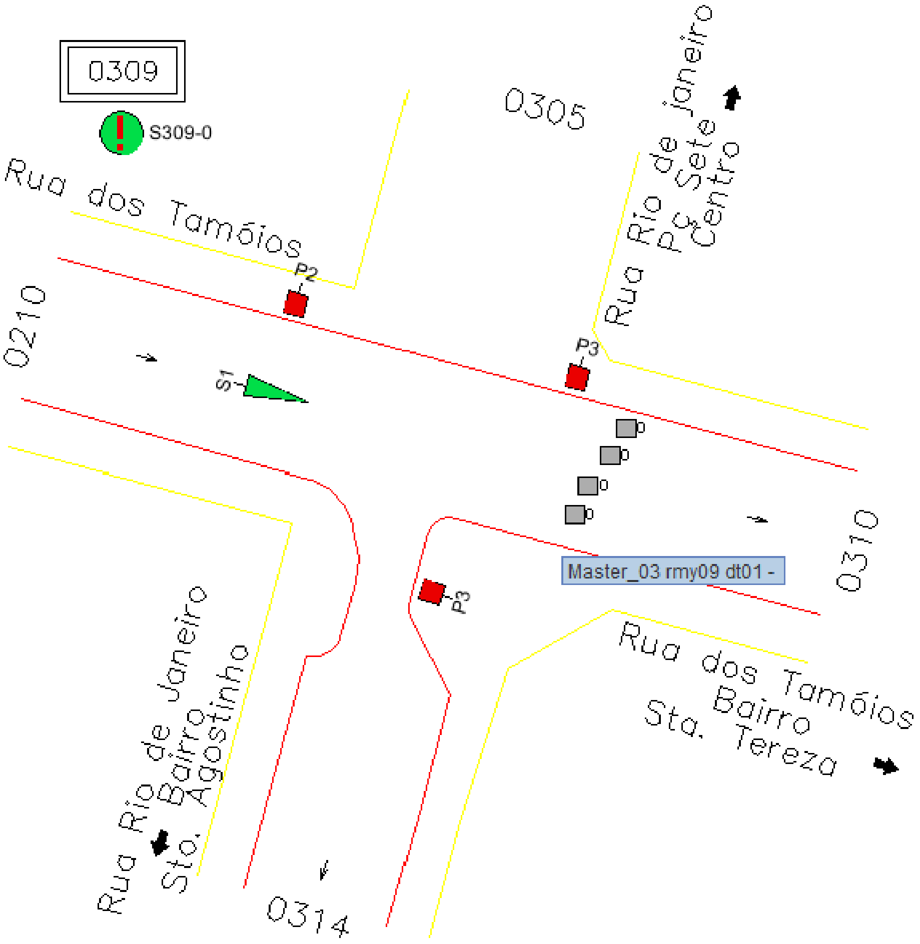
0309
S309-0
Rua dos Tamóios
0305
Rua Rio de janeiro
Pç Sete
Centro
P2
P3
0210
S1
Master_03 rmy09 dt01 -
0310
P3
Rua dos Tamóios
Bairro
Sta. Tereza
Rua Rio de Janeiro
Bairro
Sto. Agostinho
0314

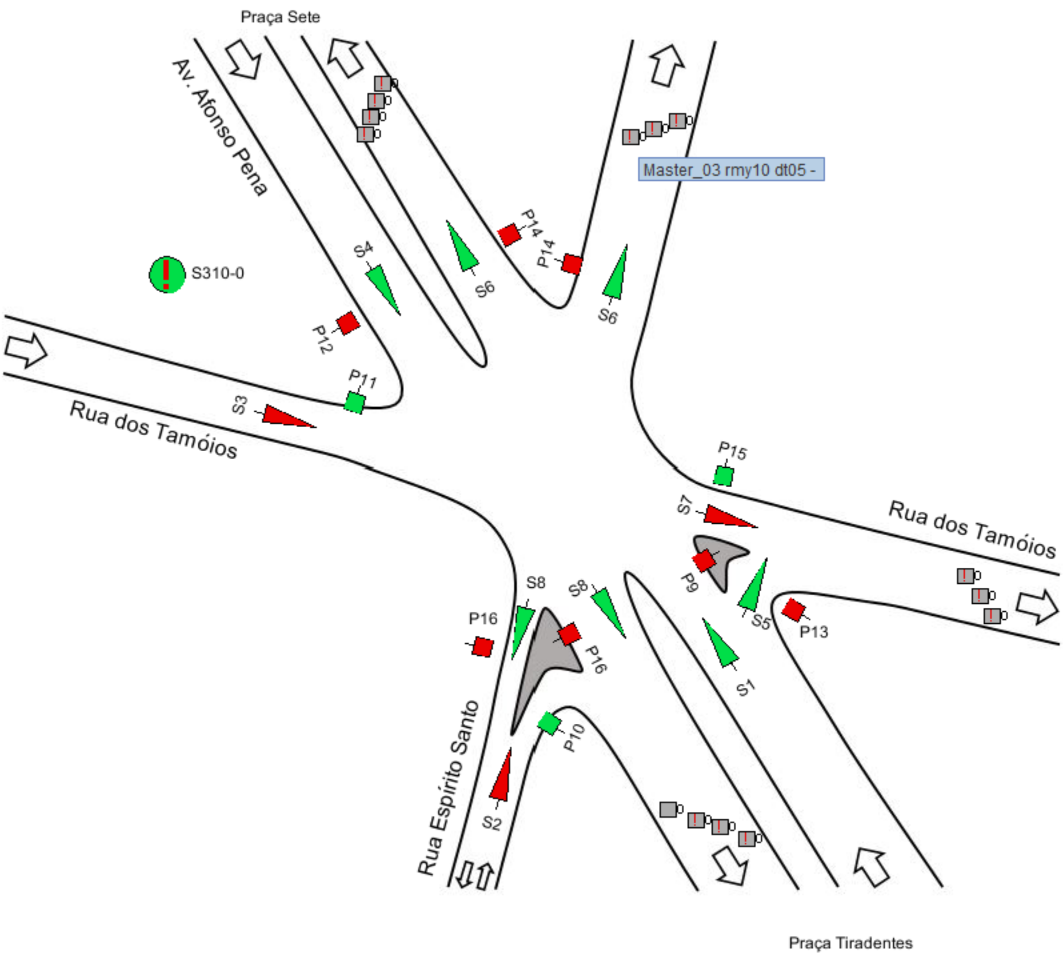

S7:8-10, S8:11-14, S6:1-4

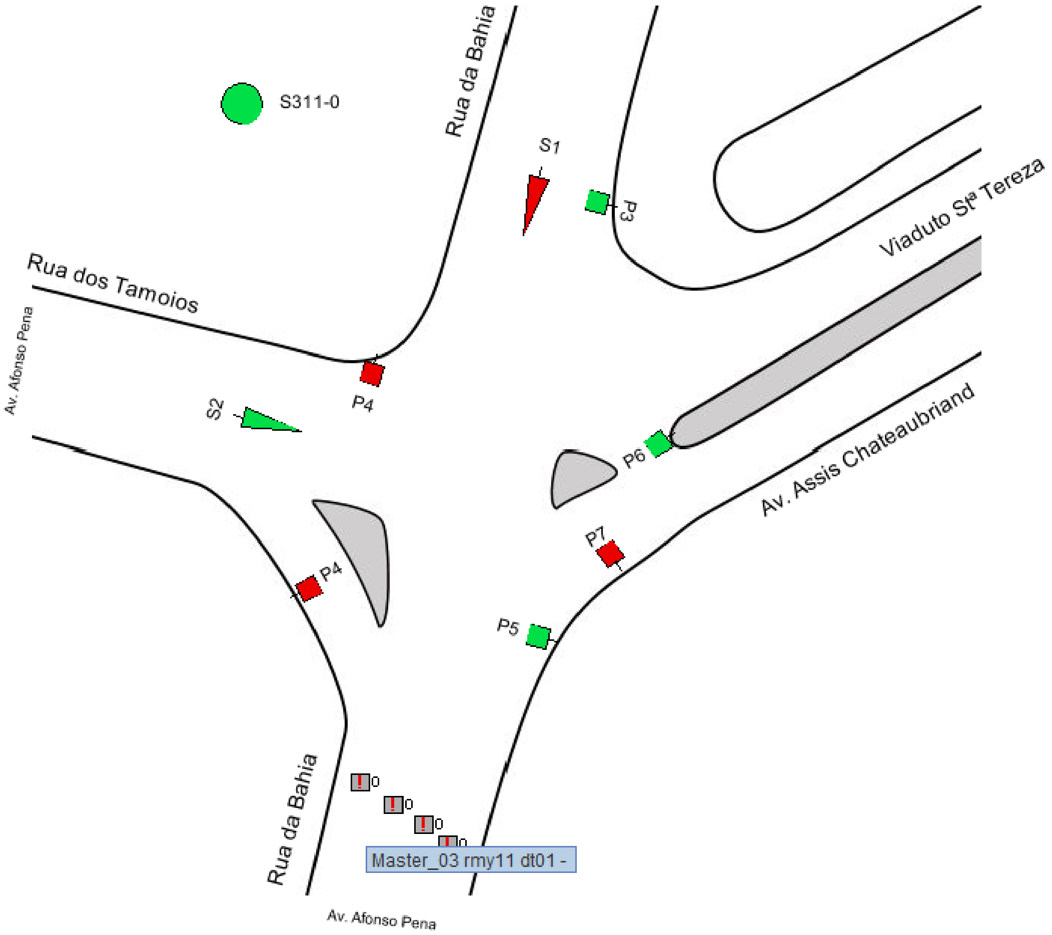
S311-0
Rua da Bahia
S1
P3
Viaduto Stª Tereza
Rua dos Tamoios
Av. Afonso Pena
P4
S2
P6
Av. Assis Chateaubriand
P7
P4
P5
Rua da Bahia
Master_03 rmy11 dt01 -
Av. Afonso Pena

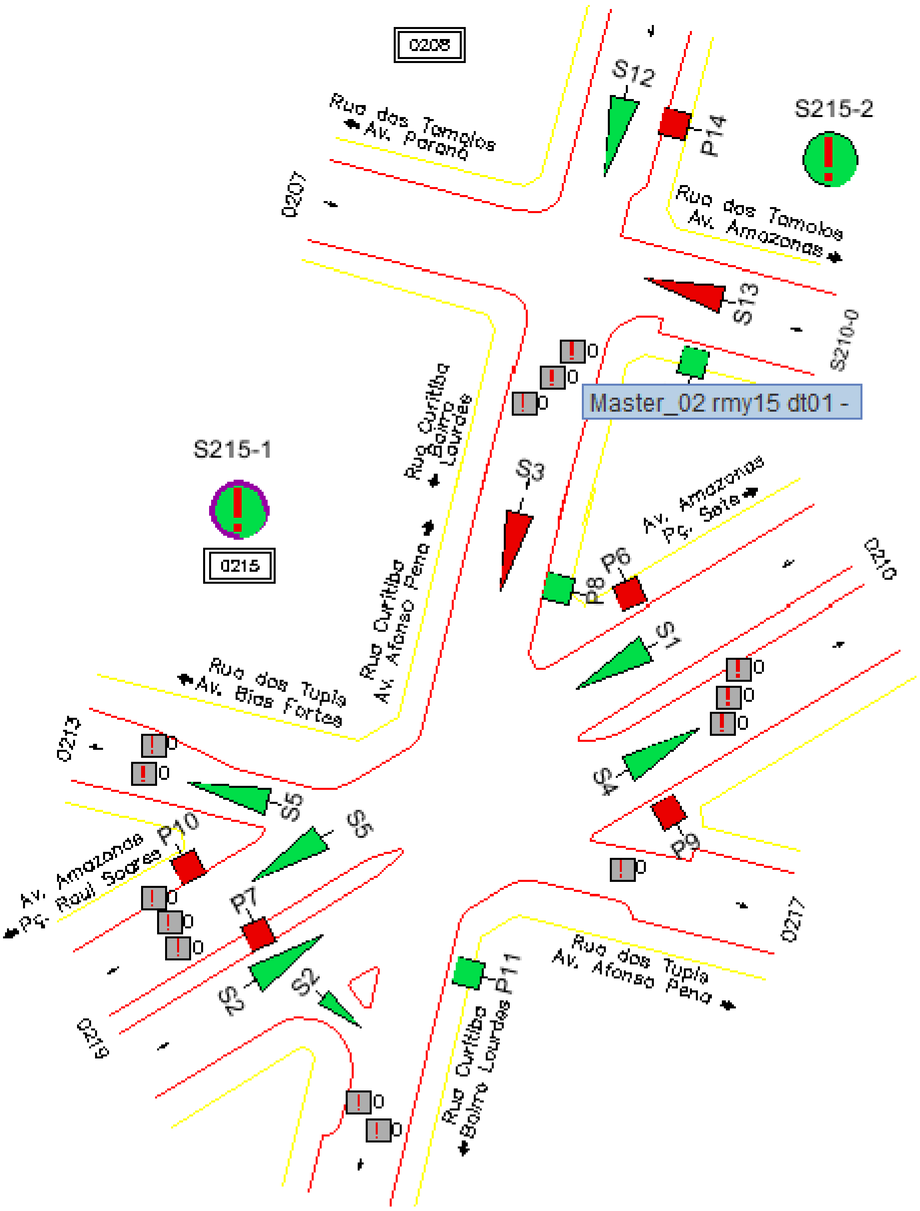

S4:4-6, S5-:7, S3:8-9, S5:10-12, S5:13-14

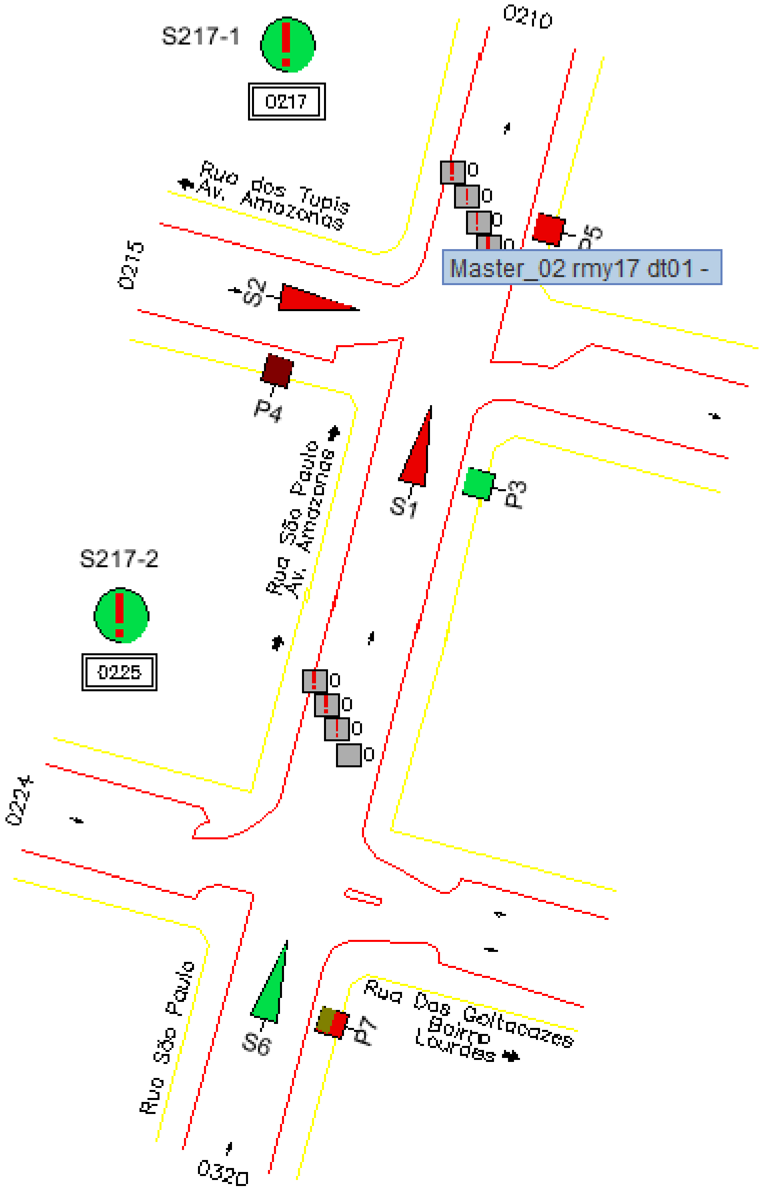
S217-1
0217
0210
Rua dos Tupis
Av. Amazonas
0215
S2
P5
Master_02 rmy17 dt01 -
P4
S1
P3
Rua São Paulo
Av. Amazonas
S217-2
0225
0224
Rua São Paulo
S6
P7
Rua Dos Goitacazes
Bairro Lourdes
0320

S6:5-8

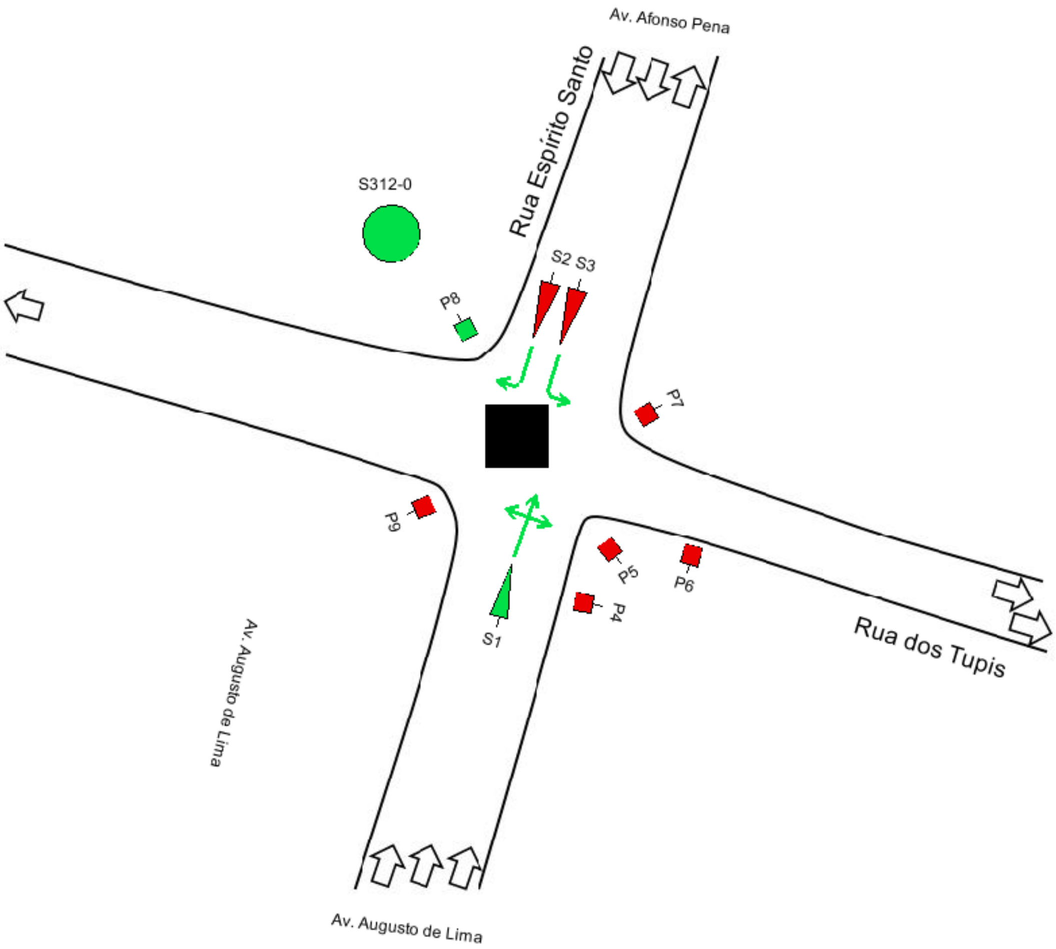
Av. Afonso Pena
Rua Espírito Santo
S312-0
S2 S3
P8
P7
P9
P5
P6
P4
S1
Rua dos Tupis
Av. Augusto de Lima
Av. Augusto de Lima

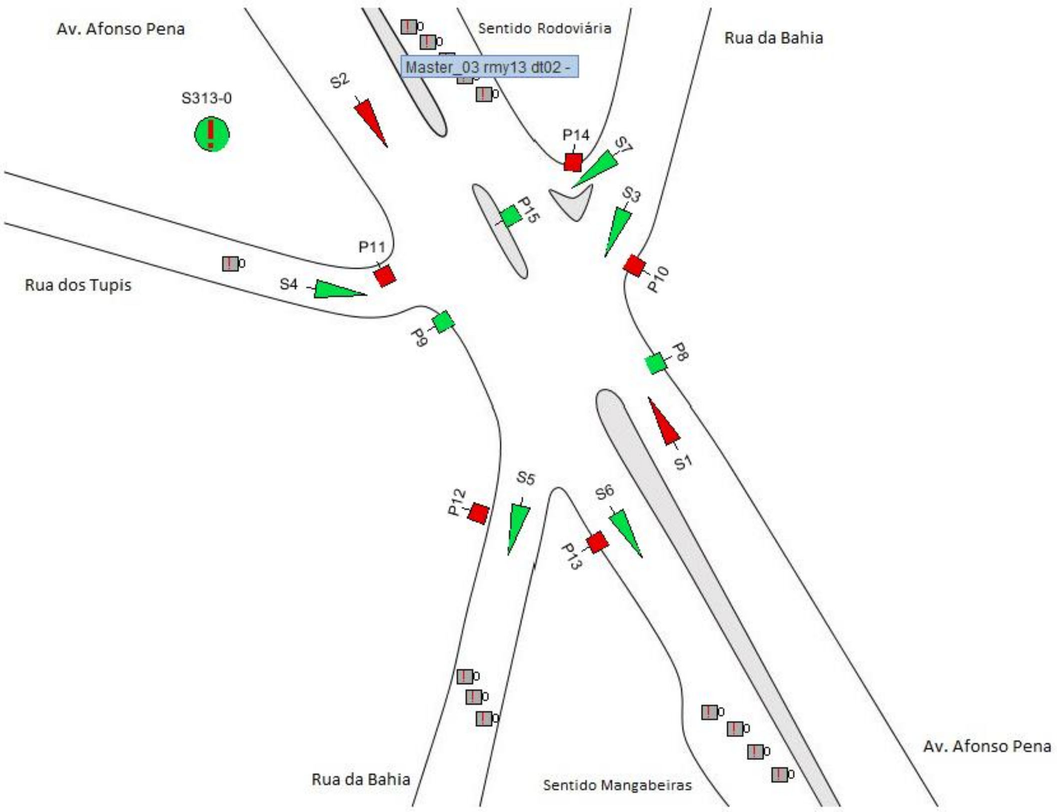

S6:7-10, S5:11-13, S4:1

Savassi

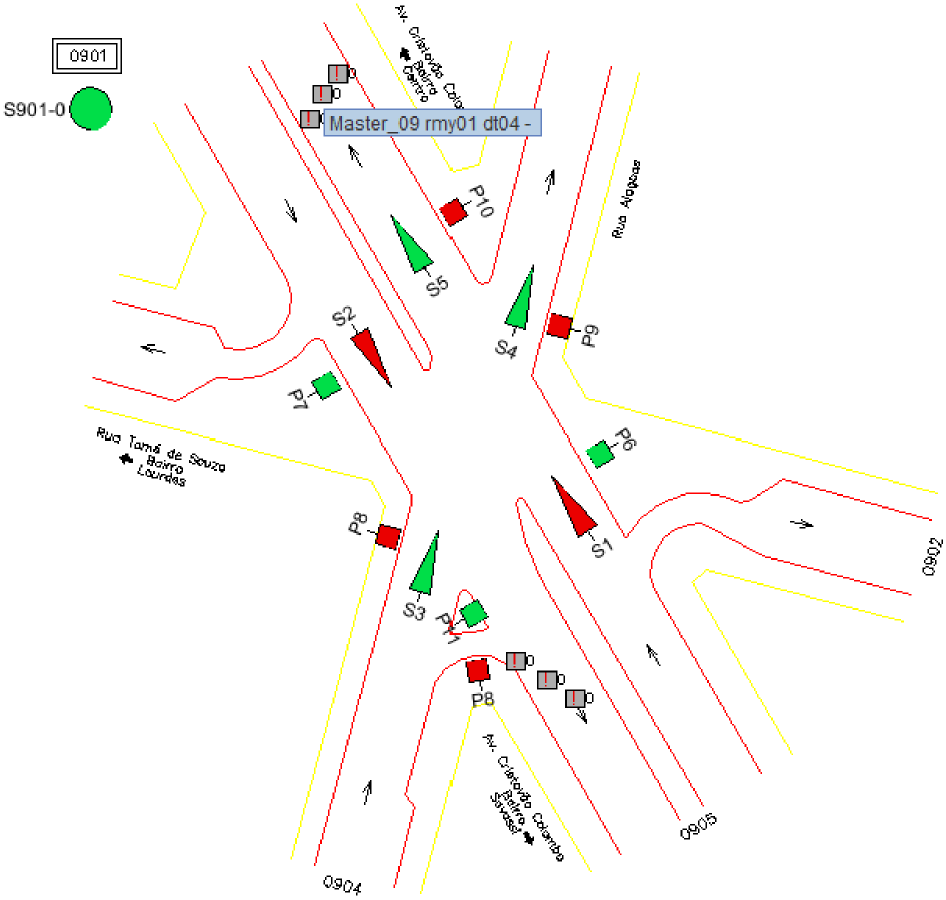

S2:1-3,

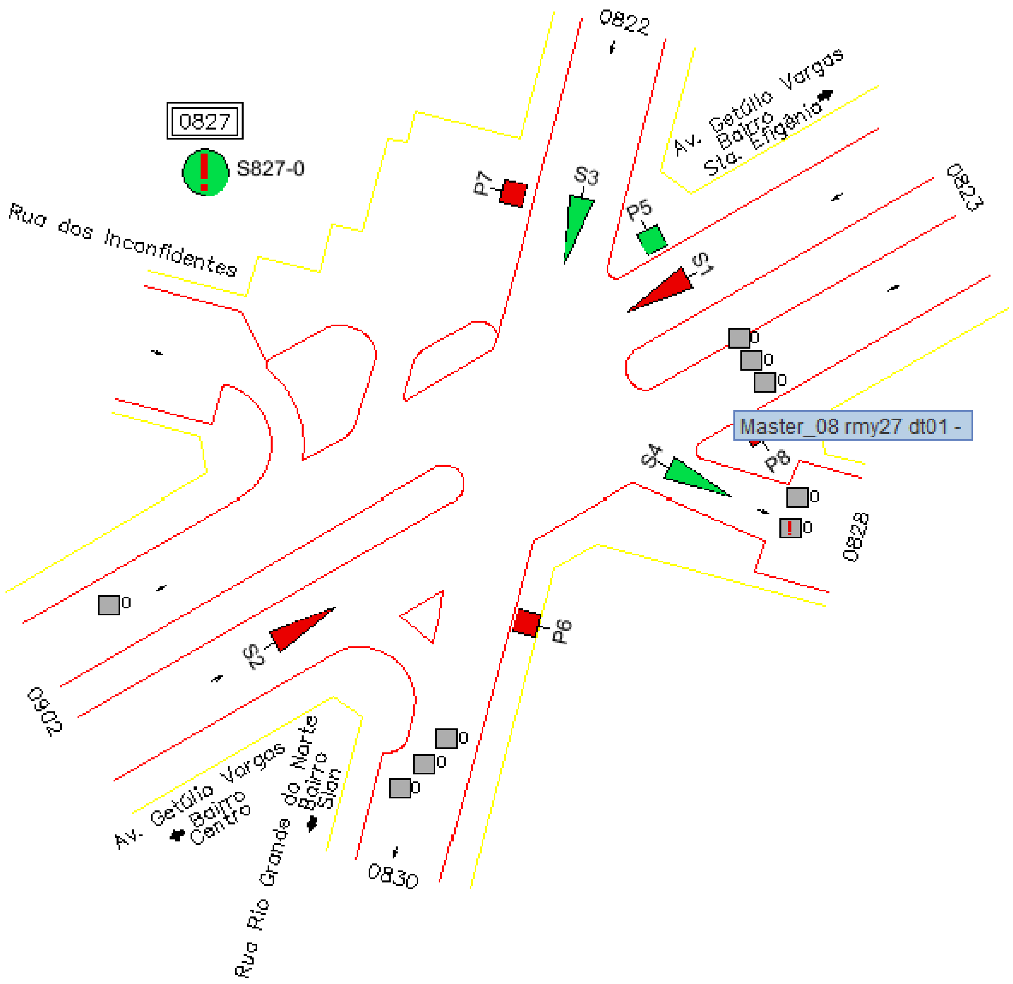

S4:4-5, S3:6-8, S1:9,

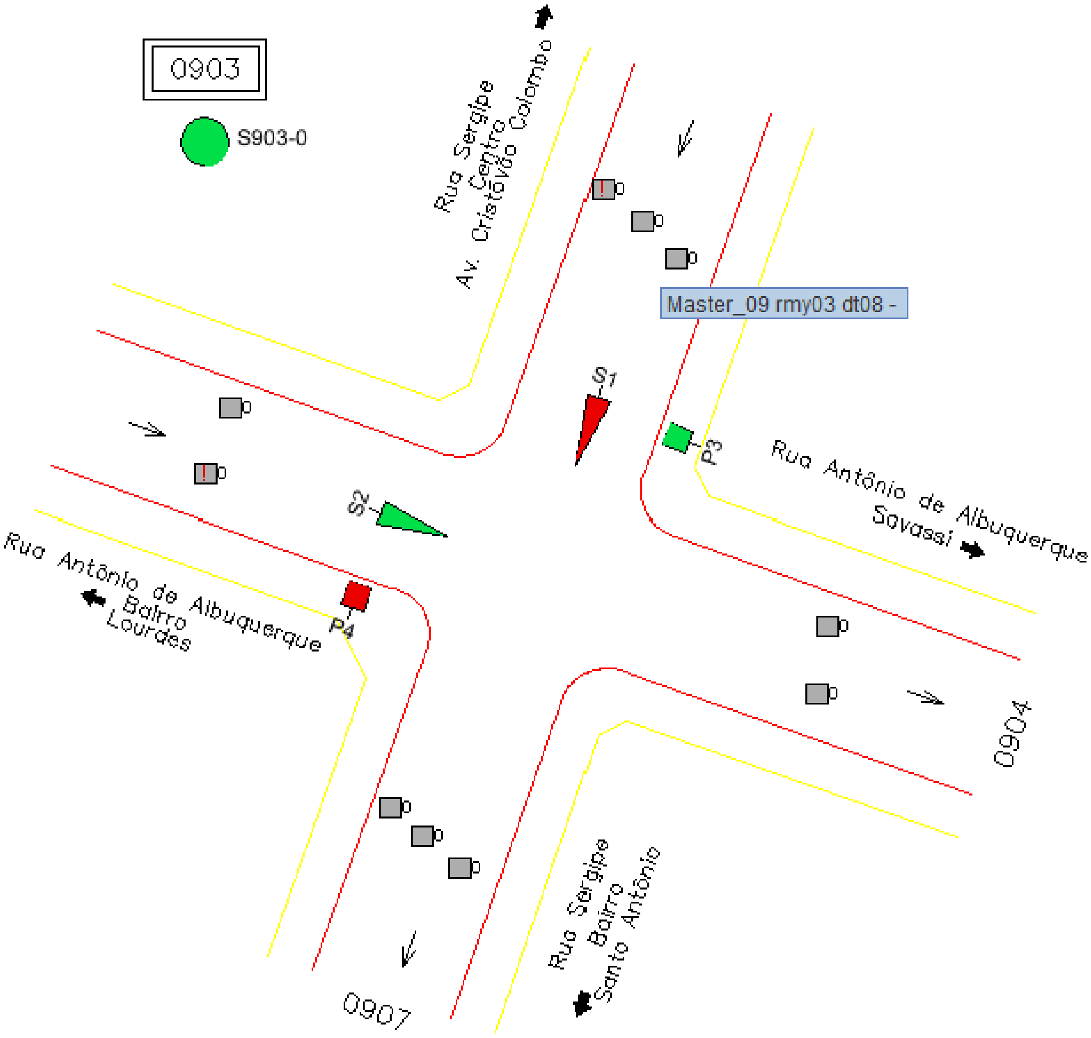

S2:1-2, S1:3-5, S2:6-7,

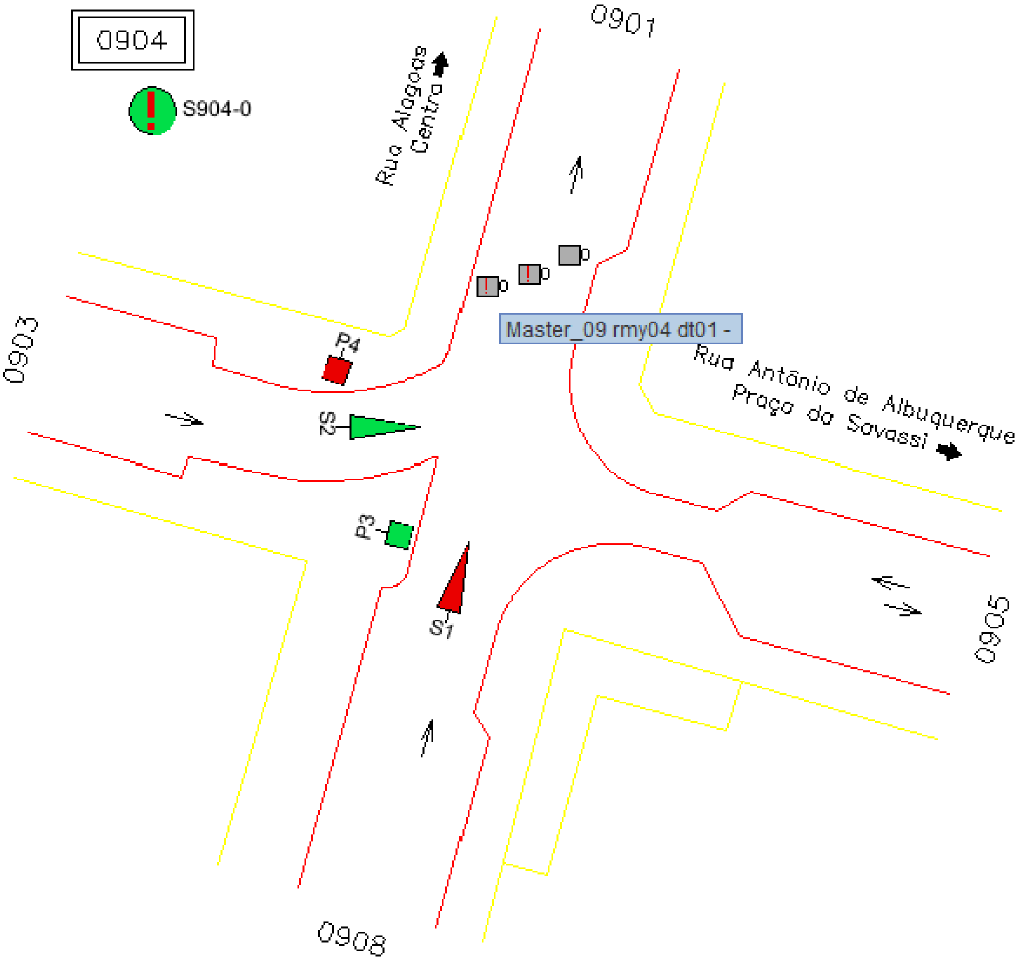

0904
S904-0
Rua Alagoas
Centro
0901
Master_09 rmy04 dt01 -
Rua Antônio de Albuquerque
Praça da Savassi
0903
P4
S2
P3
S1
0905
0908

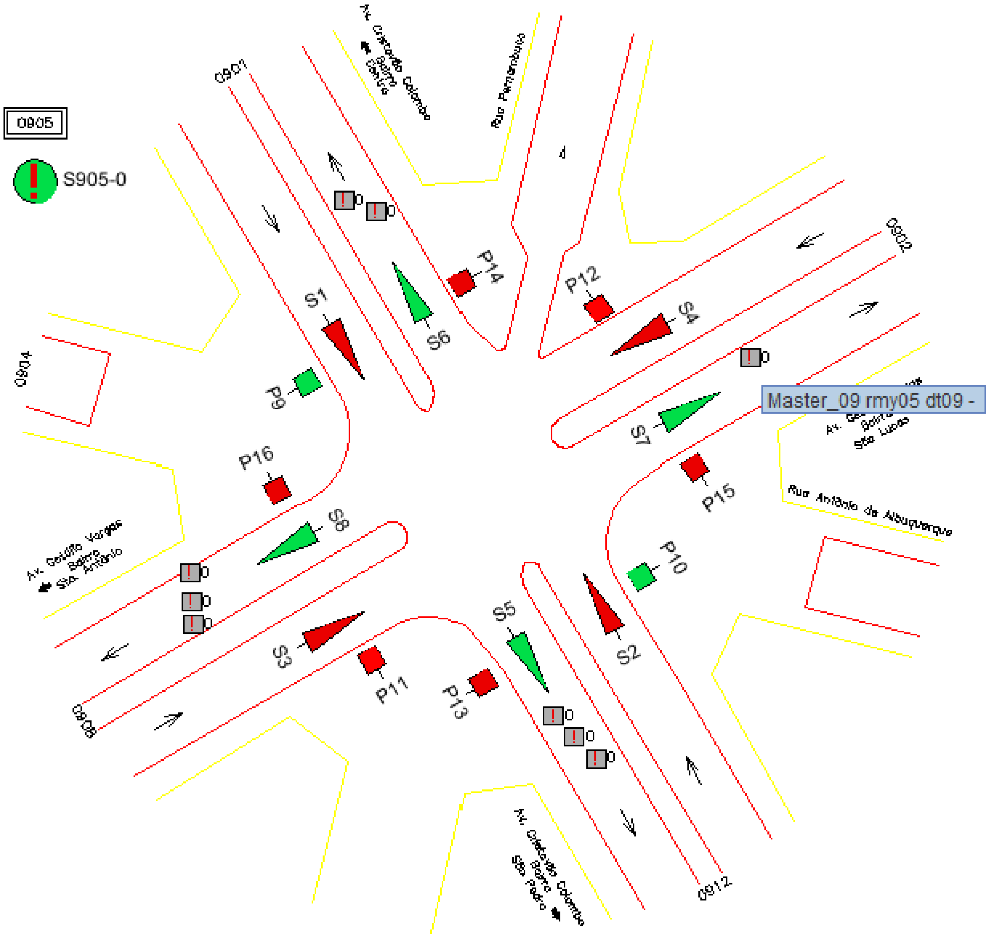

S5:1-3, S8:5-7, S6:7-8

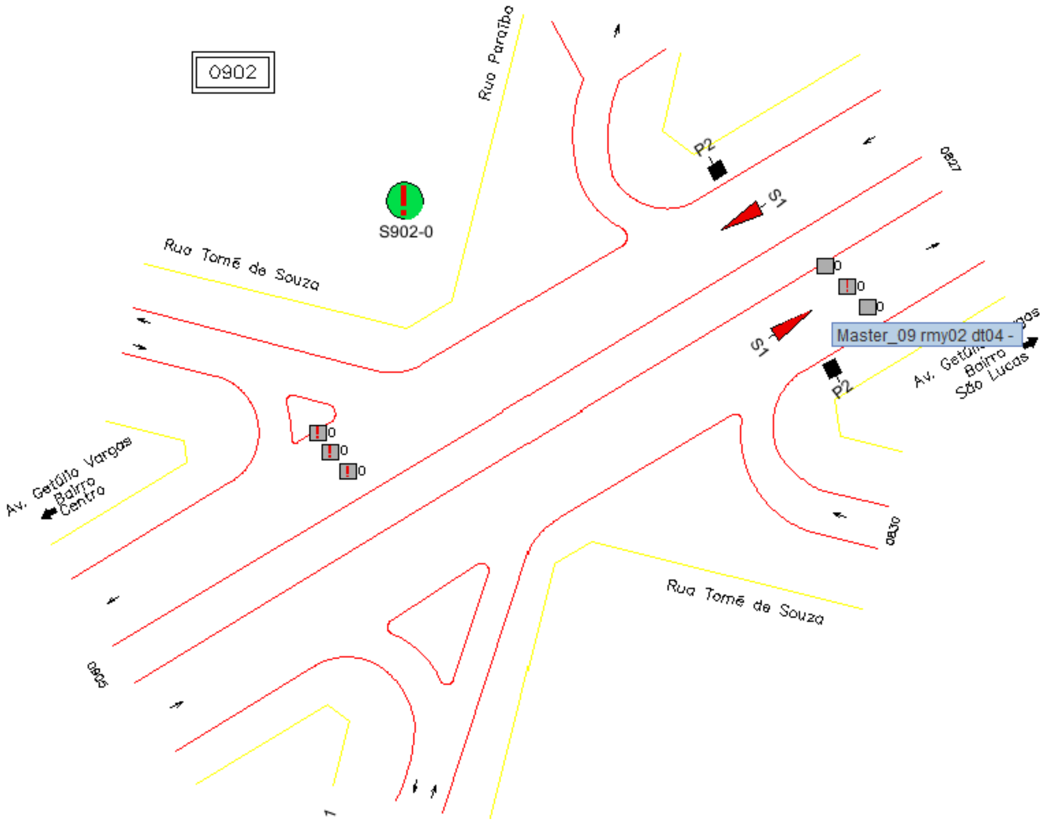

0902
Rua Paraíba
S902-0
Rua Tomé de Souza
P2
S1
Master_09 rmy02 dt04 -
Av. Getúlio Vargas Bairro Centro
Av. Getúlio Vargas Bairro São Lucas
Rua Tomé de Souza

S1:1-3

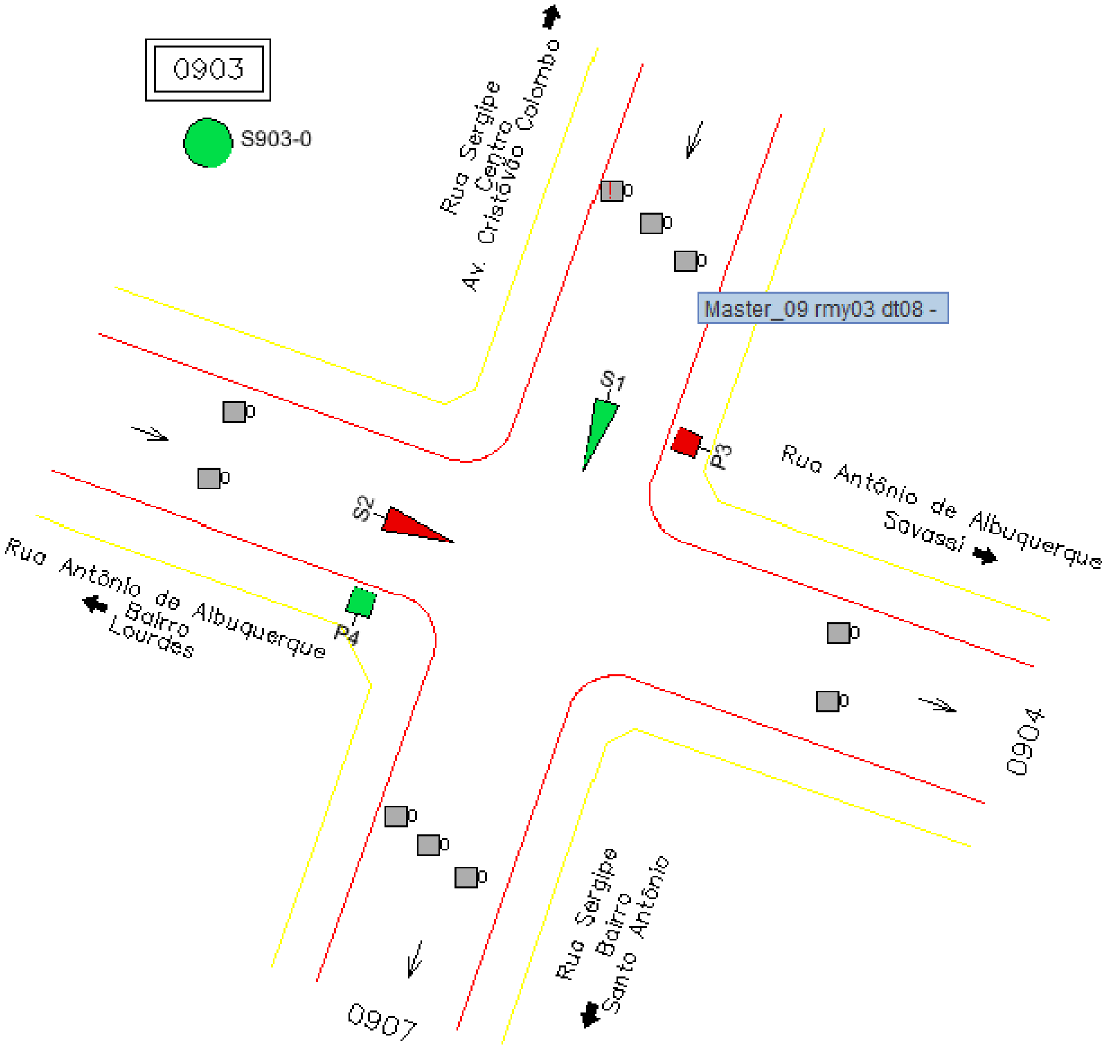

S2:1-2, S1:3-5, S2:6-7

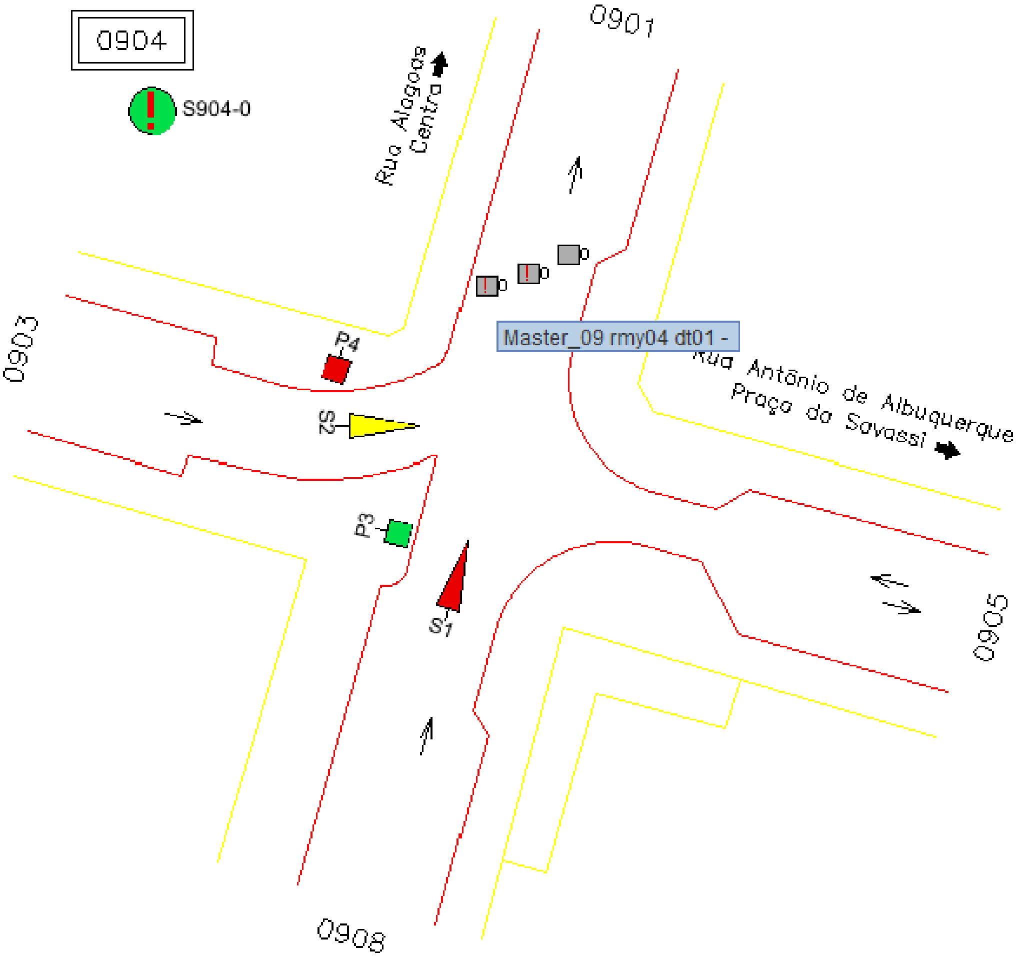
0904
S904-0
Rua Alagoas
Centro
0901
0903
Master_09 rmy04 dt01 -
Rua Antônio de Albuquerque
Praça da Savassi
P4
S2
P3
S1
0905
0908

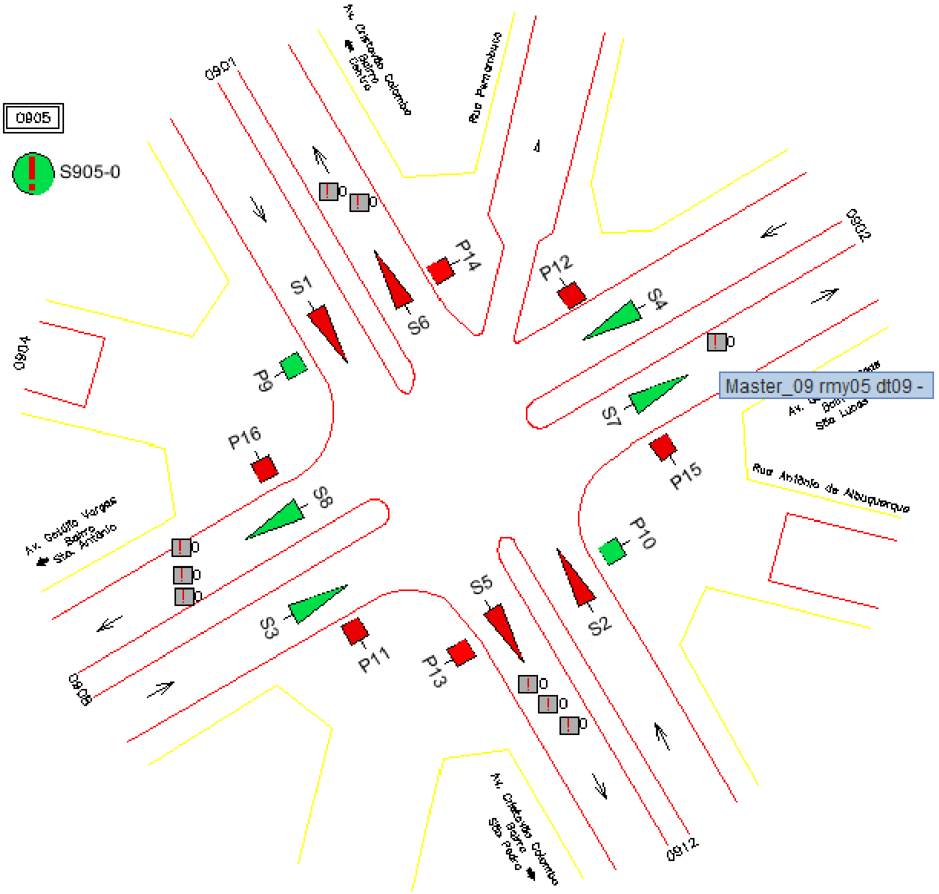

S4:1-3, S8:4-6, S6:7-8

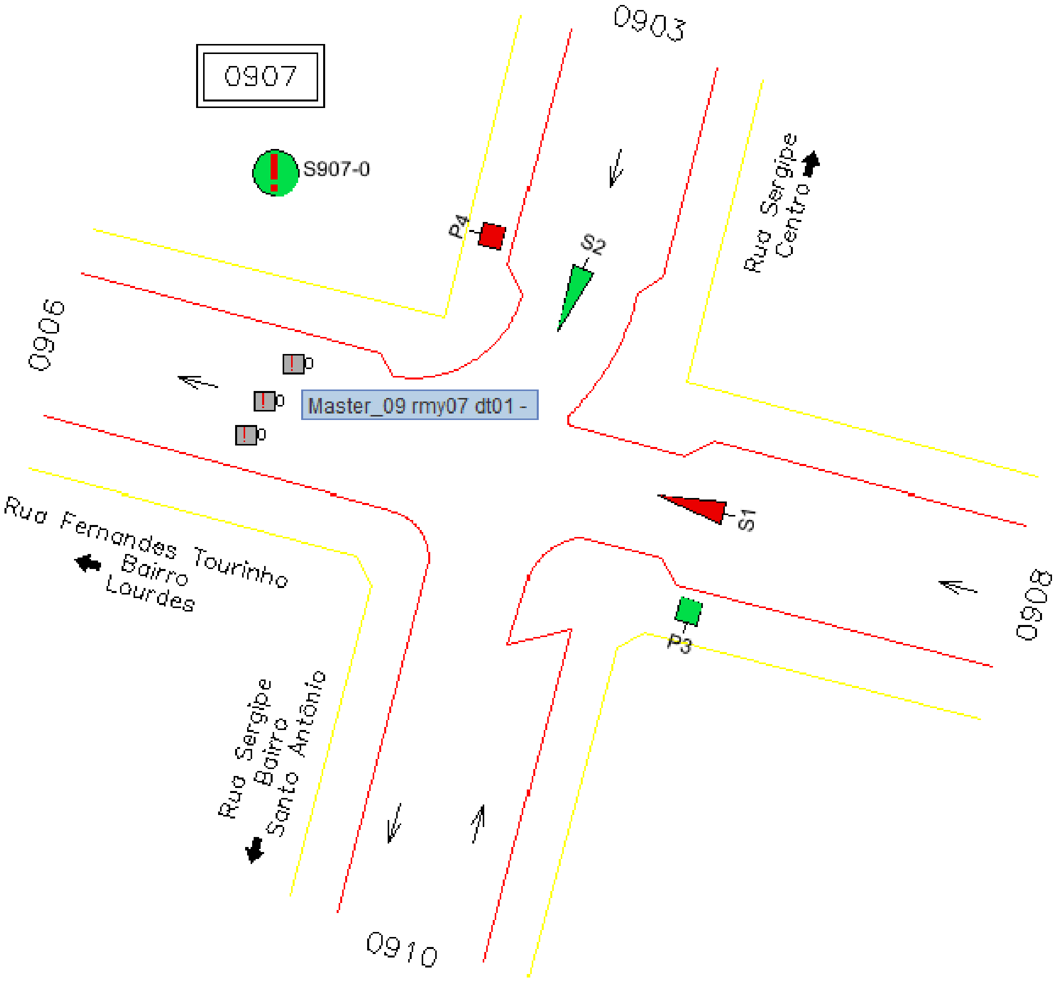

0907
S907-0
0903
Rua Sergipe
Centro
P4
S2
0906
Master_09 rmy07 dt01 -
S1
Rua Fernandes Tourinho
Bairro
Lourdes
P3
0908
Rua Sergipe
Bairro
Santo Antônio
0910

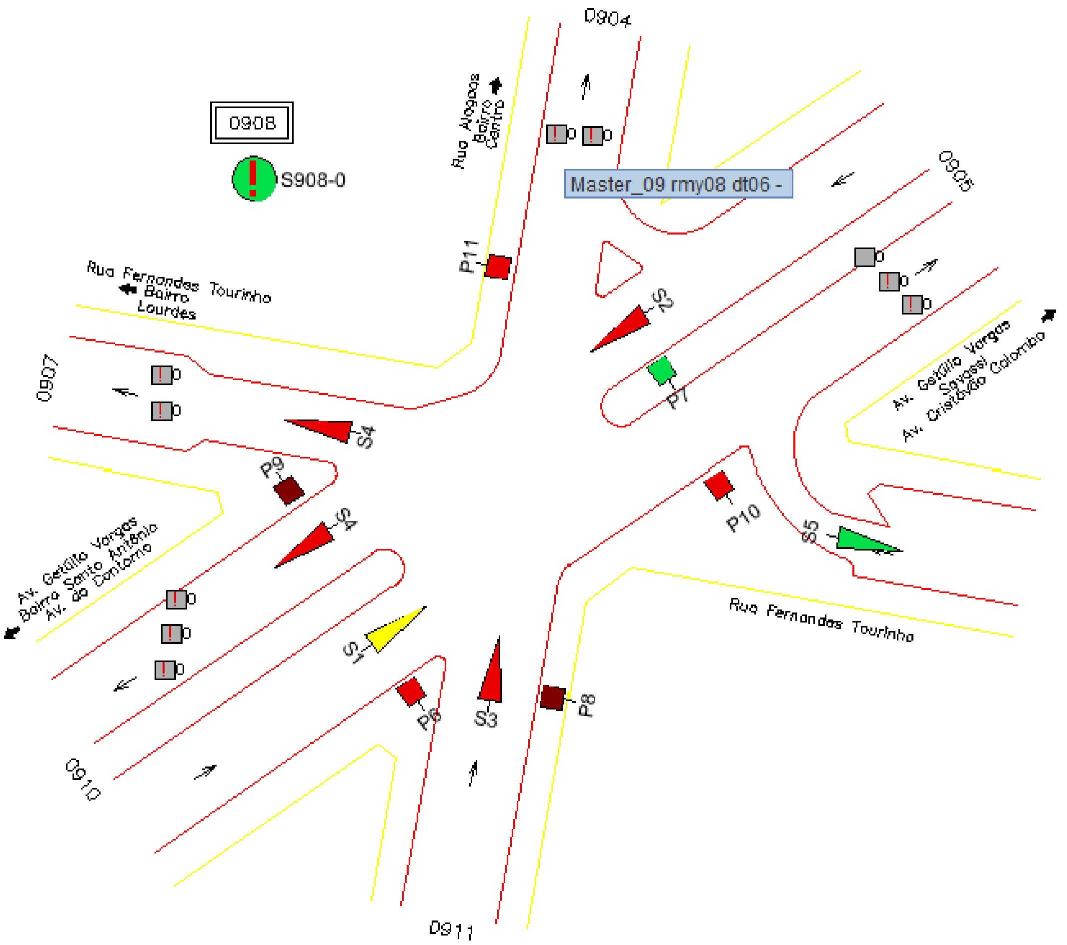

S1:8-10, S4:1-3, S4:4-5,

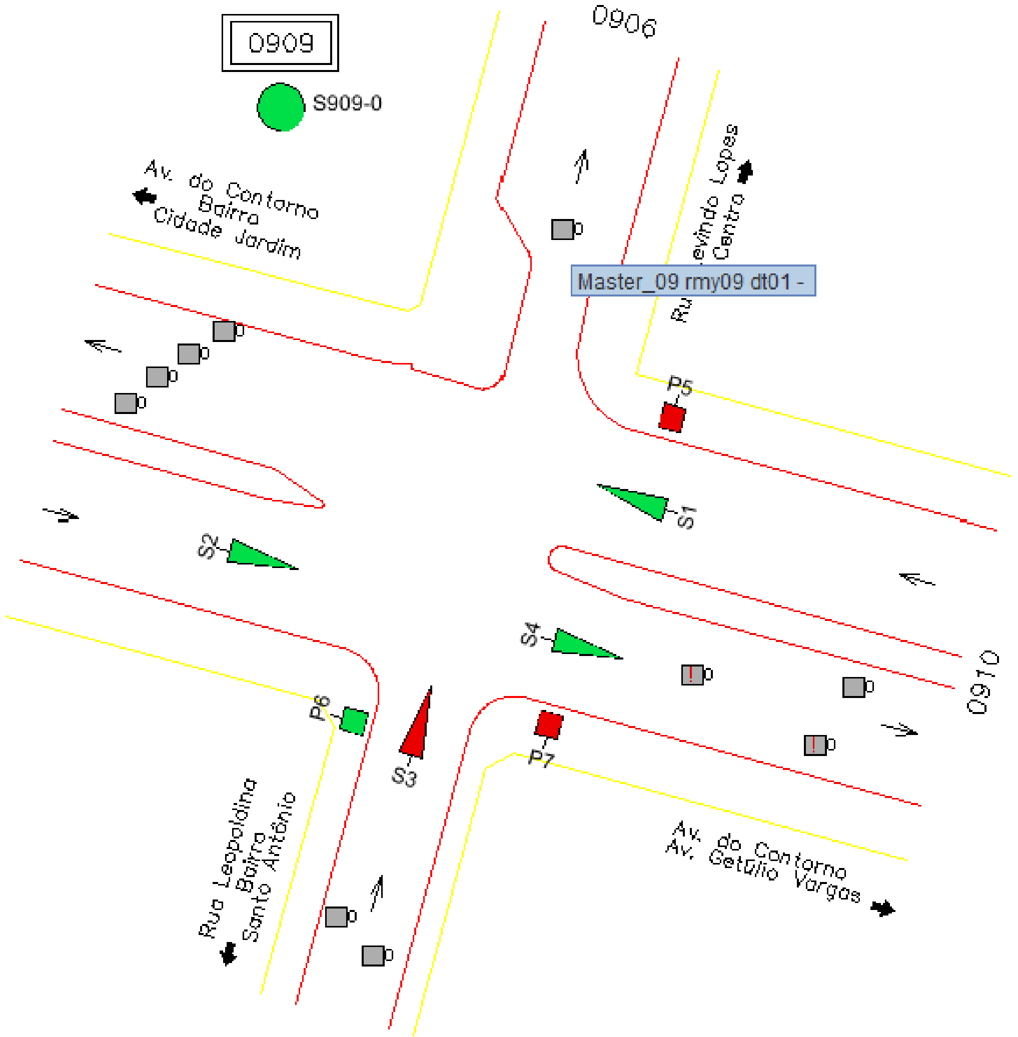

S4:2-4, S3:5-6S1:7-10

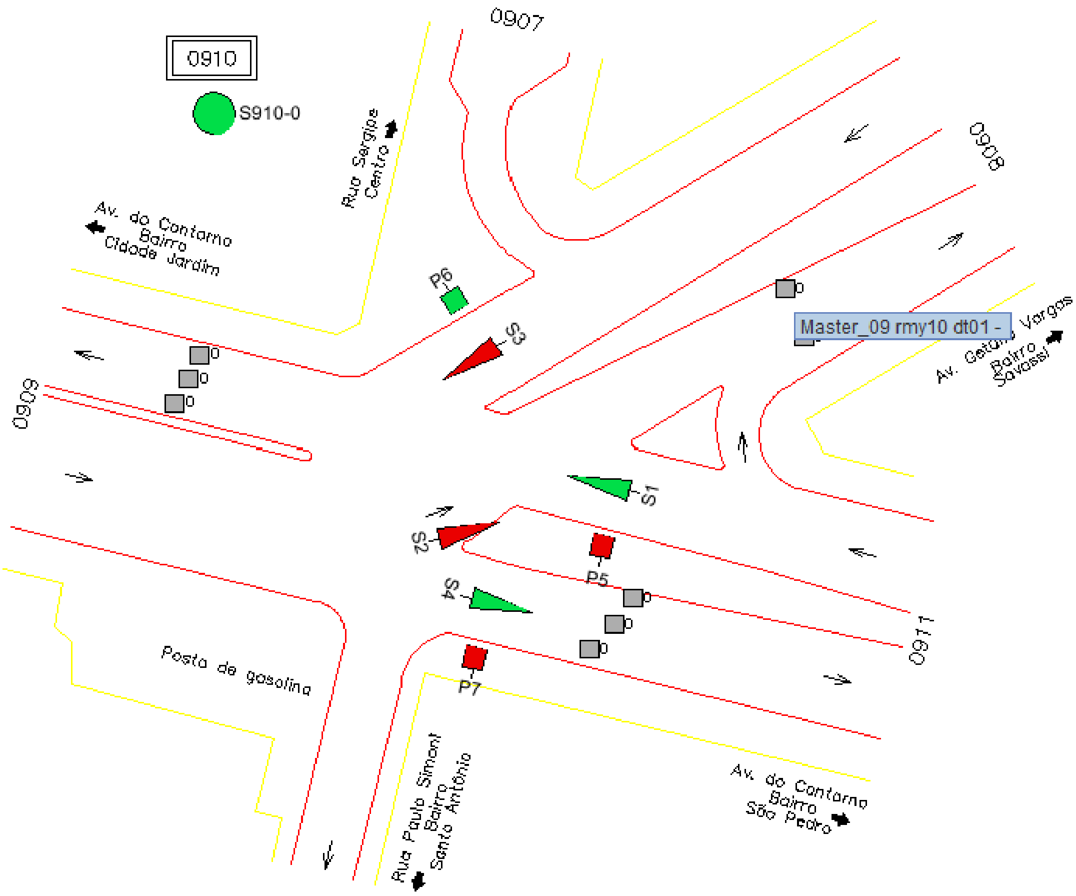
0910
S910-0
0907
0908
0909
0911
Rua Sergipe
Centro
Av. do Contorno
Bairro
Cidade Jardim
Master_09 rmy10 dt01 -
Bairro
Savassi
P6
S3
S1
S2
S4
P5
P7
Posto de gasolina
Rua Paulo Simoni
Bairro
Santo Antônio
Av. do Contorno
Bairro
São Pedro

S4:3-5, S1:6-8

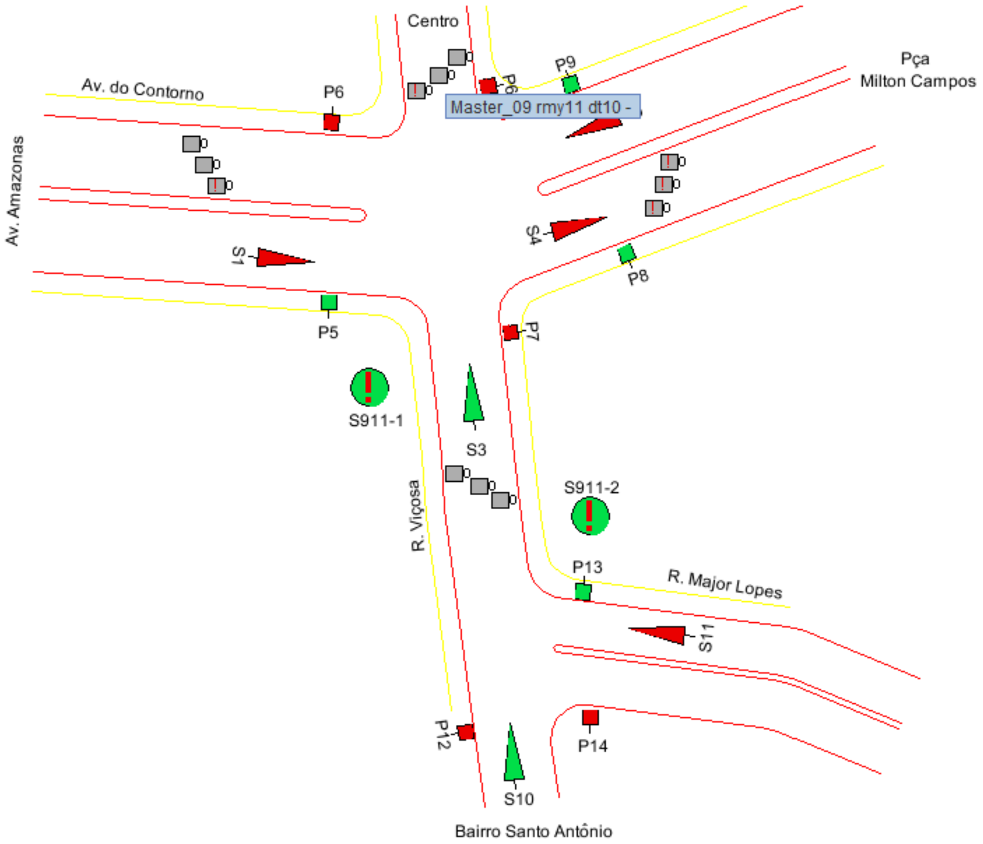

S4:4-6, S3:1-3, S2:7-9

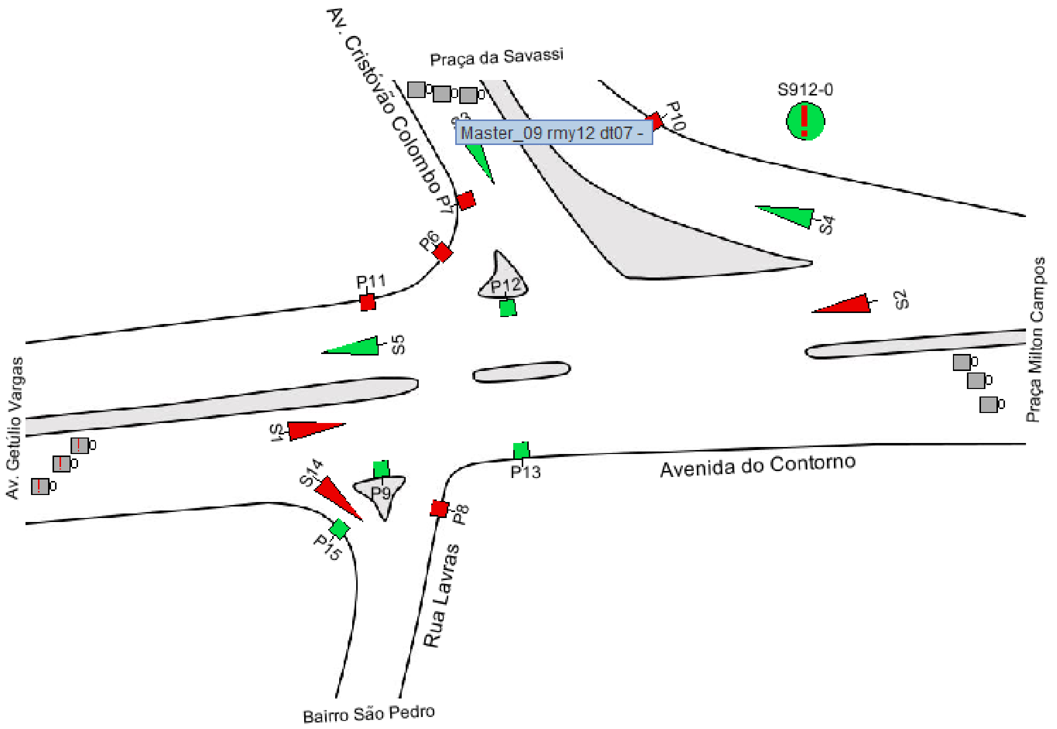

Av. Cristóvão Colombo
Praça da Savassi
S912-0
Master_09 rmy12 dt07 -
P10
P7
P6
P11
P12
S4
S2
S5
Praça Milton Campos
Av. Getúlio Vargas
S1
S14
P9
P13
P8
P15
Avenida do Contorno
Rua Lavras
Bairro São Pedro

S1:1-3, S1-:4-6

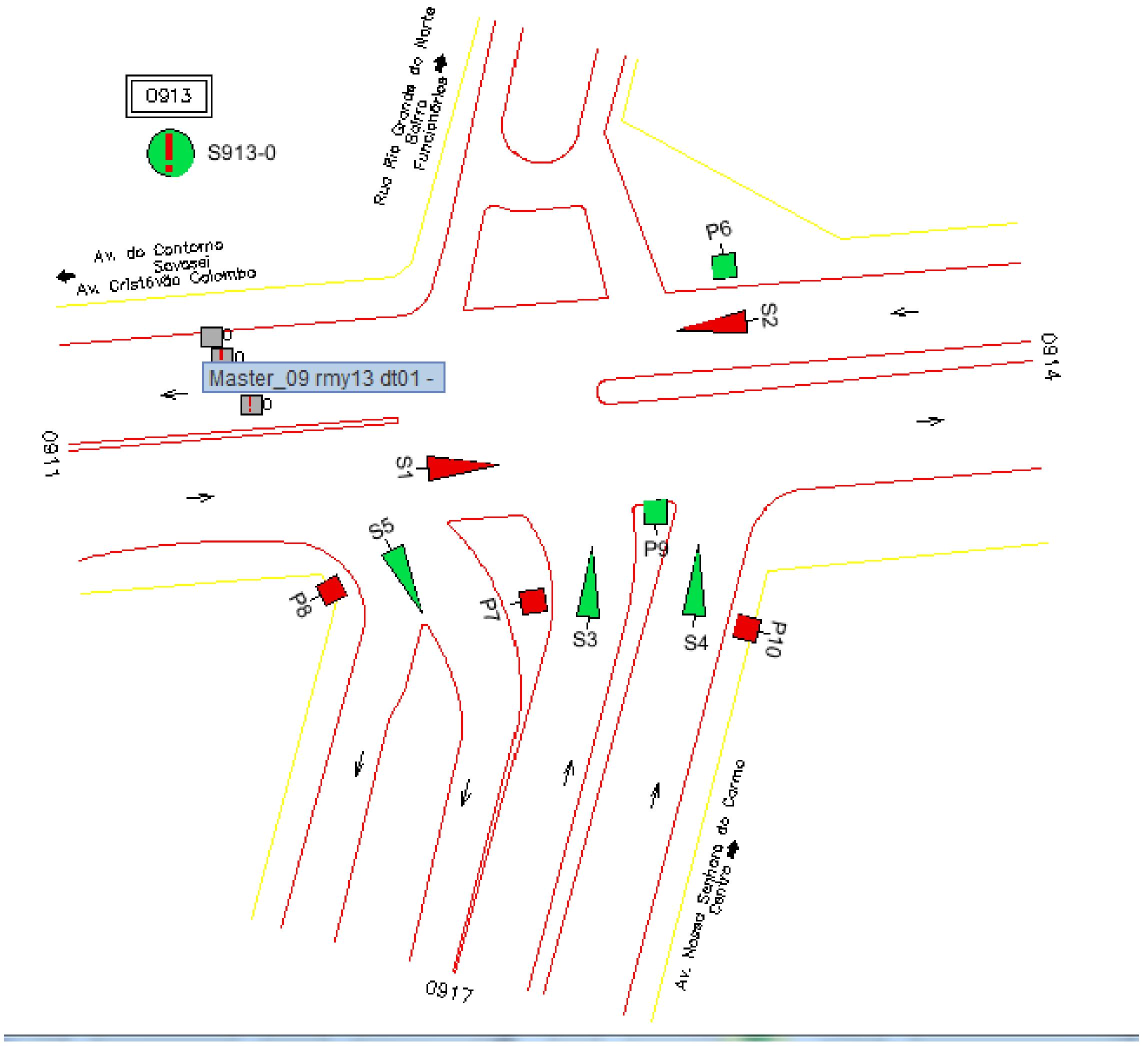
0913
S913-0
Rua Rio Grande do Norte
Bafra
Funcionários
Av. do Contorno
Savassi
Av. Cristóvão Colombo
P6
S2
0914
Master_09 rmy13 dt01 -
0911
S1
S5
P8
P7
S3
P9
S4
P10
Av. Nossa Senhora do Carmo
Centro
0917

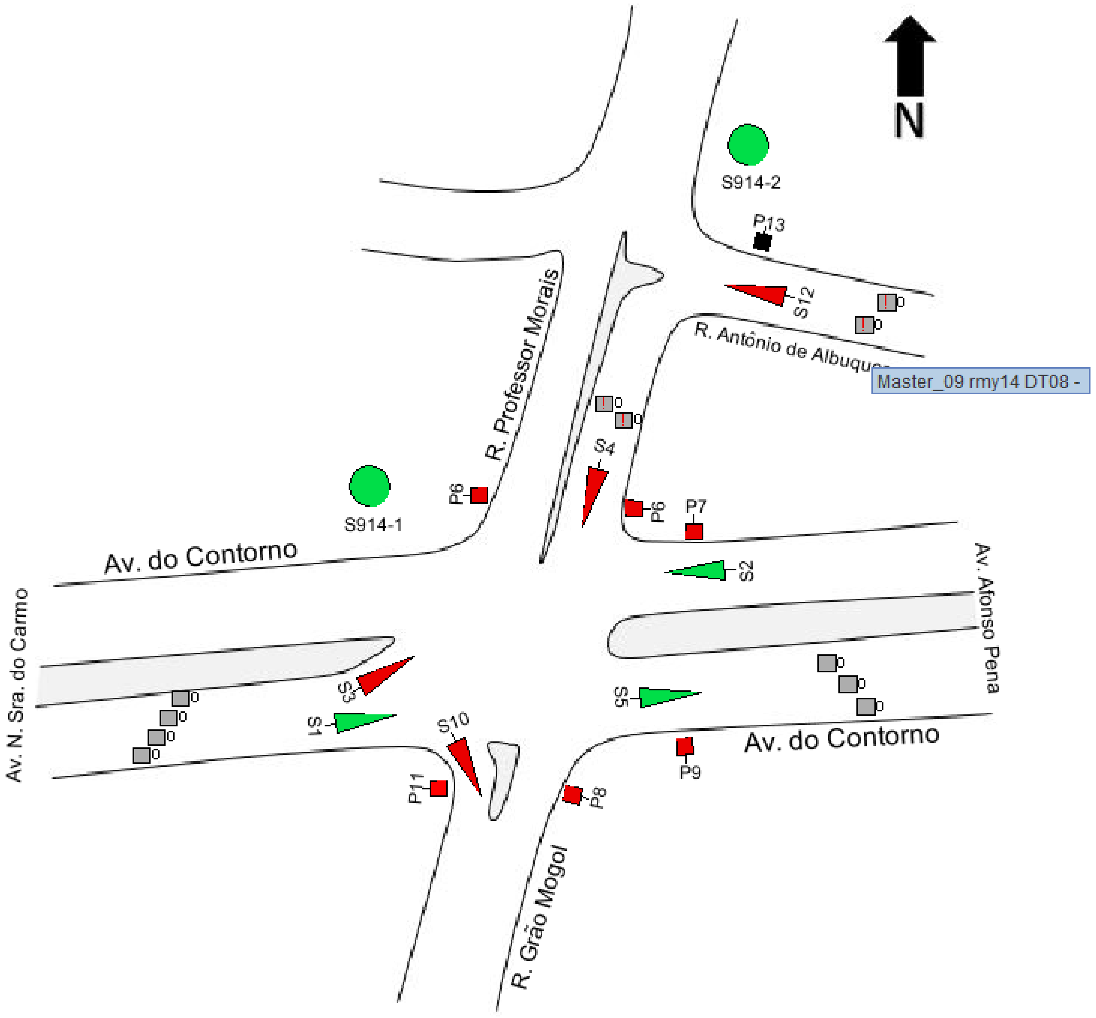

S5:1-3, S1-:4-7, S4:10-11

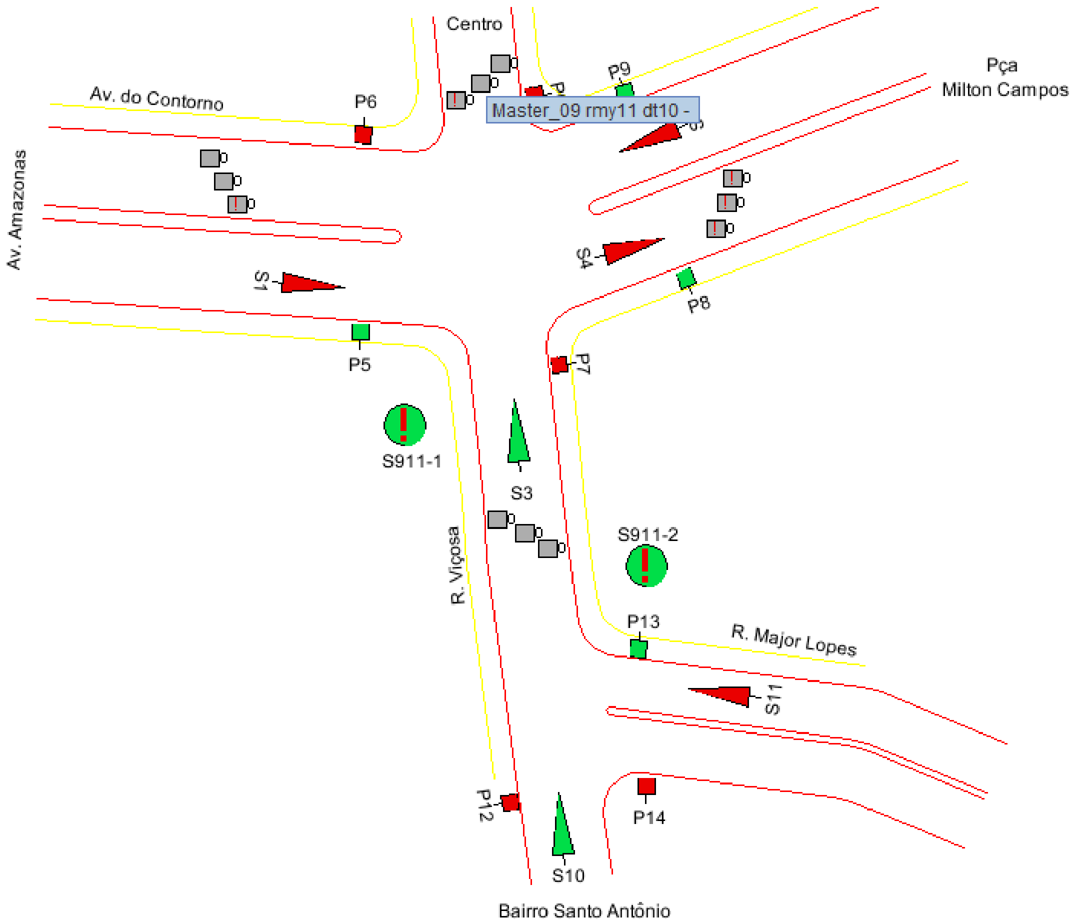

S4:4-6, S3:1-3, S2:7-9

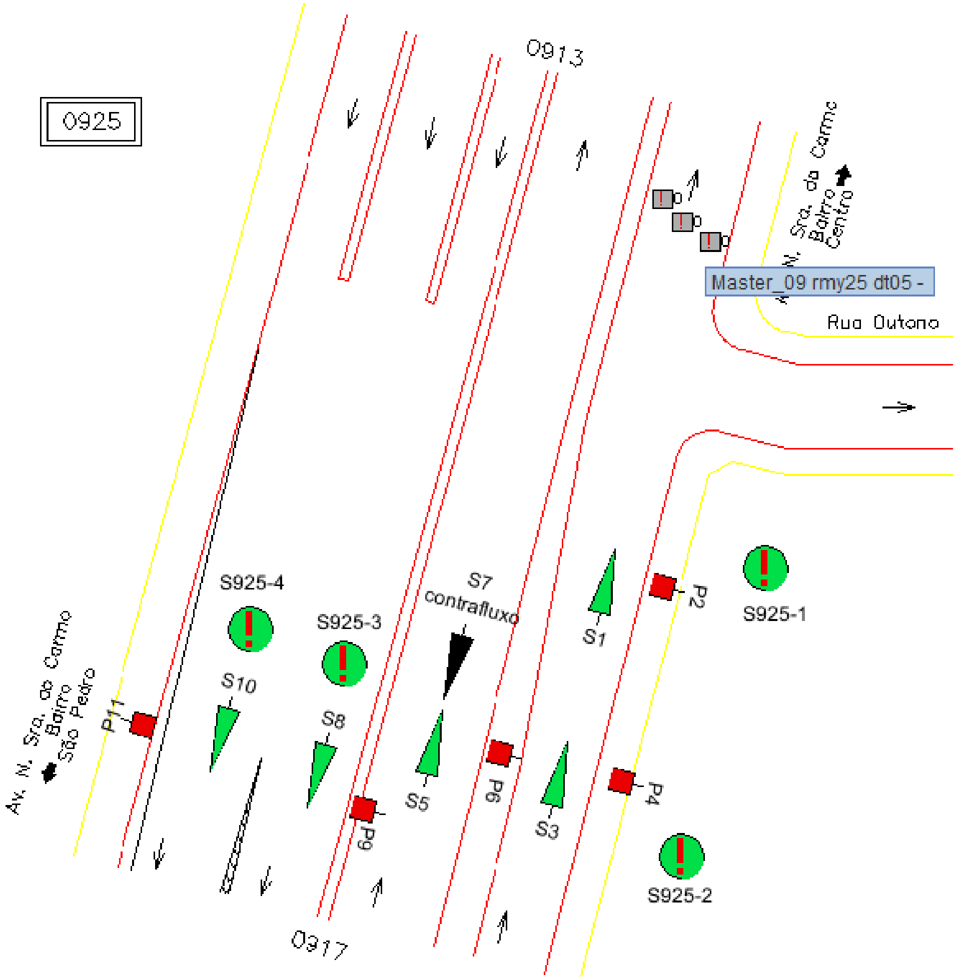
0925
0913
0917
Master_09 rmy25 dt05 -
Rua Outono
Av. N. Sra. do Carmo Bairro São Pedro
S925-1
S925-2
S925-3
S925-4
S7 contrafluxo
S1
S3
S5
S8
S10
P2
P4
P6
P9
P11

Praça da estação

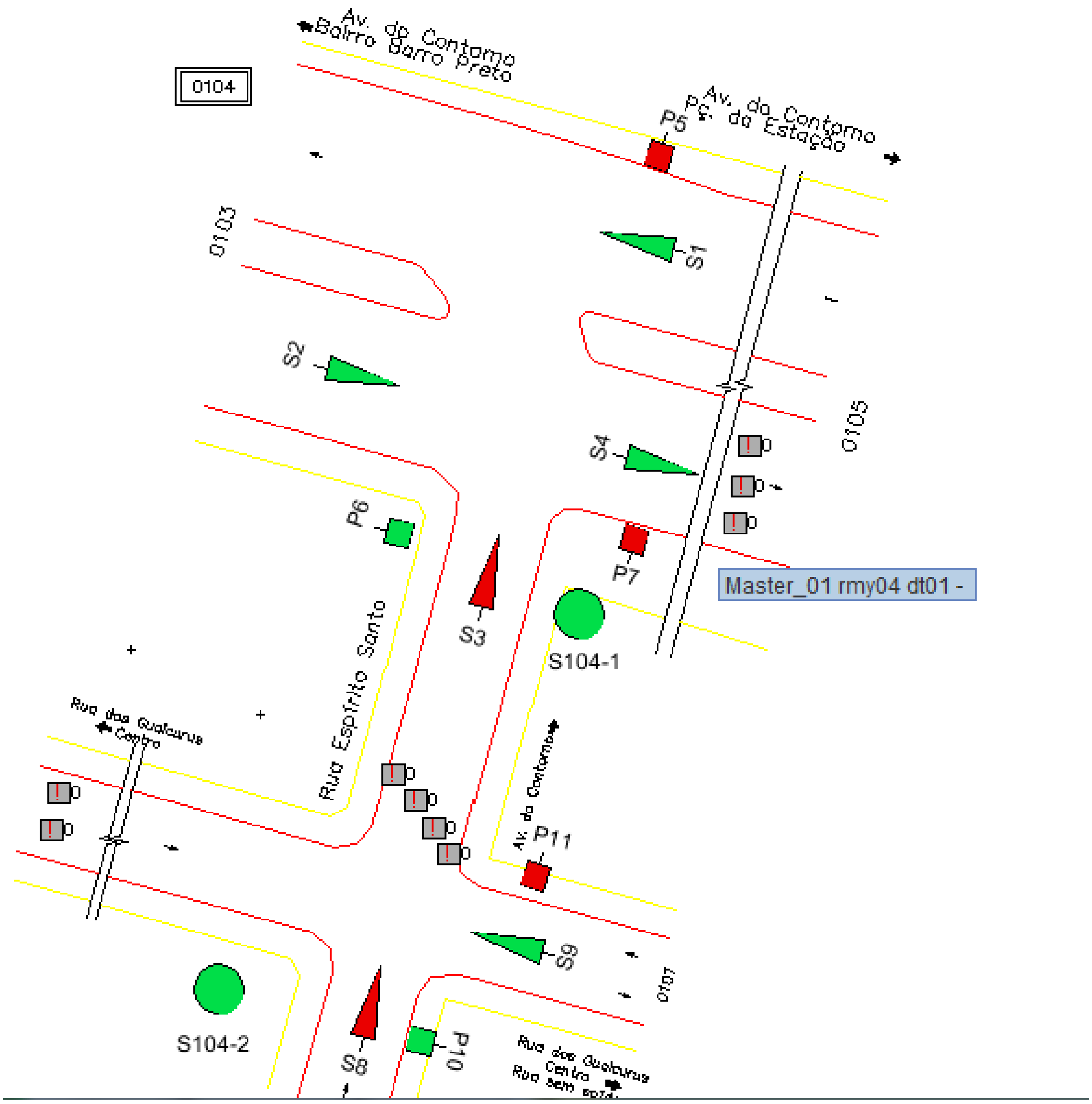

S3:4-7, S9:8-9

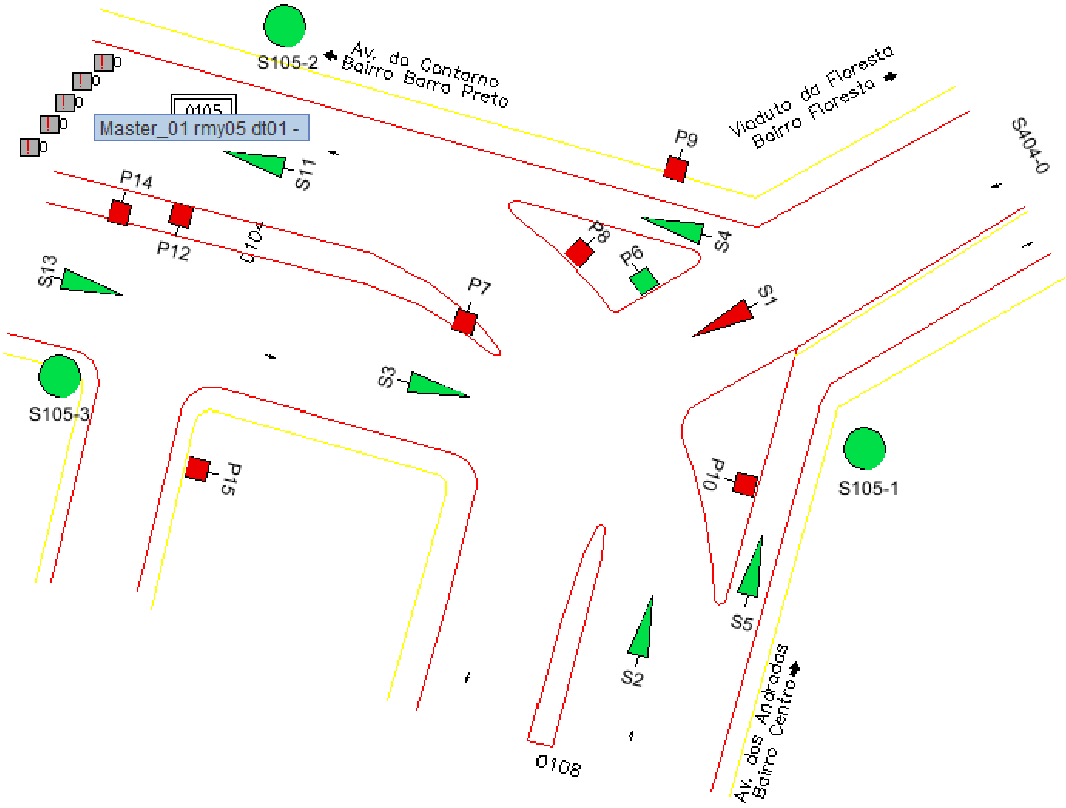

S105-2
Av. do Contorno
Bairro Barro Preto
0105
Master_01 rmy05 dt01 -
Viaduto da Floresta
Bairro Floresta
S404-0
P9
P14
S11
P12
0104
P8
S4
P6
S13
P7
S1
S3
S105-3
P15
P10
S105-1
S5
S2
0108
Av. dos Andradas
Bairro Centro

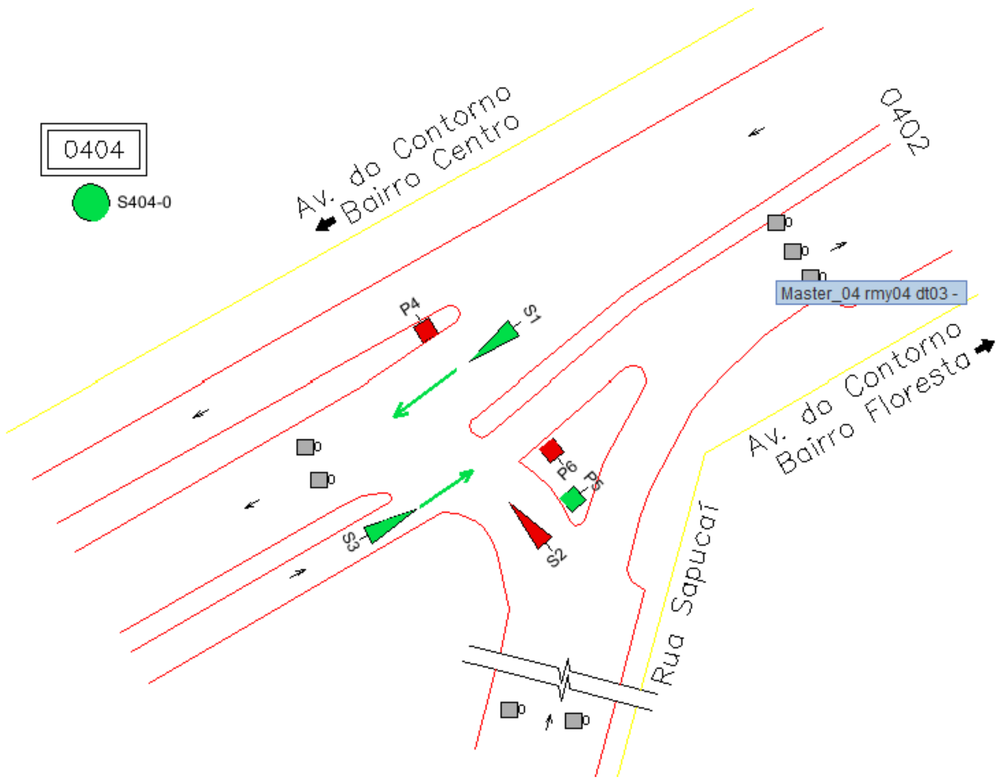
0404
S404-0
Av. do Contorno
Bairro Centro
0402
Master_04 rmy04 dt03 -
Av. do Contorno
Bairro Floresta
Rua Sapucaí
P4
S1
S3
S2
P6
P5

S2:6-7, S1:1-2

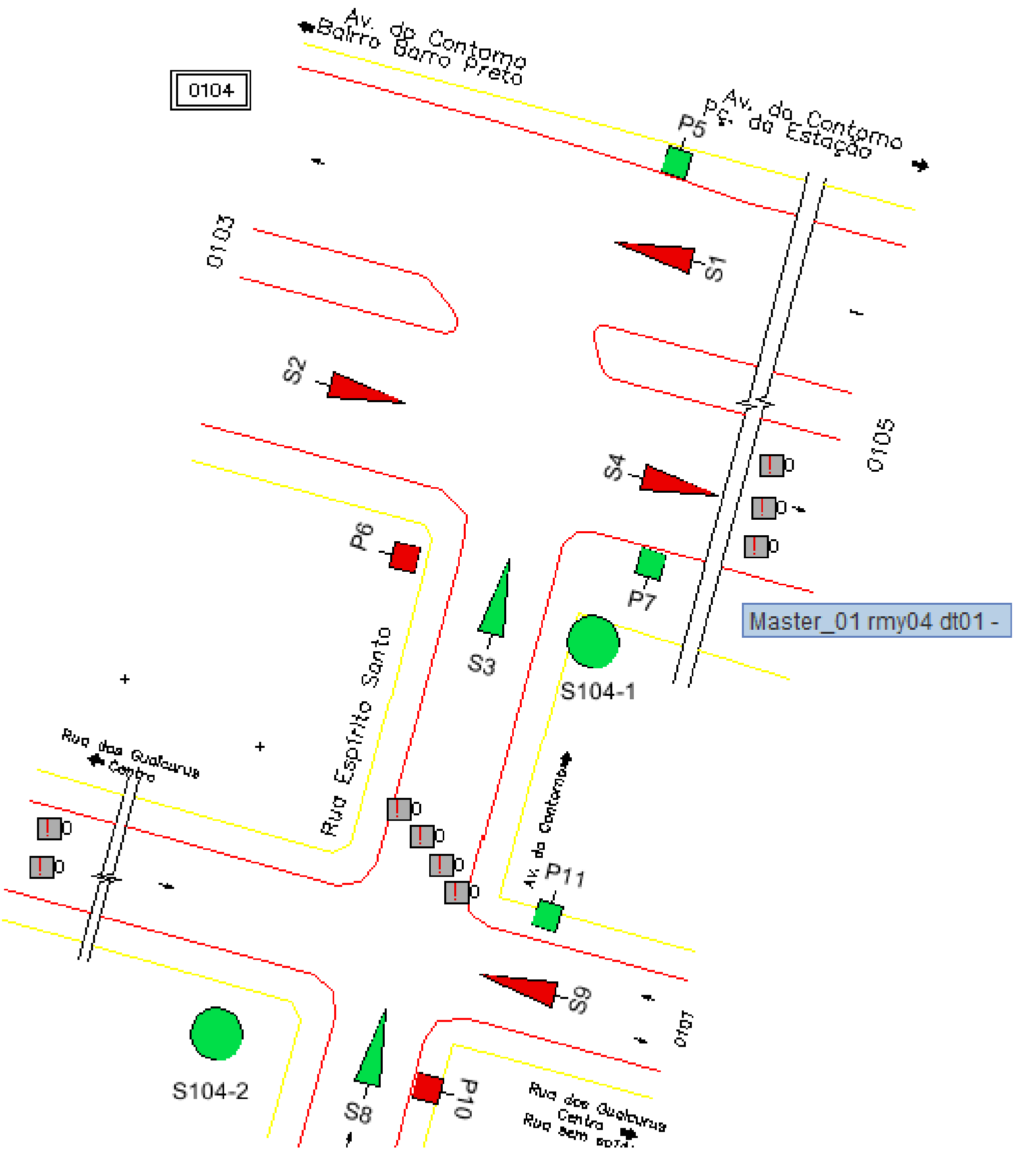

0104
Av. do Contorno
Bairro Barro Preto
Av. do Contorno
Pç. da Estação
P5
0103
S1
S2
0105
S4
P6
P7
S3
Master_01 rmy04 dt01 -
S104-1
Rua Espírito Santo
Rua dos Guaicurus
Centro
Av. do Contorno
P11
S9
0107
S104-2
S8
P10

S3:4-7, S9:8-9

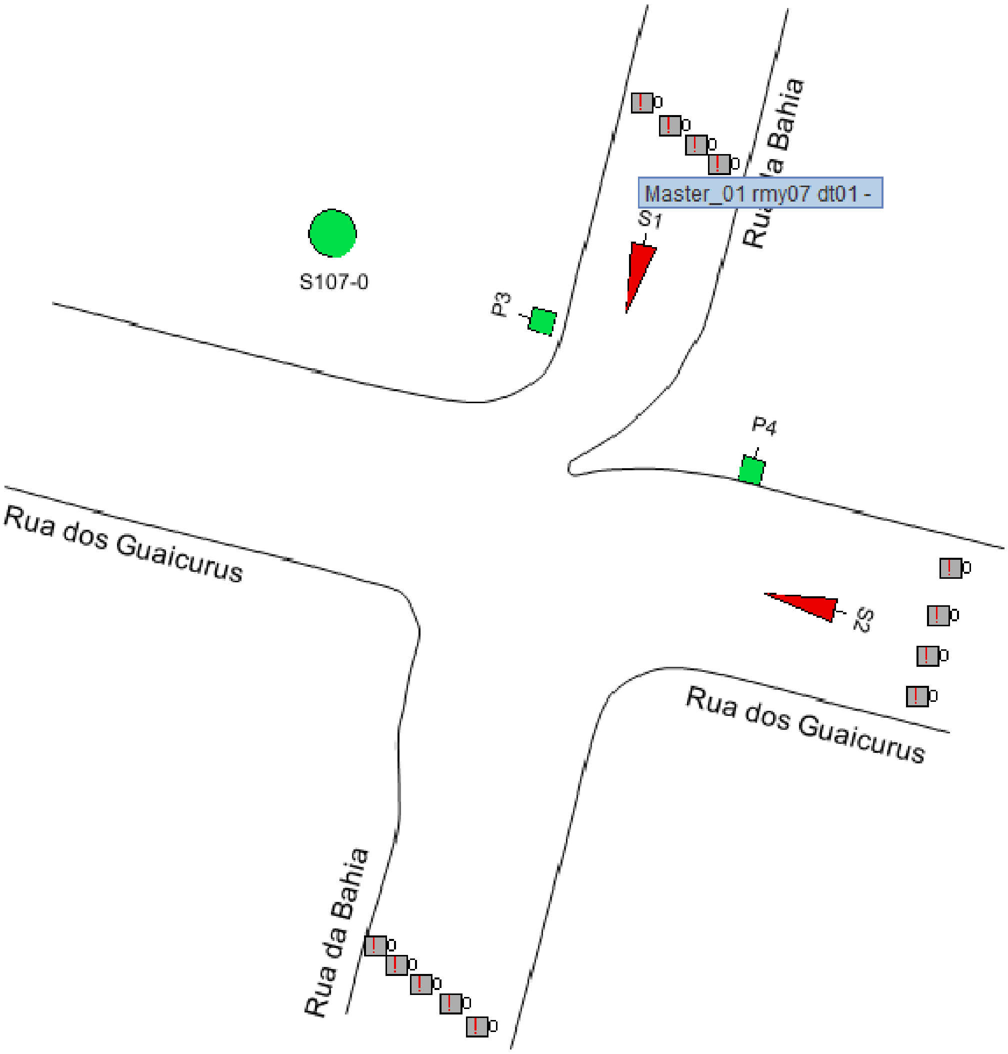
Ruas da Bahia
Master_01 rmy07 dt01 -
S1
S107-0
P3
P4
Rua dos Guaicurus
S2
Rua dos Guaicurus
Rua da Bahia

S2:5-8, S2d:9-13

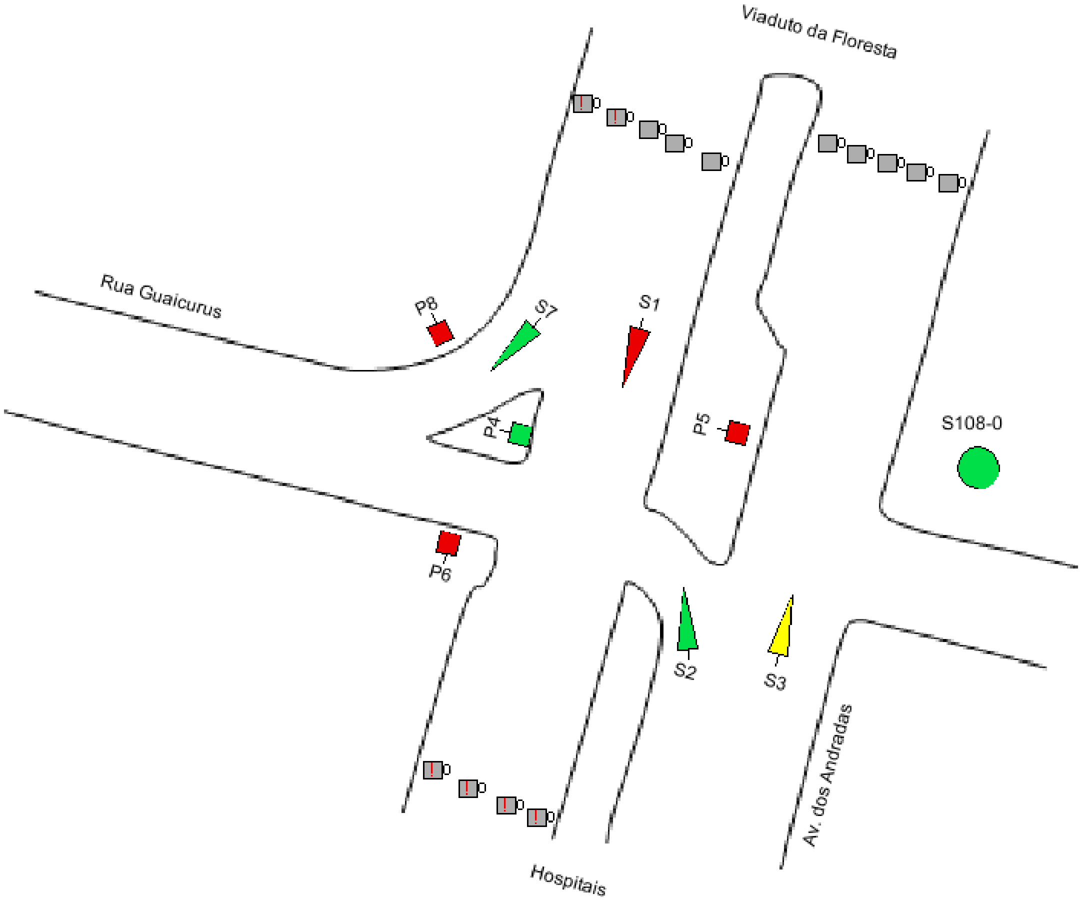

Viaduto da Floresta
Rua Guaicurus
P8
S7
S1
P4
P5
S108-0
P6
S2
S3
Av. dos Andradas
Hospitais

S1:11-14, s1-:1-5

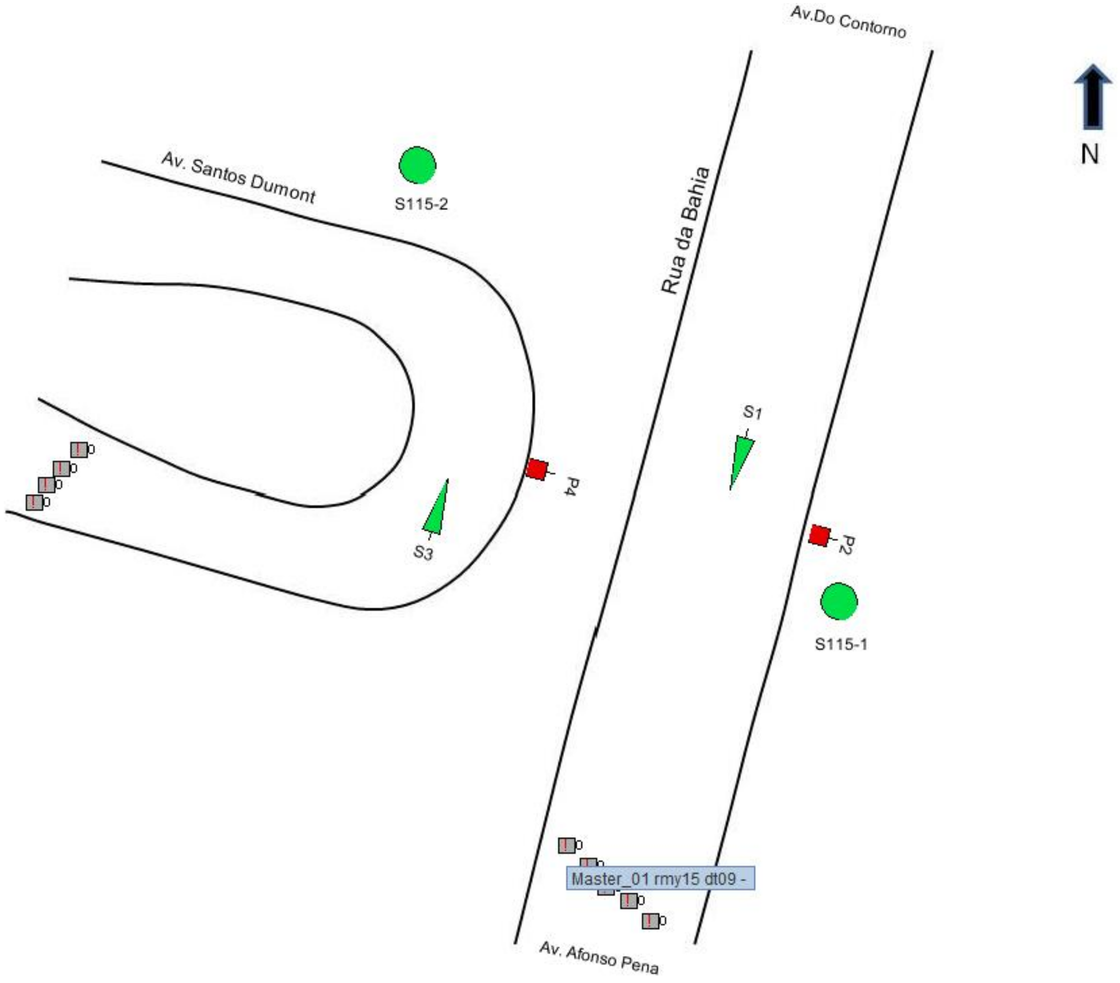

Av.Do Contorno
N
Av. Santos Dumont
S115-2
Rua da Bahia
S1
P4
S3
P2
S115-1
Master_01 rmy15 dt09 -
Av. Afonso Pena

S3:1-4

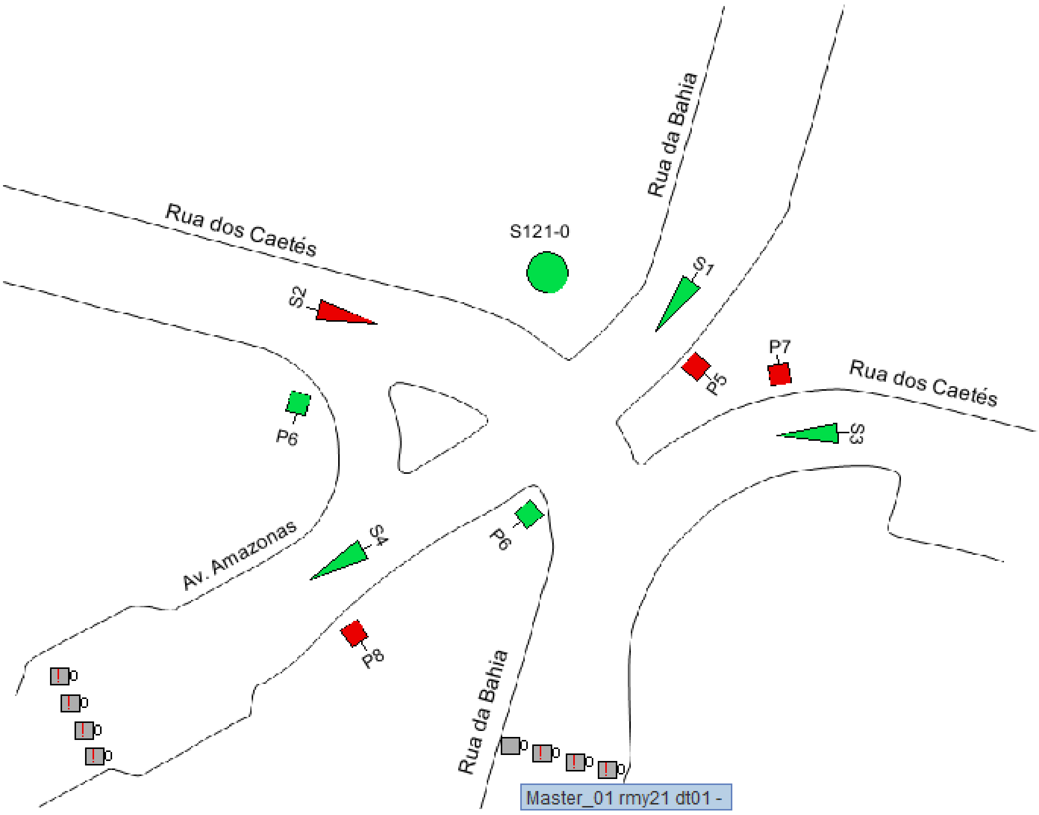
Rua da Bahia
Rua dos Caetés
S121-0
S1
S2
P7
P5
Rua dos Caetés
P6
S3
Av. Amazonas
S4
P6
P8
Rua da Bahia
Master_01 rmy21 dt01 -

S4:5-8

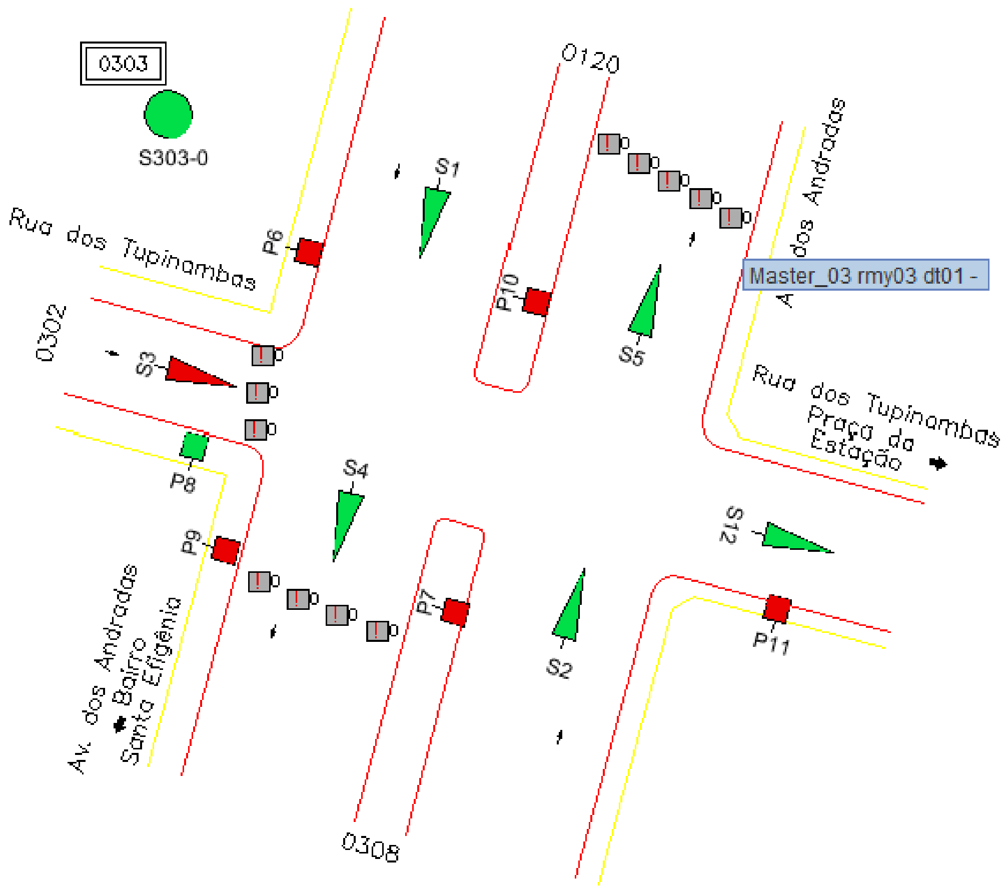
0303
S303-0
Rua dos Tupinambas
0302
S3
P6
S1
0120
P10
S5
Master_03 rmy03 dt01 -
Av. dos Andradas
Rua dos Tupinambas
Praça da Estação
P8
S4
P9
S12
P7
S2
P11
Av. dos Andradas
Bairro Santa Efigênia
0308

S4:6-9, S3:10-12

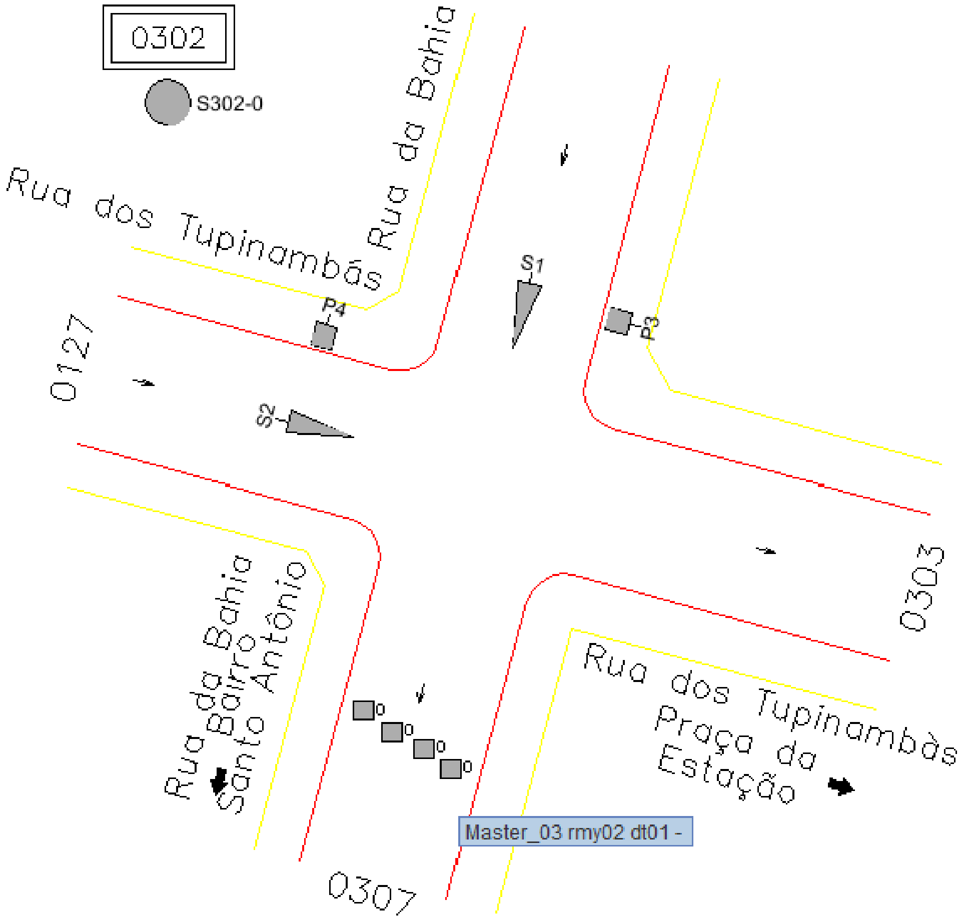
0302
S302-0
Rua dos Tupinambás
Rua da Bahia
P4
S1
P3
0127
S2
0303
Rua da Bahia
Bairro
Santo Antônio
Rua dos Tupinambàs
Praça da
Estação
Master_03 rmy02 dt01 -
0307

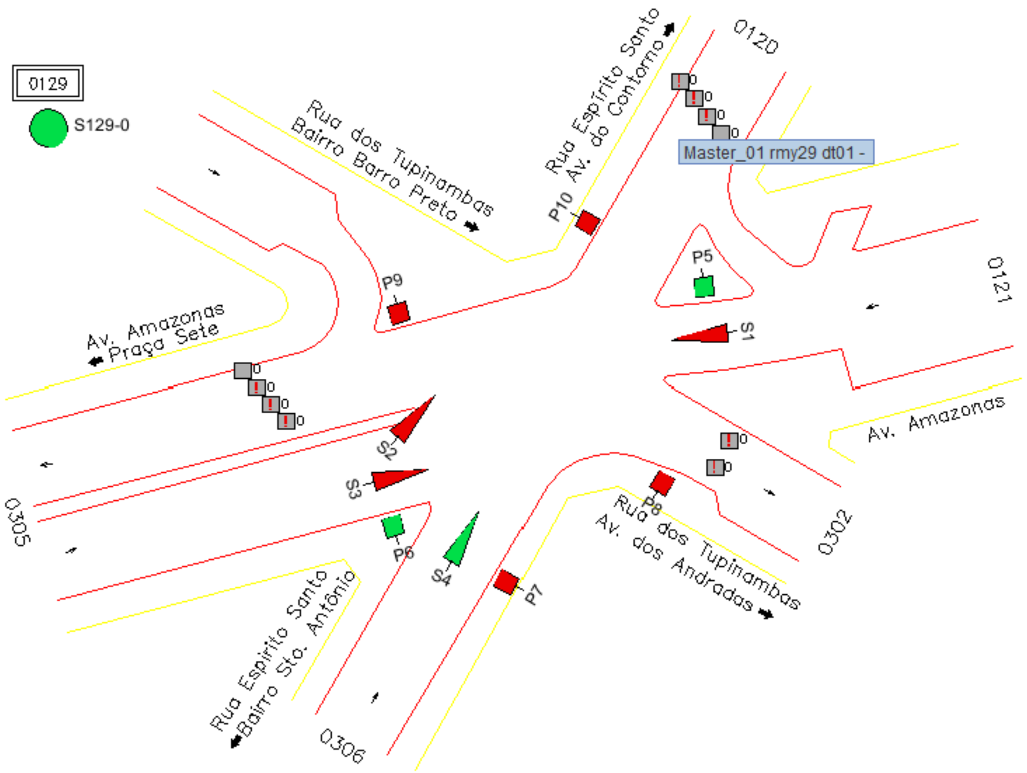

0129
S129-0
Rua dos Tupinambas
Bairro Barro Preto
Rua Espírito Santo
Av. do Contorno
0120
Master_01 rmy29 dt01 -
P10
P9
P5
0121
S1
Av. Amazonas
Praça Sete
S2
S3
Av. Amazonas
0305
P6
S4
P7
P8
Rua dos Tupinambas
Av. dos Andradas
0302
Rua Espírito Santo
Bairro Sto. Antônio
0306

S3:5-6, S1:7-10

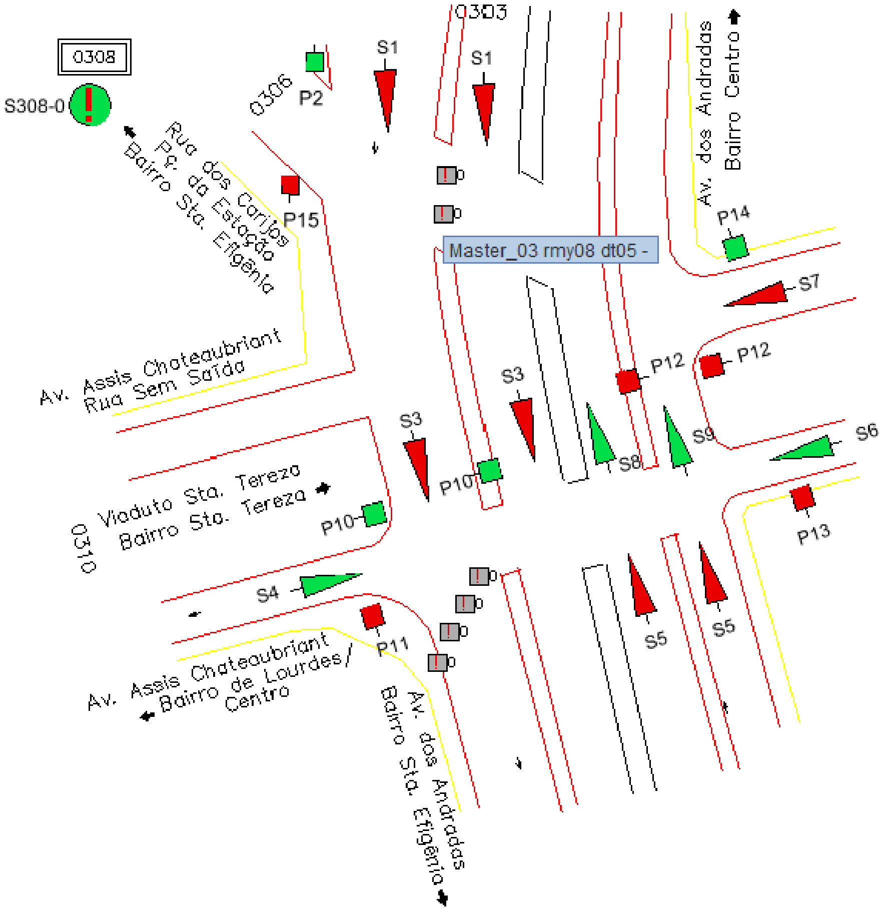
0308
S308-0
0306
P2
S1
S1
0303
Rua dos Carijós
Pç. da Estação
Bairro Sta. Efigênia
P15
Master_03 rmy08 dt05 -
Av. dos Andradas
Bairro Centro
P14
S7
Av. Assis Chateaubriant
Rua Sem Saída
S3
S3
P12
P12
S9
S8
S6
Viaduto Sta. Tereza
Bairro Sta. Tereza
0310
P10
P10
P13
S4
P11
S5
S5
Av. Assis Chateaubriant
Bairro de Lourdes/
Centro
Av. dos Andradas
Bairro Sta. Efigênia

S3:1-4

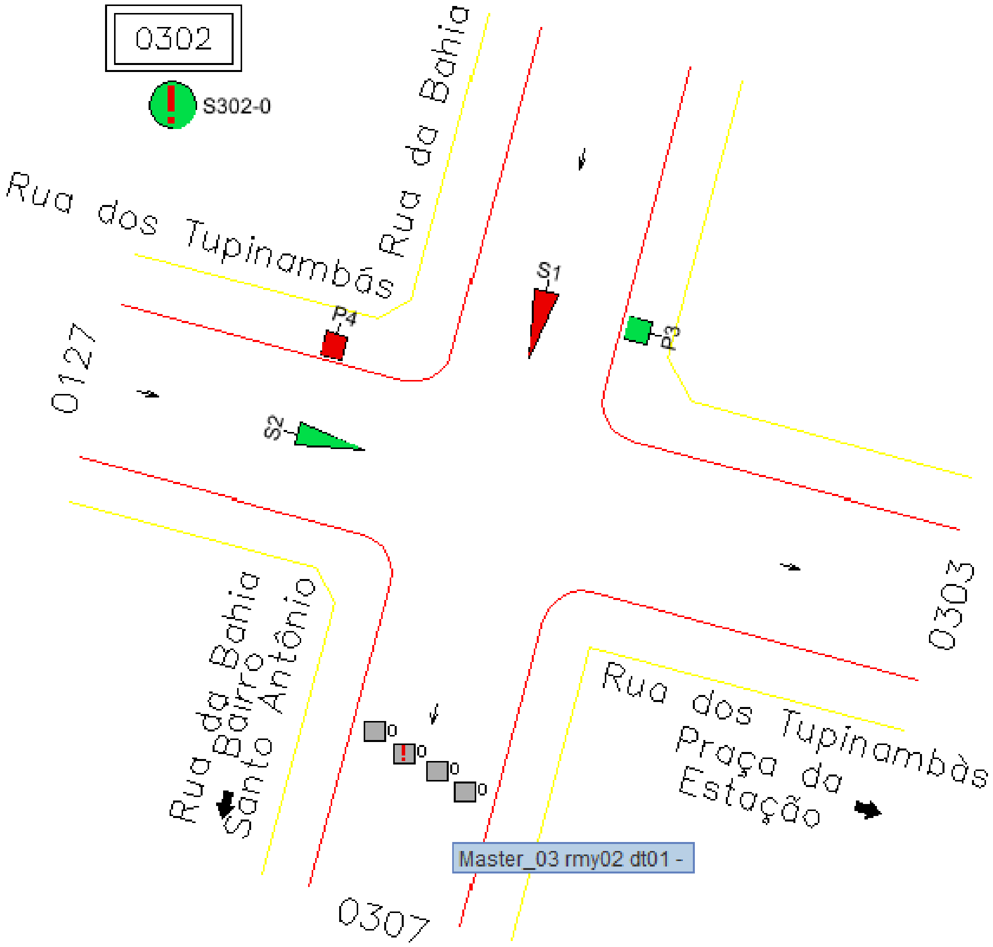
0302
S302-0
Rua dos Tupinambás
Rua da Bahia
S1
P4
P3
0127
S2
0303
Rua da Bahia
Bairro
Santo Antônio
Rua dos Tupinambàs
Praça da
Estação
Master_03 rmy02 dt01 -
0307

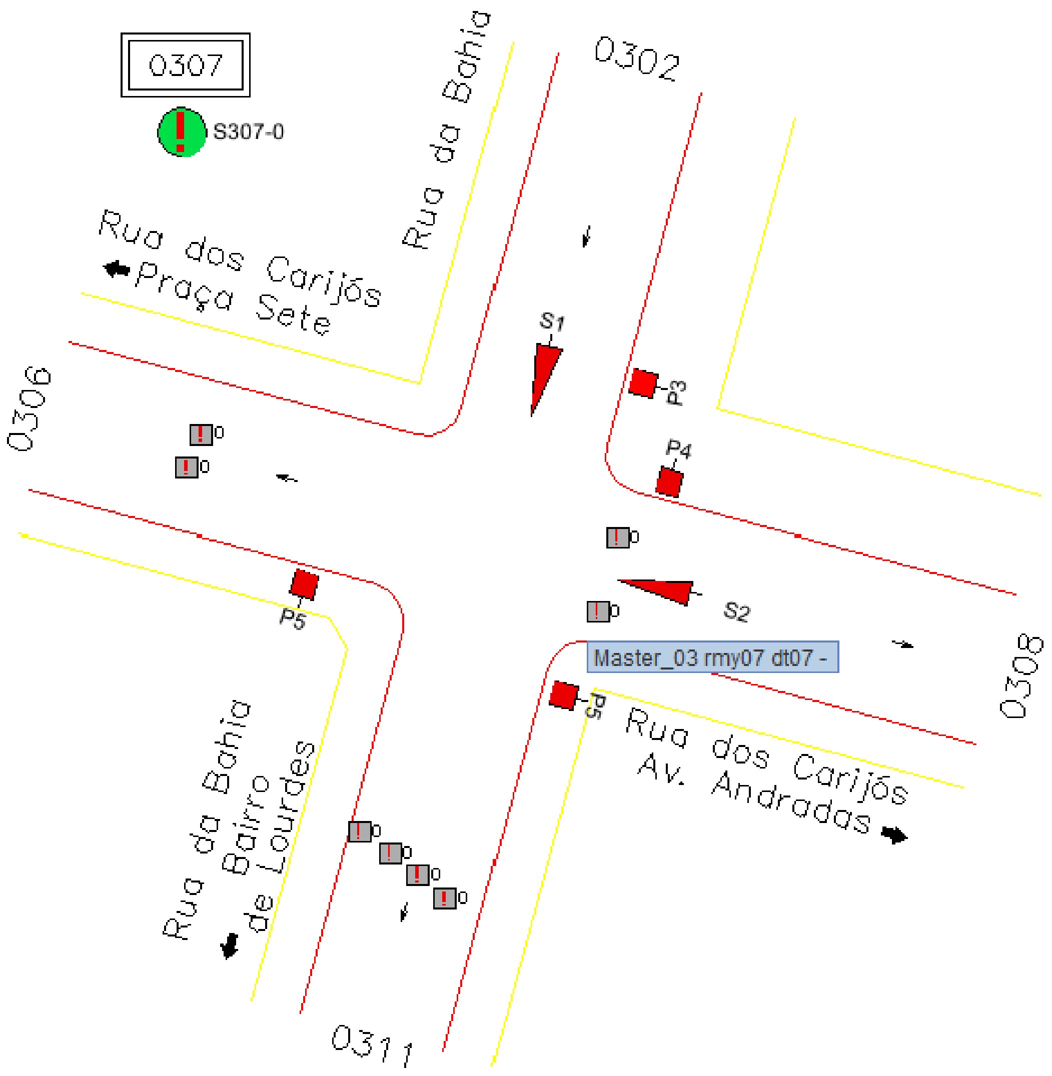

0307
S307-0
Rua da Bahia
0302
Rua dos Carijós
Praça Sete
S1
P3
P4
0306
P5
S2
Master_03 rmy07 dt07 -
0308
P5
Rua dos Carijós
Av. Andradas
Rua da Bahia
Bairro de Lourdes
0311

S1:1-4, S2:5-6

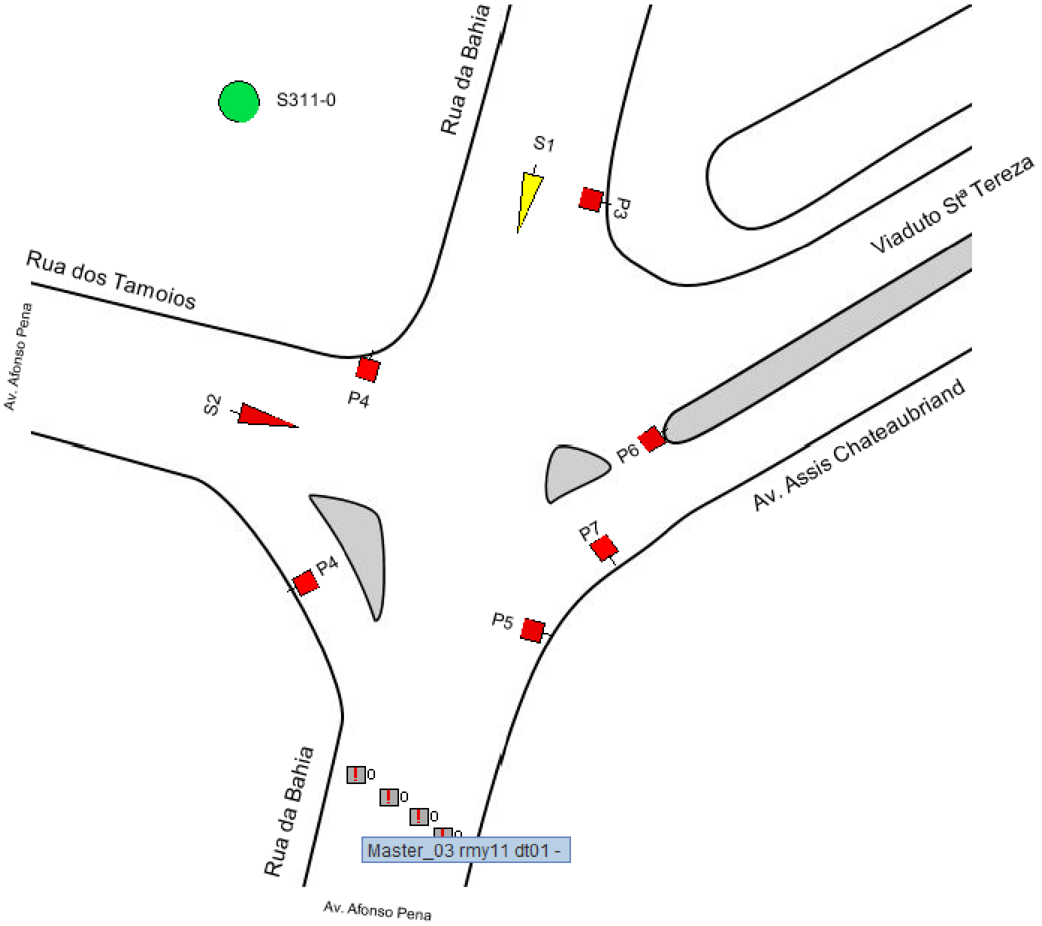
S311-0
Rua da Bahia
S1
P3
Viaduto Stª Tereza
Rua dos Tamoios
Av. Afonso Pena
P4
S2
P6
Av. Assis Chateaubriand
P7
P4
P5
Rua da Bahia
Master_03 rmy11 dt01 -
Av. Afonso Pena
