# Supplementary material for: Traffic light optimization using non-dominated sorting genetic algorithm (NSGA2)
Source: Sci Rep. 2023 Sep 20;13:15550. doi: 10.1038/s41598-023-38884-2 (PMC10511403; doi:10.1038/s41598-023-38884-2)

Table of Contents

[Praça Raul Soares 2](#__RefHeading___Toc265_2514567647)

[Praça Sete 28](#__RefHeading___Toc267_2514567647)

[Savassi 51](#__RefHeading___Toc269_2514567647)

[Praça da estação 70](#__RefHeading___Toc271_2514567647)


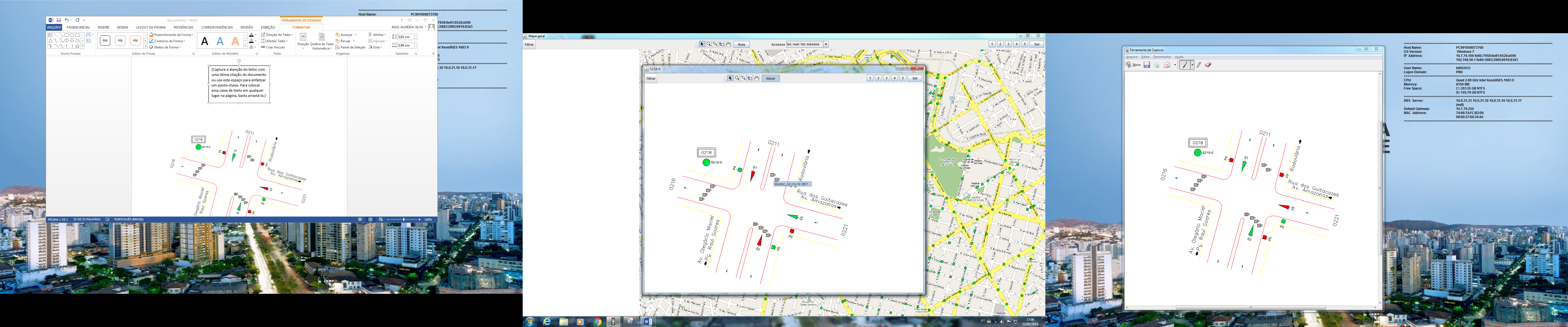


S2:3-6

S3:7-10

# Praça Raul Soares

S4:1-3, S3:4-6
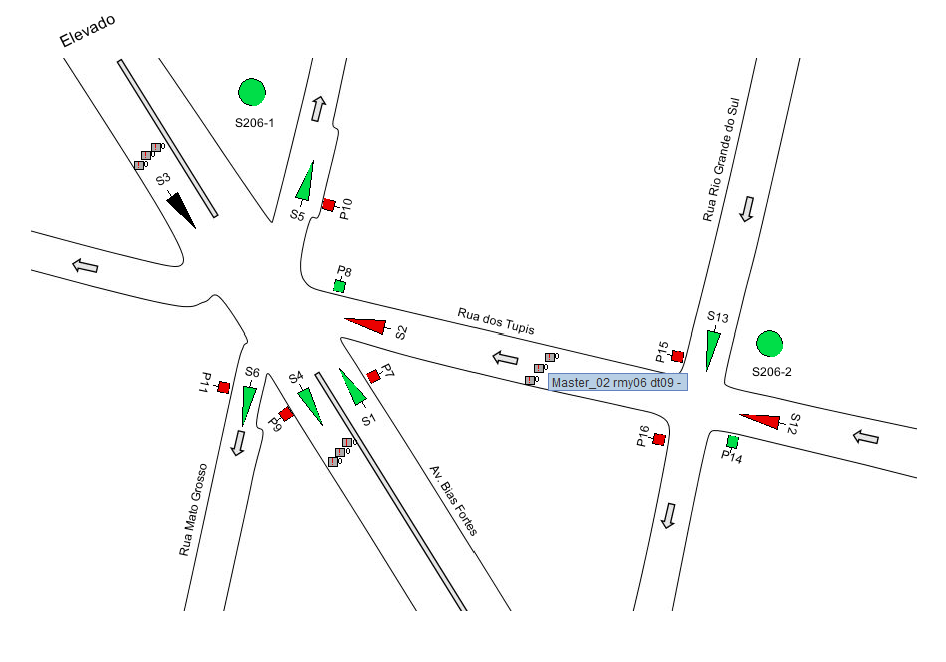


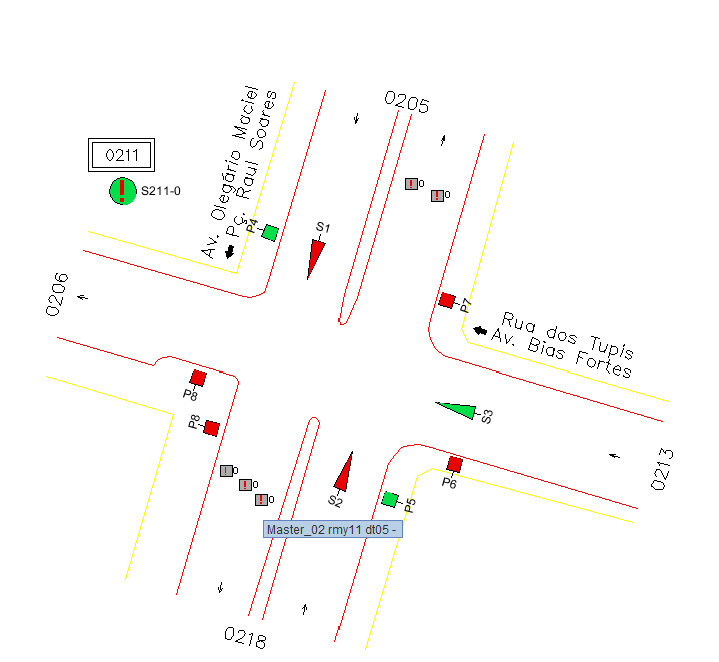


S1:3-5, S2:1-2


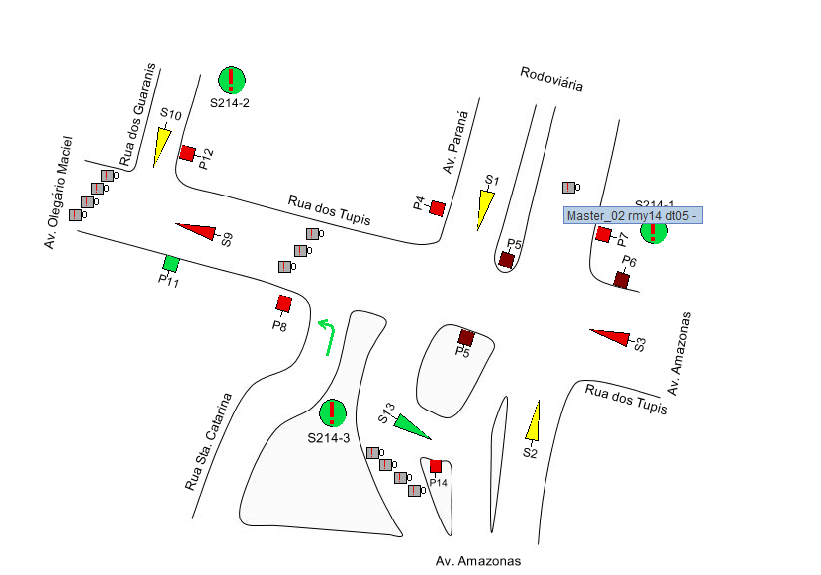


S13:1-4, S9:6-8, S10:9-12, S2:5


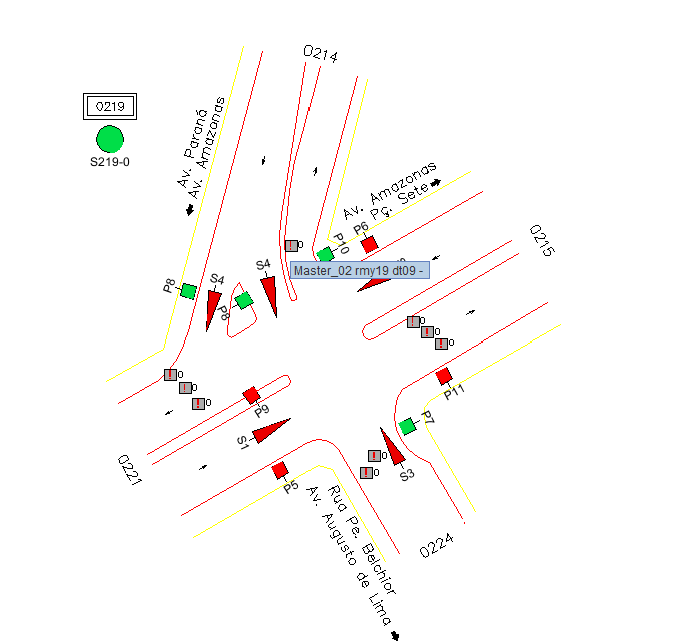


S1:1-3, S3:4-5, S2:6-8,


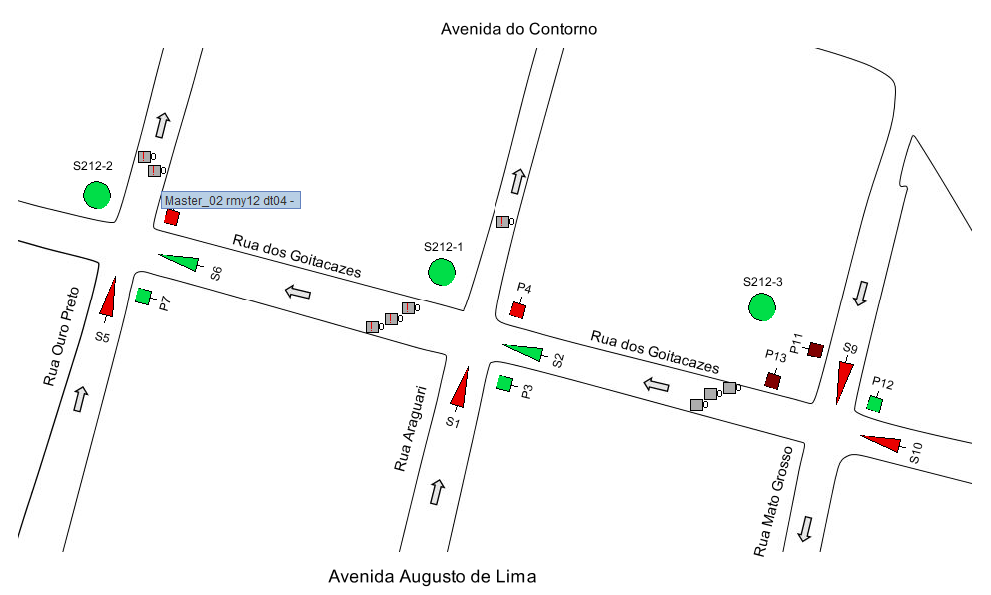


S1:6, S2:6-8 S6:1-3


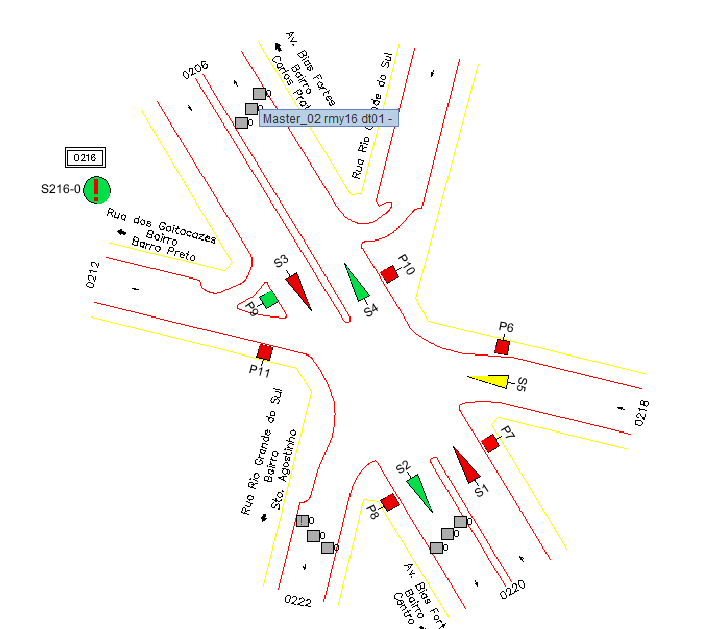


S2:4-6, S5:7-9, S4:1-3


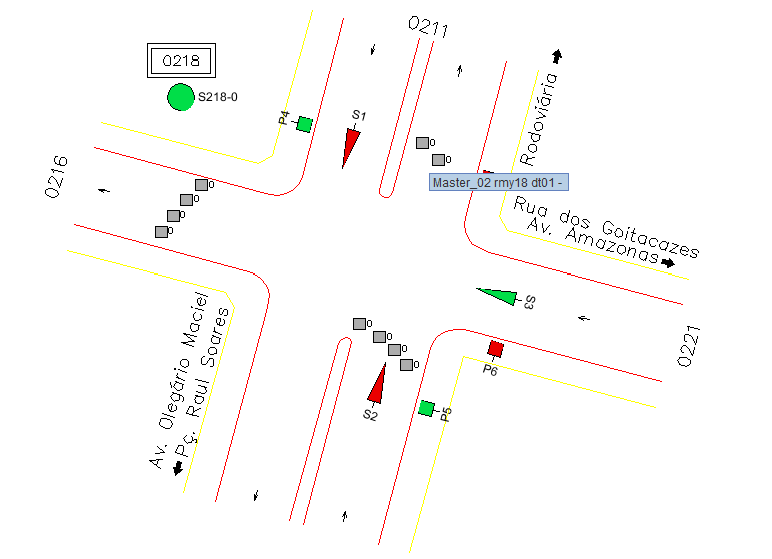


S2:3-6, S3:7-10


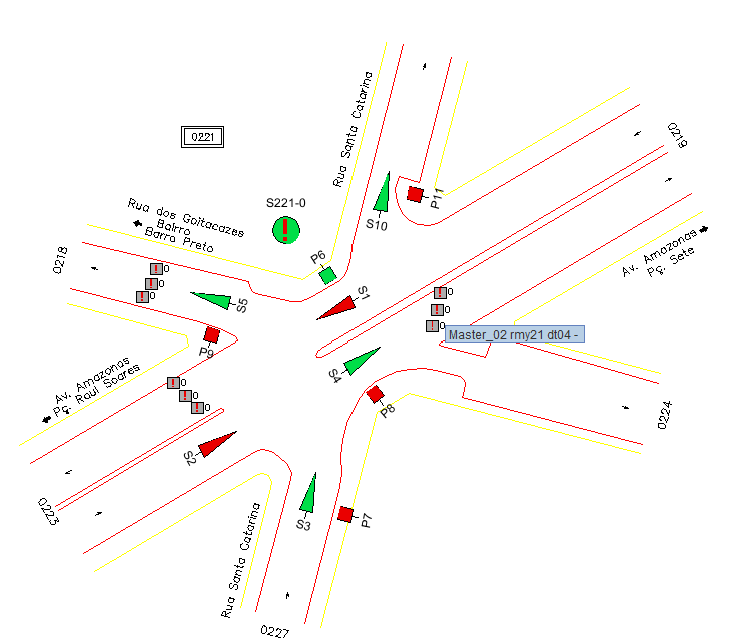


S1:7-9, S5:1-3


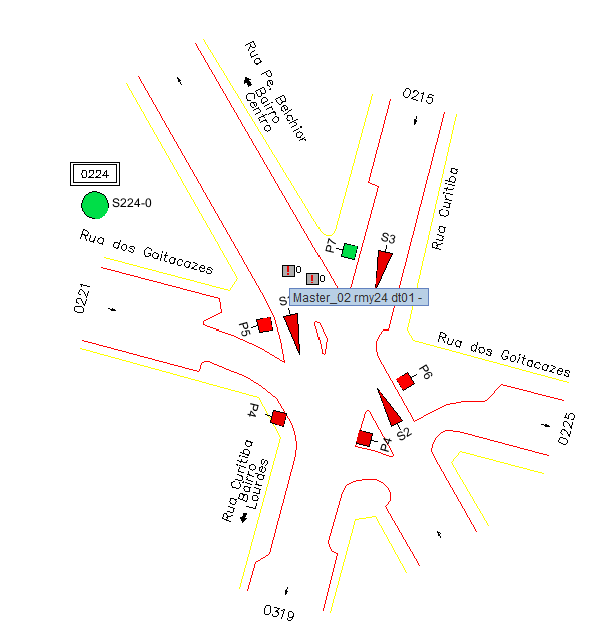


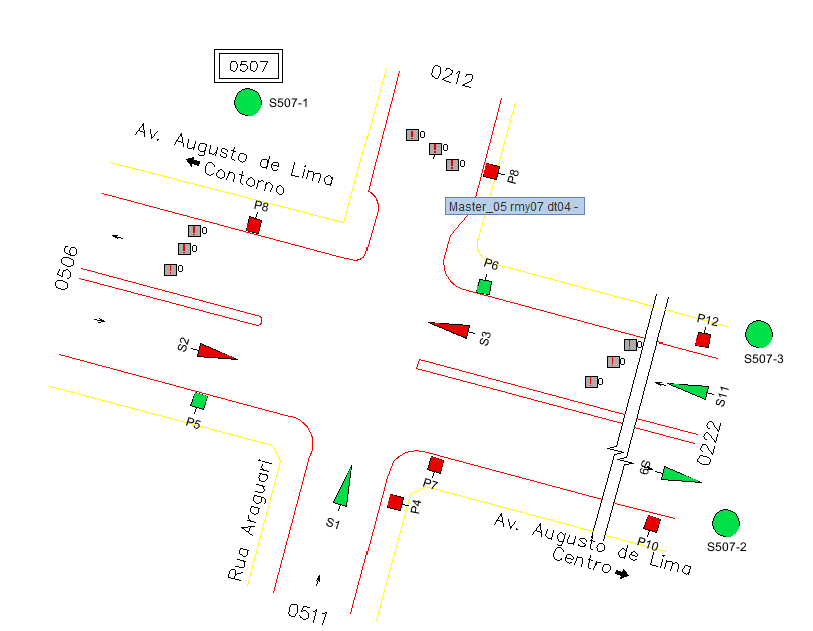


S11:7-9, S3:1-3


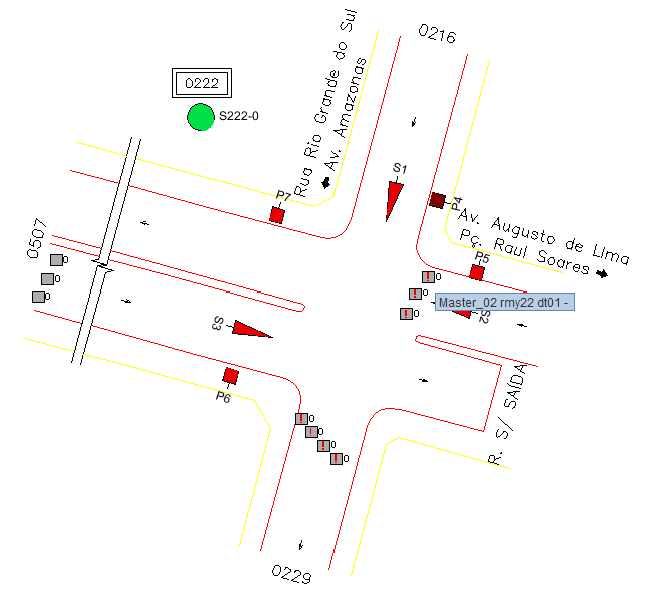


S1:4-7, S3:8-10


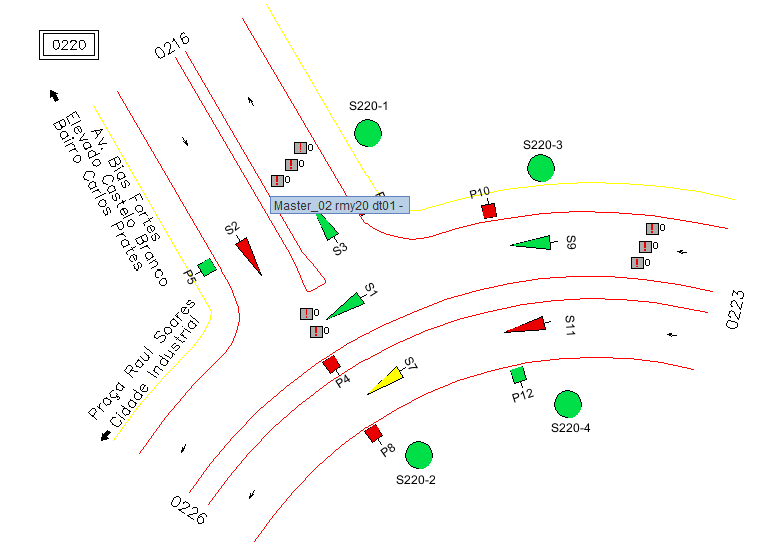


S9:4-6, S1:7-8


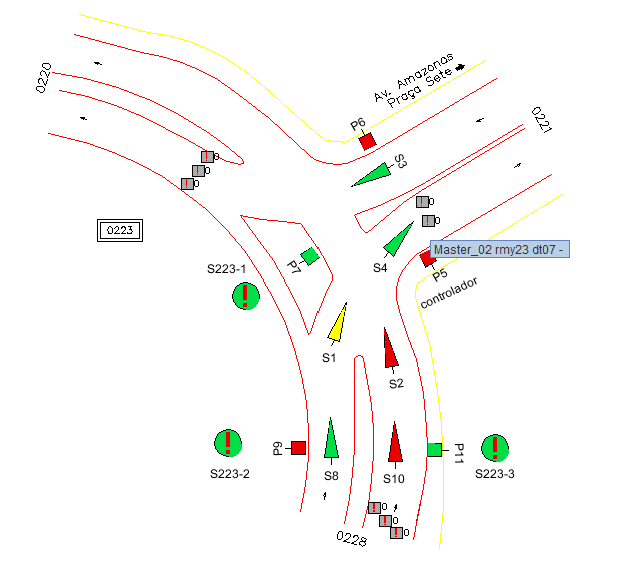


S10:1-3, S3:4-6


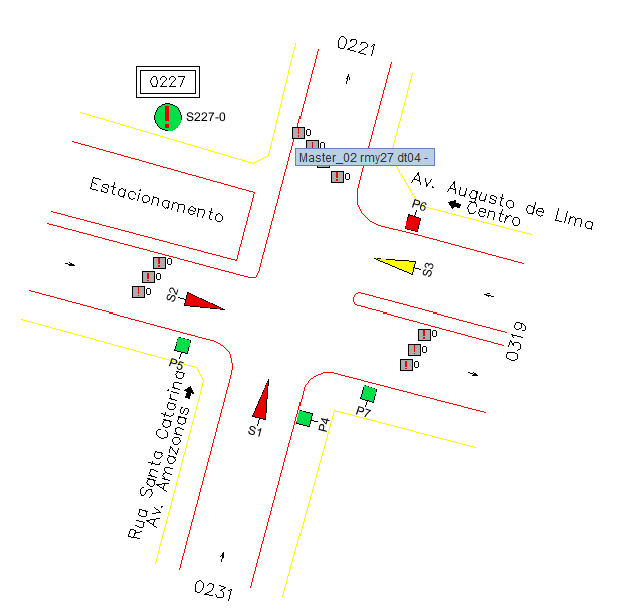


S2:8-10, S2:1-3,


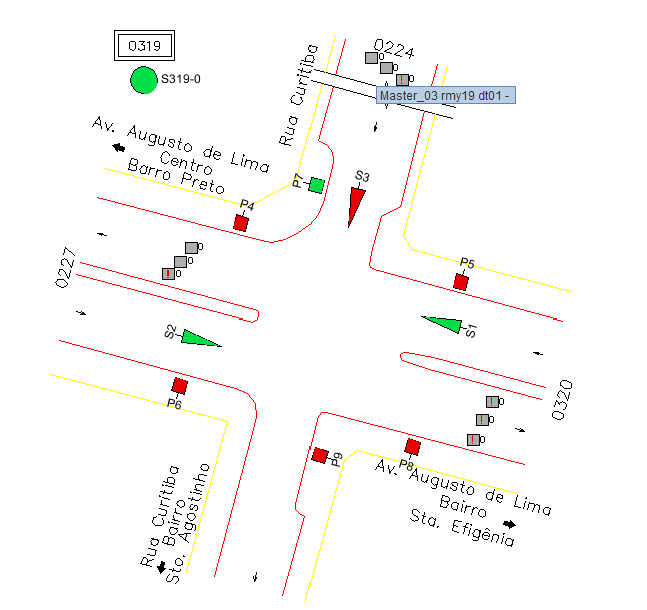


S2:4-6, S1:7-9,


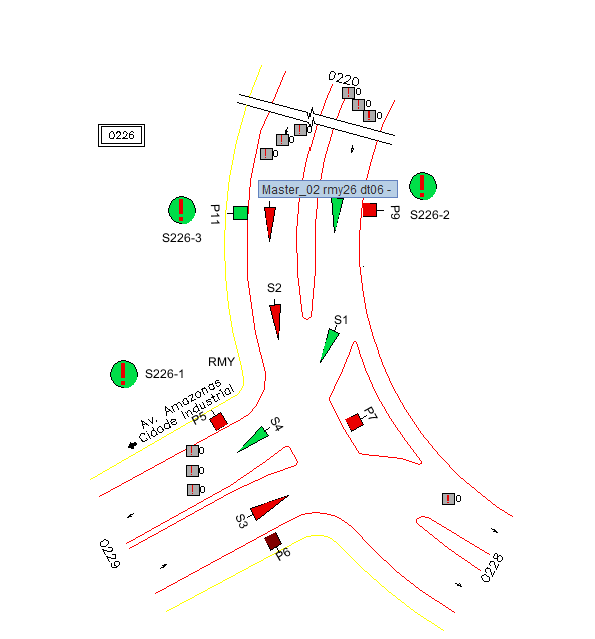


S8:1-3, S3:7, S4:8-10,


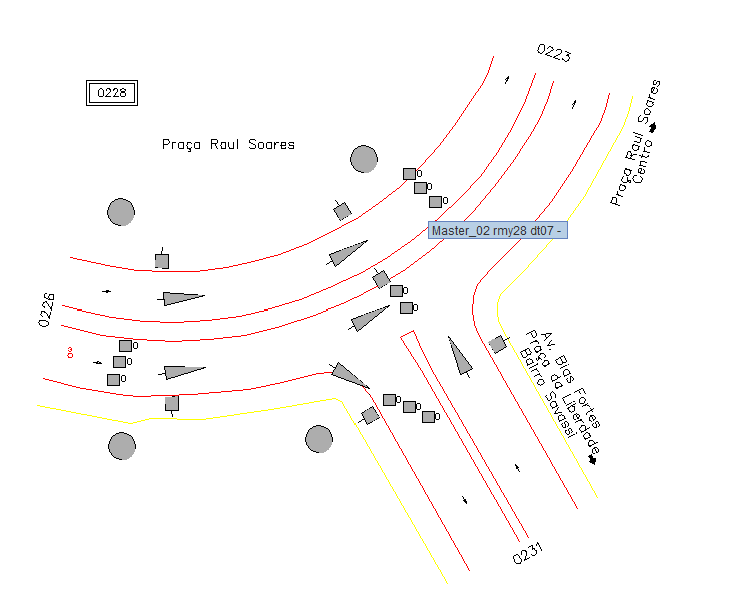


S2:10-11, S3:1-3, S9:4-6


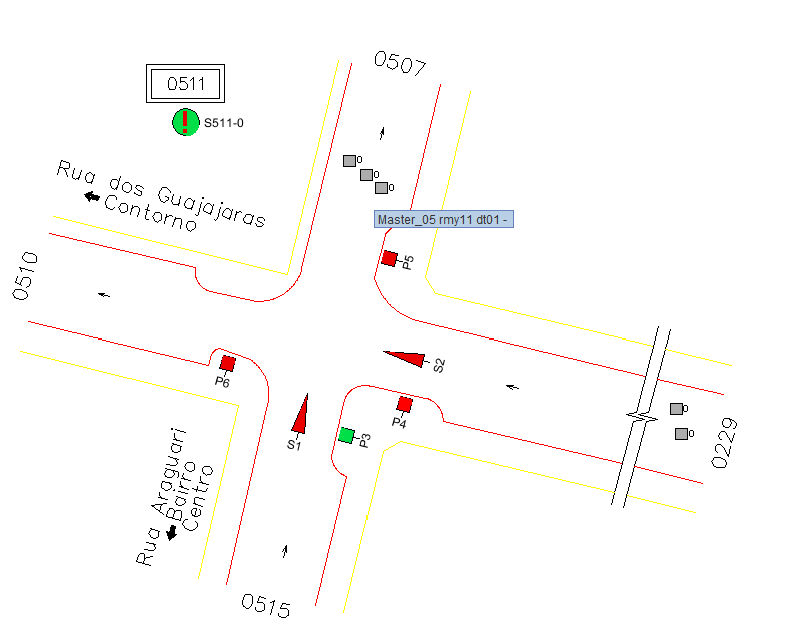


S2:4-5,


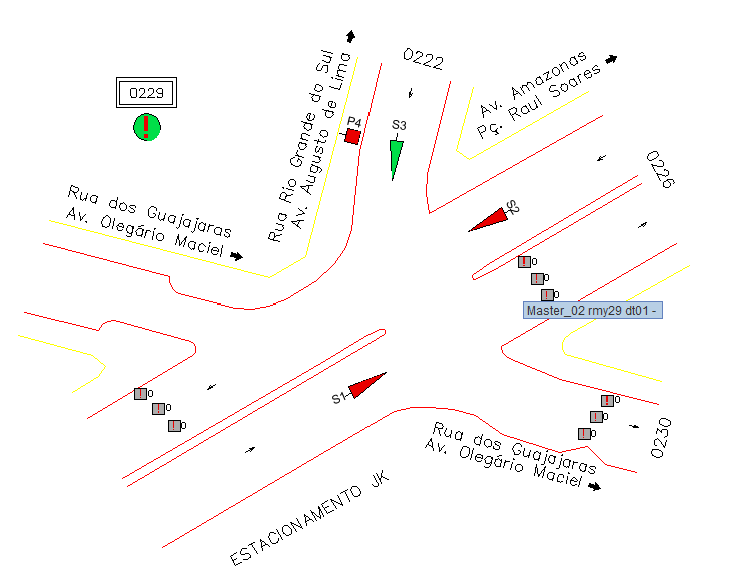


S3:4-6, S2:7-9


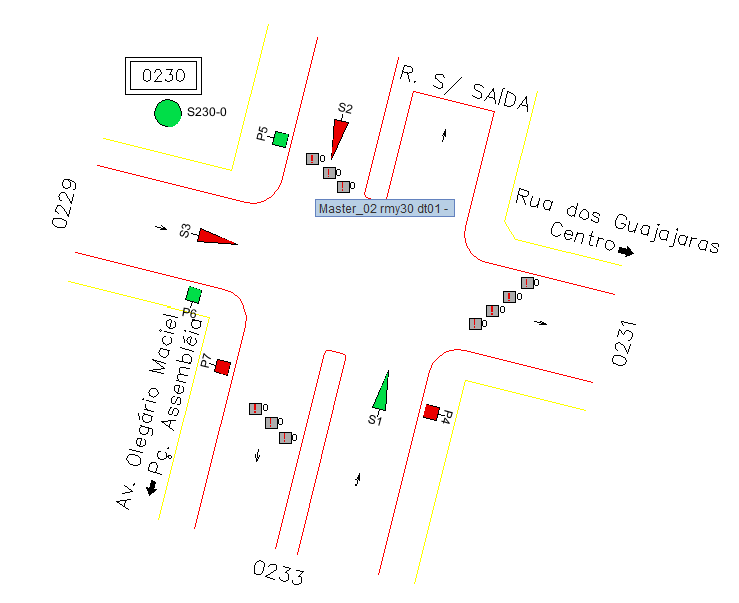


S3:4-7, S2:8-10


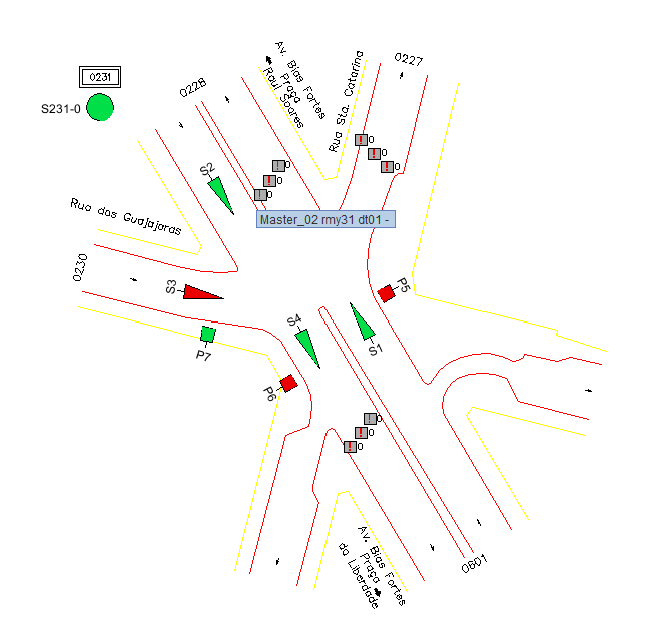


S3:4-6, S4:7-9, S1:1-3


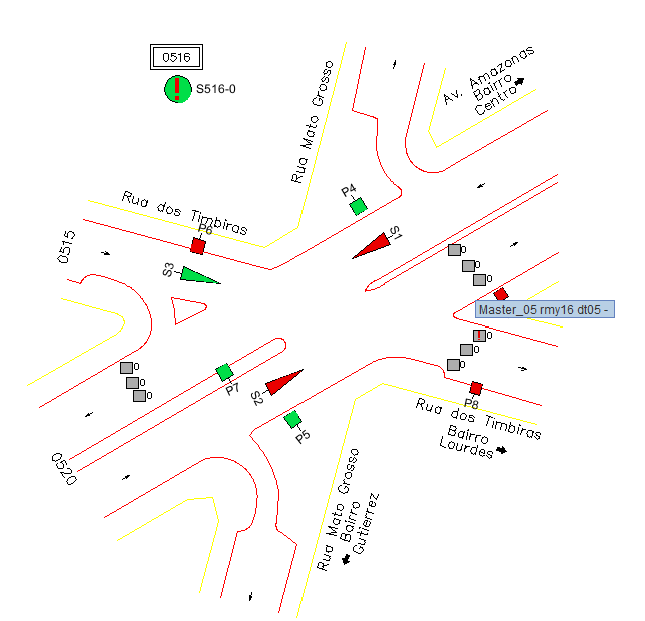


S3:7-9, S1:1-3


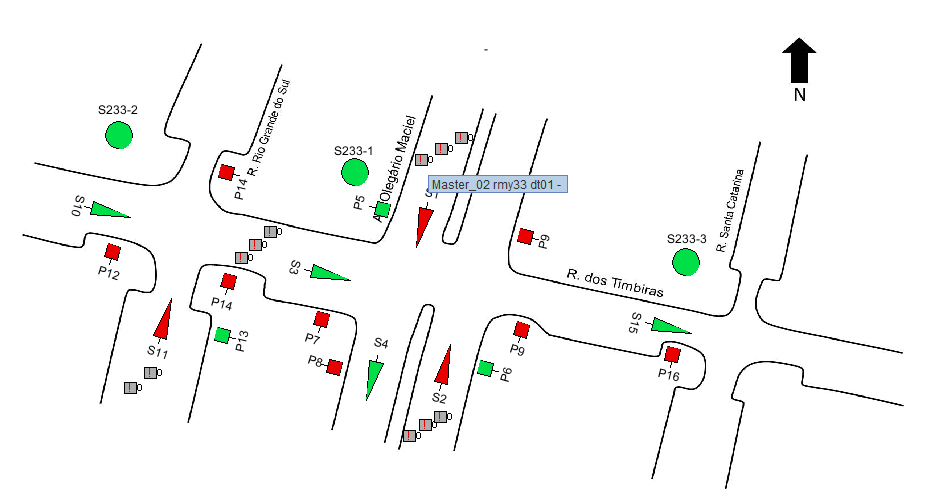


S2:4-6, S11:7-8, S3:9-11,


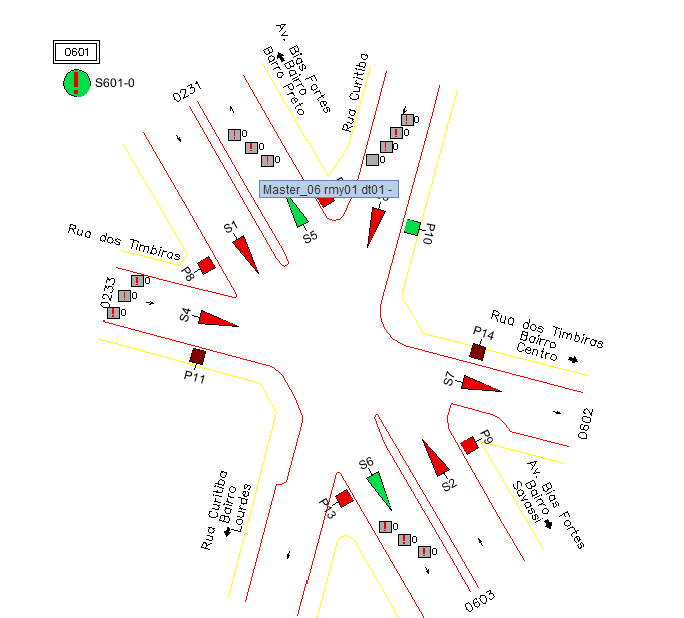


S3:10-13, S6:4-6, S4:7-9


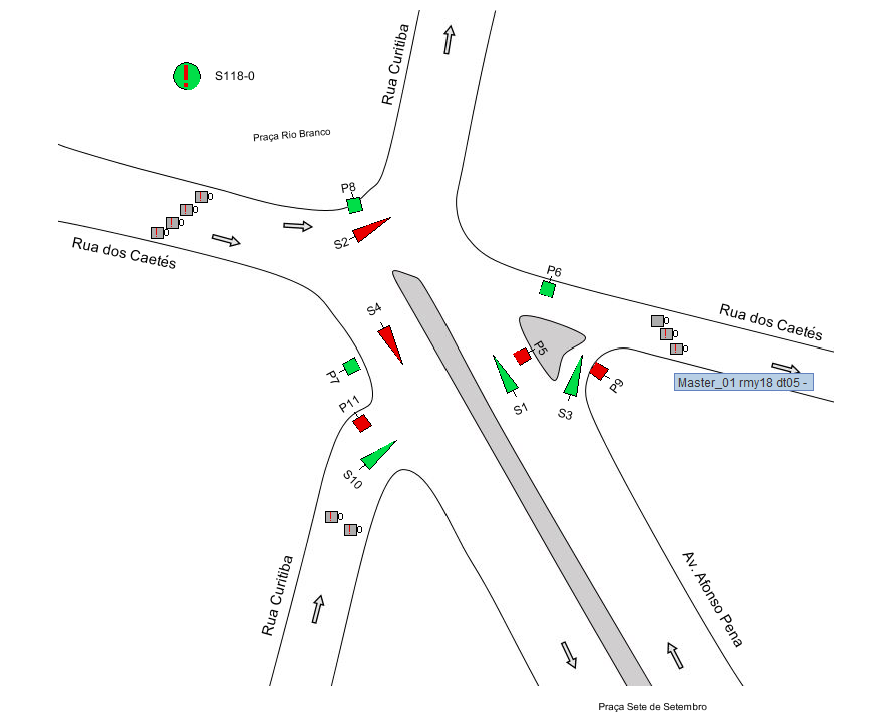


S10:8-9, S2:1-4,

# Praça Sete


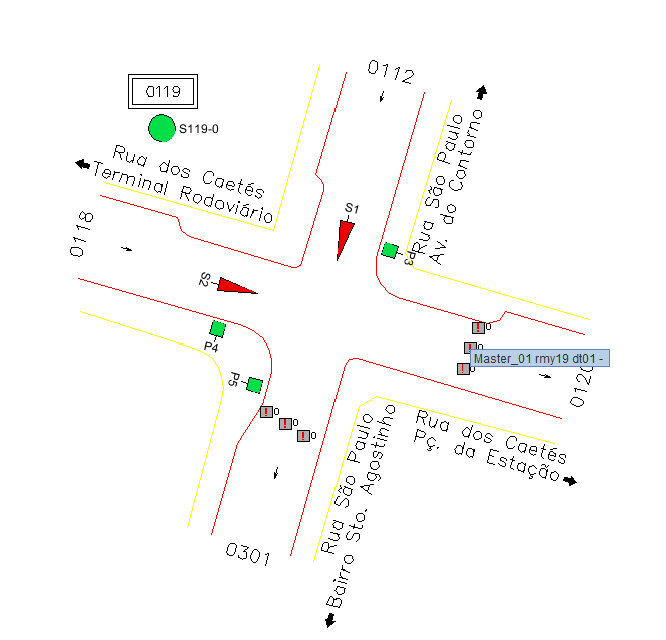


S1:4-6,


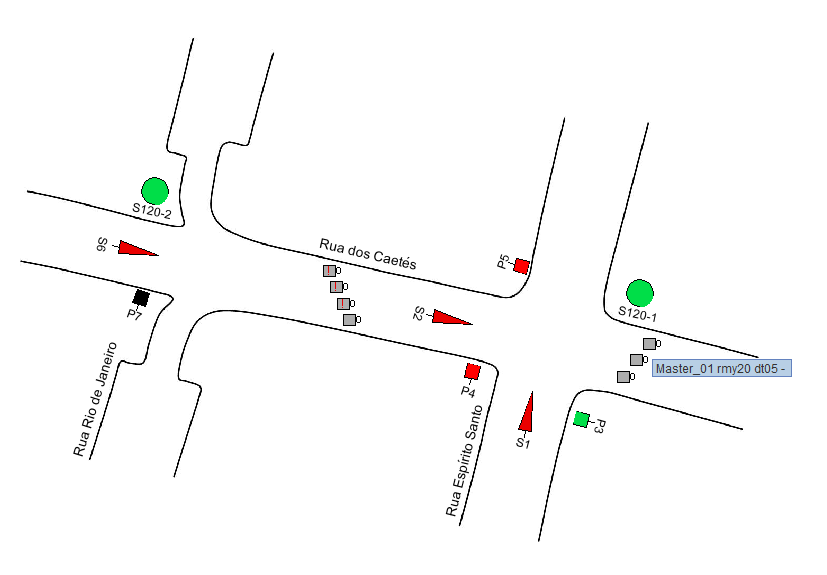


S2:1-4


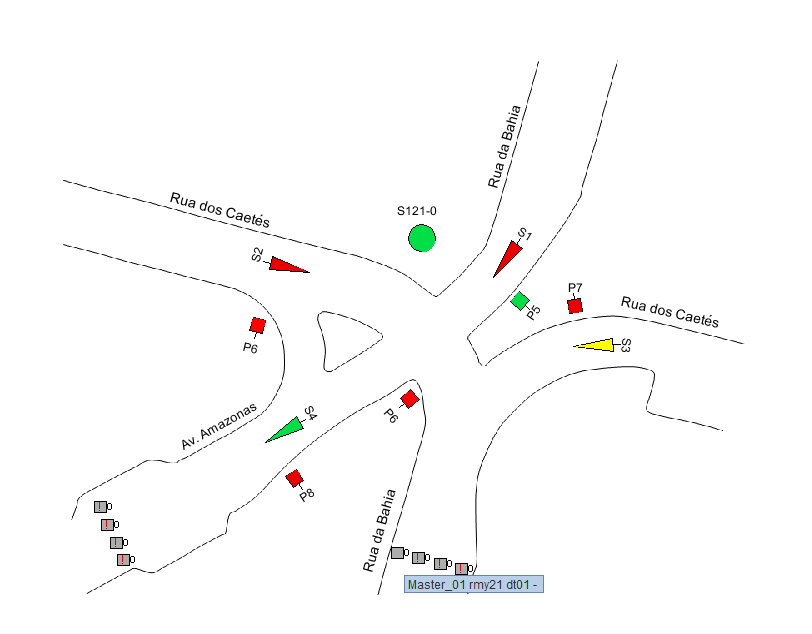


S4:5-8


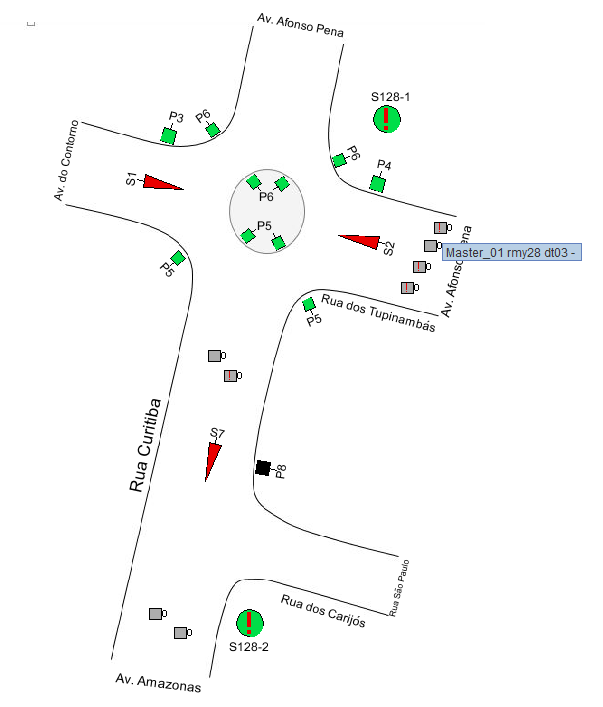


S7:7-8, S7-:1-2


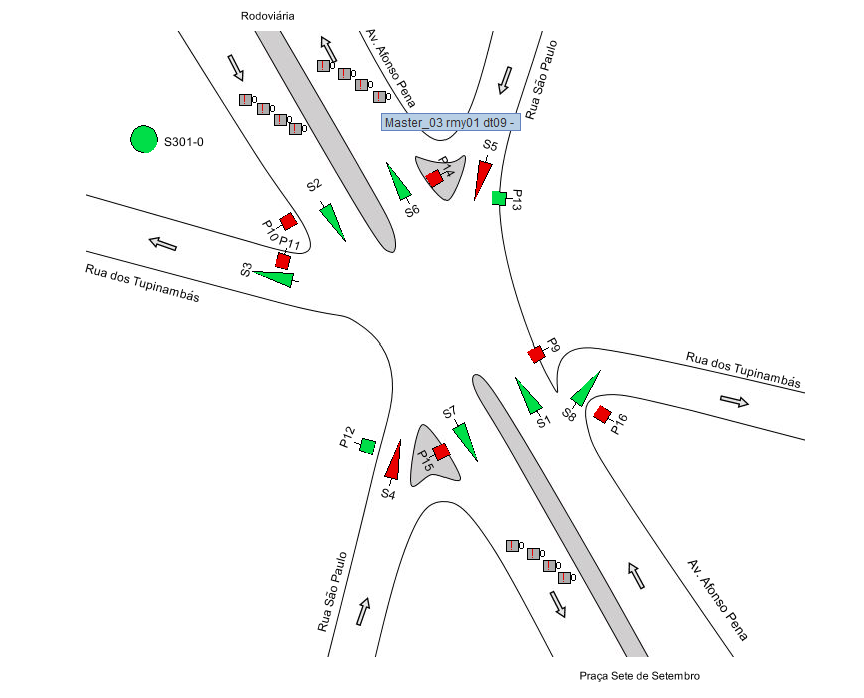


S7:1-4, S2:5-8


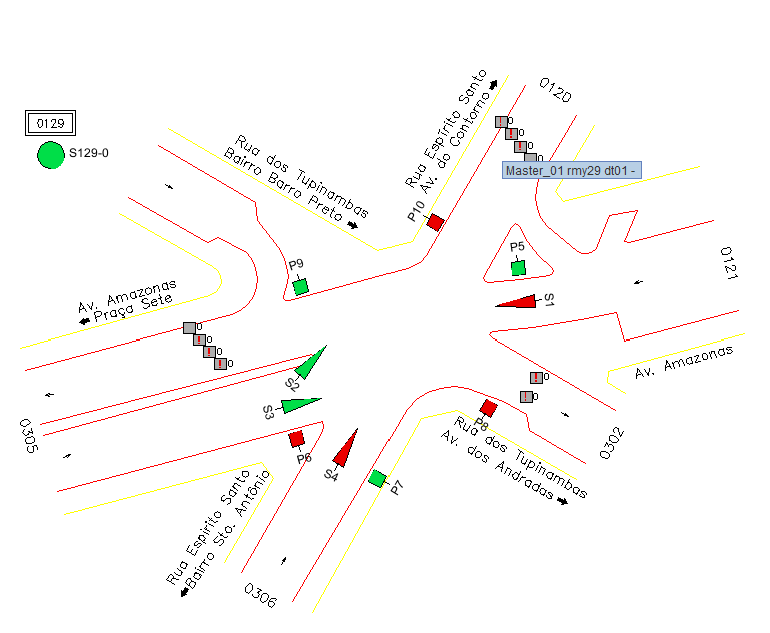


S3:5-6, S1:7-10


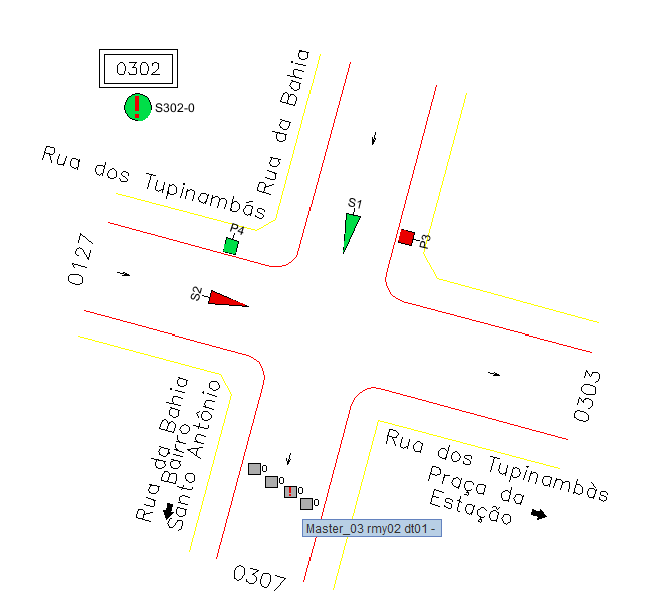


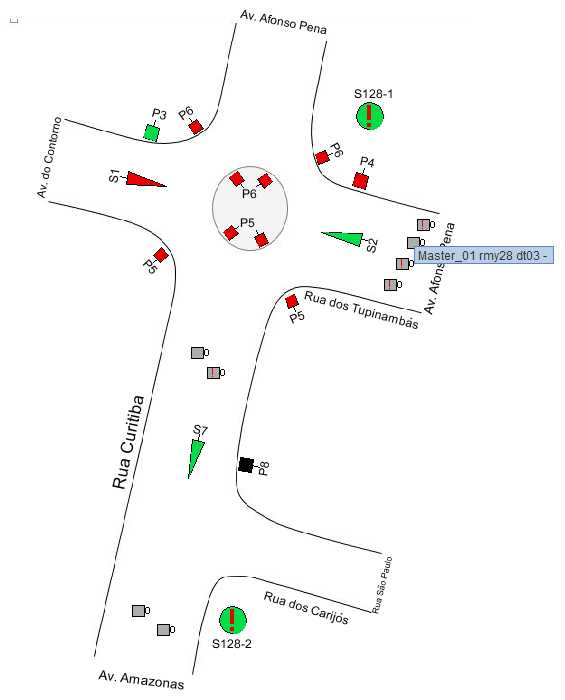


S7:7-8, S7-:1-2


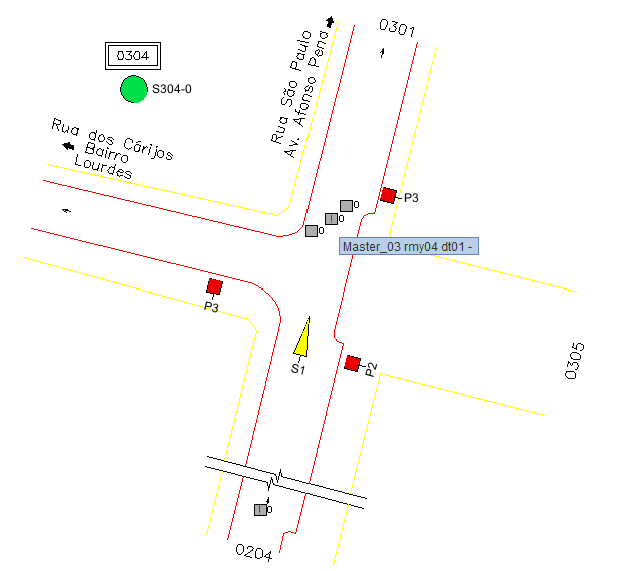


S1:4


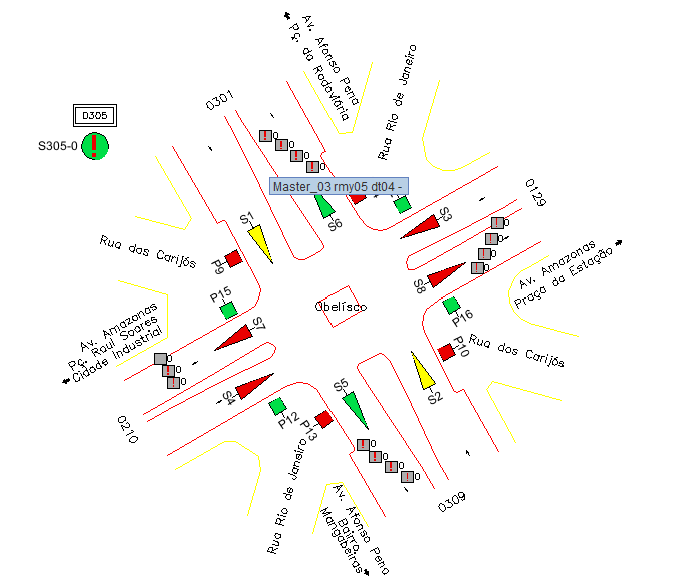


S8:8-11, S5:12-15, S7:1-3,


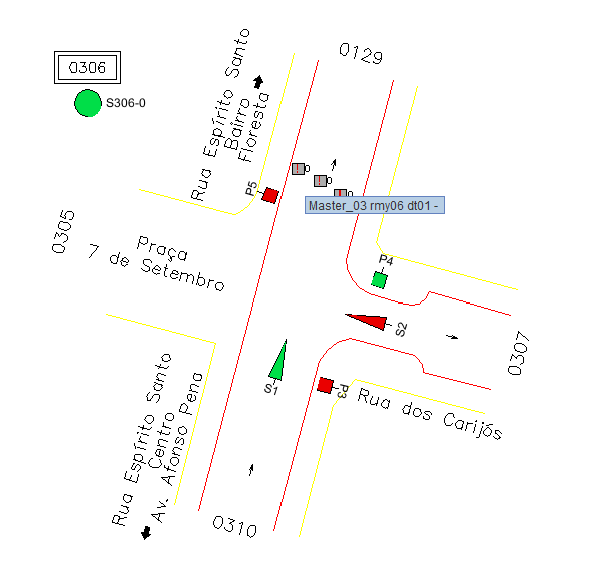


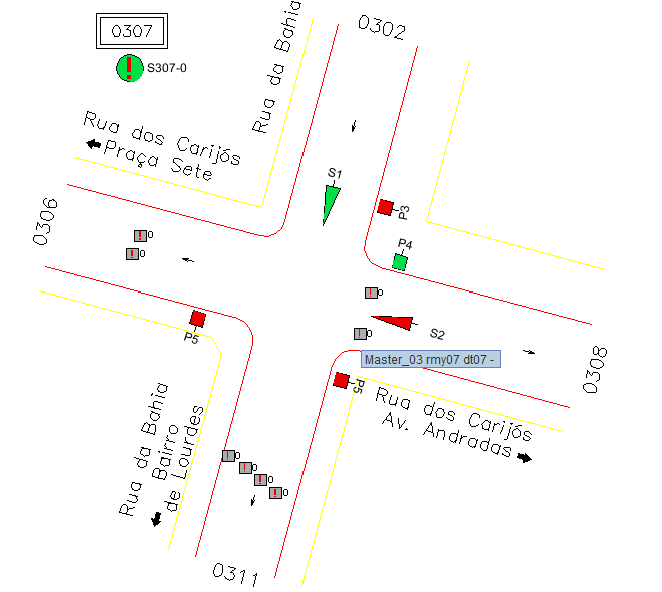


S1:1-4, S2:5-6


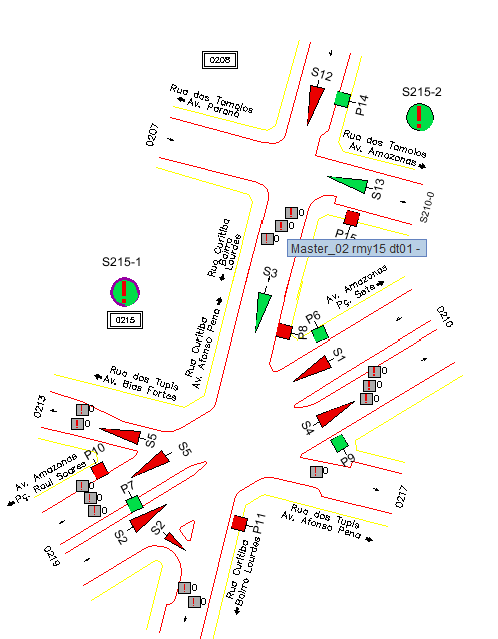


S4:4-6, S5-:7, S2:8-9, S5:10-12, S5:13-14,


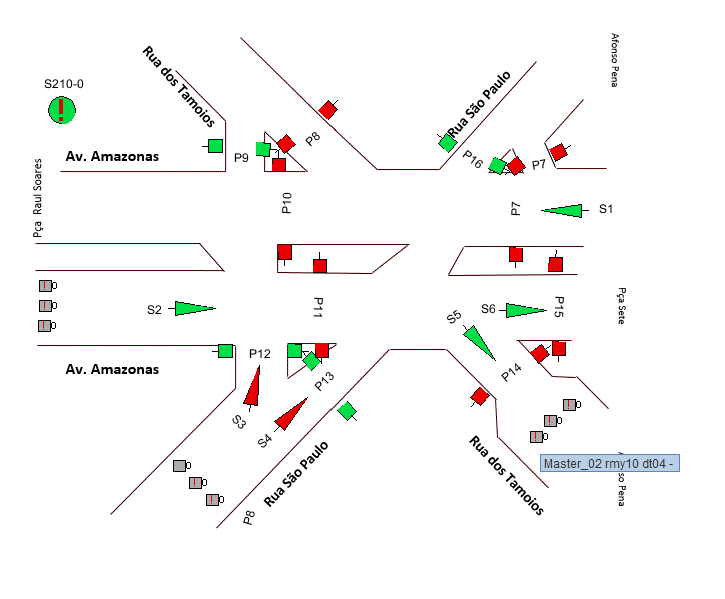


S4:7-9, S2:1-3,


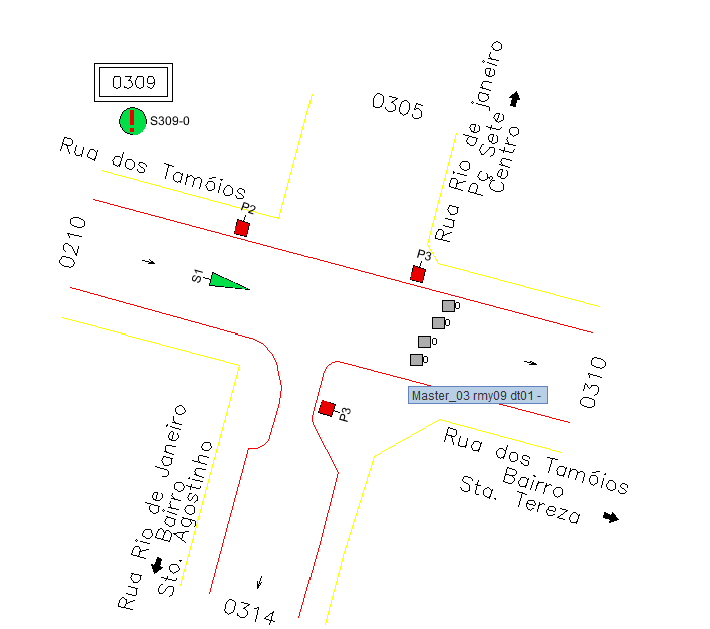


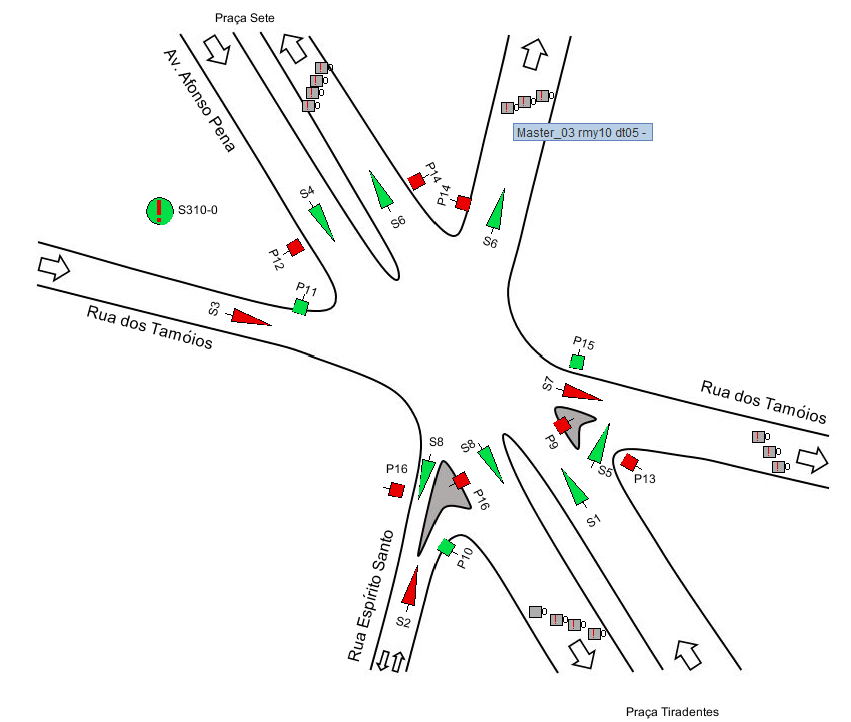


S7:8-10, S8:11-14, S6:1-4


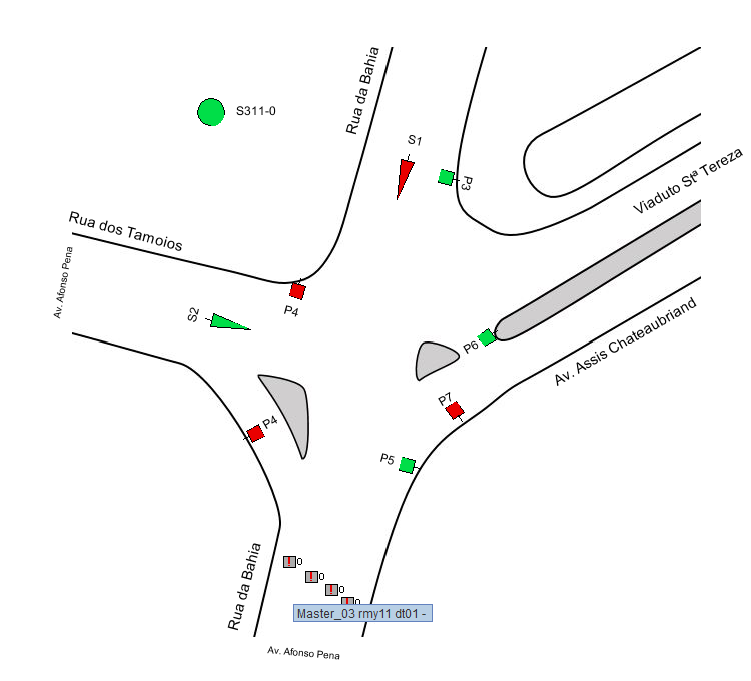


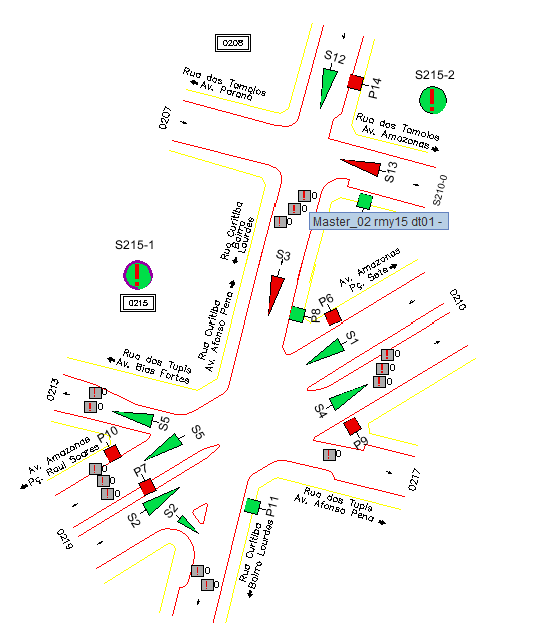


S4:4-6, S5-:7, S3:8-9, S5:10-12, S5:13-14


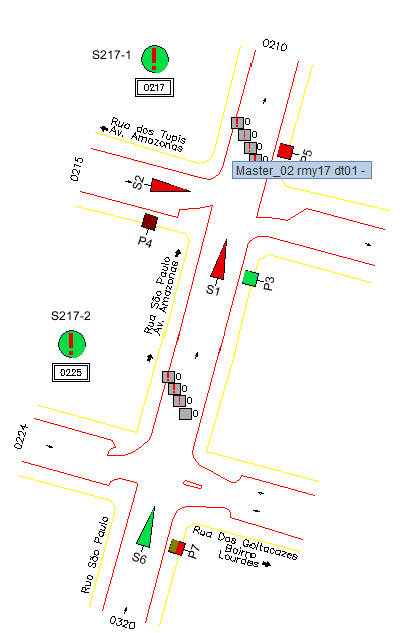


S6:5-8


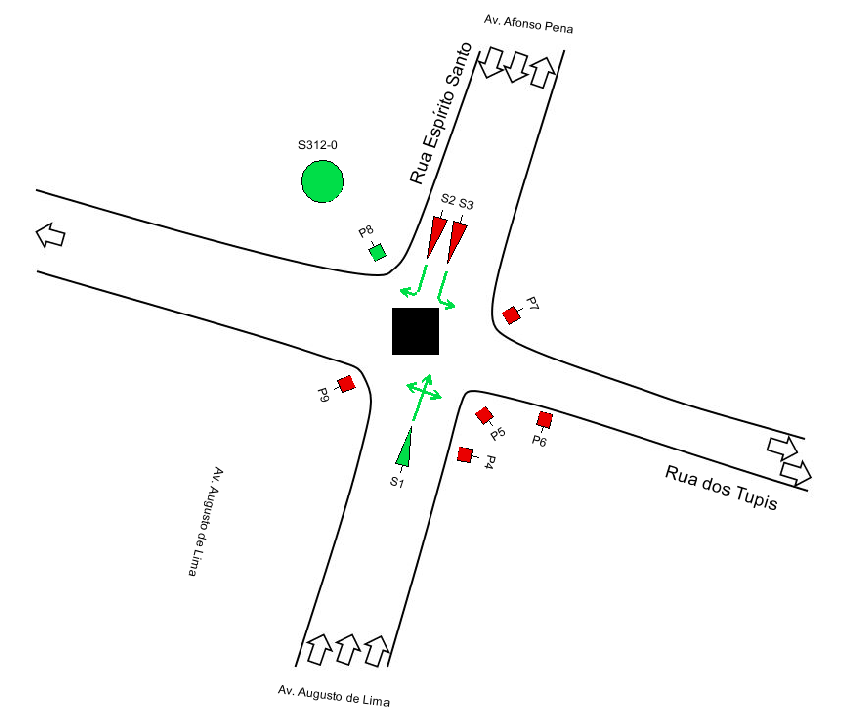


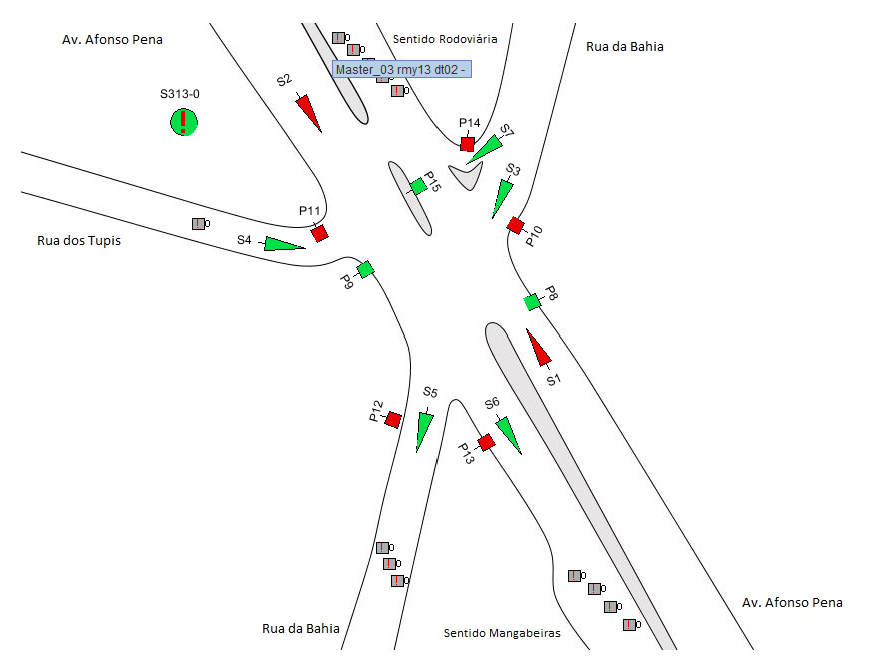


S6:7-10, S5:11-13, S4:1

# Savassi


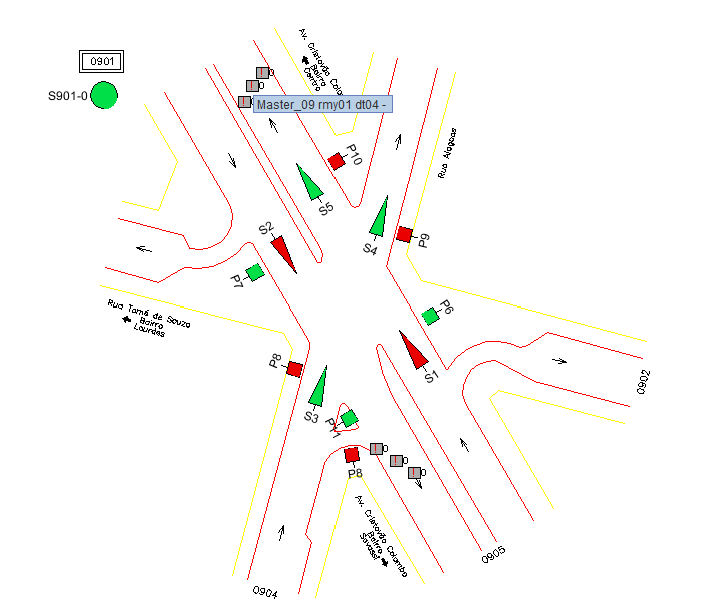


S2:1-3,


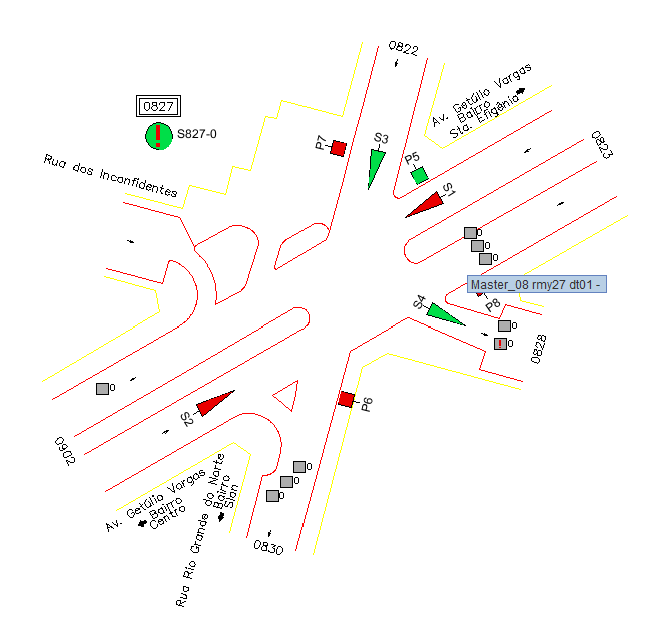


S4:4-5, S3:6-8, S1:9,


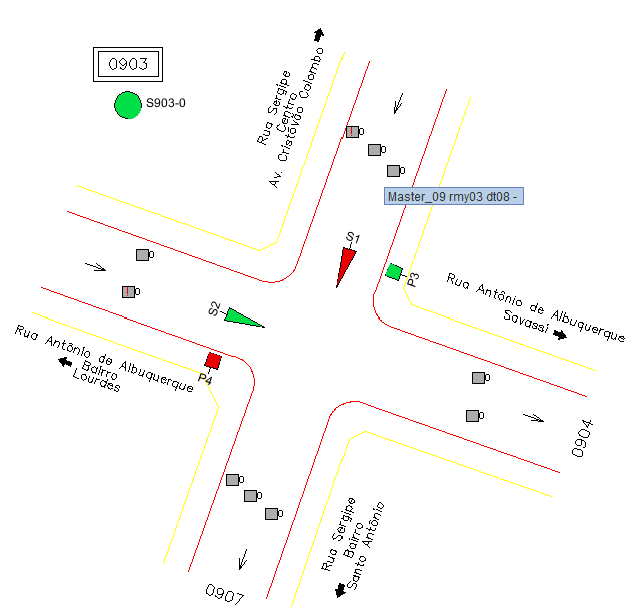


S2:1-2, S1:3-5, S2:6-7,


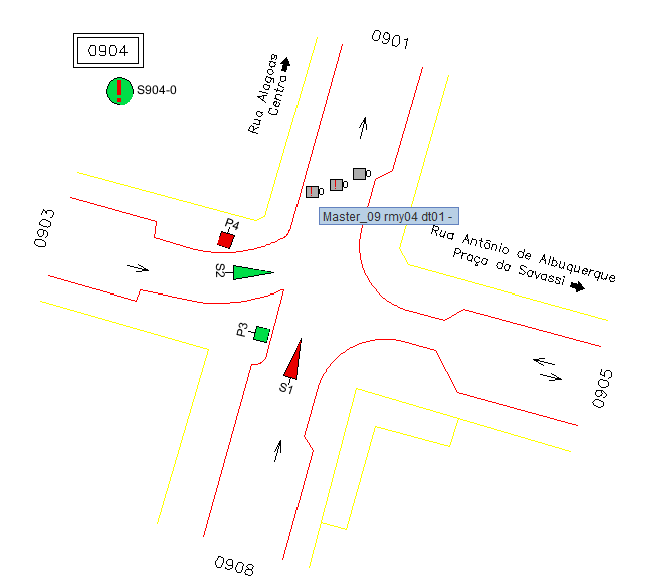


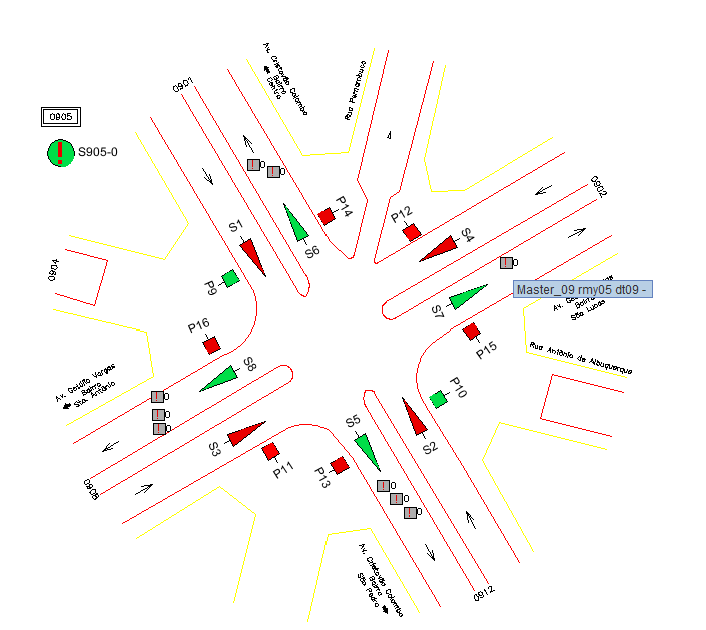


S5:1-3, S8:5-7, S6:7-8


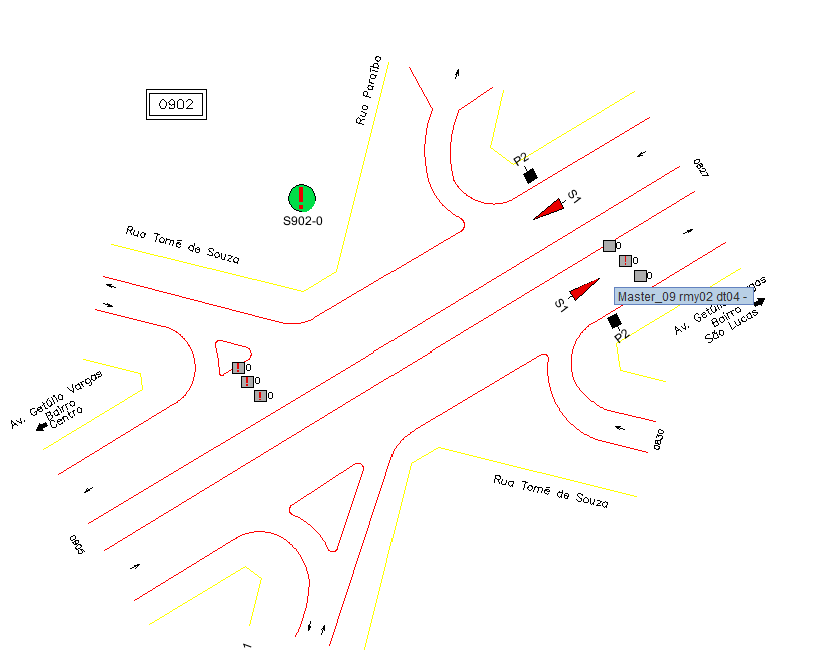


S1:1-3


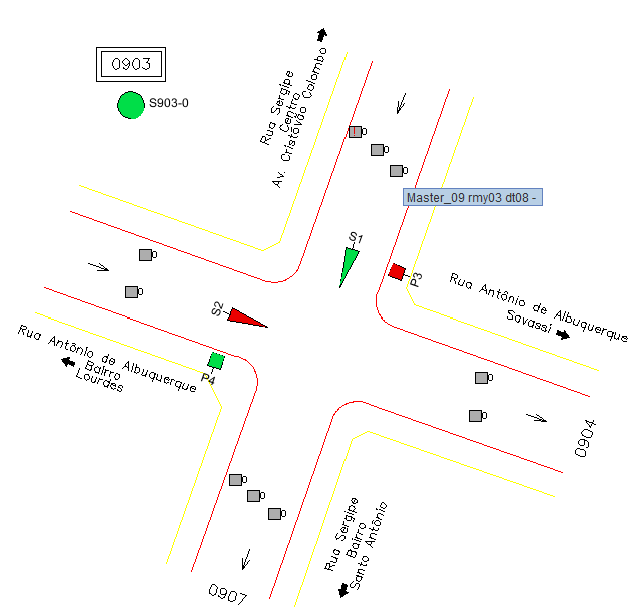


S2:1-2, S1:3-5, S2:6-7


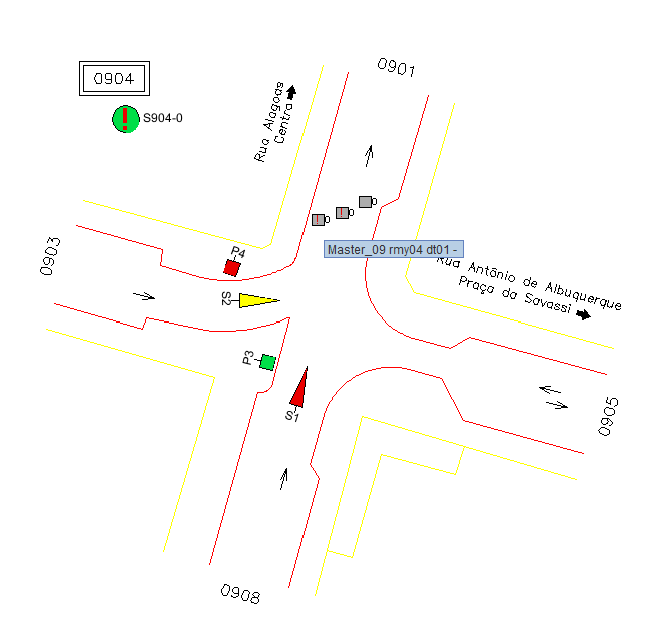


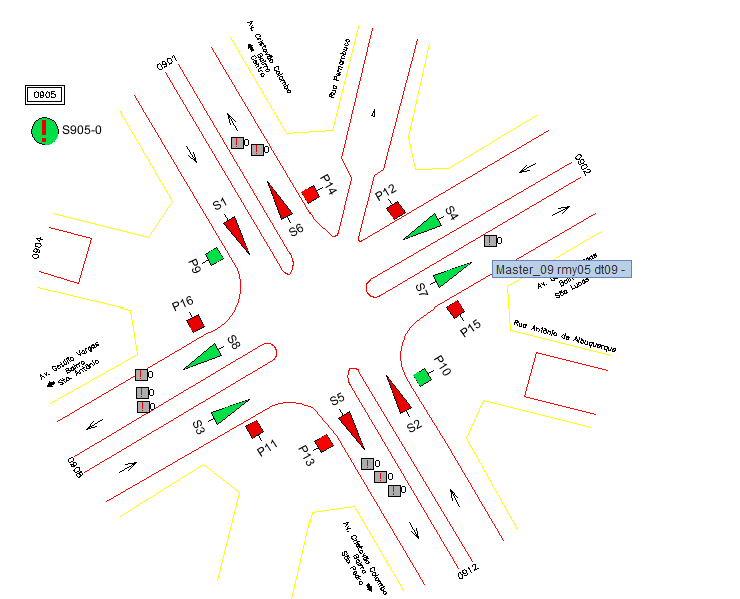


S4:1-3, S8:4-6, S6:7-8


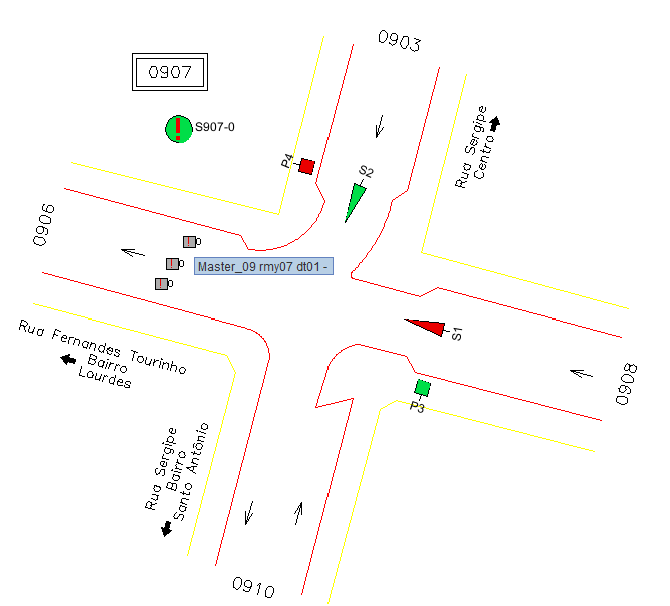


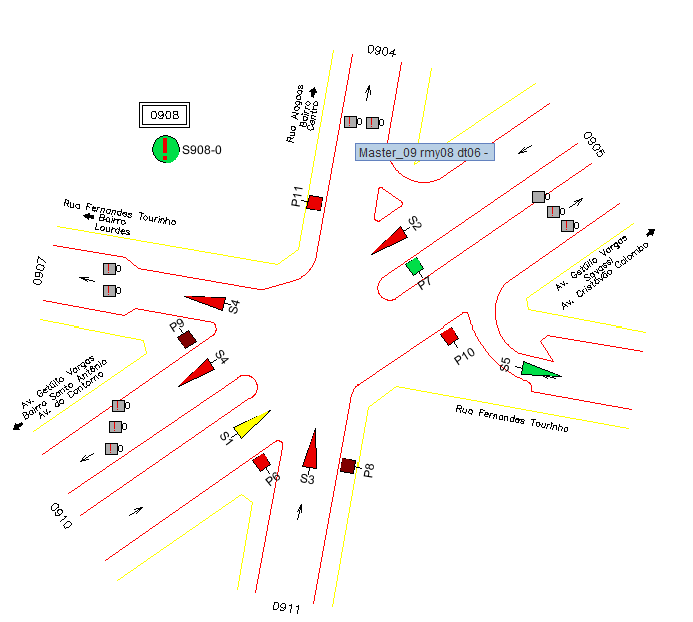


S1:8-10, S4:1-3, S4:4-5,


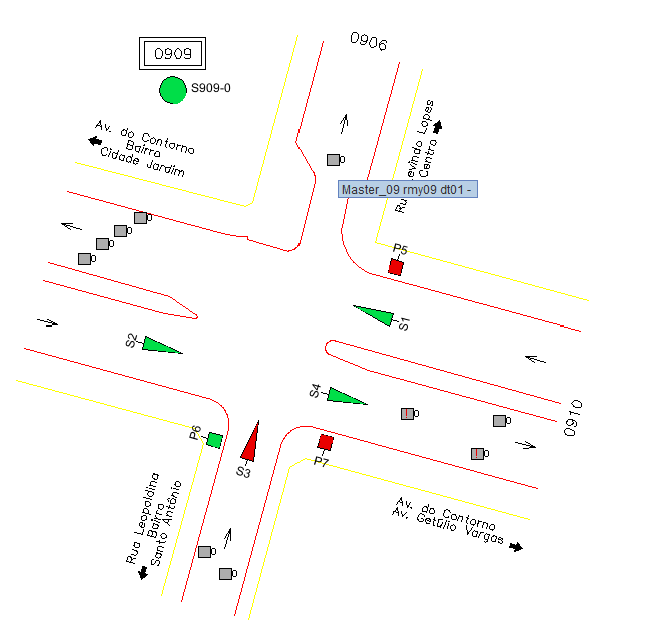
S4:2-4, S3:5-6S1:7-10


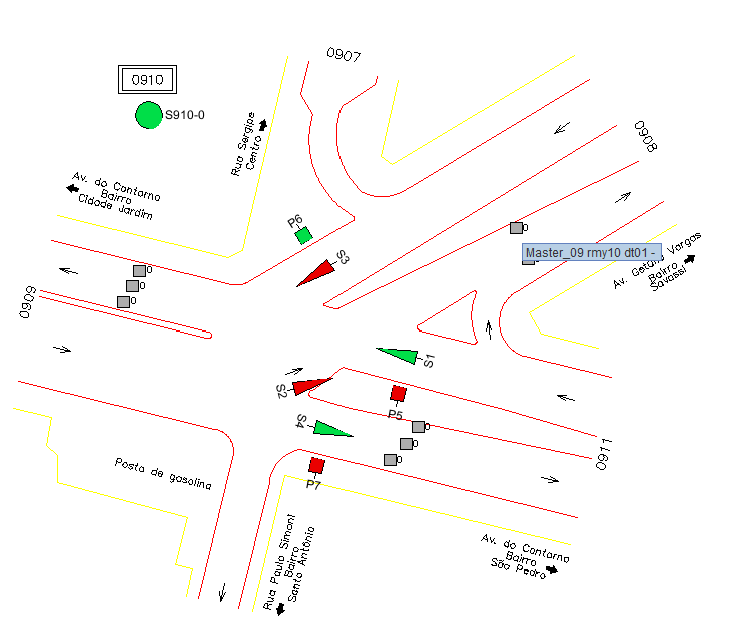


S4:3-5, S1:6-8


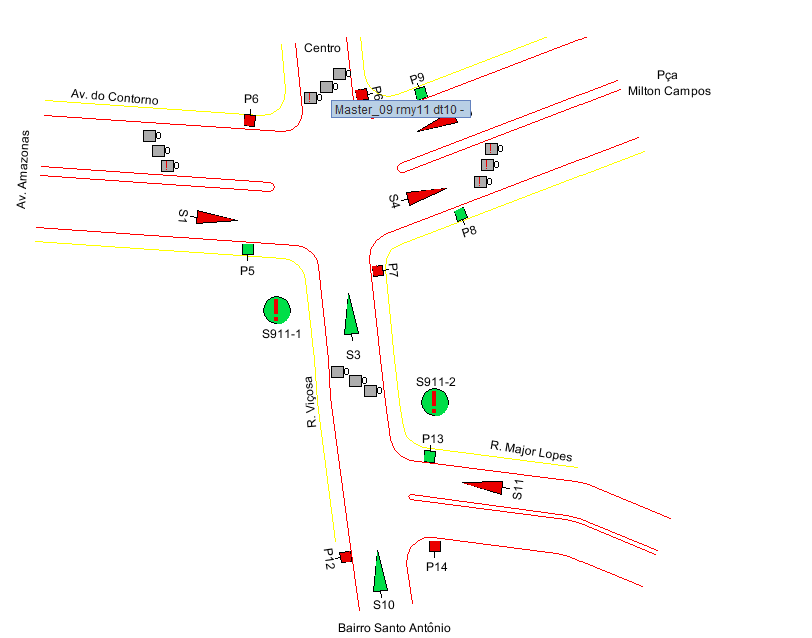


S4:4-6, S3:1-3, S2:7-9


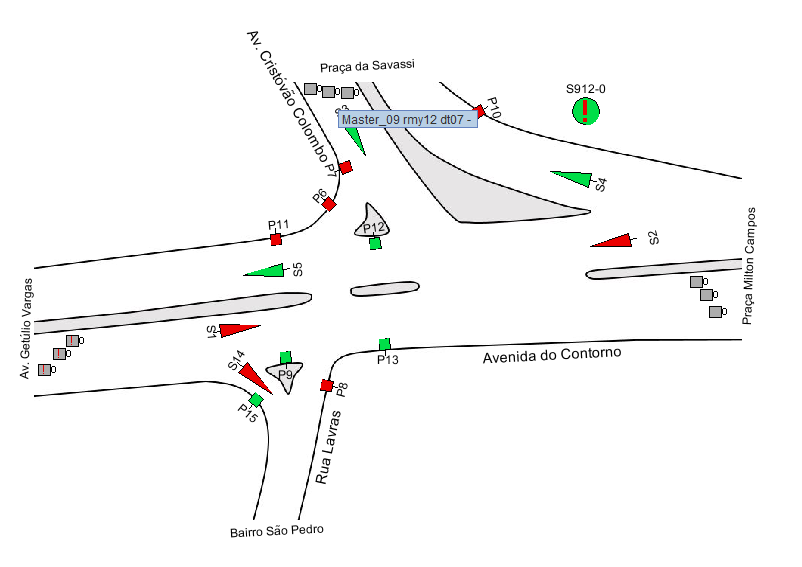


S1:1-3, S1-:4-6


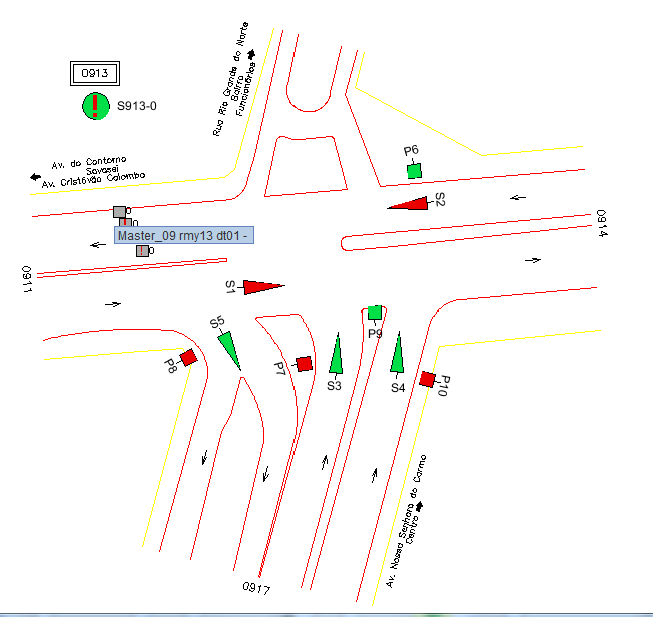


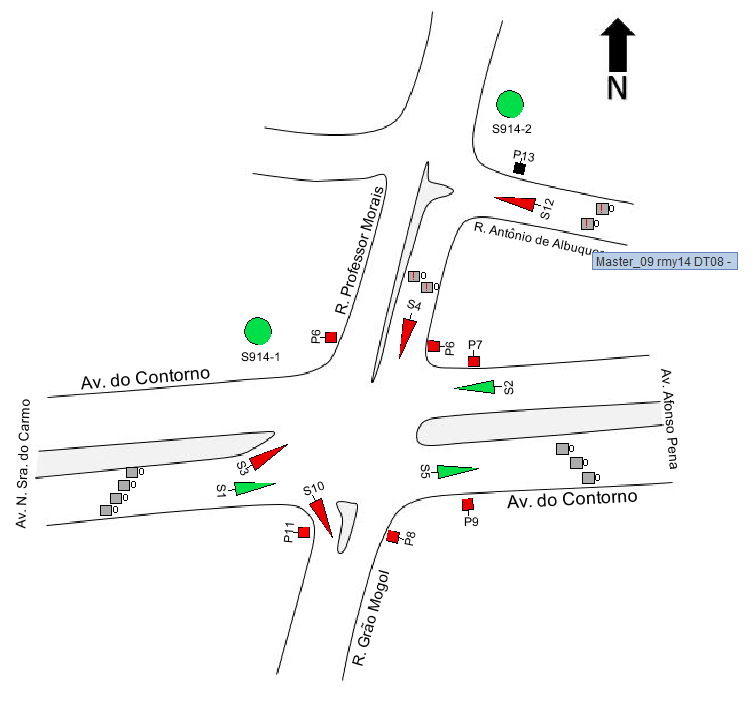


S5:1-3, S1-:4-7, S4:10-11


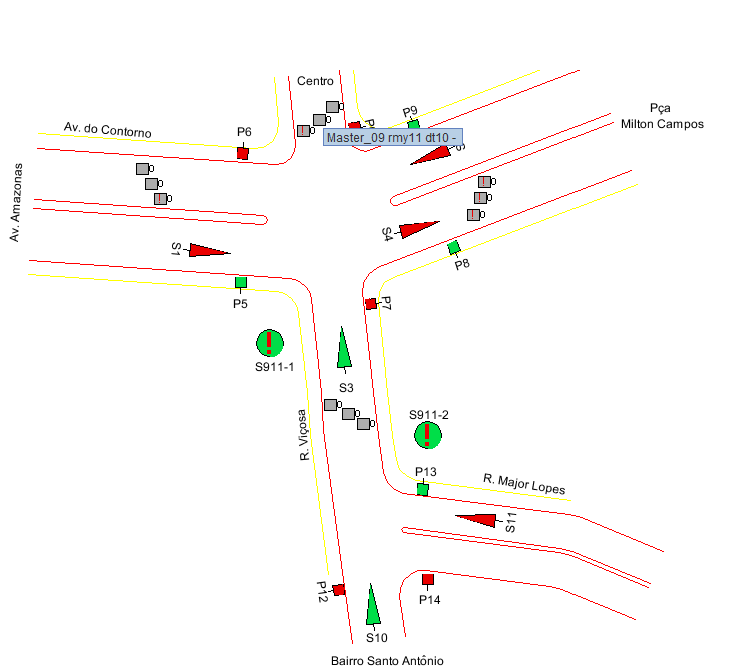


S4:4-6, S3:1-3, S2:7-9


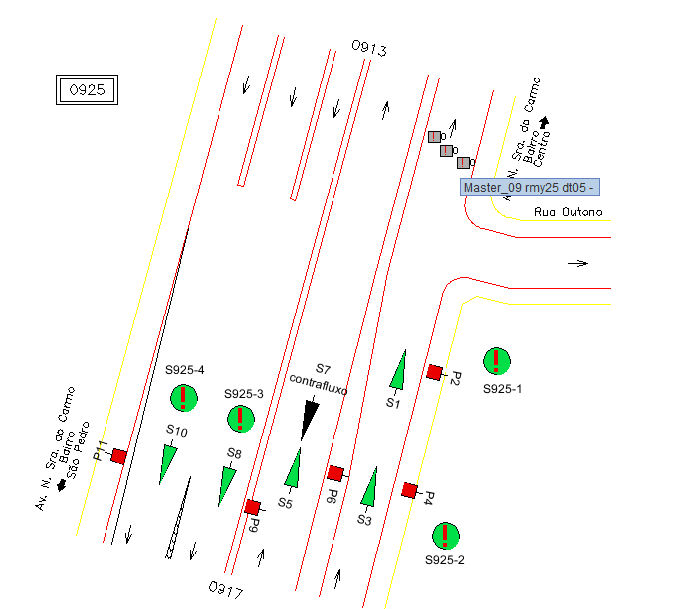


# Praça da estação


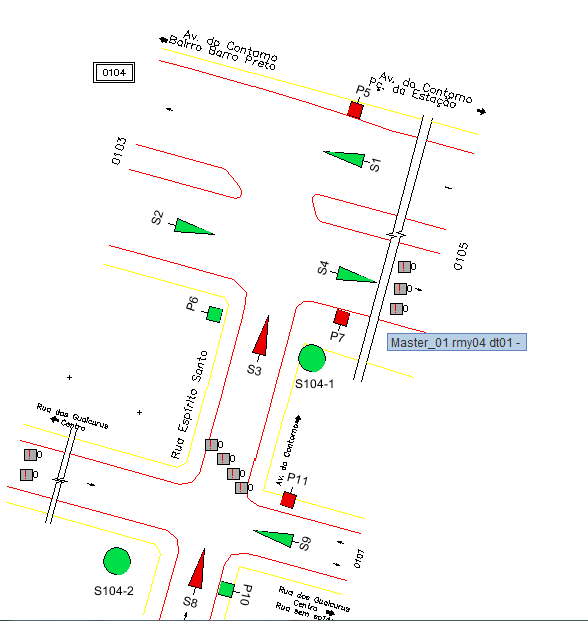


S3:4-7, S9:8-9


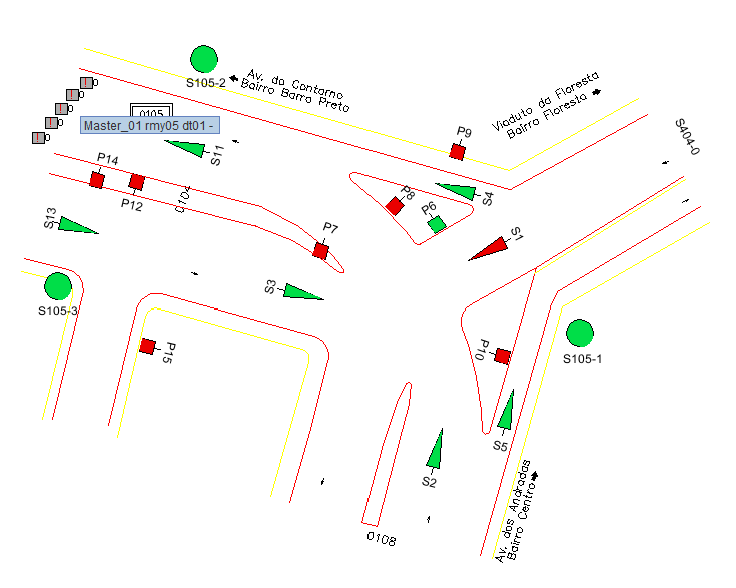


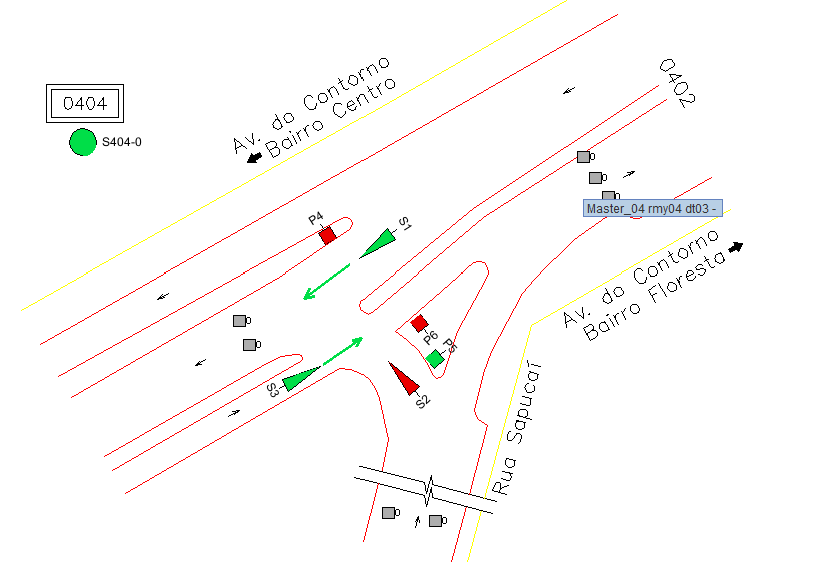


S2:6-7, S1:1-2


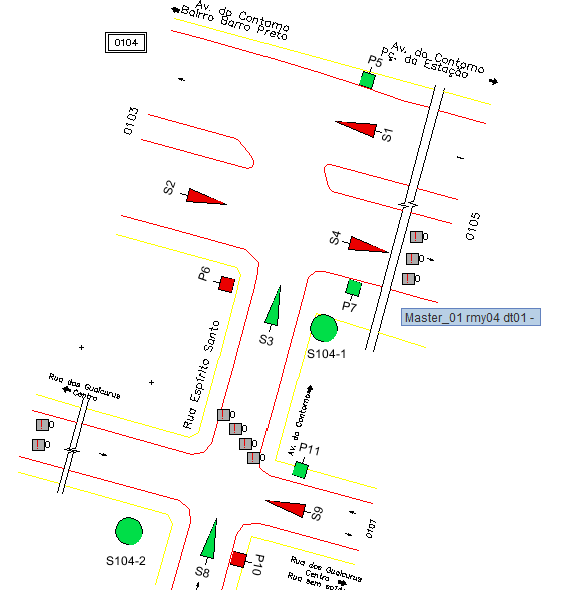


S3:4-7, S9:8-9


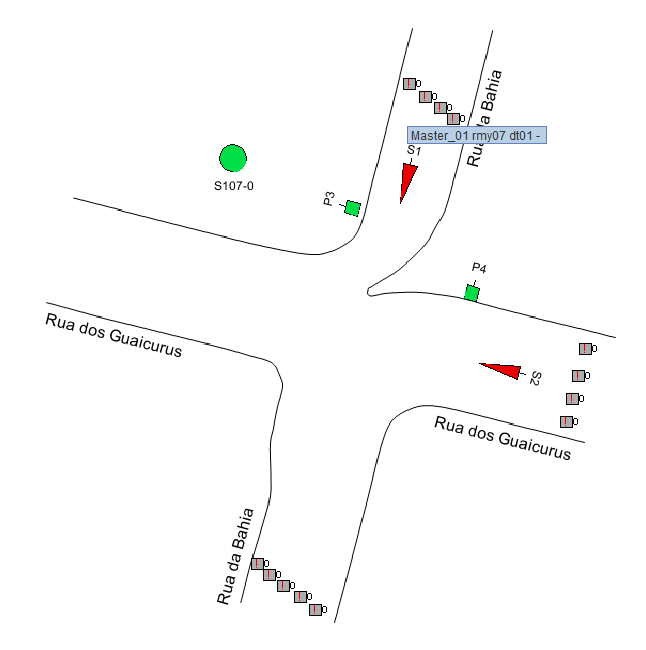


S2:5-8, S2d:9-13


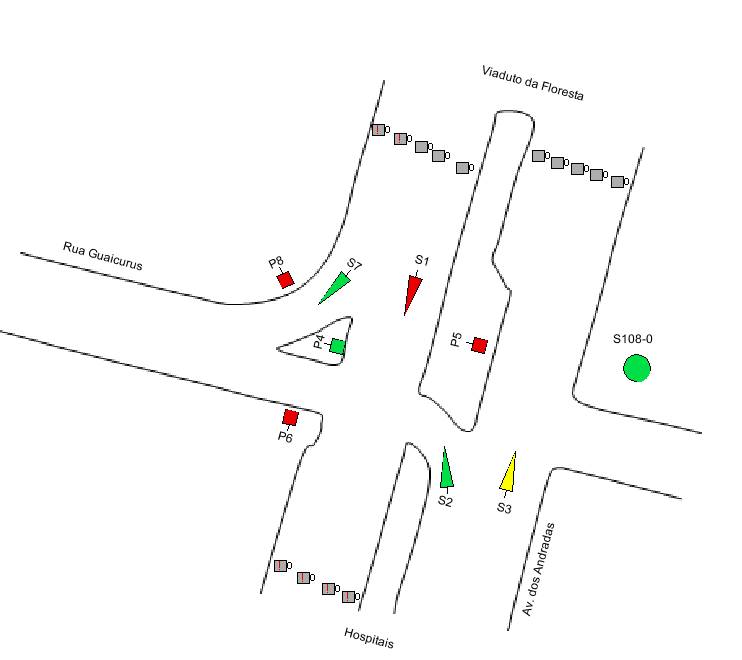


S1:11-14, s1-:1-5


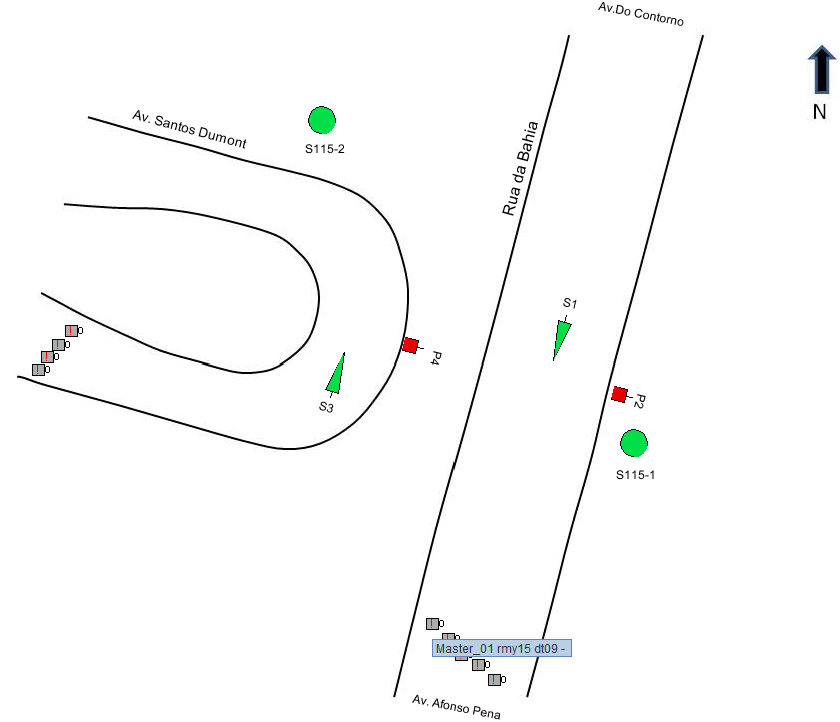


S3:1-4


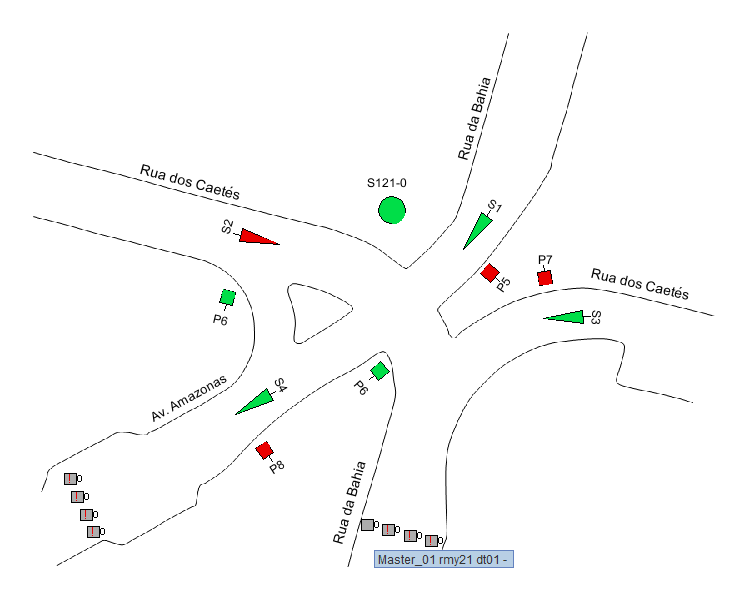


S4:5-8


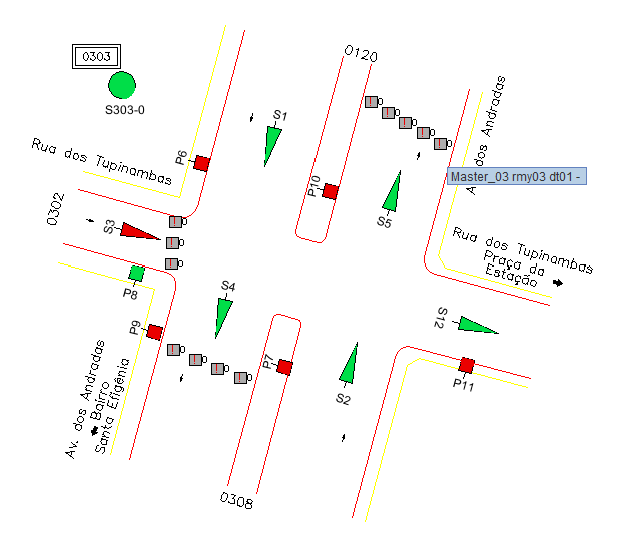


S4:6-9, S3:10-12


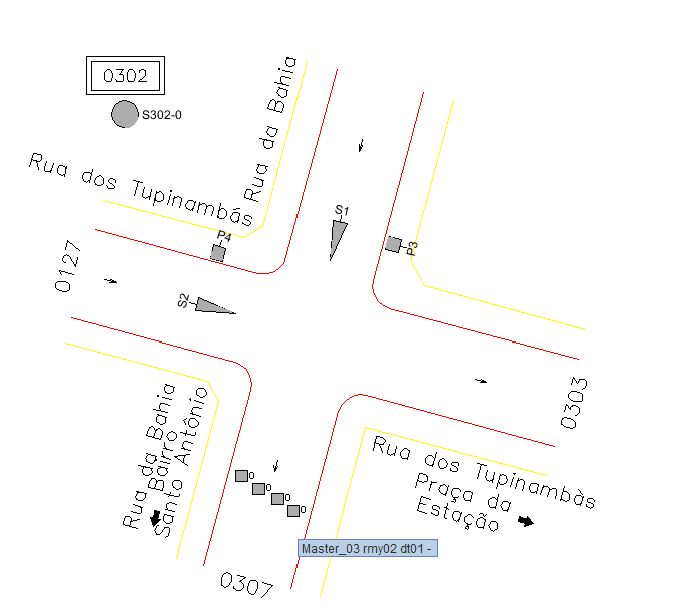


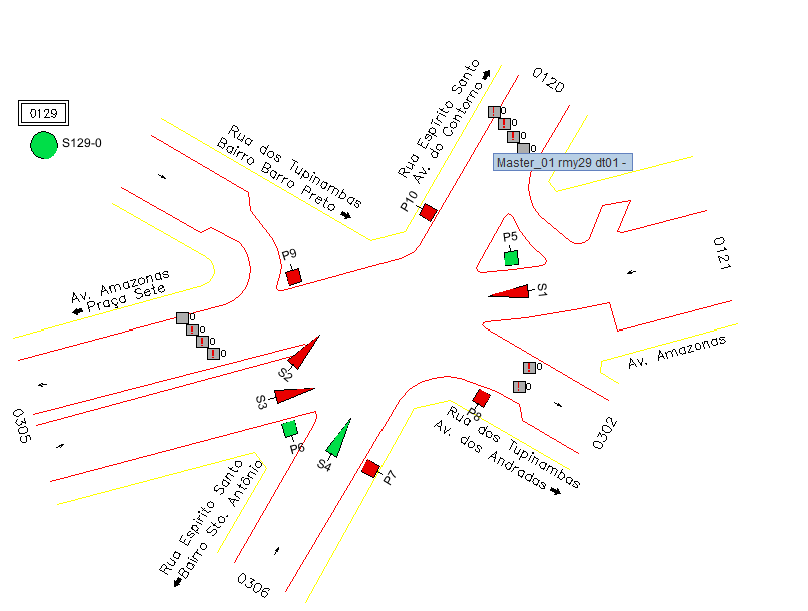


S3:5-6, S1:7-10


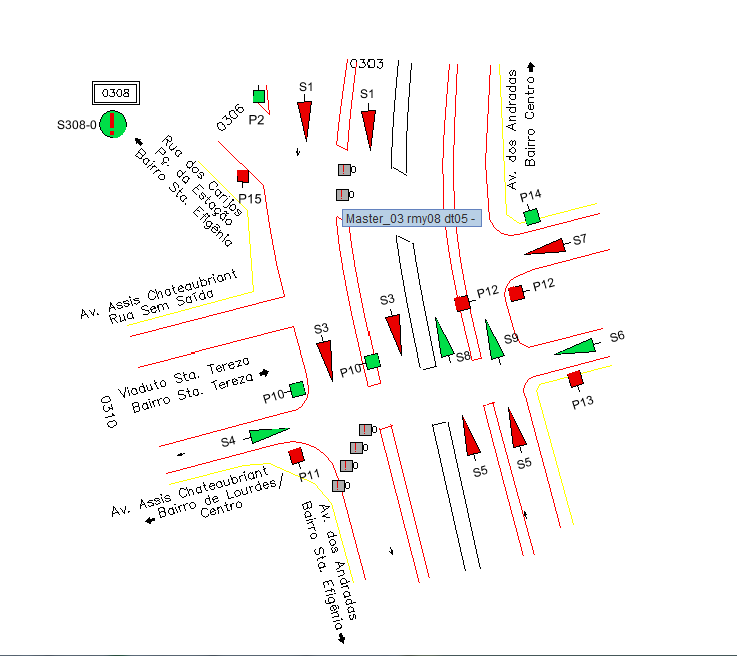


S3:1-4


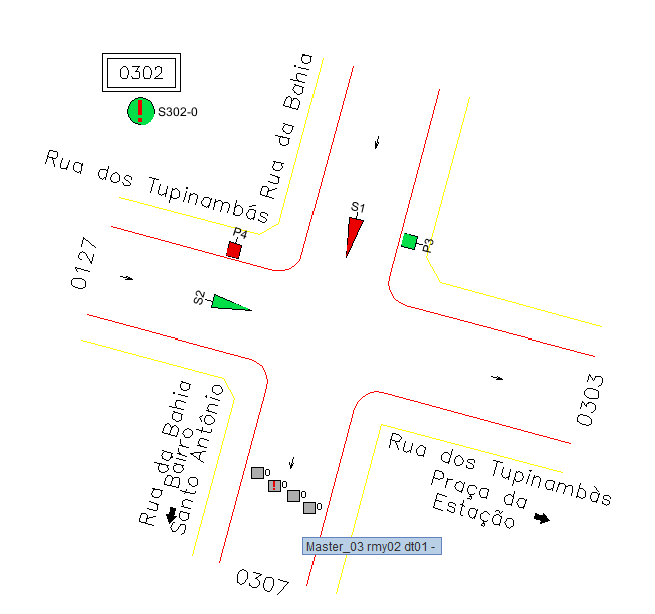


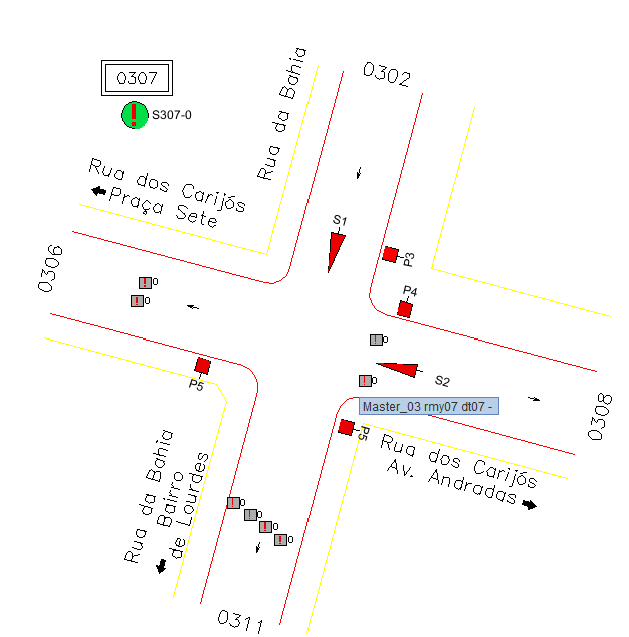


S1:1-4, S2:5-6


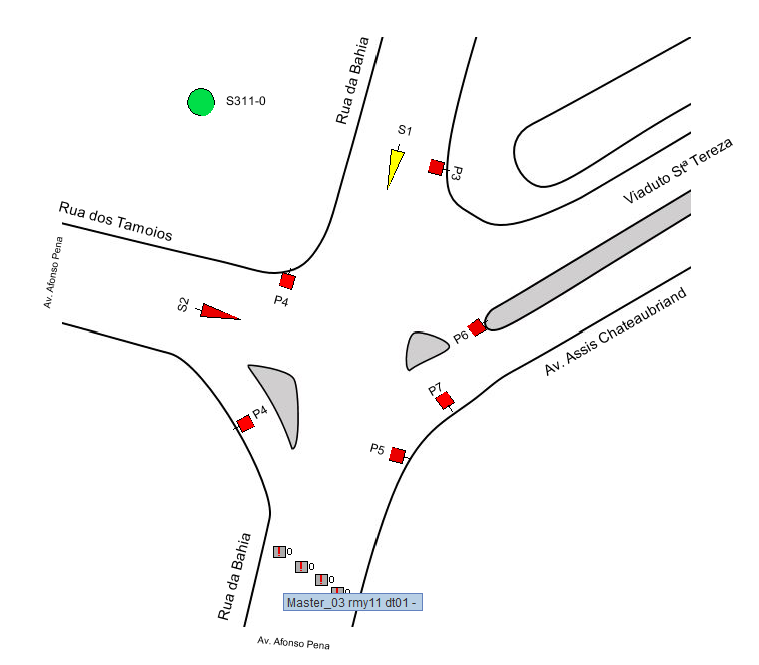

Supplement: Supplementary file 1 — Supplementary Information. [file 41598_2023_38884_MOESM1_ESM.zip › dadosBHTrans/dados BHTrans 2019/22_05_2019/dados3.docx]
