# Supplementary material for: Traffic light optimization using non-dominated sorting genetic algorithm (NSGA2)
Source: Sci Rep. 2023 Sep 20;13:15550. doi: 10.1038/s41598-023-38884-2 (PMC10511403; doi:10.1038/s41598-023-38884-2)
Supplement: Supplementary file 1 — Supplementary Information. [file 41598_2023_38884_MOESM1_ESM.zip › dadosBHTrans/dados2]

Sistema de Controle de Tráfego Urbano OPTIMUS

CARGA DE 4 PONTOS DE MEDIDA
DADOS DE 5 MINUTOS

PONTO DE MEDIDA 1:PM 0402004 (Contorno)
PONTO DE MEDIDA 2:PM 0402007 (Contorno)
PONTO DE MEDIDA 3:PM 04030031 (Contorno)
PONTO DE MEDIDA 4:PM 04030032 ()
DESDE:14/05/2015 00:00
ATÉ:15/05/201500:00

CARGA / 5 MINUTOS

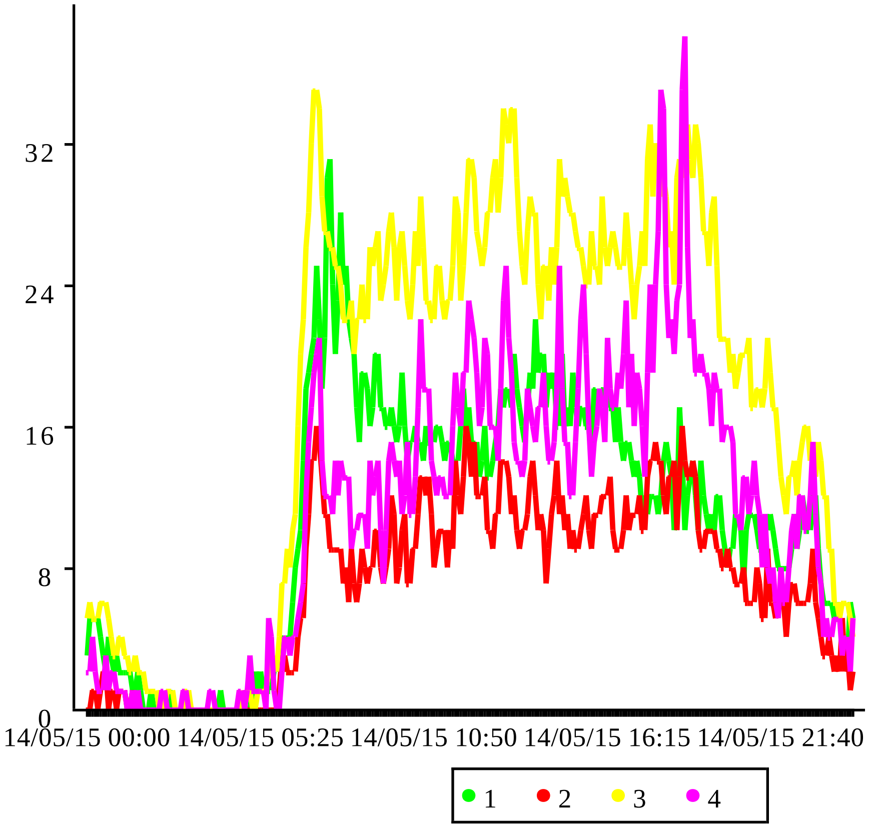

5 MINUTOS	CARGA			
	PM 0402004	PM 0402007	PM 04030031	PM 04030032
14/05/15 00:00	3	0	5	2
14/05/15 00:05	5	0	6	2
14/05/15 00:10	5	1	5	4
14/05/15 00:15	5	1	5	2
14/05/15 00:20	5	0	5	1
14/05/15 00:25	4	1	6	1
14/05/15 00:30	3	2	6	1
14/05/15 00:35	2	2	6	3
14/05/15 00:40	4	0	5	1
14/05/15 00:45	3	1	4	2
14/05/15 00:50	2	1	3	2
14/05/15 00:55	3	0	3	1
14/05/15 01:00	2	1	4	1
14/05/15 01:05	2	1	4	1
14/05/15 01:10	2	1	3	1

Sistema de Controle de Tráfego Urbano OPTIMUS

5 MINUTOS	CARGA			
	PM 0402004	PM 0402007	PM04030031	PM04030032
14/05/15 01:15	2	0	3	0
14/05/15 01:20	2	0	2	0
14/05/15 01:25	1	1	2	1
14/05/15 01:30	1	0	3	0
14/05/15 01:35	2	0	2	1
14/05/15 01:40	1	0	2	0
14/05/15 01:45	0	0	2	0
14/05/15 01:50	0	0	1	0
14/05/15 01:55	0	0	1	0
14/05/15 02:00	1	0	1	0
14/05/15 02:05	0	0	1	0
14/05/15 02:10	0	0	1	0
14/05/15 02:15	0	0	0	0
14/05/15 02:20	0	0	0	1
14/05/15 02:25	0	0	0	1
14/05/15 02:30	0	0	1	0
14/05/15 02:35	1	0	1	0
14/05/15 02:40	0	0	1	0
14/05/15 02:45	0	0	0	0
14/05/15 02:50	0	0	0	0
14/05/15 02:55	0	0	0	0
14/05/15 03:00	0	0	0	1
14/05/15 03:05	0	0	1	1
14/05/15 03:10	0	0	1	0
14/05/15 03:15	0	0	0	0
14/05/15 03:20	0	0	0	0
14/05/15 03:25	0	0	0	0
14/05/15 03:30	0	0	0	0
14/05/15 03:35	0	0	0	0
14/05/15 03:40	0	0	0	0
14/05/15 03:45	0	0	0	0
14/05/15 03:50	0	0	0	1
14/05/15 03:55	0	0	0	1
14/05/15 04:00	0	0	0	0
14/05/15 04:05	0	0	0	0
14/05/15 04:10	1	0	0	0
14/05/15 04:15	0	0	0	0
14/05/15 04:20	0	0	0	0
14/05/15 04:25	0	0	0	0
14/05/15 04:30	0	0	0	0
14/05/15 04:35	0	0	0	0
14/05/15 04:40	0	0	0	0
14/05/15 04:45	0	0	0	1
14/05/15 04:50	0	0	1	1
14/05/15 04:55	0	0	0	0
14/05/15 05:00	0	0	1	1
14/05/15 05:05	0	0	1	3
14/05/15 05:10	1	0	0	1
14/05/15 05:15	2	0	0	1
14/05/15 05:20	1	0	1	1
14/05/15 05:25	2	0	1	1
14/05/15 05:30	1	0	1	1
14/05/15 05:35	1	0	1	0
14/05/15 05:40	1	0	2	5
14/05/15 05:45	1	0	3	4
14/05/15 05:50	2	0	3	1
14/05/15 05:55	2	0	2	0
14/05/15 06:00	3	1	4	0

Sistema de Controle de Tráfego Urbano OPTIMUS

5 MINUTOS	CARGA			
	PM 0402004	PM 0402007	PM04030031	PM04030032
14/05/15 06:05	4	3	7	2
14/05/15 06:10	4	3	7	4
14/05/15 06:15	4	2	9	4
14/05/15 06:20	4	2	8	3
14/05/15 06:25	6	2	10	4
14/05/15 06:30	8	2	11	4
14/05/15 06:35	9	4	16	5
14/05/15 06:40	10	5	20	6
14/05/15 06:45	14	5	22	7
14/05/15 06:50	18	9	26	12
14/05/15 06:55	19	11	28	15
14/05/15 07:00	20	14	32	17
14/05/15 07:05	21	14	35	19
14/05/15 07:10	25	16	35	20
14/05/15 07:15	22	15	34	21
14/05/15 07:20	18	13	29	14
14/05/15 07:25	21	11	27	12
14/05/15 07:30	30	11	27	12
14/05/15 07:35	31	9	26	12
14/05/15 07:40	24	9	26	11
14/05/15 07:45	20	9	25	14
14/05/15 07:50	23	9	25	12
14/05/15 07:55	28	9	24	14
14/05/15 08:00	24	7	22	13
14/05/15 08:05	25	8	22	13
14/05/15 08:10	22	6	22	13
14/05/15 08:15	21	9	23	9
14/05/15 08:20	20	7	20	10
14/05/15 08:25	17	6	22	10
14/05/15 08:30	15	7	22	11
14/05/15 08:35	19	9	24	11
14/05/15 08:40	19	8	22	11
14/05/15 08:45	18	7	22	9
14/05/15 08:50	16	8	26	14
14/05/15 08:55	17	8	25	12
14/05/15 09:00	20	10	26	13
14/05/15 09:05	20	10	27	14
14/05/15 09:10	17	8	23	10
14/05/15 09:15	17	7	24	7
14/05/15 09:20	16	8	25	10
14/05/15 09:25	16	9	27	14
14/05/15 09:30	17	12	28	15
14/05/15 09:35	16	11	26	14
14/05/15 09:40	15	7	23	13
14/05/15 09:45	16	8	26	14
14/05/15 09:50	19	10	27	11
14/05/15 09:55	16	11	25	12
14/05/15 10:00	14	7	23	15
14/05/15 10:05	15	7	22	11
14/05/15 10:10	15	9	24	11
14/05/15 10:15	16	9	27	13
14/05/15 10:20	15	11	25	17
14/05/15 10:25	15	13	29	22
14/05/15 10:30	14	13	26	18
14/05/15 10:35	16	12	23	18
14/05/15 10:40	15	13	23	18
14/05/15 10:45	15	11	22	14
14/05/15 10:50	15	8	22	13

Sistema de Controle de Tráfego Urbano OPTIMUS

5 MINUTOS	CARGA			
	PM 0402004	PM 0402007	PM 04030031	PM 04030032
14/05/15 10:55	16	9	25	12
14/05/15 11:00	16	10	25	13
14/05/15 11:05	15	10	23	13
14/05/15 11:10	14	10	22	12
14/05/15 11:15	15	8	23	12
14/05/15 11:20	15	10	23	12
14/05/15 11:25	14	9	25	16
14/05/15 11:30	14	14	29	19
14/05/15 11:35	14	12	28	17
14/05/15 11:40	16	11	23	16
14/05/15 11:45	18	13	25	19
14/05/15 11:50	16	16	28	19
14/05/15 11:55	17	15	31	23
14/05/15 12:00	15	13	31	22
14/05/15 12:05	15	15	30	21
14/05/15 12:10	15	12	27	19
14/05/15 12:15	13	12	26	16
14/05/15 12:20	14	12	25	17
14/05/15 12:25	16	13	26	21
14/05/15 12:30	13	10	28	20
14/05/15 12:35	13	10	28	16
14/05/15 12:40	14	9	30	16
14/05/15 12:45	15	11	31	16
14/05/15 12:50	16	11	28	14
14/05/15 12:55	18	14	30	18
14/05/15 13:00	17	14	34	23
14/05/15 13:05	18	14	33	25
14/05/15 13:10	18	13	32	21
14/05/15 13:15	17	11	34	19
14/05/15 13:20	20	12	34	15
14/05/15 13:25	18	10	30	14
14/05/15 13:30	17	9	27	14
14/05/15 13:35	16	10	25	13
14/05/15 13:40	15	10	24	14
14/05/15 13:45	17	11	27	18
14/05/15 13:50	19	13	29	17
14/05/15 13:55	18	14	28	16
14/05/15 14:00	22	12	28	15
14/05/15 14:05	19	10	24	17
14/05/15 14:10	20	11	22	17
14/05/15 14:15	20	10	25	19
14/05/15 14:20	17	7	25	16
14/05/15 14:25	19	9	23	14
14/05/15 14:30	18	11	26	14
14/05/15 14:35	19	12	24	15
14/05/15 14:40	16	14	26	18
14/05/15 14:45	16	11	31	25
14/05/15 14:50	20	12	29	18
14/05/15 14:55	16	10	30	15
14/05/15 15:00	17	11	29	15
14/05/15 15:05	16	9	28	12
14/05/15 15:10	19	10	28	12
14/05/15 15:15	17	9	27	15
14/05/15 15:20	16	9	26	18
14/05/15 15:25	17	10	26	22
14/05/15 15:30	17	11	25	24
14/05/15 15:35	16	12	24	20
14/05/15 15:40	16	10	24	16

Sistema de Controle de Tráfego Urbano OPTIMUS

5 MINUTOS	CARGA			
	PM 0402004	PM 0402007	PM 04030031	PM 04030032
14/05/15 15:45	14	9	27	13
14/05/15 15:50	18	11	25	15
14/05/15 15:55	18	11	25	16
14/05/15 16:00	17	11	24	18
14/05/15 16:05	18	12	29	17
14/05/15 16:10	18	12	26	15
14/05/15 16:15	18	12	25	21
14/05/15 16:20	17	13	26	18
14/05/15 16:25	17	10	27	17
14/05/15 16:30	15	9	26	17
14/05/15 16:35	17	9	25	19
14/05/15 16:40	15	9	25	18
14/05/15 16:45	14	10	25	20
14/05/15 16:50	15	12	28	23
14/05/15 16:55	15	10	26	17
14/05/15 17:00	14	11	24	20
14/05/15 17:05	13	11	22	16
14/05/15 17:10	14	11	24	19
14/05/15 17:15	13	12	25	18
14/05/15 17:20	11	10	27	16
14/05/15 17:25	12	10	25	13
14/05/15 17:30	11	13	31	19
14/05/15 17:35	12	14	33	24
14/05/15 17:40	12	14	29	19
14/05/15 17:45	12	15	32	24
14/05/15 17:50	11	14	31	27
14/05/15 17:55	12	14	29	35
14/05/15 18:00	14	12	30	34
14/05/15 18:05	15	11	29	24
14/05/15 18:10	14	13	26	21
14/05/15 18:15	13	13	27	22
14/05/15 18:20	10	14	24	20
14/05/15 18:25	11	10	30	23
14/05/15 18:30	17	13	31	24
14/05/15 18:35	14	16	30	35
14/05/15 18:40	10	14	33	38
14/05/15 18:45	12	13	33	26
14/05/15 18:50	13	13	31	21
14/05/15 18:55	14	14	30	22
14/05/15 19:00	12	13	33	19
14/05/15 19:05	10	10	32	19
14/05/15 19:10	14	9	30	20
14/05/15 19:15	12	9	27	19
14/05/15 19:20	11	10	27	19
14/05/15 19:25	10	10	25	18
14/05/15 19:30	11	10	28	16
14/05/15 19:35	10	10	29	19
14/05/15 19:40	12	9	25	18
14/05/15 19:45	12	9	21	18
14/05/15 19:50	10	8	21	15
14/05/15 19:55	9	8	21	16
14/05/15 20:00	8	9	21	16
14/05/15 20:05	9	8	19	16
14/05/15 20:10	9	8	20	15
14/05/15 20:15	11	7	18	11
14/05/15 20:20	11	7	19	11
14/05/15 20:25	10	7	20	10
14/05/15 20:30	7	8	20	13

Sistema de Controle de Tráfego Urbano OPTIMUS

5 MINUTOS	CARGA			
	PM 0402004	PM 0402007	PM04030031	PM04030032
14/05/15 20:35	10	6	20	13
14/05/15 20:40	11	6	21	11
14/05/15 20:45	11	6	17	12
14/05/15 20:50	11	6	17	14
14/05/15 20:55	10	8	18	12
14/05/15 21:00	9	7	18	11
14/05/15 21:05	9	5	17	8
14/05/15 21:10	10	5	18	11
14/05/15 21:15	10	9	21	8
14/05/15 21:20	11	6	19	7
14/05/15 21:25	10	6	17	8
14/05/15 21:30	9	5	17	6
14/05/15 21:35	8	6	15	5
14/05/15 21:40	8	7	13	8
14/05/15 21:45	8	6	12	6
14/05/15 21:50	8	4	11	6
14/05/15 21:55	8	6	13	8
14/05/15 22:00	9	7	13	10
14/05/15 22:05	11	7	14	11
14/05/15 22:10	9	6	12	9
14/05/15 22:15	10	6	14	12
14/05/15 22:20	12	6	15	12
14/05/15 22:25	11	6	16	10
14/05/15 22:30	10	6	16	10
14/05/15 22:35	10	7	14	12
14/05/15 22:40	12	9	15	15
14/05/15 22:45	12	6	13	11
14/05/15 22:50	9	5	15	8
14/05/15 22:55	7	4	14	7
14/05/15 23:00	6	3	12	4
14/05/15 23:05	6	3	12	5
14/05/15 23:10	6	4	9	4
14/05/15 23:15	6	3	9	4
14/05/15 23:20	5	2	6	5
14/05/15 23:25	6	3	6	5
14/05/15 23:30	5	2	5	5
14/05/15 23:35	4	5	6	3
14/05/15 23:40	4	2	6	4
14/05/15 23:45	3	3	6	4
14/05/15 23:50	6	1	5	2
14/05/15 23:55	5	2	4	5
