# Supplementary material for: Traffic light optimization using non-dominated sorting genetic algorithm (NSGA2)
Source: Sci Rep. 2023 Sep 20;13:15550. doi: 10.1038/s41598-023-38884-2 (PMC10511403; doi:10.1038/s41598-023-38884-2)
Supplement: Supplementary file 1 — Supplementary Information. [file 41598_2023_38884_MOESM1_ESM.zip › dadosBHTrans/dados3]

Sistema de Controle de Tráfego Urbano OPTIMUS

CARGA DE 4 PONTOS DE MEDIDA

DADOS DE 5 MINUTOS

PONTO DE MEDIDA 1:PM 0403004 (Floresta)
PONTO DE MEDIDA 2:PM 0403006 ()
PONTO DE MEDIDA 3:PM 0404002 (Sapucaí)
PONTO DE MEDIDA 4:PM 0404004 (Contorno)
DESDE:14/05/2015 00:00
ATÉ:15/05/201500:00

CARGA / 5 MINUTOS

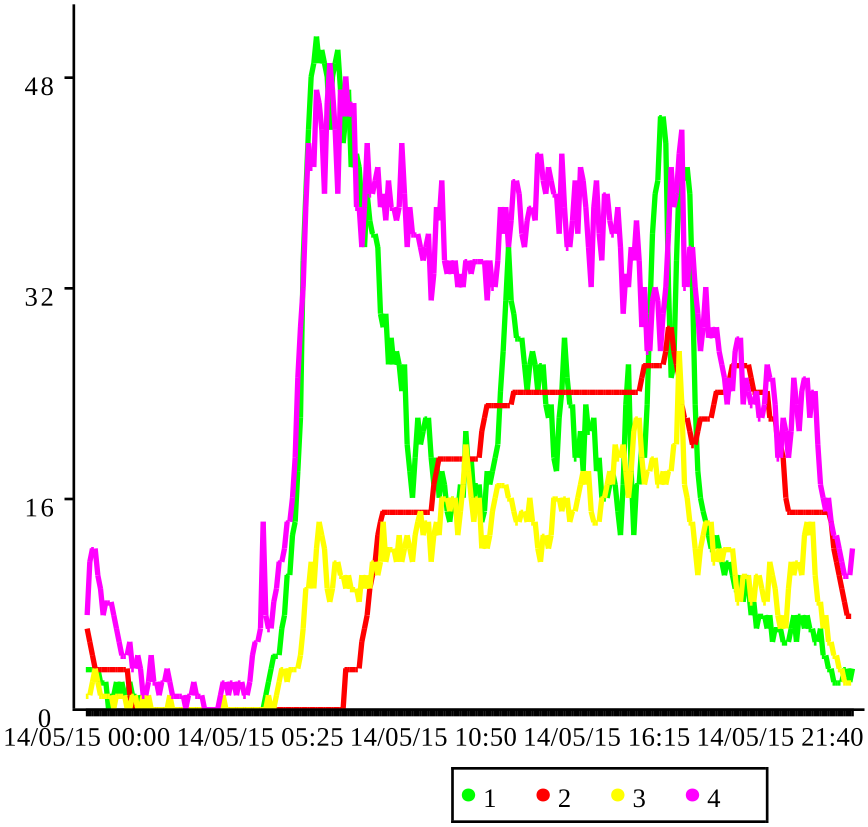

5 MINUTOS	CARGA			
	PM 0403004	PM 0403006	PM 0404002	PM 0404004
14/05/15 00:00	3	6	1	7
14/05/15 00:05	3	5	1	11
14/05/15 00:10	3	4	2	12
14/05/15 00:15	3	3	3	12
14/05/15 00:20	3	3	2	10
14/05/15 00:25	2	3	1	9
14/05/15 00:30	2	3	1	7
14/05/15 00:35	2	3	1	8
14/05/15 00:40	0	3	1	8
14/05/15 00:45	1	3	1	8
14/05/15 00:50	1	3	0	7
14/05/15 00:55	2	3	1	6
14/05/15 01:00	1	3	1	5
14/05/15 01:05	2	3	1	4
14/05/15 01:10	1	3	1	4

Sistema de Controle de Tráfego Urbano OPTIMUS

5 MINUTOS	CARGA			
	PM 0403004	PM 0403006	PM 0404002	PM 0404004
14/05/15 01:15	1	3	0	4
14/05/15 01:20	2	1	0	5
14/05/15 01:25	1	0	1	3
14/05/15 01:30	1	0	1	3
14/05/15 01:35	1	0	0	4
14/05/15 01:40	0	0	0	3
14/05/15 01:45	0	0	1	1
14/05/15 01:50	0	0	0	1
14/05/15 01:55	0	0	1	2
14/05/15 02:00	0	0	0	4
14/05/15 02:05	0	0	0	2
14/05/15 02:10	0	0	0	2
14/05/15 02:15	0	0	0	1
14/05/15 02:20	0	0	0	2
14/05/15 02:25	0	0	0	2
14/05/15 02:30	0	0	0	3
14/05/15 02:35	0	0	1	2
14/05/15 02:40	0	0	0	1
14/05/15 02:45	0	0	0	1
14/05/15 02:50	0	0	0	1
14/05/15 02:55	0	0	0	1
14/05/15 03:00	0	0	0	1
14/05/15 03:05	0	0	0	0
14/05/15 03:10	0	0	0	1
14/05/15 03:15	0	0	0	1
14/05/15 03:20	0	0	0	2
14/05/15 03:25	0	0	0	1
14/05/15 03:30	0	0	0	1
14/05/15 03:35	0	0	0	1
14/05/15 03:40	0	0	0	0
14/05/15 03:45	0	0	0	0
14/05/15 03:50	0	0	0	0
14/05/15 03:55	0	0	0	0
14/05/15 04:00	0	0	0	0
14/05/15 04:05	0	0	0	0
14/05/15 04:10	0	0	0	1
14/05/15 04:15	0	0	1	2
14/05/15 04:20	0	0	0	2
14/05/15 04:25	0	0	0	1
14/05/15 04:30	0	0	0	2
14/05/15 04:35	0	0	0	2
14/05/15 04:40	0	0	0	1
14/05/15 04:45	0	0	0	2
14/05/15 04:50	0	0	0	2
14/05/15 04:55	0	0	0	1
14/05/15 05:00	0	0	0	1
14/05/15 05:05	0	0	0	2
14/05/15 05:10	0	0	0	4
14/05/15 05:15	0	0	0	5
14/05/15 05:20	0	0	0	5
14/05/15 05:25	0	0	0	6
14/05/15 05:30	0	0	0	14
14/05/15 05:35	1	0	0	7
14/05/15 05:40	2	0	1	6
14/05/15 05:45	3	0	0	6
14/05/15 05:50	4	0	0	8
14/05/15 05:55	4	0	1	9
14/05/15 06:00	4	0	2	11

Sistema de Controle de Tráfego Urbano OPTIMUS

5 MINUTOS	CARGA			
	PM 040300 4	PM 040300 6	PM 040400 2	PM 040400 4
14/05/15 06:05	6	0	3	11
14/05/15 06:10	7	0	3	12
14/05/15 06:15	10	0	2	14
14/05/15 06:20	10	0	3	14
14/05/15 06:25	13	0	3	16
14/05/15 06:30	14	0	3	19
14/05/15 06:35	18	0	3	25
14/05/15 06:40	22	0	4	29
14/05/15 06:45	34	0	6	32
14/05/15 06:50	39	0	9	38
14/05/15 06:55	44	0	9	43
14/05/15 07:00	48	0	11	41
14/05/15 07:05	49	0	9	41
14/05/15 07:10	51	0	12	47
14/05/15 07:15	49	0	14	46
14/05/15 07:20	50	0	13	44
14/05/15 07:25	49	0	12	39
14/05/15 07:30	48	0	9	46
14/05/15 07:35	44	0	8	49
14/05/15 07:40	48	0	9	47
14/05/15 07:45	49	0	11	44
14/05/15 07:50	50	0	11	39
14/05/15 07:55	47	0	10	47
14/05/15 08:00	43	0	10	45
14/05/15 08:05	45	3	9	48
14/05/15 08:10	47	3	10	45
14/05/15 08:15	41	3	9	46
14/05/15 08:20	42	3	9	46
14/05/15 08:25	42	3	9	38
14/05/15 08:30	41	3	8	38
14/05/15 08:35	38	5	10	35
14/05/15 08:40	35	6	9	38
14/05/15 08:45	39	7	10	43
14/05/15 08:50	37	9	9	39
14/05/15 08:55	36	10	11	39
14/05/15 09:00	36	11	11	40
14/05/15 09:05	35	13	10	41
14/05/15 09:10	30	14	11	38
14/05/15 09:15	29	15	14	39
14/05/15 09:20	30	15	11	37
14/05/15 09:25	26	15	12	40
14/05/15 09:30	28	15	12	38
14/05/15 09:35	26	15	12	38
14/05/15 09:40	27	15	11	37
14/05/15 09:45	26	15	13	38
14/05/15 09:50	24	15	11	43
14/05/15 09:55	26	15	12	39
14/05/15 10:00	20	15	13	35
14/05/15 10:05	18	15	12	38
14/05/15 10:10	16	15	11	36
14/05/15 10:15	19	15	13	36
14/05/15 10:20	22	15	14	36
14/05/15 10:25	20	15	15	35
14/05/15 10:30	21	15	13	34
14/05/15 10:35	22	15	14	35
14/05/15 10:40	22	15	14	36
14/05/15 10:45	19	15	11	31
14/05/15 10:50	17	17	13	33

5 MINUTOS	CARGA			
	PM 040300 4	PM 040300 6	PM 040400 2	PM 040400 4
14/05/15 10:55	19	18	14	38
14/05/15 11:00	16	19	13	37
14/05/15 11:05	18	19	16	40
14/05/15 11:10	17	19	16	34
14/05/15 11:15	15	19	16	33
14/05/15 11:20	14	19	15	34
14/05/15 11:25	16	19	16	33
14/05/15 11:30	16	19	16	34
14/05/15 11:35	15	19	13	32
14/05/15 11:40	17	19	15	33
14/05/15 11:45	16	19	17	32
14/05/15 11:50	21	19	20	34
14/05/15 11:55	18	19	18	34
14/05/15 12:00	19	19	16	33
14/05/15 12:05	16	19	14	34
14/05/15 12:10	17	19	16	34
14/05/15 12:15	17	19	16	34
14/05/15 12:20	14	21	12	34
14/05/15 12:25	15	22	13	34
14/05/15 12:30	18	23	12	31
14/05/15 12:35	17	23	13	34
14/05/15 12:40	18	23	15	32
14/05/15 12:45	19	23	16	32
14/05/15 12:50	20	23	17	34
14/05/15 12:55	24	23	17	38
14/05/15 13:00	27	23	17	36
14/05/15 13:05	31	23	17	38
14/05/15 13:10	35	23	16	35
14/05/15 13:15	31	23	16	37
14/05/15 13:20	30	24	15	40
14/05/15 13:25	28	24	14	40
14/05/15 13:30	28	24	14	39
14/05/15 13:35	28	24	15	36
14/05/15 13:40	26	24	15	35
14/05/15 13:45	24	24	14	37
14/05/15 13:50	26	24	16	38
14/05/15 13:55	27	24	14	38
14/05/15 14:00	26	24	14	37
14/05/15 14:05	24	24	12	42
14/05/15 14:10	26	24	11	42
14/05/15 14:15	26	24	13	40
14/05/15 14:20	23	24	13	39
14/05/15 14:25	22	24	12	41
14/05/15 14:30	23	24	13	40
14/05/15 14:35	19	24	16	39
14/05/15 14:40	18	24	16	39
14/05/15 14:45	22	24	16	36
14/05/15 14:50	24	24	15	42
14/05/15 14:55	28	24	16	38
14/05/15 15:00	25	24	16	35
14/05/15 15:05	23	24	14	35
14/05/15 15:10	23	24	15	37
14/05/15 15:15	19	24	15	40
14/05/15 15:20	19	24	16	36
14/05/15 15:25	21	24	17	41
14/05/15 15:30	18	24	18	40
14/05/15 15:35	23	24	17	38
14/05/15 15:40	21	24	18	35

Sistema de Controle de Tráfego Urbano OPTIMUS

5 MINUTOS	CARGA			
	PM 0403004	PM 0403006	PM 0404002	PM 0404004
14/05/15 15:45	21	24	15	32
14/05/15 15:50	22	24	14	38
14/05/15 15:55	18	24	14	40
14/05/15 16:00	19	24	14	36
14/05/15 16:05	16	24	16	34
14/05/15 16:10	16	24	16	39
14/05/15 16:15	16	24	17	39
14/05/15 16:20	17	24	18	37
14/05/15 16:25	18	24	17	36
14/05/15 16:30	17	24	20	36
14/05/15 16:35	15	24	19	38
14/05/15 16:40	13	24	19	35
14/05/15 16:45	17	24	20	30
14/05/15 16:50	23	24	18	33
14/05/15 16:55	26	24	16	32
14/05/15 17:00	18	24	18	35
14/05/15 17:05	13	24	21	34
14/05/15 17:10	17	24	22	37
14/05/15 17:15	17	24	22	34
14/05/15 17:20	20	25	19	29
14/05/15 17:25	19	26	17	32
14/05/15 17:30	23	26	18	27
14/05/15 17:35	30	26	18	27
14/05/15 17:40	36	26	19	31
14/05/15 17:45	39	26	19	32
14/05/15 17:50	40	26	17	31
14/05/15 17:55	45	26	17	27
14/05/15 18:00	45	26	18	30
14/05/15 18:05	43	27	17	32
14/05/15 18:10	32	29	18	36
14/05/15 18:15	25	29	18	41
14/05/15 18:20	26	27	20	38
14/05/15 18:25	34	26	20	39
14/05/15 18:30	40	25	27	42
14/05/15 18:35	40	23	23	44
14/05/15 18:40	40	22	17	32
14/05/15 18:45	41	22	16	32
14/05/15 18:50	39	21	14	35
14/05/15 18:55	32	20	14	35
14/05/15 19:00	23	20	12	32
14/05/15 19:05	18	21	10	30
14/05/15 19:10	16	22	12	27
14/05/15 19:15	15	22	13	29
14/05/15 19:20	14	22	14	32
14/05/15 19:25	13	22	14	28
14/05/15 19:30	12	22	14	29
14/05/15 19:35	12	23	11	28
14/05/15 19:40	13	24	11	29
14/05/15 19:45	12	24	12	27
14/05/15 19:50	11	24	11	26
14/05/15 19:55	10	24	12	25
14/05/15 20:00	11	24	12	23
14/05/15 20:05	11	25	12	25
14/05/15 20:10	10	26	12	24
14/05/15 20:15	9	26	10	27
14/05/15 20:20	9	26	8	28
14/05/15 20:25	10	26	8	28
14/05/15 20:30	8	26	10	23

Sistema de Controle de Tráfego Urbano OPTIMUS

5 MINUTOS	CARGA			
	PM 0403004	PM 0403006	PM 0404002	PM 0404004
14/05/15 20:35	10	26	10	25
14/05/15 20:40	9	26	10	24
14/05/15 20:45	7	25	8	23
14/05/15 20:50	8	24	8	23
14/05/15 20:55	6	24	10	24
14/05/15 21:00	7	24	10	22
14/05/15 21:05	7	24	9	22
14/05/15 21:10	7	24	8	23
14/05/15 21:15	6	24	8	26
14/05/15 21:20	7	22	11	25
14/05/15 21:25	5	22	10	25
14/05/15 21:30	6	22	9	23
14/05/15 21:35	6	20	7	19
14/05/15 21:40	6	20	6	19
14/05/15 21:45	5	19	7	22
14/05/15 21:50	5	16	6	21
14/05/15 21:55	5	15	9	19
14/05/15 22:00	6	15	11	21
14/05/15 22:05	7	15	10	25
14/05/15 22:10	5	15	11	23
14/05/15 22:15	7	15	11	21
14/05/15 22:20	7	15	10	24
14/05/15 22:25	6	15	13	25
14/05/15 22:30	7	15	14	25
14/05/15 22:35	6	15	13	22
14/05/15 22:40	6	15	14	24
14/05/15 22:45	5	15	10	24
14/05/15 22:50	5	15	8	20
14/05/15 22:55	6	15	8	17
14/05/15 23:00	4	15	6	16
14/05/15 23:05	4	15	7	15
14/05/15 23:10	3	15	5	16
14/05/15 23:15	3	14	5	14
14/05/15 23:20	2	12	4	13
14/05/15 23:25	2	11	4	13
14/05/15 23:30	2	10	3	12
14/05/15 23:35	2	9	3	11
14/05/15 23:40	3	8	2	10
14/05/15 23:45	3	7	2	10
14/05/15 23:50	2	7	2	10
14/05/15 23:55	3	7	2	12
