# Supplementary material for: Traffic light optimization using non-dominated sorting genetic algorithm (NSGA2)
Source: Sci Rep. 2023 Sep 20;13:15550. doi: 10.1038/s41598-023-38884-2 (PMC10511403; doi:10.1038/s41598-023-38884-2)
Supplement: Supplementary file 1 — Supplementary Information. [file 41598_2023_38884_MOESM1_ESM.zip › dadosBHTrans/dados4]

Sistema de Controle de Tráfego Urbano OPTIMUS

CARGA DE 4 PONTOS DE MEDIDA
DADOS DE 5 MINUTOS

PONTO DE MEDIDA 1:PM 0105102 (Andradas)
PONTO DE MEDIDA 2:PM 0105108 (Contorno)
PONTO DE MEDIDA 3:PM 0105204 (Andradas)
PONTO DE MEDIDA 4:PM 0105303 (Contorno)
DESDE:14/05/2015 00:00
ATÉ:15/05/201500:00

CARGA / 5 MINUTOS

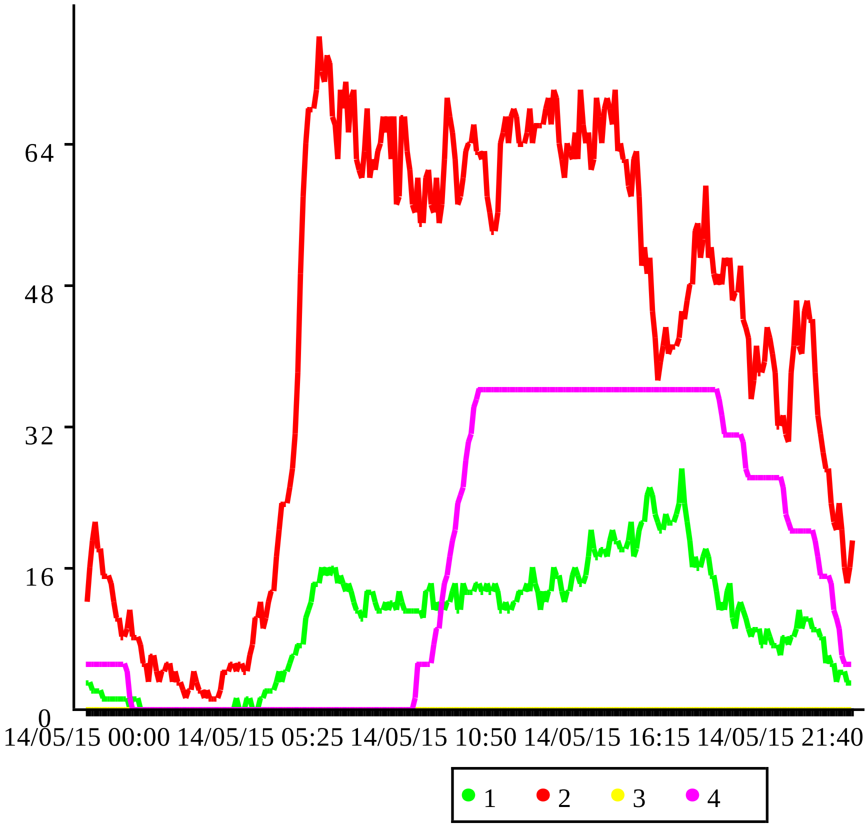

5 MINUTOS	CARGA			
	PM 0105102	PM 0105108	PM 0105204	PM 0105303
14/05/15 00:00	3	12	0	5
14/05/15 00:05	3	16	0	5
14/05/15 00:10	2	19	0	5
14/05/15 00:15	2	21	0	5
14/05/15 00:20	2	18	0	5
14/05/15 00:25	2	18	0	5
14/05/15 00:30	1	15	0	5
14/05/15 00:35	1	15	0	5
14/05/15 00:40	1	15	0	5
14/05/15 00:45	1	14	0	5
14/05/15 00:50	1	12	0	5
14/05/15 00:55	1	10	0	5
14/05/15 01:00	1	10	0	5
14/05/15 01:05	1	8	0	5
14/05/15 01:10	1	8	0	5

Sistema de Controle de Tráfego Urbano OPTIMUS

5 MINUTOS	CARGA			
	PM 010510 2	PM 010510 8	PM 010520 4	PM 010530 3
14/05/15 01:15	1	9	0	4
14/05/15 01:20	0	11	0	1
14/05/15 01:25	1	8	0	0
14/05/15 01:30	1	8	0	0
14/05/15 01:35	1	8	0	0
14/05/15 01:40	0	7	0	0
14/05/15 01:45	0	5	0	0
14/05/15 01:50	0	5	0	0
14/05/15 01:55	0	3	0	0
14/05/15 02:00	0	6	0	0
14/05/15 02:05	0	6	0	0
14/05/15 02:10	0	4	0	0
14/05/15 02:15	0	3	0	0
14/05/15 02:20	0	4	0	0
14/05/15 02:25	0	4	0	0
14/05/15 02:30	0	5	0	0
14/05/15 02:35	0	5	0	0
14/05/15 02:40	0	3	0	0
14/05/15 02:45	0	4	0	0
14/05/15 02:50	0	3	0	0
14/05/15 02:55	0	3	0	0
14/05/15 03:00	0	2	0	0
14/05/15 03:05	0	1	0	0
14/05/15 03:10	0	2	0	0
14/05/15 03:15	0	2	0	0
14/05/15 03:20	0	4	0	0
14/05/15 03:25	0	3	0	0
14/05/15 03:30	0	2	0	0
14/05/15 03:35	0	2	0	0
14/05/15 03:40	0	1	0	0
14/05/15 03:45	0	2	0	0
14/05/15 03:50	0	1	0	0
14/05/15 03:55	0	1	0	0
14/05/15 04:00	0	1	0	0
14/05/15 04:05	0	1	0	0
14/05/15 04:10	0	2	0	0
14/05/15 04:15	0	4	0	0
14/05/15 04:20	0	4	0	0
14/05/15 04:25	0	4	0	0
14/05/15 04:30	0	5	0	0
14/05/15 04:35	0	5	0	0
14/05/15 04:40	1	4	0	0
14/05/15 04:45	0	5	0	0
14/05/15 04:50	0	5	0	0
14/05/15 04:55	0	4	0	0
14/05/15 05:00	1	4	0	0
14/05/15 05:05	1	6	0	0
14/05/15 05:10	0	7	0	0
14/05/15 05:15	0	10	0	0
14/05/15 05:20	0	10	0	0
14/05/15 05:25	1	12	0	0
14/05/15 05:30	1	9	0	0
14/05/15 05:35	2	10	0	0
14/05/15 05:40	2	12	0	0
14/05/15 05:45	2	13	0	0
14/05/15 05:50	2	13	0	0
14/05/15 05:55	3	17	0	0
14/05/15 06:00	4	20	0	0

Sistema de Controle de Tráfego Urbano OPTIMUS

5 MINUTOS	CARGA			
	PM 0105102	PM 0105108	PM 0105204	PM 0105303
14/05/15 06:05	3	23	0	0
14/05/15 06:10	4	23	0	0
14/05/15 06:15	4	23	0	0
14/05/15 06:20	5	25	0	0
14/05/15 06:25	6	27	0	0
14/05/15 06:30	6	31	0	0
14/05/15 06:35	7	38	0	0
14/05/15 06:40	7	49	0	0
14/05/15 06:45	7	58	0	0
14/05/15 06:50	10	64	0	0
14/05/15 06:55	11	68	0	0
14/05/15 07:00	12	68	0	0
14/05/15 07:05	14	68	0	0
14/05/15 07:10	14	70	0	0
14/05/15 07:15	14	76	0	0
14/05/15 07:20	16	72	0	0
14/05/15 07:25	15	71	0	0
14/05/15 07:30	16	74	0	0
14/05/15 07:35	15	73	0	0
14/05/15 07:40	16	67	0	0
14/05/15 07:45	16	66	0	0
14/05/15 07:50	14	62	0	0
14/05/15 07:55	15	70	0	0
14/05/15 08:00	14	68	0	0
14/05/15 08:05	13	71	0	0
14/05/15 08:10	14	65	0	0
14/05/15 08:15	13	69	0	0
14/05/15 08:20	12	70	0	0
14/05/15 08:25	11	62	0	0
14/05/15 08:30	11	61	0	0
14/05/15 08:35	10	60	0	0
14/05/15 08:40	10	63	0	0
14/05/15 08:45	13	68	0	0
14/05/15 08:50	13	60	0	0
14/05/15 08:55	13	62	0	0
14/05/15 09:00	12	61	0	0
14/05/15 09:05	11	63	0	0
14/05/15 09:10	11	64	0	0
14/05/15 09:15	11	67	0	0
14/05/15 09:20	12	65	0	0
14/05/15 09:25	11	67	0	0
14/05/15 09:30	12	62	0	0
14/05/15 09:35	12	67	0	0
14/05/15 09:40	11	57	0	0
14/05/15 09:45	13	58	0	0
14/05/15 09:50	12	67	0	0
14/05/15 09:55	11	67	0	0
14/05/15 10:00	11	63	0	0
14/05/15 10:05	11	61	0	0
14/05/15 10:10	11	57	0	0
14/05/15 10:15	11	56	0	1
14/05/15 10:20	11	60	0	5
14/05/15 10:25	11	55	0	5
14/05/15 10:30	10	55	0	5
14/05/15 10:35	13	60	0	5
14/05/15 10:40	13	61	0	5
14/05/15 10:45	14	57	0	5
14/05/15 10:50	11	56	0	7

Sistema de Controle de Tráfego Urbano OPTIMUS

5 MINUTOS	CARGA			
	PM 010510 2	PM 010510 8	PM 010520 4	PM 010530 3
14/05/15 10:55	12	60	0	9
14/05/15 11:00	11	55	0	9
14/05/15 11:05	11	57	0	12
14/05/15 11:10	11	62	0	14
14/05/15 11:15	12	69	0	15
14/05/15 11:20	12	67	0	17
14/05/15 11:25	13	65	0	19
14/05/15 11:30	14	62	0	20
14/05/15 11:35	11	57	0	23
14/05/15 11:40	11	58	0	24
14/05/15 11:45	14	60	0	25
14/05/15 11:50	13	63	0	28
14/05/15 11:55	13	64	0	30
14/05/15 12:00	13	64	0	31
14/05/15 12:05	13	66	0	34
14/05/15 12:10	14	63	0	35
14/05/15 12:15	14	63	0	36
14/05/15 12:20	13	62	0	36
14/05/15 12:25	13	63	0	36
14/05/15 12:30	14	58	0	36
14/05/15 12:35	13	56	0	36
14/05/15 12:40	13	54	0	36
14/05/15 12:45	14	54	0	36
14/05/15 12:50	13	56	0	36
14/05/15 12:55	11	64	0	36
14/05/15 13:00	11	65	0	36
14/05/15 13:05	12	67	0	36
14/05/15 13:10	11	64	0	36
14/05/15 13:15	11	67	0	36
14/05/15 13:20	12	68	0	36
14/05/15 13:25	12	67	0	36
14/05/15 13:30	13	64	0	36
14/05/15 13:35	13	64	0	36
14/05/15 13:40	13	64	0	36
14/05/15 13:45	14	65	0	36
14/05/15 13:50	13	68	0	36
14/05/15 13:55	16	64	0	36
14/05/15 14:00	14	66	0	36
14/05/15 14:05	13	66	0	36
14/05/15 14:10	11	66	0	36
14/05/15 14:15	13	66	0	36
14/05/15 14:20	12	68	0	36
14/05/15 14:25	13	69	0	36
14/05/15 14:30	13	66	0	36
14/05/15 14:35	16	70	0	36
14/05/15 14:40	15	69	0	36
14/05/15 14:45	15	64	0	36
14/05/15 14:50	13	62	0	36
14/05/15 14:55	12	60	0	36
14/05/15 15:00	13	64	0	36
14/05/15 15:05	13	63	0	36
14/05/15 15:10	15	62	0	36
14/05/15 15:15	16	65	0	36
14/05/15 15:20	15	62	0	36
14/05/15 15:25	14	70	0	36
14/05/15 15:30	14	66	0	36
14/05/15 15:35	15	64	0	36
14/05/15 15:40	17	65	0	36

Sistema de Controle de Tráfego Urbano OPTIMUS

5 MINUTOS	CARGA			
	PM 0105102	PM 0105108	PM 0105204	PM 0105303
14/05/15 15:45	20	61	0	36
14/05/15 15:50	18	62	0	36
14/05/15 15:55	17	69	0	36
14/05/15 16:00	17	67	0	36
14/05/15 16:05	18	64	0	36
14/05/15 16:10	18	68	0	36
14/05/15 16:15	17	69	0	36
14/05/15 16:20	19	68	0	36
14/05/15 16:25	20	66	0	36
14/05/15 16:30	19	70	0	36
14/05/15 16:35	19	63	0	36
14/05/15 16:40	18	64	0	36
14/05/15 16:45	18	62	0	36
14/05/15 16:50	18	62	0	36
14/05/15 16:55	19	59	0	36
14/05/15 17:00	21	58	0	36
14/05/15 17:05	17	62	0	36
14/05/15 17:10	18	63	0	36
14/05/15 17:15	20	58	0	36
14/05/15 17:20	21	50	0	36
14/05/15 17:25	21	52	0	36
14/05/15 17:30	24	49	0	36
14/05/15 17:35	25	51	0	36
14/05/15 17:40	24	45	0	36
14/05/15 17:45	22	42	0	36
14/05/15 17:50	21	37	0	36
14/05/15 17:55	20	39	0	36
14/05/15 18:00	20	41	0	36
14/05/15 18:05	22	43	0	36
14/05/15 18:10	21	40	0	36
14/05/15 18:15	21	41	0	36
14/05/15 18:20	21	41	0	36
14/05/15 18:25	22	41	0	36
14/05/15 18:30	23	42	0	36
14/05/15 18:35	27	45	0	36
14/05/15 18:40	23	44	0	36
14/05/15 18:45	21	46	0	36
14/05/15 18:50	19	48	0	36
14/05/15 18:55	16	48	0	36
14/05/15 19:00	17	54	0	36
14/05/15 19:05	16	55	0	36
14/05/15 19:10	16	51	0	36
14/05/15 19:15	17	53	0	36
14/05/15 19:20	18	59	0	36
14/05/15 19:25	17	51	0	36
14/05/15 19:30	15	52	0	36
14/05/15 19:35	15	49	0	36
14/05/15 19:40	13	48	0	36
14/05/15 19:45	11	49	0	35
14/05/15 19:50	12	48	0	33
14/05/15 19:55	11	51	0	31
14/05/15 20:00	13	50	0	31
14/05/15 20:05	14	51	0	31
14/05/15 20:10	10	46	0	31
14/05/15 20:15	9	47	0	31
14/05/15 20:20	11	47	0	31
14/05/15 20:25	12	50	0	31
14/05/15 20:30	11	44	0	30

Sistema de Controle de Tráfego Urbano OPTIMUS

5 MINUTOS	CARGA			
	PM 0105102	PM 0105108	PM 0105204	PM 0105303
14/05/15 20:35	10	43	0	27
14/05/15 20:40	9	42	0	26
14/05/15 20:45	8	35	0	26
14/05/15 20:50	9	37	0	26
14/05/15 20:55	9	41	0	26
14/05/15 21:00	9	38	0	26
14/05/15 21:05	7	38	0	26
14/05/15 21:10	7	39	0	26
14/05/15 21:15	9	43	0	26
14/05/15 21:20	8	42	0	26
14/05/15 21:25	7	40	0	26
14/05/15 21:30	7	38	0	26
14/05/15 21:35	7	32	0	26
14/05/15 21:40	6	32	0	26
14/05/15 21:45	8	33	0	25
14/05/15 21:50	8	31	0	22
14/05/15 21:55	7	30	0	21
14/05/15 22:00	8	38	0	20
14/05/15 22:05	8	41	0	20
14/05/15 22:10	9	46	0	20
14/05/15 22:15	11	41	0	20
14/05/15 22:20	9	40	0	20
14/05/15 22:25	10	45	0	20
14/05/15 22:30	10	46	0	20
14/05/15 22:35	10	44	0	20
14/05/15 22:40	9	44	0	20
14/05/15 22:45	9	38	0	19
14/05/15 22:50	9	33	0	17
14/05/15 22:55	8	31	0	15
14/05/15 23:00	8	29	0	15
14/05/15 23:05	5	27	0	15
14/05/15 23:10	6	27	0	15
14/05/15 23:15	5	23	0	14
14/05/15 23:20	5	21	0	11
14/05/15 23:25	3	20	0	10
14/05/15 23:30	4	23	0	9
14/05/15 23:35	4	20	0	6
14/05/15 23:40	4	16	0	5
14/05/15 23:45	3	14	0	5
14/05/15 23:50	3	16	0	5
14/05/15 23:55	3	19	0	5
