# Supplementary material for: Traffic light optimization using non-dominated sorting genetic algorithm (NSGA2)
Source: Sci Rep. 2023 Sep 20;13:15550. doi: 10.1038/s41598-023-38884-2 (PMC10511403; doi:10.1038/s41598-023-38884-2)
Supplement: Supplementary file 1 — Supplementary Information. [file 41598_2023_38884_MOESM1_ESM.zip › dadosBHTrans/Dados-BHTRANS02/I_1_dat_4_cs_5_min_st-1.pdf]

Sistema de Controle de Tráfego Urbano OPTIMUS

INTENSIDADE DE 4 PONTOS DE MEDIDA
DADOS DE 5 MINUTOS

PONTO DE MEDIDA 1:PM 04010 03 (Pouso Alegre)
PONTO DE MEDIDA 2:PM 04010 04 (Pouso Alegre)
PONTO DE MEDIDA 3:PM 04010 06 (Curvelo)
PONTO DE MEDIDA 4:PM 04020 01 (Itajubá)
DESDE:01/05/2015 16:21
ATÉ:01/06/201516:21

INTENSIDADE / 5 MINUTOS

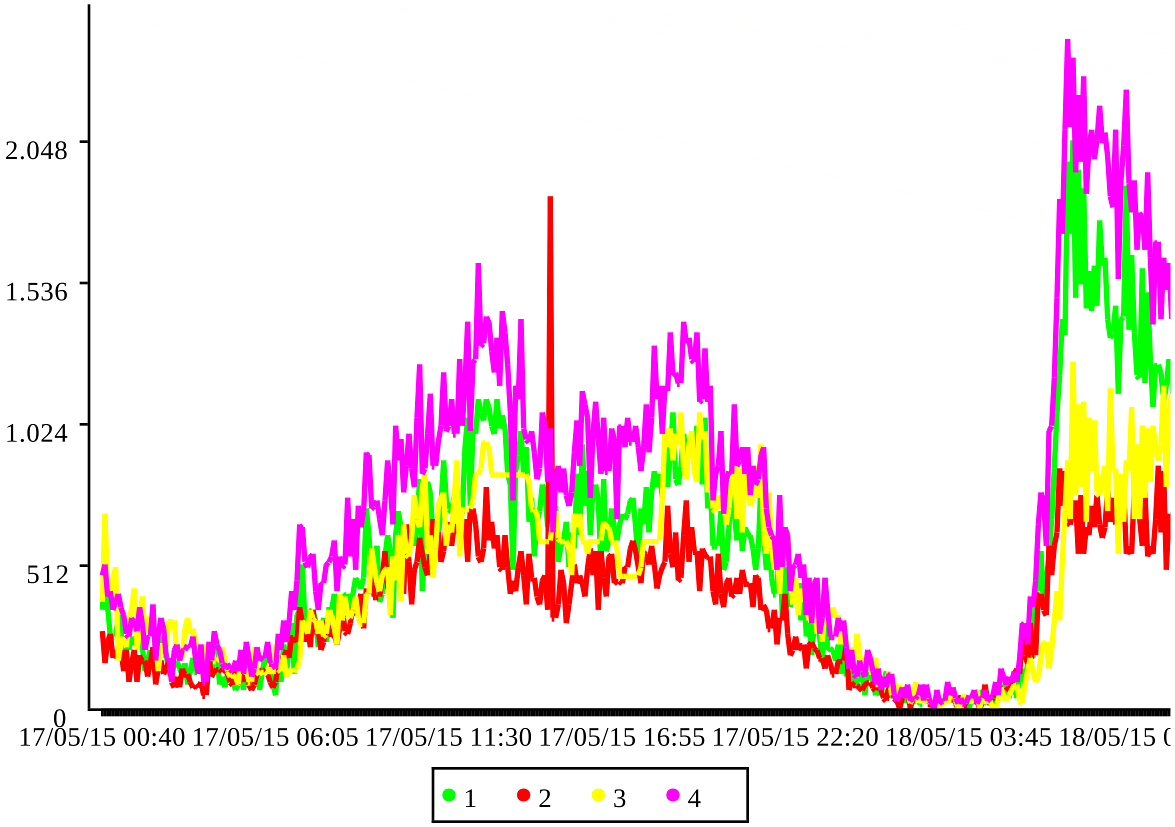

5 MINUTOS	INTENSIDADE			
	PM 0401003	PM 0401004	PM 0401006	PM 0402001
17/05/15 00:40	348	276	384	480
17/05/15 00:45	504	156	696	516
17/05/15 00:50	360	240	432	396
17/05/15 00:55	252	264	420	420
17/05/15 01:00	252	180	468	348
17/05/15 01:05	252	204	504	384
17/05/15 01:10	336	192	168	408
17/05/15 01:15	264	204	240	360
17/05/15 01:20	240	132	252	336
17/05/15 01:25	204	204	216	264
17/05/15 01:30	204	96	324	252
17/05/15 01:35	252	156	348	324
17/05/15 01:40	228	204	432	312
17/05/15 01:45	240	96	204	276
17/05/15 01:50	252	192	288	360

Sistema de Controle de Tráfego Urbano OPTIMUS

5 MINUTOS	INTENSIDADE			
	PM 0401003	PM 0401004	PM 0401006	PM 0402001
17/05/15 01:55	216	168	396	300
17/05/15 02:00	132	168	264	204
17/05/15 02:05	168	108	324	252
17/05/15 02:10	204	156	324	252
17/05/15 02:15	192	216	288	372
17/05/15 02:20	132	84	228	168
17/05/15 02:25	216	144	132	264
17/05/15 02:30	228	168	228	324
17/05/15 02:35	252	120	228	288
17/05/15 02:40	192	144	216	180
17/05/15 02:45	132	168	312	180
17/05/15 02:50	108	96	312	96
17/05/15 02:55	156	72	312	204
17/05/15 03:00	168	108	180	228
17/05/15 03:05	144	72	216	168
17/05/15 03:10	120	144	180	204
17/05/15 03:15	156	120	288	204
17/05/15 03:20	84	120	324	228
17/05/15 03:25	132	96	264	216
17/05/15 03:30	180	72	288	252
17/05/15 03:35	132	84	240	168
17/05/15 03:40	132	72	204	120
17/05/15 03:45	156	96	132	228
17/05/15 03:50	132	48	120	96
17/05/15 03:55	120	48	228	96
17/05/15 04:00	144	180	168	240
17/05/15 04:05	120	108	204	168
17/05/15 04:10	240	132	168	276
17/05/15 04:15	192	144	168	228
17/05/15 04:20	84	144	180	204
17/05/15 04:25	108	132	216	156
17/05/15 04:30	72	144	168	144
17/05/15 04:35	120	120	132	156
17/05/15 04:40	108	96	108	144
17/05/15 04:45	96	84	120	132
17/05/15 04:50	60	144	108	168
17/05/15 04:55	84	84	84	132
17/05/15 05:00	132	132	192	204
17/05/15 05:05	60	96	120	120
17/05/15 05:10	180	144	144	240
17/05/15 05:15	168	84	96	180
17/05/15 05:20	60	60	96	108
17/05/15 05:25	120	84	216	144
17/05/15 05:30	132	120	132	216
17/05/15 05:35	60	120	120	168
17/05/15 05:40	132	144	132	192
17/05/15 05:45	180	132	132	180
17/05/15 05:50	192	120	120	240
17/05/15 05:55	132	96	168	156
17/05/15 06:00	84	72	120	156
17/05/15 06:05	48	96	228	144
17/05/15 06:10	192	156	144	264
17/05/15 06:15	96	180	132	204
17/05/15 06:20	228	180	192	312
17/05/15 06:25	120	204	108	240
17/05/15 06:30	204	180	132	300
17/05/15 06:35	300	252	144	420
17/05/15 06:40	120	252	132	348

Sistema de Controle de Tráfego Urbano OPTIMUS

5 MINUTOS	INTENSIDADE			
	PM 0401003	PM 0401004	PM 0401006	PM 0402001
17/05/15 06:45	336	240	144	456
17/05/15 06:50	348	360	168	660
17/05/15 06:55	516	300	312	648
17/05/15 07:00	300	240	300	528
17/05/15 07:05	300	300	264	504
17/05/15 07:10	348	216	348	540
17/05/15 07:15	348	348	324	552
17/05/15 07:20	312	276	312	420
17/05/15 07:25	216	276	264	348
17/05/15 07:30	264	204	312	444
17/05/15 07:35	324	240	252	444
17/05/15 07:40	240	312	264	516
17/05/15 07:45	360	288	348	528
17/05/15 07:50	348	252	336	540
17/05/15 07:55	408	264	288	600
17/05/15 08:00	264	228	228	420
17/05/15 08:05	324	288	408	540
17/05/15 08:10	324	360	312	504
17/05/15 08:15	408	276	396	516
17/05/15 08:20	408	264	336	756
17/05/15 08:25	348	300	324	540
17/05/15 08:30	408	360	348	684
17/05/15 08:35	468	336	396	492
17/05/15 08:40	456	384	324	732
17/05/15 08:45	432	408	300	648
17/05/15 08:50	468	288	300	708
17/05/15 08:55	720	432	408	924
17/05/15 09:00	624	408	408	912
17/05/15 09:05	444	468	576	720
17/05/15 09:10	516	396	504	732
17/05/15 09:15	588	396	444	744
17/05/15 09:20	384	396	420	684
17/05/15 09:25	408	468	480	624
17/05/15 09:30	540	564	492	744
17/05/15 09:35	624	432	504	888
17/05/15 09:40	564	480	336	792
17/05/15 09:45	324	492	540	660
17/05/15 09:50	624	528	456	1020
17/05/15 09:55	708	516	624	888
17/05/15 10:00	648	528	384	972
17/05/15 10:05	516	408	660	780
17/05/15 10:10	600	660	552	876
17/05/15 10:15	600	528	540	984
17/05/15 10:20	624	372	612	864
17/05/15 10:25	588	492	768	792
17/05/15 10:30	672	528	684	1044
17/05/15 10:35	828	612	648	1236
17/05/15 10:40	420	552	792	840
17/05/15 10:45	600	504	840	864
17/05/15 10:50	816	480	552	984
17/05/15 10:55	780	564	624	1128
17/05/15 11:00	612	684	468	876
17/05/15 11:05	576	552	576	876
17/05/15 11:10	612	636	732	960
17/05/15 11:15	732	528	756	1020
17/05/15 11:20	888	564	780	1212
17/05/15 11:25	744	672	588	996
17/05/15 11:30	720	672	660	1020

Sistema de Controle de Tráfego Urbano OPTIMUS

5 MINUTOS	INTENSIDADE			
	PM 0401003	PM 0401004	PM 0401006	PM 0402001
17/05/15 11:35	708	588	636	1116
17/05/15 11:40	768	660	768	984
17/05/15 11:45	792	648	888	984
17/05/15 11:50	744	624	540	1260
17/05/15 11:55	696	648	720	1020
17/05/15 12:00	936	684	720	1176
17/05/15 12:05	1044	528	720	1392
17/05/15 12:10	732	720	720	996
17/05/15 12:15	1008	720	744	1248
17/05/15 12:20	984	660	840	1260
17/05/15 12:25	1116	540	840	1608
17/05/15 12:30	1068	528	864	1308
17/05/15 12:35	1032	576	960	1320
17/05/15 12:40	1116	792	960	1416
17/05/15 12:45	1080	600	936	1392
17/05/15 12:50	1020	672	840	1284
17/05/15 12:55	984	576	840	1212
17/05/15 13:00	1116	624	840	1332
17/05/15 13:05	1008	504	840	1164
17/05/15 13:10	1056	492	840	1428
17/05/15 13:15	1008	624	840	1356
17/05/15 13:20	840	504	840	1236
17/05/15 13:25	804	408	840	1068
17/05/15 13:30	492	456	840	744
17/05/15 13:35	852	420	840	1164
17/05/15 13:40	912	504	840	1116
17/05/15 13:45	996	564	840	1404
17/05/15 13:50	840	480	840	1008
17/05/15 13:55	936	372	840	972
17/05/15 14:00	672	552	816	960
17/05/15 14:05	756	468	720	996
17/05/15 14:10	540	468	720	924
17/05/15 14:15	672	396	696	828
17/05/15 14:20	708	372	600	864
17/05/15 14:25	804	456	600	1068
17/05/15 14:30	780	480	600	1020
17/05/15 14:35	648	348	600	816
17/05/15 14:40	1236	1848	600	1008
17/05/15 14:45	612	324	684	636
17/05/15 14:50	732	324	708	828
17/05/15 14:55	528	372	612	876
17/05/15 15:00	588	492	600	780
17/05/15 15:05	600	444	600	864
17/05/15 15:10	672	300	600	768
17/05/15 15:15	504	372	576	732
17/05/15 15:20	636	444	480	780
17/05/15 15:25	576	516	696	924
17/05/15 15:30	840	444	648	1032
17/05/15 15:35	624	480	696	876
17/05/15 15:40	948	444	600	1140
17/05/15 15:45	732	396	600	1092
17/05/15 15:50	852	504	552	1044
17/05/15 15:55	624	576	600	756
17/05/15 16:00	792	480	600	984
17/05/15 16:05	804	564	600	1104
17/05/15 16:10	744	348	600	972
17/05/15 16:15	504	564	600	840
17/05/15 16:20	828	468	672	1044

Sistema de Controle de Tráfego Urbano OPTIMUS

5 MINUTOS	INTENSIDADE			
	PM 0401003	PM 0401004	PM 0401006	PM 0402001
17/05/15 16:25	564	396	660	852
17/05/15 16:30	600	540	648	852
17/05/15 16:35	720	552	600	1008
17/05/15 16:40	648	444	600	972
17/05/15 16:45	600	456	576	684
17/05/15 16:50	660	456	480	1020
17/05/15 16:55	696	444	480	1020
17/05/15 17:00	684	504	480	936
17/05/15 17:05	696	504	480	1044
17/05/15 17:10	744	576	480	960
17/05/15 17:15	756	600	480	960
17/05/15 17:20	648	564	480	1020
17/05/15 17:25	516	504	480	936
17/05/15 17:30	720	444	504	852
17/05/15 17:35	660	564	600	936
17/05/15 17:40	792	552	600	1092
17/05/15 17:45	636	516	600	924
17/05/15 17:50	780	588	600	1044
17/05/15 17:55	852	516	600	1308
17/05/15 18:00	792	432	600	1116
17/05/15 18:05	840	480	600	1164
17/05/15 18:10	756	504	756	984
17/05/15 18:15	948	528	1008	1164
17/05/15 18:20	792	732	936	1140
17/05/15 18:25	924	552	1008	1356
17/05/15 18:30	1068	504	888	1200
17/05/15 18:35	828	636	984	1212
17/05/15 18:40	804	456	948	1176
17/05/15 18:45	876	468	1068	1176
17/05/15 18:50	984	624	864	1392
17/05/15 18:55	912	744	828	1320
17/05/15 19:00	924	528	948	1332
17/05/15 19:05	924	648	996	1260
17/05/15 19:10	900	564	852	1248
17/05/15 19:15	1020	564	816	1356
17/05/15 19:20	924	420	1068	1104
17/05/15 19:25	804	576	972	1116
17/05/15 19:30	1044	552	996	1296
17/05/15 19:35	720	540	780	1104
17/05/15 19:40	804	540	864	1164
17/05/15 19:45	588	420	720	744
17/05/15 19:50	588	372	708	840
17/05/15 19:55	588	552	768	876
17/05/15 20:00	708	408	720	996
17/05/15 20:05	492	360	744	696
17/05/15 20:10	528	444	660	828
17/05/15 20:15	648	468	720	852
17/05/15 20:20	852	396	828	840
17/05/15 20:25	696	408	876	1092
17/05/15 20:30	600	468	684	876
17/05/15 20:35	744	408	864	888
17/05/15 20:40	564	492	636	936
17/05/15 20:45	648	444	840	840
17/05/15 20:50	636	444	804	936
17/05/15 20:55	624	444	732	768
17/05/15 21:00	576	360	756	876
17/05/15 21:05	492	480	876	804
17/05/15 21:10	576	468	756	828

Sistema de Controle de Tráfego Urbano OPTIMUS

5 MINUTOS	INTENSIDADE			
	PM 0401003	PM 0401004	PM 0401006	PM 0402001
17/05/15 21:15	816	348	948	912
17/05/15 21:20	624	372	636	936
17/05/15 21:25	492	348	552	720
17/05/15 21:30	612	288	780	648
17/05/15 21:35	468	288	576	624
17/05/15 21:40	408	348	492	612
17/05/15 21:45	408	228	624	492
17/05/15 21:50	636	324	408	768
17/05/15 21:55	324	312	636	504
17/05/15 22:00	504	408	540	648
17/05/15 22:05	456	264	636	624
17/05/15 22:10	360	192	444	420
17/05/15 22:15	420	204	372	504
17/05/15 22:20	408	252	468	516
17/05/15 22:25	444	204	336	552
17/05/15 22:30	312	228	432	468
17/05/15 22:35	444	228	384	516
17/05/15 22:40	252	144	396	336
17/05/15 22:45	300	228	312	468
17/05/15 22:50	192	240	384	300
17/05/15 22:55	300	216	432	444
17/05/15 23:00	300	204	264	468
17/05/15 23:05	252	180	348	264
17/05/15 23:10	156	180	240	324
17/05/15 23:15	384	144	360	468
17/05/15 23:20	204	192	300	348
17/05/15 23:25	216	156	336	252
17/05/15 23:30	204	120	360	252
17/05/15 23:35	168	120	324	252
17/05/15 23:40	228	168	336	324
17/05/15 23:45	120	168	228	276
17/05/15 23:50	276	168	312	312
17/05/15 23:55	108	228	240	216
18/05/15 00:00	132	60	144	144
18/05/15 00:05	96	96	216	204
18/05/15 00:10	120	84	168	120
18/05/15 00:15	84	84	264	108
18/05/15 00:20	96	72	192	168
18/05/15 00:25	120	60	168	132
18/05/15 00:30	48	84	156	108
18/05/15 00:35	144	96	156	204
18/05/15 00:40	120	96	132	180
18/05/15 00:45	96	72	132	132
18/05/15 00:50	48	60	180	84
18/05/15 00:55	84	96	72	144
18/05/15 01:00	48	60	108	60
18/05/15 01:05	96	36	72	108
18/05/15 01:10	132	24	96	72
18/05/15 01:15	36	84	132	120
18/05/15 01:20	72	72	48	120
18/05/15 01:25	48	36	96	48
18/05/15 01:30	48	24	24	24
18/05/15 01:35	48	0	84	48
18/05/15 01:40	72	36	48	72
18/05/15 01:45	12	36	72	36
18/05/15 01:50	72	60	60	84
18/05/15 01:55	36	0	12	24
18/05/15 02:00	12	36	36	36

Sistema de Controle de Tráfego Urbano OPTIMUS

5 MINUTOS	INTENSIDADE			
	PM 0401003	PM 0401004	PM 0401006	PM 0402001
18/05/15 02:05	36	36	96	36
18/05/15 02:10	24	36	12	48
18/05/15 02:15	12	60	60	84
18/05/15 02:20	12	24	12	24
18/05/15 02:25	48	60	36	84
18/05/15 02:30	36	12	24	36
18/05/15 02:35	0	12	24	12
18/05/15 02:40	12	0	0	0
18/05/15 02:45	36	24	12	60
18/05/15 02:50	12	12	0	12
18/05/15 02:55	36	24	24	24
18/05/15 03:00	12	12	24	24
18/05/15 03:05	72	48	12	96
18/05/15 03:10	24	36	48	48
18/05/15 03:15	36	48	60	72
18/05/15 03:20	24	12	24	36
18/05/15 03:25	12	0	0	12
18/05/15 03:30	0	48	0	36
18/05/15 03:35	12	0	48	12
18/05/15 03:40	12	24	48	12
18/05/15 03:45	0	36	12	48
18/05/15 03:50	36	36	48	36
18/05/15 03:55	48	24	12	60
18/05/15 04:00	24	24	60	12
18/05/15 04:05	60	0	0	36
18/05/15 04:10	48	12	48	36
18/05/15 04:15	24	84	24	60
18/05/15 04:20	12	24	24	24
18/05/15 04:25	36	12	24	36
18/05/15 04:30	12	36	0	48
18/05/15 04:35	36	84	0	96
18/05/15 04:40	24	72	24	48
18/05/15 04:45	84	72	72	144
18/05/15 04:50	48	60	48	108
18/05/15 04:55	96	60	24	84
18/05/15 05:00	36	72	48	108
18/05/15 05:05	72	72	60	96
18/05/15 05:10	72	120	84	132
18/05/15 05:15	36	144	84	96
18/05/15 05:20	120	120	24	180
18/05/15 05:25	144	252	12	300
18/05/15 05:30	84	192	84	300
18/05/15 05:35	156	156	132	228
18/05/15 05:40	252	300	180	396
18/05/15 05:45	180	192	132	360
18/05/15 05:50	432	192	96	456
18/05/15 05:55	420	408	108	660
18/05/15 06:00	564	408	180	780
18/05/15 06:05	348	348	240	744
18/05/15 06:10	456	336	240	588
18/05/15 06:15	540	588	144	996
18/05/15 06:20	684	480	204	1020
18/05/15 06:25	864	588	312	1188
18/05/15 06:30	1068	636	420	1476
18/05/15 06:35	1200	864	312	1836
18/05/15 06:40	1404	732	528	1716
18/05/15 06:45	1344	852	708	2088
18/05/15 06:50	1968	672	888	2412

Sistema de Controle de Tráfego Urbano OPTIMUS

5 MINUTOS	INTENSIDADE			
	PM 040100 3	PM 040100 4	PM 040100 6	PM 040200 1
18/05/15 06:55	1716	660	684	2100
18/05/15 07:00	2052	708	1248	2352
18/05/15 07:05	1476	744	768	1932
18/05/15 07:10	1944	552	1092	2208
18/05/15 07:15	1524	768	792	1968
18/05/15 07:20	1872	552	1104	2280
18/05/15 07:25	1440	648	672	1860
18/05/15 07:30	1572	624	1044	2028
18/05/15 07:35	1428	732	852	2088
18/05/15 07:40	1596	648	1032	1980
18/05/15 07:45	1452	768	780	2052
18/05/15 07:50	1764	660	768	2172
18/05/15 07:55	1596	612	828	2040
18/05/15 08:00	1620	648	876	2076
18/05/15 08:05	1404	792	708	1992
18/05/15 08:10	1332	672	1152	1848
18/05/15 08:15	1356	756	852	1812
18/05/15 08:20	1452	660	864	2088
18/05/15 08:25	1128	732	552	1548
18/05/15 08:30	1404	720	840	1908
18/05/15 08:35	1416	792	672	2028
18/05/15 08:40	1884	552	888	2232
18/05/15 08:45	1368	588	852	1896
18/05/15 08:50	1632	552	1080	1788
18/05/15 08:55	1356	792	684	1908
18/05/15 09:00	1200	744	948	1656
18/05/15 09:05	1188	684	684	1788
18/05/15 09:10	1584	588	1020	1776
18/05/15 09:15	1176	780	756	1656
18/05/15 09:20	1500	540	1008	1932
18/05/15 09:25	1260	588	816	1620
18/05/15 09:30	1080	552	1020	1380
18/05/15 09:35	1236	684	936	1680
18/05/15 09:40	1236	876	888	1680
18/05/15 09:45	1212	636	948	1404
18/05/15 09:50	1104	852	1164	1620
18/05/15 09:55	1164	492	792	1512
18/05/15 10:00	1260	696	1068	1608
18/05/15 10:05	1188	660	1128	1404
18/05/15 10:10	1056	744	912	1728
18/05/15 10:15	912	828	864	1356
18/05/15 10:20	1020	612	1116	1572
18/05/15 10:25	1032	732	840	1500
18/05/15 10:30	936	660	1056	1488
18/05/15 10:35	912	648	948	1236
18/05/15 10:40	984	696	1044	1512
18/05/15 10:45	984	648	828	1248
18/05/15 10:50	984	708	996	1560
18/05/15 10:55	984	708	924	1092
18/05/15 11:00	1032	780	1044	1512
18/05/15 11:05	828	612	696	1164
18/05/15 11:10	1068	780	888	1500
18/05/15 11:15	1068	684	1140	1548
18/05/15 11:20	1092	684	924	1404
18/05/15 11:25	996	636	1248	1392
18/05/15 11:30	1272	588	1392	1440
18/05/15 11:35	1224	696	1104	1404
18/05/15 11:40	948	660	1464	1404

Sistema de Controle de Tráfego Urbano OPTIMUS

5 MINUTOS	INTENSIDADE			
	PM 040100 3	PM 040100 4	PM 040100 6	PM 040200 1
18/05/15 11:45	876	768	1260	1296
18/05/15 11:50	1296	732	1104	1332
18/05/15 11:55	1428	588	1356	1764
18/05/15 12:00	1404	696	1308	1812
18/05/15 12:05	1128	744	1392	1512
18/05/15 12:10	1176	600	1296	1392
18/05/15 12:15	1188	804	1320	1584
18/05/15 12:20	1308	696	1416	1536
18/05/15 12:25	1428	564	1308	1692
18/05/15 12:30	1104	564	1488	1392
18/05/15 12:35	1152	768	1224	1440
18/05/15 12:40	1320	732	1008	1512
18/05/15 12:45	1416	828	1356	1800
18/05/15 12:50	1524	696	1296	1752
18/05/15 12:55	1476	624	1236	1740
18/05/15 13:00	1308	840	1080	1704
18/05/15 13:05	1188	900	1044	1788
18/05/15 13:10	1368	576	1176	1524
18/05/15 13:15	1404	660	1140	1920
18/05/15 13:20	1728	672	1152	1980
18/05/15 13:25	1632	756	1152	2220
18/05/15 13:30	1068	624	1056	1416
18/05/15 13:35	1044	1008	1080	1776
18/05/15 13:40	1344	720	876	1704
18/05/15 13:45	1512	756	1032	2172
18/05/15 13:50	1308	672	1236	1716
18/05/15 13:55	1452	840	1044	1896
18/05/15 14:00	1680	576	1344	1932
18/05/15 14:05	1284	780	948	1896
18/05/15 14:10	960	792	1164	1476
18/05/15 14:15	1188	588	1164	1596
18/05/15 14:20	1440	648	996	1740
18/05/15 14:25	1188	720	1356	1620
18/05/15 14:30	1284	816	948	1836
18/05/15 14:35	1176	936	1116	1788
18/05/15 14:40	1440	756	1368	1788
18/05/15 14:45	1512	684	1068	1884
18/05/15 14:50	1536	648	1176	1908
18/05/15 14:55	1140	804	1452	1728
18/05/15 15:00	1164	732	912	1416
18/05/15 15:05	1164	636	1680	1728
18/05/15 15:10	1236	756	1236	1644
18/05/15 15:15	1032	792	1236	1644
18/05/15 15:20	1284	744	1404	1620
18/05/15 15:25	1152	672	984	1620
18/05/15 15:30	1056	552	1224	1452
18/05/15 15:35	1248	672	1200	1464
18/05/15 15:40	1200	684	1260	1608
18/05/15 15:45	1152	840	1332	1416
18/05/15 15:50	1116	624	1164	1680
18/05/15 15:55	1188	612	1272	1524
18/05/15 16:00	1116	660	1212	1464
18/05/15 16:05	1032	588	1104	1260
18/05/15 16:10	1032	576	1188	1260
18/05/15 16:15	960	816	1284	1416
18/05/15 16:20	1236	660	1176	1584
18/05/15 16:25	996	528	1476	1248
18/05/15 16:30	1068	516	1740	1260

Sistema de Controle de Tráfego Urbano OPTIMUS

5 MINUTOS	INTENSIDADE			
	PM 0401003	PM 0401004	PM 0401006	PM 0402001
18/05/15 16:35	1248	708	1272	1524
18/05/15 16:40	1164	804	1752	1572
18/05/15 16:45	1080	528	1668	1344
18/05/15 16:50	1092	852	1608	1404
18/05/15 16:55	1272	756	1452	1608
18/05/15 17:00	1296	672	1944	1440
18/05/15 17:05	996	672	1872	1380
18/05/15 17:10	504	648	1812	564
18/05/15 17:15	924	360	1440	1020
18/05/15 17:20	1524	600	1596	1776
18/05/15 17:25	1596	552	1848	1776
18/05/15 17:30	1188	636	1872	1200
18/05/15 17:35	1272	468	1308	1620
18/05/15 17:40	1044	684	1260	1212
18/05/15 17:45	1356	372	1668	1428
18/05/15 17:50	1188	504	1788	924
18/05/15 17:55	1308	204	1812	1116
18/05/15 18:00	1032	456	1632	1068
18/05/15 18:05	984	372	1548	924
18/05/15 18:10	960	384	1488	1092
18/05/15 18:15	1668	408	1800	1740
18/05/15 18:20	1272	672	1344	1584
18/05/15 18:25	972	396	1812	1356
18/05/15 18:30	804	840	1812	948
18/05/15 18:35	936	420	1788	1128
18/05/15 18:40	1104	768	1524	1524
18/05/15 18:45	1116	660	1560	1560
18/05/15 18:50	1404	600	1728	1620
18/05/15 18:55	1284	660	2040	1584
18/05/15 19:00	1188	624	1800	1452
18/05/15 19:05	1224	552	1836	1452
18/05/15 19:10	1140	600	1608	1200
18/05/15 19:15	984	588	1644	1320
18/05/15 19:20	1200	696	1608	1440
18/05/15 19:25	1248	564	1632	1548
18/05/15 19:30	1032	528	1632	1332
18/05/15 19:35	816	576	1104	1068
18/05/15 19:40	900	576	972	1116
18/05/15 19:45	972	528	1080	1176
18/05/15 19:50	1056	564	1188	1236
18/05/15 19:55	756	480	1176	1140
18/05/15 20:00	936	396	1224	1020
18/05/15 20:05	600	396	876	780
18/05/15 20:10	828	432	780	852
18/05/15 20:15	732	432	1056	1272
18/05/15 20:20	768	468	852	948
18/05/15 20:25	588	492	900	864
18/05/15 20:30	828	528	864	1068
18/05/15 20:35	672	420	768	876
18/05/15 20:40	744	444	924	960
18/05/15 20:45	600	420	816	768
18/05/15 20:50	756	408	864	948
18/05/15 20:55	636	360	828	876
18/05/15 21:00	672	384	744	768
18/05/15 21:05	672	384	972	768
18/05/15 21:10	492	348	912	648
18/05/15 21:15	516	396	864	660
18/05/15 21:20	756	396	984	912

Sistema de Controle de Tráfego Urbano OPTIMUS

5 MINUTOS	INTENSIDADE			
	PM 0401003	PM 0401004	PM 0401006	PM 0402001
18/05/15 21:25	492	324	780	720
18/05/15 21:30	420	288	756	564
18/05/15 21:35	600	492	744	720
18/05/15 21:40	480	360	684	648
18/05/15 21:45	468	276	756	612
18/05/15 21:50	540	396	816	696
18/05/15 21:55	528	312	732	672
18/05/15 22:00	552	252	636	672
18/05/15 22:05	564	456	720	756
18/05/15 22:10	600	312	864	816
18/05/15 22:15	444	432	876	792
18/05/15 22:20	708	312	708	840
18/05/15 22:25	600	384	816	804
18/05/15 22:30	780	276	876	900
18/05/15 22:35	324	120	648	384
18/05/15 22:40	480	360	1104	720
18/05/15 22:45	480	336	864	720
18/05/15 22:50	480	240	732	720
18/05/15 22:55	480	240	816	720
18/05/15 23:00	456	240	420	672
18/05/15 23:05	360	240	384	480
18/05/15 23:10	360	240	408	480
18/05/15 23:15	336	216	444	456
18/05/15 23:20	240	120	300	360
18/05/15 23:25	240	120	192	360
18/05/15 23:30	216	120	324	336
18/05/15 23:35	120	120	264	240
18/05/15 23:40	120	120	252	240
18/05/15 23:45	120	96	264	240
18/05/15 23:50	120	0	132	240
18/05/15 23:55	120	0	132	240
19/05/15 00:00	120	0	192	216
19/05/15 00:05	120	0	132	120
19/05/15 00:10	120	0	120	120
19/05/15 00:15	96	0	180	120
19/05/15 00:20	0	0	204	120
19/05/15 00:25	0	0	120	120
19/05/15 00:30	0	0	84	120
19/05/15 00:35	0	0	156	120
19/05/15 00:40	0	0	168	120
19/05/15 00:45	0	0	120	120
19/05/15 00:50	0	0	120	120
19/05/15 00:55	0	0	108	120
19/05/15 01:00	0	0	72	96
19/05/15 01:05	0	0	84	0
19/05/15 01:10	0	0	60	0
19/05/15 01:15	0	0	84	0
19/05/15 01:20	0	0	72	0
19/05/15 01:25	0	0	24	0
19/05/15 01:30	0	0	96	0
19/05/15 01:35	0	0	96	0
19/05/15 01:40	0	0	72	0
19/05/15 01:45	0	0	24	0
19/05/15 01:50	0	0	36	0
19/05/15 01:55	0	0	96	0
19/05/15 02:00	0	0	108	0
19/05/15 02:05	0	0	60	0
19/05/15 02:10	0	0	48	0

Sistema de Controle de Tráfego Urbano OPTIMUS

5 MINUTOS	INTENSIDADE			
	PM 0401003	PM 0401004	PM 0401006	PM 0402001
19/05/15 02:15	0	0	48	0
19/05/15 02:20	0	0	48	0
19/05/15 02:25	0	0	72	0
19/05/15 02:30	0	0	0	0
19/05/15 02:35	0	0	36	0
19/05/15 02:40	0	0	48	0
19/05/15 02:45	0	0	36	0
19/05/15 02:50	0	0	12	0
19/05/15 02:55	0	0	0	0
19/05/15 03:00	0	0	36	0
19/05/15 03:05	0	0	36	0
19/05/15 03:10	0	0	60	0
19/05/15 03:15	0	0	48	0
19/05/15 03:20	0	0	24	0
19/05/15 03:25	0	0	12	0
19/05/15 03:30	0	0	48	0
19/05/15 03:35	0	0	0	0
19/05/15 03:40	0	0	12	0
19/05/15 03:45	0	0	24	0
19/05/15 03:50	0	0	12	0
19/05/15 03:55	0	0	36	0
19/05/15 04:00	0	0	0	0
19/05/15 04:05	0	0	24	0
19/05/15 04:10	0	0	48	0
19/05/15 04:15	0	0	12	0
19/05/15 04:20	0	0	12	0
19/05/15 04:25	0	0	0	0
19/05/15 04:30	0	0	24	0
19/05/15 04:35	0	0	12	0
19/05/15 04:40	0	0	36	0
19/05/15 04:45	0	0	60	0
19/05/15 04:50	0	0	24	0
19/05/15 04:55	0	0	36	0
19/05/15 05:00	0	0	96	0
19/05/15 05:05	0	0	72	0
19/05/15 05:10	0	0	60	0
19/05/15 05:15	0	0	36	0
19/05/15 05:20	0	0	36	0
19/05/15 05:25	0	0	72	0
19/05/15 05:30	0	24	36	24
19/05/15 05:35	0	120	72	120
19/05/15 05:40	0	120	96	120
19/05/15 05:45	24	120	192	144
19/05/15 05:50	120	120	120	240
19/05/15 05:55	120	120	72	240
19/05/15 06:00	144	144	204	288
19/05/15 06:05	240	240	96	480
19/05/15 06:10	240	240	228	480
19/05/15 06:15	288	264	276	504
19/05/15 06:20	480	360	192	600
19/05/15 06:25	480	360	348	600
19/05/15 06:30	528	384	312	696
19/05/15 06:35	720	480	336	1080
19/05/15 06:40	720	480	156	1080
19/05/15 06:45	840	504	540	1200
19/05/15 06:50	1320	600	840	1680
19/05/15 06:55	1320	600	984	1680
19/05/15 07:00	1368	624	972	1752

Sistema de Controle de Tráfego Urbano OPTIMUS

5 MINUTOS	INTENSIDADE			
	PM 0401003	PM 0401004	PM 0401006	PM 0402001
19/05/15 07:05	1560	720	888	2040
19/05/15 07:10	1560	720	1116	2040
19/05/15 07:15	1584	696	852	2064
19/05/15 07:20	1680	600	744	2160
19/05/15 07:25	1680	600	912	2160
19/05/15 07:30	1632	600	828	2088
19/05/15 07:35	1440	600	912	1800
19/05/15 07:40	1440	600	756	1800
19/05/15 07:45	1344	576	864	1704
19/05/15 07:50	960	480	744	1320
19/05/15 07:55	960	480	900	1320
19/05/15 08:00	1032	480	816	1368
19/05/15 08:05	1320	480	912	1560
19/05/15 08:10	1320	480	1200	1560
19/05/15 08:15	1320	504	768	1584
19/05/15 08:20	1320	600	804	1680
19/05/15 08:25	1320	600	1080	1680
19/05/15 08:30	1320	600	744	1704
19/05/15 08:35	1320	600	996	1800
19/05/15 08:40	1320	600	1020	1800
19/05/15 08:45	1320	600	1020	1776
19/05/15 08:50	1320	600	816	1680
19/05/15 08:55	1320	600	912	1680
19/05/15 09:00	1296	600	696	1680
19/05/15 09:05	1200	600	1068	1680
19/05/15 09:10	1200	600	1032	1680
19/05/15 09:15	1224	600	840	1680
19/05/15 09:20	1320	600	636	1680
19/05/15 09:25	1320	600	1140	1680
19/05/15 09:30	1296	600	1008	1680
19/05/15 09:35	1200	600	960	1680
19/05/15 09:40	1200	600	888	1680
19/05/15 09:45	1200	600	1044	1656
19/05/15 09:50	1200	600	696	1560
19/05/15 09:55	1200	600	1116	1560
19/05/15 10:00	1200	600	876	1584
19/05/15 10:05	1200	600	996	1680
19/05/15 10:10	1200	600	852	1680
19/05/15 10:15	1176	624	1092	1656
19/05/15 10:20	1080	720	816	1560
19/05/15 10:25	1080	720	1080	1560
19/05/15 10:30	1080	720	960	1560
19/05/15 10:35	1080	720	1236	1560
19/05/15 10:40	1080	720	804	1560
19/05/15 10:45	1056	696	1092	1512
19/05/15 10:50	960	600	1200	1320
19/05/15 10:55	960	600	1128	1320
19/05/15 11:00	960	600	1200	1344
19/05/15 11:05	960	600	1464	1440
19/05/15 11:10	960	600	1356	1440
19/05/15 11:15	960	600	1560	1416
19/05/15 11:20	960	600	1332	1320
19/05/15 11:25	960	600	1416	1320
19/05/15 11:30	984	600	1500	1320
19/05/15 11:35	1080	600	1560	1320
19/05/15 11:40	1080	600	1392	1320
19/05/15 11:45	1056	600	1584	1320
19/05/15 11:50	960	600	1692	1320

Sistema de Controle de Tráfego Urbano OPTIMUS

5 MINUTOS	INTENSIDADE			
	PM 0401003	PM 0401004	PM 0401006	PM 0402001
19/05/15 11:55	960	600	1248	1320
19/05/15 12:00	1008	624	1236	1344
19/05/15 12:05	1200	720	1584	1440
19/05/15 12:10	1200	720	1404	1440
19/05/15 12:15	1200	696	1176	1440
19/05/15 12:20	1200	600	1404	1440
19/05/15 12:25	1200	600	1512	1440
19/05/15 12:30	1176	600	1260	1440
19/05/15 12:35	1080	600	1296	1440
19/05/15 12:40	1080	600	1212	1440
19/05/15 12:45	1080	624	1080	1440
19/05/15 12:50	1080	720	984	1440
19/05/15 12:55	1080	720	1260	1440
19/05/15 13:00	1128	696	948	1464
19/05/15 13:05	1320	600	1260	1560
19/05/15 13:10	1320	600	1440	1560
19/05/15 13:15	1296	624	1152	1560
19/05/15 13:20	1200	720	1332	1560
19/05/15 13:25	1200	720	1440	1560
19/05/15 13:30	1200	696	984	1560
19/05/15 13:35	1200	600	1080	1560
19/05/15 13:40	1200	600	792	1560
19/05/15 13:45	1200	600	1176	1560
19/05/15 13:50	1200	600	996	1560
19/05/15 13:55	1200	600	1308	1560
19/05/15 14:00	1200	624	1344	1584
19/05/15 14:05	1200	720	1104	1680
19/05/15 14:10	1200	720	1344	1680
19/05/15 14:15	1176	720	960	1656
19/05/15 14:20	1080	720	840	1560
19/05/15 14:25	1080	720	948	1560
19/05/15 14:30	1080	696	1080	1560
19/05/15 14:35	1080	600	1080	1560
19/05/15 14:40	1080	600	1080	1560
19/05/15 14:45	1080	600	1080	1536
19/05/15 14:50	1080	600	1080	1440
19/05/15 14:55	1080	600	1080	1440
19/05/15 15:00	1080	600	1080	1464
19/05/15 15:05	1080	600	1080	1560
19/05/15 15:10	1080	600	1080	1560
19/05/15 15:15	1080	600	1080	1536
19/05/15 15:20	1080	600	1080	1440
19/05/15 15:25	1080	600	1188	1440
19/05/15 15:30	1080	600	1320	1440
19/05/15 15:35	1080	600	948	1440
19/05/15 15:40	1080	600	1080	1440
19/05/15 15:45	1080	600	1104	1416
19/05/15 15:50	1080	600	1200	1320
19/05/15 15:55	1080	600	1200	1320
19/05/15 16:00	1104	600	1224	1344
19/05/15 16:05	1200	600	1320	1440
19/05/15 16:10	1200	600	1320	1440
19/05/15 16:15	1176	600	1320	1440
19/05/15 16:20	1080	600	1320	1440
19/05/15 16:25	1080	600	1164	1440
19/05/15 16:30	1056	624	1320	1416
19/05/15 16:35	960	720	1380	1320
19/05/15 16:40	960	720	1572	1320

Sistema de Controle de Tráfego Urbano OPTIMUS

5 MINUTOS	INTENSIDADE			
	PM 0401003	PM 0401004	PM 0401006	PM 0402001
19/05/15 16:45	984	696	1296	1344
19/05/15 16:50	1080	600	1440	1440
19/05/15 16:55	1080	600	1440	1440
19/05/15 17:00	1080	600	1440	1440
19/05/15 17:05	1080	600	1440	1440
19/05/15 17:10	1080	600	1440	1440
19/05/15 17:15	1080	600	1440	1440
19/05/15 17:20	1080	600	1440	1440
19/05/15 17:25	1080	600	1440	1440
19/05/15 17:30	1080	600	1464	1416
19/05/15 17:35	1080	600	1560	1320
19/05/15 17:40	1080	600	1560	1320
19/05/15 17:45	1080	600	1584	1296
19/05/15 17:50	1080	600	1680	1200
19/05/15 17:55	1080	600	1680	1200
19/05/15 18:00	1104	600	1632	1248
19/05/15 18:05	1200	600	1248	1440
19/05/15 18:10	1200	600	1440	1440
19/05/15 18:15	1200	576	1440	1440
19/05/15 18:20	1200	480	1596	1440
19/05/15 18:25	1200	480	1812	1440
19/05/15 18:30	1176	528	1584	1440
19/05/15 18:35	1080	720	1440	1440
19/05/15 18:40	1080	720	1464	1440
19/05/15 18:45	1056	696	1524	1392
19/05/15 18:50	960	600	1332	1200
19/05/15 18:55	960	600	1236	1200
19/05/15 19:00	960	600	1320	1224
19/05/15 19:05	960	600	1704	1320
19/05/15 19:10	960	600	1812	1320
19/05/15 19:15	984	600	1320	1320
19/05/15 19:20	1080	600	1716	1320
19/05/15 19:25	1080	600	1524	1320
19/05/15 19:30	1080	576	1488	1320
19/05/15 19:35	1080	480	996	1320
19/05/15 19:40	1080	480	852	1320
19/05/15 19:45	1056	480	1296	1296
19/05/15 19:50	960	480	972	1200
19/05/15 19:55	960	480	1344	1200
19/05/15 20:00	936	480	960	1176
19/05/15 20:05	840	480	1212	1080
19/05/15 20:10	840	480	924	1080
19/05/15 20:15	816	480	1068	1080
19/05/15 20:20	720	480	936	1080
19/05/15 20:25	720	480	948	1080
19/05/15 20:30	720	456	780	1032
19/05/15 20:35	720	360	780	840
19/05/15 20:40	720	360	1056	840
19/05/15 20:45	744	360	1008	864
19/05/15 20:50	840	360	828	960
19/05/15 20:55	840	360	756	960
19/05/15 21:00	816	360	996	936
19/05/15 21:05	720	360	984	840
19/05/15 21:10	720	360	1032	840
19/05/15 21:15	696	360	1092	840
19/05/15 21:20	600	360	912	840
19/05/15 21:25	600	360	1020	840
19/05/15 21:30	600	336	900	816

Sistema de Controle de Tráfego Urbano OPTIMUS

5 MINUTOS	INTENSIDADE			
	PM 0401003	PM 0401004	PM 0401006	PM 0402001
19/05/15 21:35	600	240	684	720
19/05/15 21:40	600	240	780	720
19/05/15 21:45	600	264	876	720
19/05/15 21:50	600	360	900	720
19/05/15 21:55	600	360	768	720
19/05/15 22:00	600	360	684	744
19/05/15 22:05	600	360	816	840
19/05/15 22:10	600	360	1032	840
19/05/15 22:15	600	360	888	840
19/05/15 22:20	600	360	936	840
19/05/15 22:25	600	360	816	840
19/05/15 22:30	600	336	456	816
19/05/15 22:35	600	240	720	720
19/05/15 22:40	600	240	720	720
19/05/15 22:45	576	240	720	720
19/05/15 22:50	480	240	720	720
19/05/15 22:55	480	240	720	720
19/05/15 23:00	480	216	720	696
19/05/15 23:05	480	120	720	600
19/05/15 23:10	480	120	720	600
19/05/15 23:15	432	120	672	552
19/05/15 23:20	240	120	480	360
19/05/15 23:25	240	120	480	360
19/05/15 23:30	240	120	480	336
19/05/15 23:35	240	120	480	240
19/05/15 23:40	240	120	480	240
19/05/15 23:45	216	120	432	240
19/05/15 23:50	120	120	240	240
19/05/15 23:55	120	120	240	240
20/05/15 00:00	120	120	240	240
20/05/15 00:05	120	120	240	240
20/05/15 00:10	120	120	240	240
20/05/15 00:15	120	96	240	216
20/05/15 00:20	120	0	240	120
20/05/15 00:25	120	0	240	120
20/05/15 00:30	120	0	216	120
20/05/15 00:35	120	0	120	120
20/05/15 00:40	120	0	120	120
20/05/15 00:45	96	0	96	120
20/05/15 00:50	0	0	0	120
20/05/15 00:55	0	0	0	120
20/05/15 01:00	0	0	0	96
20/05/15 01:05	0	0	0	0
20/05/15 01:10	0	0	0	0
20/05/15 01:15	0	0	24	0
20/05/15 01:20	0	0	120	0
20/05/15 01:25	0	0	120	0
20/05/15 01:30	0	0	96	0
20/05/15 01:35	0	0	0	0
20/05/15 01:40	0	0	0	0
20/05/15 01:45	0	0	0	0
20/05/15 01:50	0	0	0	0
20/05/15 01:55	0	0	0	0
20/05/15 02:00	0	0	0	0
20/05/15 02:05	0	0	0	0
20/05/15 02:10	0	0	0	0
20/05/15 02:15	0	0	0	0
20/05/15 02:20	0	0	0	0

Sistema de Controle de Tráfego Urbano OPTIMUS

5 MINUTOS	INTENSIDADE			
	PM 0401003	PM 0401004	PM 0401006	PM 0402001
20/05/15 02:25	0	0	0	0
20/05/15 02:30	0	0	0	0
20/05/15 02:35	0	0	0	0
20/05/15 02:40	0	0	0	0
20/05/15 02:45	0	0	0	0
20/05/15 02:50	0	0	0	0
20/05/15 02:55	0	0	0	0
20/05/15 03:00	0	0	0	0
20/05/15 03:05	0	0	0	0
20/05/15 03:10	0	0	0	0
20/05/15 03:15	0	0	0	0
20/05/15 03:20	0	0	0	0
20/05/15 03:25	0	0	0	0
20/05/15 03:30	0	0	0	0
20/05/15 03:35	0	0	0	0
20/05/15 03:40	0	0	0	0
20/05/15 03:45	0	0	0	0
20/05/15 03:50	0	0	0	0
20/05/15 03:55	0	0	0	0
20/05/15 04:00	0	0	0	0
20/05/15 04:05	0	0	0	0
20/05/15 04:10	0	0	0	0
20/05/15 04:15	0	0	0	0
20/05/15 04:20	0	0	0	0
20/05/15 04:25	0	0	0	0
20/05/15 04:30	0	0	0	0
20/05/15 04:35	0	0	0	0
20/05/15 04:40	0	0	0	0
20/05/15 04:45	0	0	0	0
20/05/15 04:50	0	0	0	0
20/05/15 04:55	0	0	0	0
20/05/15 05:00	0	0	0	0
20/05/15 05:05	0	0	0	0
20/05/15 05:10	0	0	0	0
20/05/15 05:15	0	0	0	0
20/05/15 05:20	0	0	0	0
20/05/15 05:25	0	0	0	0
20/05/15 05:30	0	24	0	24
20/05/15 05:35	0	120	0	120
20/05/15 05:40	0	120	0	120
20/05/15 05:45	24	120	0	144
20/05/15 05:50	120	120	0	240
20/05/15 05:55	120	120	0	240
20/05/15 06:00	168	144	0	288
20/05/15 06:05	360	240	0	480
20/05/15 06:10	360	240	0	480
20/05/15 06:15	360	264	0	504
20/05/15 06:20	360	360	0	600
20/05/15 06:25	360	360	0	600
20/05/15 06:30	456	384	24	720
20/05/15 06:35	840	480	120	1200
20/05/15 06:40	840	480	120	1200
20/05/15 06:45	936	528	144	1320
20/05/15 06:50	1320	720	240	1800
20/05/15 06:55	1320	720	240	1800
20/05/15 07:00	1368	720	312	1872
20/05/15 07:05	1560	720	600	2160
20/05/15 07:10	1560	720	600	2160

Sistema de Controle de Tráfego Urbano OPTIMUS

5 MINUTOS	INTENSIDADE			
	PM 040100 3	PM 040100 4	PM 040100 6	PM 040200 1
20/05/15 07:15	1560	720	648	2160
20/05/15 07:20	1560	720	840	2160
20/05/15 07:25	1560	720	840	2160
20/05/15 07:30	1560	696	840	2136
20/05/15 07:35	1560	600	840	2040
20/05/15 07:40	1560	600	840	2040
20/05/15 07:45	1536	600	816	1992
20/05/15 07:50	1440	600	684	1800
20/05/15 07:55	1440	600	744	1800
20/05/15 08:00	1440	600	720	1824
20/05/15 08:05	1440	600	720	1920
20/05/15 08:10	1440	600	720	1920
20/05/15 08:15	1416	600	720	1920
20/05/15 08:20	1320	600	720	1920
20/05/15 08:25	1320	600	720	1920
20/05/15 08:30	1320	600	720	1896
20/05/15 08:35	1320	600	720	1800
20/05/15 08:40	1320	600	720	1800
20/05/15 08:45	1344	600	720	1824
20/05/15 08:50	1440	600	720	1920
20/05/15 08:55	1440	600	720	1920
20/05/15 09:00	1416	600	744	1896
20/05/15 09:05	1320	600	840	1800
20/05/15 09:10	1320	600	840	1800
20/05/15 09:15	1344	600	840	1800
20/05/15 09:20	1440	600	840	1800
20/05/15 09:25	1440	600	840	1800
20/05/15 09:30	1392	624	816	1776
20/05/15 09:35	1200	720	720	1680
20/05/15 09:40	1200	720	720	1680
20/05/15 09:45	1200	720	720	1680
20/05/15 09:50	1200	720	720	1680
20/05/15 09:55	1200	720	720	1680
20/05/15 10:00	1200	720	744	1680
20/05/15 10:05	1200	720	840	1680
20/05/15 10:10	1200	720	840	1680
20/05/15 10:15	1200	720	840	1656
20/05/15 10:20	1200	720	840	1560
20/05/15 10:25	1200	720	840	1560
20/05/15 10:30	1176	720	912	1560
20/05/15 10:35	1080	720	1272	1560
20/05/15 10:40	1080	720	1008	1560
20/05/15 10:45	1080	696	960	1560
20/05/15 10:50	1080	600	780	1560
20/05/15 10:55	1080	600	960	1560
20/05/15 11:00	1080	600	960	1536
20/05/15 11:05	1080	600	960	1440
20/05/15 11:10	1080	600	960	1440
20/05/15 11:15	1080	600	960	1440
20/05/15 11:20	1080	600	960	1440
20/05/15 11:25	1080	600	960	1440
20/05/15 11:30	1080	600	984	1440
20/05/15 11:35	1080	600	1080	1440
20/05/15 11:40	1080	600	1080	1440
20/05/15 11:45	1080	600	1128	1440
20/05/15 11:50	1080	600	1320	1440
20/05/15 11:55	1080	600	1320	1440
20/05/15 12:00	1128	600	1284	1464

Sistema de Controle de Tráfego Urbano OPTIMUS

5 MINUTOS	INTENSIDADE			
	PM 040100 3	PM 040100 4	PM 040100 6	PM 040200 1
20/05/15 12:05	1320	600	1416	1560
20/05/15 12:10	1320	600	1272	1560
20/05/15 12:15	1296	600	1044	1560
20/05/15 12:20	1200	600	1440	1560
20/05/15 12:25	1200	600	1512	1560
20/05/15 12:30	1200	624	1476	1560
20/05/15 12:35	1200	720	1776	1560
20/05/15 12:40	1200	720	1116	1560
20/05/15 12:45	1224	720	1500	1584
20/05/15 12:50	1320	720	1248	1680
20/05/15 12:55	1320	720	1248	1680
20/05/15 13:00	1320	720	924	1680
20/05/15 13:05	1320	720	1032	1680
20/05/15 13:10	1320	720	1200	1680
20/05/15 13:15	1344	720	780	1704
20/05/15 13:20	1440	720	1200	1800
20/05/15 13:25	1440	720	1404	1800
20/05/15 13:30	1416	720	984	1800
20/05/15 13:35	1320	720	1080	1800
20/05/15 13:40	1320	720	780	1800
20/05/15 13:45	1296	720	912	1776
20/05/15 13:50	1200	720	840	1680
20/05/15 13:55	1200	720	840	1680
20/05/15 14:00	1200	720	1152	1680
20/05/15 14:05	1200	720	840	1680
20/05/15 14:10	1200	720	816	1680
20/05/15 14:15	1200	720	732	1656
20/05/15 14:20	1200	720	744	1560
20/05/15 14:25	1200	720	852	1560
20/05/15 14:30	1224	720	948	1584
20/05/15 14:35	1320	720	804	1680
20/05/15 14:40	1320	720	1080	1680
20/05/15 14:45	1296	696	936	1656
20/05/15 14:50	1200	600	780	1560
20/05/15 14:55	1200	600	1080	1560
20/05/15 15:00	1200	600	1104	1560
20/05/15 15:05	1200	600	1200	1560
20/05/15 15:10	1200	600	1200	1560
20/05/15 15:15	1176	624	1200	1560
20/05/15 15:20	1080	720	1200	1560
20/05/15 15:25	1080	720	1200	1560
20/05/15 15:30	1080	696	1200	1560
20/05/15 15:35	1080	600	1200	1560
20/05/15 15:40	1080	600	1200	1560
20/05/15 15:45	1080	600	1200	1536
20/05/15 15:50	1080	600	1200	1440
20/05/15 15:55	1080	600	1200	1440
20/05/15 16:00	1080	600	1200	1440
20/05/15 16:05	1080	600	1200	1440
20/05/15 16:10	1080	600	1200	1440
20/05/15 16:15	1080	600	1200	1440
20/05/15 16:20	1080	600	936	1440
20/05/15 16:25	1080	600	1296	1440
20/05/15 16:30	1056	600	1656	1440
20/05/15 16:35	960	600	1440	1440
20/05/15 16:40	960	600	1308	1440
20/05/15 16:45	960	600	1464	1416
20/05/15 16:50	960	600	1500	1320

Sistema de Controle de Tráfego Urbano OPTIMUS

5 MINUTOS	INTENSIDADE			
	PM 0401003	PM 0401004	PM 0401006	PM 0402001
20/05/15 16:55	960	600	1584	1320
20/05/15 17:00	984	600	1356	1344
20/05/15 17:05	1080	600	1560	1440
20/05/15 17:10	1080	600	1440	1440
20/05/15 17:15	1104	600	1788	1464
20/05/15 17:20	1200	600	1728	1560
20/05/15 17:25	1200	600	1800	1560
20/05/15 17:30	1152	600	1992	1488
20/05/15 17:35	960	600	1968	1200
20/05/15 17:40	960	600	1896	1200
20/05/15 17:45	960	576	1704	1176
20/05/15 17:50	960	480	1752	1080
20/05/15 17:55	960	480	1488	1080
20/05/15 18:00	960	480	1428	1080
20/05/15 18:05	960	480	1620	1080
20/05/15 18:10	960	480	1860	1080
20/05/15 18:15	984	480	1692	1104
20/05/15 18:20	1080	480	1464	1200
20/05/15 18:25	1080	480	1884	1200
20/05/15 18:30	1032	480	1488	1176
20/05/15 18:35	840	480	1668	1080
20/05/15 18:40	840	480	1824	1080
20/05/15 18:45	792	504	1764	1056
20/05/15 18:50	600	600	1716	960
20/05/15 18:55	600	600	1308	960
20/05/15 19:00	744	576	1524	1056
20/05/15 19:05	1320	480	1476	1440
20/05/15 19:10	1320	480	1740	1440
20/05/15 19:15	1296	480	1632	1440
20/05/15 19:20	1200	480	1140	1440
20/05/15 19:25	1200	480	1536	1440
20/05/15 19:30	1128	480	1548	1368
20/05/15 19:35	840	480	1104	1080
20/05/15 19:40	840	480	1140	1080
20/05/15 19:45	888	480	1128	1128
20/05/15 19:50	1080	480	816	1320
20/05/15 19:55	1080	480	1452	1320
20/05/15 20:00	1056	480	972	1320
20/05/15 20:05	960	480	1056	1320
20/05/15 20:10	960	480	1068	1320
20/05/15 20:15	936	480	1008	1272
20/05/15 20:20	840	480	852	1080
20/05/15 20:25	840	480	984	1080
20/05/15 20:30	840	480	1128	1080
20/05/15 20:35	840	480	816	1080
20/05/15 20:40	840	480	816	1080
20/05/15 20:45	840	480	888	1080
20/05/15 20:50	840	480	696	1080
20/05/15 20:55	840	480	972	1080
20/05/15 21:00	816	456	804	1056
20/05/15 21:05	720	360	852	960
20/05/15 21:10	720	360	1104	960
20/05/15 21:15	696	360	912	936
20/05/15 21:20	600	360	1116	840
20/05/15 21:25	600	360	756	840
20/05/15 21:30	600	384	792	840
20/05/15 21:35	600	480	816	840
20/05/15 21:40	600	480	588	840

Sistema de Controle de Tráfego Urbano OPTIMUS

5 MINUTOS	INTENSIDADE			
	PM 0401003	PM 0401004	PM 0401006	PM 0402001
20/05/15 21:45	576	456	684	816
20/05/15 21:50	480	360	516	720
20/05/15 21:55	480	360	492	720
20/05/15 22:00	504	360	576	744
20/05/15 22:05	600	360	720	840
20/05/15 22:10	600	360	552	840
20/05/15 22:15	600	360	720	816
20/05/15 22:20	600	360	720	720
20/05/15 22:25	600	360	780	720
20/05/15 22:30	576	360	564	720
20/05/15 22:35	480	360	972	720
20/05/15 22:40	480	360	876	720
20/05/15 22:45	480	336	864	696
20/05/15 22:50	480	240	588	600
20/05/15 22:55	480	240	636	600
20/05/15 23:00	480	240	420	600
20/05/15 23:05	480	240	492	600
20/05/15 23:10	480	240	456	600
20/05/15 23:15	432	216	576	552
20/05/15 23:20	240	120	480	360
20/05/15 23:25	240	120	480	360
20/05/15 23:30	240	120	384	336
20/05/15 23:35	240	120	360	240
20/05/15 23:40	240	120	360	240
20/05/15 23:45	216	120	228	240
20/05/15 23:50	120	120	276	240
20/05/15 23:55	120	120	192	240
21/05/15 00:00	120	120	216	240
21/05/15 00:05	120	120	240	240
21/05/15 00:10	120	120	132	240
21/05/15 00:15	144	168	132	288
21/05/15 00:20	240	360	168	480
21/05/15 00:25	240	360	144	480
21/05/15 00:30	240	360	132	456
21/05/15 00:35	240	360	132	360
21/05/15 00:40	240	360	132	360
21/05/15 00:45	216	360	204	360
21/05/15 00:50	120	360	60	360
21/05/15 00:55	120	360	72	360
21/05/15 01:00	120	312	156	336
21/05/15 01:05	120	120	96	240
21/05/15 01:10	120	120	108	240
21/05/15 01:15	96	120	120	216
21/05/15 01:20	0	120	72	120
21/05/15 01:25	0	120	48	120
21/05/15 01:30	0	96	120	120
21/05/15 01:35	0	0	96	120
21/05/15 01:40	0	0	48	120
21/05/15 01:45	0	0	60	96
21/05/15 01:50	0	0	60	0
21/05/15 01:55	0	0	84	0
21/05/15 02:00	0	0	24	0
21/05/15 02:05	0	0	48	0
21/05/15 02:10	0	0	36	0
21/05/15 02:15	0	0	48	0
21/05/15 02:20	0	0	48	0
21/05/15 02:25	0	0	48	0
21/05/15 02:30	0	0	60	0

Sistema de Controle de Tráfego Urbano OPTIMUS

5 MINUTOS	INTENSIDADE			
	PM 0401003	PM 0401004	PM 0401006	PM 0402001
21/05/15 02:35	0	0	12	0
21/05/15 02:40	0	0	24	0
21/05/15 02:45	0	0	60	0
21/05/15 02:50	0	0	48	0
21/05/15 02:55	0	0	48	0
21/05/15 03:00	0	0	24	0
21/05/15 03:05	0	0	24	0
21/05/15 03:10	0	0	12	0
21/05/15 03:15	0	0	24	0
21/05/15 03:20	0	0	48	0
21/05/15 03:25	0	0	36	0
21/05/15 03:30	0	0	36	0
21/05/15 03:35	0	0	12	0
21/05/15 03:40	0	0	24	0
21/05/15 03:45	0	0	36	0
21/05/15 03:50	0	0	12	0
21/05/15 03:55	0	0	60	0
21/05/15 04:00	0	0	24	0
21/05/15 04:05	0	0	0	0
21/05/15 04:10	0	0	36	0
21/05/15 04:15	0	0	24	0
21/05/15 04:20	0	0	24	0
21/05/15 04:25	0	0	24	0
21/05/15 04:30	0	0	48	0
21/05/15 04:35	0	0	36	0
21/05/15 04:40	0	0	36	0
21/05/15 04:45	0	0	12	0
21/05/15 04:50	0	0	36	0
21/05/15 04:55	0	0	12	0
21/05/15 05:00	0	0	24	0
21/05/15 05:05	0	0	96	0
21/05/15 05:10	0	0	48	0
21/05/15 05:15	0	0	36	0
21/05/15 05:20	0	0	36	0
21/05/15 05:25	0	0	84	0
21/05/15 05:30	0	0	84	24
21/05/15 05:35	0	0	84	120
21/05/15 05:40	0	0	84	120
21/05/15 05:45	24	24	48	144
21/05/15 05:50	120	120	132	240
21/05/15 05:55	120	120	12	240
21/05/15 06:00	144	144	144	288
21/05/15 06:05	240	240	132	480
21/05/15 06:10	240	240	192	480
21/05/15 06:15	288	264	132	504
21/05/15 06:20	480	360	132	600
21/05/15 06:25	480	360	216	600
21/05/15 06:30	528	384	144	696
21/05/15 06:35	720	480	216	1080
21/05/15 06:40	720	480	408	1080
21/05/15 06:45	840	528	324	1224
21/05/15 06:50	1320	720	480	1800
21/05/15 06:55	1320	720	384	1800
21/05/15 07:00	1392	720	492	1872
21/05/15 07:05	1680	720	516	2160
21/05/15 07:10	1680	720	588	2160
21/05/15 07:15	1656	720	600	2160
21/05/15 07:20	1560	720	600	2160

Sistema de Controle de Tráfego Urbano OPTIMUS

5 MINUTOS	INTENSIDADE			
	PM 0401003	PM 0401004	PM 0401006	PM 0402001
21/05/15 07:25	1560	720	600	2160
21/05/15 07:30	1560	696	588	2160
21/05/15 07:35	1560	600	600	2160
21/05/15 07:40	1560	600	672	2160
21/05/15 07:45	1536	600	504	2112
21/05/15 07:50	1440	600	528	1920
21/05/15 07:55	1440	600	636	1920
21/05/15 08:00	1416	600	432	1896
21/05/15 08:05	1320	600	576	1800
21/05/15 08:10	1320	600	696	1800
21/05/15 08:15	1344	600	720	1800
21/05/15 08:20	1440	600	720	1800
21/05/15 08:25	1440	600	672	1800
21/05/15 08:30	1440	600	696	1824
21/05/15 08:35	1440	600	600	1920
21/05/15 08:40	1440	600	600	1920
21/05/15 08:45	1440	600	624	1896
21/05/15 08:50	1440	600	720	1800
21/05/15 08:55	1440	600	792	1800
21/05/15 09:00	1440	600	732	1824
21/05/15 09:05	1440	600	984	1920
21/05/15 09:10	1440	600	816	1920
21/05/15 09:15	1416	600	792	1872
21/05/15 09:20	1320	600	768	1680
21/05/15 09:25	1320	600	1152	1680
21/05/15 09:30	1320	600	840	1704
21/05/15 09:35	1320	600	960	1800
21/05/15 09:40	1320	600	840	1800
21/05/15 09:45	1296	624	840	1776
21/05/15 09:50	1200	720	840	1680
21/05/15 09:55	1200	720	840	1680
21/05/15 10:00	1200	720	804	1656
21/05/15 10:05	1200	720	1248	1560
21/05/15 10:10	1200	720	768	1560
21/05/15 10:15	1200	696	984	1560
21/05/15 10:20	1200	600	720	1560
21/05/15 10:25	1200	600	840	1560
21/05/15 10:30	1200	624	780	1560
21/05/15 10:35	1200	720	744	1560
21/05/15 10:40	1200	720	852	1560
21/05/15 10:45	1176	696	912	1536
21/05/15 10:50	1080	600	840	1440
21/05/15 10:55	1080	600	924	1440
21/05/15 11:00	1080	600	780	1440
21/05/15 11:05	1080	600	1032	1440
21/05/15 11:10	1080	600	696	1440
21/05/15 11:15	1080	624	1092	1440
21/05/15 11:20	1080	720	876	1440
21/05/15 11:25	1080	720	1080	1440
21/05/15 11:30	1104	696	780	1464
21/05/15 11:35	1200	600	936	1560
21/05/15 11:40	1200	600	1224	1560
21/05/15 11:45	1176	600	924	1536
21/05/15 11:50	1080	600	1080	1440
21/05/15 11:55	1080	600	1080	1440
21/05/15 12:00	1128	600	1104	1464
21/05/15 12:05	1320	600	1032	1560
21/05/15 12:10	1320	600	1200	1560

Sistema de Controle de Tráfego Urbano OPTIMUS

5 MINUTOS	INTENSIDADE			
	PM 040100 3	PM 040100 4	PM 040100 6	PM 040200 1
21/05/15 12:15	1296	600	1236	1536
21/05/15 12:20	1200	600	1032	1440
21/05/15 12:25	1200	600	1200	1440
21/05/15 12:30	1200	600	1200	1440
21/05/15 12:35	1200	600	1200	1440
21/05/15 12:40	1200	600	1200	1440
21/05/15 12:45	1200	624	972	1488
21/05/15 12:50	1200	720	852	1680
21/05/15 12:55	1200	720	960	1680
21/05/15 13:00	1224	696	984	1704
21/05/15 13:05	1320	600	1080	1800
21/05/15 13:10	1320	600	1080	1800
21/05/15 13:15	1344	600	1104	1800
21/05/15 13:20	1440	600	1200	1800
21/05/15 13:25	1440	600	1200	1800
21/05/15 13:30	1416	624	1200	1776
21/05/15 13:35	1320	720	1200	1680
21/05/15 13:40	1320	720	1200	1680
21/05/15 13:45	1296	696	1152	1656
21/05/15 13:50	1200	600	960	1560
21/05/15 13:55	1200	600	960	1560
21/05/15 14:00	1224	600	960	1584
21/05/15 14:05	1320	600	960	1680
21/05/15 14:10	1320	600	960	1680
21/05/15 14:15	1296	624	960	1656
21/05/15 14:20	1200	720	960	1560
21/05/15 14:25	1200	720	960	1560
21/05/15 14:30	1176	720	984	1584
21/05/15 14:35	1080	720	1080	1680
21/05/15 14:40	1080	720	1080	1680
21/05/15 14:45	1104	696	1056	1656
21/05/15 14:50	1200	600	960	1560
21/05/15 14:55	1200	600	960	1560
21/05/15 15:00	1200	624	984	1584
21/05/15 15:05	1200	720	1080	1680
21/05/15 15:10	1200	720	1080	1680
21/05/15 15:15	1200	720	1056	1656
21/05/15 15:20	1200	720	960	1560
21/05/15 15:25	1200	720	960	1560
21/05/15 15:30	1176	720	960	1560
21/05/15 15:35	1080	720	960	1560
21/05/15 15:40	1080	720	960	1560
21/05/15 15:45	1080	696	1008	1560
21/05/15 15:50	1080	600	1200	1560
21/05/15 15:55	1080	600	1200	1560
21/05/15 16:00	1080	600	1176	1536
21/05/15 16:05	1080	600	1080	1440
21/05/15 16:10	1080	600	1080	1440
21/05/15 16:15	1080	600	1104	1416
21/05/15 16:20	1080	600	1200	1320
21/05/15 16:25	1080	600	1200	1320
21/05/15 16:30	1080	600	1044	1344
21/05/15 16:35	1080	600	1320	1440
21/05/15 16:40	1080	600	1320	1440
21/05/15 16:45	1080	600	1344	1440
21/05/15 16:50	1080	600	1440	1440
21/05/15 16:55	1080	600	1440	1440
21/05/15 17:00	1080	600	1440	1440

Sistema de Controle de Tráfego Urbano OPTIMUS

5 MINUTOS	INTENSIDADE			
	PM 0401003	PM 0401004	PM 0401006	PM 0402001
21/05/15 17:05	1080	600	1440	1440
21/05/15 17:10	1080	600	1440	1440
21/05/15 17:15	1104	600	1356	1440
21/05/15 17:20	1200	600	1116	1440
21/05/15 17:25	1200	600	1308	1440
21/05/15 17:30	1176	600	1344	1440
21/05/15 17:35	1080	600	1440	1440
21/05/15 17:40	1080	600	948	1440
21/05/15 17:45	1080	576	1668	1416
21/05/15 17:50	1080	480	1368	1320
21/05/15 17:55	1080	480	1164	1320
21/05/15 18:00	1104	480	1440	1320
21/05/15 18:05	1200	480	936	1320
21/05/15 18:10	1200	480	828	1320
21/05/15 18:15	1176	480	1320	1296
21/05/15 18:20	1080	480	444	1200
21/05/15 18:25	1080	480	1464	1200
21/05/15 18:30	1080	480	888	1224
21/05/15 18:35	1080	480	1296	1320
21/05/15 18:40	1080	480	984	1320
21/05/15 18:45	1104	480	1380	1344
21/05/15 18:50	1200	480	1164	1440
21/05/15 18:55	1200	480	1764	1440
21/05/15 19:00	1176	504	1368	1464
21/05/15 19:05	1080	600	1692	1560
21/05/15 19:10	1080	600	1740	1560
21/05/15 19:15	1080	600	1668	1536
21/05/15 19:20	1080	600	1632	1440
21/05/15 19:25	1080	600	1740	1440
21/05/15 19:30	1080	600	1644	1440
21/05/15 19:35	1080	600	1740	1440
21/05/15 19:40	1080	600	1632	1440
21/05/15 19:45	1080	600	1404	1440
21/05/15 19:50	1080	600	1428	1440
21/05/15 19:55	1080	600	1092	1440
21/05/15 20:00	1056	600	1284	1392
21/05/15 20:05	960	600	1104	1200
21/05/15 20:10	960	600	1032	1200
21/05/15 20:15	960	576	1392	1200
21/05/15 20:20	960	480	1260	1200
21/05/15 20:25	960	480	996	1200
21/05/15 20:30	936	480	792	1176
21/05/15 20:35	840	480	792	1080
21/05/15 20:40	840	480	1092	1080
21/05/15 20:45	816	480	1008	1080
21/05/15 20:50	720	480	1080	1080
21/05/15 20:55	720	480	1020	1080
21/05/15 21:00	720	480	828	1056
21/05/15 21:05	720	480	840	960
21/05/15 21:10	720	480	1092	960
21/05/15 21:15	744	456	1116	960
21/05/15 21:20	840	360	1212	960
21/05/15 21:25	840	360	900	960
21/05/15 21:30	792	360	816	960
21/05/15 21:35	600	360	912	960
21/05/15 21:40	600	360	960	960
21/05/15 21:45	576	360	864	936
21/05/15 21:50	480	360	828	840

Sistema de Controle de Tráfego Urbano OPTIMUS

5 MINUTOS	INTENSIDADE			
	PM 0401003	PM 0401004	PM 0401006	PM 0402001
21/05/15 21:55	480	360	660	840
21/05/15 22:00	504	360	828	840
21/05/15 22:05	600	360	792	840
21/05/15 22:10	600	360	576	840
21/05/15 22:15	600	360	552	840
21/05/15 22:20	600	360	744	840
21/05/15 22:25	600	360	840	840
21/05/15 22:30	624	360	1008	840
21/05/15 22:35	720	360	876	840
21/05/15 22:40	720	360	852	840
21/05/15 22:45	696	336	756	816
21/05/15 22:50	600	240	612	720
21/05/15 22:55	600	240	456	720
21/05/15 23:00	576	240	480	720
21/05/15 23:05	480	240	576	720
21/05/15 23:10	480	240	492	720
21/05/15 23:15	456	240	432	672
21/05/15 23:20	360	240	408	480
21/05/15 23:25	360	240	288	480
21/05/15 23:30	336	240	348	456
21/05/15 23:35	240	240	252	360
21/05/15 23:40	240	240	288	360
21/05/15 23:45	240	216	444	360
21/05/15 23:50	240	120	228	360
21/05/15 23:55	240	120	288	360
22/05/15 00:00	240	120	252	336
22/05/15 00:05	240	120	348	240
22/05/15 00:10	240	120	240	240
22/05/15 00:15	216	120	264	240
22/05/15 00:20	120	120	360	240
22/05/15 00:25	120	120	360	240
22/05/15 00:30	120	120	396	240
22/05/15 00:35	120	120	360	240
22/05/15 00:40	120	120	204	240
22/05/15 00:45	120	120	252	216
22/05/15 00:50	120	120	216	120
22/05/15 00:55	120	120	144	120
22/05/15 01:00	120	96	180	120
22/05/15 01:05	120	0	156	120
22/05/15 01:10	120	0	228	120
22/05/15 01:15	96	0	168	120
22/05/15 01:20	0	0	192	120
22/05/15 01:25	0	0	108	120
22/05/15 01:30	0	0	108	96
22/05/15 01:35	0	0	132	0
22/05/15 01:40	0	0	132	0
22/05/15 01:45	0	0	84	24
22/05/15 01:50	0	0	60	120
22/05/15 01:55	0	0	48	120
22/05/15 02:00	0	0	120	96
22/05/15 02:05	0	0	120	0
22/05/15 02:10	0	0	168	0
22/05/15 02:15	0	0	60	0
22/05/15 02:20	0	0	108	0
22/05/15 02:25	0	0	72	0
22/05/15 02:30	0	0	48	0
22/05/15 02:35	0	0	24	0
22/05/15 02:40	0	0	24	0

Sistema de Controle de Tráfego Urbano OPTIMUS

5 MINUTOS	INTENSIDADE			
	PM 040100 3	PM 040100 4	PM 040100 6	PM 040200 1
22/05/15 02:45	0	0	132	0
22/05/15 02:50	0	0	96	0
22/05/15 02:55	0	0	24	0
22/05/15 03:00	0	0	96	0
22/05/15 03:05	0	0	72	0
22/05/15 03:10	0	0	48	0
22/05/15 03:15	0	0	48	0
22/05/15 03:20	0	0	36	0
22/05/15 03:25	0	0	36	0
22/05/15 03:30	0	0	36	0
22/05/15 03:35	0	0	84	0
22/05/15 03:40	0	0	108	0
22/05/15 03:45	0	0	108	0
22/05/15 03:50	0	0	36	0
22/05/15 03:55	0	0	48	0
22/05/15 04:00	0	0	24	0
22/05/15 04:05	0	0	132	0
22/05/15 04:10	0	0	48	0
22/05/15 04:15	0	0	84	0
22/05/15 04:20	0	0	24	0
22/05/15 04:25	0	0	60	0
22/05/15 04:30	0	0	24	0
22/05/15 04:35	0	0	12	0
22/05/15 04:40	0	0	60	0
22/05/15 04:45	0	0	48	0
22/05/15 04:50	0	0	60	0
22/05/15 04:55	0	0	12	0
22/05/15 05:00	0	0	36	0
22/05/15 05:05	0	0	60	0
22/05/15 05:10	0	0	12	0
22/05/15 05:15	0	0	48	24
22/05/15 05:20	0	0	36	120
22/05/15 05:25	0	0	60	120
22/05/15 05:30	24	24	96	120
22/05/15 05:35	120	120	84	120
22/05/15 05:40	120	120	132	120
22/05/15 05:45	120	120	132	144
22/05/15 05:50	120	120	144	240
22/05/15 05:55	120	120	72	240
22/05/15 06:00	144	144	216	288
22/05/15 06:05	240	240	96	480
22/05/15 06:10	240	240	228	480
22/05/15 06:15	264	264	288	504
22/05/15 06:20	360	360	252	600
22/05/15 06:25	360	360	288	600
22/05/15 06:30	432	360	324	672
22/05/15 06:35	720	360	312	960
22/05/15 06:40	720	360	612	960
22/05/15 06:45	792	432	660	1080
22/05/15 06:50	1080	720	660	1560
22/05/15 06:55	1080	720	948	1560
22/05/15 07:00	1152	696	1152	1608
22/05/15 07:05	1440	600	912	1800
22/05/15 07:10	1440	600	756	1800
22/05/15 07:15	1464	600	828	1824
22/05/15 07:20	1560	600	1080	1920
22/05/15 07:25	1560	600	792	1920
22/05/15 07:30	1512	600	624	1896

Sistema de Controle de Tráfego Urbano OPTIMUS

5 MINUTOS	INTENSIDADE			
	PM 0401003	PM 0401004	PM 0401006	PM 0402001
22/05/15 07:35	1320	600	696	1800
22/05/15 07:40	1320	600	924	1800
22/05/15 07:45	1344	600	924	1800
22/05/15 07:50	1440	600	768	1800
22/05/15 07:55	1440	600	792	1800
22/05/15 08:00	1416	600	924	1800
22/05/15 08:05	1320	600	696	1800
22/05/15 08:10	1320	600	756	1800
22/05/15 08:15	1320	600	768	1776
22/05/15 08:20	1320	600	804	1680
22/05/15 08:25	1320	600	648	1680
22/05/15 08:30	1296	600	816	1680
22/05/15 08:35	1200	600	780	1680
22/05/15 08:40	1200	600	540	1680
22/05/15 08:45	1224	600	780	1704
22/05/15 08:50	1320	600	888	1800
22/05/15 08:55	1320	600	984	1800
22/05/15 09:00	1320	600	1308	1776
22/05/15 09:05	1320	600	840	1680
22/05/15 09:10	1320	600	1020	1680
22/05/15 09:15	1296	600	864	1656
22/05/15 09:20	1200	600	936	1560
22/05/15 09:25	1200	600	924	1560
22/05/15 09:30	1200	624	960	1560
22/05/15 09:35	1200	720	1044	1560
22/05/15 09:40	1200	720	1224	1560
22/05/15 09:45	1176	720	996	1560
22/05/15 09:50	1080	720	792	1560
22/05/15 09:55	1080	720	1080	1560
22/05/15 10:00	1080	696	852	1536
22/05/15 10:05	1080	600	936	1440
22/05/15 10:10	1080	600	1032	1440
22/05/15 10:15	1080	600	1044	1440
22/05/15 10:20	1080	600	816	1440
22/05/15 10:25	1080	600	936	1440
22/05/15 10:30	1056	600	1008	1416
22/05/15 10:35	960	600	936	1320
22/05/15 10:40	960	600	996	1320
22/05/15 10:45	984	600	960	1320
22/05/15 10:50	1080	600	948	1320
22/05/15 10:55	1080	600	756	1320
22/05/15 11:00	1056	600	1404	1320
22/05/15 11:05	960	600	1080	1320
22/05/15 11:10	960	600	1164	1320
22/05/15 11:15	984	600	1596	1344
22/05/15 11:20	1080	600	996	1440
22/05/15 11:25	1080	600	1560	1440
22/05/15 11:30	1080	600	1308	1440
22/05/15 11:35	1080	600	1320	1440
22/05/15 11:40	1080	600	1368	1440
22/05/15 11:45	1080	600	1224	1416
22/05/15 11:50	1080	600	1452	1320
22/05/15 11:55	1080	600	1488	1320
22/05/15 12:00	1104	600	1632	1344
22/05/15 12:05	1200	600	1284	1440
22/05/15 12:10	1200	600	1188	1440
22/05/15 12:15	1200	600	1368	1440
22/05/15 12:20	1200	600	1284	1440

Sistema de Controle de Tráfego Urbano OPTIMUS

5 MINUTOS	INTENSIDADE			
	PM 040100 3	PM 040100 4	PM 040100 6	PM 040200 1
22/05/15 12:25	1200	600	1308	1440
22/05/15 12:30	1176	600	1608	1440
22/05/15 12:35	1080	600	1464	1440
22/05/15 12:40	1080	600	1236	1440
22/05/15 12:45	1104	624	1332	1464
22/05/15 12:50	1200	720	1200	1560
22/05/15 12:55	1200	720	1296	1560
22/05/15 13:00	1224	696	1428	1584
22/05/15 13:05	1320	600	1368	1680
22/05/15 13:10	1320	600	1224	1680
22/05/15 13:15	1320	600	1200	1656
22/05/15 13:20	1320	600	1164	1560
22/05/15 13:25	1320	600	1332	1560
22/05/15 13:30	1320	600	1236	1560
22/05/15 13:35	1320	600	1392	1560
22/05/15 13:40	1320	600	1092	1560
22/05/15 13:45	1296	600	1068	1560
22/05/15 13:50	1200	600	1404	1560
22/05/15 13:55	1200	600	1092	1560
22/05/15 14:00	1176	624	1320	1560
22/05/15 14:05	1080	720	1380	1560
22/05/15 14:10	1080	720	1176	1560
22/05/15 14:15	1104	696	960	1560
22/05/15 14:20	1200	600	1152	1560
22/05/15 14:25	1200	600	1236	1560
22/05/15 14:30	1176	600	1512	1536
22/05/15 14:35	1080	600	1140	1440
22/05/15 14:40	1080	600	1296	1440
22/05/15 14:45	1104	600	1584	1440
22/05/15 14:50	1200	600	1272	1440
22/05/15 14:55	1200	600	1284	1440
22/05/15 15:00	1200	600	1380	1440
22/05/15 15:05	1200	600	1308	1440
22/05/15 15:10	1200	600	1440	1440
22/05/15 15:15	1176	576	1236	1416
22/05/15 15:20	1080	480	1512	1320
22/05/15 15:25	1080	480	1464	1320
22/05/15 15:30	1056	480	1572	1296
22/05/15 15:35	960	480	1404	1200
22/05/15 15:40	960	480	1296	1200
22/05/15 15:45	960	480	1584	1200
22/05/15 15:50	960	480	1596	1200
22/05/15 15:55	960	480	1320	1200
22/05/15 16:00	960	480	1200	1200
22/05/15 16:05	960	480	1128	1200
22/05/15 16:10	960	480	1236	1200
22/05/15 16:15	960	480	1416	1200
22/05/15 16:20	960	480	1704	1200
22/05/15 16:25	960	480	1416	1200
22/05/15 16:30	984	480	1416	1224
22/05/15 16:35	1080	480	1620	1320
22/05/15 16:40	1080	480	1608	1320
22/05/15 16:45	1056	504	1704	1296
22/05/15 16:50	960	600	1872	1200
22/05/15 16:55	960	600	1764	1200
22/05/15 17:00	984	576	1704	1224
22/05/15 17:05	1080	480	1704	1320
22/05/15 17:10	1080	480	1620	1320

Sistema de Controle de Tráfego Urbano OPTIMUS

5 MINUTOS	INTENSIDADE			
	PM 0401003	PM 0401004	PM 0401006	PM 0402001
22/05/15 17:15	1056	480	1596	1272
22/05/15 17:20	960	480	1680	1080
22/05/15 17:25	960	480	1584	1080
22/05/15 17:30	960	480	1500	1104
22/05/15 17:35	960	480	1140	1200
22/05/15 17:40	960	480	1716	1200
22/05/15 17:45	936	480	1176	1128
22/05/15 17:50	840	480	1524	840
22/05/15 17:55	840	480	864	840
22/05/15 18:00	864	480	1728	888
22/05/15 18:05	960	480	1500	1080
22/05/15 18:10	960	480	2028	1080
22/05/15 18:15	1008	456	1584	1128
22/05/15 18:20	1200	360	1692	1320
22/05/15 18:25	1200	360	1740	1320
22/05/15 18:30	1152	384	1644	1296
22/05/15 18:35	960	480	1632	1200
22/05/15 18:40	960	480	1704	1200
22/05/15 18:45	936	480	1188	1176
22/05/15 18:50	840	480	1392	1080
22/05/15 18:55	840	480	1476	1080
22/05/15 19:00	912	480	1452	1128
22/05/15 19:05	1200	480	1452	1320
22/05/15 19:10	1200	480	1080	1320
22/05/15 19:15	1176	504	1380	1344
22/05/15 19:20	1080	600	1368	1440
22/05/15 19:25	1080	600	1020	1440
22/05/15 19:30	1080	600	1296	1440
22/05/15 19:35	1080	600	1332	1440
22/05/15 19:40	1080	600	1092	1440
22/05/15 19:45	1080	600	1080	1416
22/05/15 19:50	1080	600	1248	1320
22/05/15 19:55	1080	600	984	1320
22/05/15 20:00	1056	600	1044	1320
22/05/15 20:05	960	600	1176	1320
22/05/15 20:10	960	600	900	1320
22/05/15 20:15	960	600	1116	1320
22/05/15 20:20	960	600	876	1320
22/05/15 20:25	960	600	888	1320
22/05/15 20:30	960	600	996	1320
22/05/15 20:35	960	600	804	1320
22/05/15 20:40	960	600	1020	1320
22/05/15 20:45	936	600	1056	1320
22/05/15 20:50	840	600	876	1320
22/05/15 20:55	840	600	780	1320
22/05/15 21:00	864	600	960	1320
22/05/15 21:05	960	600	960	1320
22/05/15 21:10	960	600	960	1320
22/05/15 21:15	912	576	960	1272
22/05/15 21:20	720	480	960	1080
22/05/15 21:25	720	480	960	1080
22/05/15 21:30	720	480	936	1056
22/05/15 21:35	720	480	840	960
22/05/15 21:40	720	480	840	960
22/05/15 21:45	696	480	816	960
22/05/15 21:50	600	480	720	960
22/05/15 21:55	600	480	720	960
22/05/15 22:00	624	480	720	960

Sistema de Controle de Tráfego Urbano OPTIMUS

5 MINUTOS	INTENSIDADE			
	PM 0401003	PM 0401004	PM 0401006	PM 0402001
22/05/15 22:05	720	480	720	960
22/05/15 22:10	720	480	756	960
22/05/15 22:15	720	456	840	936
22/05/15 22:20	720	360	720	840
22/05/15 22:25	720	360	828	840
22/05/15 22:30	696	384	924	864
22/05/15 22:35	600	480	900	960
22/05/15 22:40	600	480	972	960
22/05/15 22:45	600	456	888	936
22/05/15 22:50	600	360	984	840
22/05/15 22:55	600	360	792	840
22/05/15 23:00	576	360	672	816
22/05/15 23:05	480	360	780	720
22/05/15 23:10	480	360	600	720
22/05/15 23:15	480	336	672	696
22/05/15 23:20	480	240	504	600
22/05/15 23:25	480	240	636	600
22/05/15 23:30	456	240	456	600
22/05/15 23:35	360	240	756	600
22/05/15 23:40	360	240	408	600
22/05/15 23:45	360	240	576	600
22/05/15 23:50	360	240	576	600
22/05/15 23:55	360	240	420	600
23/05/15 00:00	336	240	336	576
23/05/15 00:05	240	240	516	480
23/05/15 00:10	240	240	456	480
23/05/15 00:15	240	216	456	456
23/05/15 00:20	240	120	444	360
23/05/15 00:25	240	120	360	360
23/05/15 00:30	264	120	456	360
23/05/15 00:35	360	120	240	360
23/05/15 00:40	360	120	192	360
23/05/15 00:45	336	120	456	360
23/05/15 00:50	240	120	480	360
23/05/15 00:55	240	120	336	360
23/05/15 01:00	240	120	324	336
23/05/15 01:05	240	120	324	240
23/05/15 01:10	240	120	372	240
23/05/15 01:15	240	120	372	240
23/05/15 01:20	240	120	240	240
23/05/15 01:25	240	120	228	240
23/05/15 01:30	216	120	180	240
23/05/15 01:35	120	120	228	240
23/05/15 01:40	120	120	264	240
23/05/15 01:45	120	120	204	240
23/05/15 01:50	120	120	228	240
23/05/15 01:55	120	120	240	240
23/05/15 02:00	120	96	180	216
23/05/15 02:05	120	0	180	120
23/05/15 02:10	120	0	144	120
23/05/15 02:15	120	0	324	120
23/05/15 02:20	120	0	156	120
23/05/15 02:25	120	0	132	120
23/05/15 02:30	120	0	192	120
23/05/15 02:35	120	0	132	120
23/05/15 02:40	120	0	144	120
23/05/15 02:45	120	0	96	120
23/05/15 02:50	120	0	168	120

Sistema de Controle de Tráfego Urbano OPTIMUS

5 MINUTOS	INTENSIDADE			
	PM 0401003	PM 0401004	PM 0401006	PM 0402001
23/05/15 02:55	120	0	132	120
23/05/15 03:00	120	0	156	120
23/05/15 03:05	120	0	120	120
23/05/15 03:10	120	0	192	120
23/05/15 03:15	96	0	144	120
23/05/15 03:20	0	0	84	120
23/05/15 03:25	0	0	108	120
23/05/15 03:30	0	0	84	120
23/05/15 03:35	0	0	144	120
23/05/15 03:40	0	0	132	120
23/05/15 03:45	0	0	108	120
23/05/15 03:50	0	0	132	120
23/05/15 03:55	0	0	60	120
23/05/15 04:00	0	0	120	120
23/05/15 04:05	0	0	48	120
23/05/15 04:10	0	0	84	120
23/05/15 04:15	0	0	108	120
23/05/15 04:20	0	0	156	120
23/05/15 04:25	0	0	108	120
23/05/15 04:30	0	0	120	96
23/05/15 04:35	0	0	84	0
23/05/15 04:40	0	0	300	0
23/05/15 04:45	0	0	96	0
23/05/15 04:50	0	0	144	0
23/05/15 04:55	0	0	144	0
23/05/15 05:00	0	0	144	24
23/05/15 05:05	0	0	108	120
23/05/15 05:10	0	0	120	120
23/05/15 05:15	0	0	156	120
23/05/15 05:20	0	0	120	120
23/05/15 05:25	0	0	72	120
23/05/15 05:30	0	0	108	120
23/05/15 05:35	0	0	84	120
23/05/15 05:40	0	0	132	120
23/05/15 05:45	24	24	228	144
23/05/15 05:50	120	120	168	240
23/05/15 05:55	120	120	96	240
23/05/15 06:00	120	120	144	240
23/05/15 06:05	120	120	132	240
23/05/15 06:10	120	120	108	240
23/05/15 06:15	120	120	96	240
23/05/15 06:20	120	120	180	240
23/05/15 06:25	120	120	156	240
23/05/15 06:30	144	144	240	264
23/05/15 06:35	240	240	84	360
23/05/15 06:40	240	240	120	360
23/05/15 06:45	264	240	216	384
23/05/15 06:50	360	240	132	480
23/05/15 06:55	360	240	264	480
23/05/15 07:00	384	264	228	552
23/05/15 07:05	480	360	240	840
23/05/15 07:10	480	360	240	840
23/05/15 07:15	480	360	264	816
23/05/15 07:20	480	360	360	720
23/05/15 07:25	480	360	360	720
23/05/15 07:30	480	360	360	744
23/05/15 07:35	480	360	360	840
23/05/15 07:40	480	360	360	840

Sistema de Controle de Tráfego Urbano OPTIMUS

5 MINUTOS	INTENSIDADE			
	PM 0401003	PM 0401004	PM 0401006	PM 0402001
23/05/15 07:45	504	384	360	888
23/05/15 07:50	600	480	360	1080
23/05/15 07:55	600	480	360	1080
23/05/15 08:00	648	504	384	1104
23/05/15 08:05	840	600	504	1200
23/05/15 08:10	840	600	492	1200
23/05/15 08:15	816	576	540	1200
23/05/15 08:20	720	480	444	1200
23/05/15 08:25	720	480	648	1200
23/05/15 08:30	744	504	492	1176
23/05/15 08:35	840	600	492	1080
23/05/15 08:40	840	600	744	1080
23/05/15 08:45	840	600	624	1104
23/05/15 08:50	840	600	600	1200
23/05/15 08:55	840	600	888	1200
23/05/15 09:00	864	600	828	1224
23/05/15 09:05	960	600	828	1320
23/05/15 09:10	960	600	696	1320
23/05/15 09:15	960	600	744	1320
23/05/15 09:20	960	600	948	1320
23/05/15 09:25	960	600	600	1320
23/05/15 09:30	984	600	528	1320
23/05/15 09:35	1080	600	660	1320
23/05/15 09:40	1080	600	696	1320
23/05/15 09:45	1080	600	792	1344
23/05/15 09:50	1080	600	804	1440
23/05/15 09:55	1080	600	900	1440
23/05/15 10:00	1080	600	684	1416
23/05/15 10:05	1080	600	960	1320
23/05/15 10:10	1080	600	960	1320
23/05/15 10:15	1080	600	936	1320
23/05/15 10:20	1080	600	840	1320
23/05/15 10:25	1080	600	732	1320
23/05/15 10:30	1080	600	780	1320
23/05/15 10:35	1080	600	1164	1320
23/05/15 10:40	1080	600	840	1320
23/05/15 10:45	1104	600	936	1344
23/05/15 10:50	1200	600	720	1440
23/05/15 10:55	1200	600	1008	1440
23/05/15 11:00	1200	600	768	1416
23/05/15 11:05	1200	600	972	1320
23/05/15 11:10	1200	600	984	1320
23/05/15 11:15	1200	600	1212	1344
23/05/15 11:20	1200	600	1152	1440
23/05/15 11:25	1200	600	1368	1440
23/05/15 11:30	1224	600	948	1440
23/05/15 11:35	1320	600	828	1440
23/05/15 11:40	1320	600	720	1440
23/05/15 11:45	1320	600	804	1440
23/05/15 11:50	1320	600	1080	1440
23/05/15 11:55	1320	600	1080	1440
23/05/15 12:00	1296	600	1080	1416
23/05/15 12:05	1200	600	1080	1320
23/05/15 12:10	1200	600	816	1320
23/05/15 12:15	1200	600	1080	1344
23/05/15 12:20	1200	600	1056	1440
23/05/15 12:25	1200	600	1272	1440
23/05/15 12:30	1200	600	1104	1416

Sistema de Controle de Tráfego Urbano OPTIMUS

5 MINUTOS	INTENSIDADE			
	PM 0401003	PM 0401004	PM 0401006	PM 0402001
23/05/15 12:35	1200	600	1176	1320
23/05/15 12:40	1200	600	1020	1320
23/05/15 12:45	1224	600	912	1344
23/05/15 12:50	1320	600	984	1440
23/05/15 12:55	1320	600	1380	1440
23/05/15 13:00	1296	624	1236	1464
23/05/15 13:05	1200	720	1128	1560
23/05/15 13:10	1200	720	1044	1560
23/05/15 13:15	1200	696	972	1536
23/05/15 13:20	1200	600	1092	1440
23/05/15 13:25	1200	600	1248	1440
23/05/15 13:30	1176	600	1164	1440
23/05/15 13:35	1080	600	1464	1440
23/05/15 13:40	1080	600	1188	1440
23/05/15 13:45	1080	600	1128	1416
23/05/15 13:50	1080	600	1008	1320
23/05/15 13:55	1080	600	1212	1320
23/05/15 14:00	1056	624	888	1344
23/05/15 14:05	960	720	1020	1440
23/05/15 14:10	960	720	948	1440
23/05/15 14:15	960	696	1068	1416
23/05/15 14:20	960	600	756	1320
23/05/15 14:25	960	600	792	1320
23/05/15 14:30	960	624	696	1320
23/05/15 14:35	960	720	708	1320
23/05/15 14:40	960	720	720	1320
23/05/15 14:45	960	696	792	1296
23/05/15 14:50	960	600	828	1200
23/05/15 14:55	960	600	1044	1200
23/05/15 15:00	936	600	684	1200
23/05/15 15:05	840	600	972	1200
23/05/15 15:10	840	600	996	1200
23/05/15 15:15	840	576	1116	1200
23/05/15 15:20	840	480	1128	1200
23/05/15 15:25	840	480	840	1200
23/05/15 15:30	816	480	936	1176
23/05/15 15:35	720	480	924	1080
23/05/15 15:40	720	480	948	1080
23/05/15 15:45	720	504	1092	1080
23/05/15 15:50	720	600	924	1080
23/05/15 15:55	720	600	996	1080
23/05/15 16:00	744	600	852	1104
23/05/15 16:05	840	600	696	1200
23/05/15 16:10	840	600	876	1200
23/05/15 16:15	840	600	612	1200
23/05/15 16:20	840	600	780	1200
23/05/15 16:25	840	600	936	1200
23/05/15 16:30	816	600	1080	1176
23/05/15 16:35	720	600	732	1080
23/05/15 16:40	720	600	960	1080
23/05/15 16:45	720	576	912	1080
23/05/15 16:50	720	480	1044	1080
23/05/15 16:55	720	480	1116	1080
23/05/15 17:00	744	480	1032	1080
23/05/15 17:05	840	480	840	1080
23/05/15 17:10	840	480	876	1080
23/05/15 17:15	840	504	876	1104
23/05/15 17:20	840	600	840	1200

Sistema de Controle de Tráfego Urbano OPTIMUS

5 MINUTOS	INTENSIDADE			
	PM 0401003	PM 0401004	PM 0401006	PM 0402001
23/05/15 17:25	840	600	912	1200
23/05/15 17:30	816	576	744	1176
23/05/15 17:35	720	480	1032	1080
23/05/15 17:40	720	480	924	1080
23/05/15 17:45	744	504	852	1104
23/05/15 17:50	840	600	1068	1200
23/05/15 17:55	840	600	1092	1200
23/05/15 18:00	840	600	1032	1200
23/05/15 18:05	840	600	1008	1200
23/05/15 18:10	840	600	852	1200
23/05/15 18:15	816	600	900	1176
23/05/15 18:20	720	600	948	1080
23/05/15 18:25	720	600	1056	1080
23/05/15 18:30	744	576	840	1104
23/05/15 18:35	840	480	840	1200
23/05/15 18:40	840	480	1176	1200
23/05/15 18:45	840	504	864	1200
23/05/15 18:50	840	600	924	1200
23/05/15 18:55	840	600	948	1200
23/05/15 19:00	864	600	828	1224
23/05/15 19:05	960	600	972	1320
23/05/15 19:10	960	600	756	1320
23/05/15 19:15	936	600	1152	1320
23/05/15 19:20	840	600	1212	1320
23/05/15 19:25	840	600	996	1320
23/05/15 19:30	840	600	804	1320
23/05/15 19:35	840	600	960	1320
23/05/15 19:40	840	600	684	1320
23/05/15 19:45	864	600	912	1296
23/05/15 19:50	960	600	888	1200
23/05/15 19:55	960	600	1032	1200
23/05/15 20:00	936	600	768	1200
23/05/15 20:05	840	600	864	1200
23/05/15 20:10	840	600	720	1200
23/05/15 20:15	840	600	768	1200
23/05/15 20:20	840	600	780	1200
23/05/15 20:25	840	600	684	1200
23/05/15 20:30	816	576	600	1176
23/05/15 20:35	720	480	696	1080
23/05/15 20:40	720	480	624	1080
23/05/15 20:45	720	480	720	1080
23/05/15 20:50	720	480	816	1080
23/05/15 20:55	720	480	744	1080
23/05/15 21:00	720	480	744	1080
23/05/15 21:05	720	480	888	1080
23/05/15 21:10	720	480	888	1080
23/05/15 21:15	696	456	828	1032
23/05/15 21:20	600	360	732	840
23/05/15 21:25	600	360	684	840
23/05/15 21:30	600	360	804	840
23/05/15 21:35	600	360	696	840
23/05/15 21:40	600	360	624	840
23/05/15 21:45	600	360	708	816
23/05/15 21:50	600	360	672	720
23/05/15 21:55	600	360	672	720
23/05/15 22:00	600	360	744	744
23/05/15 22:05	600	360	768	840
23/05/15 22:10	600	360	468	840

Sistema de Controle de Tráfego Urbano OPTIMUS

5 MINUTOS	INTENSIDADE			
	PM 0401003	PM 0401004	PM 0401006	PM 0402001
23/05/15 22:15	600	360	948	840
23/05/15 22:20	600	360	888	840
23/05/15 22:25	600	360	792	840
23/05/15 22:30	576	360	876	816
23/05/15 22:35	480	360	588	720
23/05/15 22:40	480	360	408	720
23/05/15 22:45	480	360	600	720
23/05/15 22:50	480	360	600	720
23/05/15 22:55	480	360	600	720
23/05/15 23:00	480	360	660	720
23/05/15 23:05	480	360	648	720
23/05/15 23:10	480	360	516	720
23/05/15 23:15	480	360	540	720
23/05/15 23:20	480	360	480	720
23/05/15 23:25	480	360	480	720
23/05/15 23:30	456	336	480	696
23/05/15 23:35	360	240	504	600
23/05/15 23:40	360	240	516	600
23/05/15 23:45	360	240	456	576
23/05/15 23:50	360	240	552	480
23/05/15 23:55	360	240	504	480
24/05/15 00:00	360	240	480	480
24/05/15 00:05	360	240	480	480
24/05/15 00:10	360	240	576	480
24/05/15 00:15	360	240	444	480
24/05/15 00:20	360	240	468	480
24/05/15 00:25	360	240	552	480
24/05/15 00:30	360	240	396	480
24/05/15 00:35	360	240	312	480
24/05/15 00:40	360	240	156	480
24/05/15 00:45	336	240	336	456
24/05/15 00:50	240	240	252	360
24/05/15 00:55	240	240	252	360
24/05/15 01:00	240	216	480	360
24/05/15 01:05	240	120	528	360
24/05/15 01:10	240	120	480	360
24/05/15 01:15	240	120	420	360
24/05/15 01:20	240	120	276	360
24/05/15 01:25	240	120	336	360
24/05/15 01:30	216	120	204	336
24/05/15 01:35	120	120	228	240
24/05/15 01:40	120	120	288	240
24/05/15 01:45	120	120	348	240
24/05/15 01:50	120	120	264	240
24/05/15 01:55	120	120	324	240
24/05/15 02:00	120	120	216	240
24/05/15 02:05	120	120	252	240
24/05/15 02:10	120	120	312	240
24/05/15 02:15	120	120	300	240
24/05/15 02:20	120	120	156	240
24/05/15 02:25	120	120	168	240
24/05/15 02:30	120	120	240	240
24/05/15 02:35	120	120	228	240
24/05/15 02:40	120	120	168	240
24/05/15 02:45	120	120	156	216
24/05/15 02:50	120	120	204	120
24/05/15 02:55	120	120	144	120
24/05/15 03:00	120	96	156	120

Sistema de Controle de Tráfego Urbano OPTIMUS

5 MINUTOS	INTENSIDADE			
	PM 0401003	PM 0401004	PM 0401006	PM 0402001
24/05/15 03:05	120	0	108	120
24/05/15 03:10	120	0	132	120
24/05/15 03:15	120	0	216	120
24/05/15 03:20	120	0	168	120
24/05/15 03:25	120	0	180	120
24/05/15 03:30	120	0	120	120
24/05/15 03:35	120	0	120	120
24/05/15 03:40	120	0	132	120
24/05/15 03:45	120	0	144	120
24/05/15 03:50	120	0	96	120
24/05/15 03:55	120	0	168	120
24/05/15 04:00	96	0	144	120
24/05/15 04:05	0	0	168	120
24/05/15 04:10	0	0	60	120
24/05/15 04:15	24	0	120	120
24/05/15 04:20	120	0	60	120
24/05/15 04:25	120	0	108	120
24/05/15 04:30	96	0	120	120
24/05/15 04:35	0	0	108	120
24/05/15 04:40	0	0	60	120
24/05/15 04:45	0	0	180	120
24/05/15 04:50	0	0	132	120
24/05/15 04:55	0	0	120	120
24/05/15 05:00	0	24	72	120
24/05/15 05:05	0	120	132	120
24/05/15 05:10	0	120	72	120
24/05/15 05:15	0	96	96	120
24/05/15 05:20	0	0	132	120
24/05/15 05:25	0	0	84	120
24/05/15 05:30	0	0	36	120
24/05/15 05:35	0	0	144	120
24/05/15 05:40	0	0	168	120
24/05/15 05:45	0	24	204	120
24/05/15 05:50	0	120	96	120
24/05/15 05:55	0	120	144	120
24/05/15 06:00	24	120	108	120
24/05/15 06:05	120	120	168	120
24/05/15 06:10	120	120	60	120
24/05/15 06:15	120	120	132	120
24/05/15 06:20	120	120	96	120
24/05/15 06:25	120	120	108	120
24/05/15 06:30	120	120	84	144
24/05/15 06:35	120	120	108	240
24/05/15 06:40	120	120	96	240
24/05/15 06:45	120	144	180	264
24/05/15 06:50	120	240	84	360
24/05/15 06:55	120	240	216	360
24/05/15 07:00	168	240	144	384
24/05/15 07:05	360	240	120	480
24/05/15 07:10	360	240	120	480
24/05/15 07:15	336	240	144	480
24/05/15 07:20	240	240	240	480
24/05/15 07:25	240	240	240	480
24/05/15 07:30	240	240	240	456
24/05/15 07:35	240	240	240	360
24/05/15 07:40	240	240	240	360
24/05/15 07:45	240	240	240	384
24/05/15 07:50	240	240	240	480

Sistema de Controle de Tráfego Urbano OPTIMUS

5 MINUTOS	INTENSIDADE			
	PM 0401003	PM 0401004	PM 0401006	PM 0402001
24/05/15 07:55	240	240	240	480
24/05/15 08:00	240	240	240	480
24/05/15 08:05	240	240	240	480
24/05/15 08:10	240	240	240	480
24/05/15 08:15	240	240	240	480
24/05/15 08:20	240	240	240	480
24/05/15 08:25	240	240	240	480
24/05/15 08:30	264	240	240	480
24/05/15 08:35	360	240	300	480
24/05/15 08:40	360	240	168	480
24/05/15 08:45	360	240	132	504
24/05/15 08:50	360	240	240	600
24/05/15 08:55	360	240	240	600
24/05/15 09:00	384	264	264	624
24/05/15 09:05	480	360	444	720
24/05/15 09:10	480	360	516	720
24/05/15 09:15	480	360	564	720
24/05/15 09:20	480	360	348	720
24/05/15 09:25	480	360	528	720
24/05/15 09:30	480	360	432	720
24/05/15 09:35	480	360	420	720
24/05/15 09:40	480	360	432	720
24/05/15 09:45	480	360	540	720
24/05/15 09:50	480	360	528	720
24/05/15 09:55	480	360	516	720
24/05/15 10:00	504	360	432	744
24/05/15 10:05	600	360	432	840
24/05/15 10:10	600	360	432	840
24/05/15 10:15	576	384	468	840
24/05/15 10:20	480	480	456	840
24/05/15 10:25	480	480	504	840
24/05/15 10:30	504	480	480	840
24/05/15 10:35	600	480	540	840
24/05/15 10:40	600	480	576	840
24/05/15 10:45	600	480	564	864
24/05/15 10:50	600	480	552	960
24/05/15 10:55	600	480	588	960
24/05/15 11:00	624	480	588	960
24/05/15 11:05	720	480	612	960
24/05/15 11:10	720	480	516	960
24/05/15 11:15	696	480	600	960
24/05/15 11:20	600	480	636	960
24/05/15 11:25	600	480	480	960
24/05/15 11:30	624	480	576	984
24/05/15 11:35	720	480	600	1080
24/05/15 11:40	720	480	600	1080
24/05/15 11:45	720	504	624	1080
24/05/15 11:50	720	600	624	1080
24/05/15 11:55	720	600	720	1080
24/05/15 12:00	744	600	660	1080
24/05/15 12:05	840	600	720	1080
24/05/15 12:10	840	600	720	1080
24/05/15 12:15	840	600	1092	1104
24/05/15 12:20	840	600	972	1200
24/05/15 12:25	840	600	864	1200
24/05/15 12:30	888	576	912	1224
24/05/15 12:35	1080	480	924	1320
24/05/15 12:40	1080	480	756	1320

Sistema de Controle de Tráfego Urbano OPTIMUS

5 MINUTOS	INTENSIDADE			
	PM 0401003	PM 0401004	PM 0401006	PM 0402001
24/05/15 12:45	1056	504	852	1320
24/05/15 12:50	960	600	948	1320
24/05/15 12:55	960	600	888	1320
24/05/15 13:00	960	576	612	1296
24/05/15 13:05	960	480	660	1200
24/05/15 13:10	960	480	840	1200
24/05/15 13:15	960	480	840	1200
24/05/15 13:20	960	480	840	1200
24/05/15 13:25	960	480	840	1200
24/05/15 13:30	912	480	840	1176
24/05/15 13:35	720	480	840	1080
24/05/15 13:40	720	480	828	1080
24/05/15 13:45	720	480	780	1080
24/05/15 13:50	720	480	720	1080
24/05/15 13:55	720	480	660	1080
24/05/15 14:00	720	456	804	1056
24/05/15 14:05	720	360	756	960
24/05/15 14:10	720	360	612	960
24/05/15 14:15	696	360	636	936
24/05/15 14:20	600	360	660	840
24/05/15 14:25	600	360	516	840
24/05/15 14:30	600	360	600	840
24/05/15 14:35	600	360	600	840
24/05/15 14:40	600	360	600	840
24/05/15 14:45	624	408	600	840
24/05/15 14:50	720	600	600	840
24/05/15 14:55	720	600	600	840
24/05/15 15:00	696	552	564	840
24/05/15 15:05	600	360	648	840
24/05/15 15:10	600	360	780	840
24/05/15 15:15	600	360	444	816
24/05/15 15:20	600	360	480	720
24/05/15 15:25	600	360	480	720
24/05/15 15:30	600	360	504	744
24/05/15 15:35	600	360	600	840
24/05/15 15:40	600	360	636	840
24/05/15 15:45	624	360	480	864
24/05/15 15:50	720	360	600	960
24/05/15 15:55	720	360	600	960
24/05/15 16:00	720	360	600	936
24/05/15 16:05	720	360	600	840
24/05/15 16:10	720	360	600	840
24/05/15 16:15	696	360	600	864
24/05/15 16:20	600	360	600	960
24/05/15 16:25	600	360	600	960
24/05/15 16:30	600	384	600	936
24/05/15 16:35	600	480	600	840
24/05/15 16:40	600	480	600	840
24/05/15 16:45	600	456	576	840
24/05/15 16:50	600	360	480	840
24/05/15 16:55	600	360	480	840
24/05/15 17:00	600	360	480	864
24/05/15 17:05	600	360	480	960
24/05/15 17:10	600	360	480	960
24/05/15 17:15	600	384	492	960
24/05/15 17:20	600	480	480	960
24/05/15 17:25	600	480	480	960
24/05/15 17:30	600	480	480	936

Sistema de Controle de Tráfego Urbano OPTIMUS

5 MINUTOS	INTENSIDADE			
	PM 0401003	PM 0401004	PM 0401006	PM 0402001
24/05/15 17:35	600	480	480	840
24/05/15 17:40	600	480	480	840
24/05/15 17:45	600	480	504	864
24/05/15 17:50	600	480	600	960
24/05/15 17:55	600	480	600	960
24/05/15 18:00	624	480	600	984
24/05/15 18:05	720	480	708	1080
24/05/15 18:10	720	480	900	1080
24/05/15 18:15	720	480	864	1080
24/05/15 18:20	720	480	972	1080
24/05/15 18:25	720	480	720	1080
24/05/15 18:30	744	480	720	1104
24/05/15 18:35	840	480	792	1200
24/05/15 18:40	840	480	600	1200
24/05/15 18:45	840	480	840	1200
24/05/15 18:50	840	480	828	1200
24/05/15 18:55	840	480	504	1200
24/05/15 19:00	840	504	816	1224
24/05/15 19:05	840	600	696	1320
24/05/15 19:10	840	600	888	1320
24/05/15 19:15	840	576	792	1296
24/05/15 19:20	840	480	888	1200
24/05/15 19:25	840	480	636	1200
24/05/15 19:30	840	480	648	1200
24/05/15 19:35	840	480	672	1200
24/05/15 19:40	840	480	840	1200
24/05/15 19:45	816	480	624	1152
24/05/15 19:50	720	480	684	960
24/05/15 19:55	720	480	612	960
24/05/15 20:00	696	456	660	936
24/05/15 20:05	600	360	492	840
24/05/15 20:10	600	360	720	840
24/05/15 20:15	600	360	720	840
24/05/15 20:20	600	360	720	840
24/05/15 20:25	600	360	576	840
24/05/15 20:30	600	360	696	840
24/05/15 20:35	600	360	780	840
24/05/15 20:40	600	360	612	840
24/05/15 20:45	600	360	720	840
24/05/15 20:50	600	360	720	840
24/05/15 20:55	600	360	720	840
24/05/15 21:00	600	360	720	816
24/05/15 21:05	600	360	720	720
24/05/15 21:10	600	360	720	720
24/05/15 21:15	576	360	720	720
24/05/15 21:20	480	360	720	720
24/05/15 21:25	480	360	720	720
24/05/15 21:30	480	360	696	720
24/05/15 21:35	480	360	600	720
24/05/15 21:40	480	360	480	720
24/05/15 21:45	456	336	492	696
24/05/15 21:50	360	240	456	600
24/05/15 21:55	360	240	480	600
24/05/15 22:00	360	240	480	600
24/05/15 22:05	360	240	480	600
24/05/15 22:10	360	240	480	600
24/05/15 22:15	360	240	480	576
24/05/15 22:20	360	240	480	480

Sistema de Controle de Tráfego Urbano OPTIMUS

5 MINUTOS	INTENSIDADE			
	PM 0401003	PM 0401004	PM 0401006	PM 0402001
24/05/15 22:25	360	240	480	480
24/05/15 22:30	360	216	456	480
24/05/15 22:35	360	120	360	480
24/05/15 22:40	360	120	360	480
24/05/15 22:45	336	120	360	456
24/05/15 22:50	240	120	360	360
24/05/15 22:55	240	120	360	360
24/05/15 23:00	240	120	336	360
24/05/15 23:05	240	120	240	360
24/05/15 23:10	240	120	240	360
24/05/15 23:15	240	120	240	360
24/05/15 23:20	240	120	240	360
24/05/15 23:25	240	108	240	240
24/05/15 23:30	216	192	240	228
24/05/15 23:35	192	168	240	312
24/05/15 23:40	144	108	240	204
24/05/15 23:45	108	108	240	144
24/05/15 23:50	192	108	240	252
24/05/15 23:55	144	132	240	180
25/05/15 00:00	96	144	240	168
25/05/15 00:05	144	180	240	240
25/05/15 00:10	228	108	240	240
25/05/15 00:15	144	144	216	216
25/05/15 00:20	156	48	120	192
25/05/15 00:25	96	48	120	108
25/05/15 00:30	132	24	120	132
25/05/15 00:35	84	48	120	72
25/05/15 00:40	96	120	120	180
25/05/15 00:45	84	48	120	108
25/05/15 00:50	24	48	120	48
25/05/15 00:55	84	60	120	84
25/05/15 01:00	0	48	96	12
25/05/15 01:05	96	12	0	84
25/05/15 01:10	72	84	0	108
25/05/15 01:15	84	48	0	120
25/05/15 01:20	132	12	0	96
25/05/15 01:25	48	72	0	96
25/05/15 01:30	96	48	0	60
25/05/15 01:35	24	48	0	36
25/05/15 01:40	24	48	0	48
25/05/15 01:45	36	24	0	60
25/05/15 01:50	60	12	0	72
25/05/15 01:55	24	48	0	48
25/05/15 02:00	48	12	0	48
25/05/15 02:05	0	12	0	12
25/05/15 02:10	48	24	0	48
25/05/15 02:15	24	36	0	60
25/05/15 02:20	60	36	0	72
25/05/15 02:25	36	48	0	60
25/05/15 02:30	24	12	0	48
25/05/15 02:35	12	24	0	24
25/05/15 02:40	36	24	0	36
25/05/15 02:45	12	24	0	24
25/05/15 02:50	60	48	0	72
25/05/15 02:55	36	0	0	12
25/05/15 03:00	12	36	0	72
25/05/15 03:05	0	12	0	12
25/05/15 03:10	24	12	0	24

Sistema de Controle de Tráfego Urbano OPTIMUS

5 MINUTOS	INTENSIDADE			
	PM 0401003	PM 0401004	PM 0401006	PM 0402001
25/05/15 03:15	36	24	0	60
25/05/15 03:20	24	24	0	36
25/05/15 03:25	24	0	0	0
25/05/15 03:30	0	24	0	24
25/05/15 03:35	12	0	0	12
25/05/15 03:40	12	48	0	48
25/05/15 03:45	0	48	0	48
25/05/15 03:50	24	12	0	24
25/05/15 03:55	12	36	0	24
25/05/15 04:00	12	24	0	36
25/05/15 04:05	0	24	0	12
25/05/15 04:10	36	36	0	36
25/05/15 04:15	60	60	0	108
25/05/15 04:20	12	12	0	12
25/05/15 04:25	36	60	0	60
25/05/15 04:30	36	12	0	48
25/05/15 04:35	12	96	0	96
25/05/15 04:40	48	48	0	36
25/05/15 04:45	72	96	0	108
25/05/15 04:50	36	48	0	84
25/05/15 04:55	48	24	0	72
25/05/15 05:00	48	72	0	60
25/05/15 05:05	36	108	0	132
25/05/15 05:10	96	132	0	168
25/05/15 05:15	24	72	0	72
25/05/15 05:20	144	300	0	180
25/05/15 05:25	108	84	0	144
25/05/15 05:30	156	204	0	288
25/05/15 05:35	156	180	0	312
25/05/15 05:40	216	276	0	372
25/05/15 05:45	216	204	0	396
25/05/15 05:50	348	264	0	432
25/05/15 05:55	408	312	0	564
25/05/15 06:00	516	228	24	624
25/05/15 06:05	492	264	120	624
25/05/15 06:10	372	396	120	600
25/05/15 06:15	456	528	120	828
25/05/15 06:20	612	444	120	900
25/05/15 06:25	1104	600	120	1332
25/05/15 06:30	912	624	144	1308
25/05/15 06:35	1140	696	240	1680
25/05/15 06:40	1188	636	240	1512
25/05/15 06:45	1476	720	264	2040
25/05/15 06:50	1524	792	360	2064
25/05/15 06:55	2004	576	360	2364
25/05/15 07:00	1476	828	432	2076
25/05/15 07:05	1344	936	720	1848
25/05/15 07:10	1212	828	720	1944
25/05/15 07:15	1860	696	744	2424
25/05/15 07:20	1536	768	840	1872
25/05/15 07:25	1596	648	840	2160
25/05/15 07:30	1332	720	840	1872
25/05/15 07:35	1584	660	840	2124
25/05/15 07:40	1536	744	840	2040
25/05/15 07:45	1644	672	840	2172
25/05/15 07:50	1596	720	840	2100
25/05/15 07:55	1656	456	840	2028
25/05/15 08:00	1428	612	816	1812

Sistema de Controle de Tráfego Urbano OPTIMUS

5 MINUTOS	INTENSIDADE			
	PM 0401003	PM 0401004	PM 0401006	PM 0402001
25/05/15 08:05	1344	768	720	1980
25/05/15 08:10	1212	816	720	1812
25/05/15 08:15	1392	624	744	1884
25/05/15 08:20	1296	684	840	1692
25/05/15 08:25	1188	744	840	1872
25/05/15 08:30	1404	732	816	1512
25/05/15 08:35	1368	612	720	2064
25/05/15 08:40	1356	648	720	1608
25/05/15 08:45	1224	672	720	1752
25/05/15 08:50	1896	588	720	2112
25/05/15 08:55	1440	732	720	1872
25/05/15 09:00	1332	636	720	1860
25/05/15 09:05	1452	888	720	1932
25/05/15 09:10	1188	588	720	1608
25/05/15 09:15	1092	576	744	1632
25/05/15 09:20	1464	720	840	1764
25/05/15 09:25	1176	744	840	1704
25/05/15 09:30	1416	684	840	1872
25/05/15 09:35	1236	648	840	1656
25/05/15 09:40	1140	732	840	1524
25/05/15 09:45	1260	780	840	1740
25/05/15 09:50	1116	648	840	1764
25/05/15 09:55	1128	660	840	1284
25/05/15 10:00	1248	576	840	1596
25/05/15 10:05	1092	696	840	1476
25/05/15 10:10	1068	708	840	1476
25/05/15 10:15	1116	780	840	1584
25/05/15 10:20	1188	636	840	1548
25/05/15 10:25	1188	876	840	1560
25/05/15 10:30	1068	552	840	1488
25/05/15 10:35	840	696	840	1608
25/05/15 10:40	996	720	840	1020
25/05/15 10:45	1104	624	840	1476
25/05/15 10:50	1008	576	840	1404
25/05/15 10:55	1080	660	804	1176
25/05/15 11:00	900	576	876	1512
25/05/15 11:05	984	684	960	1608
25/05/15 11:10	1212	660	960	1380
25/05/15 11:15	876	576	936	1296
25/05/15 11:20	1056	456	840	1176
25/05/15 11:25	1020	720	840	1356
25/05/15 11:30	1080	660	888	1416
25/05/15 11:35	1272	648	1080	1620
25/05/15 11:40	1200	828	1080	1536
25/05/15 11:45	1188	696	1080	1608
25/05/15 11:50	1092	840	1080	1356
25/05/15 11:55	1320	612	1080	1704
25/05/15 12:00	1368	804	1080	1644
25/05/15 12:05	1368	660	1080	1620
25/05/15 12:10	1092	720	1080	1548
25/05/15 12:15	1104	708	1104	1212
25/05/15 12:20	1116	792	1200	1632
25/05/15 12:25	1284	708	1200	1632
25/05/15 12:30	1308	900	1200	1656
25/05/15 12:35	1260	852	1200	1692
25/05/15 12:40	1188	648	1200	1680
25/05/15 12:45	1368	732	1176	1524
25/05/15 12:50	1404	540	984	1656

Sistema de Controle de Tráfego Urbano OPTIMUS

5 MINUTOS	INTENSIDADE			
	PM 0401003	PM 0401004	PM 0401006	PM 0402001
25/05/15 12:55	1428	684	744	1740
25/05/15 13:00	1332	864	924	1656
25/05/15 13:05	1188	708	1080	1644
25/05/15 13:10	1536	744	1080	1980
25/05/15 13:15	1392	576	1056	1716
25/05/15 13:20	1536	708	960	1884
25/05/15 13:25	1596	600	960	1980
25/05/15 13:30	1200	900	984	1752
25/05/15 13:35	1200	924	1080	1656
25/05/15 13:40	1296	732	1080	1824
25/05/15 13:45	1584	732	1056	1896
25/05/15 13:50	1440	708	960	1944
25/05/15 13:55	1320	828	960	1728
25/05/15 14:00	1500	744	984	1728
25/05/15 14:05	1272	648	708	1620
25/05/15 14:10	1356	600	1080	1644
25/05/15 14:15	1140	972	1080	1812
25/05/15 14:20	1128	792	1080	1644
25/05/15 14:25	1176	744	1080	1812
25/05/15 14:30	1032	660	1080	1344
25/05/15 14:35	1320	720	1080	1596
25/05/15 14:40	1104	900	1080	1716
25/05/15 14:45	1224	828	1080	1572
25/05/15 14:50	1212	660	1080	1440
25/05/15 14:55	1320	684	1080	1836
25/05/15 15:00	1008	840	1080	1404
25/05/15 15:05	1056	636	1080	1380
25/05/15 15:10	1068	684	1080	1476
25/05/15 15:15	1056	756	1104	1452
25/05/15 15:20	936	636	1200	1212
25/05/15 15:25	996	804	1200	1452
25/05/15 15:30	1056	552	1176	1512
25/05/15 15:35	1176	684	1080	1416
25/05/15 15:40	1116	852	1080	1740
25/05/15 15:45	1248	744	1104	1644
25/05/15 15:50	1200	576	1200	1284
25/05/15 15:55	1188	708	1200	1356
25/05/15 16:00	1068	672	1200	1164
25/05/15 16:05	1260	744	1200	1608
25/05/15 16:10	924	672	1212	1416
25/05/15 16:15	732	636	1056	1140
25/05/15 16:20	984	672	1200	1524
25/05/15 16:25	876	744	1200	1212
25/05/15 16:30	1308	576	1248	1308
25/05/15 16:35	996	756	1440	1572
25/05/15 16:40	1404	672	1440	1788
25/05/15 16:45	1092	720	1440	1440
25/05/15 16:50	1200	612	1440	1488
25/05/15 16:55	1248	684	1440	1344
25/05/15 17:00	1068	756	1488	1572
25/05/15 17:05	1140	720	1680	1620
25/05/15 17:10	1308	768	1680	1656
25/05/15 17:15	1116	888	1656	1548
25/05/15 17:20	1260	780	1560	1500
25/05/15 17:25	1032	876	1560	1200
25/05/15 17:30	504	732	1560	744
25/05/15 17:35	672	516	1560	600
25/05/15 17:40	672	396	1560	636

Sistema de Controle de Tráfego Urbano OPTIMUS

5 MINUTOS	INTENSIDADE			
	PM 0401003	PM 0401004	PM 0401006	PM 0402001
25/05/15 17:45	960	384	1536	996
25/05/15 17:50	1332	624	1440	1488
25/05/15 17:55	1656	600	1440	1740
25/05/15 18:00	1668	708	1464	1908
25/05/15 18:05	1404	576	1560	1704
25/05/15 18:10	1200	732	1560	1416
25/05/15 18:15	1308	492	1560	1404
25/05/15 18:20	1224	744	1560	1668
25/05/15 18:25	840	828	1560	1452
25/05/15 18:30	1008	840	1560	1296
25/05/15 18:35	1116	696	1560	1512
25/05/15 18:40	1104	516	1560	1584
25/05/15 18:45	1068	732	1560	1368
25/05/15 18:50	1212	816	1560	1512
25/05/15 18:55	1128	636	1560	1332
25/05/15 19:00	1140	552	1584	1476
25/05/15 19:05	960	576	1680	1404
25/05/15 19:10	1164	552	1680	1332
25/05/15 19:15	1296	768	1632	1680
25/05/15 19:20	1092	588	1440	1440
25/05/15 19:25	1032	660	1440	1224
25/05/15 19:30	1272	564	1464	1680
25/05/15 19:35	888	552	1560	1116
25/05/15 19:40	1080	492	1560	1128
25/05/15 19:45	936	588	1488	1164
25/05/15 19:50	804	516	1200	948
25/05/15 19:55	588	504	1200	1020
25/05/15 20:00	1080	600	1224	1260
25/05/15 20:05	696	516	1320	876
25/05/15 20:10	756	600	1320	948
25/05/15 20:15	684	444	1248	948
25/05/15 20:20	900	492	960	984
25/05/15 20:25	732	456	960	1020
25/05/15 20:30	792	216	936	792
25/05/15 20:35	732	396	840	912
25/05/15 20:40	804	588	840	948
25/05/15 20:45	708	312	816	1008
25/05/15 20:50	576	348	720	672
25/05/15 20:55	780	492	720	1032
25/05/15 21:00	600	396	720	696
25/05/15 21:05	576	456	720	804
25/05/15 21:10	600	444	720	840
25/05/15 21:15	504	348	744	672
25/05/15 21:20	588	192	840	696
25/05/15 21:25	636	456	840	864
25/05/15 21:30	516	348	816	588
25/05/15 21:35	660	444	720	936
25/05/15 21:40	516	372	720	684
25/05/15 21:45	624	300	720	756
25/05/15 21:50	636	300	720	780
25/05/15 21:55	552	432	720	756
25/05/15 22:00	660	396	720	876
25/05/15 22:05	444	204	720	480
25/05/15 22:10	564	420	720	792
25/05/15 22:15	756	432	720	876
25/05/15 22:20	720	360	720	900
25/05/15 22:25	684	336	720	888
25/05/15 22:30	540	276	720	708

Sistema de Controle de Tráfego Urbano OPTIMUS

5 MINUTOS	INTENSIDADE			
	PM 0401003	PM 0401004	PM 0401006	PM 0402001
25/05/15 22:35	444	372	720	600
25/05/15 22:40	732	228	720	852
25/05/15 22:45	576	240	744	696
25/05/15 22:50	672	228	840	708
25/05/15 22:55	456	180	840	540
25/05/15 23:00	432	228	792	504
25/05/15 23:05	276	144	600	348
25/05/15 23:10	300	180	600	468
25/05/15 23:15	228	168	552	300
25/05/15 23:20	240	180	360	324
25/05/15 23:25	204	144	300	252
25/05/15 23:30	180	144	216	288
25/05/15 23:35	192	108	264	180
25/05/15 23:40	168	144	228	276
25/05/15 23:45	228	72	252	252
25/05/15 23:50	132	192	204	192
25/05/15 23:55	144	60	192	156
26/05/15 00:00	180	84	300	192
26/05/15 00:05	96	120	180	144
26/05/15 00:10	120	84	264	168
26/05/15 00:15	96	120	168	204
26/05/15 00:20	132	72	96	168
26/05/15 00:25	96	36	132	108
26/05/15 00:30	156	84	96	156
26/05/15 00:35	48	72	108	84
26/05/15 00:40	84	108	108	60
26/05/15 00:45	96	108	120	168
26/05/15 00:50	24	24	60	12
26/05/15 00:55	48	60	108	60
26/05/15 01:00	48	36	120	72
26/05/15 01:05	12	36	72	12
26/05/15 01:10	60	24	84	60
26/05/15 01:15	60	48	48	60
26/05/15 01:20	84	84	72	72
26/05/15 01:25	84	60	96	108
26/05/15 01:30	24	24	36	48
26/05/15 01:35	96	24	108	60
26/05/15 01:40	48	24	48	48
26/05/15 01:45	36	24	60	12
26/05/15 01:50	36	24	36	36
26/05/15 01:55	12	48	36	48
26/05/15 02:00	24	12	60	36
26/05/15 02:05	60	36	24	48
26/05/15 02:10	24	60	72	48
26/05/15 02:15	36	36	24	60
26/05/15 02:20	12	72	24	60
26/05/15 02:25	48	0	72	48
26/05/15 02:30	48	36	48	60
26/05/15 02:35	12	36	24	12
26/05/15 02:40	12	12	36	24
26/05/15 02:45	36	12	12	12
26/05/15 02:50	0	24	48	12
26/05/15 02:55	24	0	36	12
26/05/15 03:00	24	12	12	24
26/05/15 03:05	12	0	12	12
26/05/15 03:10	72	72	0	72
26/05/15 03:15	36	36	72	72
26/05/15 03:20	0	12	48	24

Sistema de Controle de Tráfego Urbano OPTIMUS

5 MINUTOS	INTENSIDADE			
	PM 0401003	PM 0401004	PM 0401006	PM 0402001
26/05/15 03:25	12	24	12	24
26/05/15 03:30	0	36	0	12
26/05/15 03:35	12	72	24	48
26/05/15 03:40	12	0	0	0
26/05/15 03:45	0	24	48	24
26/05/15 03:50	24	36	24	24
26/05/15 03:55	0	24	0	48
26/05/15 04:00	36	0	0	24
26/05/15 04:05	12	36	12	48
26/05/15 04:10	24	12	24	60
26/05/15 04:15	24	48	24	48
26/05/15 04:20	36	24	0	60
26/05/15 04:25	24	72	12	72
26/05/15 04:30	24	60	36	60
26/05/15 04:35	24	48	12	48
26/05/15 04:40	72	48	0	48
26/05/15 04:45	60	60	24	96
26/05/15 04:50	48	84	36	108
26/05/15 04:55	48	36	12	36
26/05/15 05:00	48	48	36	36
26/05/15 05:05	60	60	24	132
26/05/15 05:10	84	60	84	96
26/05/15 05:15	72	60	48	108
26/05/15 05:20	60	108	72	108
26/05/15 05:25	108	108	60	216
26/05/15 05:30	204	168	84	204
26/05/15 05:35	132	240	36	276
26/05/15 05:40	348	192	84	456
26/05/15 05:45	144	168	120	276
26/05/15 05:50	336	276	72	468
26/05/15 05:55	168	300	60	468
26/05/15 06:00	588	276	108	684
26/05/15 06:05	348	288	144	540
26/05/15 06:10	420	360	156	564
26/05/15 06:15	564	432	168	876
26/05/15 06:20	624	504	132	852
26/05/15 06:25	852	588	180	1128
26/05/15 06:30	936	600	204	1656
26/05/15 06:35	744	564	276	1524
26/05/15 06:40	1296	720	288	1656
26/05/15 06:45	1452	804	396	1848
26/05/15 06:50	1884	540	444	2184
26/05/15 06:55	1752	720	516	2268
26/05/15 07:00	1704	528	564	2028
26/05/15 07:05	1488	960	720	2172
26/05/15 07:10	1896	588	720	2064
26/05/15 07:15	1260	960	744	2232
26/05/15 07:20	1740	648	840	2232
26/05/15 07:25	1464	780	840	1980
26/05/15 07:30	1764	528	840	2208
26/05/15 07:35	1368	804	852	2016
26/05/15 07:40	1812	552	756	2088
26/05/15 07:45	1464	852	744	2112
26/05/15 07:50	1776	492	744	2280
26/05/15 07:55	1380	636	768	1896
26/05/15 08:00	1344	744	720	2076
26/05/15 08:05	1428	768	744	1980
26/05/15 08:10	1656	564	600	2232

Sistema de Controle de Tráfego Urbano OPTIMUS

5 MINUTOS	INTENSIDADE			
	PM 0401003	PM 0401004	PM 0401006	PM 0402001
26/05/15 08:15	1332	708	624	1824
26/05/15 08:20	1488	828	672	2112
26/05/15 08:25	1344	720	840	1764
26/05/15 08:30	1536	720	840	2148
26/05/15 08:35	1464	732	840	1908
26/05/15 08:40	1416	504	828	1872
26/05/15 08:45	1392	732	588	1728
26/05/15 08:50	1692	588	792	2052
26/05/15 08:55	1440	720	720	1908
26/05/15 09:00	1308	672	972	1740
26/05/15 09:05	1332	564	612	1632
26/05/15 09:10	1392	792	648	1956
26/05/15 09:15	1104	804	564	1740
26/05/15 09:20	1236	660	756	1428
26/05/15 09:25	792	876	840	1560
26/05/15 09:30	1200	696	840	1560
26/05/15 09:35	1428	756	840	1836
26/05/15 09:40	1128	756	840	1704
26/05/15 09:45	1200	684	852	1644
26/05/15 09:50	1092	696	780	1428
26/05/15 09:55	924	600	1152	1392
26/05/15 10:00	1224	768	828	1512
26/05/15 10:05	1344	624	720	1632
26/05/15 10:10	1032	768	768	1620
26/05/15 10:15	972	708	756	1296
26/05/15 10:20	1152	684	804	1476
26/05/15 10:25	1296	672	660	1500
26/05/15 10:30	1008	528	864	1380
26/05/15 10:35	1212	660	960	1500
26/05/15 10:40	1044	612	960	1524
26/05/15 10:45	912	684	1116	1128
26/05/15 10:50	972	624	684	1404
26/05/15 10:55	1236	684	960	1704
26/05/15 11:00	948	576	984	1116
26/05/15 11:05	996	624	1080	1356
26/05/15 11:10	888	720	1080	1188
26/05/15 11:15	1176	612	1104	1380
26/05/15 11:20	1176	456	1200	1296
26/05/15 11:25	972	672	1200	1356
26/05/15 11:30	1188	600	1200	1416
26/05/15 11:35	960	852	1200	1560
26/05/15 11:40	1008	672	1200	1344
26/05/15 11:45	936	780	1224	1224
26/05/15 11:50	1284	576	1320	1500
26/05/15 11:55	1320	540	1320	1524
26/05/15 12:00	1200	660	1320	1476
26/05/15 12:05	1524	648	1320	1476
26/05/15 12:10	1128	564	1320	1416
26/05/15 12:15	1080	648	1320	1248
26/05/15 12:20	1080	576	1320	1368
26/05/15 12:25	1152	576	1320	1224
26/05/15 12:30	1272	564	1320	1548
26/05/15 12:35	984	624	1320	1368
26/05/15 12:40	1464	624	1320	1668
26/05/15 12:45	1404	672	1272	1548
26/05/15 12:50	1380	768	1080	1908
26/05/15 12:55	1188	792	1080	1668
26/05/15 13:00	1416	792	1080	2028

Sistema de Controle de Tráfego Urbano OPTIMUS

5 MINUTOS	INTENSIDADE			
	PM 0401003	PM 0401004	PM 0401006	PM 0402001
26/05/15 13:05	1452	744	1080	1764
26/05/15 13:10	1560	720	1080	1788
26/05/15 13:15	1344	744	1080	1824
26/05/15 13:20	1344	744	1080	1824
26/05/15 13:25	1452	684	1080	1716
26/05/15 13:30	1332	684	1104	1800
26/05/15 13:35	1440	660	1200	1620
26/05/15 13:40	1320	684	900	1788
26/05/15 13:45	1356	780	1164	1704
26/05/15 13:50	1284	804	864	1764
26/05/15 13:55	1200	756	756	1740
26/05/15 14:00	1176	708	852	1464
26/05/15 14:05	1404	636	852	1692
26/05/15 14:10	1272	780	1224	1932
26/05/15 14:15	1128	768	1176	1416
26/05/15 14:20	972	972	1080	1440
26/05/15 14:25	1056	696	1080	1428
26/05/15 14:30	1068	672	1056	1536
26/05/15 14:35	1296	720	960	1716
26/05/15 14:40	1188	648	960	1764
26/05/15 14:45	1200	792	984	1692
26/05/15 14:50	1128	684	1080	1500
26/05/15 14:55	1248	672	1080	1536
26/05/15 15:00	1164	804	1080	1656
26/05/15 15:05	1152	684	1080	1416
26/05/15 15:10	1056	708	1080	1608
26/05/15 15:15	1032	816	1080	1524
26/05/15 15:20	1260	636	1080	1452
26/05/15 15:25	1188	816	1080	1716
26/05/15 15:30	972	648	1080	1380
26/05/15 15:35	1500	540	1080	1728
26/05/15 15:40	1248	720	1080	1764
26/05/15 15:45	1224	756	1080	1632
26/05/15 15:50	1224	756	1080	1656
26/05/15 15:55	1164	816	1080	1788
26/05/15 16:00	876	792	1104	1176
26/05/15 16:05	1248	624	1200	1416
26/05/15 16:10	1236	612	1200	1584
26/05/15 16:15	1140	816	1224	1488
26/05/15 16:20	1152	900	1320	1668
26/05/15 16:25	1164	660	1320	1680
26/05/15 16:30	1092	564	1320	1356
26/05/15 16:35	936	648	1320	1380
26/05/15 16:40	1176	456	1320	1176
26/05/15 16:45	1176	528	1344	1164
26/05/15 16:50	1116	708	1440	1512
26/05/15 16:55	1032	708	1440	1260
26/05/15 17:00	1164	624	1440	1344
26/05/15 17:05	1212	768	1440	1632
26/05/15 17:10	1272	708	1440	1596
26/05/15 17:15	1164	852	1440	1656
26/05/15 17:20	1224	792	1440	1644
26/05/15 17:25	1176	612	1440	1368
26/05/15 17:30	900	852	1440	1428
26/05/15 17:35	876	768	1440	1116
26/05/15 17:40	936	552	1440	876
26/05/15 17:45	948	528	1464	1404
26/05/15 17:50	1128	576	1560	1212

Sistema de Controle de Tráfego Urbano OPTIMUS

5 MINUTOS	INTENSIDADE			
	PM 0401003	PM 0401004	PM 0401006	PM 0402001
26/05/15 17:55	1620	360	1560	1764
26/05/15 18:00	1404	420	1560	1404
26/05/15 18:05	1344	456	1560	1356
26/05/15 18:10	912	300	1560	852
26/05/15 18:15	1020	420	1512	1200
26/05/15 18:20	1152	492	1320	1452
26/05/15 18:25	1104	300	1320	1404
26/05/15 18:30	1188	696	1344	1068
26/05/15 18:35	504	372	1440	564
26/05/15 18:40	1008	444	1440	1368
26/05/15 18:45	1368	732	1440	1692
26/05/15 18:50	1296	624	1440	1524
26/05/15 18:55	1608	504	1440	1944
26/05/15 19:00	1200	708	1416	1524
26/05/15 19:05	1164	828	1320	1812
26/05/15 19:10	1236	660	1320	1656
26/05/15 19:15	1044	696	1344	1356
26/05/15 19:20	1200	744	1440	1344
26/05/15 19:25	888	732	1440	1500
26/05/15 19:30	1008	456	1440	1224
26/05/15 19:35	1008	624	1440	1428
26/05/15 19:40	888	480	1440	1092
26/05/15 19:45	1044	576	1368	1392
26/05/15 19:50	1020	468	1080	1164
26/05/15 19:55	696	732	1080	1188
26/05/15 20:00	1092	684	1080	1236
26/05/15 20:05	696	528	1080	972
26/05/15 20:10	900	408	1080	1068
26/05/15 20:15	768	672	1056	1128
26/05/15 20:20	948	372	960	1128
26/05/15 20:25	732	456	960	1116
26/05/15 20:30	744	420	960	1032
26/05/15 20:35	768	372	960	924
26/05/15 20:40	888	492	960	972
26/05/15 20:45	648	564	936	912
26/05/15 20:50	816	468	840	1140
26/05/15 20:55	696	588	840	1092
26/05/15 21:00	636	444	840	924
26/05/15 21:05	600	408	840	828
26/05/15 21:10	600	456	840	732
26/05/15 21:15	564	408	864	828
26/05/15 21:20	492	324	960	708
26/05/15 21:25	684	456	960	804
26/05/15 21:30	648	480	936	1020
26/05/15 21:35	624	408	840	684
26/05/15 21:40	612	228	840	744
26/05/15 21:45	660	432	816	852
26/05/15 21:50	576	324	720	768
26/05/15 21:55	588	408	720	780
26/05/15 22:00	696	396	720	840
26/05/15 22:05	552	312	720	648
26/05/15 22:10	648	348	720	960
26/05/15 22:15	732	528	720	912
26/05/15 22:20	696	264	720	840
26/05/15 22:25	564	468	720	924
26/05/15 22:30	672	372	720	900
26/05/15 22:35	612	264	720	720
26/05/15 22:40	540	276	720	696

Sistema de Controle de Tráfego Urbano OPTIMUS

5 MINUTOS	INTENSIDADE			
	PM 0401003	PM 0401004	PM 0401006	PM 0402001
26/05/15 22:45	480	300	720	576
26/05/15 22:50	672	228	720	636
26/05/15 22:55	552	204	720	588
26/05/15 23:00	468	240	720	588
26/05/15 23:05	300	264	720	444
26/05/15 23:10	300	228	720	456
26/05/15 23:15	240	132	696	336
26/05/15 23:20	288	228	600	360
26/05/15 23:25	276	156	600	312
26/05/15 23:30	252	204	576	360
26/05/15 23:35	168	276	480	420
26/05/15 23:40	216	132	480	324
26/05/15 23:45	204	72	456	192
26/05/15 23:50	168	132	360	180
26/05/15 23:55	216	192	360	336
27/05/15 00:00	192	108	336	228
27/05/15 00:05	132	84	240	156
27/05/15 00:10	156	132	240	228
27/05/15 00:15	132	36	240	156
27/05/15 00:20	168	108	240	204
27/05/15 00:25	120	108	240	168
27/05/15 00:30	156	84	216	168
27/05/15 00:35	144	96	120	144
27/05/15 00:40	132	108	120	204
27/05/15 00:45	108	72	96	144
27/05/15 00:50	72	84	0	120
27/05/15 00:55	132	36	0	84
27/05/15 01:00	72	48	0	108
27/05/15 01:05	36	36	0	84
27/05/15 01:10	60	48	0	60
27/05/15 01:15	36	36	0	84
27/05/15 01:20	60	24	0	96
27/05/15 01:25	96	12	0	108
27/05/15 01:30	72	48	0	72
27/05/15 01:35	60	48	0	72
27/05/15 01:40	48	24	0	84
27/05/15 01:45	60	60	0	72
27/05/15 01:50	48	96	0	132
27/05/15 01:55	60	36	0	108
27/05/15 02:00	36	24	0	24
27/05/15 02:05	24	0	0	36
27/05/15 02:10	48	36	0	60
27/05/15 02:15	72	60	0	84
27/05/15 02:20	96	60	0	180
27/05/15 02:25	48	24	0	84
27/05/15 02:30	48	36	0	72
27/05/15 02:35	72	12	0	72
27/05/15 02:40	36	0	0	36
27/05/15 02:45	84	36	0	84
27/05/15 02:50	0	24	0	24
27/05/15 02:55	12	36	0	60
27/05/15 03:00	12	0	0	0
27/05/15 03:05	0	24	0	24
27/05/15 03:10	36	60	0	72
27/05/15 03:15	24	60	0	48
27/05/15 03:20	24	36	0	72
27/05/15 03:25	0	24	0	24
27/05/15 03:30	12	60	0	60

Sistema de Controle de Tráfego Urbano OPTIMUS

5 MINUTOS	INTENSIDADE			
	PM 0401003	PM 0401004	PM 0401006	PM 0402001
27/05/15 03:35	0	24	0	24
27/05/15 03:40	12	36	0	48
27/05/15 03:45	36	24	0	48
27/05/15 03:50	12	36	0	36
27/05/15 03:55	24	36	0	48
27/05/15 04:00	48	0	0	24
27/05/15 04:05	48	0	0	24
27/05/15 04:10	60	72	0	84
27/05/15 04:15	48	72	0	108
27/05/15 04:20	0	12	0	24
27/05/15 04:25	24	48	0	48
27/05/15 04:30	36	24	0	36
27/05/15 04:35	48	60	0	84
27/05/15 04:40	48	72	0	36
27/05/15 04:45	48	108	0	144
27/05/15 04:50	60	36	0	60
27/05/15 04:55	60	60	0	96
27/05/15 05:00	72	36	0	108
27/05/15 05:05	36	84	0	72
27/05/15 05:10	60	132	0	144
27/05/15 05:15	36	84	0	120
27/05/15 05:20	180	72	0	120
27/05/15 05:25	108	132	0	180
27/05/15 05:30	120	156	0	216
27/05/15 05:35	204	192	0	336
27/05/15 05:40	228	252	0	348
27/05/15 05:45	180	156	0	324
27/05/15 05:50	396	312	0	648
27/05/15 05:55	432	252	0	660
27/05/15 06:00	504	396	0	756
27/05/15 06:05	396	300	0	648
27/05/15 06:10	468	576	0	744
27/05/15 06:15	624	612	0	1068
27/05/15 06:20	816	444	0	1056
27/05/15 06:25	948	504	0	1344
27/05/15 06:30	1128	660	0	1476
27/05/15 06:35	1248	684	0	1728
27/05/15 06:40	1272	900	0	1848
27/05/15 06:45	1344	768	24	2004
27/05/15 06:50	1824	840	120	2328
27/05/15 06:55	1620	672	120	2232
27/05/15 07:00	1824	672	168	2244
27/05/15 07:05	1560	660	360	1944
27/05/15 07:10	1800	696	360	2148
27/05/15 07:15	1620	636	432	2016
27/05/15 07:20	1584	804	720	2184
27/05/15 07:25	1596	720	720	2100
27/05/15 07:30	1740	696	744	2352
27/05/15 07:35	1356	708	840	1848
27/05/15 07:40	1428	840	840	2112
27/05/15 07:45	1680	612	816	2040
27/05/15 07:50	1536	756	720	2184
27/05/15 07:55	1488	816	720	1980
27/05/15 08:00	1560	672	720	2208
27/05/15 08:05	1356	612	720	1848
27/05/15 08:10	1404	756	720	1956
27/05/15 08:15	1488	672	720	1836
27/05/15 08:20	1536	720	720	2124

Sistema de Controle de Tráfego Urbano OPTIMUS

5 MINUTOS	INTENSIDADE			
	PM 0401003	PM 0401004	PM 0401006	PM 0402001
27/05/15 08:25	1500	720	720	1680
27/05/15 08:30	1416	660	720	1980
27/05/15 08:35	1344	540	720	1680
27/05/15 08:40	1392	852	720	1908
27/05/15 08:45	1176	960	720	1980
27/05/15 08:50	1800	492	720	2076
27/05/15 08:55	1272	900	720	2112
27/05/15 09:00	1440	552	720	1668
27/05/15 09:05	1344	708	720	1716
27/05/15 09:10	1428	492	720	1548
27/05/15 09:15	1296	648	744	1680
27/05/15 09:20	1368	576	840	1812
27/05/15 09:25	1332	792	840	1908
27/05/15 09:30	1224	684	816	1512
27/05/15 09:35	1176	540	720	1548
27/05/15 09:40	1200	768	720	1620
27/05/15 09:45	1164	600	720	1800
27/05/15 09:50	1200	528	720	1608
27/05/15 09:55	1128	744	720	1296
27/05/15 10:00	960	720	720	1596
27/05/15 10:05	1356	696	720	1680
27/05/15 10:10	1248	768	720	1800
27/05/15 10:15	1128	564	744	1584
27/05/15 10:20	1020	600	840	1308
27/05/15 10:25	1164	696	840	1560
27/05/15 10:30	840	720	840	1296
27/05/15 10:35	1140	576	840	1440
27/05/15 10:40	1140	720	840	1404
27/05/15 10:45	1116	804	864	1692
27/05/15 10:50	1152	576	960	1176
27/05/15 10:55	1188	624	960	1344
27/05/15 11:00	1020	528	960	1428
27/05/15 11:05	1464	636	960	1392
27/05/15 11:10	1056	600	960	1416
27/05/15 11:15	1152	708	960	1380
27/05/15 11:20	1236	684	960	1536
27/05/15 11:25	1212	588	960	1632
27/05/15 11:30	1164	516	960	1380
27/05/15 11:35	1164	648	960	1392
27/05/15 11:40	1008	624	960	1356
27/05/15 11:45	972	612	1008	1320
27/05/15 11:50	1152	528	1200	1260
27/05/15 11:55	1440	804	1200	1656
27/05/15 12:00	1440	564	1200	1620
27/05/15 12:05	1368	744	1200	1800
27/05/15 12:10	1332	684	1200	1776
27/05/15 12:15	1368	660	1200	1656
27/05/15 12:20	1296	528	1200	1524
27/05/15 12:25	1188	816	1200	1764
27/05/15 12:30	1200	744	1224	1308
27/05/15 12:35	1452	804	1320	1896
27/05/15 12:40	1596	708	1320	1824
27/05/15 12:45	1320	744	1320	1620
27/05/15 12:50	1656	528	1320	1728
27/05/15 12:55	1344	912	1320	1932
27/05/15 13:00	1488	672	1296	1884
27/05/15 13:05	1200	864	1200	1692
27/05/15 13:10	1404	528	1200	1632

Sistema de Controle de Tráfego Urbano OPTIMUS

5 MINUTOS	INTENSIDADE			
	PM 0401003	PM 0401004	PM 0401006	PM 0402001
27/05/15 13:15	1344	708	1176	1680
27/05/15 13:20	1368	816	1080	1764
27/05/15 13:25	1524	624	1080	1680
27/05/15 13:30	1428	696	1080	2028
27/05/15 13:35	1296	576	1080	1656
27/05/15 13:40	1332	792	1080	1860
27/05/15 13:45	1224	672	1056	1740
27/05/15 13:50	1236	888	960	1692
27/05/15 13:55	1416	576	960	1812
27/05/15 14:00	1212	648	960	1380
27/05/15 14:05	1272	768	960	1764
27/05/15 14:10	1044	840	960	1656
27/05/15 14:15	1152	780	936	1656
27/05/15 14:20	1164	756	840	1524
27/05/15 14:25	1032	720	840	1812
27/05/15 14:30	1056	636	864	1104
27/05/15 14:35	1176	864	960	1476
27/05/15 14:40	1344	552	960	1728
27/05/15 14:45	1260	828	960	1668
27/05/15 14:50	1320	804	960	1788
27/05/15 14:55	1104	540	960	1344
27/05/15 15:00	1056	672	984	1752
27/05/15 15:05	1128	912	1080	1512
27/05/15 15:10	1176	636	1080	1620
27/05/15 15:15	1092	684	1104	1404
27/05/15 15:20	1320	828	1200	1728
27/05/15 15:25	1164	768	1200	1860
27/05/15 15:30	1056	876	1008	1500
27/05/15 15:35	1140	612	816	1416
27/05/15 15:40	1032	624	816	1356
27/05/15 15:45	1128	696	720	1416
27/05/15 15:50	1164	660	1128	1488
27/05/15 15:55	1140	672	1200	1464
27/05/15 16:00	1104	600	1200	1392
27/05/15 16:05	1164	636	1200	1452
27/05/15 16:10	1092	756	1200	1536
27/05/15 16:15	1260	696	1200	1356
27/05/15 16:20	912	624	1200	1524
27/05/15 16:25	924	660	1200	1296
27/05/15 16:30	1140	828	1224	1404
27/05/15 16:35	1140	708	1320	1704
27/05/15 16:40	1092	468	1320	1332
27/05/15 16:45	1020	792	1320	1368
27/05/15 16:50	1008	720	1320	1404
27/05/15 16:55	1080	780	1320	1476
27/05/15 17:00	1212	600	1344	1476
27/05/15 17:05	1392	780	1440	1644
27/05/15 17:10	1368	756	1440	1692
27/05/15 17:15	1224	876	1440	1884
27/05/15 17:20	1212	756	1440	1632
27/05/15 17:25	684	648	1440	864
27/05/15 17:30	840	540	1464	1080
27/05/15 17:35	996	672	1560	1236
27/05/15 17:40	1080	420	1560	1092
27/05/15 17:45	1428	684	1560	1644
27/05/15 17:50	1236	420	1560	1428
27/05/15 17:55	1068	540	1560	1332
27/05/15 18:00	1272	360	1536	1332

Sistema de Controle de Tráfego Urbano OPTIMUS

5 MINUTOS	INTENSIDADE			
	PM 0401003	PM 0401004	PM 0401006	PM 0402001
27/05/15 18:05	696	708	1440	984
27/05/15 18:10	900	420	1440	1032
27/05/15 18:15	924	648	1464	1080
27/05/15 18:20	924	204	1560	840
27/05/15 18:25	480	300	1560	432
27/05/15 18:30	252	168	1536	804
27/05/15 18:35	288	204	1440	516
27/05/15 18:40	1356	420	1440	1248
27/05/15 18:45	1476	480	1464	1728
27/05/15 18:50	1500	456	1392	1536
27/05/15 18:55	504	324	1560	600
27/05/15 19:00	1128	228	1512	1092
27/05/15 19:05	1644	576	1320	2040
27/05/15 19:10	1536	480	1320	1944
27/05/15 19:15	888	864	1368	1416
27/05/15 19:20	1068	660	1560	1344
27/05/15 19:25	1116	612	1560	1524
27/05/15 19:30	1080	732	1536	1524
27/05/15 19:35	864	624	1440	1248
27/05/15 19:40	780	420	1440	972
27/05/15 19:45	936	648	1392	1404
27/05/15 19:50	912	564	1200	1128
27/05/15 19:55	828	576	1200	1212
27/05/15 20:00	972	588	1200	1284
27/05/15 20:05	720	564	1200	960
27/05/15 20:10	684	384	1200	888
27/05/15 20:15	804	588	1176	1200
27/05/15 20:20	876	432	1080	1020
27/05/15 20:25	708	456	1080	936
27/05/15 20:30	876	516	1056	996
27/05/15 20:35	624	468	960	996
27/05/15 20:40	1020	396	960	1092
27/05/15 20:45	828	420	936	1056
27/05/15 20:50	660	456	840	864
27/05/15 20:55	516	408	840	756
27/05/15 21:00	696	408	840	744
27/05/15 21:05	792	396	840	1044
27/05/15 21:10	624	384	840	876
27/05/15 21:15	708	444	840	864
27/05/15 21:20	660	492	840	912
27/05/15 21:25	624	408	840	804
27/05/15 21:30	648	396	840	864
27/05/15 21:35	576	372	840	732
27/05/15 21:40	528	384	840	744
27/05/15 21:45	660	480	792	840
27/05/15 21:50	804	372	600	876
27/05/15 21:55	564	420	600	888
27/05/15 22:00	588	456	600	804
27/05/15 22:05	552	372	600	672
27/05/15 22:10	384	372	600	624
27/05/15 22:15	468	396	624	600
27/05/15 22:20	672	264	720	864
27/05/15 22:25	636	300	720	768
27/05/15 22:30	528	276	720	660
27/05/15 22:35	408	240	720	588
27/05/15 22:40	600	156	720	624
27/05/15 22:45	480	180	720	600
27/05/15 22:50	444	228	720	528

Sistema de Controle de Tráfego Urbano OPTIMUS

5 MINUTOS	INTENSIDADE			
	PM 0401003	PM 0401004	PM 0401006	PM 0402001
27/05/15 22:55	396	240	720	576
27/05/15 23:00	228	156	696	288
27/05/15 23:05	336	252	600	396
27/05/15 23:10	276	144	600	348
27/05/15 23:15	192	180	576	204
27/05/15 23:20	312	120	480	264
27/05/15 23:25	204	144	480	228
27/05/15 23:30	216	156	456	360
27/05/15 23:35	180	120	360	216
27/05/15 23:40	264	168	360	360
27/05/15 23:45	204	72	336	156
27/05/15 23:50	276	132	240	324
27/05/15 23:55	204	132	240	204
28/05/15 00:00	420	132	216	360
28/05/15 00:05	252	132	120	324
28/05/15 00:10	324	84	120	300
28/05/15 00:15	276	180	120	492
28/05/15 00:20	300	168	120	312
28/05/15 00:25	276	120	120	348
28/05/15 00:30	276	228	120	384
28/05/15 00:35	240	180	120	360
28/05/15 00:40	264	144	120	312
28/05/15 00:45	396	168	120	408
28/05/15 00:50	276	204	120	360
28/05/15 00:55	264	96	120	276
28/05/15 01:00	180	132	120	240
28/05/15 01:05	120	96	120	156
28/05/15 01:10	228	132	120	264
28/05/15 01:15	132	144	120	240
28/05/15 01:20	156	72	120	156
28/05/15 01:25	96	60	120	120
28/05/15 01:30	108	36	96	144
28/05/15 01:35	96	84	0	132
28/05/15 01:40	24	12	0	72
28/05/15 01:45	60	96	0	156
28/05/15 01:50	84	48	0	96
28/05/15 01:55	84	36	0	96
28/05/15 02:00	84	48	0	96
28/05/15 02:05	0	24	0	24
28/05/15 02:10	72	36	0	84
28/05/15 02:15	24	60	0	72
28/05/15 02:20	60	24	0	60
28/05/15 02:25	48	24	0	60
28/05/15 02:30	24	36	0	60
28/05/15 02:35	36	24	0	48
28/05/15 02:40	36	0	0	36
28/05/15 02:45	36	12	0	24
28/05/15 02:50	48	60	0	36
28/05/15 02:55	24	48	0	36
28/05/15 03:00	48	72	0	48
28/05/15 03:05	24	48	0	72
28/05/15 03:10	60	24	0	48
28/05/15 03:15	36	48	0	72
28/05/15 03:20	24	36	0	24
28/05/15 03:25	0	24	0	24
28/05/15 03:30	48	60	0	60
28/05/15 03:35	24	12	0	60
28/05/15 03:40	72	0	0	24

Sistema de Controle de Tráfego Urbano OPTIMUS

5 MINUTOS	INTENSIDADE			
	PM 0401003	PM 0401004	PM 0401006	PM 0402001
28/05/15 03:45	24	60	0	36
28/05/15 03:50	60	36	0	24
28/05/15 03:55	12	36	0	36
28/05/15 04:00	36	24	0	36
28/05/15 04:05	36	24	0	60
28/05/15 04:10	48	60	0	84
28/05/15 04:15	24	48	0	72
28/05/15 04:20	48	36	0	60
28/05/15 04:25	12	12	0	24
28/05/15 04:30	36	48	0	60
28/05/15 04:35	48	60	0	84
28/05/15 04:40	36	24	0	48
28/05/15 04:45	72	84	0	144
28/05/15 04:50	60	108	0	132
28/05/15 04:55	72	36	0	120
28/05/15 05:00	108	60	0	156
28/05/15 05:05	120	24	0	108
28/05/15 05:10	72	60	0	72
28/05/15 05:15	120	96	0	168
28/05/15 05:20	108	132	0	192
28/05/15 05:25	72	156	0	156
28/05/15 05:30	84	144	0	204
28/05/15 05:35	180	156	0	216
28/05/15 05:40	192	216	0	348
28/05/15 05:45	120	240	0	312
28/05/15 05:50	324	300	0	492
28/05/15 05:55	540	276	0	732
28/05/15 06:00	396	300	0	576
28/05/15 06:05	456	288	0	624
28/05/15 06:10	420	336	0	612
28/05/15 06:15	588	468	24	876
28/05/15 06:20	648	408	120	924
28/05/15 06:25	960	552	120	1188
28/05/15 06:30	1164	684	120	1656
28/05/15 06:35	1188	780	120	1584
28/05/15 06:40	1512	660	120	1956
28/05/15 06:45	1572	780	144	2004
28/05/15 06:50	1704	696	240	2184
28/05/15 06:55	1656	636	240	2052
28/05/15 07:00	1716	696	288	2292
28/05/15 07:05	1560	636	480	1812
28/05/15 07:10	1824	792	480	2400
28/05/15 07:15	1632	672	504	2100
28/05/15 07:20	1668	648	600	2172
28/05/15 07:25	1452	612	600	1788
28/05/15 07:30	1608	792	600	2256
28/05/15 07:35	1512	540	600	2028
28/05/15 07:40	1512	648	600	2220
28/05/15 07:45	1752	684	564	2124
28/05/15 07:50	1572	744	612	1992
28/05/15 07:55	1584	504	600	2004
28/05/15 08:00	1488	720	600	2064
28/05/15 08:05	1380	864	600	1944
28/05/15 08:10	1428	588	600	1884
28/05/15 08:15	1440	612	624	1608
28/05/15 08:20	1176	816	720	1884
28/05/15 08:25	1332	696	720	1764
28/05/15 08:30	1380	648	696	1956

Sistema de Controle de Tráfego Urbano OPTIMUS

5 MINUTOS	INTENSIDADE			
	PM 0401003	PM 0401004	PM 0401006	PM 0402001
28/05/15 08:35	1296	636	600	1776
28/05/15 08:40	1416	648	600	1692
28/05/15 08:45	1500	588	600	1956
28/05/15 08:50	1548	804	600	2016
28/05/15 08:55	1584	636	600	1944
28/05/15 09:00	1560	612	624	1812
28/05/15 09:05	1140	804	720	1848
28/05/15 09:10	1008	696	720	1476
28/05/15 09:15	1332	696	744	1884
28/05/15 09:20	1152	756	840	1476
28/05/15 09:25	1164	696	840	1584
28/05/15 09:30	1176	648	840	1668
28/05/15 09:35	1248	672	840	1464
28/05/15 09:40	1128	696	840	1740
28/05/15 09:45	1344	672	840	1548
28/05/15 09:50	1020	660	840	1644
28/05/15 09:55	1224	744	840	1788
28/05/15 10:00	1164	612	840	1536
28/05/15 10:05	1116	684	840	1608
28/05/15 10:10	1056	720	840	1536
28/05/15 10:15	948	876	840	1320
28/05/15 10:20	1188	636	840	1572
28/05/15 10:25	1164	684	840	1416
28/05/15 10:30	1128	696	840	1752
28/05/15 10:35	1356	636	840	1608
28/05/15 10:40	1188	636	840	1716
28/05/15 10:45	1104	516	840	1368
28/05/15 10:50	1236	804	840	1680
28/05/15 10:55	1008	672	840	1368
28/05/15 11:00	1152	612	864	1488
28/05/15 11:05	1056	708	960	1464
28/05/15 11:10	936	540	960	1200
28/05/15 11:15	1032	588	960	1260
28/05/15 11:20	1200	600	960	1356
28/05/15 11:25	1032	720	960	1188
28/05/15 11:30	1092	576	960	1416
28/05/15 11:35	1152	864	960	1488
28/05/15 11:40	1128	792	960	1584
28/05/15 11:45	1080	780	984	1440
28/05/15 11:50	1380	636	1080	1668
28/05/15 11:55	1476	600	1080	1452
28/05/15 12:00	1128	720	1080	1644
28/05/15 12:05	1320	516	1080	1332
28/05/15 12:10	1092	624	1080	1368
28/05/15 12:15	1020	768	1080	1404
28/05/15 12:20	948	600	1080	1260
28/05/15 12:25	948	744	1080	1332
28/05/15 12:30	1212	564	1104	1572
28/05/15 12:35	1188	732	1200	1584
28/05/15 12:40	1356	816	1200	1512
28/05/15 12:45	1296	768	1176	1608
28/05/15 12:50	984	780	1080	1572
28/05/15 12:55	1260	732	1080	1668
28/05/15 13:00	1548	708	1056	1956
28/05/15 13:05	1560	648	960	1884
28/05/15 13:10	1404	636	960	1800
28/05/15 13:15	1368	612	984	1704
28/05/15 13:20	1440	792	1080	1908

Sistema de Controle de Tráfego Urbano OPTIMUS

5 MINUTOS	INTENSIDADE			
	PM 0401003	PM 0401004	PM 0401006	PM 0402001
28/05/15 13:25	1284	780	1080	1656
28/05/15 13:30	1416	492	1104	1824
28/05/15 13:35	1224	828	1200	1560
28/05/15 13:40	1440	648	1200	1848
28/05/15 13:45	1296	864	1176	1824
28/05/15 13:50	1176	744	1080	1500
28/05/15 13:55	1164	792	1080	1740
28/05/15 14:00	1224	612	1056	1560
28/05/15 14:05	1236	840	960	1656
28/05/15 14:10	1236	732	960	1848
28/05/15 14:15	1428	672	960	1920
28/05/15 14:20	1356	600	960	1596
28/05/15 14:25	1308	684	960	1788
28/05/15 14:30	1188	564	960	1572
28/05/15 14:35	1296	684	960	1680
28/05/15 14:40	1308	756	960	1560
28/05/15 14:45	1044	876	960	1572
28/05/15 14:50	1308	564	960	1572
28/05/15 14:55	1212	600	960	1632
28/05/15 15:00	1212	612	960	1464
28/05/15 15:05	1188	684	960	1500
28/05/15 15:10	1200	756	960	1584
28/05/15 15:15	1284	708	960	1620
28/05/15 15:20	1356	636	960	1824
28/05/15 15:25	1320	696	960	1536
28/05/15 15:30	1176	636	960	1680
28/05/15 15:35	1140	780	960	1728
28/05/15 15:40	1068	648	960	1548
28/05/15 15:45	1152	624	984	1296
28/05/15 15:50	1224	672	1080	1452
28/05/15 15:55	1176	720	1080	1308
28/05/15 16:00	1032	720	1080	1548
28/05/15 16:05	1056	672	1080	1488
28/05/15 16:10	1176	804	1080	1524
28/05/15 16:15	1164	624	1080	1656
28/05/15 16:20	840	636	1080	1284
28/05/15 16:25	1068	696	1080	1344
28/05/15 16:30	900	636	1104	1164
28/05/15 16:35	1116	624	1200	1500
28/05/15 16:40	1056	552	1200	1212
28/05/15 16:45	1176	492	1224	1284
28/05/15 16:50	1068	672	1320	1452
28/05/15 16:55	1176	816	1320	1560
28/05/15 17:00	1140	696	1344	1368
28/05/15 17:05	960	576	1440	1236
28/05/15 17:10	1104	564	1440	1284
28/05/15 17:15	1236	744	1416	1644
28/05/15 17:20	1104	708	1320	1524
28/05/15 17:25	1452	852	1320	1656
28/05/15 17:30	1296	672	1320	1452
28/05/15 17:35	1236	540	1320	1452
28/05/15 17:40	1332	600	1320	1776
28/05/15 17:45	948	912	1344	1440
28/05/15 17:50	1188	576	1440	1608
28/05/15 17:55	1344	768	1440	1548
28/05/15 18:00	1044	648	1440	1296
28/05/15 18:05	1356	624	1440	1572
28/05/15 18:10	1032	528	1440	1272

Sistema de Controle de Tráfego Urbano OPTIMUS

5 MINUTOS	INTENSIDADE			
	PM 0401003	PM 0401004	PM 0401006	PM 0402001
28/05/15 18:15	1080	252	1368	1188
28/05/15 18:20	1224	624	1080	1368
28/05/15 18:25	900	756	1080	1296
28/05/15 18:30	1224	708	1104	1728
28/05/15 18:35	936	660	1200	1332
28/05/15 18:40	1284	660	1200	1380
28/05/15 18:45	1308	732	1224	1896
28/05/15 18:50	1128	492	1320	1260
28/05/15 18:55	1056	420	1320	1296
28/05/15 19:00	1248	396	1344	1212
28/05/15 19:05	1212	576	1440	1392
28/05/15 19:10	1380	600	1440	1548
28/05/15 19:15	1212	624	1464	1656
28/05/15 19:20	1068	492	1560	1464
28/05/15 19:25	792	624	1560	1224
28/05/15 19:30	1176	384	1560	1200
28/05/15 19:35	960	636	1560	1344
28/05/15 19:40	1188	636	1560	1404
28/05/15 19:45	1068	684	1536	1440
28/05/15 19:50	1236	576	1440	1572
28/05/15 19:55	1020	636	1440	1296
28/05/15 20:00	1008	552	1392	1200
28/05/15 20:05	708	480	1200	888
28/05/15 20:10	804	528	1200	1056
28/05/15 20:15	792	504	1176	1044
28/05/15 20:20	960	564	1080	1332
28/05/15 20:25	732	552	1080	996
28/05/15 20:30	792	432	1056	900
28/05/15 20:35	1188	492	960	1308
28/05/15 20:40	780	408	960	1068
28/05/15 20:45	852	480	936	1152
28/05/15 20:50	828	420	840	960
28/05/15 20:55	804	420	840	1020
28/05/15 21:00	924	576	840	1152
28/05/15 21:05	708	432	840	948
28/05/15 21:10	576	384	840	720
28/05/15 21:15	816	540	840	948
28/05/15 21:20	804	480	840	1056
28/05/15 21:25	756	396	840	1068
28/05/15 21:30	612	444	840	852
28/05/15 21:35	756	360	840	972
28/05/15 21:40	756	360	840	948
28/05/15 21:45	636	588	840	900
28/05/15 21:50	600	360	840	912
28/05/15 21:55	672	372	840	876
28/05/15 22:00	612	384	816	792
28/05/15 22:05	672	540	720	936
28/05/15 22:10	732	504	720	1032
28/05/15 22:15	720	576	696	1176
28/05/15 22:20	792	408	600	1236
28/05/15 22:25	720	492	600	1080
28/05/15 22:30	600	396	624	888
28/05/15 22:35	864	468	720	1104
28/05/15 22:40	552	324	720	816
28/05/15 22:45	672	324	720	876
28/05/15 22:50	756	324	720	768
28/05/15 22:55	492	204	720	648
28/05/15 23:00	504	168	696	480

Sistema de Controle de Tráfego Urbano OPTIMUS

5 MINUTOS	INTENSIDADE			
	PM 0401003	PM 0401004	PM 0401006	PM 0402001
28/05/15 23:05	468	276	600	720
28/05/15 23:10	300	264	600	420
28/05/15 23:15	528	348	576	612
28/05/15 23:20	276	240	480	444
28/05/15 23:25	360	312	480	552
28/05/15 23:30	240	288	456	372
28/05/15 23:35	216	156	360	288
28/05/15 23:40	156	84	360	204
28/05/15 23:45	228	120	336	312
28/05/15 23:50	276	252	240	372
28/05/15 23:55	264	120	240	336
29/05/15 00:00	276	168	240	276
29/05/15 00:05	156	120	240	228
29/05/15 00:10	180	108	240	252
29/05/15 00:15	180	36	240	180
29/05/15 00:20	180	84	240	144
29/05/15 00:25	192	156	240	216
29/05/15 00:30	180	96	240	240
29/05/15 00:35	228	96	240	276
29/05/15 00:40	144	156	240	204
29/05/15 00:45	72	84	240	120
29/05/15 00:50	156	60	240	180
29/05/15 00:55	96	60	240	156
29/05/15 01:00	120	132	216	192
29/05/15 01:05	108	36	120	108
29/05/15 01:10	72	36	120	60
29/05/15 01:15	60	60	120	72
29/05/15 01:20	84	48	120	60
29/05/15 01:25	96	84	120	144
29/05/15 01:30	96	36	120	84
29/05/15 01:35	108	72	120	120
29/05/15 01:40	60	48	120	84
29/05/15 01:45	36	48	96	48
29/05/15 01:50	36	108	0	144
29/05/15 01:55	48	12	0	12
29/05/15 02:00	108	60	0	108
29/05/15 02:05	48	36	0	48
29/05/15 02:10	60	24	0	84
29/05/15 02:15	96	60	0	72
29/05/15 02:20	84	48	0	84
29/05/15 02:25	48	48	0	60
29/05/15 02:30	72	24	0	60
29/05/15 02:35	24	48	0	60
29/05/15 02:40	48	0	0	24
29/05/15 02:45	36	36	0	48
29/05/15 02:50	36	12	0	48
29/05/15 02:55	60	24	0	60
29/05/15 03:00	36	12	0	24
29/05/15 03:05	24	0	0	36
29/05/15 03:10	48	36	0	60
29/05/15 03:15	72	24	0	84
29/05/15 03:20	12	12	0	36
29/05/15 03:25	24	12	0	24
29/05/15 03:30	24	84	0	72
29/05/15 03:35	48	24	0	48
29/05/15 03:40	72	24	0	12
29/05/15 03:45	84	60	0	120
29/05/15 03:50	12	36	0	60

Sistema de Controle de Tráfego Urbano OPTIMUS

5 MINUTOS	INTENSIDADE			
	PM 0401003	PM 0401004	PM 0401006	PM 0402001
29/05/15 03:55	0	36	0	24
29/05/15 04:00	24	48	0	60
29/05/15 04:05	12	24	0	24
29/05/15 04:10	36	48	0	48
29/05/15 04:15	36	48	0	60
29/05/15 04:20	36	24	0	60
29/05/15 04:25	48	36	0	48
29/05/15 04:30	60	48	0	60
29/05/15 04:35	72	72	0	120
29/05/15 04:40	48	24	0	72
29/05/15 04:45	48	24	0	36
29/05/15 04:50	72	72	0	108
29/05/15 04:55	36	60	0	72
29/05/15 05:00	96	84	0	120
29/05/15 05:05	12	24	0	36
29/05/15 05:10	48	96	0	120
29/05/15 05:15	120	132	0	168
29/05/15 05:20	84	108	0	156
29/05/15 05:25	132	108	0	216
29/05/15 05:30	192	120	0	252
29/05/15 05:35	144	216	0	324
29/05/15 05:40	240	276	0	420
29/05/15 05:45	276	264	0	540
29/05/15 05:50	228	252	0	384
29/05/15 05:55	504	216	0	612
29/05/15 06:00	456	396	0	696
29/05/15 06:05	408	324	0	672
29/05/15 06:10	492	396	0	696
29/05/15 06:15	468	468	24	780
29/05/15 06:20	480	444	120	804
29/05/15 06:25	1044	600	120	1368
29/05/15 06:30	1248	732	120	1644
29/05/15 06:35	924	804	120	1500
29/05/15 06:40	1104	864	120	1500
29/05/15 06:45	1416	828	168	2040
29/05/15 06:50	1608	756	360	2136
29/05/15 06:55	1512	672	360	1944
29/05/15 07:00	1788	624	384	2316
29/05/15 07:05	1560	780	480	1980
29/05/15 07:10	1716	576	480	2220
29/05/15 07:15	1584	804	504	2016
29/05/15 07:20	1704	588	600	1992
29/05/15 07:25	1596	528	600	2052
29/05/15 07:30	1572	696	600	2004
29/05/15 07:35	1596	468	600	1824
29/05/15 07:40	1248	744	600	1752
29/05/15 07:45	1452	456	600	1488
29/05/15 07:50	1092	624	600	1416
29/05/15 07:55	1440	516	600	1776
29/05/15 08:00	1296	696	600	1416
29/05/15 08:05	1368	504	600	1680
29/05/15 08:10	1200	660	600	1512
29/05/15 08:15	1296	552	624	1464
29/05/15 08:20	1320	444	720	1548
29/05/15 08:25	1464	420	720	1788
29/05/15 08:30	1332	360	720	1476
29/05/15 08:35	1380	720	1152	1752
29/05/15 08:40	1524	528	828	1776

Sistema de Controle de Tráfego Urbano OPTIMUS

5 MINUTOS	INTENSIDADE			
	PM 0401003	PM 0401004	PM 0401006	PM 0402001
29/05/15 08:45	1536	660	1080	2172
29/05/15 08:50	1560	564	708	1824
29/05/15 08:55	1392	840	1044	1896
29/05/15 09:00	1284	768	756	1872
29/05/15 09:05	1464	600	1008	1968
29/05/15 09:10	1116	612	660	1344
29/05/15 09:15	1104	576	1008	1572
29/05/15 09:20	1068	612	684	1380
29/05/15 09:25	1260	828	1080	1848
29/05/15 09:30	1212	684	672	1728
29/05/15 09:35	1584	744	1032	2052
29/05/15 09:40	1620	540	900	1788
29/05/15 09:45	1488	636	1044	1908
29/05/15 09:50	1512	600	684	1872
29/05/15 09:55	1524	708	1020	1920
29/05/15 10:00	1692	720	780	2076
29/05/15 10:05	1512	720	1152	2040
29/05/15 10:10	1464	600	744	1764
29/05/15 10:15	1404	768	1032	1800
29/05/15 10:20	1452	780	1080	1920
29/05/15 10:25	1212	924	1020	1800
29/05/15 10:30	1464	744	972	1848
29/05/15 10:35	1380	756	768	1992
29/05/15 10:40	1224	1020	840	1824
29/05/15 10:45	1284	744	840	1896
29/05/15 10:50	1164	816	840	1668
29/05/15 10:55	1164	792	840	1620
29/05/15 11:00	1116	816	840	1548
29/05/15 11:05	1248	576	840	1644
29/05/15 11:10	1176	696	840	1380
29/05/15 11:15	1224	564	888	1344
29/05/15 11:20	1344	828	1080	1716
29/05/15 11:25	1284	780	1080	1680
29/05/15 11:30	1308	672	1080	1704
29/05/15 11:35	1128	672	1080	1500
29/05/15 11:40	1044	912	1080	1536
29/05/15 11:45	1200	744	1104	1464
29/05/15 11:50	1320	660	1200	1440
29/05/15 11:55	1452	708	1200	1620
29/05/15 12:00	1644	660	1224	1860
29/05/15 12:05	1476	720	1320	1800
29/05/15 12:10	1344	768	1320	1788
29/05/15 12:15	1464	720	1296	1848
29/05/15 12:20	1188	936	1200	1644
29/05/15 12:25	1344	792	1200	1788
29/05/15 12:30	1044	696	1200	1536
29/05/15 12:35	1368	708	1200	1488
29/05/15 12:40	1332	660	1200	1608
29/05/15 12:45	1416	612	1200	1920
29/05/15 12:50	1284	804	1200	1620
29/05/15 12:55	1428	600	1200	1632
29/05/15 13:00	1284	888	1200	1884
29/05/15 13:05	1824	540	1200	1884
29/05/15 13:10	1512	768	1200	1836
29/05/15 13:15	1668	540	1200	1668
29/05/15 13:20	1668	600	1200	2016
29/05/15 13:25	1356	684	1200	1560
29/05/15 13:30	1320	888	1200	1956

Sistema de Controle de Tráfego Urbano OPTIMUS

5 MINUTOS	INTENSIDADE			
	PM 0401003	PM 0401004	PM 0401006	PM 0402001
29/05/15 13:35	1128	660	1200	1524
29/05/15 13:40	1152	1008	1200	1680
29/05/15 13:45	1092	852	1176	1560
29/05/15 13:50	1644	672	1080	1992
29/05/15 13:55	1392	672	1080	1668
29/05/15 14:00	1488	732	1080	1956
29/05/15 14:05	1080	780	1080	1656
29/05/15 14:10	1212	756	1080	1440
29/05/15 14:15	1524	624	1056	1668
29/05/15 14:20	1224	696	960	1740
29/05/15 14:25	960	984	960	1572
29/05/15 14:30	1272	648	1008	1536
29/05/15 14:35	1416	732	1200	1752
29/05/15 14:40	1236	612	1200	1692
29/05/15 14:45	1164	732	1200	1608
29/05/15 14:50	1068	852	1200	1452
29/05/15 14:55	1248	816	1200	1572
29/05/15 15:00	1236	720	1200	1716
29/05/15 15:05	1080	852	1200	1512
29/05/15 15:10	1296	672	1200	1560
29/05/15 15:15	1572	660	1200	1800
29/05/15 15:20	1284	684	1200	1848
29/05/15 15:25	1500	576	1200	1668
29/05/15 15:30	1188	648	1224	1584
29/05/15 15:35	1056	660	1320	1380
29/05/15 15:40	1140	684	1320	1524
29/05/15 15:45	1032	648	1320	1284
29/05/15 15:50	1056	840	1320	1260
29/05/15 15:55	1284	672	1320	1668
29/05/15 16:00	1332	744	1320	1644
29/05/15 16:05	1176	936	1320	1752
29/05/15 16:10	1128	792	1320	1476
29/05/15 16:15	1092	744	1296	1488
29/05/15 16:20	936	828	1200	1668
29/05/15 16:25	1416	720	1200	1632
29/05/15 16:30	1308	624	1248	1512
29/05/15 16:35	1164	756	1440	1500
29/05/15 16:40	1152	636	1440	1284
29/05/15 16:45	1140	780	1440	1572
29/05/15 16:50	1284	732	1440	1620
29/05/15 16:55	1356	564	1440	1704
29/05/15 17:00	1020	660	1464	1080
29/05/15 17:05	492	348	1560	636
29/05/15 17:10	780	552	1560	852
29/05/15 17:15	912	480	1560	1164
29/05/15 17:20	696	612	1560	864
29/05/15 17:25	456	228	1560	456
29/05/15 17:30	1092	444	1560	1260
29/05/15 17:35	1140	396	1560	1140
29/05/15 17:40	756	576	1560	648
29/05/15 17:45	648	228	1512	528
29/05/15 17:50	336	180	1320	384
29/05/15 17:55	780	144	1320	516
29/05/15 18:00	672	396	1296	612
29/05/15 18:05	612	180	1200	696
29/05/15 18:10	1260	564	1200	1500
29/05/15 18:15	1140	408	1248	1332
29/05/15 18:20	648	312	1440	504

Sistema de Controle de Tráfego Urbano OPTIMUS

5 MINUTOS	INTENSIDADE			
	PM 0401003	PM 0401004	PM 0401006	PM 0402001
29/05/15 18:25	540	192	1440	648
29/05/15 18:30	540	396	1440	552
29/05/15 18:35	348	168	1440	420
29/05/15 18:40	876	492	1440	1140
29/05/15 18:45	1380	480	1440	1548
29/05/15 18:50	1260	396	1440	1164
29/05/15 18:55	672	288	1440	696
29/05/15 19:00	888	456	1440	948
29/05/15 19:05	600	240	1440	780
29/05/15 19:10	732	312	1440	336
29/05/15 19:15	516	264	1416	624
29/05/15 19:20	1524	240	1320	1440
29/05/15 19:25	1332	660	1320	1752
29/05/15 19:30	1152	384	1296	1248
29/05/15 19:35	948	708	1200	1200
29/05/15 19:40	840	936	1200	1332
29/05/15 19:45	1020	660	1200	1344
29/05/15 19:50	1788	588	1200	1920
29/05/15 19:55	1476	756	1200	2028
29/05/15 20:00	1428	636	1176	1728
29/05/15 20:05	1284	636	1080	1692
29/05/15 20:10	1116	612	1080	1164
29/05/15 20:15	900	900	1056	1596
29/05/15 20:20	1212	732	960	1560
29/05/15 20:25	1044	780	960	1368
29/05/15 20:30	1212	696	960	1512
29/05/15 20:35	1020	612	960	1560
29/05/15 20:40	828	552	960	1296
29/05/15 20:45	960	660	936	1308
29/05/15 20:50	900	708	840	1284
29/05/15 20:55	1104	576	840	1416
29/05/15 21:00	984	624	840	1344
29/05/15 21:05	1104	528	840	1308
29/05/15 21:10	732	552	840	1080
29/05/15 21:15	924	612	864	1284
29/05/15 21:20	756	492	960	1176
29/05/15 21:25	804	552	960	1080
29/05/15 21:30	852	528	936	1080
29/05/15 21:35	624	324	840	780
29/05/15 21:40	768	540	840	1068
29/05/15 21:45	972	420	816	1188
29/05/15 21:50	780	432	720	1224
29/05/15 21:55	840	624	720	1272
29/05/15 22:00	672	528	720	1020
29/05/15 22:05	768	636	720	1068
29/05/15 22:10	828	408	720	1092
29/05/15 22:15	624	672	720	996
29/05/15 22:20	948	528	720	1260
29/05/15 22:25	636	588	720	960
29/05/15 22:30	732	444	744	924
29/05/15 22:35	684	384	840	960
29/05/15 22:40	600	336	840	936
29/05/15 22:45	540	444	840	828
29/05/15 22:50	732	276	840	792
29/05/15 22:55	576	360	840	780
29/05/15 23:00	384	444	816	588
29/05/15 23:05	564	372	720	828
29/05/15 23:10	504	408	720	732

Sistema de Controle de Tráfego Urbano OPTIMUS

5 MINUTOS	INTENSIDADE			
	PM 0401003	PM 0401004	PM 0401006	PM 0402001
29/05/15 23:15	552	288	696	792
29/05/15 23:20	360	324	600	372
29/05/15 23:25	384	180	600	420
29/05/15 23:30	324	264	576	612
29/05/15 23:35	420	384	480	660
29/05/15 23:40	300	312	480	504
29/05/15 23:45	384	264	480	516
29/05/15 23:50	420	276	480	516
29/05/15 23:55	348	192	480	468
30/05/15 00:00	420	240	480	504
30/05/15 00:05	408	324	480	636
30/05/15 00:10	336	264	480	468
30/05/15 00:15	288	228	480	432
30/05/15 00:20	432	204	480	420
30/05/15 00:25	264	300	480	420
30/05/15 00:30	336	240	456	408
30/05/15 00:35	300	204	360	396
30/05/15 00:40	420	252	360	528
30/05/15 00:45	312	180	336	372
30/05/15 00:50	240	240	240	408
30/05/15 00:55	276	204	240	372
30/05/15 01:00	156	120	264	180
30/05/15 01:05	204	180	360	324
30/05/15 01:10	204	180	360	312
30/05/15 01:15	192	132	336	216
30/05/15 01:20	312	108	240	276
30/05/15 01:25	180	180	240	300
30/05/15 01:30	192	216	240	276
30/05/15 01:35	180	132	240	204
30/05/15 01:40	168	96	240	252
30/05/15 01:45	192	132	240	264
30/05/15 01:50	240	108	240	348
30/05/15 01:55	132	108	240	180
30/05/15 02:00	156	84	240	204
30/05/15 02:05	120	108	240	216
30/05/15 02:10	84	144	240	228
30/05/15 02:15	120	156	216	180
30/05/15 02:20	132	96	120	192
30/05/15 02:25	24	84	120	108
30/05/15 02:30	108	96	120	156
30/05/15 02:35	132	108	120	120
30/05/15 02:40	84	72	120	156
30/05/15 02:45	144	72	120	120
30/05/15 02:50	132	132	120	204
30/05/15 02:55	60	144	120	168
30/05/15 03:00	132	48	120	144
30/05/15 03:05	156	48	120	108
30/05/15 03:10	120	24	120	72
30/05/15 03:15	84	72	120	132
30/05/15 03:20	180	60	120	132
30/05/15 03:25	120	48	120	96
30/05/15 03:30	108	108	120	108
30/05/15 03:35	72	48	120	72
30/05/15 03:40	108	96	120	156
30/05/15 03:45	72	48	120	72
30/05/15 03:50	120	72	120	120
30/05/15 03:55	156	72	120	180
30/05/15 04:00	48	96	120	120

Sistema de Controle de Tráfego Urbano OPTIMUS

5 MINUTOS	INTENSIDADE			
	PM 0401003	PM 0401004	PM 0401006	PM 0402001
30/05/15 04:05	96	96	120	144
30/05/15 04:10	144	36	120	132
30/05/15 04:15	108	84	96	108
30/05/15 04:20	120	60	0	108
30/05/15 04:25	48	36	0	24
30/05/15 04:30	48	72	24	84
30/05/15 04:35	48	84	120	48
30/05/15 04:40	132	60	120	132
30/05/15 04:45	132	84	120	132
30/05/15 04:50	36	156	120	96
30/05/15 04:55	84	60	120	84
30/05/15 05:00	36	120	120	84
30/05/15 05:05	60	96	120	156
30/05/15 05:10	120	84	120	144
30/05/15 05:15	84	108	96	120
30/05/15 05:20	144	96	0	180
30/05/15 05:25	60	84	0	96
30/05/15 05:30	60	108	0	156
30/05/15 05:35	204	132	0	252
30/05/15 05:40	192	204	0	300
30/05/15 05:45	216	156	24	360
30/05/15 05:50	120	180	120	288
30/05/15 05:55	168	156	120	204
30/05/15 06:00	96	180	120	204
30/05/15 06:05	168	228	120	324
30/05/15 06:10	264	180	120	420
30/05/15 06:15	264	204	120	384
30/05/15 06:20	264	240	120	444
30/05/15 06:25	276	192	120	324
30/05/15 06:30	204	324	120	420
30/05/15 06:35	492	300	120	516
30/05/15 06:40	408	228	120	708
30/05/15 06:45	456	300	120	708
30/05/15 06:50	396	600	120	744
30/05/15 06:55	528	336	120	840
30/05/15 07:00	420	432	144	708
30/05/15 07:05	504	348	240	588
30/05/15 07:10	516	360	240	840
30/05/15 07:15	540	384	240	684
30/05/15 07:20	492	540	240	852
30/05/15 07:25	468	504	240	816
30/05/15 07:30	648	420	264	936
30/05/15 07:35	588	408	360	672
30/05/15 07:40	804	432	360	1284
30/05/15 07:45	756	684	360	984
30/05/15 07:50	804	648	360	1272
30/05/15 07:55	1068	660	360	1428
30/05/15 08:00	972	576	360	1188
30/05/15 08:05	792	576	360	1080
30/05/15 08:10	840	660	360	1332
30/05/15 08:15	996	708	384	1356
30/05/15 08:20	888	660	480	1440
30/05/15 08:25	1020	744	480	1344
30/05/15 08:30	828	672	480	1296
30/05/15 08:35	780	744	480	1296
30/05/15 08:40	1056	720	480	1392
30/05/15 08:45	1056	804	504	1440
30/05/15 08:50	1200	684	600	1656

Sistema de Controle de Tráfego Urbano OPTIMUS

5 MINUTOS	INTENSIDADE			
	PM 0401003	PM 0401004	PM 0401006	PM 0402001
30/05/15 08:55	1104	672	600	1296
30/05/15 09:00	1128	576	624	1512
30/05/15 09:05	1080	660	720	1428
30/05/15 09:10	1116	648	720	1512
30/05/15 09:15	996	684	720	1212
30/05/15 09:20	1236	660	720	1644
30/05/15 09:25	1296	624	720	1392
30/05/15 09:30	1164	588	720	1620
30/05/15 09:35	1068	684	720	1488
30/05/15 09:40	1176	624	720	1404
30/05/15 09:45	1128	552	720	1284
30/05/15 09:50	1140	648	720	1692
30/05/15 09:55	1164	672	720	1344
30/05/15 10:00	1152	612	744	1656
30/05/15 10:05	1164	708	840	1248
30/05/15 10:10	1356	564	840	1464
30/05/15 10:15	1032	504	840	1056
30/05/15 10:20	1092	576	840	1332
30/05/15 10:25	1140	552	840	1164
30/05/15 10:30	1176	600	840	1536
30/05/15 10:35	1152	600	840	1260
30/05/15 10:40	1128	696	840	1572
30/05/15 10:45	1116	672	840	1344
30/05/15 10:50	1260	564	840	1836
30/05/15 10:55	1284	624	840	1392
30/05/15 11:00	1068	600	864	1416
30/05/15 11:05	1272	732	960	1572
30/05/15 11:10	1224	696	960	1476
30/05/15 11:15	1272	672	960	1392
30/05/15 11:20	1188	588	960	1464
30/05/15 11:25	1248	648	960	1284
30/05/15 11:30	1308	588	1008	1740
30/05/15 11:35	1212	696	1200	1296
30/05/15 11:40	1272	636	1200	1440
30/05/15 11:45	1248	636	1128	1356
30/05/15 11:50	1332	708	840	1656
30/05/15 11:55	1212	744	840	1488
30/05/15 12:00	1188	576	888	1380
30/05/15 12:05	1248	816	1080	1608
30/05/15 12:10	1332	636	1080	1584
30/05/15 12:15	1404	636	1080	1368
30/05/15 12:20	1476	432	1080	1644
30/05/15 12:25	1404	660	1080	1584
30/05/15 12:45	648	468	624	876
30/05/15 12:50	1596	612	960	1644
30/05/15 12:55	1080	840	960	1440
30/05/15 13:00	1548	612	960	1704
30/05/15 13:05	1308	876	960	1752
30/05/15 13:10	1428	648	960	1620
30/05/15 13:15	1344	876	960	1656
30/05/15 13:20	1560	516	960	1512
30/05/15 13:25	1236	864	960	1596
30/05/15 13:30	1404	648	960	1620
30/05/15 13:35	1032	828	960	1428
30/05/15 13:40	1152	468	960	1560
30/05/15 13:45	1248	828	984	1464
30/05/15 13:50	1488	612	1080	1644
30/05/15 13:55	1308	648	1080	1716

Sistema de Controle de Tráfego Urbano OPTIMUS

5 MINUTOS	INTENSIDADE			
	PM 0401003	PM 0401004	PM 0401006	PM 0402001
30/05/15 14:00	1224	660	1056	1404
30/05/15 14:05	1176	792	960	1572
30/05/15 14:10	924	708	960	1092
30/05/15 14:15	1224	912	936	1680
30/05/15 14:20	1152	720	840	1644
30/05/15 14:25	996	768	840	1608
30/05/15 14:30	1176	624	816	1392
30/05/15 14:35	1080	624	720	1404
30/05/15 14:40	1008	576	720	1116
30/05/15 14:45	1020	648	720	1320
30/05/15 14:50	864	816	720	1212
30/05/15 14:55	924	720	720	1488
30/05/15 15:00	792	552	720	1152
30/05/15 15:05	1068	672	720	1416
30/05/15 15:10	1080	732	720	1416
30/05/15 15:15	972	648	744	1452
30/05/15 15:20	888	564	840	1212
30/05/15 15:25	984	612	840	1296
30/05/15 15:30	732	588	840	1080
30/05/15 15:35	708	540	840	1092
30/05/15 15:40	876	612	840	1164
30/05/15 15:45	804	624	840	1236
30/05/15 15:50	864	612	840	1188
30/05/15 15:55	948	540	840	1224
30/05/15 16:00	852	648	840	1032
30/05/15 16:05	912	600	840	1164
30/05/15 16:10	1080	636	840	1320
30/05/15 16:15	960	636	816	1128
30/05/15 16:20	744	540	720	1116
30/05/15 16:25	864	648	720	1248
30/05/15 16:30	792	600	720	1224
30/05/15 16:35	1020	600	720	1164
30/05/15 16:40	684	660	720	1128
30/05/15 16:45	948	708	720	1176
30/05/15 16:50	1056	720	720	1512
30/05/15 16:55	864	516	720	1152
30/05/15 17:00	996	648	744	1368
30/05/15 17:05	912	672	840	1296
30/05/15 17:10	864	756	840	1296
30/05/15 17:15	912	564	816	1116
30/05/15 17:20	792	552	720	876
30/05/15 17:25	1032	564	720	1380
30/05/15 17:30	804	552	720	1008
30/05/15 17:35	852	696	720	1248
30/05/15 17:40	1080	600	720	1236
30/05/15 17:45	936	444	744	1236
30/05/15 17:50	756	600	840	1068
30/05/15 17:55	1020	420	840	1104
30/05/15 18:00	924	588	864	1212
30/05/15 18:05	816	588	960	948
30/05/15 18:10	780	744	960	1068
30/05/15 18:15	1092	696	924	1368
30/05/15 18:20	1104	696	744	1440
30/05/15 18:25	900	612	840	1488
30/05/15 18:30	912	624	840	1248
30/05/15 18:35	924	696	840	1200
30/05/15 18:40	912	552	840	1464
30/05/15 18:45	912	588	864	1344

Sistema de Controle de Tráfego Urbano OPTIMUS

5 MINUTOS	INTENSIDADE			
	PM 0401003	PM 0401004	PM 0401006	PM 0402001
30/05/15 18:50	984	696	960	1188
30/05/15 18:55	1020	588	960	1356
30/05/15 19:00	1056	624	936	1116
30/05/15 19:05	900	516	840	1296
30/05/15 19:10	900	756	840	1116
30/05/15 19:15	1092	588	840	1524
30/05/15 19:20	972	540	840	1104
30/05/15 19:25	924	504	840	1200
30/05/15 19:30	756	672	864	1236
30/05/15 19:35	1176	636	960	1548
30/05/15 19:40	1020	444	960	1116
30/05/15 19:45	1152	624	936	1656
30/05/15 19:50	912	516	840	1116
30/05/15 19:55	852	744	840	1368
30/05/15 20:00	1080	756	840	1416
30/05/15 20:05	1032	432	840	1356
30/05/15 20:10	984	588	840	1308
30/05/15 20:15	984	612	816	1368
30/05/15 20:20	840	408	720	1248
30/05/15 20:25	732	648	720	984
30/05/15 20:30	924	504	720	1212
30/05/15 20:35	864	588	720	1236
30/05/15 20:40	1008	516	720	1176
30/05/15 20:45	828	480	720	1152
30/05/15 20:50	756	492	720	1056
30/05/15 20:55	684	444	720	888
30/05/15 21:00	864	540	720	1068
30/05/15 21:05	864	504	720	1092
30/05/15 21:10	648	576	720	1176
30/05/15 21:15	996	468	720	1128
30/05/15 21:20	840	540	720	1152
30/05/15 21:25	588	420	720	912
30/05/15 21:30	780	468	696	972
30/05/15 21:35	612	480	600	780
30/05/15 21:40	612	600	600	996
30/05/15 21:45	708	420	600	960
30/05/15 21:50	948	408	600	996
30/05/15 21:55	612	492	600	960
30/05/15 22:00	576	264	600	744
30/05/15 22:05	756	444	600	1068
30/05/15 22:10	636	420	600	900
30/05/15 22:15	744	504	624	1032
30/05/15 22:20	708	540	720	936
30/05/15 22:25	624	480	720	816
30/05/15 22:30	684	396	720	864
30/05/15 22:35	600	240	720	780
30/05/15 22:40	612	480	720	888
30/05/15 22:45	588	444	672	744
30/05/15 22:50	600	324	480	708
30/05/15 22:55	468	408	480	744
30/05/15 23:00	516	252	504	600
30/05/15 23:05	516	324	600	696
30/05/15 23:10	576	336	600	828
30/05/15 23:15	420	540	576	792
30/05/15 23:20	480	348	480	648
30/05/15 23:25	600	276	480	672
30/05/15 23:30	480	432	480	768
30/05/15 23:35	504	408	480	756

Sistema de Controle de Tráfego Urbano OPTIMUS

5 MINUTOS	INTENSIDADE			
	PM 0401003	PM 0401004	PM 0401006	PM 0402001
30/05/15 23:40	372	324	480	552
30/05/15 23:45	312	336	480	480
30/05/15 23:50	480	276	480	648
30/05/15 23:55	396	192	480	384
31/05/15 00:00	468	300	480	636
31/05/15 00:05	372	336	480	564
31/05/15 00:10	372	168	480	420
31/05/15 00:15	420	132	480	396
31/05/15 00:20	300	216	480	360
31/05/15 00:25	396	264	480	564
31/05/15 00:30	348	300	480	456
31/05/15 00:35	372	312	480	444
31/05/15 00:40	324	216	480	468
31/05/15 00:45	324	252	432	468
31/05/15 00:50	300	180	240	372
31/05/15 00:55	252	228	240	336
31/05/15 01:00	324	276	264	480
31/05/15 01:05	312	264	408	444
31/05/15 01:10	288	192	276	396
31/05/15 01:15	444	156	324	516
31/05/15 01:20	240	216	312	372
31/05/15 01:25	228	144	336	288
31/05/15 01:30	252	192	240	324
31/05/15 01:35	348	228	312	444
31/05/15 01:40	240	168	252	372
31/05/15 01:45	156	132	324	240
31/05/15 01:50	264	192	228	360
31/05/15 01:55	288	84	252	324
31/05/15 02:00	240	180	216	312
31/05/15 02:05	180	132	240	288
31/05/15 02:10	132	72	216	156
31/05/15 02:15	228	120	288	192
31/05/15 02:20	72	120	240	240
31/05/15 02:25	132	120	216	168
31/05/15 02:30	132	144	192	192
31/05/15 02:35	192	96	120	264
31/05/15 02:40	228	84	132	180
31/05/15 02:45	144	168	276	204
31/05/15 02:50	168	156	264	288
31/05/15 02:55	156	96	144	216
31/05/15 03:00	168	156	228	228
31/05/15 03:05	120	120	216	192
31/05/15 03:10	156	156	192	264
31/05/15 03:15	156	108	156	192
31/05/15 03:20	192	132	264	252
31/05/15 03:25	204	156	240	264
31/05/15 03:30	252	72	192	276
31/05/15 03:35	84	120	144	108
31/05/15 03:40	108	108	96	156
31/05/15 03:45	144	48	168	144
31/05/15 03:50	216	132	132	252
31/05/15 03:55	168	120	264	216
31/05/15 04:00	108	84	120	144
31/05/15 04:05	168	84	132	192
31/05/15 04:10	108	84	96	168
31/05/15 04:15	180	84	168	168
31/05/15 04:20	132	60	168	120
31/05/15 04:25	132	72	192	132

Sistema de Controle de Tráfego Urbano OPTIMUS

5 MINUTOS	INTENSIDADE			
	PM 0401003	PM 0401004	PM 0401006	PM 0402001
31/05/15 04:30	60	132	168	168
31/05/15 04:35	60	84	156	84
31/05/15 04:40	120	48	120	60
31/05/15 04:45	120	108	72	144
31/05/15 04:50	132	120	144	168
31/05/15 04:55	24	120	108	132
31/05/15 05:00	96	144	204	204
31/05/15 05:05	96	36	96	120
31/05/15 05:10	96	84	84	144
31/05/15 05:15	108	72	84	96
31/05/15 05:20	84	120	120	144
31/05/15 05:25	96	84	96	156
31/05/15 05:30	84	96	96	120
31/05/15 05:35	108	108	72	156
31/05/15 05:40	108	96	84	204
31/05/15 05:45	156	72	84	156
31/05/15 05:50	132	180	84	192
31/05/15 05:55	180	120	132	240
31/05/15 06:00	84	132	132	192
31/05/15 06:05	144	120	96	192
31/05/15 06:10	96	156	144	204
31/05/15 06:15	108	204	96	300
31/05/15 06:20	180	216	168	324
31/05/15 06:25	240	144	132	300
31/05/15 06:30	180	216	156	336
31/05/15 06:35	204	192	96	312
31/05/15 06:40	288	264	168	444
31/05/15 06:45	300	360	144	564
31/05/15 06:50	408	348	108	672
31/05/15 06:55	360	408	216	672
31/05/15 07:00	252	168	144	348
31/05/15 07:05	216	324	120	468
31/05/15 07:10	252	324	120	432
31/05/15 07:15	264	144	120	348
31/05/15 07:20	336	276	120	504
31/05/15 07:25	300	300	120	432
31/05/15 07:30	228	312	144	432
31/05/15 07:35	300	300	240	480
31/05/15 07:40	324	312	240	504
31/05/15 07:45	276	348	240	504
31/05/15 07:50	360	312	240	648
31/05/15 07:55	276	324	240	504
31/05/15 08:00	432	300	240	540
31/05/15 08:05	372	216	240	516
31/05/15 08:10	360	312	240	516
31/05/15 08:15	432	300	240	552
31/05/15 08:20	504	348	240	636
31/05/15 08:25	420	384	240	552
31/05/15 08:30	444	444	240	600
31/05/15 08:35	348	492	240	516
31/05/15 08:40	468	432	240	696
31/05/15 08:45	528	384	240	804
31/05/15 08:50	636	444	240	876
31/05/15 08:55	444	432	240	780
31/05/15 09:00	528	384	240	696
31/05/15 09:05	516	312	240	660
31/05/15 09:10	564	324	240	708
31/05/15 09:15	564	348	264	528

Sistema de Controle de Tráfego Urbano OPTIMUS

5 MINUTOS	INTENSIDADE			
	PM 0401003	PM 0401004	PM 0401006	PM 0402001
31/05/15 09:20	600	348	360	864
31/05/15 09:25	588	468	360	816
31/05/15 09:30	504	348	360	744
31/05/15 09:35	528	468	360	804
31/05/15 09:40	468	408	360	696
31/05/15 09:45	744	504	360	888
31/05/15 09:50	588	504	360	732
31/05/15 09:55	588	432	360	936
31/05/15 10:00	660	444	384	960
31/05/15 10:05	588	528	480	804
31/05/15 10:10	696	444	480	912
31/05/15 10:15	576	540	480	780
31/05/15 10:20	612	552	480	996
31/05/15 10:25	816	432	480	1044
31/05/15 10:30	684	516	480	1020
31/05/15 10:35	600	708	480	828
31/05/15 10:40	780	612	480	1188
31/05/15 10:45	696	588	504	1248
31/05/15 10:50	768	564	600	960
31/05/15 10:55	612	516	600	960
31/05/15 11:00	756	348	600	936
31/05/15 11:05	708	528	600	1056
31/05/15 11:10	528	420	600	768
31/05/15 11:15	792	492	600	960
31/05/15 11:20	840	468	600	1104
31/05/15 11:25	864	396	600	1104
31/05/15 11:30	756	456	600	972
31/05/15 11:35	648	684	600	1092
31/05/15 11:40	744	564	600	1056
31/05/15 11:45	804	636	600	1056
31/05/15 11:50	948	480	600	1212
31/05/15 11:55	852	780	600	1344
31/05/15 12:00	864	588	600	1200
31/05/15 12:05	756	720	600	1068
31/05/15 12:10	948	588	600	1236
31/05/15 12:15	1020	576	624	1332
31/05/15 12:20	1140	636	720	1308
31/05/15 12:25	1176	624	720	1488
31/05/15 12:30	1104	744	744	1464
31/05/15 12:35	1296	660	840	1692
31/05/15 12:40	1044	420	840	1320
31/05/15 12:45	1068	744	840	1464
31/05/15 12:50	1200	552	840	1368
31/05/15 12:55	1272	684	840	1500
31/05/15 13:00	1032	480	840	1392
31/05/15 13:05	972	612	840	1152
31/05/15 13:10	1008	480	840	1356
31/05/15 13:15	864	576	816	1164
31/05/15 13:20	912	624	720	1032
31/05/15 13:25	1116	612	720	1488
31/05/15 13:30	696	564	744	1224
31/05/15 13:35	888	552	840	1056
31/05/15 13:40	612	612	840	1128
31/05/15 13:45	900	552	816	1200
31/05/15 13:50	960	432	720	1128
31/05/15 13:55	852	468	720	912
31/05/15 14:00	924	324	516	1080
31/05/15 14:05	828	372	720	912

Sistema de Controle de Tráfego Urbano OPTIMUS

5 MINUTOS	INTENSIDADE			
	PM 0401003	PM 0401004	PM 0401006	PM 0402001
31/05/15 14:10	612	516	720	948
31/05/15 14:15	816	528	696	1140
31/05/15 14:20	792	540	600	1128
31/05/15 14:25	792	420	600	900
31/05/15 14:30	756	564	600	1116
31/05/15 14:35	768	576	600	1152
31/05/15 14:40	816	576	600	1200
31/05/15 14:45	624	552	600	924
31/05/15 14:50	852	588	600	1080
31/05/15 14:55	708	468	600	1104
31/05/15 15:00	648	660	600	1020
31/05/15 15:05	672	720	600	1248
31/05/15 15:10	828	660	600	1344
31/05/15 15:15	852	396	600	1068
31/05/15 15:20	936	624	600	1236
31/05/15 15:25	756	576	600	1200
31/05/15 15:30	756	672	576	1200
31/05/15 15:35	636	600	480	1008
31/05/15 15:40	792	552	480	1080
31/05/15 15:45	684	564	504	1080
31/05/15 15:50	840	552	600	1140
31/05/15 15:55	696	504	600	1008
31/05/15 16:00	780	540	600	1032
31/05/15 16:05	792	552	600	1092
31/05/15 16:10	684	408	600	984
31/05/15 16:15	768	456	600	1032
31/05/15 16:20	600	492	600	912
31/05/15 16:25	684	384	600	936
31/05/15 16:30	780	480	600	984
31/05/15 16:35	528	540	600	912
31/05/15 16:40	720	480	600	996
31/05/15 16:45	756	396	576	924
31/05/15 16:50	684	468	480	888
31/05/15 16:55	696	492	480	936
31/05/15 17:00	672	468	480	1128
31/05/15 17:05	648	540	480	996
31/05/15 17:10	660	504	480	1140
31/05/15 17:15	684	528	480	924
31/05/15 17:20	528	360	480	720
31/05/15 17:25	732	576	480	1032
31/05/15 17:30	816	492	672	960
31/05/15 17:35	792	480	396	1080
31/05/15 17:40	636	516	480	984
31/05/15 17:45	732	516	480	876
31/05/15 17:50	648	672	480	1092
31/05/15 17:55	852	696	480	1236
31/05/15 18:00	792	804	468	1344
31/05/15 18:05	660	996	540	1416
31/05/15 18:10	972	1164	600	1620
31/05/15 18:15	768	1080	492	1548
31/05/15 18:20	840	1116	720	1440
31/05/15 18:25	816	1332	720	1704
31/05/15 18:30	1164	1224	804	1812
31/05/15 18:35	924	1260	624	1752
31/05/15 18:40	1152	1008	732	1596
31/05/15 18:45	888	1200	984	1764
31/05/15 18:50	888	960	768	1548
31/05/15 18:55	1032	912	828	1824

Sistema de Controle de Tráfego Urbano OPTIMUS

5 MINUTOS	INTENSIDADE			
	PM 0401003	PM 0401004	PM 0401006	PM 0402001
31/05/15 19:00	936	684	612	1308
31/05/15 19:05	744	492	720	1068
31/05/15 19:10	684	672	720	1068
31/05/15 19:15	936	516	720	1296
31/05/15 19:20	852	672	720	1248
31/05/15 19:25	852	396	720	1236
31/05/15 19:30	696	552	720	972
31/05/15 19:35	624	552	720	1056
31/05/15 19:40	684	576	648	1056
31/05/15 19:45	708	516	732	1068
31/05/15 19:50	684	420	780	900
31/05/15 19:55	660	504	828	876
31/05/15 20:00	456	276	792	684
31/05/15 20:05	648	456	732	804
31/05/15 20:10	564	360	744	696
31/05/15 20:15	708	480	684	936
31/05/15 20:20	624	444	672	924
31/05/15 20:25	732	444	636	876
31/05/15 20:30	648	480	696	852
31/05/15 20:35	900	432	684	1080
31/05/15 20:40	648	336	864	996
31/05/15 20:45	600	384	732	732
31/05/15 20:50	588	432	876	708
31/05/15 20:55	576	336	636	744
31/05/15 21:00	744	324	936	840
31/05/15 21:05	660	372	924	972
31/05/15 21:10	744	360	900	900
31/05/15 21:15	468	444	936	648
31/05/15 21:20	552	456	852	816
31/05/15 21:25	480	504	684	828
31/05/15 21:30	456	384	696	672
31/05/15 21:35	504	300	672	720
31/05/15 21:40	492	300	480	744
31/05/15 21:45	492	432	756	648
31/05/15 21:50	564	348	540	696
31/05/15 21:55	348	252	348	468
31/05/15 22:00	420	324	528	600
31/05/15 22:05	360	240	540	420
31/05/15 22:10	516	228	456	600
31/05/15 22:15	456	324	564	660
31/05/15 22:20	408	240	480	528
31/05/15 22:25	432	252	504	564
31/05/15 22:30	228	228	516	396
31/05/15 22:35	336	264	384	468
31/05/15 22:40	276	156	348	456
31/05/15 22:45	276	180	276	372
31/05/15 22:50	312	180	408	408
31/05/15 22:55	312	216	336	420
31/05/15 23:00	360	132	552	456
31/05/15 23:05	300	288	312	516
31/05/15 23:10	192	216	252	348
31/05/15 23:15	204	252	252	348
31/05/15 23:20	312	240	360	420
31/05/15 23:25	204	96	324	312
31/05/15 23:30	180	156	324	228
31/05/15 23:35	252	132	312	300
31/05/15 23:40	168	84	264	204
31/05/15 23:45	264	168	264	360

Sistema de Controle de Tráfego Urbano OPTIMUS

5 MINUTOS	INTENSIDADE			
	PM 0401003	PM 0401004	PM 0401006	PM 0402001
31/05/15 23:50	168	132	240	216
31/05/15 23:55	180	120	192	252
01/06/15 00:00	144	108	276	156
01/06/15 00:05	144	84	204	180
01/06/15 00:10	84	72	132	132
01/06/15 00:15	108	132	192	156
01/06/15 00:20	144	84	192	156
01/06/15 00:25	60	36	144	48
01/06/15 00:30	48	132	144	108
01/06/15 00:35	24	84	192	60
01/06/15 00:40	72	72	168	108
01/06/15 00:45	72	132	108	108
01/06/15 00:50	84	48	108	156
01/06/15 00:55	108	72	168	120
01/06/15 01:00	48	36	72	60
01/06/15 01:05	72	12	108	48
01/06/15 01:10	60	36	72	72
01/06/15 01:15	36	36	72	36
01/06/15 01:20	48	72	96	60
01/06/15 01:25	48	36	120	96
01/06/15 01:30	36	12	48	36
01/06/15 01:35	96	72	36	108
01/06/15 01:40	12	36	48	24
01/06/15 01:45	24	36	60	60
01/06/15 01:50	60	108	36	132
01/06/15 01:55	24	48	24	60
01/06/15 02:00	24	0	60	0
01/06/15 02:05	72	36	36	48
01/06/15 02:10	36	24	84	60
01/06/15 02:15	24	84	84	108
01/06/15 02:20	72	48	36	60
01/06/15 02:25	24	12	48	72
01/06/15 02:30	24	36	24	36
01/06/15 02:35	72	24	24	60
01/06/15 02:40	24	24	60	36
01/06/15 02:45	36	24	60	72
01/06/15 02:50	72	48	72	120
01/06/15 02:55	24	12	84	60
01/06/15 03:00	48	24	36	36
01/06/15 03:05	60	12	36	24
01/06/15 03:10	36	24	48	48
01/06/15 03:15	36	12	24	36
01/06/15 03:20	24	48	72	60
01/06/15 03:25	12	12	72	12
01/06/15 03:30	36	108	12	96
01/06/15 03:35	12	12	24	24
01/06/15 03:40	24	36	12	36
01/06/15 03:45	60	60	36	96
01/06/15 03:50	12	36	12	48
01/06/15 03:55	24	36	36	48
01/06/15 04:00	24	36	36	36
01/06/15 04:05	24	12	36	36
01/06/15 04:10	60	36	12	60
01/06/15 04:15	24	72	24	60
01/06/15 04:20	84	36	12	108
01/06/15 04:25	24	60	36	48
01/06/15 04:30	36	24	84	60
01/06/15 04:35	36	96	0	120

Sistema de Controle de Tráfego Urbano OPTIMUS

5 MINUTOS	INTENSIDADE			
	PM 0401003	PM 0401004	PM 0401006	PM 0402001
01/06/15 04:40	24	24	24	48
01/06/15 04:45	96	48	36	120
01/06/15 04:50	60	48	12	84
01/06/15 04:55	48	12	36	60
01/06/15 05:00	72	96	60	96
01/06/15 05:05	84	84	36	156
01/06/15 05:10	84	72	48	156
01/06/15 05:15	96	132	60	120
01/06/15 05:20	96	180	96	180
01/06/15 05:25	156	156	120	216
01/06/15 05:30	144	240	48	396
01/06/15 05:35	168	192	72	264
01/06/15 05:40	180	300	120	432
01/06/15 05:45	228	228	108	432
01/06/15 05:50	384	252	156	528
01/06/15 05:55	300	240	120	456
01/06/15 06:00	372	276	156	504
01/06/15 06:05	540	492	192	816
01/06/15 06:10	528	372	180	744
01/06/15 06:15	468	312	300	756
01/06/15 06:20	744	576	144	1008
01/06/15 06:25	780	612	276	1080
01/06/15 06:30	1284	564	360	1728
01/06/15 06:35	1080	684	504	1416
01/06/15 06:40	1488	696	276	2052
01/06/15 06:45	1512	804	708	2160
01/06/15 06:50	1308	936	756	1980
01/06/15 06:55	1728	708	624	2184
01/06/15 07:00	1740	744	1284	2124
01/06/15 07:05	1800	696	936	2328
01/06/15 07:10	1656	660	924	2052
01/06/15 07:15	1668	732	948	2256
01/06/15 07:20	1296	744	864	1860
01/06/15 07:25	1464	660	1092	2016
01/06/15 07:30	1716	672	864	2004
01/06/15 07:35	1644	732	792	2232
01/06/15 07:40	1380	660	600	1932
01/06/15 07:45	1728	576	912	2184
01/06/15 07:50	1620	588	792	1956
01/06/15 07:55	1656	660	996	2040
01/06/15 08:00	1584	588	804	1956
01/06/15 08:05	1572	612	840	2244
01/06/15 08:10	1404	696	720	1728
01/06/15 08:15	1512	660	984	1980
01/06/15 08:20	1380	672	852	1800
01/06/15 08:25	1296	720	780	1812
01/06/15 08:30	1428	624	792	1668
01/06/15 08:35	1284	684	996	1776
01/06/15 08:40	1404	816	828	1908
01/06/15 08:45	1380	624	972	1932
01/06/15 08:50	1236	660	720	1536
01/06/15 08:55	1440	528	876	1704
01/06/15 09:00	1596	744	888	2028
01/06/15 09:05	1332	552	936	1908
01/06/15 09:10	1332	660	960	1680
01/06/15 09:15	1224	756	864	1560
01/06/15 09:20	1308	648	840	1704
01/06/15 09:25	1224	708	1140	1776

Sistema de Controle de Tráfego Urbano OPTIMUS

5 MINUTOS	INTENSIDADE			
	PM 0401003	PM 0401004	PM 0401006	PM 0402001
01/06/15 09:30	960	648	972	1404
01/06/15 09:35	1200	792	1056	1716
01/06/15 09:40	1296	744	756	1656
01/06/15 09:45	1224	684	1044	1488
01/06/15 09:50	1104	840	720	1536
01/06/15 09:55	1212	804	888	1740
01/06/15 10:00	1440	720	1296	1812
01/06/15 10:05	1092	684	864	1572
01/06/15 10:10	1080	648	888	1476
01/06/15 10:15	1332	624	912	1536
01/06/15 10:20	1104	780	852	1800
01/06/15 10:25	960	792	1068	1512
01/06/15 10:30	996	840	1116	1536
01/06/15 10:35	984	576	936	1428
01/06/15 10:40	984	732	1080	1392
01/06/15 10:45	1116	624	1020	1320
01/06/15 10:50	1032	576	1164	1296
01/06/15 10:55	1212	528	1008	1320
01/06/15 11:00	1044	612	1188	1476
01/06/15 11:05	1236	612	996	1344
01/06/15 11:10	1068	816	1104	1500
01/06/15 11:15	912	720	1392	1248
01/06/15 11:20	1104	444	1296	1248
01/06/15 11:25	1140	696	1332	1572
01/06/15 11:30	1044	600	1344	1476
01/06/15 11:35	936	696	1404	1320
01/06/15 11:40	1224	684	1404	1680
01/06/15 11:45	936	648	1092	1248
01/06/15 11:50	1224	708	1176	1476
01/06/15 11:55	1344	684	1404	1728
01/06/15 12:00	1284	660	1416	1632
01/06/15 12:05	1224	648	1416	1524
01/06/15 12:10	1140	480	1392	1164
01/06/15 12:15	1236	624	1608	1596
01/06/15 12:20	1188	732	1200	1272
01/06/15 12:25	960	792	1152	1332
01/06/15 12:30	1200	672	1608	1584
01/06/15 12:35	1428	612	1152	1656
01/06/15 12:40	1356	744	948	1584
01/06/15 12:45	1296	792	1248	1896
01/06/15 12:50	1392	732	1032	1740
01/06/15 12:55	1464	696	1056	1896
01/06/15 13:00	1536	768	1104	1836
01/06/15 13:05	1320	792	1140	1632
01/06/15 13:10	1524	744	1320	1620
01/06/15 13:15	1548	792	924	1980
01/06/15 13:20	1560	624	960	1824
01/06/15 13:25	1560	612	960	1644
01/06/15 13:30	996	660	960	1608
01/06/15 13:35	1140	780	960	1536
01/06/15 13:40	1356	732	960	1884
01/06/15 13:45	1404	552	960	1680
01/06/15 13:50	1236	756	960	1668
01/06/15 13:55	1284	828	960	1764
01/06/15 14:00	1416	660	960	1764
01/06/15 14:05	1128	744	960	1668
01/06/15 14:10	1548	636	960	1776
01/06/15 14:15	1200	672	960	2016

Sistema de Controle de Tráfego Urbano OPTIMUS

5 MINUTOS	INTENSIDADE			
	PM 0401003	PM 0401004	PM 0401006	PM 0402001
01/06/15 14:20	1356	672	960	1608
01/06/15 14:25	1248	744	960	1728
01/06/15 14:30	1248	564	984	1536
01/06/15 14:35	1320	732	1104	1716
01/06/15 14:40	1380	480	912	1524
01/06/15 14:45	1188	900	1452	1848
01/06/15 14:50	1332	672	900	1728
01/06/15 14:55	1284	732	1080	1800
01/06/15 15:00	1320	612	1080	1596
01/06/15 15:05	972	648	1080	1440
01/06/15 15:10	996	780	1080	1392
01/06/15 15:15	1104	648	1080	1560
01/06/15 15:20	1236	780	1080	1392
01/06/15 15:25	960	696	1080	1512
01/06/15 15:30	1068	636	1080	1560
01/06/15 15:35	1308	744	1080	1536
01/06/15 15:40	1140	600	1080	1548
01/06/15 15:45	1032	816	1080	1284
01/06/15 15:50	1164	636	1080	1512
01/06/15 15:55	984	564	1080	1200
01/06/15 16:00	1092	684	1104	1224
01/06/15 16:05	1116	660	1200	1392
01/06/15 16:10	1056	720	1200	1476
01/06/15 16:15	1284	600	1116	1536
01/06/15 16:20	1128	720	1128	1488
