# Supplementary material for: Traffic light optimization using non-dominated sorting genetic algorithm (NSGA2)
Source: Sci Rep. 2023 Sep 20;13:15550. doi: 10.1038/s41598-023-38884-2 (PMC10511403; doi:10.1038/s41598-023-38884-2)
Supplement: Supplementary file 1 — Supplementary Information. [file 41598_2023_38884_MOESM1_ESM.zip › dadosBHTrans/Dados-BHTRANS02/I_1_dat_4_cs_5_min_st2.pdf]

Sistema de Controle de Tráfego Urbano OPTIMUS

INTENSIDADE DE 4 PONTOS DE MEDIDA
DADOS DE 5 MINUTOS

PONTO DE MEDIDA 1:PM 04020 04 (Contorno)
PONTO DE MEDIDA 2:PM 04020 07 (Contorno)
PONTO DE MEDIDA 3:PM 04030 031 (Contorno)
PONTO DE MEDIDA 4:PM 04030 032 ()
DESDE:01/05/2015 04:06
ATÉ:01/06/201516:24

INTENSIDADE / 5 MINUTOS

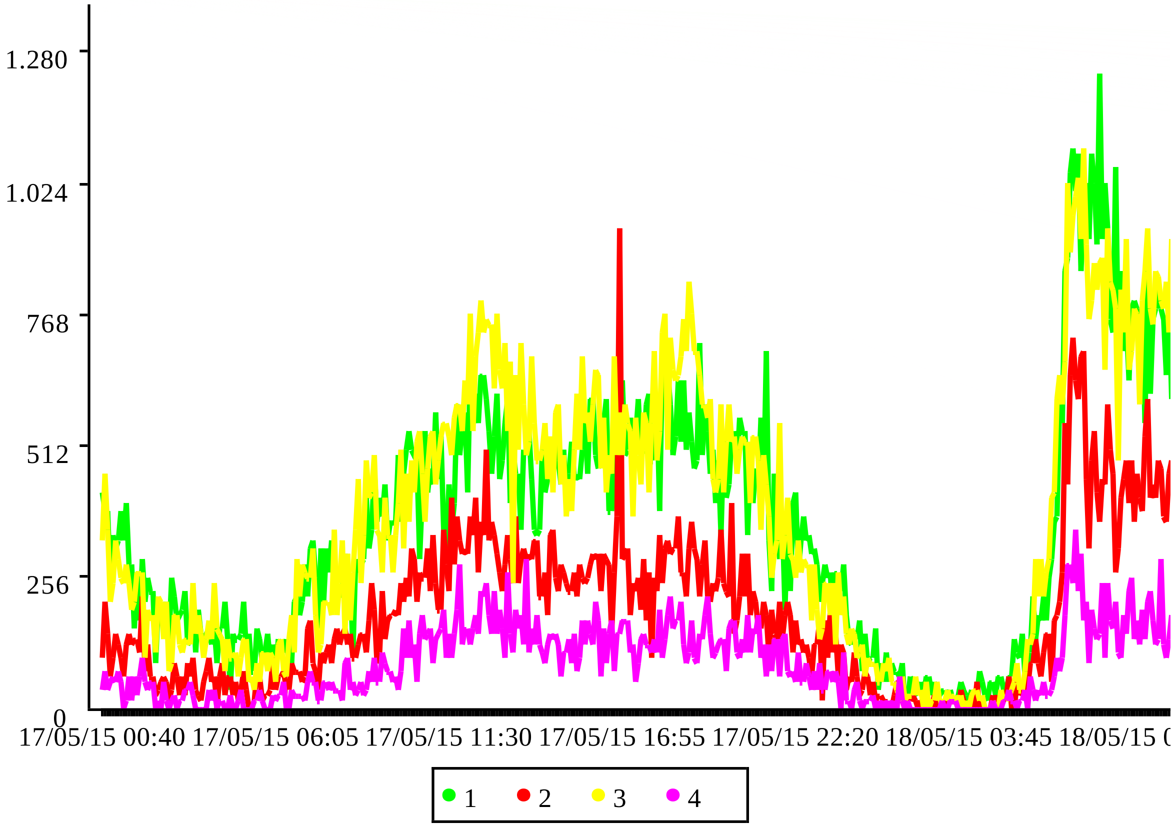

5 MINUTOS	INTENSIDADE			
	PM 0402004	PM 0402007	PM 04030031	PM 04030032
17/05/15 00:40	420	96	324	36
17/05/15 00:45	384	204	456	72
17/05/15 00:50	384	144	348	36
17/05/15 00:55	240	60	204	48
17/05/15 01:00	336	96	240	60
17/05/15 01:05	312	144	324	48
17/05/15 01:10	324	120	288	72
17/05/15 01:15	384	96	252	48
17/05/15 01:20	324	60	240	0
17/05/15 01:25	396	120	276	12
17/05/15 01:30	276	144	240	60
17/05/15 01:35	276	120	192	12
17/05/15 01:40	156	132	204	60
17/05/15 01:45	264	132	264	24
17/05/15 01:50	168	108	264	60

Sistema de Controle de Tráfego Urbano OPTIMUS

5 MINUTOS	INTENSIDADE			
	PM 0402004	PM 0402007	PM04030031	PM04030032
17/05/15 01:55	288	216	264	96
17/05/15 02:00	204	72	96	48
17/05/15 02:05	252	120	192	36
17/05/15 02:10	228	60	168	48
17/05/15 02:15	228	60	180	48
17/05/15 02:20	84	24	108	0
17/05/15 02:25	204	36	216	12
17/05/15 02:30	168	48	204	0
17/05/15 02:35	180	60	132	48
17/05/15 02:40	204	48	204	12
17/05/15 02:45	132	12	72	0
17/05/15 02:50	252	36	84	12
17/05/15 02:55	216	84	180	24
17/05/15 03:00	168	60	180	0
17/05/15 03:05	180	24	120	12
17/05/15 03:10	192	60	108	12
17/05/15 03:15	228	24	132	36
17/05/15 03:20	120	84	132	36
17/05/15 03:25	132	60	120	48
17/05/15 03:30	216	96	240	24
17/05/15 03:35	108	36	144	0
17/05/15 03:40	192	24	180	0
17/05/15 03:45	144	12	144	0
17/05/15 03:50	132	48	96	0
17/05/15 03:55	144	72	132	0
17/05/15 04:00	156	96	168	36
17/05/15 04:05	120	48	132	12
17/05/15 04:10	156	60	240	36
17/05/15 04:15	84	0	156	0
17/05/15 04:20	156	60	144	12
17/05/15 04:25	156	84	132	12
17/05/15 04:30	204	24	72	0
17/05/15 04:35	132	60	132	0
17/05/15 04:40	36	60	96	24
17/05/15 04:45	144	24	96	0
17/05/15 04:50	132	48	108	0
17/05/15 04:55	132	12	60	12
17/05/15 05:00	144	36	96	36
17/05/15 05:05	204	72	132	0
17/05/15 05:10	108	36	132	0
17/05/15 05:15	144	0	48	0
17/05/15 05:20	72	0	60	0
17/05/15 05:25	72	12	36	12
17/05/15 05:30	156	72	84	12
17/05/15 05:35	132	60	48	36
17/05/15 05:40	84	12	108	12
17/05/15 05:45	108	24	108	0
17/05/15 05:50	144	24	72	0
17/05/15 05:55	84	36	108	0
17/05/15 06:00	120	96	96	24
17/05/15 06:05	72	36	48	12
17/05/15 06:10	132	60	120	24
17/05/15 06:15	108	60	132	24
17/05/15 06:20	108	72	72	48
17/05/15 06:25	132	36	60	0
17/05/15 06:30	84	36	132	0
17/05/15 06:35	84	84	180	36
17/05/15 06:40	204	72	108	24

Sistema de Controle de Tráfego Urbano OPTIMUS

5 MINUTOS	INTENSIDADE			
	PM 0402004	PM 0402007	PM04030031	PM04030032
17/05/15 06:45	216	72	288	24
17/05/15 06:50	180	72	216	24
17/05/15 06:55	204	48	276	24
17/05/15 07:00	276	60	264	12
17/05/15 07:05	216	156	252	48
17/05/15 07:10	312	168	252	72
17/05/15 07:15	324	84	312	48
17/05/15 07:20	252	72	180	48
17/05/15 07:25	156	36	108	12
17/05/15 07:30	312	108	120	12
17/05/15 07:35	204	84	204	48
17/05/15 07:40	312	108	204	48
17/05/15 07:45	264	120	204	36
17/05/15 07:50	324	84	180	48
17/05/15 07:55	264	132	348	36
17/05/15 08:00	228	156	180	36
17/05/15 08:05	216	120	240	36
17/05/15 08:10	228	144	324	12
17/05/15 08:15	300	156	144	84
17/05/15 08:20	180	120	300	96
17/05/15 08:25	252	144	168	36
17/05/15 08:30	108	96	264	48
17/05/15 08:35	288	96	348	24
17/05/15 08:40	264	120	444	36
17/05/15 08:45	276	144	240	48
17/05/15 08:50	288	144	336	24
17/05/15 08:55	408	108	480	36
17/05/15 09:00	312	168	408	72
17/05/15 09:05	348	240	420	48
17/05/15 09:10	468	192	492	96
17/05/15 09:15	360	84	348	48
17/05/15 09:20	408	108	336	48
17/05/15 09:25	372	228	264	108
17/05/15 09:30	432	132	408	84
17/05/15 09:35	324	168	336	72
17/05/15 09:40	360	180	336	72
17/05/15 09:45	360	180	264	48
17/05/15 09:50	360	192	336	60
17/05/15 09:55	492	180	396	36
17/05/15 10:00	360	240	504	72
17/05/15 10:05	456	204	312	156
17/05/15 10:10	504	252	456	96
17/05/15 10:15	540	216	360	168
17/05/15 10:20	504	312	480	96
17/05/15 10:25	492	288	420	120
17/05/15 10:30	480	204	516	48
17/05/15 10:35	288	264	540	144
17/05/15 10:40	420	252	456	180
17/05/15 10:45	540	252	360	132
17/05/15 10:50	420	312	468	144
17/05/15 10:55	468	228	528	156
17/05/15 11:00	432	336	540	84
17/05/15 11:05	576	240	432	132
17/05/15 11:10	444	192	480	144
17/05/15 11:15	528	192	528	156
17/05/15 11:20	348	348	552	192
17/05/15 11:25	312	276	552	96
17/05/15 11:30	432	228	528	144

Sistema de Controle de Tráfego Urbano OPTIMUS

5 MINUTOS	INTENSIDADE			
	PM 0402004	PM 0402007	PM04030031	PM04030032
17/05/15 11:35	348	408	492	96
17/05/15 11:40	396	276	564	144
17/05/15 11:45	588	372	588	192
17/05/15 11:50	492	324	588	276
17/05/15 11:55	540	300	540	120
17/05/15 12:00	576	312	636	156
17/05/15 12:05	420	300	588	156
17/05/15 12:10	672	372	768	120
17/05/15 12:15	576	336	540	156
17/05/15 12:20	612	408	684	180
17/05/15 12:25	552	264	732	144
17/05/15 12:30	648	360	792	228
17/05/15 12:35	648	336	732	216
17/05/15 12:40	600	504	756	240
17/05/15 12:45	540	324	744	180
17/05/15 12:50	456	360	744	144
17/05/15 12:55	528	336	624	228
17/05/15 13:00	612	300	768	144
17/05/15 13:05	444	264	660	192
17/05/15 13:10	480	228	624	156
17/05/15 13:15	552	276	708	96
17/05/15 13:20	600	336	540	264
17/05/15 13:25	396	288	672	144
17/05/15 13:30	504	240	240	108
17/05/15 13:35	456	372	648	192
17/05/15 13:40	456	240	504	156
17/05/15 13:45	348	288	708	180
17/05/15 13:50	504	312	588	120
17/05/15 13:55	492	288	492	288
17/05/15 14:00	540	300	516	108
17/05/15 14:05	444	288	684	156
17/05/15 14:10	348	324	552	120
17/05/15 14:15	336	324	480	180
17/05/15 14:20	348	216	480	120
17/05/15 14:25	504	216	504	108
17/05/15 14:30	420	264	552	84
17/05/15 14:35	444	180	444	120
17/05/15 14:40	468	336	528	144
17/05/15 14:45	456	348	420	132
17/05/15 14:50	516	252	576	144
17/05/15 14:55	480	228	588	120
17/05/15 15:00	432	276	432	60
17/05/15 15:05	504	264	492	108
17/05/15 15:10	408	240	372	108
17/05/15 15:15	456	228	444	132
17/05/15 15:20	516	228	384	84
17/05/15 15:25	444	264	504	144
17/05/15 15:30	456	216	612	72
17/05/15 15:35	444	276	504	96
17/05/15 15:40	504	252	684	168
17/05/15 15:45	564	240	552	120
17/05/15 15:50	456	252	576	168
17/05/15 15:55	600	276	516	144
17/05/15 16:00	600	300	588	120
17/05/15 16:05	492	288	660	204
17/05/15 16:10	468	300	648	168
17/05/15 16:15	516	228	468	60
17/05/15 16:20	552	300	564	156

Sistema de Controle de Tráfego Urbano OPTIMUS

5 MINUTOS	INTENSIDADE			
	PM 0402004	PM 0402007	PM04030031	PM04030032
17/05/15 16:25	600	288	420	84
17/05/15 16:30	384	276	492	144
17/05/15 16:35	384	144	456	168
17/05/15 16:40	480	264	684	72
17/05/15 16:45	540	372	492	144
17/05/15 16:50	420	936	576	144
17/05/15 16:55	636	288	492	168
17/05/15 17:00	492	300	588	168
17/05/15 17:05	504	312	552	168
17/05/15 17:10	564	180	540	108
17/05/15 17:15	444	240	372	120
17/05/15 17:20	528	228	564	48
17/05/15 17:25	600	252	492	84
17/05/15 17:30	492	192	432	132
17/05/15 17:35	504	288	576	144
17/05/15 17:40	588	168	552	120
17/05/15 17:45	612	264	420	120
17/05/15 17:50	480	96	552	108
17/05/15 17:55	480	252	696	132
17/05/15 18:00	588	228	480	108
17/05/15 18:05	384	336	540	192
17/05/15 18:10	672	240	732	96
17/05/15 18:15	624	312	768	132
17/05/15 18:20	660	324	504	180
17/05/15 18:25	588	300	720	216
17/05/15 18:30	492	312	660	156
17/05/15 18:35	528	312	636	168
17/05/15 18:40	636	372	648	168
17/05/15 18:45	516	264	684	204
17/05/15 18:50	636	264	756	120
17/05/15 18:55	504	216	696	84
17/05/15 19:00	576	312	828	108
17/05/15 19:05	516	360	768	168
17/05/15 19:10	468	312	696	96
17/05/15 19:15	480	288	696	84
17/05/15 19:20	708	216	648	144
17/05/15 19:25	492	276	588	132
17/05/15 19:30	588	324	588	180
17/05/15 19:35	540	216	564	216
17/05/15 19:40	456	204	600	144
17/05/15 19:45	504	240	444	96
17/05/15 19:50	396	228	420	108
17/05/15 19:55	480	252	444	120
17/05/15 20:00	336	348	588	132
17/05/15 20:05	516	240	420	132
17/05/15 20:10	408	228	516	72
17/05/15 20:15	432	216	588	156
17/05/15 20:20	528	396	516	168
17/05/15 20:25	540	144	528	168
17/05/15 20:30	468	168	456	108
17/05/15 20:35	564	216	504	96
17/05/15 20:40	540	300	528	132
17/05/15 20:45	540	216	516	108
17/05/15 20:50	336	300	516	180
17/05/15 20:55	492	180	396	108
17/05/15 21:00	396	228	528	156
17/05/15 21:05	468	204	528	144
17/05/15 21:10	432	156	480	180

Sistema de Controle de Tráfego Urbano OPTIMUS

5 MINUTOS	INTENSIDADE			
	PM 0402004	PM 0402007	PM04030031	PM04030032
17/05/15 21:15	564	156	348	96
17/05/15 21:20	420	204	492	120
17/05/15 21:25	696	180	432	60
17/05/15 21:30	324	96	372	120
17/05/15 21:35	228	192	252	72
17/05/15 21:40	456	144	300	132
17/05/15 21:45	360	132	324	132
17/05/15 21:50	288	204	552	60
17/05/15 21:55	372	132	288	132
17/05/15 22:00	192	180	336	144
17/05/15 22:05	372	204	408	72
17/05/15 22:10	228	180	300	60
17/05/15 22:15	396	108	288	72
17/05/15 22:20	420	168	252	48
17/05/15 22:25	264	120	324	108
17/05/15 22:30	324	132	264	48
17/05/15 22:35	372	108	288	48
17/05/15 22:40	324	120	276	84
17/05/15 22:45	336	96	276	60
17/05/15 22:50	300	60	168	36
17/05/15 22:55	312	120	276	72
17/05/15 23:00	276	120	192	36
17/05/15 23:05	204	168	132	84
17/05/15 23:10	240	12	156	36
17/05/15 23:15	276	168	240	36
17/05/15 23:20	252	96	240	72
17/05/15 23:25	264	192	180	72
17/05/15 23:30	168	108	240	48
17/05/15 23:35	264	144	120	72
17/05/15 23:40	264	60	264	48
17/05/15 23:45	228	120	180	0
17/05/15 23:50	276	108	216	108
17/05/15 23:55	180	36	144	12
18/05/15 00:00	132	60	120	12
18/05/15 00:05	120	48	156	0
18/05/15 00:10	144	108	144	36
18/05/15 00:15	132	60	96	48
18/05/15 00:20	168	24	108	12
18/05/15 00:25	72	36	120	0
18/05/15 00:30	144	72	60	12
18/05/15 00:35	84	60	96	12
18/05/15 00:40	96	24	84	12
18/05/15 00:45	96	48	84	24
18/05/15 00:50	156	12	84	0
18/05/15 00:55	36	24	48	12
18/05/15 01:00	84	24	48	0
18/05/15 01:05	48	12	84	12
18/05/15 01:10	108	12	72	0
18/05/15 01:15	48	12	96	12
18/05/15 01:20	84	0	48	0
18/05/15 01:25	72	24	48	12
18/05/15 01:30	72	48	36	0
18/05/15 01:35	60	48	24	60
18/05/15 01:40	84	12	60	12
18/05/15 01:45	0	0	12	0
18/05/15 01:50	12	24	24	12
18/05/15 01:55	60	12	24	0
18/05/15 02:00	48	24	24	0

Sistema de Controle de Tráfego Urbano OPTIMUS

5 MINUTOS	INTENSIDADE			
	PM 0402004	PM 0402007	PM04030031	PM04030032
18/05/15 02:05	36	12	60	0
18/05/15 02:10	24	0	24	0
18/05/15 02:15	48	12	36	0
18/05/15 02:20	24	12	0	0
18/05/15 02:25	60	0	48	0
18/05/15 02:30	60	0	0	0
18/05/15 02:35	24	0	12	0
18/05/15 02:40	48	24	12	0
18/05/15 02:45	24	0	48	0
18/05/15 02:50	24	0	12	0
18/05/15 02:55	36	0	12	12
18/05/15 03:00	36	0	12	0
18/05/15 03:05	12	12	36	0
18/05/15 03:10	36	24	12	12
18/05/15 03:15	12	12	24	12
18/05/15 03:20	12	12	24	12
18/05/15 03:25	24	0	0	0
18/05/15 03:30	48	36	24	0
18/05/15 03:35	36	12	0	0
18/05/15 03:40	24	0	12	0
18/05/15 03:45	12	0	0	0
18/05/15 03:50	36	24	24	0
18/05/15 03:55	24	0	36	0
18/05/15 04:00	24	48	36	0
18/05/15 04:05	72	0	12	0
18/05/15 04:10	48	12	24	0
18/05/15 04:15	24	0	0	0
18/05/15 04:20	12	0	0	0
18/05/15 04:25	48	0	0	0
18/05/15 04:30	48	24	12	12
18/05/15 04:35	24	0	0	0
18/05/15 04:40	60	24	0	0
18/05/15 04:45	60	12	36	0
18/05/15 04:50	36	12	12	12
18/05/15 04:55	36	24	12	12
18/05/15 05:00	60	60	24	36
18/05/15 05:05	60	0	36	12
18/05/15 05:10	132	0	48	12
18/05/15 05:15	108	48	84	0
18/05/15 05:20	96	24	36	12
18/05/15 05:25	144	36	36	12
18/05/15 05:30	60	60	72	36
18/05/15 05:35	84	36	132	0
18/05/15 05:40	108	84	120	60
18/05/15 05:45	216	84	144	36
18/05/15 05:50	132	132	288	12
18/05/15 05:55	180	84	216	36
18/05/15 06:00	168	60	288	24
18/05/15 06:05	216	108	216	48
18/05/15 06:10	168	144	264	24
18/05/15 06:15	252	144	288	24
18/05/15 06:20	288	48	408	36
18/05/15 06:25	360	168	420	60
18/05/15 06:30	372	180	600	96
18/05/15 06:35	600	204	648	72
18/05/15 06:40	552	264	588	96
18/05/15 06:45	852	552	672	180
18/05/15 06:50	876	432	1020	276

5 MINUTOS	INTENSIDADE			
	PM 0402004	PM 0402007	PM04030031	PM04030032
18/05/15 06:55	1044	648	888	276
18/05/15 07:00	1092	720	960	240
18/05/15 07:05	1008	636	996	348
18/05/15 07:10	1080	600	1032	228
18/05/15 07:15	852	684	912	300
18/05/15 07:20	972	696	1092	168
18/05/15 07:25	1020	444	900	204
18/05/15 07:30	912	312	756	84
18/05/15 07:35	1080	480	792	192
18/05/15 07:40	1020	540	864	156
18/05/15 07:45	900	420	816	132
18/05/15 07:50	1236	360	864	144
18/05/15 07:55	912	444	876	240
18/05/15 08:00	1020	432	660	96
18/05/15 08:05	936	588	936	240
18/05/15 08:10	756	504	828	168
18/05/15 08:15	732	456	816	156
18/05/15 08:20	1056	264	780	204
18/05/15 08:25	696	312	480	108
18/05/15 08:30	852	408	816	96
18/05/15 08:35	696	444	732	180
18/05/15 08:40	756	480	912	144
18/05/15 08:45	636	396	660	228
18/05/15 08:50	780	480	696	252
18/05/15 08:55	792	360	780	132
18/05/15 09:00	780	456	768	168
18/05/15 09:05	696	408	588	120
18/05/15 09:10	768	384	792	192
18/05/15 09:15	552	528	852	132
18/05/15 09:20	864	600	936	204
18/05/15 09:25	612	408	780	228
18/05/15 09:30	756	432	744	168
18/05/15 09:35	780	408	852	120
18/05/15 09:40	816	480	840	132
18/05/15 09:45	780	468	792	288
18/05/15 09:50	756	372	780	120
18/05/15 09:55	648	360	828	96
18/05/15 10:00	804	456	732	132
18/05/15 10:05	600	480	912	180
18/05/15 10:10	780	408	840	156
18/05/15 10:15	552	480	636	204
18/05/15 10:20	684	420	672	132
18/05/15 10:25	708	444	684	144
18/05/15 10:30	696	492	792	252
18/05/15 10:35	732	456	636	144
18/05/15 10:40	792	492	780	180
18/05/15 10:45	612	492	780	192
18/05/15 10:50	804	384	924	132
18/05/15 10:55	564	348	576	192
18/05/15 11:00	516	360	852	132
18/05/15 11:05	624	504	624	240
18/05/15 11:10	660	408	756	180
18/05/15 11:15	816	540	804	228
18/05/15 11:20	528	516	840	240
18/05/15 11:25	768	564	756	276
18/05/15 11:30	636	384	744	168
18/05/15 11:35	612	408	864	228
18/05/15 11:40	600	456	792	216

Sistema de Controle de Tráfego Urbano OPTIMUS

5 MINUTOS	INTENSIDADE			
	PM 0402004	PM 0402007	PM04030031	PM04030032
18/05/15 11:45	780	540	672	144
18/05/15 11:50	684	492	828	252
18/05/15 11:55	648	552	1140	360
18/05/15 12:00	588	708	1032	252
18/05/15 12:05	600	516	924	228
18/05/15 12:10	480	540	864	192
18/05/15 12:15	636	588	900	324
18/05/15 12:20	612	420	852	204
18/05/15 12:25	636	612	888	288
18/05/15 12:30	516	552	912	156
18/05/15 12:35	648	492	924	180
18/05/15 12:40	684	456	792	264
18/05/15 12:45	576	696	864	336
18/05/15 12:50	720	612	972	252
18/05/15 12:55	1008	780	1020	276
18/05/15 13:00	648	552	852	252
18/05/15 13:05	792	624	984	336
18/05/15 13:10	756	492	948	216
18/05/15 13:15	912	540	888	312
18/05/15 13:20	744	528	1008	276
18/05/15 13:25	552	540	1008	228
18/05/15 13:30	912	444	804	180
18/05/15 13:35	900	408	864	168
18/05/15 13:40	612	468	864	228
18/05/15 13:45	804	504	972	132
18/05/15 13:50	768	384	876	180
18/05/15 13:55	732	612	984	252
18/05/15 14:00	1056	396	924	348
18/05/15 14:05	756	576	924	216
18/05/15 14:10	984	312	564	120
18/05/15 14:15	888	360	720	156
18/05/15 14:20	624	492	972	228
18/05/15 14:25	888	432	852	192
18/05/15 14:30	720	480	732	228
18/05/15 14:35	696	672	804	264
18/05/15 14:40	684	348	888	180
18/05/15 14:45	732	660	876	288
18/05/15 14:50	684	372	924	240
18/05/15 14:55	708	540	720	108
18/05/15 15:00	732	540	696	144
18/05/15 15:05	864	552	1128	228
18/05/15 15:10	588	504	732	228
18/05/15 15:15	672	492	696	168
18/05/15 15:20	804	444	996	120
18/05/15 15:25	744	336	804	156
18/05/15 15:30	732	600	936	264
18/05/15 15:35	756	420	852	144
18/05/15 15:40	720	708	768	336
18/05/15 15:45	660	360	624	204
18/05/15 15:50	672	636	888	156
18/05/15 15:55	804	444	876	156
18/05/15 16:00	696	408	792	240
18/05/15 16:05	540	468	708	192
18/05/15 16:10	756	456	804	276
18/05/15 16:15	756	252	720	120
18/05/15 16:20	636	408	768	216
18/05/15 16:25	948	396	708	228
18/05/15 16:30	720	480	672	228

Sistema de Controle de Tráfego Urbano OPTIMUS

5 MINUTOS	INTENSIDADE			
	PM 0402004	PM 0402007	PM04030031	PM04030032
18/05/15 16:35	660	468	864	120
18/05/15 16:40	696	576	804	312
18/05/15 16:45	732	660	804	252
18/05/15 16:50	612	828	924	420
18/05/15 16:55	624	480	912	264
18/05/15 17:00	744	636	792	252
18/05/15 17:05	828	720	708	336
18/05/15 17:10	528	372	420	120
18/05/15 17:15	408	684	840	228
18/05/15 17:20	600	324	1080	348
18/05/15 17:25	612	636	1032	288
18/05/15 17:30	600	264	684	240
18/05/15 17:35	360	648	1116	204
18/05/15 17:40	576	372	684	180
18/05/15 17:45	588	576	900	240
18/05/15 17:50	612	528	816	204
18/05/15 17:55	468	660	708	324
18/05/15 18:00	384	468	708	216
18/05/15 18:05	324	600	768	228
18/05/15 18:10	504	456	744	180
18/05/15 18:15	552	312	1116	156
18/05/15 18:20	348	408	864	192
18/05/15 18:25	408	504	960	276
18/05/15 18:30	456	600	576	276
18/05/15 18:35	420	444	732	132
18/05/15 18:40	504	480	888	420
18/05/15 18:45	576	408	960	180
18/05/15 18:50	312	384	732	264
18/05/15 18:55	612	552	900	180
18/05/15 19:00	504	672	984	396
18/05/15 19:05	456	432	816	228
18/05/15 19:10	576	516	624	312
18/05/15 19:15	552	540	792	264
18/05/15 19:20	456	624	924	300
18/05/15 19:25	396	588	1020	444
18/05/15 19:30	612	432	864	204
18/05/15 19:35	492	264	612	168
18/05/15 19:40	240	348	540	132
18/05/15 19:45	516	432	756	168
18/05/15 19:50	360	240	816	132
18/05/15 19:55	504	324	576	132
18/05/15 20:00	408	324	600	204
18/05/15 20:05	456	336	456	168
18/05/15 20:10	456	384	456	264
18/05/15 20:15	588	264	636	228
18/05/15 20:20	564	372	648	156
18/05/15 20:25	456	144	408	228
18/05/15 20:30	492	240	528	60
18/05/15 20:35	408	336	564	156
18/05/15 20:40	396	384	636	132
18/05/15 20:45	552	288	420	180
18/05/15 20:50	420	360	504	144
18/05/15 20:55	480	300	444	156
18/05/15 21:00	396	240	432	120
18/05/15 21:05	348	276	504	132
18/05/15 21:10	384	204	468	72
18/05/15 21:15	336	276	480	108
18/05/15 21:20	444	264	480	156

5 MINUTOS	INTENSIDADE			
	PM 0402004	PM 0402007	PM04030031	PM04030032
18/05/15 21:25	432	168	432	144
18/05/15 21:30	288	252	324	108
18/05/15 21:35	420	204	384	60
18/05/15 21:40	468	168	408	108
18/05/15 21:45	420	180	360	120
18/05/15 21:50	300	264	348	84
18/05/15 21:55	336	228	396	96
18/05/15 22:00	480	240	372	60
18/05/15 22:05	384	240	372	156
18/05/15 22:10	468	252	372	156
18/05/15 22:15	324	252	420	132
18/05/15 22:20	372	360	504	84
18/05/15 22:25	360	204	444	144
18/05/15 22:30	408	336	516	132
18/05/15 22:35	336	396	516	228
18/05/15 22:40	276	408	576	168
18/05/15 22:45	444	276	504	228
18/05/15 22:50	180	252	420	108
18/05/15 22:55	252	252	348	96
18/05/15 23:00	264	120	384	60
18/05/15 23:05	192	168	204	84
18/05/15 23:10	252	120	252	108
18/05/15 23:15	144	96	228	72
18/05/15 23:20	228	120	216	72
18/05/15 23:25	156	60	228	36
18/05/15 23:30	132	96	192	48
18/05/15 23:35	168	96	132	24
18/05/15 23:40	132	60	120	24
18/05/15 23:45	168	24	144	36
18/05/15 23:50	216	0	96	12
18/05/15 23:55	96	72	108	24
19/05/15 00:00	132	24	132	36
19/05/15 00:05	72	60	180	0
19/05/15 00:10	84	48	120	0
19/05/15 00:15	156	108	108	36
19/05/15 00:20	120	48	132	12
19/05/15 00:25	120	144	180	36
19/05/15 00:30	48	48	60	48
19/05/15 00:35	96	48	60	24
19/05/15 00:40	120	36	60	12
19/05/15 00:45	96	12	48	0
19/05/15 00:50	72	108	60	48
19/05/15 00:55	72	24	36	0
19/05/15 01:00	96	12	84	0
19/05/15 01:05	36	36	60	12
19/05/15 01:10	84	12	24	0
19/05/15 01:15	24	12	48	0
19/05/15 01:20	48	0	12	0
19/05/15 01:25	84	24	24	12
19/05/15 01:30	108	36	60	0
19/05/15 01:35	96	12	48	12
19/05/15 01:40	84	36	36	12
19/05/15 01:45	12	0	36	0
19/05/15 01:50	48	12	36	12
19/05/15 01:55	0	24	36	0
19/05/15 02:00	48	12	48	0
19/05/15 02:05	36	48	60	0
19/05/15 02:10	24	12	12	12

Sistema de Controle de Tráfego Urbano OPTIMUS

5 MINUTOS	INTENSIDADE			
	PM 0402004	PM 0402007	PM04030031	PM04030032
19/05/15 02:15	60	0	12	0
19/05/15 02:20	12	0	12	0
19/05/15 02:25	48	0	24	0
19/05/15 02:30	24	0	12	0
19/05/15 02:35	24	0	12	0
19/05/15 02:40	36	12	12	0
19/05/15 02:45	12	0	0	0
19/05/15 02:50	60	0	12	0
19/05/15 02:55	12	24	12	12
19/05/15 03:00	0	12	24	0
19/05/15 03:05	48	0	24	0
19/05/15 03:10	48	12	36	0
19/05/15 03:15	72	48	12	24
19/05/15 03:20	48	0	24	12
19/05/15 03:25	12	0	0	0
19/05/15 03:30	24	12	12	0
19/05/15 03:35	36	0	0	0
19/05/15 03:40	0	12	24	12
19/05/15 03:45	24	12	12	0
19/05/15 03:50	12	0	12	0
19/05/15 03:55	24	36	24	12
19/05/15 04:00	48	0	24	0
19/05/15 04:05	12	36	24	0
19/05/15 04:10	0	24	12	12
19/05/15 04:15	60	0	0	0
19/05/15 04:20	60	0	0	0
19/05/15 04:25	36	24	12	12
19/05/15 04:30	24	12	24	12
19/05/15 04:35	36	24	36	0
19/05/15 04:40	36	36	36	0
19/05/15 04:45	24	0	36	0
19/05/15 04:50	72	0	24	0
19/05/15 04:55	72	12	36	0
19/05/15 05:00	24	24	48	12
19/05/15 05:05	84	36	48	0
19/05/15 05:10	48	0	24	0
19/05/15 05:15	108	12	12	12
19/05/15 05:20	72	48	48	12
19/05/15 05:25	36	48	72	24
19/05/15 05:30	36	24	36	12
19/05/15 05:35	60	12	96	0
19/05/15 05:40	132	72	96	24
19/05/15 05:45	132	84	84	48
19/05/15 05:50	216	36	192	36
19/05/15 05:55	72	108	144	12
19/05/15 06:00	132	60	276	24
19/05/15 06:05	228	48	240	24
19/05/15 06:10	264	132	336	36
19/05/15 06:15	240	108	276	48
19/05/15 06:20	312	144	468	48
19/05/15 06:25	348	192	360	96
19/05/15 06:30	324	168	492	108
19/05/15 06:35	444	216	660	60
19/05/15 06:40	720	360	552	132
19/05/15 06:45	720	312	660	144
19/05/15 06:50	780	396	864	204
19/05/15 06:55	1008	624	924	204
19/05/15 07:00	684	516	852	264

Sistema de Controle de Tráfego Urbano OPTIMUS

5 MINUTOS	INTENSIDADE			
	PM 0402004	PM 0402007	PM04030031	PM04030032
19/05/15 07:05	960	636	840	240
19/05/15 07:10	948	660	948	336
19/05/15 07:15	972	624	792	168
19/05/15 07:20	864	528	912	240
19/05/15 07:25	924	492	840	216
19/05/15 07:30	972	348	876	120
19/05/15 07:35	948	312	804	120
19/05/15 07:40	972	528	720	156
19/05/15 07:45	1056	384	984	132
19/05/15 07:50	888	468	828	156
19/05/15 07:55	1020	408	768	156
19/05/15 08:00	888	456	840	192
19/05/15 08:05	804	504	744	204
19/05/15 08:10	984	264	780	168
19/05/15 08:15	888	312	744	144
19/05/15 08:20	852	384	852	216
19/05/15 08:25	816	252	612	168
19/05/15 08:30	900	144	468	36
19/05/15 08:35	672	240	624	156
19/05/15 08:40	936	360	648	180
19/05/15 08:45	696	216	720	228
19/05/15 08:50	732	312	612	180
19/05/15 08:55	696	336	780	216
19/05/15 09:00	732	264	972	120
19/05/15 09:05	924	360	648	108
19/05/15 09:10	852	276	792	168
19/05/15 09:15	756	264	708	120
19/05/15 09:20	540	276	864	180
19/05/15 09:25	912	264	696	120
19/05/15 09:30	744	276	780	144
19/05/15 09:35	864	336	744	120
19/05/15 09:40	612	348	576	228
19/05/15 09:45	660	324	660	144
19/05/15 09:50	792	228	648	132
19/05/15 09:55	816	264	780	192
19/05/15 10:00	684	336	660	204
19/05/15 10:05	732	204	636	132
19/05/15 10:10	540	360	684	132
19/05/15 10:15	624	348	744	180
19/05/15 10:20	708	288	708	168
19/05/15 10:25	696	360	720	132
19/05/15 10:30	564	600	780	228
19/05/15 10:35	804	540	816	156
19/05/15 10:40	744	576	936	216
19/05/15 10:45	708	588	720	372
19/05/15 10:50	540	348	768	180
19/05/15 10:55	624	432	780	204
19/05/15 11:00	504	360	588	156
19/05/15 11:05	900	516	876	288
19/05/15 11:10	636	540	720	216
19/05/15 11:15	636	540	828	240
19/05/15 11:20	648	576	912	264
19/05/15 11:25	564	456	564	228
19/05/15 11:30	504	456	780	240
19/05/15 11:35	684	504	792	180
19/05/15 11:40	732	372	696	192
19/05/15 11:45	720	768	660	312
19/05/15 11:50	876	768	912	336

Sistema de Controle de Tráfego Urbano OPTIMUS

5 MINUTOS	INTENSIDADE			
	PM 0402004	PM 0402007	PM04030031	PM04030032
19/05/15 11:55	708	420	1008	240
19/05/15 12:00	648	588	984	252
19/05/15 12:05	732	660	972	288
19/05/15 12:10	612	492	852	276
19/05/15 12:15	660	396	804	180
19/05/15 12:20	576	516	720	240
19/05/15 12:25	528	492	720	240
19/05/15 12:30	660	564	756	288
19/05/15 12:35	876	444	936	216
19/05/15 12:40	624	408	840	132
19/05/15 12:45	684	540	828	192
19/05/15 12:50	660	564	888	204
19/05/15 12:55	588	516	852	264
19/05/15 13:00	864	672	888	324
19/05/15 13:05	552	528	900	252
19/05/15 13:10	900	792	852	216
19/05/15 13:15	600	708	972	300
19/05/15 13:20	684	792	1068	216
19/05/15 13:25	912	600	780	156
19/05/15 13:30	936	636	948	276
19/05/15 13:35	744	432	660	180
19/05/15 13:40	756	420	840	120
19/05/15 13:45	780	600	708	180
19/05/15 13:50	600	576	912	264
19/05/15 13:55	852	648	900	288
19/05/15 14:00	912	456	756	192
19/05/15 14:05	780	504	852	312
19/05/15 14:10	744	456	816	240
19/05/15 14:15	936	432	852	180
19/05/15 14:20	804	504	864	228
19/05/15 14:25	684	636	948	228
19/05/15 14:30	828	444	828	192
19/05/15 14:35	624	588	984	240
19/05/15 14:40	912	624	864	216
19/05/15 14:45	672	264	888	180
19/05/15 14:50	576	576	756	264
19/05/15 14:55	828	516	996	228
19/05/15 15:00	720	324	768	144
19/05/15 15:05	804	348	744	216
19/05/15 15:10	696	420	888	204
19/05/15 15:15	624	480	864	180
19/05/15 15:20	504	360	660	216
19/05/15 15:25	828	516	840	228
19/05/15 15:30	852	372	744	228
19/05/15 15:35	588	372	660	156
19/05/15 15:40	768	588	744	156
19/05/15 15:45	924	480	804	216
19/05/15 15:50	828	468	756	192
19/05/15 15:55	588	648	840	252
19/05/15 16:00	768	384	828	168
19/05/15 16:05	648	444	792	288
19/05/15 16:10	600	732	960	264
19/05/15 16:15	864	432	792	144
19/05/15 16:20	720	372	684	216
19/05/15 16:25	720	564	804	264
19/05/15 16:30	696	528	912	156
19/05/15 16:35	720	504	768	252
19/05/15 16:40	528	576	804	288

Sistema de Controle de Tráfego Urbano OPTIMUS

5 MINUTOS	INTENSIDADE			
	PM 0402004	PM 0402007	PM 04030031	PM 04030032
19/05/15 16:45	864	492	780	228
19/05/15 16:50	708	312	780	240
19/05/15 16:55	672	528	672	228
19/05/15 17:00	576	552	852	312
19/05/15 17:05	528	660	804	288
19/05/15 17:10	540	672	852	324
19/05/15 17:15	588	480	720	276
19/05/15 17:20	360	288	528	156
19/05/15 17:25	240	360	528	132
19/05/15 17:30	444	624	552	168
19/05/15 17:35	384	444	696	144
19/05/15 17:40	456	504	648	132
19/05/15 17:45	492	396	648	240
19/05/15 17:50	708	708	648	228
19/05/15 17:55	708	348	1128	312
19/05/15 18:00	684	816	1068	252
19/05/15 18:05	720	768	1080	180
19/05/15 18:10	396	504	936	372
19/05/15 18:15	360	552	948	348
19/05/15 18:20	672	516	576	180
19/05/15 18:25	456	528	756	288
19/05/15 18:30	480	444	756	348
19/05/15 18:35	408	456	888	264
19/05/15 18:40	324	528	780	228
19/05/15 18:45	348	432	528	216
19/05/15 18:50	456	540	720	216
19/05/15 18:55	528	600	984	288
19/05/15 19:00	456	444	840	192
19/05/15 19:05	360	840	984	336
19/05/15 19:10	552	612	1080	312
19/05/15 19:15	540	492	972	288
19/05/15 19:20	528	528	936	192
19/05/15 19:25	540	420	936	264
19/05/15 19:30	480	384	636	252
19/05/15 19:35	684	312	792	156
19/05/15 19:40	420	264	768	192
19/05/15 19:45	408	480	612	264
19/05/15 19:50	336	324	672	240
19/05/15 19:55	420	276	600	132
19/05/15 20:00	528	348	828	216
19/05/15 20:05	444	300	660	132
19/05/15 20:10	480	396	732	108
19/05/15 20:15	480	180	576	132
19/05/15 20:20	552	252	336	60
19/05/15 20:25	480	396	672	204
19/05/15 20:30	516	216	420	156
19/05/15 20:35	492	348	732	84
19/05/15 20:40	396	336	504	228
19/05/15 20:45	384	300	360	144
19/05/15 20:50	360	312	612	96
19/05/15 20:55	468	204	564	156
19/05/15 21:00	504	408	456	180
19/05/15 21:05	324	252	432	168
19/05/15 21:10	504	348	480	144
19/05/15 21:15	300	288	600	108
19/05/15 21:20	540	252	504	204
19/05/15 21:25	504	336	444	192
19/05/15 21:30	324	252	432	144

Sistema de Controle de Tráfego Urbano OPTIMUS

5 MINUTOS	INTENSIDADE			
	PM 0402004	PM 0402007	PM04030031	PM04030032
19/05/15 21:35	276	288	588	108
19/05/15 21:40	384	240	492	156
19/05/15 21:45	348	252	396	132
19/05/15 21:50	360	252	552	180
19/05/15 21:55	396	228	528	120
19/05/15 22:00	504	264	408	108
19/05/15 22:05	336	312	552	144
19/05/15 22:10	528	312	444	180
19/05/15 22:15	516	276	492	132
19/05/15 22:20	324	288	396	84
19/05/15 22:25	276	240	444	132
19/05/15 22:30	528	348	600	120
19/05/15 22:35	456	240	516	60
19/05/15 22:40	432	384	444	204
19/05/15 22:45	372	204	540	168
19/05/15 22:50	324	216	348	108
19/05/15 22:55	324	204	456	60
19/05/15 23:00	468	156	276	96
19/05/15 23:05	240	168	396	48
19/05/15 23:10	240	144	180	108
19/05/15 23:15	192	144	204	72
19/05/15 23:20	264	60	204	24
19/05/15 23:25	192	156	288	24
19/05/15 23:30	204	60	240	36
19/05/15 23:35	144	84	192	12
19/05/15 23:40	144	120	180	48
19/05/15 23:45	228	36	120	12
19/05/15 23:50	228	36	204	48
19/05/15 23:55	168	96	108	24
20/05/15 00:00	312	36	204	72
20/05/15 00:05	156	0	108	12
20/05/15 00:10	144	36	204	12
20/05/15 00:15	216	60	72	12
20/05/15 00:20	120	48	96	36
20/05/15 00:25	120	84	144	36
20/05/15 00:30	108	144	156	36
20/05/15 00:35	108	72	120	36
20/05/15 00:40	72	48	60	0
20/05/15 00:45	84	108	108	36
20/05/15 00:50	120	96	48	12
20/05/15 00:55	108	24	84	12
20/05/15 01:00	84	36	84	12
20/05/15 01:05	48	60	60	0
20/05/15 01:10	84	12	84	0
20/05/15 01:15	72	48	96	36
20/05/15 01:20	96	48	60	0
20/05/15 01:25	36	12	72	12
20/05/15 01:30	48	0	24	0
20/05/15 01:35	72	0	36	0
20/05/15 01:40	36	36	36	24
20/05/15 01:45	24	12	48	12
20/05/15 01:50	48	12	60	0
20/05/15 01:55	24	24	12	12
20/05/15 02:00	132	12	72	0
20/05/15 02:05	48	12	36	0
20/05/15 02:10	24	24	48	0
20/05/15 02:15	24	0	12	0
20/05/15 02:20	60	0	12	0

Sistema de Controle de Tráfego Urbano OPTIMUS

5 MINUTOS	INTENSIDADE			
	PM 0402004	PM 0402007	PM04030031	PM04030032
20/05/15 02:25	24	0	24	0
20/05/15 02:30	48	12	24	0
20/05/15 02:35	24	0	36	0
20/05/15 02:40	48	12	48	0
20/05/15 02:45	72	12	48	0
20/05/15 02:50	36	0	36	0
20/05/15 02:55	48	12	0	0
20/05/15 03:00	24	24	24	0
20/05/15 03:05	48	0	36	0
20/05/15 03:10	24	0	72	0
20/05/15 03:15	48	12	24	12
20/05/15 03:20	84	12	24	12
20/05/15 03:25	12	24	12	24
20/05/15 03:30	48	24	36	0
20/05/15 03:35	0	0	36	0
20/05/15 03:40	0	0	12	0
20/05/15 03:45	24	0	0	0
20/05/15 03:50	24	0	0	0
20/05/15 03:55	36	0	0	0
20/05/15 04:00	24	0	12	0
20/05/15 04:05	48	24	12	12
20/05/15 04:10	24	24	0	12
20/05/15 04:15	48	24	12	12
20/05/15 04:20	24	24	24	0
20/05/15 04:25	12	0	12	0
20/05/15 04:30	36	24	24	0
20/05/15 04:35	48	0	36	0
20/05/15 04:40	36	36	24	0
20/05/15 04:45	12	24	24	12
20/05/15 04:50	60	12	36	12
20/05/15 04:55	12	24	36	0
20/05/15 05:00	48	24	60	24
20/05/15 05:05	60	24	36	12
20/05/15 05:10	108	24	24	0
20/05/15 05:15	96	24	36	0
20/05/15 05:20	72	12	48	0
20/05/15 05:25	48	36	48	24
20/05/15 05:30	60	60	48	0
20/05/15 05:35	60	48	48	24
20/05/15 05:40	48	24	84	12
20/05/15 05:45	60	48	144	24
20/05/15 05:50	228	84	156	24
20/05/15 05:55	144	144	156	72
20/05/15 06:00	156	84	228	0
20/05/15 06:05	216	96	192	48
20/05/15 06:10	192	144	264	36
20/05/15 06:15	156	120	264	60
20/05/15 06:20	276	72	336	36
20/05/15 06:25	324	168	288	60
20/05/15 06:30	408	168	720	120
20/05/15 06:35	444	216	516	48
20/05/15 06:40	528	276	672	156
20/05/15 06:45	768	264	708	180
20/05/15 06:50	876	396	924	120
20/05/15 06:55	984	516	744	156
20/05/15 07:00	828	684	900	276
20/05/15 07:05	1044	528	720	240
20/05/15 07:10	804	672	924	288

Sistema de Controle de Tráfego Urbano OPTIMUS

5 MINUTOS	INTENSIDADE			
	PM 0402004	PM 0402007	PM 04030031	PM 04030032
20/05/15 07:15	1200	720	984	288
20/05/15 07:20	864	588	900	156
20/05/15 07:25	1092	432	1044	204
20/05/15 07:30	1020	264	720	96
20/05/15 07:35	864	408	780	156
20/05/15 07:40	1128	576	828	108
20/05/15 07:45	1044	324	756	144
20/05/15 07:50	888	288	600	156
20/05/15 07:55	804	336	792	144
20/05/15 08:00	1008	420	864	120
20/05/15 08:05	792	456	732	192
20/05/15 08:10	768	492	708	144
20/05/15 08:15	720	288	624	120
20/05/15 08:20	972	492	684	168
20/05/15 08:25	888	432	636	240
20/05/15 08:30	984	312	636	156
20/05/15 08:35	828	396	636	84
20/05/15 08:40	732	756	720	204
20/05/15 08:45	816	480	732	216
20/05/15 08:50	756	360	636	192
20/05/15 08:55	996	492	636	132
20/05/15 09:00	924	432	852	204
20/05/15 09:05	972	432	684	228
20/05/15 09:10	1080	468	756	144
20/05/15 09:15	900	300	804	120
20/05/15 09:20	936	456	744	156
20/05/15 09:25	636	360	660	168
20/05/15 09:30	660	516	768	180
20/05/15 09:35	804	480	732	264
20/05/15 09:40	744	576	744	180
20/05/15 09:45	672	504	948	204
20/05/15 09:50	828	420	816	156
20/05/15 09:55	696	540	984	216
20/05/15 10:00	936	312	660	156
20/05/15 10:05	756	180	852	120
20/05/15 10:10	720	396	732	204
20/05/15 10:15	756	444	708	192
20/05/15 10:20	552	528	720	204
20/05/15 10:25	744	432	732	240
20/05/15 10:30	828	588	828	216
20/05/15 10:35	708	564	888	312
20/05/15 10:40	660	564	900	288
20/05/15 10:45	672	456	816	216
20/05/15 10:50	804	468	600	132
20/05/15 10:55	564	528	852	240
20/05/15 11:00	864	588	780	228
20/05/15 11:05	720	480	624	300
20/05/15 11:10	840	300	744	228
20/05/15 11:15	684	492	888	288
20/05/15 11:20	696	564	828	240
20/05/15 11:25	816	672	948	276
20/05/15 11:30	588	660	744	204
20/05/15 11:35	600	684	768	288
20/05/15 11:40	612	456	732	192
20/05/15 11:45	708	756	804	216
20/05/15 11:50	732	792	972	324
20/05/15 11:55	768	696	876	264
20/05/15 12:00	648	624	1140	360

Sistema de Controle de Tráfego Urbano OPTIMUS

5 MINUTOS	INTENSIDADE			
	PM 0402004	PM 0402007	PM 04030031	PM 04030032
20/05/15 12:05	528	348	912	204
20/05/15 12:10	468	636	852	324
20/05/15 12:15	624	600	768	204
20/05/15 12:20	708	600	900	240
20/05/15 12:25	696	576	876	276
20/05/15 12:30	780	660	936	216
20/05/15 12:35	600	576	864	204
20/05/15 12:40	588	528	768	192
20/05/15 12:45	756	672	900	336
20/05/15 12:50	636	564	828	276
20/05/15 12:55	828	696	984	372
20/05/15 13:00	492	696	864	192
20/05/15 13:05	864	828	912	360
20/05/15 13:10	672	420	912	240
20/05/15 13:15	840	576	1020	144
20/05/15 13:20	756	672	1020	180
20/05/15 13:25	636	588	984	276
20/05/15 13:30	744	312	804	216
20/05/15 13:35	900	624	828	264
20/05/15 13:40	816	540	912	156
20/05/15 13:45	852	648	804	276
20/05/15 13:50	648	708	912	312
20/05/15 13:55	888	516	948	216
20/05/15 14:00	972	324	744	216
20/05/15 14:05	768	528	876	144
20/05/15 14:10	720	372	804	204
20/05/15 14:15	780	504	876	180
20/05/15 14:20	936	636	996	240
20/05/15 14:25	732	480	744	240
20/05/15 14:30	900	516	852	336
20/05/15 14:35	828	384	900	216
20/05/15 14:40	684	684	912	288
20/05/15 14:45	720	420	804	228
20/05/15 14:50	672	468	768	240
20/05/15 14:55	876	624	864	252
20/05/15 15:00	912	348	816	192
20/05/15 15:05	660	492	780	168
20/05/15 15:10	612	576	816	192
20/05/15 15:15	588	336	720	144
20/05/15 15:20	696	540	660	240
20/05/15 15:25	744	600	768	216
20/05/15 15:30	888	516	924	264
20/05/15 15:35	636	540	900	264
20/05/15 15:40	552	408	588	192
20/05/15 15:45	672	480	912	300
20/05/15 15:50	756	600	816	216
20/05/15 15:55	804	468	624	156
20/05/15 16:00	768	456	876	216
20/05/15 16:05	660	360	720	156
20/05/15 16:10	636	660	756	204
20/05/15 16:15	816	516	648	96
20/05/15 16:20	648	348	696	192
20/05/15 16:25	636	408	732	156
20/05/15 16:30	792	456	936	252
20/05/15 16:35	984	324	624	252
20/05/15 16:40	756	348	732	180
20/05/15 16:45	792	540	948	180
20/05/15 16:50	708	444	720	180

Sistema de Controle de Tráfego Urbano OPTIMUS

5 MINUTOS	INTENSIDADE			
	PM 0402004	PM 0402007	PM 04030031	PM 04030032
20/05/15 16:55	744	480	828	360
20/05/15 17:00	624	468	804	192
20/05/15 17:05	660	564	804	300
20/05/15 17:10	720	576	732	228
20/05/15 17:15	696	588	816	288
20/05/15 17:20	516	564	828	276
20/05/15 17:25	600	612	972	312
20/05/15 17:30	588	576	876	372
20/05/15 17:35	600	480	972	204
20/05/15 17:40	588	972	1056	384
20/05/15 17:45	408	696	948	348
20/05/15 17:50	588	660	780	300
20/05/15 17:55	648	504	768	228
20/05/15 18:00	468	624	840	288
20/05/15 18:05	504	672	936	324
20/05/15 18:10	516	540	972	216
20/05/15 18:15	528	348	684	288
20/05/15 18:20	372	528	900	336
20/05/15 18:25	420	444	744	156
20/05/15 18:30	348	672	912	276
20/05/15 18:35	624	588	852	348
20/05/15 18:40	408	588	912	264
20/05/15 18:45	528	504	792	336
20/05/15 18:50	600	564	840	252
20/05/15 18:55	480	588	828	276
20/05/15 19:00	456	648	792	264
20/05/15 19:05	468	360	960	288
20/05/15 19:10	540	480	816	312
20/05/15 19:15	624	456	804	192
20/05/15 19:20	456	576	876	204
20/05/15 19:25	624	516	624	228
20/05/15 19:30	576	348	732	132
20/05/15 19:35	432	312	792	192
20/05/15 19:40	564	288	552	168
20/05/15 19:45	420	420	696	192
20/05/15 19:50	396	300	552	252
20/05/15 19:55	552	492	684	252
20/05/15 20:00	348	420	756	180
20/05/15 20:05	432	300	552	252
20/05/15 20:10	516	300	600	144
20/05/15 20:15	504	192	540	132
20/05/15 20:20	420	252	504	156
20/05/15 20:25	420	252	588	180
20/05/15 20:30	468	348	468	120
20/05/15 20:35	300	204	516	120
20/05/15 20:40	432	312	540	168
20/05/15 20:45	444	348	504	96
20/05/15 20:50	540	300	648	132
20/05/15 20:55	516	312	564	132
20/05/15 21:00	360	360	564	132
20/05/15 21:05	492	252	492	156
20/05/15 21:10	372	384	444	156
20/05/15 21:15	540	420	564	156
20/05/15 21:20	468	276	480	180
20/05/15 21:25	420	372	444	96
20/05/15 21:30	480	228	516	144
20/05/15 21:35	348	216	444	96
20/05/15 21:40	492	180	360	96

Sistema de Controle de Tráfego Urbano OPTIMUS

5 MINUTOS	INTENSIDADE			
	PM 0402004	PM 0402007	PM 04030031	PM 04030032
20/05/15 21:45	252	264	540	180
20/05/15 21:50	372	240	348	72
20/05/15 21:55	468	204	528	72
20/05/15 22:00	420	240	336	120
20/05/15 22:05	396	276	456	132
20/05/15 22:10	420	240	540	120
20/05/15 22:15	504	252	408	180
20/05/15 22:20	360	312	300	120
20/05/15 22:25	312	252	504	132
20/05/15 22:30	432	408	432	192
20/05/15 22:35	468	276	576	120
20/05/15 22:40	360	432	564	204
20/05/15 22:45	384	312	564	168
20/05/15 22:50	156	240	528	156
20/05/15 22:55	240	204	336	96
20/05/15 23:00	300	216	372	60
20/05/15 23:05	156	132	252	72
20/05/15 23:10	300	144	240	72
20/05/15 23:15	192	108	252	84
20/05/15 23:20	144	156	252	48
20/05/15 23:25	180	168	180	24
20/05/15 23:30	216	60	300	60
20/05/15 23:35	228	36	180	36
20/05/15 23:40	180	24	204	12
20/05/15 23:45	168	48	84	24
20/05/15 23:50	192	24	144	0
20/05/15 23:55	216	108	132	24
21/05/15 00:00	180	72	108	60
21/05/15 00:05	216	96	132	24
21/05/15 00:10	120	108	120	0
21/05/15 00:15	120	72	132	48
21/05/15 00:20	156	48	192	0
21/05/15 00:25	120	48	84	12
21/05/15 00:30	72	48	96	24
21/05/15 00:35	84	24	120	0
21/05/15 00:40	84	108	156	12
21/05/15 00:45	156	72	84	24
21/05/15 00:50	84	72	108	24
21/05/15 00:55	48	0	96	12
21/05/15 01:00	120	36	96	12
21/05/15 01:05	84	0	108	0
21/05/15 01:10	120	0	96	0
21/05/15 01:15	108	60	96	24
21/05/15 01:20	144	24	12	12
21/05/15 01:25	72	24	48	24
21/05/15 01:30	84	48	36	12
21/05/15 01:35	60	12	48	36
21/05/15 01:40	36	24	48	0
21/05/15 01:45	84	12	36	12
21/05/15 01:50	72	36	60	0
21/05/15 01:55	24	12	12	0
21/05/15 02:00	48	24	84	0
21/05/15 02:05	48	24	60	0
21/05/15 02:10	24	12	48	0
21/05/15 02:15	24	0	48	0
21/05/15 02:20	36	12	48	0
21/05/15 02:25	24	0	12	0
21/05/15 02:30	120	0	36	0

Sistema de Controle de Tráfego Urbano OPTIMUS

5 MINUTOS	INTENSIDADE			
	PM 0402004	PM 0402007	PM 04030031	PM 04030032
21/05/15 02:35	48	24	48	0
21/05/15 02:40	60	48	72	0
21/05/15 02:45	12	0	36	0
21/05/15 02:50	24	0	36	0
21/05/15 02:55	36	12	36	0
21/05/15 03:00	24	12	24	0
21/05/15 03:05	48	0	24	0
21/05/15 03:10	36	0	12	0
21/05/15 03:15	24	12	12	24
21/05/15 03:20	36	60	60	12
21/05/15 03:25	24	12	48	0
21/05/15 03:30	36	24	36	0
21/05/15 03:35	36	0	24	0
21/05/15 03:40	24	0	36	0
21/05/15 03:45	24	12	48	0
21/05/15 03:50	36	12	0	0
21/05/15 03:55	48	12	36	0
21/05/15 04:00	60	0	0	0
21/05/15 04:05	24	0	0	0
21/05/15 04:10	12	0	0	0
21/05/15 04:15	12	0	12	0
21/05/15 04:20	96	12	24	0
21/05/15 04:25	48	12	24	0
21/05/15 04:30	24	24	36	24
21/05/15 04:35	48	36	0	12
21/05/15 04:40	48	0	0	0
21/05/15 04:45	36	48	48	12
21/05/15 04:50	36	24	60	0
21/05/15 04:55	108	12	36	0
21/05/15 05:00	36	48	48	36
21/05/15 05:05	120	0	24	24
21/05/15 05:10	48	24	72	0
21/05/15 05:15	60	12	12	0
21/05/15 05:20	36	12	48	0
21/05/15 05:25	48	96	120	36
21/05/15 05:30	144	48	84	12
21/05/15 05:35	84	12	72	0
21/05/15 05:40	120	72	120	36
21/05/15 05:45	108	60	60	48
21/05/15 05:50	204	60	120	24
21/05/15 05:55	180	72	156	12
21/05/15 06:00	108	84	240	24
21/05/15 06:05	168	120	156	48
21/05/15 06:10	204	84	264	48
21/05/15 06:15	252	48	324	24
21/05/15 06:20	192	132	336	60
21/05/15 06:25	444	192	372	72
21/05/15 06:30	360	180	516	96
21/05/15 06:35	504	192	624	84
21/05/15 06:40	540	336	768	96
21/05/15 06:45	816	336	588	108
21/05/15 06:50	828	480	696	252
21/05/15 06:55	852	468	732	240
21/05/15 07:00	900	624	996	300
21/05/15 07:05	888	480	816	168
21/05/15 07:10	708	540	864	216
21/05/15 07:15	768	444	684	192
21/05/15 07:20	684	540	960	168

Sistema de Controle de Tráfego Urbano OPTIMUS

5 MINUTOS	INTENSIDADE			
	PM 0402004	PM 0402007	PM04030031	PM04030032
21/05/15 07:25	948	444	864	180
21/05/15 07:30	912	600	696	108
21/05/15 07:35	948	264	684	180
21/05/15 07:40	1032	540	900	336
21/05/15 07:45	948	576	828	96
21/05/15 07:50	1140	504	804	132
21/05/15 07:55	1128	600	804	204
21/05/15 08:00	912	504	840	144
21/05/15 08:05	1164	552	900	252
21/05/15 08:10	804	384	948	132
21/05/15 08:15	948	384	780	120
21/05/15 08:20	1032	444	852	168
21/05/15 08:25	888	300	684	132
21/05/15 08:30	1056	396	852	144
21/05/15 08:35	948	552	852	228
21/05/15 08:40	960	348	720	216
21/05/15 08:45	1020	504	744	204
21/05/15 08:50	984	336	768	216
21/05/15 08:55	996	420	876	156
21/05/15 09:00	1152	408	732	168
21/05/15 09:05	1044	396	780	168
21/05/15 09:10	876	492	804	180
21/05/15 09:15	984	432	780	204
21/05/15 09:20	864	372	840	144
21/05/15 09:25	804	324	696	120
21/05/15 09:30	828	444	684	180
21/05/15 09:35	732	420	636	144
21/05/15 09:40	612	408	708	192
21/05/15 09:45	792	540	756	228
21/05/15 09:50	924	588	636	228
21/05/15 09:55	696	360	744	156
21/05/15 10:00	792	288	660	108
21/05/15 10:05	816	396	528	192
21/05/15 10:10	504	264	684	120
21/05/15 10:15	696	468	744	240
21/05/15 10:20	624	540	756	252
21/05/15 10:25	720	444	864	204
21/05/15 10:30	792	408	816	228
21/05/15 10:35	684	588	876	192
21/05/15 10:40	564	372	792	192
21/05/15 10:45	684	480	948	216
21/05/15 10:50	792	552	864	288
21/05/15 10:55	804	564	900	192
21/05/15 11:00	684	504	804	204
21/05/15 11:05	780	504	828	168
21/05/15 11:10	552	468	672	180
21/05/15 11:15	912	432	720	192
21/05/15 11:20	660	636	852	204
21/05/15 11:25	732	504	1008	192
21/05/15 11:30	696	588	792	324
21/05/15 11:35	804	528	780	228
21/05/15 11:40	936	372	792	240
21/05/15 11:45	876	660	864	216
21/05/15 11:50	816	960	852	240
21/05/15 11:55	576	780	1104	276
21/05/15 12:00	564	600	1032	312
21/05/15 12:05	720	624	876	144
21/05/15 12:10	672	540	864	300

Sistema de Controle de Tráfego Urbano OPTIMUS

5 MINUTOS	INTENSIDADE			
	PM 0402004	PM 0402007	PM 04030031	PM 04030032
21/05/15 12:15	588	480	768	252
21/05/15 12:20	552	456	708	192
21/05/15 12:25	552	624	816	300
21/05/15 12:30	708	456	732	264
21/05/15 12:35	792	504	660	240
21/05/15 12:40	612	420	1044	168
21/05/15 12:45	780	444	864	216
21/05/15 12:50	708	624	840	312
21/05/15 12:55	768	720	984	264
21/05/15 13:00	624	912	876	300
21/05/15 13:05	816	600	972	276
21/05/15 13:10	852	504	1032	180
21/05/15 13:15	900	540	888	240
21/05/15 13:20	840	492	816	408
21/05/15 13:25	852	576	828	276
21/05/15 13:30	792	528	900	192
21/05/15 13:35	768	612	840	156
21/05/15 13:40	864	648	912	276
21/05/15 13:45	780	456	768	276
21/05/15 13:50	708	708	900	192
21/05/15 13:55	720	552	840	372
21/05/15 14:00	912	588	696	216
21/05/15 14:05	720	636	912	240
21/05/15 14:10	792	516	792	144
21/05/15 14:15	720	600	852	252
21/05/15 14:20	792	336	768	144
21/05/15 14:25	888	552	756	228
21/05/15 14:30	696	684	912	240
21/05/15 14:35	876	576	792	216
21/05/15 14:40	768	492	840	240
21/05/15 14:45	840	792	972	372
21/05/15 14:50	636	468	960	384
21/05/15 14:55	828	576	960	180
21/05/15 15:00	744	516	804	276
21/05/15 15:05	816	468	864	156
21/05/15 15:10	744	720	876	264
21/05/15 15:15	636	408	924	228
21/05/15 15:20	612	504	840	228
21/05/15 15:25	696	648	912	252
21/05/15 15:30	768	600	696	216
21/05/15 15:35	732	492	1080	192
21/05/15 15:40	684	528	552	180
21/05/15 15:45	672	888	840	312
21/05/15 15:50	732	576	768	228
21/05/15 15:55	840	444	1068	264
21/05/15 16:00	492	468	792	204
21/05/15 16:05	684	192	612	144
21/05/15 16:10	720	312	636	156
21/05/15 16:15	756	408	828	264
21/05/15 16:20	612	372	924	180
21/05/15 16:25	612	720	840	324
21/05/15 16:30	576	552	828	276
21/05/15 16:35	876	492	840	204
21/05/15 16:40	612	612	912	240
21/05/15 16:45	660	540	816	300
21/05/15 16:50	636	600	912	192
21/05/15 16:55	804	408	864	168
21/05/15 17:00	480	444	624	168

Sistema de Controle de Tráfego Urbano OPTIMUS

5 MINUTOS	INTENSIDADE			
	PM 0402004	PM 0402007	PM04030031	PM04030032
21/05/15 17:05	636	612	900	228
21/05/15 17:10	792	552	936	300
21/05/15 17:15	684	612	828	312
21/05/15 17:20	468	396	492	228
21/05/15 17:25	180	468	564	240
21/05/15 17:30	192	552	948	192
21/05/15 17:35	612	564	960	132
21/05/15 17:40	384	636	780	120
21/05/15 17:45	348	456	600	216
21/05/15 17:50	456	240	432	216
21/05/15 17:55	396	492	864	132
21/05/15 18:00	492	492	984	276
21/05/15 18:05	336	564	768	240
21/05/15 18:10	312	312	636	192
21/05/15 18:15	360	792	1164	228
21/05/15 18:20	468	420	744	192
21/05/15 18:25	360	552	912	72
21/05/15 18:30	432	276	648	276
21/05/15 18:35	252	252	708	96
21/05/15 18:40	348	912	684	252
21/05/15 18:45	192	696	912	276
21/05/15 18:50	504	564	1008	252
21/05/15 18:55	396	600	1056	228
21/05/15 19:00	408	744	1092	348
21/05/15 19:05	564	672	936	228
21/05/15 19:10	600	816	1140	300
21/05/15 19:15	744	492	996	348
21/05/15 19:20	612	564	1116	324
21/05/15 19:25	480	588	996	312
21/05/15 19:30	684	492	984	336
21/05/15 19:35	444	528	756	348
21/05/15 19:40	480	528	768	324
21/05/15 19:45	420	444	696	204
21/05/15 19:50	420	372	648	168
21/05/15 19:55	408	384	600	252
21/05/15 20:00	636	456	588	228
21/05/15 20:05	420	312	684	156
21/05/15 20:10	420	384	816	252
21/05/15 20:15	684	456	660	228
21/05/15 20:20	540	360	588	192
21/05/15 20:25	492	264	492	144
21/05/15 20:30	468	252	492	108
21/05/15 20:35	576	336	624	180
21/05/15 20:40	384	456	624	192
21/05/15 20:45	372	348	540	120
21/05/15 20:50	504	216	588	168
21/05/15 20:55	396	336	504	240
21/05/15 21:00	408	252	456	156
21/05/15 21:05	480	324	540	156
21/05/15 21:10	564	228	480	168
21/05/15 21:15	528	276	552	180
21/05/15 21:20	528	276	576	168
21/05/15 21:25	432	336	564	84
21/05/15 21:30	456	276	612	156
21/05/15 21:35	384	276	600	192
21/05/15 21:40	264	396	732	180
21/05/15 21:45	480	228	540	96
21/05/15 21:50	396	240	516	96

Sistema de Controle de Tráfego Urbano OPTIMUS

5 MINUTOS	INTENSIDADE			
	PM 0402004	PM 0402007	PM 04030031	PM 04030032
21/05/15 21:55	324	228	492	84
21/05/15 22:00	420	300	480	108
21/05/15 22:05	384	324	576	180
21/05/15 22:10	432	288	372	192
21/05/15 22:15	540	264	492	60
21/05/15 22:20	492	240	528	204
21/05/15 22:25	372	348	552	192
21/05/15 22:30	492	336	540	156
21/05/15 22:35	432	252	288	156
21/05/15 22:40	300	420	444	180
21/05/15 22:45	276	360	552	204
21/05/15 22:50	264	192	432	72
21/05/15 22:55	432	156	408	96
21/05/15 23:00	300	144	384	84
21/05/15 23:05	180	120	192	72
21/05/15 23:10	216	96	276	24
21/05/15 23:15	348	120	276	72
21/05/15 23:20	192	108	168	36
21/05/15 23:25	288	132	228	60
21/05/15 23:30	144	144	192	48
21/05/15 23:35	144	48	180	24
21/05/15 23:40	168	96	156	72
21/05/15 23:45	168	132	180	72
21/05/15 23:50	228	60	120	24
21/05/15 23:55	168	84	216	24
22/05/15 00:00	228	48	180	24
22/05/15 00:05	216	108	204	36
22/05/15 00:10	216	168	300	36
22/05/15 00:15	204	72	228	24
22/05/15 00:20	372	120	228	60
22/05/15 00:25	264	132	204	60
22/05/15 00:30	240	252	324	132
22/05/15 00:35	180	108	108	12
22/05/15 00:40	132	108	240	36
22/05/15 00:45	180	108	168	24
22/05/15 00:50	168	84	96	36
22/05/15 00:55	168	108	168	36
22/05/15 01:00	120	48	132	0
22/05/15 01:05	204	84	168	24
22/05/15 01:10	228	72	108	36
22/05/15 01:15	144	48	72	24
22/05/15 01:20	144	24	96	12
22/05/15 01:25	96	84	168	12
22/05/15 01:30	84	48	120	12
22/05/15 01:35	84	48	48	12
22/05/15 01:40	60	12	144	0
22/05/15 01:45	72	12	120	12
22/05/15 01:50	108	24	72	0
22/05/15 01:55	72	60	72	0
22/05/15 02:00	108	36	72	12
22/05/15 02:05	108	36	72	12
22/05/15 02:10	48	24	60	0
22/05/15 02:15	12	24	60	0
22/05/15 02:20	48	12	60	0
22/05/15 02:25	72	0	36	0
22/05/15 02:30	96	36	60	0
22/05/15 02:35	36	0	96	0
22/05/15 02:40	60	24	84	0

Sistema de Controle de Tráfego Urbano OPTIMUS

5 MINUTOS	INTENSIDADE			
	PM 0402004	PM 0402007	PM04030031	PM04030032
22/05/15 02:45	24	12	24	0
22/05/15 02:50	24	24	120	0
22/05/15 02:55	36	24	48	0
22/05/15 03:00	96	24	60	0
22/05/15 03:05	48	24	48	0
22/05/15 03:10	12	0	48	0
22/05/15 03:15	24	24	36	12
22/05/15 03:20	24	36	36	12
22/05/15 03:25	36	12	24	0
22/05/15 03:30	72	48	48	0
22/05/15 03:35	48	12	12	0
22/05/15 03:40	24	0	24	12
22/05/15 03:45	12	24	12	12
22/05/15 03:50	48	48	60	0
22/05/15 03:55	36	0	12	0
22/05/15 04:00	12	0	24	0
22/05/15 04:05	60	12	0	0
22/05/15 04:10	0	24	84	0
22/05/15 04:15	60	24	36	0
22/05/15 04:20	72	36	60	0
22/05/15 04:25	24	0	48	0
22/05/15 04:30	72	24	0	12
22/05/15 04:35	12	36	12	0
22/05/15 04:40	96	0	48	0
22/05/15 04:45	24	48	48	12
22/05/15 04:50	60	12	24	12
22/05/15 04:55	96	36	24	0
22/05/15 05:00	36	24	12	24
22/05/15 05:05	96	36	24	24
22/05/15 05:10	72	36	36	24
22/05/15 05:15	96	24	24	0
22/05/15 05:20	96	72	96	12
22/05/15 05:25	108	48	72	24
22/05/15 05:30	96	60	84	12
22/05/15 05:35	96	48	0	12
22/05/15 05:40	84	48	96	36
22/05/15 05:45	84	60	108	36
22/05/15 05:50	120	48	168	24
22/05/15 05:55	156	48	144	12
22/05/15 06:00	144	48	252	24
22/05/15 06:05	216	132	228	36
22/05/15 06:10	336	144	276	48
22/05/15 06:15	240	108	240	72
22/05/15 06:20	252	120	408	84
22/05/15 06:25	324	96	504	60
22/05/15 06:30	468	144	624	60
22/05/15 06:35	456	180	528	84
22/05/15 06:40	600	204	504	84
22/05/15 06:45	876	372	696	120
22/05/15 06:50	780	528	864	120
22/05/15 06:55	816	672	1020	300
22/05/15 07:00	732	636	972	216
22/05/15 07:05	972	672	1008	192
22/05/15 07:10	1032	492	924	192
22/05/15 07:15	1080	516	864	228
22/05/15 07:20	960	516	1020	168
22/05/15 07:25	888	408	708	216
22/05/15 07:30	888	516	732	204

Sistema de Controle de Tráfego Urbano OPTIMUS

5 MINUTOS	INTENSIDADE			
	PM 0402004	PM 0402007	PM04030031	PM04030032
22/05/15 07:35	1056	492	804	168
22/05/15 07:40	948	360	744	96
22/05/15 07:45	1140	348	732	144
22/05/15 07:50	1116	348	672	96
22/05/15 07:55	1044	360	720	192
22/05/15 08:00	792	504	804	144
22/05/15 08:05	996	300	792	168
22/05/15 08:10	780	468	684	144
22/05/15 08:15	936	384	912	168
22/05/15 08:20	840	264	756	132
22/05/15 08:25	732	396	696	156
22/05/15 08:30	972	360	708	96
22/05/15 08:35	852	468	756	144
22/05/15 08:40	900	432	684	108
22/05/15 08:45	780	540	708	168
22/05/15 08:50	756	636	876	120
22/05/15 08:55	816	372	900	168
22/05/15 09:00	756	504	840	192
22/05/15 09:05	612	456	648	192
22/05/15 09:10	624	384	744	216
22/05/15 09:15	828	396	780	156
22/05/15 09:20	984	516	852	216
22/05/15 09:25	552	468	888	180
22/05/15 09:30	612	420	744	192
22/05/15 09:35	588	396	924	228
22/05/15 09:40	684	372	984	132
22/05/15 09:45	540	492	768	180
22/05/15 09:50	768	516	840	276
22/05/15 09:55	744	276	780	144
22/05/15 10:00	684	492	852	204
22/05/15 10:05	648	528	768	204
22/05/15 10:10	588	348	864	168
22/05/15 10:15	708	444	804	252
22/05/15 10:20	720	552	876	216
22/05/15 10:25	672	432	648	228
22/05/15 10:30	804	396	828	216
22/05/15 10:35	624	408	900	168
22/05/15 10:40	792	420	780	132
22/05/15 10:45	588	564	792	228
22/05/15 10:50	624	288	744	204
22/05/15 10:55	840	492	780	264
22/05/15 11:00	600	708	828	180
22/05/15 11:05	660	336	744	216
22/05/15 11:10	552	540	864	168
22/05/15 11:15	732	456	756	252
22/05/15 11:20	756	528	804	180
22/05/15 11:25	780	720	888	288
22/05/15 11:30	840	516	960	192
22/05/15 11:35	636	588	816	372
22/05/15 11:40	600	528	912	228
22/05/15 11:45	876	588	696	228
22/05/15 11:50	828	876	768	360
22/05/15 11:55	696	708	1044	288
22/05/15 12:00	792	696	900	288
22/05/15 12:05	636	564	912	276
22/05/15 12:10	744	684	948	324
22/05/15 12:15	600	444	756	288
22/05/15 12:20	720	648	924	300

Sistema de Controle de Tráfego Urbano OPTIMUS

5 MINUTOS	INTENSIDADE			
	PM 0402004	PM 0402007	PM 04030031	PM 04030032
22/05/15 12:25	672	588	804	216
22/05/15 12:30	864	744	828	300
22/05/15 12:35	648	636	936	324
22/05/15 12:40	708	564	1008	312
22/05/15 12:45	744	696	864	264
22/05/15 12:50	732	744	948	216
22/05/15 12:55	792	648	1008	300
22/05/15 13:00	852	840	900	204
22/05/15 13:05	792	516	876	372
22/05/15 13:10	708	612	948	312
22/05/15 13:15	768	612	948	252
22/05/15 13:20	876	636	948	192
22/05/15 13:25	756	540	888	252
22/05/15 13:30	732	720	852	240
22/05/15 13:35	924	684	948	300
22/05/15 13:40	804	516	840	252
22/05/15 13:45	852	768	1008	216
22/05/15 13:50	636	600	972	252
22/05/15 13:55	948	648	924	204
22/05/15 14:00	816	540	828	312
22/05/15 14:05	888	492	900	168
22/05/15 14:10	672	564	840	228
22/05/15 14:15	780	576	984	180
22/05/15 14:20	696	468	828	264
22/05/15 14:25	804	588	792	180
22/05/15 14:30	912	684	984	168
22/05/15 14:35	996	636	960	252
22/05/15 14:40	780	588	1044	300
22/05/15 14:45	756	756	936	228
22/05/15 14:50	636	480	900	300
22/05/15 14:55	816	540	864	252
22/05/15 15:00	780	576	936	240
22/05/15 15:05	816	372	804	252
22/05/15 15:10	648	468	888	228
22/05/15 15:15	696	552	948	288
22/05/15 15:20	900	480	888	228
22/05/15 15:25	792	480	972	240
22/05/15 15:30	756	600	744	192
22/05/15 15:35	768	456	660	144
22/05/15 15:40	624	576	912	216
22/05/15 15:45	696	576	864	264
22/05/15 15:50	744	468	756	216
22/05/15 15:55	732	564	744	204
22/05/15 16:00	720	360	960	156
22/05/15 16:05	696	552	744	216
22/05/15 16:10	708	504	816	360
22/05/15 16:15	792	468	828	156
22/05/15 16:20	660	480	816	156
22/05/15 16:25	648	456	744	276
22/05/15 16:30	528	600	804	264
22/05/15 16:35	816	588	936	252
22/05/15 16:40	792	432	684	348
22/05/15 16:45	624	576	828	324
22/05/15 16:50	720	456	792	216
22/05/15 16:55	720	516	768	324
22/05/15 17:00	816	456	720	204
22/05/15 17:05	744	528	1020	300
22/05/15 17:10	624	552	552	180

Sistema de Controle de Tráfego Urbano OPTIMUS

5 MINUTOS	INTENSIDADE			
	PM 0402004	PM 0402007	PM 04030031	PM 04030032
22/05/15 17:15	528	432	516	264
22/05/15 17:20	432	420	432	168
22/05/15 17:25	372	528	804	348
22/05/15 17:30	252	624	864	180
22/05/15 17:35	492	564	708	276
22/05/15 17:40	240	444	768	240
22/05/15 17:45	336	564	756	204
22/05/15 17:50	372	672	720	72
22/05/15 17:55	360	480	816	264
22/05/15 18:00	372	456	960	144
22/05/15 18:05	444	756	960	264
22/05/15 18:10	624	444	744	156
22/05/15 18:15	516	828	1020	348
22/05/15 18:20	456	636	864	264
22/05/15 18:25	552	624	792	324
22/05/15 18:30	408	684	972	324
22/05/15 18:35	540	540	744	300
22/05/15 18:40	432	660	996	204
22/05/15 18:45	456	612	1044	324
22/05/15 18:50	660	636	828	264
22/05/15 18:55	456	504	852	348
22/05/15 19:00	420	564	756	264
22/05/15 19:05	552	432	828	192
22/05/15 19:10	456	588	960	336
22/05/15 19:15	552	372	636	216
22/05/15 19:20	576	468	912	264
22/05/15 19:25	492	444	720	216
22/05/15 19:30	468	528	864	240
22/05/15 19:35	444	456	852	240
22/05/15 19:40	552	408	936	288
22/05/15 19:45	564	576	672	228
22/05/15 19:50	432	432	732	324
22/05/15 19:55	696	420	636	264
22/05/15 20:00	468	480	888	264
22/05/15 20:05	576	456	840	252
22/05/15 20:10	456	456	828	204
22/05/15 20:15	600	324	576	192
22/05/15 20:20	744	300	720	216
22/05/15 20:25	552	348	720	216
22/05/15 20:30	612	300	744	156
22/05/15 20:35	480	384	696	168
22/05/15 20:40	624	480	744	204
22/05/15 20:45	420	372	684	204
22/05/15 20:50	696	240	720	180
22/05/15 20:55	756	420	828	132
22/05/15 21:00	576	408	660	144
22/05/15 21:05	588	444	660	108
22/05/15 21:10	408	264	600	156
22/05/15 21:15	708	384	840	168
22/05/15 21:20	660	360	624	216
22/05/15 21:25	576	300	744	156
22/05/15 21:30	492	444	600	156
22/05/15 21:35	612	372	672	156
22/05/15 21:40	408	396	588	180
22/05/15 21:45	420	432	468	240
22/05/15 21:50	744	312	588	168
22/05/15 21:55	648	396	696	204
22/05/15 22:00	420	204	648	132

Sistema de Controle de Tráfego Urbano OPTIMUS

5 MINUTOS	INTENSIDADE			
	PM0402004	PM0402007	PM04030031	PM04030032
22/05/15 22:05	420	420	600	120
22/05/15 22:10	516	312	540	192
22/05/15 22:15	492	216	504	156
22/05/15 22:20	504	336	552	168
22/05/15 22:25	528	504	636	204
22/05/15 22:30	456	444	672	252
22/05/15 22:35	516	240	540	144
22/05/15 22:40	420	420	564	204
22/05/15 22:45	444	348	672	228
22/05/15 22:50	420	432	708	216
22/05/15 22:55	564	252	444	192
22/05/15 23:00	528	216	504	72
22/05/15 23:05	492	180	444	120
22/05/15 23:10	360	180	468	72
22/05/15 23:15	360	240	432	60
22/05/15 23:20	312	144	456	96
22/05/15 23:25	420	156	252	96
22/05/15 23:30	324	300	324	120
22/05/15 23:35	396	204	384	132
22/05/15 23:40	432	360	384	96
22/05/15 23:45	204	180	396	108
22/05/15 23:50	324	204	324	84
22/05/15 23:55	348	144	312	96
23/05/15 00:00	324	132	324	36
23/05/15 00:05	492	276	336	108
23/05/15 00:10	384	168	372	24
23/05/15 00:15	276	120	324	24
23/05/15 00:20	276	144	324	48
23/05/15 00:25	288	156	348	48
23/05/15 00:30	288	84	216	24
23/05/15 00:35	168	132	276	36
23/05/15 00:40	288	84	240	36
23/05/15 00:45	264	192	264	60
23/05/15 00:50	384	108	288	108
23/05/15 00:55	96	108	204	36
23/05/15 01:00	300	156	216	48
23/05/15 01:05	324	144	240	24
23/05/15 01:10	288	132	300	36
23/05/15 01:15	216	60	144	24
23/05/15 01:20	204	156	192	36
23/05/15 01:25	192	72	204	48
23/05/15 01:30	204	108	264	48
23/05/15 01:35	156	108	132	72
23/05/15 01:40	216	48	180	36
23/05/15 01:45	204	96	192	24
23/05/15 01:50	168	48	204	12
23/05/15 01:55	168	72	132	24
23/05/15 02:00	192	48	180	24
23/05/15 02:05	216	24	108	12
23/05/15 02:10	192	108	96	24
23/05/15 02:15	132	72	60	36
23/05/15 02:20	168	36	132	36
23/05/15 02:25	132	96	168	36
23/05/15 02:30	240	84	192	0
23/05/15 02:35	192	132	156	0
23/05/15 02:40	144	36	60	12
23/05/15 02:45	108	36	168	24
23/05/15 02:50	180	84	132	48

Sistema de Controle de Tráfego Urbano OPTIMUS

5 MINUTOS	INTENSIDADE			
	PM 0402004	PM 0402007	PM04030031	PM04030032
23/05/15 02:55	108	60	96	12
23/05/15 03:00	120	36	144	0
23/05/15 03:05	192	36	60	24
23/05/15 03:10	228	12	72	12
23/05/15 03:15	84	36	132	12
23/05/15 03:20	132	96	132	36
23/05/15 03:25	144	72	132	36
23/05/15 03:30	144	36	132	0
23/05/15 03:35	108	36	84	24
23/05/15 03:40	120	72	144	12
23/05/15 03:45	156	60	180	12
23/05/15 03:50	108	36	132	12
23/05/15 03:55	60	24	72	0
23/05/15 04:00	240	24	108	24
23/05/15 04:05	144	60	108	0
23/05/15 04:10	156	48	156	0
23/05/15 04:15	144	24	60	0
23/05/15 04:20	180	60	156	12
23/05/15 04:25	204	96	132	12
23/05/15 04:30	84	72	132	24
23/05/15 04:35	168	60	144	24
23/05/15 04:40	156	48	60	12
23/05/15 04:45	132	72	120	24
23/05/15 04:50	204	12	60	0
23/05/15 04:55	72	12	96	0
23/05/15 05:00	120	24	72	24
23/05/15 05:05	168	12	24	0
23/05/15 05:10	96	60	72	12
23/05/15 05:15	96	24	72	0
23/05/15 05:20	156	48	72	24
23/05/15 05:25	120	0	96	0
23/05/15 05:30	168	36	108	12
23/05/15 05:35	96	60	72	24
23/05/15 05:40	228	72	84	24
23/05/15 05:45	144	60	144	48
23/05/15 05:50	132	24	72	0
23/05/15 05:55	132	24	96	0
23/05/15 06:00	144	60	168	48
23/05/15 06:05	108	72	144	12
23/05/15 06:10	156	144	216	48
23/05/15 06:15	156	120	108	36
23/05/15 06:20	204	12	168	36
23/05/15 06:25	252	72	132	36
23/05/15 06:30	252	108	204	36
23/05/15 06:35	192	72	156	24
23/05/15 06:40	240	84	204	48
23/05/15 06:45	384	144	252	48
23/05/15 06:50	240	120	324	24
23/05/15 06:55	324	204	240	108
23/05/15 07:00	348	72	336	36
23/05/15 07:05	396	168	420	36
23/05/15 07:10	324	72	312	36
23/05/15 07:15	324	168	384	120
23/05/15 07:20	300	168	300	36
23/05/15 07:25	552	168	240	48
23/05/15 07:30	384	168	420	108
23/05/15 07:35	396	120	312	72
23/05/15 07:40	360	180	444	48

Sistema de Controle de Tráfego Urbano OPTIMUS

5 MINUTOS	INTENSIDADE			
	PM 0402004	PM 0402007	PM04030031	PM04030032
23/05/15 07:45	516	240	372	96
23/05/15 07:50	348	372	552	96
23/05/15 07:55	480	300	324	108
23/05/15 08:00	372	204	516	36
23/05/15 08:05	684	312	600	132
23/05/15 08:10	480	252	444	84
23/05/15 08:15	384	240	576	108
23/05/15 08:20	528	288	456	60
23/05/15 08:25	684	204	516	84
23/05/15 08:30	564	264	492	96
23/05/15 08:35	684	324	480	144
23/05/15 08:40	540	348	684	96
23/05/15 08:45	504	420	684	168
23/05/15 08:50	552	264	672	144
23/05/15 08:55	708	444	720	180
23/05/15 09:00	504	288	588	96
23/05/15 09:05	768	432	684	168
23/05/15 09:10	504	396	744	144
23/05/15 09:15	792	444	696	144
23/05/15 09:20	576	372	720	180
23/05/15 09:25	564	312	540	108
23/05/15 09:30	660	288	696	84
23/05/15 09:35	732	372	720	180
23/05/15 09:40	552	444	708	96
23/05/15 09:45	684	420	708	168
23/05/15 09:50	612	288	660	108
23/05/15 09:55	816	408	636	228
23/05/15 10:00	540	348	720	156
23/05/15 10:05	816	456	864	228
23/05/15 10:10	552	336	864	120
23/05/15 10:15	828	372	648	168
23/05/15 10:20	684	336	636	228
23/05/15 10:25	780	456	756	216
23/05/15 10:30	660	372	744	192
23/05/15 10:35	816	444	696	264
23/05/15 10:40	816	420	720	216
23/05/15 10:45	732	468	744	216
23/05/15 10:50	648	384	696	144
23/05/15 10:55	828	396	564	120
23/05/15 11:00	672	312	696	168
23/05/15 11:05	876	480	624	228
23/05/15 11:10	780	456	780	180
23/05/15 11:15	852	408	792	216
23/05/15 11:20	576	468	720	204
23/05/15 11:25	828	612	744	336
23/05/15 11:30	564	552	768	312
23/05/15 11:35	900	516	864	288
23/05/15 11:40	564	588	816	168
23/05/15 11:45	732	480	912	324
23/05/15 11:50	768	540	828	192
23/05/15 11:55	804	480	792	312
23/05/15 12:00	732	396	1020	252
23/05/15 12:05	996	624	936	324
23/05/15 12:10	816	492	1116	288
23/05/15 12:15	984	468	996	324
23/05/15 12:20	600	552	984	276
23/05/15 12:25	708	468	876	324
23/05/15 12:30	588	552	972	264

Sistema de Controle de Tráfego Urbano OPTIMUS

5 MINUTOS	INTENSIDADE			
	PM 0402004	PM 0402007	PM04030031	PM04030032
23/05/15 12:35	708	468	780	276
23/05/15 12:40	804	468	1032	180
23/05/15 12:45	612	444	948	360
23/05/15 12:50	672	432	960	288
23/05/15 12:55	648	528	876	240
23/05/15 13:00	564	456	1020	348
23/05/15 13:05	588	576	828	264
23/05/15 13:10	696	432	1008	216
23/05/15 13:15	600	456	876	336
23/05/15 13:20	636	456	900	228
23/05/15 13:25	660	504	708	348
23/05/15 13:30	636	516	840	264
23/05/15 13:35	480	516	900	276
23/05/15 13:40	612	300	864	276
23/05/15 13:45	480	480	708	276
23/05/15 13:50	468	300	816	240
23/05/15 13:55	612	456	876	204
23/05/15 14:00	672	420	804	240
23/05/15 14:05	564	492	912	288
23/05/15 14:10	624	384	648	228
23/05/15 14:15	540	408	660	264
23/05/15 14:20	696	432	660	228
23/05/15 14:25	624	480	696	180
23/05/15 14:30	564	348	624	228
23/05/15 14:35	600	336	768	252
23/05/15 14:40	540	456	816	180
23/05/15 14:45	696	408	840	204
23/05/15 14:50	624	276	732	156
23/05/15 14:55	420	300	612	252
23/05/15 15:00	636	360	612	168
23/05/15 15:05	636	396	660	252
23/05/15 15:10	636	336	588	180
23/05/15 15:15	588	468	648	276
23/05/15 15:20	540	264	636	156
23/05/15 15:25	624	420	588	156
23/05/15 15:30	600	240	696	156
23/05/15 15:35	792	444	648	240
23/05/15 15:40	684	324	744	216
23/05/15 15:45	408	288	756	252
23/05/15 15:50	588	312	588	204
23/05/15 15:55	624	384	696	168
23/05/15 16:00	516	360	804	192
23/05/15 16:05	552	312	600	156
23/05/15 16:10	456	324	732	156
23/05/15 16:15	660	252	660	84
23/05/15 16:20	588	240	564	192
23/05/15 16:25	588	360	612	216
23/05/15 16:30	552	312	648	120
23/05/15 16:35	684	396	660	252
23/05/15 16:40	636	264	588	156
23/05/15 16:45	732	300	672	156
23/05/15 16:50	696	540	684	228
23/05/15 16:55	732	228	540	192
23/05/15 17:00	648	372	612	180
23/05/15 17:05	684	432	660	168
23/05/15 17:10	660	348	756	240
23/05/15 17:15	780	420	516	168
23/05/15 17:20	612	432	720	180

Sistema de Controle de Tráfego Urbano OPTIMUS

5 MINUTOS	INTENSIDADE			
	PM 0402004	PM 0402007	PM 04030031	PM 04030032
23/05/15 17:25	540	324	708	264
23/05/15 17:30	396	264	564	168
23/05/15 17:35	528	264	552	156
23/05/15 17:40	564	252	504	204
23/05/15 17:45	516	348	708	168
23/05/15 17:50	504	360	576	264
23/05/15 17:55	588	336	576	180
23/05/15 18:00	600	408	780	204
23/05/15 18:05	444	336	624	240
23/05/15 18:10	564	288	732	120
23/05/15 18:15	468	324	624	216
23/05/15 18:20	624	288	636	120
23/05/15 18:25	696	300	756	180
23/05/15 18:30	612	312	648	96
23/05/15 18:35	720	300	708	144
23/05/15 18:40	684	372	600	180
23/05/15 18:45	708	276	636	180
23/05/15 18:50	624	324	708	132
23/05/15 18:55	600	336	732	216
23/05/15 19:00	456	288	804	204
23/05/15 19:05	456	300	732	180
23/05/15 19:10	828	384	480	132
23/05/15 19:15	660	456	612	180
23/05/15 19:20	756	228	816	156
23/05/15 19:25	660	312	684	132
23/05/15 19:30	648	504	816	168
23/05/15 19:35	600	288	720	204
23/05/15 19:40	528	288	696	216
23/05/15 19:45	588	288	720	156
23/05/15 19:50	732	276	624	180
23/05/15 19:55	540	396	576	168
23/05/15 20:00	456	264	636	180
23/05/15 20:05	672	240	732	156
23/05/15 20:10	624	348	588	132
23/05/15 20:15	588	264	792	192
23/05/15 20:20	564	240	636	156
23/05/15 20:25	468	324	564	156
23/05/15 20:30	600	360	768	204
23/05/15 20:35	624	216	648	168
23/05/15 20:40	696	408	708	120
23/05/15 20:45	504	228	636	144
23/05/15 20:50	504	228	540	132
23/05/15 20:55	504	264	600	96
23/05/15 21:00	432	384	624	180
23/05/15 21:05	552	180	600	120
23/05/15 21:10	444	228	492	204
23/05/15 21:15	432	156	552	168
23/05/15 21:20	504	180	324	48
23/05/15 21:25	468	408	576	144
23/05/15 21:30	528	312	372	132
23/05/15 21:35	552	204	444	108
23/05/15 21:40	372	252	696	108
23/05/15 21:45	408	264	672	156
23/05/15 21:50	528	264	576	156
23/05/15 21:55	456	384	612	156
23/05/15 22:00	444	216	552	156
23/05/15 22:05	468	252	384	120
23/05/15 22:10	504	252	420	204

Sistema de Controle de Tráfego Urbano OPTIMUS

5 MINUTOS	INTENSIDADE			
	PM 0402004	PM 0402007	PM04030031	PM04030032
23/05/15 22:15	432	204	420	84
23/05/15 22:20	648	252	420	120
23/05/15 22:25	480	264	576	72
23/05/15 22:30	576	252	504	84
23/05/15 22:35	516	192	396	84
23/05/15 22:40	552	144	384	48
23/05/15 22:45	396	264	528	60
23/05/15 22:50	552	288	468	156
23/05/15 22:55	528	276	408	168
23/05/15 23:00	480	300	468	96
23/05/15 23:05	360	204	408	108
23/05/15 23:10	324	192	504	36
23/05/15 23:15	348	156	348	132
23/05/15 23:20	468	288	396	108
23/05/15 23:25	408	144	456	36
23/05/15 23:30	456	192	468	108
23/05/15 23:35	456	192	360	132
23/05/15 23:40	408	192	276	60
23/05/15 23:45	384	192	516	60
23/05/15 23:50	504	264	348	168
23/05/15 23:55	396	240	348	108
24/05/15 00:00	288	96	384	72
24/05/15 00:05	420	60	384	24
24/05/15 00:10	444	108	324	12
24/05/15 00:15	312	216	312	96
24/05/15 00:20	252	96	312	60
24/05/15 00:25	336	144	300	48
24/05/15 00:30	384	36	192	36
24/05/15 00:35	504	72	312	48
24/05/15 00:40	360	60	312	36
24/05/15 00:45	336	120	360	120
24/05/15 00:50	348	132	264	36
24/05/15 00:55	252	192	384	48
24/05/15 01:00	312	120	276	72
24/05/15 01:05	228	168	228	84
24/05/15 01:10	384	108	252	48
24/05/15 01:15	348	120	252	72
24/05/15 01:20	228	84	168	0
24/05/15 01:25	156	96	156	12
24/05/15 01:30	228	168	276	60
24/05/15 01:35	216	48	228	72
24/05/15 01:40	240	168	192	48
24/05/15 01:45	264	96	312	36
24/05/15 01:50	216	72	228	24
24/05/15 01:55	240	24	204	12
24/05/15 02:00	372	36	180	24
24/05/15 02:05	288	84	240	0
24/05/15 02:10	192	84	252	24
24/05/15 02:15	204	72	192	24
24/05/15 02:20	276	48	300	24
24/05/15 02:25	216	96	204	24
24/05/15 02:30	156	36	144	0
24/05/15 02:35	264	84	204	12
24/05/15 02:40	144	12	120	12
24/05/15 02:45	216	24	228	0
24/05/15 02:50	216	120	156	48
24/05/15 02:55	168	36	144	12
24/05/15 03:00	180	12	108	0

Sistema de Controle de Tráfego Urbano OPTIMUS

5 MINUTOS	INTENSIDADE			
	PM 0402004	PM 0402007	PM 04030031	PM 04030032
24/05/15 03:05	108	12	120	0
24/05/15 03:10	180	36	144	12
24/05/15 03:15	156	36	60	12
24/05/15 03:20	144	60	96	24
24/05/15 03:25	108	24	120	24
24/05/15 03:30	168	48	96	0
24/05/15 03:35	108	36	132	24
24/05/15 03:40	96	12	132	0
24/05/15 03:45	156	48	72	12
24/05/15 03:50	192	24	168	0
24/05/15 03:55	108	36	180	0
24/05/15 04:00	108	84	168	24
24/05/15 04:05	144	36	156	0
24/05/15 04:10	168	48	72	12
24/05/15 04:15	36	48	84	24
24/05/15 04:20	108	24	96	0
24/05/15 04:25	60	24	48	0
24/05/15 04:30	108	36	96	12
24/05/15 04:35	72	60	120	24
24/05/15 04:40	144	36	96	0
24/05/15 04:45	96	12	36	0
24/05/15 04:50	96	36	84	0
24/05/15 04:55	108	12	48	0
24/05/15 05:00	156	48	132	24
24/05/15 05:05	192	60	96	36
24/05/15 05:10	144	24	120	0
24/05/15 05:15	72	0	84	0
24/05/15 05:20	156	48	144	0
24/05/15 05:25	96	0	120	0
24/05/15 05:30	96	36	108	0
24/05/15 05:35	132	36	96	24
24/05/15 05:40	144	84	108	24
24/05/15 05:45	84	24	96	12
24/05/15 05:50	120	36	60	0
24/05/15 05:55	36	60	84	24
24/05/15 06:00	156	24	60	36
24/05/15 06:05	192	36	108	48
24/05/15 06:10	72	96	72	36
24/05/15 06:15	192	24	108	0
24/05/15 06:20	180	36	108	12
24/05/15 06:25	96	60	84	24
24/05/15 06:30	132	0	84	0
24/05/15 06:35	96	96	96	36
24/05/15 06:40	120	60	180	0
24/05/15 06:45	264	48	168	24
24/05/15 06:50	156	48	192	48
24/05/15 06:55	216	72	180	60
24/05/15 07:00	216	48	216	12
24/05/15 07:05	216	144	204	48
24/05/15 07:10	192	36	96	24
24/05/15 07:15	228	84	192	36
24/05/15 07:20	156	108	108	36
24/05/15 07:25	240	36	180	36
24/05/15 07:30	252	108	240	12
24/05/15 07:35	312	120	144	36
24/05/15 07:40	204	72	240	48
24/05/15 07:45	288	132	216	48
24/05/15 07:50	168	132	144	36

Sistema de Controle de Tráfego Urbano OPTIMUS

5 MINUTOS	INTENSIDADE			
	PM 0402004	PM 0402007	PM04030031	PM04030032
24/05/15 07:55	204	60	192	36
24/05/15 08:00	240	120	228	60
24/05/15 08:05	216	180	216	72
24/05/15 08:10	228	120	300	36
24/05/15 08:15	252	180	228	48
24/05/15 08:20	252	132	312	60
24/05/15 08:25	192	120	372	60
24/05/15 08:30	216	180	396	36
24/05/15 08:35	192	120	252	36
24/05/15 08:40	336	120	276	48
24/05/15 08:45	192	156	444	60
24/05/15 08:50	360	180	264	48
24/05/15 08:55	312	192	372	72
24/05/15 09:00	336	84	372	48
24/05/15 09:05	384	108	396	48
24/05/15 09:10	312	144	408	48
24/05/15 09:15	360	168	360	36
24/05/15 09:20	312	180	384	60
24/05/15 09:25	348	192	240	96
24/05/15 09:30	324	120	384	84
24/05/15 09:35	360	72	372	72
24/05/15 09:40	372	204	240	36
24/05/15 09:45	324	156	384	72
24/05/15 09:50	444	108	336	48
24/05/15 09:55	360	180	396	84
24/05/15 10:00	324	144	540	84
24/05/15 10:05	204	240	468	84
24/05/15 10:10	288	240	372	72
24/05/15 10:15	276	192	528	84
24/05/15 10:20	372	156	444	84
24/05/15 10:25	312	204	348	72
24/05/15 10:30	456	168	348	96
24/05/15 10:35	408	264	468	96
24/05/15 10:40	456	396	564	156
24/05/15 10:45	492	180	552	96
24/05/15 10:50	288	168	264	84
24/05/15 10:55	396	252	456	96
24/05/15 11:00	324	228	528	36
24/05/15 11:05	408	144	576	72
24/05/15 11:10	384	276	396	132
24/05/15 11:15	456	144	504	84
24/05/15 11:20	420	264	480	120
24/05/15 11:25	660	240	408	108
24/05/15 11:30	516	264	600	108
24/05/15 11:35	432	180	444	144
24/05/15 11:40	396	252	468	108
24/05/15 11:45	384	228	552	120
24/05/15 11:50	456	288	576	120
24/05/15 11:55	564	312	564	168
24/05/15 12:00	864	180	636	144
24/05/15 12:05	576	360	552	156
24/05/15 12:10	552	396	744	192
24/05/15 12:15	468	528	660	168
24/05/15 12:20	624	372	708	228
24/05/15 12:25	660	204	516	216
24/05/15 12:30	648	372	720	156
24/05/15 12:35	804	312	816	192
24/05/15 12:40	612	228	792	132

Sistema de Controle de Tráfego Urbano OPTIMUS

5 MINUTOS	INTENSIDADE			
	PM 0402004	PM 0402007	PM04030031	PM04030032
24/05/15 12:45	624	456	828	240
24/05/15 12:50	564	252	612	96
24/05/15 12:55	552	192	624	108
24/05/15 13:00	480	312	804	168
24/05/15 13:05	492	348	744	144
24/05/15 13:10	540	420	660	216
24/05/15 13:15	444	336	540	132
24/05/15 13:20	492	120	588	48
24/05/15 13:25	492	300	684	108
24/05/15 13:30	396	288	684	168
24/05/15 13:35	528	240	564	120
24/05/15 13:40	504	228	636	120
24/05/15 13:45	480	204	576	144
24/05/15 13:50	432	288	516	144
24/05/15 13:55	408	204	552	120
24/05/15 14:00	336	372	624	204
24/05/15 14:05	456	288	540	144
24/05/15 14:10	432	228	516	120
24/05/15 14:15	492	312	468	168
24/05/15 14:20	444	144	432	84
24/05/15 14:25	468	276	444	144
24/05/15 14:30	384	204	588	108
24/05/15 14:35	384	300	564	132
24/05/15 14:40	396	228	516	108
24/05/15 14:45	432	252	444	120
24/05/15 14:50	384	216	504	108
24/05/15 14:55	468	252	384	84
24/05/15 15:00	504	264	348	144
24/05/15 15:05	348	288	588	144
24/05/15 15:10	468	192	468	120
24/05/15 15:15	348	216	420	84
24/05/15 15:20	528	204	432	120
24/05/15 15:25	528	168	552	60
24/05/15 15:30	528	228	708	132
24/05/15 15:35	348	180	468	84
24/05/15 15:40	480	240	456	144
24/05/15 15:45	540	252	504	96
24/05/15 15:50	516	192	732	72
24/05/15 15:55	564	228	660	120
24/05/15 16:00	516	204	540	72
24/05/15 16:05	612	312	588	132
24/05/15 16:10	780	228	624	108
24/05/15 16:15	456	156	444	72
24/05/15 16:20	600	168	492	96
24/05/15 16:25	468	120	444	84
24/05/15 16:30	564	240	480	120
24/05/15 16:35	480	336	636	108
24/05/15 16:40	396	228	528	96
24/05/15 16:45	552	180	516	108
24/05/15 16:50	720	132	432	156
24/05/15 16:55	756	288	588	96
24/05/15 17:00	672	108	456	60
24/05/15 17:05	492	228	444	156
24/05/15 17:10	480	264	480	120
24/05/15 17:15	624	180	540	108
24/05/15 17:20	516	312	396	108
24/05/15 17:25	480	228	540	108
24/05/15 17:30	468	180	588	120

Sistema de Controle de Tráfego Urbano OPTIMUS

5 MINUTOS	INTENSIDADE			
	PM 0402004	PM 0402007	PM04030031	PM04030032
24/05/15 17:35	708	252	528	156
24/05/15 17:40	540	252	576	108
24/05/15 17:45	588	204	528	72
24/05/15 17:50	516	204	696	192
24/05/15 17:55	420	252	708	108
24/05/15 18:00	444	252	552	168
24/05/15 18:05	504	228	564	204
24/05/15 18:10	552	240	528	120
24/05/15 18:15	660	216	708	156
24/05/15 18:20	576	312	708	36
24/05/15 18:25	648	264	684	96
24/05/15 18:30	540	288	696	156
24/05/15 18:35	648	180	636	168
24/05/15 18:40	636	276	840	120
24/05/15 18:45	516	276	636	192
24/05/15 18:50	636	180	636	204
24/05/15 18:55	516	216	600	168
24/05/15 19:00	396	252	588	108
24/05/15 19:05	456	276	684	132
24/05/15 19:10	516	192	504	132
24/05/15 19:15	432	300	600	120
24/05/15 19:20	552	324	744	120
24/05/15 19:25	444	240	516	120
24/05/15 19:30	600	252	612	144
24/05/15 19:35	360	324	648	132
24/05/15 19:40	396	276	780	180
24/05/15 19:45	444	192	516	132
24/05/15 19:50	528	264	576	168
24/05/15 19:55	636	204	420	156
24/05/15 20:00	408	312	648	192
24/05/15 20:05	456	252	372	168
24/05/15 20:10	420	168	468	84
24/05/15 20:15	468	144	432	96
24/05/15 20:20	420	228	552	96
24/05/15 20:25	432	216	576	108
24/05/15 20:30	540	252	420	144
24/05/15 20:35	444	204	480	144
24/05/15 20:40	516	180	636	120
24/05/15 20:45	540	204	516	168
24/05/15 20:50	432	264	528	120
24/05/15 20:55	324	108	468	72
24/05/15 21:00	384	252	456	120
24/05/15 21:05	372	228	528	96
24/05/15 21:10	456	144	504	60
24/05/15 21:15	492	180	492	60
24/05/15 21:20	588	264	432	120
24/05/15 21:25	468	156	516	156
24/05/15 21:30	480	180	336	108
24/05/15 21:35	420	156	420	60
24/05/15 21:40	312	192	444	84
24/05/15 21:45	396	84	456	96
24/05/15 21:50	396	156	420	144
24/05/15 21:55	396	144	372	48
24/05/15 22:00	336	168	324	120
24/05/15 22:05	276	216	372	120
24/05/15 22:10	300	84	396	72
24/05/15 22:15	276	108	324	60
24/05/15 22:20	276	156	264	84

Sistema de Controle de Tráfego Urbano OPTIMUS

5 MINUTOS	INTENSIDADE			
	PM 0402004	PM 0402007	PM 04030031	PM 04030032
24/05/15 22:25	312	108	300	60
24/05/15 22:30	252	96	216	48
24/05/15 22:35	300	180	324	60
24/05/15 22:40	324	228	252	24
24/05/15 22:45	240	84	204	96
24/05/15 22:50	324	144	228	36
24/05/15 22:55	252	72	240	36
24/05/15 23:00	228	72	180	36
24/05/15 23:05	240	84	216	24
24/05/15 23:10	240	72	120	60
24/05/15 23:15	252	84	264	48
24/05/15 23:20	168	60	168	48
24/05/15 23:25	264	48	132	12
24/05/15 23:30	180	60	120	36
24/05/15 23:35	204	96	156	36
24/05/15 23:40	156	84	144	36
24/05/15 23:45	132	24	120	12
24/05/15 23:50	180	48	144	24
24/05/15 23:55	156	72	120	0
25/05/15 00:00	156	48	120	24
25/05/15 00:05	60	60	180	24
25/05/15 00:10	108	48	192	24
25/05/15 00:15	132	96	156	36
25/05/15 00:20	168	24	156	0
25/05/15 00:25	72	48	72	24
25/05/15 00:30	84	24	120	0
25/05/15 00:35	48	60	108	24
25/05/15 00:40	132	12	48	0
25/05/15 00:45	120	24	84	0
25/05/15 00:50	60	36	48	24
25/05/15 00:55	84	12	24	0
25/05/15 01:00	72	48	60	0
25/05/15 01:05	84	60	96	0
25/05/15 01:10	36	24	72	12
25/05/15 01:15	84	24	72	0
25/05/15 01:20	84	0	120	0
25/05/15 01:25	60	36	72	12
25/05/15 01:30	144	48	156	0
25/05/15 01:35	72	12	12	24
25/05/15 01:40	12	48	12	12
25/05/15 01:45	36	36	48	0
25/05/15 01:50	24	24	48	0
25/05/15 01:55	132	24	12	12
25/05/15 02:00	24	12	60	0
25/05/15 02:05	48	0	0	0
25/05/15 02:10	36	36	48	12
25/05/15 02:15	12	0	24	0
25/05/15 02:20	36	36	48	12
25/05/15 02:25	60	12	36	0
25/05/15 02:30	36	24	12	0
25/05/15 02:35	12	0	36	0
25/05/15 02:40	24	24	24	0
25/05/15 02:45	48	0	12	0
25/05/15 02:50	60	12	60	12
25/05/15 02:55	24	12	0	0
25/05/15 03:00	12	0	12	0
25/05/15 03:05	36	24	24	0
25/05/15 03:10	24	24	12	0

Sistema de Controle de Tráfego Urbano OPTIMUS

5 MINUTOS	INTENSIDADE			
	PM 0402004	PM 0402007	PM04030031	PM04030032
25/05/15 03:15	36	12	24	24
25/05/15 03:20	12	12	36	12
25/05/15 03:25	36	12	36	0
25/05/15 03:30	36	0	0	0
25/05/15 03:35	12	0	0	0
25/05/15 03:40	12	0	0	0
25/05/15 03:45	24	12	12	0
25/05/15 03:50	36	0	0	0
25/05/15 03:55	36	0	12	0
25/05/15 04:00	36	12	12	0
25/05/15 04:05	12	0	24	0
25/05/15 04:10	72	0	0	0
25/05/15 04:15	36	24	60	12
25/05/15 04:20	0	0	0	0
25/05/15 04:25	0	0	24	0
25/05/15 04:30	24	24	36	12
25/05/15 04:35	24	0	0	0
25/05/15 04:40	84	12	12	12
25/05/15 04:45	48	24	36	0
25/05/15 04:50	36	0	24	0
25/05/15 04:55	36	0	12	0
25/05/15 05:00	84	36	0	12
25/05/15 05:05	72	24	36	24
25/05/15 05:10	84	24	36	0
25/05/15 05:15	36	12	24	0
25/05/15 05:20	60	12	12	0
25/05/15 05:25	120	48	60	36
25/05/15 05:30	204	36	72	12
25/05/15 05:35	108	12	60	12
25/05/15 05:40	96	72	84	36
25/05/15 05:45	204	132	132	60
25/05/15 05:50	156	72	216	48
25/05/15 05:55	156	48	108	24
25/05/15 06:00	132	36	288	24
25/05/15 06:05	264	120	204	48
25/05/15 06:10	252	132	276	24
25/05/15 06:15	204	60	180	48
25/05/15 06:20	264	48	456	12
25/05/15 06:25	276	144	420	96
25/05/15 06:30	432	144	480	72
25/05/15 06:35	540	264	600	120
25/05/15 06:40	840	216	564	60
25/05/15 06:45	768	444	756	168
25/05/15 06:50	936	708	924	216
25/05/15 06:55	828	492	888	240
25/05/15 07:00	972	612	888	276
25/05/15 07:05	960	588	900	216
25/05/15 07:10	936	792	876	276
25/05/15 07:15	1044	672	888	180
25/05/15 07:20	1056	660	948	228
25/05/15 07:25	1188	528	912	156
25/05/15 07:30	972	384	744	156
25/05/15 07:35	1008	492	684	96
25/05/15 07:40	828	432	876	144
25/05/15 07:45	1236	348	804	168
25/05/15 07:50	972	288	756	120
25/05/15 07:55	900	432	948	180
25/05/15 08:00	756	264	768	132

Sistema de Controle de Tráfego Urbano OPTIMUS

5 MINUTOS	INTENSIDADE			
	PM 0402004	PM 0402007	PM 04030031	PM 04030032
25/05/15 08:05	1056	480	780	132
25/05/15 08:10	792	384	720	228
25/05/15 08:15	864	432	732	156
25/05/15 08:20	732	480	744	144
25/05/15 08:25	684	492	684	120
25/05/15 08:30	672	444	792	180
25/05/15 08:35	804	456	780	216
25/05/15 08:40	840	396	720	144
25/05/15 08:45	828	516	804	156
25/05/15 08:50	684	432	996	144
25/05/15 08:55	792	396	816	180
25/05/15 09:00	768	492	900	216
25/05/15 09:05	732	540	876	264
25/05/15 09:10	768	528	804	120
25/05/15 09:15	708	468	672	120
25/05/15 09:20	660	444	972	144
25/05/15 09:25	660	408	684	108
25/05/15 09:30	528	396	1044	192
25/05/15 09:35	624	492	816	180
25/05/15 09:40	648	348	864	228
25/05/15 09:45	804	396	948	204
25/05/15 09:50	960	348	876	192
25/05/15 09:55	1044	684	660	240
25/05/15 10:00	900	480	816	192
25/05/15 10:05	636	492	756	144
25/05/15 10:10	780	420	768	192
25/05/15 10:15	936	480	792	192
25/05/15 10:20	720	396	888	276
25/05/15 10:25	660	588	900	204
25/05/15 10:30	708	540	732	240
25/05/15 10:35	828	396	804	204
25/05/15 10:40	576	372	600	120
25/05/15 10:45	828	360	840	156
25/05/15 10:50	744	456	792	168
25/05/15 10:55	576	444	684	264
25/05/15 11:00	528	324	720	240
25/05/15 11:05	648	468	780	276
25/05/15 11:10	648	432	744	156
25/05/15 11:15	780	432	600	228
25/05/15 11:20	540	432	612	228
25/05/15 11:25	720	432	792	120
25/05/15 11:30	636	324	756	276
25/05/15 11:35	540	612	864	168
25/05/15 11:40	648	384	696	204
25/05/15 11:45	696	660	876	180
25/05/15 11:50	648	792	924	360
25/05/15 11:55	672	864	1152	336
25/05/15 12:00	528	780	1140	360
25/05/15 12:05	648	564	864	288
25/05/15 12:10	624	576	900	360
25/05/15 12:15	948	528	876	228
25/05/15 12:20	504	648	948	228
25/05/15 12:25	624	516	864	300
25/05/15 12:30	684	456	816	192
25/05/15 12:35	588	516	936	180
25/05/15 12:40	744	480	936	228
25/05/15 12:45	780	600	1056	300
25/05/15 12:50	792	600	888	168

Sistema de Controle de Tráfego Urbano OPTIMUS

5 MINUTOS	INTENSIDADE			
	PM 0402004	PM 0402007	PM 04030031	PM 04030032
25/05/15 12:55	804	816	900	288
25/05/15 13:00	876	636	996	276
25/05/15 13:05	960	612	1044	276
25/05/15 13:10	780	528	1092	216
25/05/15 13:15	792	480	960	192
25/05/15 13:20	708	444	948	180
25/05/15 13:25	732	360	984	132
25/05/15 13:30	828	504	924	204
25/05/15 13:35	852	396	768	156
25/05/15 13:40	540	564	900	168
25/05/15 13:45	852	312	1020	216
25/05/15 13:50	708	624	888	168
25/05/15 13:55	600	276	828	192
25/05/15 14:00	804	360	852	192
25/05/15 14:05	780	564	972	276
25/05/15 14:10	864	396	684	216
25/05/15 14:15	720	528	852	252
25/05/15 14:20	780	372	852	168
25/05/15 14:25	804	504	888	192
25/05/15 14:30	864	516	732	204
25/05/15 14:35	876	516	864	168
25/05/15 14:40	696	672	780	264
25/05/15 14:45	876	396	912	216
25/05/15 14:50	636	528	876	180
25/05/15 14:55	900	564	1068	240
25/05/15 15:00	492	444	804	156
25/05/15 15:05	792	468	780	240
25/05/15 15:10	756	528	804	216
25/05/15 15:15	720	432	804	216
25/05/15 15:20	600	432	792	192
25/05/15 15:25	864	492	804	252
25/05/15 15:30	600	396	840	168
25/05/15 15:35	708	540	888	192
25/05/15 15:40	600	372	840	168
25/05/15 15:45	780	432	948	180
25/05/15 15:50	660	504	876	204
25/05/15 15:55	732	576	756	168
25/05/15 16:00	792	564	828	192
25/05/15 16:05	768	384	888	144
25/05/15 16:10	624	588	780	216
25/05/15 16:15	876	468	684	180
25/05/15 16:20	780	384	672	180
25/05/15 16:25	732	612	708	216
25/05/15 16:30	768	456	600	180
25/05/15 16:35	756	420	816	300
25/05/15 16:40	924	456	912	216
25/05/15 16:45	756	444	792	264
25/05/15 16:50	552	384	792	216
25/05/15 16:55	564	648	864	264
25/05/15 17:00	684	600	780	228
25/05/15 17:05	480	540	876	276
25/05/15 17:10	780	492	840	264
25/05/15 17:15	468	456	708	216
25/05/15 17:20	540	444	696	228
25/05/15 17:25	552	408	756	276
25/05/15 17:30	588	636	504	264
25/05/15 17:35	492	696	624	264
25/05/15 17:40	540	732	576	228

Sistema de Controle de Tráfego Urbano OPTIMUS

5 MINUTOS	INTENSIDADE			
	PM 0402004	PM 0402007	PM 04030031	PM 04030032
25/05/15 17:45	468	636	936	216
25/05/15 17:50	612	696	1080	288
25/05/15 17:55	672	672	1236	348
25/05/15 18:00	612	648	1152	276
25/05/15 18:05	504	696	1056	276
25/05/15 18:10	456	588	912	300
25/05/15 18:15	540	276	876	132
25/05/15 18:20	384	504	864	252
25/05/15 18:25	420	432	768	168
25/05/15 18:30	348	708	648	228
25/05/15 18:35	480	468	960	300
25/05/15 18:40	240	576	888	228
25/05/15 18:45	396	600	912	312
25/05/15 18:50	480	468	852	288
25/05/15 18:55	624	480	636	240
25/05/15 19:00	444	564	924	312
25/05/15 19:05	540	444	780	216
25/05/15 19:10	528	444	768	204
25/05/15 19:15	420	432	984	240
25/05/15 19:20	468	336	864	192
25/05/15 19:25	552	480	684	156
25/05/15 19:30	372	588	852	348
25/05/15 19:35	540	408	792	252
25/05/15 19:40	324	432	744	276
25/05/15 19:45	468	552	648	252
25/05/15 19:50	384	432	660	192
25/05/15 19:55	432	336	552	156
25/05/15 20:00	408	264	564	192
25/05/15 20:05	360	372	612	180
25/05/15 20:10	552	396	504	228
25/05/15 20:15	432	264	588	120
25/05/15 20:20	420	216	636	96
25/05/15 20:25	600	300	696	144
25/05/15 20:30	432	324	480	72
25/05/15 20:35	564	324	684	132
25/05/15 20:40	480	420	612	168
25/05/15 20:45	288	288	540	108
25/05/15 20:50	504	312	480	156
25/05/15 20:55	348	336	576	168
25/05/15 21:00	396	216	408	108
25/05/15 21:05	360	240	444	120
25/05/15 21:10	516	288	480	132
25/05/15 21:15	444	264	384	108
25/05/15 21:20	456	324	480	132
25/05/15 21:25	396	288	432	108
25/05/15 21:30	360	276	396	120
25/05/15 21:35	324	264	516	108
25/05/15 21:40	504	240	288	144
25/05/15 21:45	360	252	468	120
25/05/15 21:50	324	240	468	96
25/05/15 21:55	300	288	444	144
25/05/15 22:00	372	228	468	108
25/05/15 22:05	432	276	384	168
25/05/15 22:10	276	264	516	156
25/05/15 22:15	420	372	540	168
25/05/15 22:20	480	168	540	108
25/05/15 22:25	348	288	516	180
25/05/15 22:30	432	396	444	156

Sistema de Controle de Tráfego Urbano OPTIMUS

5 MINUTOS	INTENSIDADE			
	PM 0402004	PM 0402007	PM04030031	PM04030032
25/05/15 22:35	336	252	408	156
25/05/15 22:40	408	348	504	216
25/05/15 22:45	204	276	408	144
25/05/15 22:50	204	228	396	156
25/05/15 22:55	192	228	420	120
25/05/15 23:00	168	132	324	24
25/05/15 23:05	240	120	204	84
25/05/15 23:10	216	144	312	84
25/05/15 23:15	216	120	300	72
25/05/15 23:20	360	84	264	24
25/05/15 23:25	192	156	216	60
25/05/15 23:30	132	84	180	24
25/05/15 23:35	204	0	120	36
25/05/15 23:40	204	120	180	60
25/05/15 23:45	108	36	156	12
25/05/15 23:50	144	96	132	12
25/05/15 23:55	144	0	72	36
26/05/15 00:00	156	84	120	12
26/05/15 00:05	120	72	144	24
26/05/15 00:10	132	48	168	36
26/05/15 00:15	156	36	120	12
26/05/15 00:20	108	72	180	12
26/05/15 00:25	108	12	36	12
26/05/15 00:30	96	36	72	36
26/05/15 00:35	120	72	84	36
26/05/15 00:40	60	24	120	0
26/05/15 00:45	72	36	84	0
26/05/15 00:50	72	36	36	12
26/05/15 00:55	36	60	48	12
26/05/15 01:00	84	36	72	12
26/05/15 01:05	60	0	24	0
26/05/15 01:10	84	36	36	0
26/05/15 01:15	36	12	36	12
26/05/15 01:20	72	36	60	12
26/05/15 01:25	48	36	60	12
26/05/15 01:30	84	12	24	0
26/05/15 01:35	36	24	48	12
26/05/15 01:40	60	72	84	24
26/05/15 01:45	48	36	48	0
26/05/15 01:50	72	12	12	24
26/05/15 01:55	36	0	24	0
26/05/15 02:00	48	0	12	0
26/05/15 02:05	72	0	36	0
26/05/15 02:10	60	0	48	12
26/05/15 02:15	48	0	12	0
26/05/15 02:20	24	0	12	0
26/05/15 02:25	84	12	24	0
26/05/15 02:30	96	0	36	0
26/05/15 02:35	36	0	12	0
26/05/15 02:40	24	0	0	0
26/05/15 02:45	36	0	24	0
26/05/15 02:50	24	12	12	0
26/05/15 02:55	12	12	24	0
26/05/15 03:00	24	0	12	0
26/05/15 03:05	36	0	12	0
26/05/15 03:10	12	12	48	0
26/05/15 03:15	12	12	36	12
26/05/15 03:20	24	24	12	12

Sistema de Controle de Tráfego Urbano OPTIMUS

5 MINUTOS	INTENSIDADE			
	PM0402004	PM0402007	PM04030031	PM04030032
26/05/15 03:25	24	0	24	0
26/05/15 03:30	0	24	12	12
26/05/15 03:35	12	24	12	0
26/05/15 03:40	0	0	0	0
26/05/15 03:45	12	12	24	0
26/05/15 03:50	12	12	12	0
26/05/15 03:55	60	0	12	0
26/05/15 04:00	48	0	24	0
26/05/15 04:05	12	0	24	0
26/05/15 04:10	36	0	0	0
26/05/15 04:15	12	0	0	0
26/05/15 04:20	24	0	24	0
26/05/15 04:25	36	0	12	0
26/05/15 04:30	48	24	36	12
26/05/15 04:35	84	0	24	0
26/05/15 04:40	24	12	48	0
26/05/15 04:45	24	0	36	12
26/05/15 04:50	36	12	48	0
26/05/15 04:55	48	12	24	0
26/05/15 05:00	72	36	12	24
26/05/15 05:05	168	24	36	0
26/05/15 05:10	72	24	48	0
26/05/15 05:15	60	12	24	0
26/05/15 05:20	84	24	0	0
26/05/15 05:25	96	96	96	36
26/05/15 05:30	84	36	60	12
26/05/15 05:35	24	60	60	12
26/05/15 05:40	144	108	156	24
26/05/15 05:45	72	84	72	24
26/05/15 05:50	168	72	156	24
26/05/15 05:55	132	60	84	0
26/05/15 06:00	168	72	324	36
26/05/15 06:05	192	108	216	36
26/05/15 06:10	192	72	204	24
26/05/15 06:15	216	96	240	36
26/05/15 06:20	228	96	240	36
26/05/15 06:25	372	168	396	72
26/05/15 06:30	396	252	660	60
26/05/15 06:35	312	204	564	132
26/05/15 06:40	636	324	408	60
26/05/15 06:45	984	432	804	168
26/05/15 06:50	804	564	1128	144
26/05/15 06:55	780	696	900	288
26/05/15 07:00	1056	660	1032	264
26/05/15 07:05	768	672	984	240
26/05/15 07:10	1068	360	876	168
26/05/15 07:15	936	696	888	216
26/05/15 07:20	996	576	912	180
26/05/15 07:25	708	492	768	180
26/05/15 07:30	996	480	816	204
26/05/15 07:35	960	444	744	168
26/05/15 07:40	984	324	816	108
26/05/15 07:45	1044	348	792	72
26/05/15 07:50	1128	372	984	132
26/05/15 07:55	840	444	708	156
26/05/15 08:00	924	396	696	60
26/05/15 08:05	816	336	612	144
26/05/15 08:10	864	396	876	168

Sistema de Controle de Tráfego Urbano OPTIMUS

5 MINUTOS	INTENSIDADE			
	PM 0402004	PM 0402007	PM 04030031	PM 04030032
26/05/15 08:15	864	240	624	120
26/05/15 08:20	1044	600	900	192
26/05/15 08:25	840	252	660	180
26/05/15 08:30	1008	264	732	156
26/05/15 08:35	648	372	660	132
26/05/15 08:40	768	492	708	192
26/05/15 08:45	648	516	888	228
26/05/15 08:50	1032	456	912	144
26/05/15 08:55	972	384	864	252
26/05/15 09:00	1020	336	744	168
26/05/15 09:05	660	516	792	180
26/05/15 09:10	960	420	840	96
26/05/15 09:15	828	420	780	216
26/05/15 09:20	984	456	684	180
26/05/15 09:25	744	444	648	204
26/05/15 09:30	864	312	828	228
26/05/15 09:35	672	456	804	132
26/05/15 09:40	504	348	828	192
26/05/15 09:45	672	372	852	204
26/05/15 09:50	792	264	732	132
26/05/15 09:55	492	504	744	192
26/05/15 10:00	876	492	756	216
26/05/15 10:05	648	372	912	216
26/05/15 10:10	564	444	804	156
26/05/15 10:15	708	432	720	216
26/05/15 10:20	564	372	756	84
26/05/15 10:25	780	552	900	252
26/05/15 10:30	564	396	780	168
26/05/15 10:35	708	504	720	120
26/05/15 10:40	588	288	828	168
26/05/15 10:45	720	420	672	180
26/05/15 10:50	720	504	576	180
26/05/15 10:55	696	372	816	180
26/05/15 11:00	636	360	624	216
26/05/15 11:05	792	528	708	192
26/05/15 11:10	816	492	708	180
26/05/15 11:15	936	552	720	240
26/05/15 11:20	528	588	828	264
26/05/15 11:25	708	492	720	348
26/05/15 11:30	588	696	804	288
26/05/15 11:35	720	564	876	228
26/05/15 11:40	660	468	756	132
26/05/15 11:45	672	468	660	180
26/05/15 11:50	684	720	804	312
26/05/15 11:55	672	600	864	288
26/05/15 12:00	600	684	792	240
26/05/15 12:05	768	588	864	324
26/05/15 12:10	624	336	912	264
26/05/15 12:15	696	600	792	276
26/05/15 12:20	540	588	840	216
26/05/15 12:25	708	516	792	228
26/05/15 12:30	516	420	912	192
26/05/15 12:35	576	432	672	240
26/05/15 12:40	948	420	816	144
26/05/15 12:45	732	612	1008	252
26/05/15 12:50	384	588	864	228
26/05/15 12:55	552	624	900	276
26/05/15 13:00	900	600	912	216

Sistema de Controle de Tráfego Urbano OPTIMUS

5 MINUTOS	INTENSIDADE			
	PM 0402004	PM 0402007	PM04030031	PM04030032
26/05/15 13:05	636	828	1116	408
26/05/15 13:10	900	600	972	216
26/05/15 13:15	636	504	984	264
26/05/15 13:20	852	672	828	252
26/05/15 13:25	588	456	948	276
26/05/15 13:30	852	360	852	252
26/05/15 13:35	888	420	936	252
26/05/15 13:40	768	492	732	156
26/05/15 13:45	792	540	768	108
26/05/15 13:50	840	540	828	276
26/05/15 13:55	696	324	792	168
26/05/15 14:00	768	408	756	180
26/05/15 14:05	972	480	840	180
26/05/15 14:10	696	540	888	300
26/05/15 14:15	720	372	900	228
26/05/15 14:20	744	516	708	132
26/05/15 14:25	648	576	816	216
26/05/15 14:30	684	408	828	264
26/05/15 14:35	900	492	840	180
26/05/15 14:40	660	348	876	300
26/05/15 14:45	804	588	852	216
26/05/15 14:50	756	504	780	216
26/05/15 14:55	696	372	924	228
26/05/15 15:00	792	420	672	192
26/05/15 15:05	648	444	720	228
26/05/15 15:10	660	600	756	276
26/05/15 15:15	720	552	828	168
26/05/15 15:20	768	504	888	252
26/05/15 15:25	744	528	840	264
26/05/15 15:30	876	564	804	216
26/05/15 15:35	660	360	924	204
26/05/15 15:40	792	516	1032	252
26/05/15 15:45	780	456	888	276
26/05/15 15:50	756	636	840	252
26/05/15 15:55	720	420	876	252
26/05/15 16:00	432	612	912	312
26/05/15 16:05	816	444	768	252
26/05/15 16:10	816	432	996	96
26/05/15 16:15	612	348	804	204
26/05/15 16:20	600	456	816	300
26/05/15 16:25	576	516	996	264
26/05/15 16:30	744	504	792	252
26/05/15 16:35	648	480	720	192
26/05/15 16:40	612	396	864	192
26/05/15 16:45	624	324	612	216
26/05/15 16:50	564	528	912	228
26/05/15 16:55	612	672	864	228
26/05/15 17:00	684	444	684	300
26/05/15 17:05	636	552	804	240
26/05/15 17:10	612	504	876	252
26/05/15 17:15	600	636	792	300
26/05/15 17:20	504	576	804	216
26/05/15 17:25	480	456	912	312
26/05/15 17:30	552	660	756	264
26/05/15 17:35	456	672	756	324
26/05/15 17:40	612	612	564	288
26/05/15 17:45	552	600	840	264
26/05/15 17:50	624	540	708	192

Sistema de Controle de Tráfego Urbano OPTIMUS

5 MINUTOS	INTENSIDADE			
	PM 0402004	PM 0402007	PM 04030031	PM 04030032
26/05/15 17:55	396	468	1092	312
26/05/15 18:00	552	648	1032	312
26/05/15 18:05	576	444	864	204
26/05/15 18:10	564	492	624	264
26/05/15 18:15	396	312	828	144
26/05/15 18:20	432	288	792	168
26/05/15 18:25	528	648	792	144
26/05/15 18:30	540	300	816	276
26/05/15 18:35	660	480	432	144
26/05/15 18:40	744	420	756	360
26/05/15 18:45	624	720	984	264
26/05/15 18:50	444	684	912	420
26/05/15 18:55	708	564	1236	252
26/05/15 19:00	576	612	816	264
26/05/15 19:05	456	552	972	144
26/05/15 19:10	432	432	816	252
26/05/15 19:15	660	396	900	192
26/05/15 19:20	552	408	852	192
26/05/15 19:25	432	552	756	264
26/05/15 19:30	516	408	852	240
26/05/15 19:35	480	420	792	276
26/05/15 19:40	468	456	636	192
26/05/15 19:45	468	456	720	204
26/05/15 19:50	300	504	744	204
26/05/15 19:55	324	360	732	132
26/05/15 20:00	564	408	672	228
26/05/15 20:05	456	360	696	156
26/05/15 20:10	300	456	672	144
26/05/15 20:15	552	384	792	252
26/05/15 20:20	516	324	516	276
26/05/15 20:25	588	252	636	132
26/05/15 20:30	348	252	636	144
26/05/15 20:35	348	408	576	216
26/05/15 20:40	444	444	720	192
26/05/15 20:45	432	312	576	156
26/05/15 20:50	480	312	648	120
26/05/15 20:55	612	264	624	168
26/05/15 21:00	384	276	588	96
26/05/15 21:05	432	264	492	72
26/05/15 21:10	516	264	432	96
26/05/15 21:15	360	360	576	132
26/05/15 21:20	468	348	372	132
26/05/15 21:25	492	288	540	180
26/05/15 21:30	276	300	360	168
26/05/15 21:35	468	276	432	168
26/05/15 21:40	480	240	432	144
26/05/15 21:45	396	252	516	132
26/05/15 21:50	288	312	408	144
26/05/15 21:55	324	204	468	132
26/05/15 22:00	480	180	420	72
26/05/15 22:05	444	324	408	144
26/05/15 22:10	588	372	480	216
26/05/15 22:15	444	288	636	120
26/05/15 22:20	420	264	528	156
26/05/15 22:25	396	300	564	144
26/05/15 22:30	432	312	456	204
26/05/15 22:35	420	216	432	132
26/05/15 22:40	456	444	456	180

Sistema de Controle de Tráfego Urbano OPTIMUS

5 MINUTOS	INTENSIDADE			
	PM 0402004	PM 0402007	PM04030031	PM04030032
26/05/15 22:45	312	192	408	108
26/05/15 22:50	240	264	444	168
26/05/15 22:55	276	252	324	132
26/05/15 23:00	420	120	348	84
26/05/15 23:05	312	216	240	84
26/05/15 23:10	264	72	252	48
26/05/15 23:15	216	132	264	48
26/05/15 23:20	216	120	228	36
26/05/15 23:25	204	192	204	48
26/05/15 23:30	252	108	288	72
26/05/15 23:35	240	96	228	36
26/05/15 23:40	300	72	180	36
26/05/15 23:45	120	72	156	36
26/05/15 23:50	228	72	120	12
26/05/15 23:55	144	84	228	60
27/05/15 00:00	144	12	132	0
27/05/15 00:05	132	24	84	0
27/05/15 00:10	132	48	132	0
27/05/15 00:15	132	12	96	12
27/05/15 00:20	108	0	132	12
27/05/15 00:25	96	60	120	12
27/05/15 00:30	108	12	120	12
27/05/15 00:35	96	84	180	24
27/05/15 00:40	132	96	132	24
27/05/15 00:45	60	60	96	36
27/05/15 00:50	132	120	132	24
27/05/15 00:55	72	60	84	36
27/05/15 01:00	168	24	72	0
27/05/15 01:05	96	12	60	0
27/05/15 01:10	156	48	96	12
27/05/15 01:15	84	60	48	12
27/05/15 01:20	24	24	36	24
27/05/15 01:25	48	36	108	12
27/05/15 01:30	48	24	36	12
27/05/15 01:35	12	36	24	24
27/05/15 01:40	60	48	48	24
27/05/15 01:45	48	12	84	0
27/05/15 01:50	84	36	60	12
27/05/15 01:55	96	0	36	0
27/05/15 02:00	72	0	48	12
27/05/15 02:05	72	24	12	24
27/05/15 02:10	36	24	72	24
27/05/15 02:15	60	0	48	0
27/05/15 02:20	36	0	84	12
27/05/15 02:25	24	12	36	0
27/05/15 02:30	48	0	72	0
27/05/15 02:35	36	12	60	0
27/05/15 02:40	72	12	12	0
27/05/15 02:45	0	0	24	0
27/05/15 02:50	12	24	36	0
27/05/15 02:55	24	0	12	0
27/05/15 03:00	48	12	12	0
27/05/15 03:05	36	0	0	0
27/05/15 03:10	36	0	24	0
27/05/15 03:15	24	36	12	12
27/05/15 03:20	60	12	36	12
27/05/15 03:25	12	36	0	0
27/05/15 03:30	12	0	12	0

Sistema de Controle de Tráfego Urbano OPTIMUS

5 MINUTOS	INTENSIDADE			
	PM 0402004	PM 0402007	PM04030031	PM04030032
27/05/15 03:35	0	12	36	0
27/05/15 03:40	0	0	12	0
27/05/15 03:45	24	24	12	0
27/05/15 03:50	24	36	24	0
27/05/15 03:55	36	0	12	0
27/05/15 04:00	48	12	36	0
27/05/15 04:05	24	0	24	0
27/05/15 04:10	72	0	12	0
27/05/15 04:15	24	0	12	0
27/05/15 04:20	72	0	12	0
27/05/15 04:25	12	12	12	0
27/05/15 04:30	36	0	12	12
27/05/15 04:35	36	24	12	24
27/05/15 04:40	48	36	12	12
27/05/15 04:45	24	12	48	12
27/05/15 04:50	24	24	24	0
27/05/15 04:55	36	12	36	12
27/05/15 05:00	12	60	36	24
27/05/15 05:05	48	24	12	0
27/05/15 05:10	108	24	84	0
27/05/15 05:15	36	0	0	0
27/05/15 05:20	108	48	48	48
27/05/15 05:25	96	60	84	12
27/05/15 05:30	120	24	84	12
27/05/15 05:35	144	12	96	12
27/05/15 05:40	84	36	120	36
27/05/15 05:45	84	60	48	36
27/05/15 05:50	216	60	264	24
27/05/15 05:55	168	60	192	24
27/05/15 06:00	132	96	300	24
27/05/15 06:05	156	120	216	60
27/05/15 06:10	264	168	204	0
27/05/15 06:15	132	84	300	108
27/05/15 06:20	252	192	408	84
27/05/15 06:25	360	84	480	72
27/05/15 06:30	384	120	552	60
27/05/15 06:35	432	204	696	60
27/05/15 06:40	720	240	576	96
27/05/15 06:45	936	396	660	168
27/05/15 06:50	720	672	1044	180
27/05/15 06:55	708	672	936	252
27/05/15 07:00	888	648	924	228
27/05/15 07:05	828	660	948	324
27/05/15 07:10	1104	612	948	192
27/05/15 07:15	1116	516	684	252
27/05/15 07:20	864	672	900	204
27/05/15 07:25	876	516	900	228
27/05/15 07:30	1056	552	852	192
27/05/15 07:35	768	300	708	216
27/05/15 07:40	1080	564	636	180
27/05/15 07:45	1020	480	864	192
27/05/15 07:50	936	564	948	192
27/05/15 07:55	1068	420	732	216
27/05/15 08:00	852	468	936	156
27/05/15 08:05	900	528	816	240
27/05/15 08:10	768	396	576	84
27/05/15 08:15	960	432	888	120
27/05/15 08:20	924	300	792	132

Sistema de Controle de Tráfego Urbano OPTIMUS

5 MINUTOS	INTENSIDADE			
	PM040200 4	PM040200 7	PM04030031	PM04030032
27/05/15 08:25	1008	312	624	156
27/05/15 08:30	744	480	768	180
27/05/15 08:35	852	468	792	228
27/05/15 08:40	684	384	768	204
27/05/15 08:45	960	504	768	132
27/05/15 08:50	1068	384	816	216
27/05/15 08:55	720	468	948	192
27/05/15 09:00	876	264	732	192
27/05/15 09:05	900	456	852	108
27/05/15 09:10	708	528	828	156
27/05/15 09:15	804	516	792	132
27/05/15 09:20	780	504	960	192
27/05/15 09:25	720	444	804	192
27/05/15 09:30	588	444	588	228
27/05/15 09:35	888	324	792	144
27/05/15 09:40	504	624	864	192
27/05/15 09:45	756	348	816	144
27/05/15 09:50	708	492	864	276
27/05/15 09:55	732	336	696	144
27/05/15 10:00	648	432	684	156
27/05/15 10:05	816	444	816	192
27/05/15 10:10	540	300	780	180
27/05/15 10:15	708	600	720	156
27/05/15 10:20	804	492	948	180
27/05/15 10:25	600	564	648	240
27/05/15 10:30	648	516	780	252
27/05/15 10:35	648	468	684	180
27/05/15 10:40	588	384	888	192
27/05/15 10:45	660	432	876	180
27/05/15 10:50	624	516	924	228
27/05/15 10:55	828	480	768	216
27/05/15 11:00	660	528	924	348
27/05/15 11:05	564	384	828	216
27/05/15 11:10	792	396	720	228
27/05/15 11:15	756	384	588	180
27/05/15 11:20	624	504	792	240
27/05/15 11:25	804	600	960	156
27/05/15 11:30	720	588	708	228
27/05/15 11:35	720	516	732	252
27/05/15 11:40	432	504	828	228
27/05/15 11:45	660	504	672	192
27/05/15 11:50	888	684	972	264
27/05/15 11:55	804	552	924	312
27/05/15 12:00	648	432	1068	252
27/05/15 12:05	576	684	924	348
27/05/15 12:10	672	648	936	276
27/05/15 12:15	744	528	1068	180
27/05/15 12:20	564	528	816	252
27/05/15 12:25	684	540	912	180
27/05/15 12:30	768	684	768	228
27/05/15 12:35	672	444	852	216
27/05/15 12:40	624	456	984	228
27/05/15 12:45	456	384	840	204
27/05/15 12:50	696	696	960	228
27/05/15 12:55	564	744	1056	264
27/05/15 13:00	816	624	996	240
27/05/15 13:05	720	804	972	360
27/05/15 13:10	948	624	1092	288

Sistema de Controle de Tráfego Urbano OPTIMUS

5 MINUTOS	INTENSIDADE			
	PM 0402004	PM 0402007	PM04030031	PM04030032
27/05/15 13:15	1044	552	936	168
27/05/15 13:20	504	516	1080	228
27/05/15 13:25	864	564	912	276
27/05/15 13:30	672	612	1032	348
27/05/15 13:35	960	360	924	228
27/05/15 13:40	828	444	696	228
27/05/15 13:45	900	324	972	168
27/05/15 13:50	708	492	744	180
27/05/15 13:55	696	636	924	228
27/05/15 14:00	840	372	732	216
27/05/15 14:05	936	444	864	72
27/05/15 14:10	840	528	924	264
27/05/15 14:15	984	372	612	204
27/05/15 14:20	588	516	684	84
27/05/15 14:25	792	408	780	264
27/05/15 14:30	708	468	624	204
27/05/15 14:35	696	408	864	180
27/05/15 14:40	540	576	1008	228
27/05/15 14:45	756	564	888	312
27/05/15 14:50	780	684	900	264
27/05/15 14:55	792	480	876	180
27/05/15 15:00	804	360	840	168
27/05/15 15:05	804	348	804	204
27/05/15 15:10	720	456	828	180
27/05/15 15:15	648	300	672	192
27/05/15 15:20	744	576	888	252
27/05/15 15:25	708	492	828	180
27/05/15 15:30	780	432	660	168
27/05/15 15:35	792	600	828	300
27/05/15 15:40	732	504	648	204
27/05/15 15:45	804	456	852	204
27/05/15 15:50	780	504	828	324
27/05/15 15:55	780	480	960	168
27/05/15 16:00	768	660	804	300
27/05/15 16:05	768	396	912	240
27/05/15 16:10	672	480	768	264
27/05/15 16:15	636	336	744	168
27/05/15 16:20	588	456	828	180
27/05/15 16:25	744	588	660	240
27/05/15 16:30	720	408	600	228
27/05/15 16:35	648	636	780	288
27/05/15 16:40	600	372	852	120
27/05/15 16:45	636	552	744	216
27/05/15 16:50	648	492	792	204
27/05/15 16:55	516	456	804	156
27/05/15 17:00	876	468	768	156
27/05/15 17:05	528	456	948	252
27/05/15 17:10	600	528	900	252
27/05/15 17:15	588	372	888	264
27/05/15 17:20	552	660	864	264
27/05/15 17:25	636	576	684	252
27/05/15 17:30	324	564	612	204
27/05/15 17:35	372	624	792	336
27/05/15 17:40	264	564	684	216
27/05/15 17:45	516	816	1056	324
27/05/15 17:50	612	660	876	204
27/05/15 17:55	540	420	900	288
27/05/15 18:00	480	456	780	204

Sistema de Controle de Tráfego Urbano OPTIMUS

5 MINUTOS	INTENSIDADE			
	PM 0402004	PM 0402007	PM04030031	PM04030032
27/05/15 18:05	384	432	540	192
27/05/15 18:10	432	576	708	204
27/05/15 18:15	420	552	864	240
27/05/15 18:20	516	324	816	216
27/05/15 18:25	648	492	504	156
27/05/15 18:30	468	792	468	216
27/05/15 18:35	480	492	588	204
27/05/15 18:40	480	360	768	252
27/05/15 18:45	276	636	1128	300
27/05/15 18:50	648	612	852	312
27/05/15 18:55	420	636	564	180
27/05/15 19:00	612	684	828	348
27/05/15 19:05	516	636	1200	348
27/05/15 19:10	732	504	1080	240
27/05/15 19:15	396	504	756	264
27/05/15 19:20	588	576	768	216
27/05/15 19:25	516	672	924	348
27/05/15 19:30	564	420	1020	252
27/05/15 19:35	576	324	708	240
27/05/15 19:40	408	324	504	192
27/05/15 19:45	600	348	636	264
27/05/15 19:50	324	432	612	180
27/05/15 19:55	396	240	588	204
27/05/15 20:00	384	348	600	132
27/05/15 20:05	468	252	516	84
27/05/15 20:10	384	240	480	120
27/05/15 20:15	540	324	552	168
27/05/15 20:20	396	204	564	108
27/05/15 20:25	396	324	636	192
27/05/15 20:30	456	240	432	120
27/05/15 20:35	336	360	372	60
27/05/15 20:40	552	312	648	144
27/05/15 20:45	360	396	660	156
27/05/15 20:50	444	348	480	252
27/05/15 20:55	372	240	492	60
27/05/15 21:00	396	288	360	204
27/05/15 21:05	396	312	516	120
27/05/15 21:10	432	276	528	108
27/05/15 21:15	540	348	504	120
27/05/15 21:20	432	240	528	132
27/05/15 21:25	348	288	408	108
27/05/15 21:30	432	312	648	120
27/05/15 21:35	348	252	468	96
27/05/15 21:40	468	312	504	120
27/05/15 21:45	324	252	552	144
27/05/15 21:50	372	288	480	192
27/05/15 21:55	336	192	504	96
27/05/15 22:00	324	276	576	120
27/05/15 22:05	348	312	312	108
27/05/15 22:10	348	300	324	168
27/05/15 22:15	336	216	336	96
27/05/15 22:20	432	252	516	84
27/05/15 22:25	252	276	456	120
27/05/15 22:30	336	372	444	216
27/05/15 22:35	264	288	432	96
27/05/15 22:40	312	192	516	144
27/05/15 22:45	192	228	372	120
27/05/15 22:50	336	168	372	108

Sistema de Controle de Tráfego Urbano OPTIMUS

5 MINUTOS	INTENSIDADE			
	PM 0402004	PM 0402007	PM04030031	PM04030032
27/05/15 22:55	156	180	360	84
27/05/15 23:00	300	252	216	84
27/05/15 23:05	168	204	264	96
27/05/15 23:10	204	120	336	84
27/05/15 23:15	192	72	156	24
27/05/15 23:20	204	60	240	12
27/05/15 23:25	180	72	132	36
27/05/15 23:30	180	144	240	36
27/05/15 23:35	204	84	144	36
27/05/15 23:40	132	84	204	24
27/05/15 23:45	180	108	132	60
27/05/15 23:50	144	156	228	60
27/05/15 23:55	144	72	168	24
28/05/15 00:00	228	84	336	36
28/05/15 00:05	276	72	228	72
28/05/15 00:10	288	84	240	48
28/05/15 00:15	168	72	168	48
28/05/15 00:20	180	72	252	24
28/05/15 00:25	108	72	240	12
28/05/15 00:30	216	168	204	72
28/05/15 00:35	108	84	252	36
28/05/15 00:40	120	132	300	84
28/05/15 00:45	120	96	228	36
28/05/15 00:50	132	72	180	36
28/05/15 00:55	180	132	228	48
28/05/15 01:00	96	84	252	48
28/05/15 01:05	72	84	120	36
28/05/15 01:10	84	24	168	0
28/05/15 01:15	96	48	192	12
28/05/15 01:20	120	36	120	0
28/05/15 01:25	48	84	108	24
28/05/15 01:30	48	0	84	0
28/05/15 01:35	60	24	48	12
28/05/15 01:40	72	36	84	12
28/05/15 01:45	48	24	84	12
28/05/15 01:50	72	0	60	0
28/05/15 01:55	36	36	96	24
28/05/15 02:00	36	48	132	0
28/05/15 02:05	96	12	60	12
28/05/15 02:10	84	24	60	12
28/05/15 02:15	72	24	24	0
28/05/15 02:20	72	0	72	0
28/05/15 02:25	120	60	36	0
28/05/15 02:30	60	36	72	12
28/05/15 02:35	48	12	24	0
28/05/15 02:40	48	0	24	0
28/05/15 02:45	60	0	48	0
28/05/15 02:50	12	24	72	0
28/05/15 02:55	60	24	36	0
28/05/15 03:00	72	12	48	0
28/05/15 03:05	24	0	36	0
28/05/15 03:10	24	12	60	12
28/05/15 03:15	48	24	72	12
28/05/15 03:20	24	24	12	12
28/05/15 03:25	36	24	48	0
28/05/15 03:30	12	0	12	0
28/05/15 03:35	24	0	48	0
28/05/15 03:40	12	0	48	0

Sistema de Controle de Tráfego Urbano OPTIMUS

5 MINUTOS	INTENSIDADE			
	PM 0402004	PM 0402007	PM04030031	PM04030032
28/05/15 03:45	60	24	36	0
28/05/15 03:50	12	12	36	12
28/05/15 03:55	36	12	12	0
28/05/15 04:00	12	36	36	12
28/05/15 04:05	24	0	24	0
28/05/15 04:10	84	24	48	12
28/05/15 04:15	84	12	24	0
28/05/15 04:20	48	0	36	0
28/05/15 04:25	48	36	60	12
28/05/15 04:30	84	12	12	0
28/05/15 04:35	12	24	48	12
28/05/15 04:40	24	0	12	0
28/05/15 04:45	108	12	36	0
28/05/15 04:50	24	12	36	0
28/05/15 04:55	36	12	36	0
28/05/15 05:00	36	48	48	24
28/05/15 05:05	96	0	48	0
28/05/15 05:10	108	12	36	0
28/05/15 05:15	60	12	36	0
28/05/15 05:20	84	36	72	24
28/05/15 05:25	12	60	84	24
28/05/15 05:30	48	48	84	12
28/05/15 05:35	84	48	24	24
28/05/15 05:40	108	72	84	48
28/05/15 05:45	60	48	72	24
28/05/15 05:50	120	84	180	24
28/05/15 05:55	156	72	240	48
28/05/15 06:00	168	120	324	48
28/05/15 06:05	144	84	180	36
28/05/15 06:10	180	132	300	48
28/05/15 06:15	240	96	264	36
28/05/15 06:20	252	60	348	36
28/05/15 06:25	348	120	396	48
28/05/15 06:30	396	144	624	84
28/05/15 06:35	540	252	660	60
28/05/15 06:40	708	264	792	108
28/05/15 06:45	852	288	816	216
28/05/15 06:50	828	480	936	240
28/05/15 06:55	924	648	972	324
28/05/15 07:00	1008	768	972	276
28/05/15 07:05	984	684	960	252
28/05/15 07:10	804	588	888	156
28/05/15 07:15	1116	516	744	264
28/05/15 07:20	960	504	900	108
28/05/15 07:25	828	504	756	216
28/05/15 07:30	816	588	924	156
28/05/15 07:35	972	504	840	204
28/05/15 07:40	912	300	792	168
28/05/15 07:45	1008	348	852	156
28/05/15 07:50	756	408	804	204
28/05/15 07:55	948	408	756	240
28/05/15 08:00	804	300	720	72
28/05/15 08:05	876	432	648	204
28/05/15 08:10	804	348	720	180
28/05/15 08:15	864	312	696	108
28/05/15 08:20	912	384	684	144
28/05/15 08:25	780	432	552	132
28/05/15 08:30	576	324	696	204

Sistema de Controle de Tráfego Urbano OPTIMUS

5 MINUTOS	INTENSIDADE			
	PM 0402004	PM 0402007	PM04030031	PM04030032
28/05/15 08:35	984	336	792	132
28/05/15 08:40	756	480	648	96
28/05/15 08:45	876	396	828	204
28/05/15 08:50	708	360	792	192
28/05/15 08:55	708	300	804	108
28/05/15 09:00	648	252	852	120
28/05/15 09:05	924	420	804	204
28/05/15 09:10	660	540	816	264
28/05/15 09:15	660	420	792	168
28/05/15 09:20	720	420	468	96
28/05/15 09:25	684	420	684	180
28/05/15 09:30	504	480	732	132
28/05/15 09:35	912	684	780	252
28/05/15 09:40	648	552	960	204
28/05/15 09:45	576	420	648	156
28/05/15 09:50	708	420	756	228
28/05/15 09:55	696	408	924	120
28/05/15 10:00	756	396	768	180
28/05/15 10:05	732	648	708	132
28/05/15 10:10	720	492	960	276
28/05/15 10:15	744	276	684	108
28/05/15 10:20	672	624	744	180
28/05/15 10:25	624	540	732	240
28/05/15 10:30	780	540	864	276
28/05/15 10:35	756	408	888	120
28/05/15 10:40	600	528	876	228
28/05/15 10:45	504	408	804	204
28/05/15 10:50	576	420	888	240
28/05/15 10:55	744	504	816	180
28/05/15 11:00	684	312	720	84
28/05/15 11:05	804	528	984	240
28/05/15 11:10	696	396	600	120
28/05/15 11:15	732	420	612	168
28/05/15 11:20	840	468	876	240
28/05/15 11:25	684	420	648	192
28/05/15 11:30	564	456	768	132
28/05/15 11:35	636	360	720	144
28/05/15 11:40	720	432	852	204
28/05/15 11:45	708	456	648	216
28/05/15 11:50	684	960	948	336
28/05/15 11:55	768	624	1008	312
28/05/15 12:00	552	552	984	312
28/05/15 12:05	648	552	852	204
28/05/15 12:10	528	444	648	264
28/05/15 12:15	600	576	744	276
28/05/15 12:20	552	588	744	228
28/05/15 12:25	600	432	744	252
28/05/15 12:30	444	456	780	216
28/05/15 12:35	600	516	852	264
28/05/15 12:40	780	420	768	156
28/05/15 12:45	864	384	792	252
28/05/15 12:50	588	780	864	264
28/05/15 12:55	816	540	900	264
28/05/15 13:00	816	696	948	216
28/05/15 13:05	780	792	1116	384
28/05/15 13:10	768	552	972	288
28/05/15 13:15	708	420	864	132
28/05/15 13:20	816	516	900	336

Sistema de Controle de Tráfego Urbano OPTIMUS

5 MINUTOS	INTENSIDADE			
	PM 0402004	PM 0402007	PM04030031	PM04030032
28/05/15 13:25	684	456	924	264
28/05/15 13:30	924	468	876	300
28/05/15 13:35	612	456	636	180
28/05/15 13:40	792	480	828	192
28/05/15 13:45	612	552	888	252
28/05/15 13:50	672	576	804	192
28/05/15 13:55	624	552	828	180
28/05/15 14:00	960	456	828	288
28/05/15 14:05	912	552	852	168
28/05/15 14:10	744	480	900	216
28/05/15 14:15	888	588	792	240
28/05/15 14:20	948	468	936	204
28/05/15 14:25	744	504	828	240
28/05/15 14:30	840	456	840	192
28/05/15 14:35	864	384	900	252
28/05/15 14:40	792	432	780	204
28/05/15 14:45	876	552	708	348
28/05/15 14:50	744	564	876	180
28/05/15 14:55	924	540	864	264
28/05/15 15:00	756	444	780	240
28/05/15 15:05	936	504	804	180
28/05/15 15:10	624	372	756	228
28/05/15 15:15	912	660	852	264
28/05/15 15:20	684	444	924	288
28/05/15 15:25	864	492	864	288
28/05/15 15:30	564	564	1080	216
28/05/15 15:35	828	408	744	168
28/05/15 15:40	612	672	816	288
28/05/15 15:45	696	408	696	192
28/05/15 15:50	816	492	912	144
28/05/15 15:55	816	324	648	120
28/05/15 16:00	648	408	876	96
28/05/15 16:05	792	480	708	240
28/05/15 16:10	540	396	744	204
28/05/15 16:15	684	492	948	252
28/05/15 16:20	708	432	756	216
28/05/15 16:25	744	288	660	192
28/05/15 16:30	444	360	576	204
28/05/15 16:35	636	456	696	252
28/05/15 16:40	708	552	708	288
28/05/15 16:45	660	396	768	276
28/05/15 16:50	888	612	744	240
28/05/15 16:55	696	372	768	192
28/05/15 17:00	924	348	708	192
28/05/15 17:05	828	612	732	252
28/05/15 17:10	468	492	708	264
28/05/15 17:15	612	588	876	300
28/05/15 17:20	576	528	900	156
28/05/15 17:25	588	516	852	300
28/05/15 17:30	492	684	684	300
28/05/15 17:35	468	588	1104	276
28/05/15 17:40	672	708	1044	300
28/05/15 17:45	480	384	852	168
28/05/15 17:50	684	564	1008	228
28/05/15 17:55	552	684	1008	240
28/05/15 18:00	576	672	888	300
28/05/15 18:05	384	540	924	228
28/05/15 18:10	288	408	804	276

Sistema de Controle de Tráfego Urbano OPTIMUS

5 MINUTOS	INTENSIDADE			
	PM 0402004	PM 0402007	PM 04030031	PM 04030032
28/05/15 18:15	228	708	828	180
28/05/15 18:20	552	612	948	264
28/05/15 18:25	408	504	756	228
28/05/15 18:30	348	576	804	264
28/05/15 18:35	504	444	864	216
28/05/15 18:40	432	708	792	204
28/05/15 18:45	528	684	972	252
28/05/15 18:50	672	504	888	240
28/05/15 18:55	396	552	660	300
28/05/15 19:00	468	612	744	192
28/05/15 19:05	480	408	924	312
28/05/15 19:10	384	468	924	180
28/05/15 19:15	540	444	1056	288
28/05/15 19:20	528	396	840	216
28/05/15 19:25	324	444	696	168
28/05/15 19:30	504	564	756	252
28/05/15 19:35	564	336	756	180
28/05/15 19:40	432	348	804	228
28/05/15 19:45	480	324	780	204
28/05/15 19:50	456	492	852	288
28/05/15 19:55	588	360	756	192
28/05/15 20:00	552	492	672	264
28/05/15 20:05	504	468	588	168
28/05/15 20:10	612	348	576	252
28/05/15 20:15	576	240	540	120
28/05/15 20:20	576	288	636	108
28/05/15 20:25	576	252	588	228
28/05/15 20:30	264	312	564	144
28/05/15 20:35	396	324	756	180
28/05/15 20:40	396	336	600	216
28/05/15 20:45	324	372	624	120
28/05/15 20:50	420	288	576	144
28/05/15 20:55	444	372	684	108
28/05/15 21:00	540	336	564	120
28/05/15 21:05	528	300	552	204
28/05/15 21:10	612	276	468	180
28/05/15 21:15	408	324	516	192
28/05/15 21:20	648	276	600	204
28/05/15 21:25	588	204	552	132
28/05/15 21:30	384	276	528	120
28/05/15 21:35	324	288	516	192
28/05/15 21:40	444	300	480	132
28/05/15 21:45	576	264	480	96
28/05/15 21:50	348	228	444	120
28/05/15 21:55	384	312	540	108
28/05/15 22:00	420	276	432	144
28/05/15 22:05	432	312	564	144
28/05/15 22:10	372	324	540	240
28/05/15 22:15	576	288	528	144
28/05/15 22:20	528	324	636	180
28/05/15 22:25	432	276	480	156
28/05/15 22:30	336	384	552	144
28/05/15 22:35	420	384	636	168
28/05/15 22:40	408	276	444	96
28/05/15 22:45	456	384	456	252
28/05/15 22:50	360	288	456	144
28/05/15 22:55	252	228	444	120
28/05/15 23:00	372	168	420	84

5 MINUTOS	INTENSIDADE			
	PM 0402004	PM 0402007	PM04030031	PM04030032
28/05/15 23:05	348	132	468	84
28/05/15 23:10	168	216	264	84
28/05/15 23:15	276	132	384	48
28/05/15 23:20	204	168	252	72
28/05/15 23:25	288	96	300	48
28/05/15 23:30	252	156	192	48
28/05/15 23:35	288	108	192	84
28/05/15 23:40	204	60	156	36
28/05/15 23:45	228	108	288	24
28/05/15 23:50	168	144	240	48
28/05/15 23:55	264	96	180	36
29/05/15 00:00	192	24	216	36
29/05/15 00:05	204	108	204	24
29/05/15 00:10	216	60	144	12
29/05/15 00:15	192	108	180	48
29/05/15 00:20	120	72	108	36
29/05/15 00:25	180	84	180	24
29/05/15 00:30	144	84	180	24
29/05/15 00:35	144	108	156	48
29/05/15 00:40	120	60	144	24
29/05/15 00:45	156	108	132	12
29/05/15 00:50	192	120	204	36
29/05/15 00:55	204	120	144	48
29/05/15 01:00	156	96	132	36
29/05/15 01:05	132	12	96	0
29/05/15 01:10	72	12	48	12
29/05/15 01:15	144	84	72	12
29/05/15 01:20	180	36	96	12
29/05/15 01:25	144	48	72	24
29/05/15 01:30	120	36	96	0
29/05/15 01:35	36	24	72	24
29/05/15 01:40	60	12	84	12
29/05/15 01:45	96	36	36	24
29/05/15 01:50	60	12	48	12
29/05/15 01:55	120	0	36	0
29/05/15 02:00	84	0	84	12
29/05/15 02:05	60	48	48	0
29/05/15 02:10	60	60	60	48
29/05/15 02:15	60	12	48	12
29/05/15 02:20	36	36	60	24
29/05/15 02:25	108	12	24	0
29/05/15 02:30	48	12	48	0
29/05/15 02:35	36	12	12	0
29/05/15 02:40	36	24	60	0
29/05/15 02:45	48	12	24	12
29/05/15 02:50	60	0	24	0
29/05/15 02:55	72	12	48	0
29/05/15 03:00	24	12	60	0
29/05/15 03:05	60	0	48	0
29/05/15 03:10	96	12	24	12
29/05/15 03:15	48	12	84	12
29/05/15 03:20	48	12	12	12
29/05/15 03:25	48	0	24	0
29/05/15 03:30	12	12	12	12
29/05/15 03:35	72	24	72	0
29/05/15 03:40	0	0	48	0
29/05/15 03:45	48	0	60	0
29/05/15 03:50	24	0	12	0

Sistema de Controle de Tráfego Urbano OPTIMUS

5 MINUTOS	INTENSIDADE			
	PM 0402004	PM 0402007	PM04030031	PM04030032
29/05/15 03:55	60	0	12	0
29/05/15 04:00	24	0	12	0
29/05/15 04:05	12	36	24	0
29/05/15 04:10	12	0	0	12
29/05/15 04:15	36	12	12	0
29/05/15 04:20	36	12	36	0
29/05/15 04:25	36	36	48	12
29/05/15 04:30	72	24	48	12
29/05/15 04:35	60	12	60	0
29/05/15 04:40	60	24	36	0
29/05/15 04:45	60	36	24	12
29/05/15 04:50	36	24	72	0
29/05/15 04:55	36	0	12	0
29/05/15 05:00	72	48	12	36
29/05/15 05:05	108	12	12	0
29/05/15 05:10	84	60	36	12
29/05/15 05:15	96	0	24	0
29/05/15 05:20	84	36	24	0
29/05/15 05:25	108	36	36	24
29/05/15 05:30	132	48	120	36
29/05/15 05:35	120	60	60	24
29/05/15 05:40	96	60	144	24
29/05/15 05:45	96	84	120	72
29/05/15 05:50	192	84	180	0
29/05/15 05:55	108	36	168	24
29/05/15 06:00	216	36	252	12
29/05/15 06:05	180	84	264	48
29/05/15 06:10	168	96	264	12
29/05/15 06:15	204	216	336	12
29/05/15 06:20	312	120	324	48
29/05/15 06:25	408	228	444	120
29/05/15 06:30	348	240	648	144
29/05/15 06:35	504	144	408	36
29/05/15 06:40	732	204	552	120
29/05/15 06:45	828	372	732	156
29/05/15 06:50	888	360	852	156
29/05/15 06:55	1020	480	924	180
29/05/15 07:00	1044	432	804	240
29/05/15 07:05	1044	780	972	192
29/05/15 07:10	948	528	972	156
29/05/15 07:15	1008	660	804	252
29/05/15 07:20	936	612	984	192
29/05/15 07:25	1308	588	780	240
29/05/15 07:30	1056	408	840	132
29/05/15 07:35	1152	432	732	204
29/05/15 07:40	1068	372	660	156
29/05/15 07:45	684	348	624	192
29/05/15 07:50	972	384	660	156
29/05/15 07:55	744	396	600	156
29/05/15 08:00	912	480	480	204
29/05/15 08:05	696	336	600	156
29/05/15 08:10	1164	372	720	216
29/05/15 08:15	372	252	468	144
29/05/15 08:20	1020	672	720	216
29/05/15 08:25	1128	396	744	168
29/05/15 08:30	1044	240	516	168
29/05/15 08:35	768	168	528	60
29/05/15 08:40	696	300	612	180

Sistema de Controle de Tráfego Urbano OPTIMUS

5 MINUTOS	INTENSIDADE			
	PM 0402004	PM 0402007	PM 04030031	PM 04030032
29/05/15 08:45	972	264	840	96
29/05/15 08:50	852	252	660	132
29/05/15 08:55	1164	408	588	132
29/05/15 09:00	984	168	432	156
29/05/15 09:05	1020	216	636	48
29/05/15 09:10	708	252	504	144
29/05/15 09:15	876	132	612	96
29/05/15 09:20	888	336	516	156
29/05/15 09:25	804	216	660	96
29/05/15 09:30	624	252	708	168
29/05/15 09:35	684	612	936	216
29/05/15 09:40	804	540	900	156
29/05/15 09:45	732	552	792	228
29/05/15 09:50	1068	420	828	156
29/05/15 09:55	996	492	912	132
29/05/15 10:00	804	444	1092	264
29/05/15 10:05	732	600	852	180
29/05/15 10:10	768	456	1008	276
29/05/15 10:15	816	420	816	264
29/05/15 10:20	732	552	972	168
29/05/15 10:25	732	408	780	204
29/05/15 10:30	684	480	984	204
29/05/15 10:35	732	396	936	156
29/05/15 10:40	564	456	744	192
29/05/15 10:45	936	696	888	276
29/05/15 10:50	708	468	912	120
29/05/15 10:55	720	612	780	288
29/05/15 11:00	756	552	888	132
29/05/15 11:05	636	312	732	276
29/05/15 11:10	768	576	624	276
29/05/15 11:15	624	708	864	348
29/05/15 11:20	828	636	804	264
29/05/15 11:25	828	672	852	228
29/05/15 11:30	672	564	924	264
29/05/15 11:35	684	564	732	216
29/05/15 11:40	648	612	816	168
29/05/15 11:45	732	780	912	276
29/05/15 11:50	660	756	924	252
29/05/15 11:55	792	852	960	348
29/05/15 12:00	804	924	1056	336
29/05/15 12:05	792	612	1116	312
29/05/15 12:10	684	672	948	348
29/05/15 12:15	816	744	1032	312
29/05/15 12:20	780	552	732	192
29/05/15 12:25	540	660	912	252
29/05/15 12:30	804	660	876	264
29/05/15 12:35	648	732	912	288
29/05/15 12:40	816	624	996	300
29/05/15 12:45	816	576	1092	204
29/05/15 12:50	864	672	816	276
29/05/15 12:55	804	660	912	276
29/05/15 13:00	828	828	984	300
29/05/15 13:05	672	612	1080	264
29/05/15 13:10	780	792	1080	336
29/05/15 13:15	840	576	948	360
29/05/15 13:20	924	540	924	348
29/05/15 13:25	744	600	1020	312
29/05/15 13:30	624	696	816	216

Sistema de Controle de Tráfego Urbano OPTIMUS

5 MINUTOS	INTENSIDADE			
	PM 0402004	PM 0402007	PM04030031	PM04030032
29/05/15 13:35	852	480	924	240
29/05/15 13:40	840	624	696	204
29/05/15 13:45	612	324	852	312
29/05/15 13:50	744	612	900	180
29/05/15 13:55	672	492	840	228
29/05/15 14:00	804	480	780	216
29/05/15 14:05	840	468	852	84
29/05/15 14:10	660	504	720	216
29/05/15 14:15	984	588	1032	312
29/05/15 14:20	732	468	900	276
29/05/15 14:25	708	480	792	276
29/05/15 14:30	888	600	828	252
29/05/15 14:35	720	540	972	204
29/05/15 14:40	876	612	876	252
29/05/15 14:45	852	684	840	300
29/05/15 14:50	792	372	660	180
29/05/15 14:55	684	660	720	204
29/05/15 15:00	864	528	1044	276
29/05/15 15:05	912	444	696	192
29/05/15 15:10	852	492	804	216
29/05/15 15:15	720	636	960	312
29/05/15 15:20	576	648	876	240
29/05/15 15:25	816	492	912	216
29/05/15 15:30	696	444	876	216
29/05/15 15:35	840	708	840	312
29/05/15 15:40	1032	588	684	180
29/05/15 15:45	648	432	732	288
29/05/15 15:50	840	696	684	180
29/05/15 15:55	828	492	948	228
29/05/15 16:00	912	360	780	216
29/05/15 16:05	648	528	1008	228
29/05/15 16:10	840	420	828	204
29/05/15 16:15	816	552	768	264
29/05/15 16:20	780	576	756	300
29/05/15 16:25	768	636	1020	324
29/05/15 16:30	636	636	804	204
29/05/15 16:35	852	576	804	240
29/05/15 16:40	792	480	900	276
29/05/15 16:45	744	612	1008	240
29/05/15 16:50	672	624	828	288
29/05/15 16:55	636	600	996	300
29/05/15 17:00	624	672	684	360
29/05/15 17:05	252	660	360	228
29/05/15 17:10	396	192	480	276
29/05/15 17:15	216	300	624	120
29/05/15 17:20	240	312	528	192
29/05/15 17:25	180	252	468	120
29/05/15 17:30	468	600	756	276
29/05/15 17:35	516	432	996	180
29/05/15 17:40	396	312	504	132
29/05/15 17:45	780	408	468	168
29/05/15 17:50	444	672	552	168
29/05/15 17:55	528	384	564	168
29/05/15 18:00	444	396	456	192
29/05/15 18:05	468	324	576	192
29/05/15 18:10	444	564	1104	276
29/05/15 18:15	348	384	792	144
29/05/15 18:20	516	432	384	156

Sistema de Controle de Tráfego Urbano OPTIMUS

5 MINUTOS	INTENSIDADE			
	PM 0402004	PM 0402007	PM 04030031	PM 04030032
29/05/15 18:25	168	216	372	156
29/05/15 18:30	456	456	384	228
29/05/15 18:35	252	264	348	108
29/05/15 18:40	516	276	756	192
29/05/15 18:45	204	420	948	252
29/05/15 18:50	420	336	636	252
29/05/15 18:55	492	264	576	144
29/05/15 19:00	384	372	516	240
29/05/15 19:05	336	564	528	120
29/05/15 19:10	468	432	336	240
29/05/15 19:15	396	408	636	84
29/05/15 19:20	492	408	840	288
29/05/15 19:25	372	528	852	216
29/05/15 19:30	420	360	780	180
29/05/15 19:35	588	540	792	180
29/05/15 19:40	408	456	780	216
29/05/15 19:45	408	444	828	204
29/05/15 19:50	636	468	1164	228
29/05/15 19:55	516	672	1068	348
29/05/15 20:00	528	756	1284	348
29/05/15 20:05	564	888	972	324
29/05/15 20:10	456	588	888	372
29/05/15 20:15	624	516	1020	204
29/05/15 20:20	576	456	732	252
29/05/15 20:25	708	348	600	192
29/05/15 20:30	756	300	804	204
29/05/15 20:35	636	372	852	180
29/05/15 20:40	588	540	756	324
29/05/15 20:45	756	468	624	240
29/05/15 20:50	600	444	588	180
29/05/15 20:55	744	384	648	228
29/05/15 21:00	504	432	672	180
29/05/15 21:05	540	432	804	180
29/05/15 21:10	636	336	756	144
29/05/15 21:15	696	504	528	324
29/05/15 21:20	588	336	804	204
29/05/15 21:25	468	396	648	132
29/05/15 21:30	456	348	792	120
29/05/15 21:35	480	468	612	156
29/05/15 21:40	468	348	588	156
29/05/15 21:45	504	288	720	156
29/05/15 21:50	564	396	612	264
29/05/15 21:55	588	288	744	204
29/05/15 22:00	456	276	504	96
29/05/15 22:05	468	384	696	144
29/05/15 22:10	480	300	720	180
29/05/15 22:15	516	276	480	144
29/05/15 22:20	612	372	852	132
29/05/15 22:25	564	312	636	180
29/05/15 22:30	540	336	576	72
29/05/15 22:35	576	180	576	84
29/05/15 22:40	456	360	576	132
29/05/15 22:45	432	300	540	156
29/05/15 22:50	444	324	576	132
29/05/15 22:55	372	204	564	120
29/05/15 23:00	540	180	348	60
29/05/15 23:05	492	324	552	108
29/05/15 23:10	372	300	420	180

Sistema de Controle de Tráfego Urbano OPTIMUS

5 MINUTOS	INTENSIDADE			
	PM 0402004	PM 0402007	PM04030031	PM04030032
29/05/15 23:15	552	168	576	108
29/05/15 23:20	492	180	216	36
29/05/15 23:25	360	240	396	60
29/05/15 23:30	252	264	348	84
29/05/15 23:35	360	252	396	108
29/05/15 23:40	360	216	300	96
29/05/15 23:45	444	144	348	72
29/05/15 23:50	312	216	312	84
29/05/15 23:55	360	168	348	72
30/05/15 00:00	228	180	276	72
30/05/15 00:05	348	204	336	108
30/05/15 00:10	312	132	312	72
30/05/15 00:15	444	120	348	36
30/05/15 00:20	240	120	288	60
30/05/15 00:25	372	132	372	36
30/05/15 00:30	264	240	276	72
30/05/15 00:35	336	228	348	84
30/05/15 00:40	456	156	444	72
30/05/15 00:45	360	144	348	36
30/05/15 00:50	216	96	240	24
30/05/15 00:55	180	132	228	48
30/05/15 01:00	348	84	108	84
30/05/15 01:05	228	192	300	60
30/05/15 01:10	228	84	144	48
30/05/15 01:15	192	96	180	12
30/05/15 01:20	216	156	360	24
30/05/15 01:25	240	84	216	24
30/05/15 01:30	180	72	228	24
30/05/15 01:35	228	108	132	12
30/05/15 01:40	180	168	252	24
30/05/15 01:45	204	96	264	84
30/05/15 01:50	96	96	216	48
30/05/15 01:55	144	108	168	12
30/05/15 02:00	264	84	156	0
30/05/15 02:05	336	48	132	0
30/05/15 02:10	144	72	144	12
30/05/15 02:15	264	96	180	24
30/05/15 02:20	84	84	204	24
30/05/15 02:25	204	48	72	0
30/05/15 02:30	192	48	72	12
30/05/15 02:35	144	48	168	0
30/05/15 02:40	168	84	168	24
30/05/15 02:45	96	96	84	36
30/05/15 02:50	144	84	132	60
30/05/15 02:55	144	24	108	0
30/05/15 03:00	108	36	108	24
30/05/15 03:05	252	24	156	12
30/05/15 03:10	168	36	108	0
30/05/15 03:15	108	72	168	24
30/05/15 03:20	168	36	156	24
30/05/15 03:25	132	60	132	24
30/05/15 03:30	108	12	96	12
30/05/15 03:35	84	36	60	0
30/05/15 03:40	120	72	156	0
30/05/15 03:45	144	36	84	12
30/05/15 03:50	48	24	156	0
30/05/15 03:55	72	60	132	36
30/05/15 04:00	84	60	60	12

Sistema de Controle de Tráfego Urbano OPTIMUS

5 MINUTOS	INTENSIDADE			
	PM 0402004	PM 0402007	PM04030031	PM04030032
30/05/15 04:05	120	12	84	12
30/05/15 04:10	84	36	132	0
30/05/15 04:15	108	36	60	12
30/05/15 04:20	108	24	108	0
30/05/15 04:25	168	24	60	0
30/05/15 04:30	96	36	72	0
30/05/15 04:35	120	48	36	0
30/05/15 04:40	144	36	96	24
30/05/15 04:45	120	24	72	12
30/05/15 04:50	120	60	36	36
30/05/15 04:55	132	36	60	12
30/05/15 05:00	72	72	84	36
30/05/15 05:05	48	0	72	0
30/05/15 05:10	132	12	72	12
30/05/15 05:15	120	72	60	24
30/05/15 05:20	144	60	84	12
30/05/15 05:25	120	36	48	0
30/05/15 05:30	96	48	36	0
30/05/15 05:35	120	0	120	12
30/05/15 05:40	108	48	96	36
30/05/15 05:45	96	84	108	48
30/05/15 05:50	144	36	72	36
30/05/15 05:55	72	12	48	0
30/05/15 06:00	180	48	36	24
30/05/15 06:05	180	48	120	36
30/05/15 06:10	240	48	228	0
30/05/15 06:15	144	84	156	36
30/05/15 06:20	168	72	192	36
30/05/15 06:25	156	84	168	48
30/05/15 06:30	312	24	156	0
30/05/15 06:35	180	84	204	36
30/05/15 06:40	252	108	228	60
30/05/15 06:45	228	72	216	48
30/05/15 06:50	252	180	264	24
30/05/15 06:55	276	180	360	48
30/05/15 07:00	288	108	168	36
30/05/15 07:05	324	156	252	48
30/05/15 07:10	372	156	348	72
30/05/15 07:15	408	192	348	84
30/05/15 07:20	276	144	324	72
30/05/15 07:25	384	240	156	120
30/05/15 07:30	444	120	336	60
30/05/15 07:35	480	216	288	60
30/05/15 07:40	456	108	588	72
30/05/15 07:45	528	192	312	48
30/05/15 07:50	492	168	456	60
30/05/15 07:55	600	312	552	132
30/05/15 08:00	540	312	600	84
30/05/15 08:05	576	372	528	108
30/05/15 08:10	552	408	648	120
30/05/15 08:15	756	216	588	132
30/05/15 08:20	480	180	600	96
30/05/15 08:25	540	312	540	120
30/05/15 08:30	576	216	624	96
30/05/15 08:35	552	264	432	156
30/05/15 08:40	672	192	528	156
30/05/15 08:45	564	324	504	168
30/05/15 08:50	444	360	792	96

Sistema de Controle de Tráfego Urbano OPTIMUS

5 MINUTOS	INTENSIDADE			
	PM 0402004	PM 0402007	PM04030031	PM04030032
30/05/15 08:55	708	300	576	156
30/05/15 09:00	684	360	756	60
30/05/15 09:05	588	396	768	228
30/05/15 09:10	636	360	804	180
30/05/15 09:15	744	252	516	84
30/05/15 09:20	636	504	804	228
30/05/15 09:25	660	348	756	240
30/05/15 09:30	660	420	732	84
30/05/15 09:35	672	372	696	252
30/05/15 09:40	636	420	660	216
30/05/15 09:45	744	468	624	252
30/05/15 09:50	612	396	792	108
30/05/15 09:55	780	420	672	240
30/05/15 10:00	708	336	768	168
30/05/15 10:05	792	312	732	180
30/05/15 10:10	576	492	696	156
30/05/15 10:15	996	444	612	276
30/05/15 10:20	600	552	720	228
30/05/15 10:25	792	552	768	240
30/05/15 10:30	576	264	876	156
30/05/15 10:35	720	588	696	216
30/05/15 10:40	588	408	672	228
30/05/15 10:45	744	372	732	216
30/05/15 10:50	768	468	936	240
30/05/15 10:55	876	348	732	288
30/05/15 11:00	732	492	864	144
30/05/15 11:05	936	468	816	252
30/05/15 11:10	660	540	768	252
30/05/15 11:15	864	600	852	288
30/05/15 11:20	828	420	972	216
30/05/15 11:25	936	564	792	180
30/05/15 11:30	684	456	840	192
30/05/15 11:35	816	444	792	252
30/05/15 11:40	720	492	780	228
30/05/15 11:45	660	576	840	276
30/05/15 11:50	660	552	888	264
30/05/15 11:55	792	744	792	240
30/05/15 12:00	684	696	924	324
30/05/15 12:05	804	612	852	288
30/05/15 12:10	732	564	1044	264
30/05/15 12:15	828	564	948	300
30/05/15 12:20	612	360	960	276
30/05/15 12:25	864	516	1056	288
30/05/15 12:45	312	264	480	156
30/05/15 12:50	624	468	1152	288
30/05/15 12:55	540	420	744	240
30/05/15 13:00	852	372	1008	324
30/05/15 13:05	624	564	960	228
30/05/15 13:10	660	516	1044	264
30/05/15 13:15	528	780	984	360
30/05/15 13:20	708	540	1032	252
30/05/15 13:25	708	516	912	336
30/05/15 13:30	744	648	1008	228
30/05/15 13:35	612	624	816	372
30/05/15 13:40	648	432	984	264
30/05/15 13:45	516	396	768	264
30/05/15 13:50	564	444	1104	288
30/05/15 13:55	528	372	804	324

Sistema de Controle de Tráfego Urbano OPTIMUS

5 MINUTOS	INTENSIDADE			
	PM 0402004	PM 0402007	PM04030031	PM04030032
30/05/15 14:00	768	492	780	240
30/05/15 14:05	480	420	708	324
30/05/15 14:10	636	432	660	180
30/05/15 14:15	504	468	996	228
30/05/15 14:20	720	324	996	264
30/05/15 14:25	576	372	720	240
30/05/15 14:30	828	396	864	228
30/05/15 14:35	480	420	780	228
30/05/15 14:40	564	384	732	144
30/05/15 14:45	588	552	696	252
30/05/15 14:50	708	216	792	156
30/05/15 14:55	432	348	552	204
30/05/15 15:00	792	276	600	96
30/05/15 15:05	504	384	744	228
30/05/15 15:10	804	288	804	168
30/05/15 15:15	720	408	708	168
30/05/15 15:20	696	372	780	96
30/05/15 15:25	600	312	708	228
30/05/15 15:30	624	288	492	156
30/05/15 15:35	540	324	576	132
30/05/15 15:40	588	324	672	168
30/05/15 15:45	612	348	660	156
30/05/15 15:50	540	216	528	192
30/05/15 15:55	564	336	660	72
30/05/15 16:00	480	252	492	192
30/05/15 16:05	564	420	696	204
30/05/15 16:10	600	324	792	192
30/05/15 16:15	612	420	720	192
30/05/15 16:20	636	252	684	192
30/05/15 16:25	480	360	768	168
30/05/15 16:30	516	312	624	144
30/05/15 16:35	780	360	648	132
30/05/15 16:40	696	468	660	144
30/05/15 16:45	624	432	744	180
30/05/15 16:50	588	384	768	228
30/05/15 16:55	732	384	636	204
30/05/15 17:00	648	360	876	276
30/05/15 17:05	636	348	564	120
30/05/15 17:10	636	348	672	180
30/05/15 17:15	588	276	720	144
30/05/15 17:20	456	408	588	132
30/05/15 17:25	648	276	744	240
30/05/15 17:30	564	384	648	156
30/05/15 17:35	720	336	576	240
30/05/15 17:40	528	348	636	216
30/05/15 17:45	528	264	756	96
30/05/15 17:50	660	348	624	156
30/05/15 17:55	468	408	816	180
30/05/15 18:00	636	324	648	204
30/05/15 18:05	660	444	636	156
30/05/15 18:10	612	384	708	264
30/05/15 18:15	768	372	804	192
30/05/15 18:20	648	288	744	228
30/05/15 18:25	732	324	780	108
30/05/15 18:30	540	504	612	180
30/05/15 18:35	588	216	660	192
30/05/15 18:40	540	420	732	120
30/05/15 18:45	648	240	756	192

Sistema de Controle de Tráfego Urbano OPTIMUS

5 MINUTOS	INTENSIDADE			
	PM 0402004	PM 0402007	PM04030031	PM04030032
30/05/15 18:50	684	288	648	108
30/05/15 18:55	528	312	792	144
30/05/15 19:00	624	288	612	216
30/05/15 19:05	648	216	660	84
30/05/15 19:10	636	348	576	168
30/05/15 19:15	540	360	876	120
30/05/15 19:20	552	312	552	192
30/05/15 19:25	564	336	660	132
30/05/15 19:30	648	300	636	168
30/05/15 19:35	696	360	876	180
30/05/15 19:40	480	312	552	192
30/05/15 19:45	432	276	864	192
30/05/15 19:50	468	288	636	132
30/05/15 19:55	588	336	696	144
30/05/15 20:00	528	324	780	180
30/05/15 20:05	492	348	828	204
30/05/15 20:10	564	384	768	168
30/05/15 20:15	804	168	732	144
30/05/15 20:20	540	288	588	132
30/05/15 20:25	624	480	564	204
30/05/15 20:30	708	444	876	192
30/05/15 20:35	456	384	840	144
30/05/15 20:40	564	420	564	204
30/05/15 20:45	624	384	852	168
30/05/15 20:50	540	324	564	180
30/05/15 20:55	480	300	600	60
30/05/15 21:00	564	348	672	192
30/05/15 21:05	744	288	600	108
30/05/15 21:10	648	324	576	60
30/05/15 21:15	600	276	612	156
30/05/15 21:20	468	204	540	168
30/05/15 21:25	576	240	468	120
30/05/15 21:30	516	276	696	108
30/05/15 21:35	468	312	396	84
30/05/15 21:40	576	408	564	180
30/05/15 21:45	576	288	420	192
30/05/15 21:50	492	240	624	156
30/05/15 21:55	516	312	588	108
30/05/15 22:00	444	204	504	72
30/05/15 22:05	576	192	660	60
30/05/15 22:10	480	204	468	120
30/05/15 22:15	492	288	528	120
30/05/15 22:20	540	180	492	192
30/05/15 22:25	588	240	432	144
30/05/15 22:30	552	192	480	60
30/05/15 22:35	540	288	492	132
30/05/15 22:40	624	228	468	144
30/05/15 22:45	576	192	552	132
30/05/15 22:50	432	168	456	72
30/05/15 22:55	660	276	384	84
30/05/15 23:00	444	204	360	72
30/05/15 23:05	516	132	432	48
30/05/15 23:10	480	264	528	144
30/05/15 23:15	564	132	384	60
30/05/15 23:20	372	240	372	108
30/05/15 23:25	372	180	492	84
30/05/15 23:30	672	168	360	36
30/05/15 23:35	396	216	360	60

Sistema de Controle de Tráfego Urbano OPTIMUS

5 MINUTOS	INTENSIDADE			
	PM 0402004	PM 0402007	PM04030031	PM04030032
30/05/15 23:40	276	252	384	96
30/05/15 23:45	480	168	420	36
30/05/15 23:50	408	192	372	36
30/05/15 23:55	408	264	360	108
31/05/15 00:00	348	132	336	60
31/05/15 00:05	372	216	336	48
31/05/15 00:10	360	120	312	84
31/05/15 00:15	372	156	336	84
31/05/15 00:20	396	204	276	60
31/05/15 00:25	336	228	432	24
31/05/15 00:30	372	192	396	84
31/05/15 00:35	228	192	372	72
31/05/15 00:40	348	168	336	60
31/05/15 00:45	312	108	312	36
31/05/15 00:50	384	108	240	12
31/05/15 00:55	372	144	216	120
31/05/15 01:00	408	96	324	48
31/05/15 01:05	468	168	408	36
31/05/15 01:10	312	108	240	36
31/05/15 01:15	360	96	348	36
31/05/15 01:20	288	120	288	36
31/05/15 01:25	408	60	300	24
31/05/15 01:30	240	120	156	48
31/05/15 01:35	348	132	420	36
31/05/15 01:40	384	156	252	36
31/05/15 01:45	240	48	144	24
31/05/15 01:50	276	120	204	36
31/05/15 01:55	288	108	240	48
31/05/15 02:00	216	72	192	48
31/05/15 02:05	240	96	228	24
31/05/15 02:10	324	84	168	36
31/05/15 02:15	180	168	288	36
31/05/15 02:20	204	72	96	0
31/05/15 02:25	216	60	72	24
31/05/15 02:30	336	72	192	24
31/05/15 02:35	180	60	132	24
31/05/15 02:40	132	60	240	0
31/05/15 02:45	216	108	144	0
31/05/15 02:50	204	120	216	48
31/05/15 02:55	180	36	84	48
31/05/15 03:00	192	96	120	36
31/05/15 03:05	180	48	168	12
31/05/15 03:10	216	72	192	24
31/05/15 03:15	144	36	144	12
31/05/15 03:20	204	96	300	12
31/05/15 03:25	108	84	204	24
31/05/15 03:30	132	36	252	24
31/05/15 03:35	144	60	120	24
31/05/15 03:40	108	12	96	0
31/05/15 03:45	168	96	120	36
31/05/15 03:50	144	96	180	0
31/05/15 03:55	144	96	204	24
31/05/15 04:00	84	36	108	12
31/05/15 04:05	180	24	96	12
31/05/15 04:10	96	84	120	12
31/05/15 04:15	192	36	156	12
31/05/15 04:20	144	48	108	12
31/05/15 04:25	132	72	144	0

Sistema de Controle de Tráfego Urbano OPTIMUS

5 MINUTOS	INTENSIDADE			
	PM 0402004	PM 0402007	PM04030031	PM04030032
31/05/15 04:30	168	60	156	12
31/05/15 04:35	180	60	72	0
31/05/15 04:40	108	36	72	0
31/05/15 04:45	120	24	132	0
31/05/15 04:50	48	12	60	0
31/05/15 04:55	96	36	96	0
31/05/15 05:00	120	108	144	60
31/05/15 05:05	108	48	48	12
31/05/15 05:10	96	36	120	0
31/05/15 05:15	144	36	84	0
31/05/15 05:20	96	36	60	0
31/05/15 05:25	96	48	48	0
31/05/15 05:30	216	48	96	12
31/05/15 05:35	156	36	60	24
31/05/15 05:40	84	12	72	0
31/05/15 05:45	108	48	60	12
31/05/15 05:50	168	12	72	0
31/05/15 05:55	108	12	108	36
31/05/15 06:00	168	36	84	12
31/05/15 06:05	108	84	156	12
31/05/15 06:10	96	60	36	12
31/05/15 06:15	108	84	132	36
31/05/15 06:20	192	60	72	60
31/05/15 06:25	156	84	168	12
31/05/15 06:30	108	48	120	36
31/05/15 06:35	96	96	168	36
31/05/15 06:40	144	72	180	48
31/05/15 06:45	192	48	180	36
31/05/15 06:50	120	72	216	24
31/05/15 06:55	96	36	120	24
31/05/15 07:00	204	132	204	24
31/05/15 07:05	276	84	192	12
31/05/15 07:10	312	60	132	24
31/05/15 07:15	168	120	168	96
31/05/15 07:20	264	84	228	36
31/05/15 07:25	228	60	192	36
31/05/15 07:30	180	72	240	36
31/05/15 07:35	228	120	168	36
31/05/15 07:40	252	84	192	24
31/05/15 07:45	300	120	240	48
31/05/15 07:50	336	96	192	48
31/05/15 07:55	144	192	228	48
31/05/15 08:00	180	60	132	36
31/05/15 08:05	264	120	264	60
31/05/15 08:10	312	144	252	48
31/05/15 08:15	204	72	312	36
31/05/15 08:20	348	120	252	60
31/05/15 08:25	264	156	300	12
31/05/15 08:30	324	84	300	36
31/05/15 08:35	264	192	300	120
31/05/15 08:40	300	192	312	108
31/05/15 08:45	504	144	420	72
31/05/15 08:50	312	168	420	36
31/05/15 08:55	468	192	360	60
31/05/15 09:00	324	180	468	36
31/05/15 09:05	324	192	324	48
31/05/15 09:10	408	204	396	72
31/05/15 09:15	204	120	276	60

Sistema de Controle de Tráfego Urbano OPTIMUS

5 MINUTOS	INTENSIDADE			
	PM 0402004	PM 0402007	PM04030031	PM04030032
31/05/15 09:20	384	72	408	84
31/05/15 09:25	384	216	408	84
31/05/15 09:30	492	216	324	96
31/05/15 09:35	372	96	360	48
31/05/15 09:40	324	240	456	60
31/05/15 09:45	264	300	420	108
31/05/15 09:50	408	144	372	60
31/05/15 09:55	324	288	336	120
31/05/15 10:00	276	192	420	72
31/05/15 10:05	312	180	432	48
31/05/15 10:10	348	192	432	144
31/05/15 10:15	336	252	300	120
31/05/15 10:20	504	336	492	144
31/05/15 10:25	408	180	516	120
31/05/15 10:30	456	288	552	180
31/05/15 10:35	360	312	468	120
31/05/15 10:40	456	276	468	132
31/05/15 10:45	600	300	600	84
31/05/15 10:50	456	156	540	84
31/05/15 10:55	348	180	480	72
31/05/15 11:00	420	288	540	132
31/05/15 11:05	528	324	540	120
31/05/15 11:10	480	324	324	132
31/05/15 11:15	504	312	564	192
31/05/15 11:20	348	216	576	156
31/05/15 11:25	600	288	480	48
31/05/15 11:30	444	264	492	120
31/05/15 11:35	516	288	432	216
31/05/15 11:40	432	216	552	96
31/05/15 11:45	552	276	516	156
31/05/15 11:50	564	264	492	180
31/05/15 11:55	564	264	636	108
31/05/15 12:00	672	288	540	168
31/05/15 12:05	504	420	660	144
31/05/15 12:10	408	324	672	144
31/05/15 12:15	564	348	696	132
31/05/15 12:20	516	312	840	156
31/05/15 12:25	816	384	792	192
31/05/15 12:30	672	228	576	144
31/05/15 12:35	408	360	912	228
31/05/15 12:40	516	456	816	120
31/05/15 12:45	444	264	816	108
31/05/15 12:50	480	396	744	144
31/05/15 12:55	348	288	900	168
31/05/15 13:00	372	348	684	216
31/05/15 13:05	444	432	684	144
31/05/15 13:10	528	276	732	168
31/05/15 13:15	420	384	588	120
31/05/15 13:20	516	372	624	192
31/05/15 13:25	576	204	636	156
31/05/15 13:30	504	228	612	108
31/05/15 13:35	540	336	588	84
31/05/15 13:40	612	276	552	180
31/05/15 13:45	600	456	720	216
31/05/15 13:50	540	228	696	108
31/05/15 13:55	540	288	624	156
31/05/15 14:00	432	264	588	108
31/05/15 14:05	492	228	576	144

Sistema de Controle de Tráfego Urbano OPTIMUS

5 MINUTOS	INTENSIDADE			
	PM 0402004	PM 0402007	PM 04030031	PM 04030032
31/05/15 14:10	480	300	468	144
31/05/15 14:15	396	156	528	156
31/05/15 14:20	300	312	504	120
31/05/15 14:25	432	300	504	228
31/05/15 14:30	480	288	504	72
31/05/15 14:35	432	216	576	192
31/05/15 14:40	468	324	732	144
31/05/15 14:45	444	276	432	108
31/05/15 14:50	492	420	696	120
31/05/15 14:55	468	192	576	144
31/05/15 15:00	420	264	588	180
31/05/15 15:05	504	264	660	120
31/05/15 15:10	528	372	696	204
31/05/15 15:15	684	204	624	108
31/05/15 15:20	348	264	768	144
31/05/15 15:25	432	264	636	132
31/05/15 15:30	516	288	720	132
31/05/15 15:35	528	180	636	84
31/05/15 15:40	552	252	624	108
31/05/15 15:45	408	324	552	108
31/05/15 15:50	564	144	624	132
31/05/15 15:55	624	216	564	72
31/05/15 16:00	576	252	576	48
31/05/15 16:05	504	192	732	120
31/05/15 16:10	588	168	576	108
31/05/15 16:15	600	336	564	120
31/05/15 16:20	516	228	588	72
31/05/15 16:25	576	180	444	144
31/05/15 16:30	552	396	408	132
31/05/15 16:35	420	348	360	156
31/05/15 16:40	576	216	648	120
31/05/15 16:45	396	132	456	168
31/05/15 16:50	636	144	408	120
31/05/15 16:55	396	168	360	84
31/05/15 17:00	384	276	528	180
31/05/15 17:05	540	264	552	84
31/05/15 17:10	588	240	624	168
31/05/15 17:15	552	312	480	108
31/05/15 17:20	492	288	516	144
31/05/15 17:25	552	204	540	192
31/05/15 17:30	492	348	564	168
31/05/15 17:35	564	240	576	168
31/05/15 17:40	528	240	540	132
31/05/15 17:45	600	264	600	120
31/05/15 17:50	840	348	732	168
31/05/15 17:55	624	192	528	84
31/05/15 18:00	852	132	600	48
31/05/15 18:05	972	276	504	132
31/05/15 18:10	936	300	708	120
31/05/15 18:15	1008	276	528	144
31/05/15 18:20	984	300	708	228
31/05/15 18:25	1152	252	504	180
31/05/15 18:30	852	216	828	204
31/05/15 18:35	1140	300	672	144
31/05/15 18:40	936	252	876	96
31/05/15 18:45	1056	300	792	204
31/05/15 18:50	768	192	684	168
31/05/15 18:55	864	264	852	180

Sistema de Controle de Tráfego Urbano OPTIMUS

5 MINUTOS	INTENSIDADE			
	PM 0402004	PM 0402007	PM04030031	PM04030032
31/05/15 19:00	720	312	636	156
31/05/15 19:05	744	240	600	120
31/05/15 19:10	684	336	576	156
31/05/15 19:15	600	360	744	192
31/05/15 19:20	588	300	636	132
31/05/15 19:25	540	288	696	132
31/05/15 19:30	648	372	672	168
31/05/15 19:35	432	264	660	156
31/05/15 19:40	420	264	696	84
31/05/15 19:45	528	192	540	120
31/05/15 19:50	588	204	468	132
31/05/15 19:55	636	228	480	84
31/05/15 20:00	444	204	336	96
31/05/15 20:05	600	216	516	96
31/05/15 20:10	456	276	384	84
31/05/15 20:15	444	180	528	72
31/05/15 20:20	456	192	480	108
31/05/15 20:25	396	240	540	108
31/05/15 20:30	480	204	552	180
31/05/15 20:35	468	120	456	72
31/05/15 20:40	444	300	540	156
31/05/15 20:45	612	288	468	168
31/05/15 20:50	600	252	444	192
31/05/15 20:55	456	240	456	48
31/05/15 21:00	504	168	480	156
31/05/15 21:05	420	300	552	180
31/05/15 21:10	396	180	504	120
31/05/15 21:15	468	216	396	84
31/05/15 21:20	516	132	456	72
31/05/15 21:25	564	228	396	132
31/05/15 21:30	432	240	396	156
31/05/15 21:35	384	180	444	60
31/05/15 21:40	336	264	540	84
31/05/15 21:45	324	300	348	204
31/05/15 21:50	384	228	492	96
31/05/15 21:55	336	156	372	36
31/05/15 22:00	360	156	324	84
31/05/15 22:05	276	192	324	96
31/05/15 22:10	372	204	396	144
31/05/15 22:15	252	192	372	96
31/05/15 22:20	240	96	264	60
31/05/15 22:25	384	168	384	84
31/05/15 22:30	312	168	204	72
31/05/15 22:35	288	24	276	24
31/05/15 22:40	360	84	216	60
31/05/15 22:45	336	156	264	24
31/05/15 22:50	264	48	204	36
31/05/15 22:55	216	12	216	60
31/05/15 23:00	252	84	288	96
31/05/15 23:05	336	36	228	48
31/05/15 23:10	156	144	204	36
31/05/15 23:15	216	96	192	48
31/05/15 23:20	168	144	228	60
31/05/15 23:25	180	96	144	60
31/05/15 23:30	228	72	144	24
31/05/15 23:35	168	60	216	24
31/05/15 23:40	96	84	168	36
31/05/15 23:45	192	36	168	36

Sistema de Controle de Tráfego Urbano OPTIMUS

5 MINUTOS	INTENSIDADE			
	PM 0402004	PM 0402007	PM04030031	PM04030032
31/05/15 23:50	132	72	108	12
31/05/15 23:55	180	144	204	36
01/06/15 00:00	96	0	132	12
01/06/15 00:05	108	0	144	12
01/06/15 00:10	96	108	132	24
01/06/15 00:15	84	36	60	24
01/06/15 00:20	180	72	204	0
01/06/15 00:25	144	36	72	12
01/06/15 00:30	156	24	48	24
01/06/15 00:35	108	24	36	12
01/06/15 00:40	84	48	96	0
01/06/15 00:45	120	0	36	0
01/06/15 00:50	72	24	48	12
01/06/15 00:55	72	12	84	12
01/06/15 01:00	72	36	84	0
01/06/15 01:05	120	36	84	0
01/06/15 01:10	96	96	96	24
01/06/15 01:15	120	48	60	24
01/06/15 01:20	48	24	60	0
01/06/15 01:25	60	24	24	12
01/06/15 01:30	60	24	24	24
01/06/15 01:35	24	0	36	0
01/06/15 01:40	24	12	12	0
01/06/15 01:45	36	36	48	0
01/06/15 01:50	36	12	48	12
01/06/15 01:55	12	12	60	0
01/06/15 02:00	48	12	24	0
01/06/15 02:05	72	0	60	0
01/06/15 02:10	60	36	48	0
01/06/15 02:15	24	12	36	0
01/06/15 02:20	84	12	48	0
01/06/15 02:25	60	0	12	0
01/06/15 02:30	48	12	12	0
01/06/15 02:35	24	12	24	0
01/06/15 02:40	24	0	12	0
01/06/15 02:45	60	0	12	0
01/06/15 02:50	36	12	84	12
01/06/15 02:55	36	24	36	12
01/06/15 03:00	36	0	48	0
01/06/15 03:05	36	0	12	0
01/06/15 03:10	24	24	36	12
01/06/15 03:15	24	24	36	12
01/06/15 03:20	36	12	36	12
01/06/15 03:25	36	12	12	0
01/06/15 03:30	36	24	24	0
01/06/15 03:35	24	12	48	0
01/06/15 03:40	36	0	48	0
01/06/15 03:45	24	0	36	0
01/06/15 03:50	12	12	12	0
01/06/15 03:55	24	12	48	0
01/06/15 04:00	12	0	24	0
01/06/15 04:05	60	0	12	0
01/06/15 04:10	48	0	0	0
01/06/15 04:15	24	0	0	0
01/06/15 04:20	12	12	84	0
01/06/15 04:25	48	12	48	0
01/06/15 04:30	72	12	36	0
01/06/15 04:35	48	0	24	0

Sistema de Controle de Tráfego Urbano OPTIMUS

5 MINUTOS	INTENSIDADE			
	PM 0402004	PM 0402007	PM 04030031	PM 04030032
01/06/15 04:40	24	0	12	0
01/06/15 04:45	48	12	24	12
01/06/15 04:50	36	12	24	0
01/06/15 04:55	24	12	48	0
01/06/15 05:00	48	36	36	24
01/06/15 05:05	72	60	36	24
01/06/15 05:10	60	12	60	12
01/06/15 05:15	60	24	48	0
01/06/15 05:20	144	48	60	0
01/06/15 05:25	84	36	60	36
01/06/15 05:30	84	72	120	24
01/06/15 05:35	84	36	60	12
01/06/15 05:40	156	36	60	24
01/06/15 05:45	180	84	96	36
01/06/15 05:50	108	120	168	48
01/06/15 05:55	168	48	228	24
01/06/15 06:00	168	108	276	24
01/06/15 06:05	228	96	336	48
01/06/15 06:10	144	60	384	12
01/06/15 06:15	204	108	276	48
01/06/15 06:20	240	120	408	36
01/06/15 06:25	324	156	348	72
01/06/15 06:30	408	132	516	96
01/06/15 06:35	348	192	588	96
01/06/15 06:40	684	168	756	120
01/06/15 06:45	936	540	720	132
01/06/15 06:50	1128	372	804	180
01/06/15 06:55	756	708	828	252
01/06/15 07:00	972	756	984	300
01/06/15 07:05	864	684	1056	264
01/06/15 07:10	1152	600	816	192
01/06/15 07:15	888	696	1056	192
01/06/15 07:20	1212	660	888	216
01/06/15 07:25	1044	408	780	144
01/06/15 07:30	1068	492	756	192
01/06/15 07:35	1008	648	900	204
01/06/15 07:40	972	348	540	204
01/06/15 07:45	936	420	744	156
01/06/15 07:50	1044	624	804	240
01/06/15 07:55	936	384	804	180
01/06/15 08:00	864	396	768	276
01/06/15 08:05	792	432	876	216
01/06/15 08:10	936	384	648	120
01/06/15 08:15	1044	264	720	156
01/06/15 08:20	708	444	852	168
01/06/15 08:25	804	468	660	204
01/06/15 08:30	852	408	756	156
01/06/15 08:35	828	288	672	84
01/06/15 08:40	804	420	744	96
01/06/15 08:45	648	372	708	108
01/06/15 08:50	732	600	684	276
01/06/15 08:55	744	360	756	168
01/06/15 09:00	648	552	876	240
01/06/15 09:05	648	336	684	192
01/06/15 09:10	780	564	780	180
01/06/15 09:15	576	468	660	216
01/06/15 09:20	780	456	816	168
01/06/15 09:25	840	408	732	144

Sistema de Controle de Tráfego Urbano OPTIMUS

5 MINUTOS	INTENSIDADE			
	PM 0402004	PM 0402007	PM04030031	PM04030032
01/06/15 09:30	732	444	852	132
01/06/15 09:35	888	408	648	228
01/06/15 09:40	720	528	888	204
01/06/15 09:45	744	420	804	120
01/06/15 09:50	780	360	720	228
01/06/15 09:55	816	396	708	144
01/06/15 10:00	612	528	960	192
01/06/15 10:05	672	396	852	288
01/06/15 10:10	648	588	792	240
01/06/15 10:15	756	300	720	204
01/06/15 10:20	660	384	840	132
01/06/15 10:25	588	432	744	168
01/06/15 10:30	804	564	672	216
01/06/15 10:35	780	516	720	288
01/06/15 10:40	876	504	888	168
01/06/15 10:45	708	384	816	144
01/06/15 10:50	756	528	780	204
01/06/15 10:55	744	468	816	252
01/06/15 11:00	744	432	792	168
01/06/15 11:05	744	504	756	360
01/06/15 11:10	744	480	684	192
01/06/15 11:15	720	564	816	252
01/06/15 11:20	576	624	804	216
01/06/15 11:25	852	576	900	216
01/06/15 11:30	660	420	864	336
01/06/15 11:35	564	540	672	300
01/06/15 11:40	780	612	816	192
01/06/15 11:45	684	288	648	144
01/06/15 11:50	660	600	828	264
01/06/15 11:55	708	636	900	168
01/06/15 12:00	648	648	1020	336
01/06/15 12:05	624	492	876	312
01/06/15 12:10	660	780	756	264
01/06/15 12:15	876	564	900	312
01/06/15 12:20	696	432	828	168
01/06/15 12:25	624	552	756	276
01/06/15 12:30	720	420	768	216
01/06/15 12:35	612	720	1008	228
01/06/15 12:40	720	408	924	252
01/06/15 12:45	720	696	948	264
01/06/15 12:50	552	636	888	252
01/06/15 12:55	672	708	1116	348
01/06/15 13:00	744	552	924	216
01/06/15 13:05	720	672	984	252
01/06/15 13:10	804	516	1020	300
01/06/15 13:15	708	552	1080	252
01/06/15 13:20	816	432	1008	192
01/06/15 13:25	960	444	1008	120
01/06/15 13:30	696	576	720	168
01/06/15 13:35	648	540	804	144
01/06/15 13:40	720	516	864	252
01/06/15 13:45	1056	504	804	288
01/06/15 13:50	756	516	756	252
01/06/15 13:55	900	552	876	240
01/06/15 14:00	816	588	816	192
01/06/15 14:05	732	408	756	252
01/06/15 14:10	732	516	828	240
01/06/15 14:15	720	552	972	216

Sistema de Controle de Tráfego Urbano OPTIMUS

5 MINUTOS	INTENSIDADE			
	PM 0402004	PM 0402007	PM04030031	PM04030032
01/06/15 14:20	876	492	948	204
01/06/15 14:25	756	576	900	180
01/06/15 14:30	936	360	864	180
01/06/15 14:35	900	420	852	168
01/06/15 14:40	732	504	1020	216
01/06/15 14:45	888	408	744	192
01/06/15 14:50	648	492	912	276
01/06/15 14:55	840	468	972	144
01/06/15 15:00	780	480	804	216
01/06/15 15:05	864	396	864	156
01/06/15 15:10	840	588	852	288
01/06/15 15:15	1068	372	900	252
01/06/15 15:20	900	408	828	216
01/06/15 15:25	744	480	852	228
01/06/15 15:30	696	744	876	228
01/06/15 15:35	840	552	912	192
01/06/15 15:40	804	480	996	192
01/06/15 15:45	900	444	816	252
01/06/15 15:50	708	396	852	168
01/06/15 15:55	684	576	840	216
01/06/15 16:00	744	408	804	180
01/06/15 16:05	732	504	780	132
01/06/15 16:10	804	540	924	288
01/06/15 16:15	876	456	780	144
01/06/15 16:20	648	540	900	180
