# Supplementary material for: Traffic light optimization using non-dominated sorting genetic algorithm (NSGA2)
Source: Sci Rep. 2023 Sep 20;13:15550. doi: 10.1038/s41598-023-38884-2 (PMC10511403; doi:10.1038/s41598-023-38884-2)
Supplement: Supplementary file 1 — Supplementary Information. [file 41598_2023_38884_MOESM1_ESM.zip › dadosBHTrans/Dados-BHTRANS02/I_1_dat_4_cs_5_min_st3.pdf]

Sistema de Controle de Tráfego Urbano OPTIMUS

INTENSIDADE DE 4 PONTOS DE MEDIDA

DADOS DE 5 MINUTOS

PONTO DE MEDIDA 1:PM 04030 04 (Floresta)
PONTO DE MEDIDA 2:PM 04030 06 ()
PONTO DE MEDIDA 3:PM 04040 02 (Sapucaí)
PONTO DE MEDIDA 4:PM 04040 04 (Contorno)
DESDE:01/05/2015 16:27
ATÉ:01/06/201516:27

INTENSIDADE / 5 MINUTOS

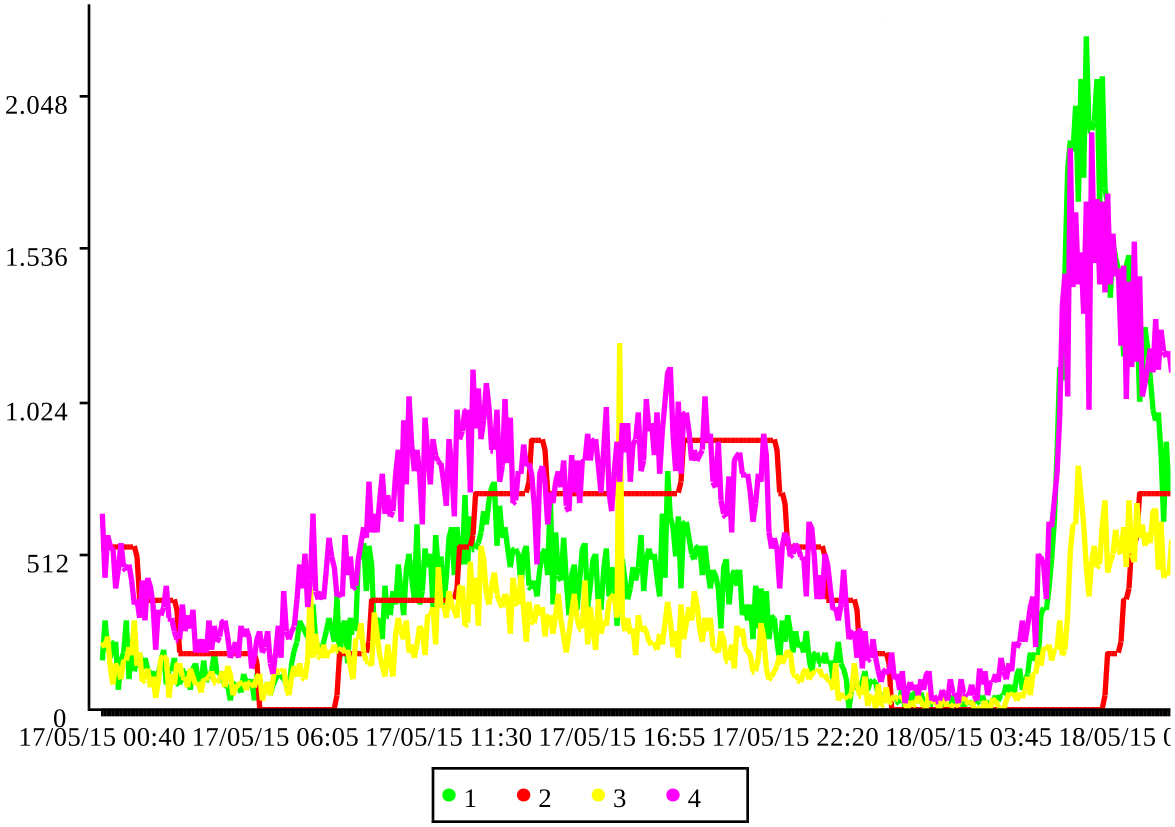

5 MINUTOS	INTENSIDADE			
	PM 0403004	PM 0403006	PM 0404002	PM 0404004
17/05/15 00:40	156	540	217	648
17/05/15 00:45	288	540	204	432
17/05/15 00:50	204	540	236	576
17/05/15 00:55	168	540	127	540
17/05/15 01:00	216	540	73	528
17/05/15 01:05	216	540	145	396
17/05/15 01:10	60	540	129	456
17/05/15 01:15	156	540	94	552
17/05/15 01:20	180	540	154	468
17/05/15 01:25	288	540	159	456
17/05/15 01:30	96	540	201	480
17/05/15 01:35	228	540	170	420
17/05/15 01:40	120	540	294	348
17/05/15 01:45	156	504	141	360
17/05/15 01:50	180	360	186	300

Sistema de Controle de Tráfego Urbano OPTIMUS

5 MINUTOS	INTENSIDADE			
	PM 0403004	PM 0403006	PM 0404002	PM 0404004
17/05/15 01:55	132	360	82	420
17/05/15 02:00	168	360	98	288
17/05/15 02:05	132	360	126	432
17/05/15 02:10	120	360	70	408
17/05/15 02:15	84	360	106	348
17/05/15 02:20	120	360	31	192
17/05/15 02:25	108	360	87	324
17/05/15 02:30	156	360	163	312
17/05/15 02:35	192	360	175	336
17/05/15 02:40	72	360	166	408
17/05/15 02:45	120	360	31	300
17/05/15 02:50	120	360	115	300
17/05/15 02:55	168	360	79	252
17/05/15 03:00	84	324	151	228
17/05/15 03:05	72	180	144	228
17/05/15 03:10	132	180	103	348
17/05/15 03:15	84	180	99	252
17/05/15 03:20	120	180	67	312
17/05/15 03:25	60	180	127	264
17/05/15 03:30	132	180	105	324
17/05/15 03:35	144	180	79	180
17/05/15 03:40	84	180	87	228
17/05/15 03:45	72	180	46	180
17/05/15 03:50	156	180	96	228
17/05/15 03:55	84	180	98	180
17/05/15 04:00	108	180	103	288
17/05/15 04:05	120	180	123	192
17/05/15 04:10	180	180	86	276
17/05/15 04:15	108	180	87	216
17/05/15 04:20	120	180	94	240
17/05/15 04:25	96	180	122	288
17/05/15 04:30	72	180	63	288
17/05/15 04:35	72	180	135	240
17/05/15 04:40	24	180	108	168
17/05/15 04:45	84	180	36	192
17/05/15 04:50	72	180	63	216
17/05/15 04:55	72	180	75	168
17/05/15 05:00	48	180	86	276
17/05/15 05:05	108	180	84	240
17/05/15 05:10	84	180	55	264
17/05/15 05:15	84	180	84	240
17/05/15 05:20	96	180	84	132
17/05/15 05:25	24	180	63	168
17/05/15 05:30	108	144	106	228
17/05/15 05:35	72	0	110	252
17/05/15 05:40	60	0	24	180
17/05/15 05:45	60	0	55	204
17/05/15 05:50	84	0	82	252
17/05/15 05:55	36	0	36	144
17/05/15 06:00	72	0	94	108
17/05/15 06:05	96	0	127	180
17/05/15 06:10	108	0	118	276
17/05/15 06:15	108	0	118	168
17/05/15 06:20	96	0	127	384
17/05/15 06:25	96	0	67	252
17/05/15 06:30	48	0	43	228
17/05/15 06:35	144	0	106	252
17/05/15 06:40	192	0	142	276

Sistema de Controle de Tráfego Urbano OPTIMUS

5 MINUTOS	INTENSIDADE			
	PM 04030 04	PM 04030 06	PM 04040 02	PM 04040 04
17/05/15 06:45	216	0	103	360
17/05/15 06:50	288	0	146	480
17/05/15 06:55	276	0	99	396
17/05/15 07:00	252	0	94	516
17/05/15 07:05	240	0	153	336
17/05/15 07:10	252	0	398	420
17/05/15 07:15	348	0	162	648
17/05/15 07:20	228	0	249	384
17/05/15 07:25	204	0	178	360
17/05/15 07:30	180	0	166	384
17/05/15 07:35	216	0	210	360
17/05/15 07:40	252	0	169	456
17/05/15 07:45	300	0	190	564
17/05/15 07:50	264	0	186	504
17/05/15 07:55	216	0	213	468
17/05/15 08:00	372	36	189	372
17/05/15 08:05	156	180	189	384
17/05/15 08:10	276	180	205	372
17/05/15 08:15	288	180	182	576
17/05/15 08:20	144	180	189	444
17/05/15 08:25	300	180	134	504
17/05/15 08:30	252	180	94	396
17/05/15 08:35	252	180	229	408
17/05/15 08:40	492	180	230	492
17/05/15 08:45	492	180	285	552
17/05/15 08:50	552	180	182	600
17/05/15 08:55	384	180	154	564
17/05/15 09:00	540	216	153	756
17/05/15 09:05	480	360	136	588
17/05/15 09:10	240	360	314	648
17/05/15 09:15	300	360	242	588
17/05/15 09:20	288	360	178	696
17/05/15 09:25	228	360	141	780
17/05/15 09:30	408	360	105	648
17/05/15 09:35	312	360	212	708
17/05/15 09:40	312	360	148	636
17/05/15 09:45	384	360	102	660
17/05/15 09:50	348	360	249	780
17/05/15 09:55	480	360	302	864
17/05/15 10:00	384	360	253	624
17/05/15 10:05	312	360	224	960
17/05/15 10:10	456	360	292	744
17/05/15 10:15	516	360	180	1044
17/05/15 10:20	432	360	213	876
17/05/15 10:25	360	360	166	792
17/05/15 10:30	612	360	213	864
17/05/15 10:35	348	360	268	780
17/05/15 10:40	384	360	261	612
17/05/15 10:45	528	360	178	972
17/05/15 10:50	468	360	309	852
17/05/15 10:55	516	360	309	744
17/05/15 11:00	336	360	336	900
17/05/15 11:05	576	360	210	840
17/05/15 11:10	456	360	472	828
17/05/15 11:15	504	360	344	816
17/05/15 11:20	348	360	347	756
17/05/15 11:25	432	360	303	672
17/05/15 11:30	564	360	384	900

Sistema de Controle de Tráfego Urbano OPTIMUS

5 MINUTOS	INTENSIDADE			
	PM 0403004	PM 0403006	PM 0404002	PM 0404004
17/05/15 11:35	600	360	346	804
17/05/15 11:40	444	360	328	636
17/05/15 11:45	600	396	297	996
17/05/15 11:50	516	540	405	900
17/05/15 11:55	480	540	405	924
17/05/15 12:00	708	540	271	996
17/05/15 12:05	480	540	278	996
17/05/15 12:10	636	540	490	720
17/05/15 12:15	528	576	342	1128
17/05/15 12:20	528	720	436	936
17/05/15 12:25	552	720	270	1068
17/05/15 12:30	588	720	538	900
17/05/15 12:35	660	720	496	960
17/05/15 12:40	612	720	379	1080
17/05/15 12:45	696	720	348	1008
17/05/15 12:50	732	720	410	864
17/05/15 12:55	756	720	453	864
17/05/15 13:00	540	720	375	996
17/05/15 13:05	672	720	351	756
17/05/15 13:10	564	720	333	804
17/05/15 13:15	600	720	379	1032
17/05/15 13:20	528	720	372	816
17/05/15 13:25	468	720	248	972
17/05/15 13:30	528	720	435	684
17/05/15 13:35	528	720	356	696
17/05/15 13:40	516	720	342	792
17/05/15 13:45	408	720	439	780
17/05/15 13:50	516	720	326	876
17/05/15 13:55	540	720	220	804
17/05/15 14:00	408	756	351	816
17/05/15 14:05	396	900	288	780
17/05/15 14:10	420	900	277	672
17/05/15 14:15	372	900	343	480
17/05/15 14:20	456	900	306	780
17/05/15 14:25	516	900	332	804
17/05/15 14:30	528	864	240	756
17/05/15 14:35	444	720	310	612
17/05/15 14:40	708	720	304	720
17/05/15 14:45	348	720	248	684
17/05/15 14:50	588	720	309	756
17/05/15 14:55	444	720	380	792
17/05/15 15:00	420	720	292	732
17/05/15 15:05	564	720	240	828
17/05/15 15:10	420	720	184	672
17/05/15 15:15	312	720	246	660
17/05/15 15:20	444	720	321	840
17/05/15 15:25	480	720	376	720
17/05/15 15:30	324	720	297	828
17/05/15 15:35	420	720	265	684
17/05/15 15:40	528	720	267	828
17/05/15 15:45	552	720	428	828
17/05/15 15:50	336	720	210	912
17/05/15 15:55	408	720	336	828
17/05/15 16:00	492	720	255	900
17/05/15 16:05	516	720	193	900
17/05/15 16:10	348	720	363	804
17/05/15 16:15	324	720	241	720
17/05/15 16:20	456	720	294	864

Sistema de Controle de Tráfego Urbano OPTIMUS

5 MINUTOS	INTENSIDADE			
	PM 0403004	PM 0403006	PM 0404002	PM 0404004
17/05/15 16:25	528	720	272	1008
17/05/15 16:30	372	720	356	720
17/05/15 16:35	468	720	366	660
17/05/15 16:40	432	720	378	792
17/05/15 16:45	276	720	298	888
17/05/15 16:50	420	720	1218	756
17/05/15 16:55	492	720	306	948
17/05/15 17:00	420	720	254	852
17/05/15 17:05	360	720	303	948
17/05/15 17:10	396	720	249	756
17/05/15 17:15	468	720	262	876
17/05/15 17:20	420	720	170	900
17/05/15 17:25	468	720	308	984
17/05/15 17:30	576	720	258	792
17/05/15 17:35	384	720	272	828
17/05/15 17:40	492	720	258	1032
17/05/15 17:45	528	720	222	924
17/05/15 17:50	492	720	223	948
17/05/15 17:55	516	720	216	840
17/05/15 18:00	432	720	193	984
17/05/15 18:05	372	720	247	780
17/05/15 18:10	648	720	208	924
17/05/15 18:15	432	720	280	1020
17/05/15 18:20	792	720	356	1116
17/05/15 18:25	636	720	289	1140
17/05/15 18:30	600	720	204	912
17/05/15 18:35	504	720	256	792
17/05/15 18:40	636	720	164	1020
17/05/15 18:45	396	756	344	780
17/05/15 18:50	624	900	229	984
17/05/15 18:55	624	900	280	984
17/05/15 19:00	564	900	339	924
17/05/15 19:05	516	900	288	828
17/05/15 19:10	492	900	391	864
17/05/15 19:15	540	900	344	828
17/05/15 19:20	540	900	261	840
17/05/15 19:25	444	900	216	864
17/05/15 19:30	540	900	303	1044
17/05/15 19:35	456	900	250	888
17/05/15 19:40	396	900	297	912
17/05/15 19:45	456	900	201	756
17/05/15 19:50	444	900	199	732
17/05/15 19:55	396	900	181	888
17/05/15 20:00	264	900	252	696
17/05/15 20:05	468	900	228	648
17/05/15 20:10	492	900	160	636
17/05/15 20:15	348	900	153	768
17/05/15 20:20	432	900	211	588
17/05/15 20:25	456	900	126	828
17/05/15 20:30	408	900	271	840
17/05/15 20:35	456	900	238	852
17/05/15 20:40	312	900	267	780
17/05/15 20:45	348	900	204	780
17/05/15 20:50	288	900	195	780
17/05/15 20:55	348	900	172	720
17/05/15 21:00	228	900	124	624
17/05/15 21:05	408	900	121	684
17/05/15 21:10	264	900	165	780

Sistema de Controle de Tráfego Urbano OPTIMUS

5 MINUTOS	INTENSIDADE			
	PM 0403004	PM 0403006	PM 0404002	PM 0404004
17/05/15 21:15	396	900	280	756
17/05/15 21:20	192	900	238	912
17/05/15 21:25	384	900	133	840
17/05/15 21:30	228	900	94	588
17/05/15 21:35	312	900	102	528
17/05/15 21:40	300	900	122	564
17/05/15 21:45	216	864	134	528
17/05/15 21:50	156	720	174	396
17/05/15 21:55	288	720	146	540
17/05/15 22:00	216	684	164	588
17/05/15 22:05	312	540	193	564
17/05/15 22:10	252	540	184	504
17/05/15 22:15	180	540	116	516
17/05/15 22:20	252	540	110	552
17/05/15 22:25	204	540	98	528
17/05/15 22:30	216	540	85	504
17/05/15 22:35	180	540	118	504
17/05/15 22:40	252	540	129	372
17/05/15 22:45	168	540	94	624
17/05/15 22:50	108	540	115	600
17/05/15 22:55	180	540	122	504
17/05/15 23:00	180	540	117	360
17/05/15 23:05	144	540	127	444
17/05/15 23:10	144	540	103	360
17/05/15 23:15	180	504	114	516
17/05/15 23:20	144	360	90	480
17/05/15 23:25	156	360	97	396
17/05/15 23:30	96	360	57	324
17/05/15 23:35	156	360	148	336
17/05/15 23:40	216	360	19	288
17/05/15 23:45	144	360	50	348
17/05/15 23:50	132	360	34	456
17/05/15 23:55	96	360	39	336
18/05/15 00:00	0	360	31	228
18/05/15 00:05	60	360	51	252
18/05/15 00:10	120	360	150	252
18/05/15 00:15	60	324	78	168
18/05/15 00:20	72	180	36	264
18/05/15 00:25	84	180	39	132
18/05/15 00:30	120	180	103	252
18/05/15 00:35	60	180	7	156
18/05/15 00:40	84	180	39	168
18/05/15 00:45	96	180	43	228
18/05/15 00:50	84	180	0	168
18/05/15 00:55	36	180	75	120
18/05/15 01:00	48	180	3	84
18/05/15 01:05	24	180	15	120
18/05/15 01:10	36	180	63	132
18/05/15 01:15	48	144	15	108
18/05/15 01:20	24	0	24	180
18/05/15 01:25	36	0	39	96
18/05/15 01:30	24	0	31	108
18/05/15 01:35	12	0	31	60
18/05/15 01:40	72	0	39	120
18/05/15 01:45	0	0	12	12
18/05/15 01:50	12	0	0	60
18/05/15 01:55	48	0	24	84
18/05/15 02:00	24	0	3	60

Sistema de Controle de Tráfego Urbano OPTIMUS

5 MINUTOS	INTENSIDADE			
	PM 0403004	PM 0403006	PM 0404002	PM 0404004
18/05/15 02:05	60	0	12	72
18/05/15 02:10	12	0	36	36
18/05/15 02:15	48	0	24	96
18/05/15 02:20	12	0	3	60
18/05/15 02:25	0	0	0	108
18/05/15 02:30	36	0	48	120
18/05/15 02:35	12	0	24	24
18/05/15 02:40	12	0	24	36
18/05/15 02:45	24	0	12	24
18/05/15 02:50	12	0	0	24
18/05/15 02:55	12	0	36	60
18/05/15 03:00	12	0	12	48
18/05/15 03:05	0	0	12	24
18/05/15 03:10	0	0	15	96
18/05/15 03:15	36	0	3	48
18/05/15 03:20	24	0	3	48
18/05/15 03:25	12	0	12	12
18/05/15 03:30	36	0	19	96
18/05/15 03:35	0	0	36	48
18/05/15 03:40	24	0	24	48
18/05/15 03:45	24	0	12	36
18/05/15 03:50	12	0	24	72
18/05/15 03:55	12	0	0	36
18/05/15 04:00	12	0	7	24
18/05/15 04:05	24	0	0	132
18/05/15 04:10	36	0	0	72
18/05/15 04:15	12	0	24	120
18/05/15 04:20	12	0	0	36
18/05/15 04:25	24	0	0	84
18/05/15 04:30	12	0	27	84
18/05/15 04:35	36	0	24	108
18/05/15 04:40	24	0	7	84
18/05/15 04:45	36	0	0	168
18/05/15 04:50	36	0	0	120
18/05/15 04:55	72	0	24	96
18/05/15 05:00	36	0	50	144
18/05/15 05:05	72	0	36	120
18/05/15 05:10	48	0	48	216
18/05/15 05:15	108	0	19	204
18/05/15 05:20	132	0	51	216
18/05/15 05:25	60	0	34	288
18/05/15 05:30	84	0	55	216
18/05/15 05:35	108	0	103	276
18/05/15 05:40	180	0	43	336
18/05/15 05:45	156	0	115	372
18/05/15 05:50	180	0	115	204
18/05/15 05:55	168	0	170	516
18/05/15 06:00	312	0	142	504
18/05/15 06:05	336	0	198	480
18/05/15 06:10	324	0	189	360
18/05/15 06:15	396	0	211	624
18/05/15 06:20	492	0	154	600
18/05/15 06:25	624	0	196	696
18/05/15 06:30	840	0	196	780
18/05/15 06:35	1140	0	286	972
18/05/15 06:40	1092	0	174	1344
18/05/15 06:45	1464	0	190	1452
18/05/15 06:50	1800	0	304	1044

Sistema de Controle de Tráfego Urbano OPTIMUS

5 MINUTOS	INTENSIDADE			
	PM 040300 4	PM 040300 6	PM 040400 2	PM 040400 4
18/05/15 06:55	1896	0	518	1872
18/05/15 07:00	1860	0	614	1404
18/05/15 07:05	2016	0	615	1656
18/05/15 07:10	1692	0	812	1416
18/05/15 07:15	2100	0	708	1524
18/05/15 07:20	1776	0	608	1320
18/05/15 07:25	2244	0	465	1692
18/05/15 07:30	1932	0	369	996
18/05/15 07:35	1932	0	488	1920
18/05/15 07:40	1956	0	542	1488
18/05/15 07:45	2100	0	465	1704
18/05/15 07:50	1680	0	475	1416
18/05/15 07:55	2112	0	613	1692
18/05/15 08:00	1740	36	692	1392
18/05/15 08:05	1680	180	451	1716
18/05/15 08:10	1368	180	483	1416
18/05/15 08:15	1560	180	511	1584
18/05/15 08:20	1500	180	596	1440
18/05/15 08:25	1464	180	535	1464
18/05/15 08:30	1380	216	511	1212
18/05/15 08:35	1176	360	603	1476
18/05/15 08:40	1476	360	471	1032
18/05/15 08:45	1512	396	692	1428
18/05/15 08:50	1368	540	514	1140
18/05/15 08:55	1464	540	489	1560
18/05/15 09:00	1296	576	682	1152
18/05/15 09:05	1020	720	564	1440
18/05/15 09:10	1128	720	614	1044
18/05/15 09:15	1272	720	537	1080
18/05/15 09:20	1200	720	582	1140
18/05/15 09:25	1104	720	552	1200
18/05/15 09:30	996	720	652	1116
18/05/15 09:35	960	720	664	1296
18/05/15 09:40	984	720	457	1128
18/05/15 09:45	852	720	616	1260
18/05/15 09:50	624	720	431	1188
18/05/15 09:55	888	720	459	1176
18/05/15 10:00	720	720	444	1188
18/05/15 10:05	744	720	555	1116
18/05/15 10:10	732	720	565	1248
18/05/15 10:15	684	720	553	1248
18/05/15 10:20	804	720	592	1068
18/05/15 10:25	792	720	692	1356
18/05/15 10:30	732	720	738	1008
18/05/15 10:35	720	720	477	1296
18/05/15 10:40	888	720	616	1068
18/05/15 10:45	864	720	682	1152
18/05/15 10:50	912	720	660	1200
18/05/15 10:55	936	720	508	1116
18/05/15 11:00	648	720	569	900
18/05/15 11:05	600	720	721	1176
18/05/15 11:10	696	720	606	1128
18/05/15 11:15	708	720	727	1164
18/05/15 11:20	468	720	772	1092
18/05/15 11:25	708	720	792	1368
18/05/15 11:30	612	720	672	936
18/05/15 11:35	768	720	764	912
18/05/15 11:40	588	720	674	1248

Sistema de Controle de Tráfego Urbano OPTIMUS

5 MINUTOS	INTENSIDADE			
	PM 040300 4	PM 040300 6	PM 040400 2	PM 040400 4
18/05/15 11:45	1020	720	786	1116
18/05/15 11:50	672	720	824	1080
18/05/15 11:55	684	720	859	1044
18/05/15 12:00	588	720	781	1080
18/05/15 12:05	624	720	852	912
18/05/15 12:10	660	720	800	900
18/05/15 12:15	780	756	720	1080
18/05/15 12:20	756	900	687	1092
18/05/15 12:25	804	900	716	1092
18/05/15 12:30	636	900	732	864
18/05/15 12:35	888	900	619	864
18/05/15 12:40	684	900	446	1308
18/05/15 12:45	876	900	700	948
18/05/15 12:50	984	900	742	1188
18/05/15 12:55	1200	900	768	1404
18/05/15 13:00	1008	900	666	876
18/05/15 13:05	1008	900	727	1452
18/05/15 13:10	888	900	705	984
18/05/15 13:15	1044	900	794	1560
18/05/15 13:20	948	900	556	1128
18/05/15 13:25	960	900	616	1128
18/05/15 13:30	1092	900	576	1380
18/05/15 13:35	1128	900	654	1476
18/05/15 13:40	888	900	649	1260
18/05/15 13:45	1200	900	540	1272
18/05/15 13:50	840	900	482	1200
18/05/15 13:55	1176	900	544	1284
18/05/15 14:00	972	900	615	1596
18/05/15 14:05	888	900	676	1272
18/05/15 14:10	1032	900	514	1308
18/05/15 14:15	1092	900	672	1464
18/05/15 14:20	900	900	741	1212
18/05/15 14:25	888	900	631	1200
18/05/15 14:30	984	900	589	1428
18/05/15 14:35	1008	900	660	1476
18/05/15 14:40	960	900	771	1140
18/05/15 14:45	1092	900	664	1200
18/05/15 14:50	768	900	655	1356
18/05/15 14:55	708	900	661	1152
18/05/15 15:00	756	900	709	1368
18/05/15 15:05	708	900	589	1488
18/05/15 15:10	804	900	769	1224
18/05/15 15:15	768	900	548	1176
18/05/15 15:20	852	900	735	1248
18/05/15 15:25	696	900	534	1200
18/05/15 15:30	732	900	854	1380
18/05/15 15:35	780	900	697	1104
18/05/15 15:40	816	900	1015	1344
18/05/15 15:45	624	936	498	1188
18/05/15 15:50	888	1080	901	1224
18/05/15 15:55	888	1080	698	1308
18/05/15 16:00	708	1080	1033	1092
18/05/15 16:05	804	1080	543	1152
18/05/15 16:10	588	1080	932	1092
18/05/15 16:15	792	1080	514	1188
18/05/15 16:20	804	1080	1102	1236
18/05/15 16:25	576	1080	860	1188
18/05/15 16:30	612	1080	987	1068

Sistema de Controle de Tráfego Urbano OPTIMUS

5 MINUTOS	INTENSIDADE			
	PM 040300 4	PM 040300 6	PM 040400 2	PM 040400 4
18/05/15 16:35	768	1080	736	1008
18/05/15 16:40	684	1080	826	1188
18/05/15 16:45	780	1080	772	1140
18/05/15 16:50	708	1080	964	1092
18/05/15 16:55	732	1080	705	1152
18/05/15 17:00	672	1080	489	1188
18/05/15 17:05	828	1080	594	780
18/05/15 17:10	792	1080	350	252
18/05/15 17:15	624	1080	396	768
18/05/15 17:20	852	1080	304	1032
18/05/15 17:25	1020	1080	408	948
18/05/15 17:30	828	1080	254	768
18/05/15 17:35	840	1080	416	912
18/05/15 17:40	732	1080	132	960
18/05/15 17:45	768	1080	288	648
18/05/15 17:50	816	1080	314	924
18/05/15 17:55	648	1080	290	600
18/05/15 18:00	588	1080	206	660
18/05/15 18:05	744	1080	390	444
18/05/15 18:10	684	1080	100	900
18/05/15 18:15	816	1080	63	912
18/05/15 18:20	804	1080	254	768
18/05/15 18:25	756	1080	237	672
18/05/15 18:30	840	1044	440	624
18/05/15 18:35	660	900	262	768
18/05/15 18:40	564	900	352	744
18/05/15 18:45	708	900	368	1080
18/05/15 18:50	600	900	341	984
18/05/15 18:55	876	900	180	780
18/05/15 19:00	720	900	413	900
18/05/15 19:05	600	900	388	648
18/05/15 19:10	816	900	552	840
18/05/15 19:15	468	900	457	660
18/05/15 19:20	624	900	462	1152
18/05/15 19:25	564	900	344	960
18/05/15 19:30	600	900	636	1020
18/05/15 19:35	468	900	538	852
18/05/15 19:40	432	900	508	612
18/05/15 19:45	432	900	568	912
18/05/15 19:50	360	900	490	816
18/05/15 19:55	432	900	438	828
18/05/15 20:00	324	900	631	732
18/05/15 20:05	360	900	498	708
18/05/15 20:10	408	900	663	864
18/05/15 20:15	456	900	446	924
18/05/15 20:20	468	900	596	840
18/05/15 20:25	516	900	398	768
18/05/15 20:30	336	900	463	840
18/05/15 20:35	480	900	345	684
18/05/15 20:40	348	900	603	708
18/05/15 20:45	336	900	427	816
18/05/15 20:50	312	900	338	828
18/05/15 20:55	492	900	390	792
18/05/15 21:00	276	900	310	1008
18/05/15 21:05	300	900	254	696
18/05/15 21:10	216	900	516	864
18/05/15 21:15	252	900	472	684
18/05/15 21:20	324	900	541	636

Sistema de Controle de Tráfego Urbano OPTIMUS

5 MINUTOS	INTENSIDADE			
	PM 0403004	PM 0403006	PM 0404002	PM 0404004
18/05/15 21:25	204	900	327	720
18/05/15 21:30	252	900	307	492
18/05/15 21:35	228	900	244	696
18/05/15 21:40	240	900	284	840
18/05/15 21:45	276	864	328	792
18/05/15 21:50	240	720	411	480
18/05/15 21:55	240	720	319	636
18/05/15 22:00	240	720	475	684
18/05/15 22:05	408	720	320	540
18/05/15 22:10	288	720	558	720
18/05/15 22:15	396	720	348	684
18/05/15 22:20	300	720	457	684
18/05/15 22:25	312	720	559	708
18/05/15 22:30	252	720	780	744
18/05/15 22:35	300	720	576	600
18/05/15 22:40	168	720	637	504
18/05/15 22:45	204	720	488	624
18/05/15 22:50	228	720	246	528
18/05/15 22:55	168	720	192	288
18/05/15 23:00	216	720	174	456
18/05/15 23:05	180	720	219	432
18/05/15 23:10	84	720	127	276
18/05/15 23:15	84	684	69	372
18/05/15 23:20	192	540	248	312
18/05/15 23:25	156	540	91	324
18/05/15 23:30	48	504	130	312
18/05/15 23:35	120	360	105	240
18/05/15 23:40	132	360	82	312
18/05/15 23:45	0	360	94	192
18/05/15 23:50	120	360	24	324
18/05/15 23:55	60	360	3	240
19/05/15 00:00	72	324	67	180
19/05/15 00:05	96	180	79	120
19/05/15 00:10	24	180	115	144
19/05/15 00:15	72	180	74	252
19/05/15 00:20	72	180	46	192
19/05/15 00:25	48	180	50	240
19/05/15 00:30	60	180	43	132
19/05/15 00:35	96	180	22	120
19/05/15 00:40	108	180	103	84
19/05/15 00:45	72	180	36	192
19/05/15 00:50	24	180	50	120
19/05/15 00:55	84	180	39	120
19/05/15 01:00	72	144	3	132
19/05/15 01:05	48	0	34	60
19/05/15 01:10	72	0	39	108
19/05/15 01:15	12	0	48	48
19/05/15 01:20	36	0	12	84
19/05/15 01:25	48	0	39	108
19/05/15 01:30	12	0	27	144
19/05/15 01:35	36	0	36	72
19/05/15 01:40	36	0	34	132
19/05/15 01:45	0	0	48	48
19/05/15 01:50	24	0	3	132
19/05/15 01:55	12	0	39	24
19/05/15 02:00	36	0	27	48
19/05/15 02:05	36	0	3	36
19/05/15 02:10	12	0	19	72

Sistema de Controle de Tráfego Urbano OPTIMUS

5 MINUTOS	INTENSIDADE			
	PM 040300 4	PM 040300 6	PM 040400 2	PM 040400 4
19/05/15 02:15	36	0	12	144
19/05/15 02:20	36	0	12	24
19/05/15 02:25	12	0	7	72
19/05/15 02:30	36	0	48	48
19/05/15 02:35	24	0	12	12
19/05/15 02:40	48	0	24	48
19/05/15 02:45	12	0	0	24
19/05/15 02:50	0	0	12	60
19/05/15 02:55	12	0	12	36
19/05/15 03:00	0	0	15	24
19/05/15 03:05	0	0	48	96
19/05/15 03:10	36	0	15	72
19/05/15 03:15	12	0	26	72
19/05/15 03:20	36	0	0	96
19/05/15 03:25	48	0	15	24
19/05/15 03:30	12	0	12	48
19/05/15 03:35	0	0	12	36
19/05/15 03:40	0	0	48	12
19/05/15 03:45	24	0	12	84
19/05/15 03:50	0	0	12	24
19/05/15 03:55	12	0	39	36
19/05/15 04:00	24	0	24	72
19/05/15 04:05	0	0	19	36
19/05/15 04:10	0	0	75	36
19/05/15 04:15	48	0	48	144
19/05/15 04:20	24	0	24	96
19/05/15 04:25	0	0	0	60
19/05/15 04:30	0	0	15	84
19/05/15 04:35	48	0	31	96
19/05/15 04:40	24	0	27	84
19/05/15 04:45	24	0	0	60
19/05/15 04:50	36	0	12	156
19/05/15 04:55	24	0	24	144
19/05/15 05:00	48	0	15	12
19/05/15 05:05	60	0	67	108
19/05/15 05:10	24	0	0	144
19/05/15 05:15	60	0	48	204
19/05/15 05:20	36	0	46	180
19/05/15 05:25	72	0	79	168
19/05/15 05:30	60	0	94	144
19/05/15 05:35	72	0	60	216
19/05/15 05:40	168	0	70	288
19/05/15 05:45	192	0	82	276
19/05/15 05:50	300	0	46	288
19/05/15 05:55	216	0	74	456
19/05/15 06:00	300	0	79	324
19/05/15 06:05	312	0	154	432
19/05/15 06:10	396	0	170	456
19/05/15 06:15	360	0	150	600
19/05/15 06:20	624	0	186	636
19/05/15 06:25	636	0	191	708
19/05/15 06:30	816	0	198	828
19/05/15 06:35	1068	0	133	852
19/05/15 06:40	1428	0	259	1224
19/05/15 06:45	1548	0	378	1296
19/05/15 06:50	1908	0	490	1248
19/05/15 06:55	1872	0	658	1284
19/05/15 07:00	1716	0	618	1308

Sistema de Controle de Tráfego Urbano OPTIMUS

5 MINUTOS	INTENSIDADE			
	PM 040300 4	PM 040300 6	PM 040400 2	PM 040400 4
19/05/15 07:05	2040	0	578	1488
19/05/15 07:10	1932	0	549	1608
19/05/15 07:15	1872	0	708	1392
19/05/15 07:20	1800	0	666	1536
19/05/15 07:25	2016	0	501	1488
19/05/15 07:30	1740	0	499	1224
19/05/15 07:35	1872	0	392	1608
19/05/15 07:40	1932	0	585	1620
19/05/15 07:45	1824	0	414	1764
19/05/15 07:50	1740	0	440	1524
19/05/15 07:55	1908	0	544	1536
19/05/15 08:00	1740	36	595	1308
19/05/15 08:05	1500	180	399	1620
19/05/15 08:10	1596	180	627	1440
19/05/15 08:15	1356	216	430	1752
19/05/15 08:20	1488	360	474	1584
19/05/15 08:25	1452	360	505	1584
19/05/15 08:30	1392	396	446	1440
19/05/15 08:35	1584	540	459	1236
19/05/15 08:40	1788	540	443	1788
19/05/15 08:45	1476	576	540	1488
19/05/15 08:50	1668	720	482	1332
19/05/15 08:55	1224	720	496	1356
19/05/15 09:00	1488	720	466	1248
19/05/15 09:05	1152	720	525	1440
19/05/15 09:10	1272	720	645	1488
19/05/15 09:15	984	720	588	1416
19/05/15 09:20	1164	720	543	1116
19/05/15 09:25	1128	720	639	1416
19/05/15 09:30	1140	720	633	1428
19/05/15 09:35	1056	720	592	1380
19/05/15 09:40	1044	720	699	1308
19/05/15 09:45	984	720	643	1164
19/05/15 09:50	1056	720	475	1440
19/05/15 09:55	1068	720	571	1404
19/05/15 10:00	1008	720	631	1476
19/05/15 10:05	792	720	631	1140
19/05/15 10:10	744	720	655	1128
19/05/15 10:15	780	720	646	936
19/05/15 10:20	804	720	501	1320
19/05/15 10:25	840	720	669	996
19/05/15 10:30	912	720	606	1164
19/05/15 10:35	1056	720	589	1236
19/05/15 10:40	888	720	529	1392
19/05/15 10:45	852	720	631	1032
19/05/15 10:50	876	720	570	1068
19/05/15 10:55	948	720	584	900
19/05/15 11:00	576	720	696	1404
19/05/15 11:05	672	720	692	1092
19/05/15 11:10	756	720	554	1164
19/05/15 11:15	708	720	723	1176
19/05/15 11:20	612	720	644	900
19/05/15 11:25	768	720	818	1164
19/05/15 11:30	552	756	833	1032
19/05/15 11:35	744	900	728	1164
19/05/15 11:40	924	900	595	1260
19/05/15 11:45	840	900	734	1284
19/05/15 11:50	468	900	759	1056

Sistema de Controle de Tráfego Urbano OPTIMUS

5 MINUTOS	INTENSIDADE			
	PM 040300 4	PM 040300 6	PM 040400 2	PM 040400 4
19/05/15 11:55	816	900	798	1188
19/05/15 12:00	576	900	669	1272
19/05/15 12:05	792	900	651	1152
19/05/15 12:10	672	900	668	888
19/05/15 12:15	744	900	705	1308
19/05/15 12:20	492	900	642	1164
19/05/15 12:25	708	900	639	804
19/05/15 12:30	720	900	675	1248
19/05/15 12:35	852	900	751	1284
19/05/15 12:40	648	900	535	1128
19/05/15 12:45	912	900	578	1116
19/05/15 12:50	900	900	872	1044
19/05/15 12:55	1020	900	835	1044
19/05/15 13:00	1092	900	765	1416
19/05/15 13:05	936	900	730	1020
19/05/15 13:10	1092	900	790	1224
19/05/15 13:15	984	900	670	1260
19/05/15 13:20	1056	900	668	1284
19/05/15 13:25	1164	900	776	1068
19/05/15 13:30	948	900	589	1500
19/05/15 13:35	1248	900	657	1200
19/05/15 13:40	1104	900	706	1212
19/05/15 13:45	1068	900	619	1116
19/05/15 13:50	852	900	552	1236
19/05/15 13:55	1044	900	728	1404
19/05/15 14:00	660	900	589	1272
19/05/15 14:05	984	900	596	1272
19/05/15 14:10	1092	900	657	1224
19/05/15 14:15	960	900	579	1248
19/05/15 14:20	876	900	696	1284
19/05/15 14:25	864	900	654	1248
19/05/15 14:30	888	900	732	1056
19/05/15 14:35	780	900	687	1332
19/05/15 14:40	792	900	687	1200
19/05/15 14:45	840	900	573	1464
19/05/15 14:50	852	900	547	1380
19/05/15 14:55	828	900	649	1212
19/05/15 15:00	840	900	813	1008
19/05/15 15:05	828	900	512	1608
19/05/15 15:10	720	900	727	1104
19/05/15 15:15	684	900	644	1140
19/05/15 15:20	780	900	890	1032
19/05/15 15:25	648	900	579	1224
19/05/15 15:30	876	900	711	1296
19/05/15 15:35	768	900	542	1152
19/05/15 15:40	600	900	795	1260
19/05/15 15:45	840	900	656	1224
19/05/15 15:50	852	900	666	1296
19/05/15 15:55	804	900	667	1260
19/05/15 16:00	624	900	868	984
19/05/15 16:05	708	900	724	1092
19/05/15 16:10	840	900	830	1140
19/05/15 16:15	768	900	765	1092
19/05/15 16:20	924	900	878	1224
19/05/15 16:25	780	900	781	1200
19/05/15 16:30	684	900	862	1128
19/05/15 16:35	624	900	802	1164
19/05/15 16:40	648	900	925	1188

Sistema de Controle de Tráfego Urbano OPTIMUS

5 MINUTOS	INTENSIDADE			
	PM 0403004	PM 0403006	PM 0404002	PM 0404004
19/05/15 16:45	576	900	644	1236
19/05/15 16:50	852	900	709	984
19/05/15 16:55	732	900	790	1308
19/05/15 17:00	768	900	874	972
19/05/15 17:05	648	900	927	768
19/05/15 17:10	1032	900	534	624
19/05/15 17:15	540	900	261	600
19/05/15 17:20	684	900	118	792
19/05/15 17:25	408	900	219	408
19/05/15 17:30	1152	900	332	552
19/05/15 17:35	768	900	228	744
19/05/15 17:40	672	900	223	648
19/05/15 17:45	840	936	280	972
19/05/15 17:50	828	1080	484	972
19/05/15 17:55	1128	1080	138	1392
19/05/15 18:00	924	1044	316	1104
19/05/15 18:05	876	900	634	1200
19/05/15 18:10	708	900	550	1104
19/05/15 18:15	708	900	535	804
19/05/15 18:20	852	900	457	708
19/05/15 18:25	852	900	572	936
19/05/15 18:30	768	900	337	804
19/05/15 18:35	900	900	212	1008
19/05/15 18:40	768	900	418	564
19/05/15 18:45	768	900	359	252
19/05/15 18:50	612	900	332	1032
19/05/15 18:55	768	900	436	792
19/05/15 19:00	744	900	525	996
19/05/15 19:05	720	900	612	828
19/05/15 19:10	660	900	689	912
19/05/15 19:15	516	900	574	1080
19/05/15 19:20	588	900	727	696
19/05/15 19:25	576	900	599	876
19/05/15 19:30	528	900	574	1020
19/05/15 19:35	552	900	424	1224
19/05/15 19:40	456	900	626	756
19/05/15 19:45	540	900	525	852
19/05/15 19:50	432	900	490	900
19/05/15 19:55	384	900	448	828
19/05/15 20:00	396	900	458	936
19/05/15 20:05	444	900	447	912
19/05/15 20:10	480	900	499	756
19/05/15 20:15	384	936	351	924
19/05/15 20:20	372	1080	333	732
19/05/15 20:25	504	1080	314	792
19/05/15 20:30	300	1080	331	972
19/05/15 20:35	444	1080	338	708
19/05/15 20:40	312	1080	541	708
19/05/15 20:45	300	1044	399	816
19/05/15 20:50	492	900	457	636
19/05/15 20:55	324	900	294	744
19/05/15 21:00	324	900	498	624
19/05/15 21:05	492	900	342	756
19/05/15 21:10	264	900	321	696
19/05/15 21:15	300	900	483	600
19/05/15 21:20	360	900	463	672
19/05/15 21:25	336	900	484	948
19/05/15 21:30	228	900	164	552

Sistema de Controle de Tráfego Urbano OPTIMUS

5 MINUTOS	INTENSIDADE			
	PM 0403004	PM 0403006	PM 0404002	PM 0404004
19/05/15 21:35	300	900	266	552
19/05/15 21:40	264	900	255	636
19/05/15 21:45	264	864	344	720
19/05/15 21:50	336	720	280	612
19/05/15 21:55	276	720	325	804
19/05/15 22:00	312	720	406	696
19/05/15 22:05	312	720	414	684
19/05/15 22:10	420	720	552	804
19/05/15 22:15	312	720	439	864
19/05/15 22:20	288	720	606	768
19/05/15 22:25	228	720	621	756
19/05/15 22:30	264	720	694	804
19/05/15 22:35	288	720	744	804
19/05/15 22:40	336	720	558	708
19/05/15 22:45	204	720	410	660
19/05/15 22:50	216	720	403	588
19/05/15 22:55	180	720	255	444
19/05/15 23:00	216	720	261	744
19/05/15 23:05	204	720	319	432
19/05/15 23:10	252	720	180	312
19/05/15 23:15	108	720	184	468
19/05/15 23:20	84	720	106	432
19/05/15 23:25	336	720	70	348
19/05/15 23:30	168	684	193	348
19/05/15 23:35	192	540	58	240
19/05/15 23:40	72	540	134	360
19/05/15 23:45	84	504	94	288
19/05/15 23:50	156	360	103	312
19/05/15 23:55	108	360	114	300
20/05/15 00:00	192	324	122	228
20/05/15 00:05	156	180	87	300
20/05/15 00:10	120	180	86	204
20/05/15 00:15	180	180	66	276
20/05/15 00:20	72	180	123	192
20/05/15 00:25	24	180	151	168
20/05/15 00:30	60	180	106	192
20/05/15 00:35	108	180	79	156
20/05/15 00:40	120	180	78	120
20/05/15 00:45	24	180	134	192
20/05/15 00:50	12	180	48	132
20/05/15 00:55	72	180	31	192
20/05/15 01:00	60	180	27	156
20/05/15 01:05	36	180	111	144
20/05/15 01:10	72	180	31	132
20/05/15 01:15	48	180	31	132
20/05/15 01:20	24	180	27	108
20/05/15 01:25	36	180	27	60
20/05/15 01:30	12	180	51	72
20/05/15 01:35	60	180	12	96
20/05/15 01:40	0	180	55	96
20/05/15 01:45	12	144	24	72
20/05/15 01:50	24	0	15	84
20/05/15 01:55	12	0	36	60
20/05/15 02:00	36	0	36	156
20/05/15 02:05	12	0	12	72
20/05/15 02:10	0	0	15	36
20/05/15 02:15	36	0	12	84
20/05/15 02:20	36	0	36	84

Sistema de Controle de Tráfego Urbano OPTIMUS

5 MINUTOS	INTENSIDADE			
	PM 040300 4	PM 040300 6	PM 040400 2	PM 040400 4
20/05/15 02:25	24	0	36	36
20/05/15 02:30	24	0	51	108
20/05/15 02:35	0	0	0	48
20/05/15 02:40	12	0	43	60
20/05/15 02:45	24	0	36	60
20/05/15 02:50	12	0	12	36
20/05/15 02:55	36	0	24	72
20/05/15 03:00	0	0	34	48
20/05/15 03:05	24	0	3	60
20/05/15 03:10	12	0	12	60
20/05/15 03:15	24	0	48	48
20/05/15 03:20	24	0	15	132
20/05/15 03:25	12	0	0	12
20/05/15 03:30	12	0	15	36
20/05/15 03:35	12	0	36	36
20/05/15 03:40	0	0	12	0
20/05/15 03:45	12	0	0	24
20/05/15 03:50	24	0	0	72
20/05/15 03:55	12	0	0	36
20/05/15 04:00	24	0	0	60
20/05/15 04:05	36	0	63	72
20/05/15 04:10	60	0	27	72
20/05/15 04:15	12	0	31	108
20/05/15 04:20	0	0	0	48
20/05/15 04:25	24	0	12	48
20/05/15 04:30	12	0	19	84
20/05/15 04:35	12	0	24	84
20/05/15 04:40	60	0	19	48
20/05/15 04:45	24	0	7	84
20/05/15 04:50	72	0	27	156
20/05/15 04:55	24	0	19	84
20/05/15 05:00	36	0	51	84
20/05/15 05:05	72	0	39	108
20/05/15 05:10	60	0	12	192
20/05/15 05:15	84	0	24	144
20/05/15 05:20	36	0	48	168
20/05/15 05:25	24	0	51	132
20/05/15 05:30	60	0	43	204
20/05/15 05:35	120	0	75	204
20/05/15 05:40	96	0	103	240
20/05/15 05:45	144	0	99	132
20/05/15 05:50	276	0	115	348
20/05/15 05:55	264	0	141	420
20/05/15 06:00	288	0	120	432
20/05/15 06:05	276	0	145	408
20/05/15 06:10	240	0	160	348
20/05/15 06:15	444	0	134	504
20/05/15 06:20	372	0	178	588
20/05/15 06:25	696	0	174	576
20/05/15 06:30	684	0	204	840
20/05/15 06:35	924	0	193	900
20/05/15 06:40	1176	0	308	1008
20/05/15 06:45	1524	0	307	1236
20/05/15 06:50	1908	0	426	1236
20/05/15 06:55	1608	0	596	1320
20/05/15 07:00	1740	0	369	1356
20/05/15 07:05	1728	0	619	1404
20/05/15 07:10	2052	0	708	1512

Sistema de Controle de Tráfego Urbano OPTIMUS

5 MINUTOS	INTENSIDADE			
	PM 040300 4	PM 040300 6	PM 040400 2	PM 040400 4
20/05/15 07:15	1668	0	657	1620
20/05/15 07:20	1896	0	634	1440
20/05/15 07:25	1824	0	493	1572
20/05/15 07:30	2052	0	558	1536
20/05/15 07:35	1836	0	525	1716
20/05/15 07:40	1776	0	572	1560
20/05/15 07:45	1656	0	381	1824
20/05/15 07:50	1860	0	428	1596
20/05/15 07:55	1632	0	558	1392
20/05/15 08:00	1632	0	439	1392
20/05/15 08:05	1284	0	478	1416
20/05/15 08:10	1140	0	520	1392
20/05/15 08:15	1464	0	493	1416
20/05/15 08:20	1512	0	379	1392
20/05/15 08:25	1344	0	534	1428
20/05/15 08:30	1416	36	517	1524
20/05/15 08:35	1248	180	396	1404
20/05/15 08:40	1320	180	566	1260
20/05/15 08:45	1368	216	548	1404
20/05/15 08:50	1656	360	447	1620
20/05/15 08:55	1716	360	424	1716
20/05/15 09:00	1584	396	573	1548
20/05/15 09:05	1632	540	656	1524
20/05/15 09:10	1536	540	448	1692
20/05/15 09:15	1356	576	501	1656
20/05/15 09:20	1572	720	730	1548
20/05/15 09:25	1116	720	518	1224
20/05/15 09:30	1368	720	709	1116
20/05/15 09:35	1284	720	685	1332
20/05/15 09:40	960	720	674	1332
20/05/15 09:45	1200	720	595	1044
20/05/15 09:50	1032	720	591	1356
20/05/15 09:55	792	720	697	1236
20/05/15 10:00	1164	720	432	1224
20/05/15 10:05	996	720	544	1404
20/05/15 10:10	732	720	697	1116
20/05/15 10:15	876	720	580	1320
20/05/15 10:20	936	720	703	972
20/05/15 10:25	636	720	675	1104
20/05/15 10:30	828	720	603	1224
20/05/15 10:35	900	720	668	1260
20/05/15 10:40	468	720	475	1188
20/05/15 10:45	780	720	565	1224
20/05/15 10:50	576	720	730	1284
20/05/15 10:55	780	720	753	1092
20/05/15 11:00	840	720	576	1272
20/05/15 11:05	672	720	596	1260
20/05/15 11:10	660	720	606	1272
20/05/15 11:15	744	756	723	936
20/05/15 11:20	612	900	684	1200
20/05/15 11:25	816	900	645	1344
20/05/15 11:30	828	900	847	1068
20/05/15 11:35	660	900	858	1260
20/05/15 11:40	684	900	632	1092
20/05/15 11:45	696	900	706	828
20/05/15 11:50	684	900	763	1368
20/05/15 11:55	948	900	765	1164
20/05/15 12:00	624	900	698	876

Sistema de Controle de Tráfego Urbano OPTIMUS

5 MINUTOS	INTENSIDADE			
	PM 040300 4	PM 040300 6	PM 040400 2	PM 040400 4
20/05/15 12:05	720	900	734	804
20/05/15 12:10	696	900	643	1008
20/05/15 12:15	732	900	771	1212
20/05/15 12:20	624	900	862	1056
20/05/15 12:25	720	900	685	1212
20/05/15 12:30	840	900	723	972
20/05/15 12:35	828	900	610	1188
20/05/15 12:40	732	900	631	1236
20/05/15 12:45	816	900	759	1164
20/05/15 12:50	972	900	826	1044
20/05/15 12:55	1128	900	848	1392
20/05/15 13:00	1056	900	776	996
20/05/15 13:05	1044	900	848	1200
20/05/15 13:10	804	900	778	1308
20/05/15 13:15	1020	900	762	1368
20/05/15 13:20	1056	900	613	1032
20/05/15 13:25	876	900	531	1020
20/05/15 13:30	1104	900	626	1404
20/05/15 13:35	996	900	535	1308
20/05/15 13:40	924	900	655	1308
20/05/15 13:45	1140	936	632	1560
20/05/15 13:50	792	1080	613	1152
20/05/15 13:55	900	1080	552	1440
20/05/15 14:00	888	1080	514	1380
20/05/15 14:05	1020	1080	614	1416
20/05/15 14:10	852	1080	594	1356
20/05/15 14:15	936	1080	550	1044
20/05/15 14:20	972	1080	645	1476
20/05/15 14:25	1128	1080	752	1332
20/05/15 14:30	840	1044	542	1260
20/05/15 14:35	912	900	660	1416
20/05/15 14:40	840	900	682	1140
20/05/15 14:45	876	900	542	1092
20/05/15 14:50	780	900	603	1392
20/05/15 14:55	888	900	555	1416
20/05/15 15:00	636	900	616	1296
20/05/15 15:05	900	900	629	1344
20/05/15 15:10	804	900	607	1068
20/05/15 15:15	912	900	597	948
20/05/15 15:20	948	900	777	1140
20/05/15 15:25	720	900	566	1320
20/05/15 15:30	816	900	801	1020
20/05/15 15:35	792	900	678	1380
20/05/15 15:40	744	900	830	1008
20/05/15 15:45	648	900	498	1044
20/05/15 15:50	708	900	769	1404
20/05/15 15:55	840	900	565	1164
20/05/15 16:00	804	900	592	1068
20/05/15 16:05	660	900	565	1020
20/05/15 16:10	888	900	804	1128
20/05/15 16:15	744	900	796	984
20/05/15 16:20	804	900	735	1284
20/05/15 16:25	696	900	685	1200
20/05/15 16:30	828	900	1046	1104
20/05/15 16:35	552	900	776	1284
20/05/15 16:40	708	900	770	1392
20/05/15 16:45	696	936	715	1344
20/05/15 16:50	816	1080	948	1032

Sistema de Controle de Tráfego Urbano OPTIMUS

5 MINUTOS	INTENSIDADE			
	PM 040300 4	PM 040300 6	PM 040400 2	PM 040400 4
20/05/15 16:55	768	1080	858	1308
20/05/15 17:00	720	1080	987	1080
20/05/15 17:05	744	1080	891	1056
20/05/15 17:10	708	1080	987	1224
20/05/15 17:15	816	1080	796	1296
20/05/15 17:20	864	1080	528	828
20/05/15 17:25	744	1080	297	1152
20/05/15 17:30	852	1116	394	924
20/05/15 17:35	516	1260	330	732
20/05/15 17:40	732	1260	591	900
20/05/15 17:45	768	1260	697	936
20/05/15 17:50	684	1260	806	912
20/05/15 17:55	612	1260	609	1092
20/05/15 18:00	780	1260	707	900
20/05/15 18:05	852	1260	759	996
20/05/15 18:10	828	1260	793	1104
20/05/15 18:15	636	1260	577	948
20/05/15 18:20	720	1260	893	912
20/05/15 18:25	780	1260	570	852
20/05/15 18:30	684	1224	837	720
20/05/15 18:35	804	1080	550	984
20/05/15 18:40	540	1080	722	768
20/05/15 18:45	732	1080	541	876
20/05/15 18:50	624	1080	546	1068
20/05/15 18:55	684	1080	604	840
20/05/15 19:00	696	1080	562	900
20/05/15 19:05	660	1080	526	960
20/05/15 19:10	684	1080	692	852
20/05/15 19:15	588	1080	684	960
20/05/15 19:20	576	1080	644	1080
20/05/15 19:25	768	1080	585	900
20/05/15 19:30	540	1080	613	1068
20/05/15 19:35	396	1080	429	840
20/05/15 19:40	528	1080	712	936
20/05/15 19:45	360	1080	482	816
20/05/15 19:50	480	1080	369	828
20/05/15 19:55	504	1080	505	1152
20/05/15 20:00	444	1080	483	672
20/05/15 20:05	492	1080	326	972
20/05/15 20:10	420	1080	579	960
20/05/15 20:15	540	1044	262	684
20/05/15 20:20	360	900	422	876
20/05/15 20:25	312	900	435	732
20/05/15 20:30	516	900	434	792
20/05/15 20:35	432	900	339	696
20/05/15 20:40	300	900	433	636
20/05/15 20:45	516	900	468	600
20/05/15 20:50	384	900	514	804
20/05/15 20:55	348	900	326	816
20/05/15 21:00	300	900	302	720
20/05/15 21:05	276	900	487	804
20/05/15 21:10	420	900	436	816
20/05/15 21:15	372	900	514	828
20/05/15 21:20	372	900	351	744
20/05/15 21:25	228	900	403	804
20/05/15 21:30	432	900	355	684
20/05/15 21:35	252	900	418	684
20/05/15 21:40	420	900	332	624

Sistema de Controle de Tráfego Urbano OPTIMUS

5 MINUTOS	INTENSIDADE			
	PM 0403004	PM 0403006	PM 0404002	PM 0404004
20/05/15 21:45	264	864	517	624
20/05/15 21:50	408	720	362	720
20/05/15 21:55	396	720	369	732
20/05/15 22:00	252	720	315	720
20/05/15 22:05	396	720	403	720
20/05/15 22:10	288	720	510	600
20/05/15 22:15	336	720	474	720
20/05/15 22:20	276	720	433	804
20/05/15 22:25	264	720	583	588
20/05/15 22:30	408	720	595	552
20/05/15 22:35	300	720	613	780
20/05/15 22:40	276	720	508	564
20/05/15 22:45	120	720	409	600
20/05/15 22:50	192	720	303	480
20/05/15 22:55	348	720	283	420
20/05/15 23:00	240	720	246	468
20/05/15 23:05	240	720	336	432
20/05/15 23:10	180	720	342	396
20/05/15 23:15	120	684	90	432
20/05/15 23:20	216	540	85	396
20/05/15 23:25	216	540	146	324
20/05/15 23:30	324	504	150	492
20/05/15 23:35	264	360	102	372
20/05/15 23:40	168	360	94	348
20/05/15 23:45	144	360	58	276
20/05/15 23:50	144	360	58	384
20/05/15 23:55	84	360	46	276
21/05/15 00:00	132	324	146	264
21/05/15 00:05	120	180	115	420
21/05/15 00:10	96	180	134	264
21/05/15 00:15	48	180	62	192
21/05/15 00:20	72	180	99	228
21/05/15 00:25	96	180	43	192
21/05/15 00:30	48	180	99	156
21/05/15 00:35	60	180	75	156
21/05/15 00:40	36	180	66	168
21/05/15 00:45	60	180	74	192
21/05/15 00:50	48	180	63	108
21/05/15 00:55	24	180	36	156
21/05/15 01:00	60	180	51	192
21/05/15 01:05	12	180	36	120
21/05/15 01:10	84	180	39	192
21/05/15 01:15	48	144	15	168
21/05/15 01:20	48	0	51	168
21/05/15 01:25	48	0	31	120
21/05/15 01:30	48	0	60	132
21/05/15 01:35	72	0	63	132
21/05/15 01:40	48	0	48	84
21/05/15 01:45	36	0	27	120
21/05/15 01:50	48	0	46	84
21/05/15 01:55	36	0	7	84
21/05/15 02:00	48	0	24	84
21/05/15 02:05	0	0	72	60
21/05/15 02:10	12	0	27	12
21/05/15 02:15	12	0	12	72
21/05/15 02:20	24	0	15	72
21/05/15 02:25	36	0	36	84
21/05/15 02:30	36	0	15	156

Sistema de Controle de Tráfego Urbano OPTIMUS

5 MINUTOS	INTENSIDADE			
	PM 0403004	PM 0403006	PM 0404002	PM 0404004
21/05/15 02:35	36	0	27	84
21/05/15 02:40	36	0	27	72
21/05/15 02:45	36	0	27	12
21/05/15 02:50	12	0	15	48
21/05/15 02:55	0	0	27	36
21/05/15 03:00	12	0	3	36
21/05/15 03:05	36	0	12	84
21/05/15 03:10	12	0	24	60
21/05/15 03:15	0	0	12	60
21/05/15 03:20	48	0	31	48
21/05/15 03:25	0	0	12	48
21/05/15 03:30	36	0	12	72
21/05/15 03:35	12	0	12	60
21/05/15 03:40	24	0	36	24
21/05/15 03:45	24	0	31	72
21/05/15 03:50	12	0	0	60
21/05/15 03:55	12	0	3	48
21/05/15 04:00	12	0	24	96
21/05/15 04:05	24	0	0	24
21/05/15 04:10	0	0	12	48
21/05/15 04:15	0	0	39	108
21/05/15 04:20	24	0	12	168
21/05/15 04:25	36	0	24	108
21/05/15 04:30	36	0	31	48
21/05/15 04:35	48	0	63	108
21/05/15 04:40	60	0	24	132
21/05/15 04:45	48	0	43	96
21/05/15 04:50	24	0	27	120
21/05/15 04:55	48	0	36	192
21/05/15 05:00	24	0	19	84
21/05/15 05:05	96	0	36	132
21/05/15 05:10	24	0	55	132
21/05/15 05:15	72	0	15	120
21/05/15 05:20	36	0	36	132
21/05/15 05:25	36	0	102	144
21/05/15 05:30	72	0	43	276
21/05/15 05:35	60	0	108	216
21/05/15 05:40	144	0	70	264
21/05/15 05:45	180	0	79	276
21/05/15 05:50	192	0	43	324
21/05/15 05:55	216	0	58	444
21/05/15 06:00	300	0	86	300
21/05/15 06:05	288	0	122	348
21/05/15 06:10	384	0	106	432
21/05/15 06:15	228	0	147	540
21/05/15 06:20	492	0	132	348
21/05/15 06:25	672	0	153	756
21/05/15 06:30	672	0	222	900
21/05/15 06:35	972	0	215	996
21/05/15 06:40	1464	0	358	948
21/05/15 06:45	1584	0	400	1224
21/05/15 06:50	1740	0	466	1092
21/05/15 06:55	1716	0	586	840
21/05/15 07:00	1392	0	527	1416
21/05/15 07:05	1524	0	459	1332
21/05/15 07:10	1332	0	667	1368
21/05/15 07:15	1704	0	597	1044
21/05/15 07:20	840	0	574	1380

Sistema de Controle de Tráfego Urbano OPTIMUS

5 MINUTOS	INTENSIDADE			
	PM 040300 4	PM 040300 6	PM 040400 2	PM 040400 4
21/05/15 07:25	1188	0	450	1536
21/05/15 07:30	1824	0	460	1344
21/05/15 07:35	1596	0	374	1464
21/05/15 07:40	1944	0	484	1560
21/05/15 07:45	1500	0	276	1608
21/05/15 07:50	1860	0	289	2016
21/05/15 07:55	2148	0	577	1860
21/05/15 08:00	2028	0	488	1620
21/05/15 08:05	1968	0	586	1416
21/05/15 08:10	1740	0	435	1668
21/05/15 08:15	1620	36	504	1500
21/05/15 08:20	1608	180	612	1692
21/05/15 08:25	1524	180	396	1344
21/05/15 08:30	1800	180	444	1464
21/05/15 08:35	1620	180	594	1656
21/05/15 08:40	1572	180	537	1848
21/05/15 08:45	1596	216	697	1428
21/05/15 08:50	1980	360	501	1584
21/05/15 08:55	1644	360	650	1560
21/05/15 09:00	1656	396	615	1536
21/05/15 09:05	1716	540	622	1680
21/05/15 09:10	1776	540	648	1488
21/05/15 09:15	1488	576	525	1548
21/05/15 09:20	1296	720	722	1272
21/05/15 09:25	1248	720	601	1488
21/05/15 09:30	1308	720	699	1524
21/05/15 09:35	1296	720	536	1368
21/05/15 09:40	996	720	574	1164
21/05/15 09:45	1176	720	554	1284
21/05/15 09:50	1116	720	595	1488
21/05/15 09:55	1188	720	616	1296
21/05/15 10:00	1200	720	554	1296
21/05/15 10:05	1080	720	434	1296
21/05/15 10:10	816	720	537	1056
21/05/15 10:15	948	720	578	1068
21/05/15 10:20	732	720	469	1188
21/05/15 10:25	804	720	613	1032
21/05/15 10:30	720	720	629	1440
21/05/15 10:35	648	720	622	1284
21/05/15 10:40	720	720	633	1056
21/05/15 10:45	636	720	704	972
21/05/15 10:50	816	720	788	1272
21/05/15 10:55	792	720	591	1068
21/05/15 11:00	732	756	768	1248
21/05/15 11:05	672	900	526	1008
21/05/15 11:10	672	900	631	996
21/05/15 11:15	876	900	751	1488
21/05/15 11:20	636	900	631	1080
21/05/15 11:25	780	900	661	1044
21/05/15 11:30	600	900	802	1356
21/05/15 11:35	804	900	642	1236
21/05/15 11:40	684	900	561	1020
21/05/15 11:45	924	900	745	1464
21/05/15 11:50	636	900	876	1248
21/05/15 11:55	708	900	915	984
21/05/15 12:00	696	900	759	936
21/05/15 12:05	732	900	738	1260
21/05/15 12:10	612	900	780	960

5 MINUTOS	INTENSIDADE			
	PM 040300 4	PM 040300 6	PM 040400 2	PM 040400 4
21/05/15 12:15	708	900	724	1152
21/05/15 12:20	576	900	805	1032
21/05/15 12:25	720	900	670	1128
21/05/15 12:30	756	936	649	1260
21/05/15 12:35	792	1080	669	1380
21/05/15 12:40	804	1080	537	888
21/05/15 12:45	1032	1080	672	1284
21/05/15 12:50	852	1080	811	1164
21/05/15 12:55	1020	1080	789	1008
21/05/15 13:00	1164	1080	812	1152
21/05/15 13:05	1020	1080	842	1284
21/05/15 13:10	912	1080	600	1116
21/05/15 13:15	1140	1080	468	1392
21/05/15 13:20	876	1080	478	1536
21/05/15 13:25	1092	1080	762	1224
21/05/15 13:30	1032	1080	535	1440
21/05/15 13:35	972	1080	646	1248
21/05/15 13:40	1008	1080	648	1284
21/05/15 13:45	924	1080	703	1260
21/05/15 13:50	1008	1080	621	1164
21/05/15 13:55	1056	1080	748	1200
21/05/15 14:00	984	1080	784	1344
21/05/15 14:05	984	1080	571	1236
21/05/15 14:10	912	1080	814	1200
21/05/15 14:15	948	1080	806	1140
21/05/15 14:20	948	1080	579	1260
21/05/15 14:25	1056	1080	613	1620
21/05/15 14:30	864	1080	554	1200
21/05/15 14:35	1116	1080	697	1188
21/05/15 14:40	804	1080	896	1260
21/05/15 14:45	1008	1080	742	1296
21/05/15 14:50	756	1080	915	1212
21/05/15 14:55	816	1080	559	1236
21/05/15 15:00	792	1080	738	1008
21/05/15 15:05	792	1080	664	1080
21/05/15 15:10	804	1080	850	1452
21/05/15 15:15	804	1080	505	1212
21/05/15 15:20	840	1080	781	948
21/05/15 15:25	804	1080	790	1332
21/05/15 15:30	768	1080	786	1296
21/05/15 15:35	732	1080	549	1092
21/05/15 15:40	900	1080	811	1296
21/05/15 15:45	732	1080	727	1212
21/05/15 15:50	780	1080	717	1020
21/05/15 15:55	804	1080	728	1140
21/05/15 16:00	660	1080	945	1104
21/05/15 16:05	732	1080	697	1104
21/05/15 16:10	732	1080	934	1320
21/05/15 16:15	540	1080	763	1260
21/05/15 16:20	744	1080	888	900
21/05/15 16:25	660	1080	819	1188
21/05/15 16:30	732	1080	816	1164
21/05/15 16:35	732	1080	744	1164
21/05/15 16:40	708	1080	1002	1104
21/05/15 16:45	780	1080	720	1260
21/05/15 16:50	756	1080	930	972
21/05/15 16:55	768	1080	620	1008
21/05/15 17:00	636	1080	824	1260

Sistema de Controle de Tráfego Urbano OPTIMUS

5 MINUTOS	INTENSIDADE			
	PM 0403004	PM 0403006	PM 0404002	PM 0404004
21/05/15 17:05	792	1080	758	936
21/05/15 17:10	864	1080	859	1068
21/05/15 17:15	888	1080	262	960
21/05/15 17:20	768	1080	286	300
21/05/15 17:25	600	1080	370	540
21/05/15 17:30	936	1080	441	504
21/05/15 17:35	624	1080	330	660
21/05/15 17:40	648	1080	336	636
21/05/15 17:45	648	1080	185	600
21/05/15 17:50	1152	1080	241	624
21/05/15 17:55	600	1080	255	900
21/05/15 18:00	744	1080	228	696
21/05/15 18:05	636	1080	352	708
21/05/15 18:10	408	1080	222	948
21/05/15 18:15	780	1080	430	1176
21/05/15 18:20	396	1080	324	1380
21/05/15 18:25	708	1080	357	900
21/05/15 18:30	756	1044	285	672
21/05/15 18:35	732	900	240	684
21/05/15 18:40	576	900	393	492
21/05/15 18:45	444	864	425	696
21/05/15 18:50	732	720	387	708
21/05/15 18:55	840	720	291	696
21/05/15 19:00	348	720	517	672
21/05/15 19:05	912	720	442	1068
21/05/15 19:10	456	720	470	1308
21/05/15 19:15	852	720	722	972
21/05/15 19:20	720	720	669	1128
21/05/15 19:25	672	720	687	996
21/05/15 19:30	696	720	645	1068
21/05/15 19:35	588	720	494	948
21/05/15 19:40	540	720	546	960
21/05/15 19:45	504	720	487	744
21/05/15 19:50	612	720	542	864
21/05/15 19:55	564	720	472	924
21/05/15 20:00	600	720	603	1056
21/05/15 20:05	444	720	368	900
21/05/15 20:10	492	720	514	876
21/05/15 20:15	432	720	615	1092
21/05/15 20:20	420	720	508	852
21/05/15 20:25	516	720	324	828
21/05/15 20:30	408	720	541	864
21/05/15 20:35	408	720	372	780
21/05/15 20:40	408	720	658	780
21/05/15 20:45	312	720	381	864
21/05/15 20:50	336	720	504	900
21/05/15 20:55	360	720	288	912
21/05/15 21:00	300	720	352	828
21/05/15 21:05	408	720	265	924
21/05/15 21:10	252	720	309	816
21/05/15 21:15	408	756	385	852
21/05/15 21:20	360	900	466	756
21/05/15 21:25	288	900	588	888
21/05/15 21:30	444	900	316	756
21/05/15 21:35	252	900	366	756
21/05/15 21:40	276	900	324	540
21/05/15 21:45	240	864	425	828
21/05/15 21:50	276	720	282	708

Sistema de Controle de Tráfego Urbano OPTIMUS

5 MINUTOS	INTENSIDADE			
	PM 040300 4	PM 040300 6	PM 040400 2	PM 040400 4
21/05/15 21:55	276	720	422	636
21/05/15 22:00	312	720	388	648
21/05/15 22:05	252	720	603	756
21/05/15 22:10	348	720	619	576
21/05/15 22:15	276	720	452	732
21/05/15 22:20	384	720	552	792
21/05/15 22:25	264	720	488	660
21/05/15 22:30	336	720	577	864
21/05/15 22:35	240	720	519	768
21/05/15 22:40	192	720	443	660
21/05/15 22:45	228	720	286	468
21/05/15 22:50	216	720	313	492
21/05/15 22:55	264	720	337	456
21/05/15 23:00	240	720	264	588
21/05/15 23:05	144	720	159	408
21/05/15 23:10	144	720	156	504
21/05/15 23:15	204	684	170	456
21/05/15 23:20	240	540	136	420
21/05/15 23:25	144	540	138	408
21/05/15 23:30	120	504	121	444
21/05/15 23:35	228	360	98	216
21/05/15 23:40	120	360	146	216
21/05/15 23:45	48	360	79	264
21/05/15 23:50	144	360	78	288
21/05/15 23:55	192	360	122	360
22/05/15 00:00	192	360	141	444
22/05/15 00:05	180	360	86	348
22/05/15 00:10	192	360	189	396
22/05/15 00:15	96	360	115	420
22/05/15 00:20	144	360	66	504
22/05/15 00:25	180	360	211	348
22/05/15 00:30	120	324	250	420
22/05/15 00:35	36	180	118	324
22/05/15 00:40	156	180	182	144
22/05/15 00:45	48	180	151	252
22/05/15 00:50	84	180	86	276
22/05/15 00:55	84	180	57	180
22/05/15 01:00	72	180	122	180
22/05/15 01:05	96	180	33	240
22/05/15 01:10	132	180	58	264
22/05/15 01:15	60	180	51	228
22/05/15 01:20	96	180	15	228
22/05/15 01:25	72	180	36	144
22/05/15 01:30	24	180	115	132
22/05/15 01:35	96	180	24	156
22/05/15 01:40	36	180	75	108
22/05/15 01:45	36	144	34	168
22/05/15 01:50	48	0	19	156
22/05/15 01:55	24	0	58	72
22/05/15 02:00	60	0	27	204
22/05/15 02:05	36	0	31	96
22/05/15 02:10	48	0	75	84
22/05/15 02:15	24	0	43	48
22/05/15 02:20	12	0	60	96
22/05/15 02:25	36	0	12	144
22/05/15 02:30	48	0	24	132
22/05/15 02:35	36	0	12	60
22/05/15 02:40	48	0	24	72

Sistema de Controle de Tráfego Urbano OPTIMUS

5 MINUTOS	INTENSIDADE			
	PM 040300 4	PM 040300 6	PM 040400 2	PM 040400 4
22/05/15 02:45	36	0	43	48
22/05/15 02:50	24	0	3	72
22/05/15 02:55	12	0	48	108
22/05/15 03:00	12	0	15	108
22/05/15 03:05	24	0	43	84
22/05/15 03:10	48	0	0	60
22/05/15 03:15	0	0	36	72
22/05/15 03:20	48	0	19	72
22/05/15 03:25	24	0	24	48
22/05/15 03:30	12	0	39	108
22/05/15 03:35	0	0	12	48
22/05/15 03:40	0	0	36	48
22/05/15 03:45	36	0	43	36
22/05/15 03:50	24	0	19	84
22/05/15 03:55	12	0	72	36
22/05/15 04:00	24	0	0	84
22/05/15 04:05	24	0	24	84
22/05/15 04:10	0	0	22	24
22/05/15 04:15	24	0	39	108
22/05/15 04:20	36	0	19	84
22/05/15 04:25	12	0	24	60
22/05/15 04:30	36	0	15	96
22/05/15 04:35	0	0	39	84
22/05/15 04:40	60	0	12	120
22/05/15 04:45	60	0	26	120
22/05/15 04:50	24	0	3	120
22/05/15 04:55	48	0	27	132
22/05/15 05:00	36	0	27	108
22/05/15 05:05	60	0	27	108
22/05/15 05:10	48	0	51	180
22/05/15 05:15	72	0	36	168
22/05/15 05:20	36	0	79	192
22/05/15 05:25	60	0	62	204
22/05/15 05:30	60	0	43	252
22/05/15 05:35	132	0	79	168
22/05/15 05:40	120	0	96	276
22/05/15 05:45	144	0	55	276
22/05/15 05:50	156	0	79	300
22/05/15 05:55	240	0	118	384
22/05/15 06:00	240	0	135	312
22/05/15 06:05	432	0	98	468
22/05/15 06:10	348	0	201	504
22/05/15 06:15	396	0	190	600
22/05/15 06:20	480	0	153	588
22/05/15 06:25	468	0	193	552
22/05/15 06:30	900	0	146	696
22/05/15 06:35	1104	0	277	1176
22/05/15 06:40	1080	0	224	936
22/05/15 06:45	1596	0	318	1344
22/05/15 06:50	1896	0	547	1404
22/05/15 06:55	1512	0	504	1212
22/05/15 07:00	1776	0	544	1224
22/05/15 07:05	1944	0	561	1476
22/05/15 07:10	2064	0	622	1692
22/05/15 07:15	1848	0	673	1572
22/05/15 07:20	1752	0	607	1548
22/05/15 07:25	1848	0	472	1404
22/05/15 07:30	1752	0	535	1656

Sistema de Controle de Tráfego Urbano OPTIMUS

5 MINUTOS	INTENSIDADE			
	PM040300 4	PM040300 6	PM040400 2	PM040400 4
22/05/15 07:35	1764	0	501	1608
22/05/15 07:40	1728	0	426	1716
22/05/15 07:45	1920	0	460	1404
22/05/15 07:50	1764	0	306	1692
22/05/15 07:55	1848	0	619	1668
22/05/15 08:00	1824	0	477	1452
22/05/15 08:05	1956	0	542	1632
22/05/15 08:10	1716	0	506	1272
22/05/15 08:15	1440	36	548	1620
22/05/15 08:20	1680	180	350	1068
22/05/15 08:25	1368	180	559	1584
22/05/15 08:30	1704	216	459	1428
22/05/15 08:35	1464	360	483	1476
22/05/15 08:40	1476	360	499	1572
22/05/15 08:45	1392	396	658	1200
22/05/15 08:50	1128	540	480	1344
22/05/15 08:55	1344	540	470	1032
22/05/15 09:00	1440	576	636	1392
22/05/15 09:05	1404	720	514	1440
22/05/15 09:10	1080	720	573	1248
22/05/15 09:15	1176	756	508	1272
22/05/15 09:20	1128	900	596	1368
22/05/15 09:25	984	900	589	1128
22/05/15 09:30	960	900	547	1152
22/05/15 09:35	984	900	525	1128
22/05/15 09:40	864	900	538	1188
22/05/15 09:45	840	900	582	1104
22/05/15 09:50	1068	900	538	1260
22/05/15 09:55	1188	900	633	1224
22/05/15 10:00	864	900	568	1236
22/05/15 10:05	1032	900	679	948
22/05/15 10:10	828	900	484	1056
22/05/15 10:15	996	900	559	1152
22/05/15 10:20	840	900	333	1248
22/05/15 10:25	972	900	312	1164
22/05/15 10:30	672	900	312	1104
22/05/15 10:35	828	900	312	1188
22/05/15 10:40	720	900	312	1224
22/05/15 10:45	732	900	312	1116
22/05/15 10:50	732	900	374	1164
22/05/15 10:55	816	900	673	1308
22/05/15 11:00	696	900	662	1068
22/05/15 11:05	780	900	636	1140
22/05/15 11:10	612	900	663	948
22/05/15 11:15	804	900	619	1056
22/05/15 11:20	912	900	679	1380
22/05/15 11:25	744	900	781	1224
22/05/15 11:30	768	900	760	1320
22/05/15 11:35	828	900	846	1260
22/05/15 11:40	552	900	681	948
22/05/15 11:45	684	936	776	1200
22/05/15 11:50	756	1080	883	1404
22/05/15 11:55	852	1080	720	1236
22/05/15 12:00	648	1080	709	1104
22/05/15 12:05	960	1080	888	1128
22/05/15 12:10	828	1080	763	1188
22/05/15 12:15	816	1080	622	1080
22/05/15 12:20	888	1080	966	1104

Sistema de Controle de Tráfego Urbano OPTIMUS

5 MINUTOS	INTENSIDADE			
	PM 0403004	PM 0403006	PM 0404002	PM 0404004
22/05/15 12:25	768	1080	754	1236
22/05/15 12:30	660	1080	811	1104
22/05/15 12:35	888	1080	730	1128
22/05/15 12:40	768	1080	646	1380
22/05/15 12:45	792	1080	790	1104
22/05/15 12:50	960	1080	924	1044
22/05/15 12:55	1068	1080	834	1272
22/05/15 13:00	912	1080	757	1164
22/05/15 13:05	936	1080	721	1428
22/05/15 13:10	912	1080	739	1200
22/05/15 13:15	1032	1080	811	1332
22/05/15 13:20	852	1080	705	1284
22/05/15 13:25	864	1080	614	1344
22/05/15 13:30	1080	1080	694	1356
22/05/15 13:35	984	1080	812	1236
22/05/15 13:40	864	1080	670	1524
22/05/15 13:45	1068	1116	800	1392
22/05/15 13:50	732	1260	608	1152
22/05/15 13:55	792	1260	633	1116
22/05/15 14:00	744	1224	559	1608
22/05/15 14:05	1104	1080	608	1284
22/05/15 14:10	912	1080	588	1248
22/05/15 14:15	804	1080	628	1488
22/05/15 14:20	888	1080	636	1332
22/05/15 14:25	876	1080	616	1188
22/05/15 14:30	996	1080	607	1308
22/05/15 14:35	1068	1080	658	1644
22/05/15 14:40	732	1080	873	1176
22/05/15 14:45	768	1080	708	1236
22/05/15 14:50	1032	1080	711	1440
22/05/15 14:55	876	1080	584	1308
22/05/15 15:00	696	1080	808	1272
22/05/15 15:05	912	1080	453	1212
22/05/15 15:10	852	1080	590	1416
22/05/15 15:15	936	1080	628	1068
22/05/15 15:20	744	1080	896	1272
22/05/15 15:25	768	1080	593	1548
22/05/15 15:30	780	1080	654	1272
22/05/15 15:35	840	1080	574	1284
22/05/15 15:40	732	1080	902	1176
22/05/15 15:45	888	1080	660	1320
22/05/15 15:50	660	1080	819	1020
22/05/15 15:55	636	1080	536	1368
22/05/15 16:00	756	1080	753	1152
22/05/15 16:05	660	1080	623	1008
22/05/15 16:10	732	1080	997	1416
22/05/15 16:15	636	1080	721	1248
22/05/15 16:20	564	1080	928	984
22/05/15 16:25	840	1080	685	1140
22/05/15 16:30	492	1080	962	876
22/05/15 16:35	804	1080	612	1212
22/05/15 16:40	816	1080	769	984
22/05/15 16:45	696	1080	752	1080
22/05/15 16:50	672	1080	810	768
22/05/15 16:55	732	1080	883	1260
22/05/15 17:00	828	1080	960	1344
22/05/15 17:05	864	1080	697	1188
22/05/15 17:10	816	1080	973	600

Sistema de Controle de Tráfego Urbano OPTIMUS

5 MINUTOS	INTENSIDADE			
	PM 0403004	PM 0403006	PM 0404002	PM 0404004
22/05/15 17:15	828	1080	398	612
22/05/15 17:20	732	1080	312	396
22/05/15 17:25	720	1080	493	720
22/05/15 17:30	684	1080	288	672
22/05/15 17:35	936	1080	458	888
22/05/15 17:40	744	1080	312	588
22/05/15 17:45	696	1080	225	744
22/05/15 17:50	432	1080	387	732
22/05/15 17:55	624	1080	340	672
22/05/15 18:00	336	1116	285	828
22/05/15 18:05	696	1260	690	732
22/05/15 18:10	480	1260	378	924
22/05/15 18:15	1020	1260	519	936
22/05/15 18:20	768	1260	568	960
22/05/15 18:25	708	1260	486	948
22/05/15 18:30	504	1260	603	1020
22/05/15 18:35	708	1260	517	900
22/05/15 18:40	540	1260	663	1080
22/05/15 18:45	612	1260	663	948
22/05/15 18:50	672	1260	632	1248
22/05/15 18:55	624	1260	483	996
22/05/15 19:00	936	1224	696	852
22/05/15 19:05	792	1080	606	840
22/05/15 19:10	552	1080	680	948
22/05/15 19:15	732	1080	678	1056
22/05/15 19:20	600	1080	709	780
22/05/15 19:25	648	1080	603	1044
22/05/15 19:30	552	1080	554	960
22/05/15 19:35	528	1080	541	1068
22/05/15 19:40	552	1080	500	1080
22/05/15 19:45	504	1080	482	1068
22/05/15 19:50	600	1080	382	768
22/05/15 19:55	708	1080	291	1068
22/05/15 20:00	540	1080	424	1068
22/05/15 20:05	624	1080	403	972
22/05/15 20:10	756	1080	363	972
22/05/15 20:15	732	1080	345	1104
22/05/15 20:20	468	1080	554	960
22/05/15 20:25	552	1080	325	1176
22/05/15 20:30	684	1080	525	1116
22/05/15 20:35	456	1080	320	792
22/05/15 20:40	552	1080	378	1020
22/05/15 20:45	612	1080	474	804
22/05/15 20:50	468	1080	442	1560
22/05/15 20:55	528	1080	507	1500
22/05/15 21:00	384	1080	356	1560
22/05/15 21:05	420	1080	450	1560
22/05/15 21:10	408	1080	543	1056
22/05/15 21:15	600	1080	418	1104
22/05/15 21:20	468	1080	463	1284
22/05/15 21:25	480	1080	423	996
22/05/15 21:30	420	1080	387	804
22/05/15 21:35	312	1080	338	864
22/05/15 21:40	408	1080	325	876
22/05/15 21:45	444	1044	383	768
22/05/15 21:50	504	900	379	1020
22/05/15 21:55	492	900	362	1068
22/05/15 22:00	480	900	297	936

Sistema de Controle de Tráfego Urbano OPTIMUS

5 MINUTOS	INTENSIDADE			
	PM 0403004	PM 0403006	PM 0404002	PM 0404004
22/05/15 22:05	348	900	520	696
22/05/15 22:10	480	900	434	900
22/05/15 22:15	444	900	441	876
22/05/15 22:20	384	900	601	780
22/05/15 22:25	432	900	739	1104
22/05/15 22:30	336	900	572	816
22/05/15 22:35	432	900	384	816
22/05/15 22:40	300	900	513	864
22/05/15 22:45	288	900	527	828
22/05/15 22:50	300	900	336	816
22/05/15 22:55	324	900	228	744
22/05/15 23:00	240	900	312	900
22/05/15 23:05	288	900	312	816
22/05/15 23:10	480	900	235	636
22/05/15 23:15	312	864	349	708
22/05/15 23:20	216	720	282	756
22/05/15 23:25	264	720	193	708
22/05/15 23:30	336	720	409	528
22/05/15 23:35	216	720	184	648
22/05/15 23:40	180	720	280	528
22/05/15 23:45	156	720	170	540
22/05/15 23:50	312	720	157	612
22/05/15 23:55	216	720	165	540
23/05/15 00:00	228	684	182	600
23/05/15 00:05	192	540	284	864
23/05/15 00:10	168	540	181	600
23/05/15 00:15	216	540	177	564
23/05/15 00:20	192	540	141	492
23/05/15 00:25	204	540	169	480
23/05/15 00:30	192	540	151	492
23/05/15 00:35	156	540	126	396
23/05/15 00:40	240	540	79	480
23/05/15 00:45	228	540	176	468
23/05/15 00:50	144	540	150	492
23/05/15 00:55	144	540	164	240
23/05/15 01:00	240	540	130	444
23/05/15 01:05	120	540	141	432
23/05/15 01:10	228	540	90	456
23/05/15 01:15	240	504	166	348
23/05/15 01:20	156	360	178	372
23/05/15 01:25	96	360	166	216
23/05/15 01:30	156	360	124	348
23/05/15 01:35	180	360	130	276
23/05/15 01:40	156	360	70	276
23/05/15 01:45	180	360	122	348
23/05/15 01:50	72	360	115	288
23/05/15 01:55	192	360	82	204
23/05/15 02:00	132	360	74	276
23/05/15 02:05	120	360	55	300
23/05/15 02:10	84	360	74	300
23/05/15 02:15	216	360	34	264
23/05/15 02:20	180	360	67	240
23/05/15 02:25	60	360	91	324
23/05/15 02:30	48	360	36	348
23/05/15 02:35	132	360	74	288
23/05/15 02:40	108	360	87	240
23/05/15 02:45	48	360	99	264
23/05/15 02:50	72	360	98	264

Sistema de Controle de Tráfego Urbano OPTIMUS

5 MINUTOS	INTENSIDADE			
	PM 0403004	PM 0403006	PM 0404002	PM 0404004
23/05/15 02:55	60	360	79	156
23/05/15 03:00	72	324	55	144
23/05/15 03:05	48	180	67	276
23/05/15 03:10	96	180	24	264
23/05/15 03:15	96	180	87	204
23/05/15 03:20	108	180	102	204
23/05/15 03:25	72	180	79	168
23/05/15 03:30	48	180	63	192
23/05/15 03:35	24	180	39	192
23/05/15 03:40	36	180	106	144
23/05/15 03:45	72	180	63	228
23/05/15 03:50	72	180	55	240
23/05/15 03:55	60	180	27	144
23/05/15 04:00	96	180	27	324
23/05/15 04:05	48	180	34	228
23/05/15 04:10	36	180	31	228
23/05/15 04:15	60	180	87	240
23/05/15 04:20	108	180	150	216
23/05/15 04:25	60	180	122	264
23/05/15 04:30	72	180	72	192
23/05/15 04:35	84	180	54	144
23/05/15 04:40	108	180	75	192
23/05/15 04:45	132	180	43	324
23/05/15 04:50	96	180	36	276
23/05/15 04:55	96	180	12	168
23/05/15 05:00	108	180	79	132
23/05/15 05:05	60	180	72	276
23/05/15 05:10	24	180	86	228
23/05/15 05:15	72	180	19	228
23/05/15 05:20	96	180	63	312
23/05/15 05:25	120	180	60	288
23/05/15 05:30	96	180	31	288
23/05/15 05:35	108	180	129	216
23/05/15 05:40	144	180	82	372
23/05/15 05:45	108	180	94	252
23/05/15 05:50	108	180	48	300
23/05/15 05:55	84	180	103	204
23/05/15 06:00	60	180	115	312
23/05/15 06:05	144	180	111	348
23/05/15 06:10	156	180	85	264
23/05/15 06:15	108	180	112	432
23/05/15 06:20	96	180	34	312
23/05/15 06:25	192	180	42	540
23/05/15 06:30	216	180	126	396
23/05/15 06:35	240	180	117	456
23/05/15 06:40	252	180	129	612
23/05/15 06:45	456	180	169	732
23/05/15 06:50	336	180	201	660
23/05/15 06:55	576	180	220	624
23/05/15 07:00	360	180	198	564
23/05/15 07:05	528	180	208	732
23/05/15 07:10	384	180	225	600
23/05/15 07:15	420	180	213	600
23/05/15 07:20	372	180	205	480
23/05/15 07:25	528	180	224	828
23/05/15 07:30	648	180	268	624
23/05/15 07:35	552	180	141	864
23/05/15 07:40	528	180	273	756

Sistema de Controle de Tráfego Urbano OPTIMUS

5 MINUTOS	INTENSIDADE			
	PM 040300 4	PM 040300 6	PM 040400 2	PM 040400 4
23/05/15 07:45	552	180	207	984
23/05/15 07:50	564	180	355	912
23/05/15 07:55	864	180	224	888
23/05/15 08:00	672	180	399	936
23/05/15 08:05	708	180	302	1020
23/05/15 08:10	660	180	342	924
23/05/15 08:15	612	180	346	888
23/05/15 08:20	708	180	278	852
23/05/15 08:25	672	180	274	996
23/05/15 08:30	624	180	295	996
23/05/15 08:35	792	180	274	1164
23/05/15 08:40	720	180	286	1164
23/05/15 08:45	744	180	237	972
23/05/15 08:50	852	180	314	912
23/05/15 08:55	864	180	375	1188
23/05/15 09:00	828	180	355	1044
23/05/15 09:05	756	180	364	1296
23/05/15 09:10	792	180	346	1104
23/05/15 09:15	756	216	478	1188
23/05/15 09:20	816	360	370	1164
23/05/15 09:25	624	360	396	1164
23/05/15 09:30	660	360	422	1152
23/05/15 09:35	852	360	422	1332
23/05/15 09:40	780	360	374	1128
23/05/15 09:45	744	396	342	1152
23/05/15 09:50	840	540	291	1056
23/05/15 09:55	708	540	484	1260
23/05/15 10:00	780	540	403	1008
23/05/15 10:05	780	540	444	1320
23/05/15 10:10	828	540	427	1896
23/05/15 10:15	888	540	334	1080
23/05/15 10:20	972	540	370	1116
23/05/15 10:25	816	540	278	1380
23/05/15 10:30	948	540	367	1092
23/05/15 10:35	756	540	423	1296
23/05/15 10:40	960	540	484	1284
23/05/15 10:45	732	540	373	1236
23/05/15 10:50	972	540	454	972
23/05/15 10:55	972	540	505	1356
23/05/15 11:00	936	576	450	1044
23/05/15 11:05	852	720	446	1488
23/05/15 11:10	720	720	481	1116
23/05/15 11:15	756	720	547	1416
23/05/15 11:20	792	720	582	1116
23/05/15 11:25	660	720	487	1296
23/05/15 11:30	684	720	412	1224
23/05/15 11:35	624	720	440	1248
23/05/15 11:40	744	720	408	1152
23/05/15 11:45	636	720	412	1152
23/05/15 11:50	876	720	472	1380
23/05/15 11:55	840	720	474	1200
23/05/15 12:00	792	756	547	1188
23/05/15 12:05	816	900	435	1308
23/05/15 12:10	768	900	642	1224
23/05/15 12:15	1104	900	614	1488
23/05/15 12:20	756	900	444	1392
23/05/15 12:25	756	900	634	1296
23/05/15 12:30	744	900	523	1104

Sistema de Controle de Tráfego Urbano OPTIMUS

5 MINUTOS	INTENSIDADE			
	PM 040300 4	PM 040300 6	PM 040400 2	PM 040400 4
23/05/15 12:35	708	900	423	1128
23/05/15 12:40	612	900	592	1116
23/05/15 12:45	804	900	346	1356
23/05/15 12:50	756	900	448	900
23/05/15 12:55	792	900	285	1356
23/05/15 13:00	828	936	493	888
23/05/15 13:05	756	1080	380	1200
23/05/15 13:10	744	1080	543	936
23/05/15 13:15	804	1080	632	1212
23/05/15 13:20	600	1080	542	960
23/05/15 13:25	636	1080	422	1440
23/05/15 13:30	480	1080	375	1068
23/05/15 13:35	780	1080	294	1236
23/05/15 13:40	636	1080	439	948
23/05/15 13:45	552	1080	298	1080
23/05/15 13:50	588	1080	439	960
23/05/15 13:55	540	1080	326	1164
23/05/15 14:00	816	1080	409	996
23/05/15 14:05	564	1080	302	1224
23/05/15 14:10	696	1080	424	948
23/05/15 14:15	648	1044	406	1332
23/05/15 14:20	516	900	429	1116
23/05/15 14:25	684	900	330	1212
23/05/15 14:30	624	900	433	1068
23/05/15 14:35	528	900	380	1008
23/05/15 14:40	624	900	350	852
23/05/15 14:45	672	900	271	1248
23/05/15 14:50	456	900	331	948
23/05/15 14:55	600	900	304	984
23/05/15 15:00	408	900	548	816
23/05/15 15:05	612	900	386	1320
23/05/15 15:10	456	900	369	936
23/05/15 15:15	504	900	320	1068
23/05/15 15:20	420	900	412	804
23/05/15 15:25	468	900	328	1128
23/05/15 15:30	504	900	219	900
23/05/15 15:35	552	900	346	1272
23/05/15 15:40	636	900	444	1080
23/05/15 15:45	480	900	464	888
23/05/15 15:50	324	900	441	1008
23/05/15 15:55	540	900	267	876
23/05/15 16:00	420	900	381	972
23/05/15 16:05	516	900	356	912
23/05/15 16:10	660	900	342	804
23/05/15 16:15	420	900	415	1032
23/05/15 16:20	408	900	351	912
23/05/15 16:25	660	900	290	900
23/05/15 16:30	516	900	304	948
23/05/15 16:35	480	900	343	1116
23/05/15 16:40	492	900	494	1368
23/05/15 16:45	576	900	360	1248
23/05/15 16:50	444	900	374	1104
23/05/15 16:55	660	900	334	1104
23/05/15 17:00	600	900	498	1032
23/05/15 17:05	468	900	400	1140
23/05/15 17:10	552	900	448	1152
23/05/15 17:15	684	900	608	1116
23/05/15 17:20	648	900	585	1092

Sistema de Controle de Tráfego Urbano OPTIMUS

5 MINUTOS	INTENSIDADE			
	PM 0403004	PM 0403006	PM 0404002	PM 0404004
23/05/15 17:25	528	900	360	888
23/05/15 17:30	504	900	454	804
23/05/15 17:35	588	900	348	876
23/05/15 17:40	480	900	411	1128
23/05/15 17:45	600	900	333	948
23/05/15 17:50	480	900	434	828
23/05/15 17:55	516	900	177	1020
23/05/15 18:00	648	900	356	948
23/05/15 18:05	612	900	412	900
23/05/15 18:10	684	900	224	1020
23/05/15 18:15	804	900	314	984
23/05/15 18:20	528	900	348	1008
23/05/15 18:25	516	900	307	1056
23/05/15 18:30	528	900	310	1056
23/05/15 18:35	684	900	375	1020
23/05/15 18:40	708	900	267	1260
23/05/15 18:45	804	900	327	1092
23/05/15 18:50	648	900	207	1092
23/05/15 18:55	624	900	308	1020
23/05/15 19:00	576	900	378	924
23/05/15 19:05	504	900	393	768
23/05/15 19:10	648	900	410	1140
23/05/15 19:15	576	900	374	1404
23/05/15 19:20	636	900	340	1128
23/05/15 19:25	744	900	367	1188
23/05/15 19:30	552	900	320	1056
23/05/15 19:35	528	900	287	1080
23/05/15 19:40	624	900	370	1116
23/05/15 19:45	516	900	238	1044
23/05/15 19:50	684	900	268	1104
23/05/15 19:55	696	900	370	1008
23/05/15 20:00	516	900	218	1056
23/05/15 20:05	408	900	214	924
23/05/15 20:10	660	900	259	1176
23/05/15 20:15	576	900	255	1200
23/05/15 20:20	528	900	260	840
23/05/15 20:25	444	900	314	876
23/05/15 20:30	480	900	235	744
23/05/15 20:35	444	900	243	1176
23/05/15 20:40	552	900	362	972
23/05/15 20:45	492	900	253	840
23/05/15 20:50	492	900	273	924
23/05/15 20:55	552	900	454	900
23/05/15 21:00	300	900	502	912
23/05/15 21:05	492	900	343	732
23/05/15 21:10	576	900	285	840
23/05/15 21:15	408	900	225	816
23/05/15 21:20	396	900	232	708
23/05/15 21:25	540	900	241	852
23/05/15 21:30	360	900	310	876
23/05/15 21:35	516	900	208	1068
23/05/15 21:40	360	900	322	732
23/05/15 21:45	360	900	265	876
23/05/15 21:50	300	900	176	768
23/05/15 21:55	420	900	199	744
23/05/15 22:00	336	900	181	900
23/05/15 22:05	528	900	192	852
23/05/15 22:10	456	900	339	756

Sistema de Controle de Tráfego Urbano OPTIMUS

5 MINUTOS	INTENSIDADE			
	PM 0403004	PM 0403006	PM 0404002	PM 0404004
23/05/15 22:15	252	900	244	948
23/05/15 22:20	300	900	232	888
23/05/15 22:25	480	900	152	996
23/05/15 22:30	312	900	314	852
23/05/15 22:35	420	900	284	1008
23/05/15 22:40	336	900	206	780
23/05/15 22:45	252	900	301	816
23/05/15 22:50	420	900	169	756
23/05/15 22:55	240	900	210	852
23/05/15 23:00	300	864	277	972
23/05/15 23:05	396	720	229	732
23/05/15 23:10	276	720	236	624
23/05/15 23:15	384	720	241	480
23/05/15 23:20	312	720	241	732
23/05/15 23:25	276	720	201	636
23/05/15 23:30	300	720	102	792
23/05/15 23:35	228	720	147	552
23/05/15 23:40	336	720	118	672
23/05/15 23:45	276	720	208	564
23/05/15 23:50	252	720	151	660
23/05/15 23:55	228	720	158	732
24/05/15 00:00	144	720	114	672
24/05/15 00:05	216	720	139	744
24/05/15 00:10	264	720	237	624
24/05/15 00:15	156	684	153	480
24/05/15 00:20	252	540	154	528
24/05/15 00:25	252	540	134	612
24/05/15 00:30	336	540	166	540
24/05/15 00:35	276	540	90	792
24/05/15 00:40	204	540	81	648
24/05/15 00:45	180	540	222	516
24/05/15 00:50	204	540	151	516
24/05/15 00:55	144	540	150	480
24/05/15 01:00	180	540	193	504
24/05/15 01:05	168	540	208	276
24/05/15 01:10	96	540	70	516
24/05/15 01:15	156	540	150	576
24/05/15 01:20	216	540	115	372
24/05/15 01:25	156	540	110	240
24/05/15 01:30	168	540	121	456
24/05/15 01:35	156	540	110	396
24/05/15 01:40	180	540	90	420
24/05/15 01:45	120	504	186	492
24/05/15 01:50	108	360	123	384
24/05/15 01:55	96	360	96	240
24/05/15 02:00	288	360	99	396
24/05/15 02:05	108	360	142	552
24/05/15 02:10	120	360	110	312
24/05/15 02:15	168	360	122	396
24/05/15 02:20	180	360	146	396
24/05/15 02:25	120	360	118	384
24/05/15 02:30	144	360	132	252
24/05/15 02:35	144	360	170	384
24/05/15 02:40	60	360	51	228
24/05/15 02:45	108	360	111	396
24/05/15 02:50	84	360	133	240
24/05/15 02:55	180	360	51	288
24/05/15 03:00	84	324	48	300

Sistema de Controle de Tráfego Urbano OPTIMUS

5 MINUTOS	INTENSIDADE			
	PM 040300 4	PM 040300 6	PM 040400 2	PM 040400 4
24/05/15 03:05	120	180	48	144
24/05/15 03:10	72	180	79	180
24/05/15 03:15	120	180	79	288
24/05/15 03:20	96	180	122	228
24/05/15 03:25	132	180	55	216
24/05/15 03:30	84	180	118	324
24/05/15 03:35	108	180	39	144
24/05/15 03:40	36	180	96	180
24/05/15 03:45	84	180	46	204
24/05/15 03:50	120	180	48	300
24/05/15 03:55	72	180	75	204
24/05/15 04:00	84	180	117	192
24/05/15 04:05	24	180	60	168
24/05/15 04:10	36	180	43	288
24/05/15 04:15	60	180	103	144
24/05/15 04:20	84	180	74	192
24/05/15 04:25	60	180	51	144
24/05/15 04:30	24	180	70	180
24/05/15 04:35	120	180	58	156
24/05/15 04:40	60	180	48	252
24/05/15 04:45	60	180	51	144
24/05/15 04:50	36	180	72	144
24/05/15 04:55	60	180	55	192
24/05/15 05:00	120	180	67	216
24/05/15 05:05	72	180	74	264
24/05/15 05:10	84	180	19	312
24/05/15 05:15	96	180	36	228
24/05/15 05:20	60	180	51	312
24/05/15 05:25	60	180	36	192
24/05/15 05:30	60	144	63	168
24/05/15 05:35	96	0	31	216
24/05/15 05:40	48	0	46	264
24/05/15 05:45	84	0	67	216
24/05/15 05:50	108	0	91	300
24/05/15 05:55	0	0	62	72
24/05/15 06:00	132	0	123	228
24/05/15 06:05	60	0	178	252
24/05/15 06:10	96	0	94	144
24/05/15 06:15	96	0	84	360
24/05/15 06:20	60	0	46	264
24/05/15 06:25	96	0	55	240
24/05/15 06:30	132	0	48	240
24/05/15 06:35	144	0	122	276
24/05/15 06:40	192	0	94	252
24/05/15 06:45	264	0	134	408
24/05/15 06:50	216	0	67	384
24/05/15 06:55	300	0	146	336
24/05/15 07:00	144	0	135	360
24/05/15 07:05	216	0	133	396
24/05/15 07:10	264	0	246	384
24/05/15 07:15	276	0	166	516
24/05/15 07:20	144	0	217	288
24/05/15 07:25	264	0	142	384
24/05/15 07:30	300	0	115	444
24/05/15 07:35	228	0	182	384
24/05/15 07:40	144	0	198	372
24/05/15 07:45	372	0	194	516
24/05/15 07:50	168	0	193	528

Sistema de Controle de Tráfego Urbano OPTIMUS

5 MINUTOS	INTENSIDADE			
	PM 0403004	PM 0403006	PM 0404002	PM 0404004
24/05/15 07:55	300	0	217	324
24/05/15 08:00	240	0	222	444
24/05/15 08:05	288	0	152	420
24/05/15 08:10	192	0	181	300
24/05/15 08:15	300	36	270	528
24/05/15 08:20	216	180	182	540
24/05/15 08:25	216	180	165	420
24/05/15 08:30	348	180	170	336
24/05/15 08:35	348	180	229	396
24/05/15 08:40	396	180	134	600
24/05/15 08:45	300	180	228	444
24/05/15 08:50	528	180	301	480
24/05/15 08:55	516	180	354	660
24/05/15 09:00	528	180	189	492
24/05/15 09:05	492	180	234	624
24/05/15 09:10	288	180	157	624
24/05/15 09:15	444	216	187	540
24/05/15 09:20	168	360	166	636
24/05/15 09:25	264	360	273	612
24/05/15 09:30	300	360	182	516
24/05/15 09:35	348	360	190	612
24/05/15 09:40	384	360	292	540
24/05/15 09:45	504	360	340	636
24/05/15 09:50	408	360	258	756
24/05/15 09:55	408	360	186	708
24/05/15 10:00	312	360	224	744
24/05/15 10:05	312	360	258	588
24/05/15 10:10	408	360	229	624
24/05/15 10:15	324	360	248	612
24/05/15 10:20	336	360	278	612
24/05/15 10:25	432	360	262	672
24/05/15 10:30	360	360	208	672
24/05/15 10:35	492	360	253	696
24/05/15 10:40	360	360	272	720
24/05/15 10:45	396	360	225	840
24/05/15 10:50	336	360	217	636
24/05/15 10:55	468	360	328	504
24/05/15 11:00	384	360	181	684
24/05/15 11:05	504	360	212	852
24/05/15 11:10	396	360	307	660
24/05/15 11:15	576	360	217	780
24/05/15 11:20	408	360	220	900
24/05/15 11:25	720	360	378	996
24/05/15 11:30	552	360	318	828
24/05/15 11:35	492	360	256	816
24/05/15 11:40	468	360	376	636
24/05/15 11:45	492	360	272	732
24/05/15 11:50	552	360	427	864
24/05/15 11:55	540	360	388	1152
24/05/15 12:00	420	396	354	996
24/05/15 12:05	588	540	247	996
24/05/15 12:10	540	540	391	816
24/05/15 12:15	588	540	405	780
24/05/15 12:20	600	540	403	972
24/05/15 12:25	612	540	496	948
24/05/15 12:30	444	576	399	912
24/05/15 12:35	672	720	480	1044
24/05/15 12:40	588	720	308	1200

Sistema de Controle de Tráfego Urbano OPTIMUS

5 MINUTOS	INTENSIDADE			
	PM 040300 4	PM 040300 6	PM 040400 2	PM 040400 4
24/05/15 12:45	768	720	300	1068
24/05/15 12:50	444	720	409	1152
24/05/15 12:55	552	720	380	972
24/05/15 13:00	600	720	328	780
24/05/15 13:05	516	720	442	972
24/05/15 13:10	696	720	356	864
24/05/15 13:15	528	720	459	696
24/05/15 13:20	612	720	258	816
24/05/15 13:25	588	720	348	852
24/05/15 13:30	492	720	316	744
24/05/15 13:35	432	720	344	720
24/05/15 13:40	744	720	244	936
24/05/15 13:45	588	720	302	708
24/05/15 13:50	384	720	319	732
24/05/15 13:55	408	720	356	744
24/05/15 14:00	420	720	294	648
24/05/15 14:05	492	720	475	612
24/05/15 14:10	372	720	217	720
24/05/15 14:15	552	756	352	636
24/05/15 14:20	696	900	217	792
24/05/15 14:25	660	900	340	780
24/05/15 14:30	384	864	200	732
24/05/15 14:35	444	720	261	600
24/05/15 14:40	324	720	253	684
24/05/15 14:45	372	720	338	720
24/05/15 14:50	516	720	230	720
24/05/15 14:55	480	720	266	672
24/05/15 15:00	384	720	243	660
24/05/15 15:05	432	720	348	744
24/05/15 15:10	348	720	284	684
24/05/15 15:15	288	720	316	660
24/05/15 15:20	480	720	337	636
24/05/15 15:25	552	720	361	840
24/05/15 15:30	468	720	364	804
24/05/15 15:35	432	720	303	564
24/05/15 15:40	384	720	336	696
24/05/15 15:45	468	720	421	828
24/05/15 15:50	384	720	193	864
24/05/15 15:55	444	720	289	876
24/05/15 16:00	372	720	280	900
24/05/15 16:05	456	720	284	876
24/05/15 16:10	600	720	217	1020
24/05/15 16:15	468	720	268	756
24/05/15 16:20	492	720	325	876
24/05/15 16:25	432	720	210	840
24/05/15 16:30	384	720	205	912
24/05/15 16:35	528	720	265	876
24/05/15 16:40	516	720	292	756
24/05/15 16:45	636	720	313	912
24/05/15 16:50	444	720	285	876
24/05/15 16:55	540	720	376	852
24/05/15 17:00	396	720	242	1032
24/05/15 17:05	528	720	308	804
24/05/15 17:10	432	720	364	696
24/05/15 17:15	588	720	336	816
24/05/15 17:20	432	720	412	792
24/05/15 17:25	516	720	297	876
24/05/15 17:30	408	720	284	780

Sistema de Controle de Tráfego Urbano OPTIMUS

5 MINUTOS	INTENSIDADE			
	PM 040300 4	PM 040300 6	PM 040400 2	PM 040400 4
24/05/15 17:35	468	720	310	840
24/05/15 17:40	408	720	235	984
24/05/15 17:45	420	720	212	1020
24/05/15 17:50	516	720	219	852
24/05/15 17:55	408	720	248	912
24/05/15 18:00	420	720	343	720
24/05/15 18:05	516	720	265	972
24/05/15 18:10	552	720	303	936
24/05/15 18:15	480	720	252	1008
24/05/15 18:20	552	720	201	900
24/05/15 18:25	696	720	205	1068
24/05/15 18:30	600	720	287	840
24/05/15 18:35	648	720	307	1020
24/05/15 18:40	432	720	339	984
24/05/15 18:45	576	720	350	996
24/05/15 18:50	576	720	234	792
24/05/15 18:55	768	720	295	900
24/05/15 19:00	504	756	205	888
24/05/15 19:05	588	900	266	744
24/05/15 19:10	564	900	362	756
24/05/15 19:15	576	900	285	900
24/05/15 19:20	396	900	375	744
24/05/15 19:25	612	900	248	912
24/05/15 19:30	408	900	304	888
24/05/15 19:35	348	900	349	852
24/05/15 19:40	348	900	224	672
24/05/15 19:45	588	900	217	744
24/05/15 19:50	528	900	236	960
24/05/15 19:55	600	900	316	840
24/05/15 20:00	420	900	243	852
24/05/15 20:05	348	900	236	768
24/05/15 20:10	336	900	153	684
24/05/15 20:15	276	900	205	696
24/05/15 20:20	504	900	129	756
24/05/15 20:25	324	900	219	708
24/05/15 20:30	444	900	165	636
24/05/15 20:35	324	900	130	732
24/05/15 20:40	408	900	166	624
24/05/15 20:45	360	900	172	768
24/05/15 20:50	348	900	217	828
24/05/15 20:55	276	900	165	804
24/05/15 21:00	480	900	188	588
24/05/15 21:05	312	900	226	696
24/05/15 21:10	180	900	116	732
24/05/15 21:15	372	900	280	780
24/05/15 21:20	300	900	284	960
24/05/15 21:25	312	900	277	780
24/05/15 21:30	444	900	186	564
24/05/15 21:35	192	900	198	708
24/05/15 21:40	408	900	124	516
24/05/15 21:45	276	864	106	540
24/05/15 21:50	252	720	146	768
24/05/15 21:55	240	720	141	624
24/05/15 22:00	168	684	94	600
24/05/15 22:05	204	540	130	516
24/05/15 22:10	240	540	105	516
24/05/15 22:15	228	540	114	384
24/05/15 22:20	288	540	126	420

Sistema de Controle de Tráfego Urbano OPTIMUS

5 MINUTOS	INTENSIDADE			
	PM 0403004	PM 0403006	PM 0404002	PM 0404004
24/05/15 22:25	276	540	170	468
24/05/15 22:30	144	540	145	480
24/05/15 22:35	192	540	114	384
24/05/15 22:40	204	540	100	540
24/05/15 22:45	240	540	98	492
24/05/15 22:50	216	540	31	408
24/05/15 22:55	192	540	154	372
24/05/15 23:00	168	540	141	360
24/05/15 23:05	204	540	98	372
24/05/15 23:10	204	540	118	396
24/05/15 23:15	48	504	114	408
24/05/15 23:20	192	360	91	324
24/05/15 23:25	96	360	146	348
24/05/15 23:30	120	360	79	336
24/05/15 23:35	120	360	50	300
24/05/15 23:40	60	360	12	324
24/05/15 23:45	60	360	22	240
24/05/15 23:50	108	360	39	288
24/05/15 23:55	168	360	79	300
25/05/15 00:00	60	360	39	180
25/05/15 00:05	120	360	27	288
25/05/15 00:10	72	360	110	204
25/05/15 00:15	36	324	50	264
25/05/15 00:20	60	180	51	252
25/05/15 00:25	36	180	67	132
25/05/15 00:30	96	180	75	96
25/05/15 00:35	24	180	43	108
25/05/15 00:40	120	180	27	252
25/05/15 00:45	36	180	51	156
25/05/15 00:50	60	180	63	96
25/05/15 00:55	0	180	12	156
25/05/15 01:00	36	180	51	108
25/05/15 01:05	12	180	24	108
25/05/15 01:10	12	180	7	36
25/05/15 01:15	24	144	15	132
25/05/15 01:20	12	0	0	132
25/05/15 01:25	60	0	7	96
25/05/15 01:30	84	0	67	144
25/05/15 01:35	24	0	0	108
25/05/15 01:40	0	0	75	48
25/05/15 01:45	0	0	51	60
25/05/15 01:50	0	0	0	84
25/05/15 01:55	36	0	27	120
25/05/15 02:00	0	0	12	60
25/05/15 02:05	36	0	24	60
25/05/15 02:10	12	0	7	36
25/05/15 02:15	36	0	3	36
25/05/15 02:20	36	0	24	84
25/05/15 02:25	24	0	12	108
25/05/15 02:30	12	0	36	36
25/05/15 02:35	12	0	12	36
25/05/15 02:40	12	0	27	60
25/05/15 02:45	12	0	39	72
25/05/15 02:50	24	0	15	72
25/05/15 02:55	0	0	3	36
25/05/15 03:00	24	0	0	36
25/05/15 03:05	48	0	7	60
25/05/15 03:10	36	0	3	48

Sistema de Controle de Tráfego Urbano OPTIMUS

5 MINUTOS	INTENSIDADE			
	PM 040300 4	PM 040300 6	PM 040400 2	PM 040400 4
25/05/15 03:15	0	0	27	72
25/05/15 03:20	24	0	15	36
25/05/15 03:25	0	0	24	36
25/05/15 03:30	0	0	0	36
25/05/15 03:35	48	0	0	12
25/05/15 03:40	36	0	12	96
25/05/15 03:45	12	0	48	72
25/05/15 03:50	24	0	12	36
25/05/15 03:55	24	0	12	60
25/05/15 04:00	24	0	3	108
25/05/15 04:05	12	0	3	24
25/05/15 04:10	24	0	0	120
25/05/15 04:15	12	0	12	108
25/05/15 04:20	12	0	12	24
25/05/15 04:25	12	0	0	48
25/05/15 04:30	12	0	15	60
25/05/15 04:35	0	0	24	96
25/05/15 04:40	36	0	12	132
25/05/15 04:45	24	0	27	180
25/05/15 04:50	24	0	24	96
25/05/15 04:55	24	0	24	60
25/05/15 05:00	36	0	27	144
25/05/15 05:05	48	0	39	120
25/05/15 05:10	72	0	27	228
25/05/15 05:15	36	0	0	96
25/05/15 05:20	96	0	19	180
25/05/15 05:25	132	0	115	156
25/05/15 05:30	96	0	103	336
25/05/15 05:35	132	0	96	324
25/05/15 05:40	168	0	46	300
25/05/15 05:45	156	0	86	384
25/05/15 05:50	204	0	118	288
25/05/15 05:55	216	0	91	468
25/05/15 06:00	228	0	111	348
25/05/15 06:05	252	0	138	528
25/05/15 06:10	432	0	150	480
25/05/15 06:15	408	0	102	576
25/05/15 06:20	396	0	187	420
25/05/15 06:25	588	0	201	576
25/05/15 06:30	948	0	160	948
25/05/15 06:35	1032	0	241	1152
25/05/15 06:40	1272	0	172	1224
25/05/15 06:45	1704	0	141	1308
25/05/15 06:50	1896	0	319	1476
25/05/15 06:55	1668	0	273	1104
25/05/15 07:00	2052	0	680	1584
25/05/15 07:05	1860	0	493	1512
25/05/15 07:10	2100	0	634	1584
25/05/15 07:15	1836	0	700	1356
25/05/15 07:20	1896	0	691	1752
25/05/15 07:25	1740	0	595	1500
25/05/15 07:30	2028	0	524	1800
25/05/15 07:35	1944	0	532	1500
25/05/15 07:40	2136	0	642	1644
25/05/15 07:45	2016	0	541	1536
25/05/15 07:50	1944	0	468	1680
25/05/15 07:55	1740	0	573	1320
25/05/15 08:00	1584	0	633	1476

Sistema de Controle de Tráfego Urbano OPTIMUS

5 MINUTOS	INTENSIDADE			
	PM 040300 4	PM 040300 6	PM 040400 2	PM 040400 4
25/05/15 08:05	1392	0	637	1452
25/05/15 08:10	1536	0	470	1404
25/05/15 08:15	1344	36	527	1296
25/05/15 08:20	1356	180	391	1368
25/05/15 08:25	1176	180	510	1296
25/05/15 08:30	1260	180	590	1332
25/05/15 08:35	1416	180	592	1248
25/05/15 08:40	1212	180	486	1332
25/05/15 08:45	1548	216	631	1452
25/05/15 08:50	1440	360	471	1200
25/05/15 08:55	1524	360	462	1584
25/05/15 09:00	1368	396	562	960
25/05/15 09:05	1320	540	588	1560
25/05/15 09:10	1140	540	540	1008
25/05/15 09:15	1008	576	489	1536
25/05/15 09:20	1032	720	538	1056
25/05/15 09:25	1140	720	547	972
25/05/15 09:30	924	720	520	1152
25/05/15 09:35	1008	720	640	984
25/05/15 09:40	780	720	640	1188
25/05/15 09:45	924	720	658	1200
25/05/15 09:50	924	720	588	1200
25/05/15 09:55	1068	720	724	1632
25/05/15 10:00	804	720	580	1428
25/05/15 10:05	1068	720	476	1032
25/05/15 10:10	876	720	637	1080
25/05/15 10:15	720	720	603	1284
25/05/15 10:20	588	720	496	1296
25/05/15 10:25	708	720	706	1104
25/05/15 10:30	624	720	656	1320
25/05/15 10:35	828	720	597	1248
25/05/15 10:40	684	720	583	936
25/05/15 10:45	636	720	558	1272
25/05/15 10:50	912	720	613	1272
25/05/15 10:55	720	720	495	936
25/05/15 11:00	612	720	585	936
25/05/15 11:05	516	720	681	984
25/05/15 11:10	744	720	721	1020
25/05/15 11:15	564	720	702	1284
25/05/15 11:20	672	720	674	924
25/05/15 11:25	696	720	709	924
25/05/15 11:30	708	720	516	1092
25/05/15 11:35	612	720	744	996
25/05/15 11:40	732	720	698	1116
25/05/15 11:45	696	720	651	1152
25/05/15 11:50	756	720	836	1212
25/05/15 11:55	504	720	884	1044
25/05/15 12:00	504	720	678	900
25/05/15 12:05	708	720	699	984
25/05/15 12:10	696	720	642	1284
25/05/15 12:15	864	720	756	1152
25/05/15 12:20	588	720	829	1068
25/05/15 12:25	636	720	661	876
25/05/15 12:30	660	756	555	1260
25/05/15 12:35	720	900	625	1068
25/05/15 12:40	912	900	554	1140
25/05/15 12:45	936	900	760	1188
25/05/15 12:50	1008	900	747	1236

Sistema de Controle de Tráfego Urbano OPTIMUS

5 MINUTOS	INTENSIDADE			
	PM 040300 4	PM 040300 6	PM 040400 2	PM 040400 4
25/05/15 12:55	1452	900	843	1416
25/05/15 13:00	972	900	662	1224
25/05/15 13:05	960	900	831	1488
25/05/15 13:10	924	900	688	1260
25/05/15 13:15	1080	900	732	1224
25/05/15 13:20	972	900	568	1248
25/05/15 13:25	1140	900	598	1020
25/05/15 13:30	1044	900	590	1320
25/05/15 13:35	1008	900	546	1176
25/05/15 13:40	768	900	612	1404
25/05/15 13:45	1008	900	752	1200
25/05/15 13:50	1056	900	573	1188
25/05/15 13:55	948	900	697	1152
25/05/15 14:00	1008	900	555	1092
25/05/15 14:05	1032	900	571	1392
25/05/15 14:10	804	900	475	1260
25/05/15 14:15	912	900	636	1524
25/05/15 14:20	828	900	522	1152
25/05/15 14:25	792	900	574	1428
25/05/15 14:30	828	900	434	984
25/05/15 14:35	888	900	765	1260
25/05/15 14:40	1032	900	856	1284
25/05/15 14:45	864	900	399	1320
25/05/15 14:50	660	900	639	1116
25/05/15 14:55	648	900	537	1368
25/05/15 15:00	804	900	660	960
25/05/15 15:05	756	900	610	1188
25/05/15 15:10	708	900	835	1224
25/05/15 15:15	876	900	693	972
25/05/15 15:20	660	900	808	1308
25/05/15 15:25	732	900	651	1260
25/05/15 15:30	696	900	632	1068
25/05/15 15:35	828	900	585	1092
25/05/15 15:40	756	900	668	1272
25/05/15 15:45	852	900	462	1068
25/05/15 15:50	732	900	738	1188
25/05/15 15:55	996	900	547	1068
25/05/15 16:00	672	936	898	1164
25/05/15 16:05	852	1080	630	1320
25/05/15 16:10	720	1080	813	1152
25/05/15 16:15	816	1080	681	1104
25/05/15 16:20	612	1080	928	1440
25/05/15 16:25	708	1080	603	1272
25/05/15 16:30	576	1080	945	1080
25/05/15 16:35	924	1080	638	1344
25/05/15 16:40	780	1080	782	1380
25/05/15 16:45	732	1080	852	1236
25/05/15 16:50	972	1080	801	1056
25/05/15 16:55	636	1080	734	900
25/05/15 17:00	624	1080	1092	1212
25/05/15 17:05	648	1080	808	972
25/05/15 17:10	804	1080	962	1140
25/05/15 17:15	828	1080	747	804
25/05/15 17:20	672	1080	874	696
25/05/15 17:25	852	1080	686	516
25/05/15 17:30	660	1080	361	924
25/05/15 17:35	720	1080	408	816
25/05/15 17:40	1008	1080	378	852

Sistema de Controle de Tráfego Urbano OPTIMUS

5 MINUTOS	INTENSIDADE			
	PM 04030 04	PM 04030 06	PM 04040 02	PM 04040 04
25/05/15 17:45	900	1080	375	876
25/05/15 17:50	816	1080	394	1188
25/05/15 17:55	792	1080	375	900
25/05/15 18:00	1008	1080	356	1068
25/05/15 18:05	792	1080	733	948
25/05/15 18:10	756	1080	697	1044
25/05/15 18:15	696	1080	589	804
25/05/15 18:20	816	1080	766	1068
25/05/15 18:25	804	1080	663	720
25/05/15 18:30	840	1044	773	948
25/05/15 18:35	684	900	534	828
25/05/15 18:40	732	900	806	624
25/05/15 18:45	588	900	607	780
25/05/15 18:50	588	900	552	936
25/05/15 18:55	756	900	567	840
25/05/15 19:00	540	900	757	828
25/05/15 19:05	396	900	434	876
25/05/15 19:10	612	900	730	948
25/05/15 19:15	588	900	694	912
25/05/15 19:20	516	900	602	792
25/05/15 19:25	600	900	674	1056
25/05/15 19:30	492	900	680	840
25/05/15 19:35	468	900	483	720
25/05/15 19:40	444	900	560	840
25/05/15 19:45	624	900	488	852
25/05/15 19:50	480	900	614	720
25/05/15 19:55	384	900	446	972
25/05/15 20:00	336	900	510	732
25/05/15 20:05	348	900	346	696
25/05/15 20:10	408	900	494	960
25/05/15 20:15	324	900	334	708
25/05/15 20:20	444	900	511	672
25/05/15 20:25	432	900	274	816
25/05/15 20:30	288	900	390	864
25/05/15 20:35	336	900	259	648
25/05/15 20:40	264	900	450	756
25/05/15 20:45	324	900	322	696
25/05/15 20:50	252	900	365	792
25/05/15 20:55	348	900	390	756
25/05/15 21:00	288	900	273	540
25/05/15 21:05	324	900	444	708
25/05/15 21:10	324	900	375	1032
25/05/15 21:15	240	900	518	1224
25/05/15 21:20	252	900	294	972
25/05/15 21:25	300	900	375	1044
25/05/15 21:30	288	900	299	996
25/05/15 21:35	240	900	273	972
25/05/15 21:40	348	900	326	612
25/05/15 21:45	252	864	276	744
25/05/15 21:50	240	720	295	540
25/05/15 21:55	300	720	447	636
25/05/15 22:00	156	720	492	696
25/05/15 22:05	276	720	381	588
25/05/15 22:10	252	720	394	564
25/05/15 22:15	396	720	517	744
25/05/15 22:20	288	720	664	876
25/05/15 22:25	228	720	782	708
25/05/15 22:30	288	720	728	588

Sistema de Controle de Tráfego Urbano OPTIMUS

5 MINUTOS	INTENSIDADE			
	PM 0403004	PM 0403006	PM 0404002	PM 0404004
25/05/15 22:35	264	720	687	576
25/05/15 22:40	192	720	538	744
25/05/15 22:45	228	720	374	480
25/05/15 22:50	156	720	355	504
25/05/15 22:55	240	720	278	336
25/05/15 23:00	120	720	171	324
25/05/15 23:05	216	720	220	480
25/05/15 23:10	180	720	170	360
25/05/15 23:15	180	684	133	336
25/05/15 23:20	96	540	106	432
25/05/15 23:25	48	540	114	324
25/05/15 23:30	72	504	82	372
25/05/15 23:35	168	360	70	252
25/05/15 23:40	168	360	105	300
25/05/15 23:45	48	360	79	216
25/05/15 23:50	144	360	100	240
25/05/15 23:55	180	360	27	276
26/05/15 00:00	108	324	30	192
26/05/15 00:05	60	180	74	168
26/05/15 00:10	96	180	75	204
26/05/15 00:15	132	180	63	228
26/05/15 00:20	36	180	34	204
26/05/15 00:25	36	180	72	144
26/05/15 00:30	36	180	38	168
26/05/15 00:35	36	180	94	96
26/05/15 00:40	24	180	63	144
26/05/15 00:45	24	180	55	180
26/05/15 00:50	12	180	15	84
26/05/15 00:55	48	180	7	120
26/05/15 01:00	12	144	27	108
26/05/15 01:05	12	0	36	84
26/05/15 01:10	48	0	46	108
26/05/15 01:15	12	0	36	60
26/05/15 01:20	24	0	38	144
26/05/15 01:25	48	0	12	60
26/05/15 01:30	48	0	48	108
26/05/15 01:35	48	0	51	108
26/05/15 01:40	36	0	34	60
26/05/15 01:45	0	0	24	96
26/05/15 01:50	12	0	27	132
26/05/15 01:55	24	0	12	72
26/05/15 02:00	12	0	0	72
26/05/15 02:05	12	0	0	84
26/05/15 02:10	48	0	0	96
26/05/15 02:15	0	0	15	60
26/05/15 02:20	36	0	24	120
26/05/15 02:25	24	0	24	120
26/05/15 02:30	36	0	0	108
26/05/15 02:35	36	0	36	48
26/05/15 02:40	12	0	31	48
26/05/15 02:45	24	0	12	60
26/05/15 02:50	12	0	15	36
26/05/15 02:55	24	0	3	12
26/05/15 03:00	12	0	12	36
26/05/15 03:05	24	0	12	48
26/05/15 03:10	0	0	0	60
26/05/15 03:15	12	0	24	48
26/05/15 03:20	24	0	27	36

Sistema de Controle de Tráfego Urbano OPTIMUS

5 MINUTOS	INTENSIDADE			
	PM 040300 4	PM 040300 6	PM 040400 2	PM 040400 4
26/05/15 03:25	24	0	0	36
26/05/15 03:30	0	0	15	12
26/05/15 03:35	12	0	39	48
26/05/15 03:40	0	0	27	12
26/05/15 03:45	24	0	12	36
26/05/15 03:50	0	0	36	36
26/05/15 03:55	36	0	0	24
26/05/15 04:00	24	0	0	72
26/05/15 04:05	0	0	0	24
26/05/15 04:10	24	0	0	84
26/05/15 04:15	12	0	0	72
26/05/15 04:20	24	0	0	72
26/05/15 04:25	12	0	0	108
26/05/15 04:30	24	0	10	84
26/05/15 04:35	48	0	36	120
26/05/15 04:40	24	0	3	60
26/05/15 04:45	0	0	12	96
26/05/15 04:50	60	0	60	144
26/05/15 04:55	48	0	7	84
26/05/15 05:00	12	0	46	72
26/05/15 05:05	144	0	15	276
26/05/15 05:10	36	0	3	132
26/05/15 05:15	60	0	48	108
26/05/15 05:20	60	0	19	192
26/05/15 05:25	12	0	55	192
26/05/15 05:30	120	0	55	276
26/05/15 05:35	96	0	91	144
26/05/15 05:40	96	0	86	300
26/05/15 05:45	168	0	91	216
26/05/15 05:50	168	0	154	324
26/05/15 05:55	240	0	166	348
26/05/15 06:00	228	0	87	384
26/05/15 06:05	336	0	142	516
26/05/15 06:10	264	0	117	324
26/05/15 06:15	552	0	166	516
26/05/15 06:20	480	0	150	408
26/05/15 06:25	636	0	194	696
26/05/15 06:30	864	0	249	780
26/05/15 06:35	984	0	193	804
26/05/15 06:40	1296	0	223	1164
26/05/15 06:45	1512	0	140	1440
26/05/15 06:50	1764	0	452	1176
26/05/15 06:55	1620	0	517	1248
26/05/15 07:00	1872	0	733	1296
26/05/15 07:05	1908	0	423	1656
26/05/15 07:10	1908	0	715	1344
26/05/15 07:15	2232	0	740	1836
26/05/15 07:20	1860	0	426	1272
26/05/15 07:25	1980	0	505	1536
26/05/15 07:30	1656	0	376	1152
26/05/15 07:35	1968	0	489	1824
26/05/15 07:40	1824	0	484	1260
26/05/15 07:45	2052	0	484	1788
26/05/15 07:50	1932	0	444	1476
26/05/15 07:55	1980	0	566	1680
26/05/15 08:00	1872	0	459	1380
26/05/15 08:05	1764	0	549	1584
26/05/15 08:10	1416	0	537	1320

Sistema de Controle de Tráfego Urbano OPTIMUS

5 MINUTOS	INTENSIDADE			
	PM 040300 4	PM 040300 6	PM 040400 2	PM 040400 4
26/05/15 08:15	1380	36	363	1584
26/05/15 08:20	1380	180	356	1452
26/05/15 08:25	1716	180	469	1608
26/05/15 08:30	1392	216	583	1380
26/05/15 08:35	1596	360	502	1392
26/05/15 08:40	1200	360	453	1188
26/05/15 08:45	1404	396	628	1236
26/05/15 08:50	1488	540	452	1236
26/05/15 08:55	1680	540	607	1596
26/05/15 09:00	1188	576	451	1284
26/05/15 09:05	1332	720	549	1344
26/05/15 09:10	1164	720	562	1236
26/05/15 09:15	1248	720	620	1620
26/05/15 09:20	1140	720	691	1128
26/05/15 09:25	900	720	742	1428
26/05/15 09:30	1116	720	588	1176
26/05/15 09:35	1008	720	732	1212
26/05/15 09:40	840	720	631	888
26/05/15 09:45	948	720	709	984
26/05/15 09:50	840	720	493	1356
26/05/15 09:55	876	720	596	1080
26/05/15 10:00	864	720	586	1212
26/05/15 10:05	900	720	656	1104
26/05/15 10:10	804	720	565	1032
26/05/15 10:15	936	720	650	996
26/05/15 10:20	948	720	652	1200
26/05/15 10:25	1044	720	589	1092
26/05/15 10:30	1140	720	614	1104
26/05/15 10:35	1032	720	769	972
26/05/15 10:40	660	720	490	1116
26/05/15 10:45	660	720	678	852
26/05/15 10:50	816	720	632	1488
26/05/15 10:55	708	720	502	1080
26/05/15 11:00	660	720	506	1116
26/05/15 11:05	600	720	735	1164
26/05/15 11:10	816	720	567	1080
26/05/15 11:15	768	720	764	1272
26/05/15 11:20	744	720	686	1008
26/05/15 11:25	636	720	774	996
26/05/15 11:30	612	720	735	1104
26/05/15 11:35	852	720	678	1284
26/05/15 11:40	636	720	681	888
26/05/15 11:45	720	756	655	936
26/05/15 11:50	672	900	792	1440
26/05/15 11:55	768	900	706	948
26/05/15 12:00	672	900	651	1056
26/05/15 12:05	768	900	796	1032
26/05/15 12:10	636	900	596	1008
26/05/15 12:15	708	900	793	1116
26/05/15 12:20	648	900	728	1128
26/05/15 12:25	708	900	789	744
26/05/15 12:30	780	900	718	1272
26/05/15 12:35	888	900	648	912
26/05/15 12:40	780	900	640	1176
26/05/15 12:45	1008	900	627	1044
26/05/15 12:50	924	900	802	1068
26/05/15 12:55	1368	900	805	900
26/05/15 13:00	948	900	826	1200

Sistema de Controle de Tráfego Urbano OPTIMUS

5 MINUTOS	INTENSIDADE			
	PM 040300 4	PM 040300 6	PM 040400 2	PM 040400 4
26/05/15 13:05	1104	900	751	1284
26/05/15 13:10	996	900	860	1140
26/05/15 13:15	996	900	822	1344
26/05/15 13:20	912	900	734	1272
26/05/15 13:25	1056	900	720	1212
26/05/15 13:30	876	900	718	1248
26/05/15 13:35	1152	900	643	1380
26/05/15 13:40	1212	900	500	1152
26/05/15 13:45	1092	900	734	1392
26/05/15 13:50	984	900	567	1284
26/05/15 13:55	840	900	588	1260
26/05/15 14:00	924	900	465	1416
26/05/15 14:05	1020	900	681	1188
26/05/15 14:10	960	900	508	1440
26/05/15 14:15	756	900	650	1140
26/05/15 14:20	1128	900	703	1440
26/05/15 14:25	900	900	572	1092
26/05/15 14:30	828	900	825	1140
26/05/15 14:35	924	900	769	1140
26/05/15 14:40	948	900	687	1416
26/05/15 14:45	840	900	599	1248
26/05/15 14:50	828	900	820	1092
26/05/15 14:55	636	900	470	1392
26/05/15 15:00	732	900	717	1356
26/05/15 15:05	720	900	612	1152
26/05/15 15:10	792	900	714	1104
26/05/15 15:15	612	900	651	996
26/05/15 15:20	828	900	879	1116
26/05/15 15:25	660	900	586	1404
26/05/15 15:30	660	900	793	1320
26/05/15 15:35	612	900	575	1032
26/05/15 15:40	816	900	772	1116
26/05/15 15:45	768	900	528	1296
26/05/15 15:50	816	900	778	1416
26/05/15 15:55	828	900	572	1272
26/05/15 16:00	744	900	957	984
26/05/15 16:05	708	900	654	1128
26/05/15 16:10	600	900	1008	1176
26/05/15 16:15	540	900	685	1044
26/05/15 16:20	732	900	996	1248
26/05/15 16:25	744	900	667	912
26/05/15 16:30	804	900	776	1308
26/05/15 16:35	732	900	610	1068
26/05/15 16:40	720	900	799	972
26/05/15 16:45	540	900	608	1020
26/05/15 16:50	852	900	997	1068
26/05/15 16:55	816	900	757	816
26/05/15 17:00	756	900	892	1308
26/05/15 17:05	696	900	800	1152
26/05/15 17:10	756	900	933	1104
26/05/15 17:15	792	900	717	1056
26/05/15 17:20	768	900	828	912
26/05/15 17:25	888	900	646	792
26/05/15 17:30	636	900	811	756
26/05/15 17:35	804	900	542	804
26/05/15 17:40	1008	900	799	588
26/05/15 17:45	792	900	528	864
26/05/15 17:50	672	900	334	996

Sistema de Controle de Tráfego Urbano OPTIMUS

5 MINUTOS	INTENSIDADE			
	PM 0403004	PM 0403006	PM 0404002	PM 0404004
26/05/15 17:55	672	900	350	936
26/05/15 18:00	912	900	325	960
26/05/15 18:05	888	900	468	708
26/05/15 18:10	864	900	447	684
26/05/15 18:15	732	900	364	744
26/05/15 18:20	864	900	264	852
26/05/15 18:25	864	900	505	888
26/05/15 18:30	780	900	246	624
26/05/15 18:35	948	900	296	912
26/05/15 18:40	960	900	438	804
26/05/15 18:45	840	900	426	1212
26/05/15 18:50	816	900	578	1080
26/05/15 18:55	636	900	508	1032
26/05/15 19:00	612	900	612	1212
26/05/15 19:05	480	900	555	816
26/05/15 19:10	588	900	758	984
26/05/15 19:15	624	900	595	1056
26/05/15 19:20	540	900	830	1044
26/05/15 19:25	696	900	620	948
26/05/15 19:30	504	900	546	732
26/05/15 19:35	444	900	398	924
26/05/15 19:40	552	900	494	1008
26/05/15 19:45	384	900	494	924
26/05/15 19:50	444	900	468	744
26/05/15 19:55	348	900	388	744
26/05/15 20:00	576	900	603	936
26/05/15 20:05	612	900	420	780
26/05/15 20:10	444	900	535	756
26/05/15 20:15	372	900	301	828
26/05/15 20:20	444	900	584	888
26/05/15 20:25	288	900	366	1032
26/05/15 20:30	492	936	346	636
26/05/15 20:35	360	1080	285	636
26/05/15 20:40	408	1080	567	684
26/05/15 20:45	240	1044	441	684
26/05/15 20:50	384	900	466	888
26/05/15 20:55	396	900	435	996
26/05/15 21:00	324	900	309	720
26/05/15 21:05	456	900	537	672
26/05/15 21:10	324	900	510	768
26/05/15 21:15	312	900	412	672
26/05/15 21:20	360	900	360	744
26/05/15 21:25	348	900	414	732
26/05/15 21:30	336	900	242	672
26/05/15 21:35	300	900	374	624
26/05/15 21:40	336	900	368	744
26/05/15 21:45	372	864	252	648
26/05/15 21:50	216	720	338	672
26/05/15 21:55	228	720	390	588
26/05/15 22:00	324	720	487	588
26/05/15 22:05	264	720	583	816
26/05/15 22:10	408	720	667	864
26/05/15 22:15	336	720	580	852
26/05/15 22:20	228	720	542	648
26/05/15 22:25	264	720	710	780
26/05/15 22:30	264	720	601	708
26/05/15 22:35	312	720	674	708
26/05/15 22:40	240	720	690	684

Sistema de Controle de Tráfego Urbano OPTIMUS

5 MINUTOS	INTENSIDADE			
	PM040300 4	PM040300 6	PM040400 2	PM040400 4
26/05/15 22:45	288	720	409	540
26/05/15 22:50	204	720	355	480
26/05/15 22:55	240	720	261	564
26/05/15 23:00	168	720	296	564
26/05/15 23:05	132	720	344	564
26/05/15 23:10	252	720	274	456
26/05/15 23:15	204	720	162	396
26/05/15 23:20	168	720	184	444
26/05/15 23:25	96	720	193	348
26/05/15 23:30	144	684	177	396
26/05/15 23:35	120	540	69	408
26/05/15 23:40	216	540	82	384
26/05/15 23:45	168	504	141	336
26/05/15 23:50	144	360	106	336
26/05/15 23:55	120	360	103	324
27/05/15 00:00	168	324	58	336
27/05/15 00:05	108	180	110	228
27/05/15 00:10	24	180	142	240
27/05/15 00:15	72	180	63	180
27/05/15 00:20	48	180	123	180
27/05/15 00:25	108	180	86	168
27/05/15 00:30	48	180	75	216
27/05/15 00:35	96	180	106	132
27/05/15 00:40	72	180	103	228
27/05/15 00:45	60	180	62	156
27/05/15 00:50	132	180	74	144
27/05/15 00:55	36	180	39	132
27/05/15 01:00	48	180	67	240
27/05/15 01:05	36	180	36	156
27/05/15 01:10	24	180	19	168
27/05/15 01:15	36	180	43	156
27/05/15 01:20	24	180	31	72
27/05/15 01:25	12	180	31	72
27/05/15 01:30	48	180	48	96
27/05/15 01:35	48	180	36	72
27/05/15 01:40	24	180	46	96
27/05/15 01:45	12	144	24	60
27/05/15 01:50	36	0	19	168
27/05/15 01:55	12	0	12	132
27/05/15 02:00	36	0	27	96
27/05/15 02:05	24	0	3	72
27/05/15 02:10	12	0	27	60
27/05/15 02:15	12	0	24	132
27/05/15 02:20	36	0	24	96
27/05/15 02:25	24	0	63	84
27/05/15 02:30	36	0	12	72
27/05/15 02:35	24	0	39	48
27/05/15 02:40	36	0	60	108
27/05/15 02:45	0	0	36	48
27/05/15 02:50	36	0	0	24
27/05/15 02:55	60	0	0	48
27/05/15 03:00	24	0	15	48
27/05/15 03:05	0	0	0	60
27/05/15 03:10	12	0	0	72
27/05/15 03:15	48	0	24	60
27/05/15 03:20	12	0	31	144
27/05/15 03:25	24	0	19	36
27/05/15 03:30	12	0	12	60

Sistema de Controle de Tráfego Urbano OPTIMUS

5 MINUTOS	INTENSIDADE			
	PM 0403004	PM 0403006	PM 0404002	PM 0404004
27/05/15 03:35	0	0	36	0
27/05/15 03:40	0	0	36	36
27/05/15 03:45	24	0	7	60
27/05/15 03:50	24	0	3	72
27/05/15 03:55	0	0	0	48
27/05/15 04:00	0	0	36	48
27/05/15 04:05	36	0	12	60
27/05/15 04:10	0	0	0	144
27/05/15 04:15	12	0	48	144
27/05/15 04:20	24	0	24	84
27/05/15 04:25	12	0	0	48
27/05/15 04:30	0	0	12	48
27/05/15 04:35	24	0	43	96
27/05/15 04:40	36	0	15	96
27/05/15 04:45	48	0	3	144
27/05/15 04:50	36	0	67	84
27/05/15 04:55	0	0	12	72
27/05/15 05:00	36	0	34	60
27/05/15 05:05	48	0	51	120
27/05/15 05:10	84	0	15	216
27/05/15 05:15	36	0	24	108
27/05/15 05:20	48	0	55	192
27/05/15 05:25	84	0	58	192
27/05/15 05:30	84	0	51	216
27/05/15 05:35	96	0	48	264
27/05/15 05:40	120	0	99	276
27/05/15 05:45	156	0	58	276
27/05/15 05:50	156	0	79	252
27/05/15 05:55	264	0	127	456
27/05/15 06:00	264	0	122	396
27/05/15 06:05	384	0	199	408
27/05/15 06:10	300	0	205	480
27/05/15 06:15	516	0	146	396
27/05/15 06:20	420	0	177	636
27/05/15 06:25	576	0	157	672
27/05/15 06:30	960	0	222	756
27/05/15 06:35	1008	0	229	816
27/05/15 06:40	1236	0	97	1236
27/05/15 06:45	1632	0	229	1536
27/05/15 06:50	1536	0	459	1260
27/05/15 06:55	1848	0	523	1128
27/05/15 07:00	1620	0	639	1308
27/05/15 07:05	2136	0	588	1452
27/05/15 07:10	1752	0	648	1344
27/05/15 07:15	2136	0	778	2016
27/05/15 07:20	1704	0	531	1368
27/05/15 07:25	1908	0	535	1464
27/05/15 07:30	1812	0	423	1296
27/05/15 07:35	1956	0	475	1632
27/05/15 07:40	1824	0	540	1752
27/05/15 07:45	2040	0	638	1704
27/05/15 07:50	1656	0	457	1416
27/05/15 07:55	2100	0	550	1788
27/05/15 08:00	1776	0	550	1260
27/05/15 08:05	1776	0	507	1452
27/05/15 08:10	1476	0	349	1572
27/05/15 08:15	1764	0	512	1548
27/05/15 08:20	1452	0	475	1440

Sistema de Controle de Tráfego Urbano OPTIMUS

5 MINUTOS	INTENSIDADE			
	PM040300 4	PM040300 6	PM040400 2	PM040400 4
27/05/15 08:25	1428	0	585	1776
27/05/15 08:30	1392	0	520	1284
27/05/15 08:35	1512	0	604	1308
27/05/15 08:40	1728	0	477	1320
27/05/15 08:45	1452	36	632	1644
27/05/15 08:50	1524	180	520	1440
27/05/15 08:55	1272	180	511	1452
27/05/15 09:00	1488	216	504	1284
27/05/15 09:05	1392	360	427	1440
27/05/15 09:10	1572	360	627	924
27/05/15 09:15	1104	396	592	1392
27/05/15 09:20	1164	540	686	1248
27/05/15 09:25	948	540	564	1248
27/05/15 09:30	1296	576	510	1320
27/05/15 09:35	996	720	547	1176
27/05/15 09:40	1044	720	554	1104
27/05/15 09:45	936	720	481	1224
27/05/15 09:50	852	720	547	996
27/05/15 09:55	960	720	620	1284
27/05/15 10:00	780	720	517	1248
27/05/15 10:05	660	720	625	1380
27/05/15 10:10	828	720	645	936
27/05/15 10:15	780	720	517	996
27/05/15 10:20	516	720	639	1344
27/05/15 10:25	768	720	519	1128
27/05/15 10:30	768	720	681	1200
27/05/15 10:35	720	720	594	888
27/05/15 10:40	696	720	537	1176
27/05/15 10:45	996	720	496	1092
27/05/15 10:50	672	720	652	1152
27/05/15 10:55	804	720	660	936
27/05/15 11:00	780	720	556	1320
27/05/15 11:05	660	720	730	888
27/05/15 11:10	600	720	675	1248
27/05/15 11:15	780	720	540	1092
27/05/15 11:20	840	720	625	1236
27/05/15 11:25	780	720	684	852
27/05/15 11:30	540	756	693	1416
27/05/15 11:35	648	900	703	1068
27/05/15 11:40	636	900	708	660
27/05/15 11:45	768	900	753	1080
27/05/15 11:50	636	900	915	1320
27/05/15 11:55	840	900	751	1140
27/05/15 12:00	624	900	601	1032
27/05/15 12:05	612	900	805	1032
27/05/15 12:10	684	900	744	1176
27/05/15 12:15	804	900	730	1176
27/05/15 12:20	780	900	592	1056
27/05/15 12:25	720	900	620	1128
27/05/15 12:30	804	900	693	1272
27/05/15 12:35	840	900	562	1068
27/05/15 12:40	816	900	759	1140
27/05/15 12:45	1032	900	632	936
27/05/15 12:50	936	900	805	1020
27/05/15 12:55	1152	900	748	1032
27/05/15 13:00	1032	900	697	984
27/05/15 13:05	1188	900	732	1464
27/05/15 13:10	972	900	810	1296

Sistema de Controle de Tráfego Urbano OPTIMUS

5 MINUTOS	INTENSIDADE			
	PM 040300 4	PM 040300 6	PM 040400 2	PM 040400 4
27/05/15 13:15	1032	900	868	1440
27/05/15 13:20	900	900	725	924
27/05/15 13:25	948	900	717	1224
27/05/15 13:30	1116	900	786	1476
27/05/15 13:35	996	900	562	984
27/05/15 13:40	1164	900	759	1644
27/05/15 13:45	828	900	710	1104
27/05/15 13:50	1116	900	718	1512
27/05/15 13:55	936	900	786	1296
27/05/15 14:00	924	936	688	1212
27/05/15 14:05	1092	1080	646	1488
27/05/15 14:10	936	1080	648	1488
27/05/15 14:15	912	1080	615	1632
27/05/15 14:20	804	1080	700	1200
27/05/15 14:25	1092	1080	740	1332
27/05/15 14:30	804	1044	603	1380
27/05/15 14:35	972	900	730	1056
27/05/15 14:40	816	900	750	1056
27/05/15 14:45	768	900	524	1188
27/05/15 14:50	912	900	753	1248
27/05/15 14:55	732	900	779	1032
27/05/15 15:00	732	900	678	1344
27/05/15 15:05	792	900	616	1248
27/05/15 15:10	828	900	831	1236
27/05/15 15:15	756	900	528	1200
27/05/15 15:20	780	900	590	1380
27/05/15 15:25	732	900	637	1356
27/05/15 15:30	876	900	694	1164
27/05/15 15:35	780	900	714	1440
27/05/15 15:40	720	900	655	1248
27/05/15 15:45	672	900	552	1140
27/05/15 15:50	804	900	700	1068
27/05/15 15:55	756	900	614	1044
27/05/15 16:00	684	900	896	1452
27/05/15 16:05	588	900	770	1140
27/05/15 16:10	708	900	904	1152
27/05/15 16:15	648	900	688	1308
27/05/15 16:20	636	900	894	1008
27/05/15 16:25	780	900	874	1140
27/05/15 16:30	624	900	922	1500
27/05/15 16:35	804	900	549	1224
27/05/15 16:40	600	900	880	852
27/05/15 16:45	708	900	736	1248
27/05/15 16:50	684	900	835	924
27/05/15 16:55	648	900	907	888
27/05/15 17:00	636	936	822	1464
27/05/15 17:05	564	1080	890	984
27/05/15 17:10	768	1080	1024	1212
27/05/15 17:15	768	1080	850	1116
27/05/15 17:20	588	1080	549	516
27/05/15 17:25	816	1080	498	540
27/05/15 17:30	708	1080	310	708
27/05/15 17:35	768	1080	432	360
27/05/15 17:40	936	1080	217	720
27/05/15 17:45	504	1116	398	864
27/05/15 17:50	1056	1260	400	1020
27/05/15 17:55	612	1260	228	756
27/05/15 18:00	924	1260	307	816

Sistema de Controle de Tráfego Urbano OPTIMUS

5 MINUTOS	INTENSIDADE			
	PM 040300 4	PM 040300 6	PM 040400 2	PM 040400 4
27/05/15 18:05	588	1260	277	468
27/05/15 18:10	600	1260	343	684
27/05/15 18:15	744	1260	559	708
27/05/15 18:20	672	1260	183	708
27/05/15 18:25	828	1260	219	708
27/05/15 18:30	1056	1224	259	720
27/05/15 18:35	396	1080	324	804
27/05/15 18:40	600	1080	373	840
27/05/15 18:45	816	1080	378	660
27/05/15 18:50	732	1080	438	672
27/05/15 18:55	960	1080	577	420
27/05/15 19:00	276	1080	714	1128
27/05/15 19:05	996	1080	585	912
27/05/15 19:10	804	1080	625	936
27/05/15 19:15	540	1080	571	936
27/05/15 19:20	516	1080	778	852
27/05/15 19:25	672	1080	716	1008
27/05/15 19:30	684	1080	632	972
27/05/15 19:35	624	1080	644	948
27/05/15 19:40	516	1080	667	672
27/05/15 19:45	456	1080	366	1116
27/05/15 19:50	540	1080	476	648
27/05/15 19:55	540	1080	428	1008
27/05/15 20:00	396	1080	510	840
27/05/15 20:05	288	1080	390	684
27/05/15 20:10	504	1080	441	864
27/05/15 20:15	492	1044	462	948
27/05/15 20:20	276	900	432	828
27/05/15 20:25	540	900	450	720
27/05/15 20:30	288	900	364	756
27/05/15 20:35	396	900	370	492
27/05/15 20:40	348	900	409	888
27/05/15 20:45	300	900	534	792
27/05/15 20:50	300	900	376	660
27/05/15 20:55	324	900	493	744
27/05/15 21:00	360	900	325	612
27/05/15 21:05	240	900	442	840
27/05/15 21:10	324	900	445	576
27/05/15 21:15	432	900	398	876
27/05/15 21:20	408	900	284	888
27/05/15 21:25	276	900	316	804
27/05/15 21:30	336	900	286	696
27/05/15 21:35	288	900	307	600
27/05/15 21:40	336	900	425	684
27/05/15 21:45	264	864	530	672
27/05/15 21:50	300	720	202	624
27/05/15 21:55	348	720	328	552
27/05/15 22:00	288	720	414	624
27/05/15 22:05	336	720	546	636
27/05/15 22:10	324	720	462	576
27/05/15 22:15	312	720	550	636
27/05/15 22:20	180	720	345	816
27/05/15 22:25	348	720	523	384
27/05/15 22:30	252	720	571	792
27/05/15 22:35	168	720	632	480
27/05/15 22:40	168	720	404	456
27/05/15 22:45	120	720	206	420
27/05/15 22:50	144	720	260	396

Sistema de Controle de Tráfego Urbano OPTIMUS

5 MINUTOS	INTENSIDADE			
	PM 0403004	PM 0403006	PM 0404002	PM 0404004
27/05/15 22:55	156	720	193	480
27/05/15 23:00	216	720	210	396
27/05/15 23:05	132	720	254	300
27/05/15 23:10	228	720	234	264
27/05/15 23:15	192	684	134	360
27/05/15 23:20	120	540	166	348
27/05/15 23:25	132	540	110	372
27/05/15 23:30	108	504	86	372
27/05/15 23:35	168	360	106	336
27/05/15 23:40	108	360	90	312
27/05/15 23:45	132	360	158	336
27/05/15 23:50	168	360	129	264
27/05/15 23:55	168	360	62	240
28/05/15 00:00	96	324	94	276
28/05/15 00:05	204	180	178	396
28/05/15 00:10	180	180	90	384
28/05/15 00:15	156	180	102	372
28/05/15 00:20	156	180	67	300
28/05/15 00:25	84	180	58	192
28/05/15 00:30	108	180	126	420
28/05/15 00:35	96	180	91	300
28/05/15 00:40	120	180	67	132
28/05/15 00:45	108	180	90	228
28/05/15 00:50	84	180	31	324
28/05/15 00:55	132	180	51	288
28/05/15 01:00	180	180	79	204
28/05/15 01:05	48	180	50	156
28/05/15 01:10	96	180	62	144
28/05/15 01:15	120	144	51	156
28/05/15 01:20	60	0	22	168
28/05/15 01:25	60	0	58	132
28/05/15 01:30	60	0	19	72
28/05/15 01:35	48	0	51	144
28/05/15 01:40	72	0	84	96
28/05/15 01:45	72	0	39	108
28/05/15 01:50	12	0	15	132
28/05/15 01:55	36	0	75	72
28/05/15 02:00	24	0	43	84
28/05/15 02:05	12	0	51	72
28/05/15 02:10	24	0	3	84
28/05/15 02:15	36	0	24	108
28/05/15 02:20	72	0	3	84
28/05/15 02:25	60	0	19	108
28/05/15 02:30	12	0	43	132
28/05/15 02:35	12	0	63	72
28/05/15 02:40	60	0	24	72
28/05/15 02:45	24	0	0	60
28/05/15 02:50	36	0	43	24
28/05/15 02:55	12	0	36	108
28/05/15 03:00	0	0	15	96
28/05/15 03:05	0	0	12	96
28/05/15 03:10	24	0	0	48
28/05/15 03:15	24	0	12	84
28/05/15 03:20	36	0	3	60
28/05/15 03:25	0	0	36	48
28/05/15 03:30	24	0	12	36
28/05/15 03:35	24	0	12	36
28/05/15 03:40	24	0	12	0

Sistema de Controle de Tráfego Urbano OPTIMUS

5 MINUTOS	INTENSIDADE			
	PM 0403004	PM 0403006	PM 0404002	PM 0404004
28/05/15 03:45	12	0	3	84
28/05/15 03:50	12	0	36	60
28/05/15 03:55	12	0	27	36
28/05/15 04:00	60	0	39	24
28/05/15 04:05	24	0	0	84
28/05/15 04:10	12	0	15	84
28/05/15 04:15	48	0	24	180
28/05/15 04:20	12	0	3	84
28/05/15 04:25	12	0	15	72
28/05/15 04:30	60	0	24	120
28/05/15 04:35	48	0	39	48
28/05/15 04:40	48	0	0	48
28/05/15 04:45	12	0	3	228
28/05/15 04:50	48	0	36	96
28/05/15 04:55	36	0	24	60
28/05/15 05:00	0	0	39	84
28/05/15 05:05	60	0	48	156
28/05/15 05:10	120	0	48	108
28/05/15 05:15	48	0	15	168
28/05/15 05:20	48	0	43	252
28/05/15 05:25	84	0	51	84
28/05/15 05:30	72	0	34	192
28/05/15 05:35	96	0	75	156
28/05/15 05:40	108	0	82	276
28/05/15 05:45	216	0	111	144
28/05/15 05:50	132	0	122	300
28/05/15 05:55	228	0	138	348
28/05/15 06:00	264	0	150	300
28/05/15 06:05	312	0	110	432
28/05/15 06:10	228	0	122	288
28/05/15 06:15	336	0	166	444
28/05/15 06:20	444	0	182	600
28/05/15 06:25	600	0	187	684
28/05/15 06:30	708	0	213	720
28/05/15 06:35	1080	0	156	924
28/05/15 06:40	1320	0	202	1200
28/05/15 06:45	1584	0	458	1308
28/05/15 06:50	1944	0	337	1164
28/05/15 06:55	1692	0	525	1308
28/05/15 07:00	2100	0	540	1344
28/05/15 07:05	1776	0	814	1740
28/05/15 07:10	1824	0	500	1344
28/05/15 07:15	1764	0	829	1584
28/05/15 07:20	1968	0	471	1668
28/05/15 07:25	1836	0	601	1488
28/05/15 07:30	2028	0	633	1116
28/05/15 07:35	2076	0	579	1392
28/05/15 07:40	1680	0	666	1500
28/05/15 07:45	2172	0	374	1524
28/05/15 07:50	1644	0	243	1512
28/05/15 07:55	1488	0	562	1428
28/05/15 08:00	1656	0	409	1440
28/05/15 08:05	1668	0	589	1452
28/05/15 08:10	1428	0	529	1320
28/05/15 08:15	1392	0	458	1356
28/05/15 08:20	1320	0	573	1248
28/05/15 08:25	1380	0	440	1596
28/05/15 08:30	1356	36	364	1296

Sistema de Controle de Tráfego Urbano OPTIMUS

5 MINUTOS	INTENSIDADE			
	PM 040300 4	PM 040300 6	PM 040400 2	PM 040400 4
28/05/15 08:35	1524	180	447	1368
28/05/15 08:40	1428	180	508	1272
28/05/15 08:45	1308	180	608	1200
28/05/15 08:50	1620	180	446	1416
28/05/15 08:55	1512	180	571	1380
28/05/15 09:00	1476	216	423	1128
28/05/15 09:05	1260	360	517	1152
28/05/15 09:10	996	360	566	1164
28/05/15 09:15	972	396	626	948
28/05/15 09:20	1116	540	562	1296
28/05/15 09:25	1092	540	657	1272
28/05/15 09:30	960	576	718	972
28/05/15 09:35	1032	720	710	1212
28/05/15 09:40	936	720	566	1176
28/05/15 09:45	936	720	685	1044
28/05/15 09:50	924	720	618	1260
28/05/15 09:55	1008	720	730	984
28/05/15 10:00	972	720	579	1248
28/05/15 10:05	768	720	580	1104
28/05/15 10:10	708	720	604	1296
28/05/15 10:15	696	720	645	996
28/05/15 10:20	660	720	488	1476
28/05/15 10:25	852	720	694	816
28/05/15 10:30	600	720	619	1584
28/05/15 10:35	768	720	678	960
28/05/15 10:40	696	720	627	1284
28/05/15 10:45	624	720	635	936
28/05/15 10:50	588	720	572	1092
28/05/15 10:55	780	720	831	1332
28/05/15 11:00	600	720	692	1164
28/05/15 11:05	756	720	789	1200
28/05/15 11:10	612	720	709	1188
28/05/15 11:15	780	756	661	1092
28/05/15 11:20	744	900	646	1452
28/05/15 11:25	660	900	564	1404
28/05/15 11:30	648	900	678	888
28/05/15 11:35	840	900	691	864
28/05/15 11:40	660	900	710	1104
28/05/15 11:45	840	900	736	1380
28/05/15 11:50	768	900	987	1128
28/05/15 11:55	684	900	684	1284
28/05/15 12:00	672	900	586	948
28/05/15 12:05	540	900	718	1032
28/05/15 12:10	660	900	780	864
28/05/15 12:15	684	900	664	1260
28/05/15 12:20	648	900	607	1020
28/05/15 12:25	852	900	564	924
28/05/15 12:30	780	900	489	1056
28/05/15 12:35	828	900	640	1176
28/05/15 12:40	1020	900	564	1032
28/05/15 12:45	984	936	694	1212
28/05/15 12:50	984	1080	856	1260
28/05/15 12:55	936	1080	662	1260
28/05/15 13:00	888	1080	584	1068
28/05/15 13:05	1116	1080	787	1248
28/05/15 13:10	864	1080	724	1248
28/05/15 13:15	1044	1080	721	1200
28/05/15 13:20	1164	1080	602	1320

Sistema de Controle de Tráfego Urbano OPTIMUS

5 MINUTOS	INTENSIDADE			
	PM 040300 4	PM 040300 6	PM 040400 2	PM 040400 4
28/05/15 13:25	996	1080	496	1320
28/05/15 13:30	948	1080	510	1332
28/05/15 13:35	972	1080	614	1440
28/05/15 13:40	1068	1080	710	936
28/05/15 13:45	1080	1080	566	1260
28/05/15 13:50	804	1080	673	1032
28/05/15 13:55	924	1080	687	1440
28/05/15 14:00	972	1080	562	1320
28/05/15 14:05	960	1080	627	1416
28/05/15 14:10	1032	1080	626	1272
28/05/15 14:15	912	1080	602	1524
28/05/15 14:20	972	1080	657	1212
28/05/15 14:25	912	1080	583	1428
28/05/15 14:30	900	1080	673	1392
28/05/15 14:35	864	1080	583	1416
28/05/15 14:40	768	1080	757	1272
28/05/15 14:45	1128	1080	608	1236
28/05/15 14:50	720	1080	802	1416
28/05/15 14:55	900	1080	664	1296
28/05/15 15:00	888	1080	662	1044
28/05/15 15:05	768	1080	812	1356
28/05/15 15:10	612	1080	633	1212
28/05/15 15:15	708	1080	624	1044
28/05/15 15:20	756	1080	798	1296
28/05/15 15:25	684	1080	644	1356
28/05/15 15:30	876	1080	763	1212
28/05/15 15:35	684	1080	552	1296
28/05/15 15:40	624	1080	946	1296
28/05/15 15:45	828	1080	639	1032
28/05/15 15:50	840	1080	783	1788
28/05/15 15:55	744	1080	654	1188
28/05/15 16:00	756	1080	740	1104
28/05/15 16:05	600	1080	778	948
28/05/15 16:10	456	1080	808	1320
28/05/15 16:15	756	1080	746	1092
28/05/15 16:20	732	1080	822	1044
28/05/15 16:25	732	1080	528	1104
28/05/15 16:30	552	1080	900	1008
28/05/15 16:35	648	1080	716	1164
28/05/15 16:40	696	1080	836	1140
28/05/15 16:45	732	1080	736	972
28/05/15 16:50	720	1080	936	1188
28/05/15 16:55	768	1080	854	960
28/05/15 17:00	864	1080	967	1236
28/05/15 17:05	804	1080	859	1236
28/05/15 17:10	768	1080	1006	768
28/05/15 17:15	828	1080	824	1080
28/05/15 17:20	984	1080	819	900
28/05/15 17:25	792	1080	620	1068
28/05/15 17:30	672	1080	830	804
28/05/15 17:35	804	1080	501	816
28/05/15 17:40	828	1080	537	972
28/05/15 17:45	768	1080	354	684
28/05/15 17:50	564	1080	283	972
28/05/15 17:55	648	1080	450	768
28/05/15 18:00	564	1080	283	1092
28/05/15 18:05	732	1080	338	900
28/05/15 18:10	648	1080	375	960

Sistema de Controle de Tráfego Urbano OPTIMUS

5 MINUTOS	INTENSIDADE			
	PM 040300 4	PM 040300 6	PM 040400 2	PM 040400 4
28/05/15 18:15	588	1080	469	420
28/05/15 18:20	780	1080	568	816
28/05/15 18:25	696	1080	500	708
28/05/15 18:30	888	1044	649	588
28/05/15 18:35	684	900	627	936
28/05/15 18:40	744	900	620	792
28/05/15 18:45	720	864	769	1008
28/05/15 18:50	840	720	746	936
28/05/15 18:55	720	720	545	936
28/05/15 19:00	792	720	612	624
28/05/15 19:05	672	720	530	912
28/05/15 19:10	648	720	795	732
28/05/15 19:15	696	720	533	924
28/05/15 19:20	504	720	610	1032
28/05/15 19:25	552	720	550	840
28/05/15 19:30	636	720	711	624
28/05/15 19:35	624	720	567	1320
28/05/15 19:40	588	720	636	684
28/05/15 19:45	468	720	435	864
28/05/15 19:50	540	720	605	936
28/05/15 19:55	516	720	465	1080
28/05/15 20:00	636	720	567	948
28/05/15 20:05	396	720	408	876
28/05/15 20:10	600	720	748	1140
28/05/15 20:15	480	720	456	972
28/05/15 20:20	564	720	513	960
28/05/15 20:25	420	720	427	948
28/05/15 20:30	312	720	519	672
28/05/15 20:35	396	720	434	684
28/05/15 20:40	324	720	588	564
28/05/15 20:45	300	720	492	756
28/05/15 20:50	492	720	386	684
28/05/15 20:55	312	720	412	756
28/05/15 21:00	444	720	375	792
28/05/15 21:05	336	720	354	948
28/05/15 21:10	528	720	414	1992
28/05/15 21:15	420	720	410	888
28/05/15 21:20	480	720	394	768
28/05/15 21:25	324	720	394	1032
28/05/15 21:30	300	756	370	756
28/05/15 21:35	420	900	418	672
28/05/15 21:40	240	900	471	636
28/05/15 21:45	288	864	284	936
28/05/15 21:50	252	720	390	780
28/05/15 21:55	336	720	455	588
28/05/15 22:00	276	720	526	756
28/05/15 22:05	456	720	429	840
28/05/15 22:10	384	720	448	756
28/05/15 22:15	408	720	540	936
28/05/15 22:20	312	720	712	912
28/05/15 22:25	468	720	453	828
28/05/15 22:30	312	720	466	660
28/05/15 22:35	336	720	549	912
28/05/15 22:40	228	720	582	612
28/05/15 22:45	312	720	391	828
28/05/15 22:50	216	720	342	756
28/05/15 22:55	144	720	300	480
28/05/15 23:00	180	720	304	528

Sistema de Controle de Tráfego Urbano OPTIMUS

5 MINUTOS	INTENSIDADE			
	PM 0403004	PM 0403006	PM 0404002	PM 0404004
28/05/15 23:05	396	720	163	576
28/05/15 23:10	156	720	196	336
28/05/15 23:15	144	684	210	468
28/05/15 23:20	192	540	162	504
28/05/15 23:25	204	540	128	408
28/05/15 23:30	96	504	169	516
28/05/15 23:35	120	360	106	408
28/05/15 23:40	84	360	121	312
28/05/15 23:45	192	360	104	324
28/05/15 23:50	96	360	145	432
28/05/15 23:55	168	360	141	456
29/05/15 00:00	120	360	146	360
29/05/15 00:05	204	360	146	348
29/05/15 00:10	216	360	126	348
29/05/15 00:15	144	360	86	204
29/05/15 00:20	132	360	127	240
29/05/15 00:25	144	360	55	216
29/05/15 00:30	120	324	63	288
29/05/15 00:35	48	180	109	252
29/05/15 00:40	60	180	66	240
29/05/15 00:45	96	180	86	288
29/05/15 00:50	36	180	110	204
29/05/15 00:55	60	180	110	288
29/05/15 01:00	48	180	150	264
29/05/15 01:05	36	180	63	180
29/05/15 01:10	36	180	91	96
29/05/15 01:15	36	180	79	132
29/05/15 01:20	84	180	39	264
29/05/15 01:25	60	180	70	216
29/05/15 01:30	12	180	67	168
29/05/15 01:35	36	180	43	108
29/05/15 01:40	48	180	39	120
29/05/15 01:45	24	144	74	120
29/05/15 01:50	48	0	48	132
29/05/15 01:55	48	0	24	108
29/05/15 02:00	36	0	27	120
29/05/15 02:05	24	0	55	120
29/05/15 02:10	60	0	46	96
29/05/15 02:15	36	0	72	108
29/05/15 02:20	24	0	12	108
29/05/15 02:25	36	0	51	144
29/05/15 02:30	12	0	60	120
29/05/15 02:35	12	0	48	108
29/05/15 02:40	60	0	39	24
29/05/15 02:45	12	0	27	60
29/05/15 02:50	0	0	12	84
29/05/15 02:55	48	0	48	96
29/05/15 03:00	12	0	48	36
29/05/15 03:05	12	0	0	84
29/05/15 03:10	60	0	27	108
29/05/15 03:15	0	0	60	84
29/05/15 03:20	12	0	39	48
29/05/15 03:25	12	0	0	60
29/05/15 03:30	48	0	15	60
29/05/15 03:35	36	0	15	72
29/05/15 03:40	36	0	51	48
29/05/15 03:45	24	0	24	84
29/05/15 03:50	36	0	72	72

Sistema de Controle de Tráfego Urbano OPTIMUS

5 MINUTOS	INTENSIDADE			
	PM 0403004	PM 0403006	PM 0404002	PM 0404004
29/05/15 03:55	24	0	0	72
29/05/15 04:00	24	0	24	48
29/05/15 04:05	24	0	67	36
29/05/15 04:10	24	0	12	60
29/05/15 04:15	24	0	43	84
29/05/15 04:20	36	0	27	84
29/05/15 04:25	12	0	51	60
29/05/15 04:30	24	0	15	96
29/05/15 04:35	24	0	51	132
29/05/15 04:40	48	0	34	96
29/05/15 04:45	72	0	55	96
29/05/15 04:50	48	0	7	96
29/05/15 04:55	24	0	24	72
29/05/15 05:00	72	0	34	120
29/05/15 05:05	72	0	36	132
29/05/15 05:10	36	0	54	180
29/05/15 05:15	72	0	24	240
29/05/15 05:20	60	0	51	168
29/05/15 05:25	72	0	34	972
29/05/15 05:30	96	0	55	288
29/05/15 05:35	144	0	79	252
29/05/15 05:40	120	0	82	288
29/05/15 05:45	96	0	99	228
29/05/15 05:50	240	0	91	348
29/05/15 05:55	240	0	122	336
29/05/15 06:00	300	0	106	408
29/05/15 06:05	276	0	93	420
29/05/15 06:10	288	0	122	396
29/05/15 06:15	372	0	181	444
29/05/15 06:20	468	0	186	564
29/05/15 06:25	756	0	322	756
29/05/15 06:30	660	0	225	828
29/05/15 06:35	996	0	325	900
29/05/15 06:40	1380	0	262	1356
29/05/15 06:45	1368	0	429	1380
29/05/15 06:50	1800	0	574	1248
29/05/15 06:55	1560	0	423	1476
29/05/15 07:00	1968	0	534	1284
29/05/15 07:05	1668	0	645	1800
29/05/15 07:10	2184	0	704	1380
29/05/15 07:15	1596	0	738	1428
29/05/15 07:20	1920	0	402	1824
29/05/15 07:25	1752	0	645	1680
29/05/15 07:30	1836	0	434	1656
29/05/15 07:35	1908	0	520	1584
29/05/15 07:40	1704	0	520	1356
29/05/15 07:45	1860	0	421	996
29/05/15 07:50	924	0	435	1620
29/05/15 07:55	1584	0	429	1236
29/05/15 08:00	1584	0	210	1380
29/05/15 08:05	1560	0	486	1248
29/05/15 08:10	1680	0	438	1608
29/05/15 08:15	1092	0	391	780
29/05/15 08:20	1320	0	433	1632
29/05/15 08:25	1224	0	328	1524
29/05/15 08:30	1644	36	411	1392
29/05/15 08:35	924	180	532	1464
29/05/15 08:40	1188	180	625	1140

Sistema de Controle de Tráfego Urbano OPTIMUS

5 MINUTOS	INTENSIDADE			
	PM 040300 4	PM 040300 6	PM 040400 2	PM 040400 4
29/05/15 08:45	1512	216	621	1368
29/05/15 08:50	1752	360	476	1668
29/05/15 08:55	1692	360	446	1644
29/05/15 09:00	1632	396	520	1644
29/05/15 09:05	1656	540	480	1284
29/05/15 09:10	1356	540	558	1032
29/05/15 09:15	996	576	476	1512
29/05/15 09:20	1704	720	439	1440
29/05/15 09:25	1284	720	442	1272
29/05/15 09:30	1284	756	452	1368
29/05/15 09:35	1140	900	544	1236
29/05/15 09:40	1320	900	520	1524
29/05/15 09:45	1116	900	424	1236
29/05/15 09:50	1416	900	570	1584
29/05/15 09:55	1212	900	537	1284
29/05/15 10:00	1428	900	510	1596
29/05/15 10:05	1176	900	520	1236
29/05/15 10:10	1452	900	544	1392
29/05/15 10:15	1320	900	496	1152
29/05/15 10:20	1296	900	429	1296
29/05/15 10:25	1356	900	651	1140
29/05/15 10:30	1020	900	513	1296
29/05/15 10:35	1164	900	740	1212
29/05/15 10:40	1020	900	739	1164
29/05/15 10:45	924	900	632	1452
29/05/15 10:50	888	900	493	1176
29/05/15 10:55	720	900	789	1560
29/05/15 11:00	756	900	579	1164
29/05/15 11:05	828	900	699	1080
29/05/15 11:10	780	900	690	1260
29/05/15 11:15	852	900	675	1056
29/05/15 11:20	828	900	734	1056
29/05/15 11:25	984	900	910	1488
29/05/15 11:30	576	900	788	1248
29/05/15 11:35	816	900	727	948
29/05/15 11:40	840	900	702	1332
29/05/15 11:45	864	900	767	1176
29/05/15 11:50	924	900	920	1128
29/05/15 11:55	756	900	962	1248
29/05/15 12:00	684	936	896	1092
29/05/15 12:05	900	1080	877	1440
29/05/15 12:10	648	1080	690	1128
29/05/15 12:15	876	1080	849	1152
29/05/15 12:20	948	1080	696	1320
29/05/15 12:25	768	1080	825	1116
29/05/15 12:30	828	1080	894	1284
29/05/15 12:35	840	1080	661	1032
29/05/15 12:40	912	1080	621	1176
29/05/15 12:45	1164	1080	670	1200
29/05/15 12:50	1104	1080	943	1236
29/05/15 12:55	1008	1080	842	1176
29/05/15 13:00	960	1080	891	1248
29/05/15 13:05	1140	1080	733	984
29/05/15 13:10	1128	1080	815	1260
29/05/15 13:15	1140	1080	891	1368
29/05/15 13:20	1068	1080	817	1248
29/05/15 13:25	1092	1080	733	1272
29/05/15 13:30	1068	1080	748	1296

Sistema de Controle de Tráfego Urbano OPTIMUS

5 MINUTOS	INTENSIDADE			
	PM 040300 4	PM 040300 6	PM 040400 2	PM 040400 4
29/05/15 13:35	924	1080	710	1356
29/05/15 13:40	984	1080	728	1080
29/05/15 13:45	1284	1080	697	1188
29/05/15 13:50	840	1080	693	1140
29/05/15 13:55	972	1080	513	1272
29/05/15 14:00	1020	1080	716	1200
29/05/15 14:05	972	1080	720	1116
29/05/15 14:10	840	1080	766	1512
29/05/15 14:15	996	1080	676	1272
29/05/15 14:20	996	1080	615	1188
29/05/15 14:25	888	1080	800	1344
29/05/15 14:30	876	1080	609	1332
29/05/15 14:35	804	1080	649	1140
29/05/15 14:40	780	1080	808	1308
29/05/15 14:45	852	1080	865	1344
29/05/15 14:50	864	1080	567	1332
29/05/15 14:55	720	1080	780	1380
29/05/15 15:00	660	1080	708	1188
29/05/15 15:05	948	1080	741	1512
29/05/15 15:10	852	1080	549	1380
29/05/15 15:15	732	1080	769	1272
29/05/15 15:20	768	1080	681	1056
29/05/15 15:25	804	1080	769	1104
29/05/15 15:30	780	1080	740	1236
29/05/15 15:35	720	1080	918	1452
29/05/15 15:40	924	1080	748	1032
29/05/15 15:45	720	1080	829	1488
29/05/15 15:50	876	1080	562	1248
29/05/15 15:55	696	1080	813	1200
29/05/15 16:00	780	1080	710	1236
29/05/15 16:05	756	1080	787	1248
29/05/15 16:10	828	1080	704	1284
29/05/15 16:15	780	1080	890	1380
29/05/15 16:20	696	1080	710	1476
29/05/15 16:25	792	1080	954	1284
29/05/15 16:30	492	1080	630	1092
29/05/15 16:35	756	1080	963	1104
29/05/15 16:40	792	1080	637	1200
29/05/15 16:45	756	1080	823	924
29/05/15 16:50	660	1080	774	996
29/05/15 16:55	840	1080	292	984
29/05/15 17:00	732	1080	302	780
29/05/15 17:05	816	1080	280	408
29/05/15 17:10	648	1080	255	444
29/05/15 17:15	468	1080	254	588
29/05/15 17:20	276	1080	109	264
29/05/15 17:25	384	1080	158	276
29/05/15 17:30	900	1080	207	684
29/05/15 17:35	852	1080	199	840
29/05/15 17:40	1176	1080	166	684
29/05/15 17:45	1176	1080	190	924
29/05/15 17:50	528	1080	291	492
29/05/15 17:55	108	1080	235	648
29/05/15 18:00	660	1080	270	564
29/05/15 18:05	396	1080	410	660
29/05/15 18:10	312	1080	283	636
29/05/15 18:15	600	1116	106	636
29/05/15 18:20	864	1260	220	420

Sistema de Controle de Tráfego Urbano OPTIMUS

5 MINUTOS	INTENSIDADE			
	PM 040300 4	PM 040300 6	PM 040400 2	PM 040400 4
29/05/15 18:25	396	1260	146	300
29/05/15 18:30	228	1260	312	540
29/05/15 18:35	576	1260	200	396
29/05/15 18:40	768	1260	195	588
29/05/15 18:45	516	1260	270	576
29/05/15 18:50	264	1260	217	504
29/05/15 18:55	1140	1260	201	744
29/05/15 19:00	384	1224	268	504
29/05/15 19:05	744	1080	337	420
29/05/15 19:10	744	1080	207	588
29/05/15 19:15	504	1080	159	660
29/05/15 19:20	660	1080	306	648
29/05/15 19:25	636	1080	372	732
29/05/15 19:30	864	1080	309	576
29/05/15 19:35	744	1080	194	984
29/05/15 19:40	756	1080	345	720
29/05/15 19:45	636	1080	361	948
29/05/15 19:50	588	1080	295	960
29/05/15 19:55	708	1080	253	1104
29/05/15 20:00	804	1080	415	1092
29/05/15 20:05	420	1080	456	1104
29/05/15 20:10	660	1080	550	900
29/05/15 20:15	744	1080	650	1032
29/05/15 20:20	792	1080	528	1080
29/05/15 20:25	672	1080	496	1296
29/05/15 20:30	684	1080	584	1236
29/05/15 20:35	552	1080	568	1164
29/05/15 20:40	576	1080	586	888
29/05/15 20:45	600	1080	458	1116
29/05/15 20:50	480	1080	496	1224
29/05/15 20:55	612	1080	693	1152
29/05/15 21:00	456	1080	554	1032
29/05/15 21:05	516	1080	361	912
29/05/15 21:10	480	1080	571	1128
29/05/15 21:15	576	1080	609	1728
29/05/15 21:20	456	1080	480	1536
29/05/15 21:25	468	1080	526	1512
29/05/15 21:30	456	1080	476	732
29/05/15 21:35	480	1080	663	732
29/05/15 21:40	408	1080	477	816
29/05/15 21:45	372	1044	414	816
29/05/15 21:50	408	900	486	996
29/05/15 21:55	468	900	373	936
29/05/15 22:00	384	900	348	1032
29/05/15 22:05	324	900	549	840
29/05/15 22:10	444	900	408	900
29/05/15 22:15	504	900	567	864
29/05/15 22:20	552	900	428	1128
29/05/15 22:25	432	900	578	1008
29/05/15 22:30	432	900	489	780
29/05/15 22:35	396	900	450	840
29/05/15 22:40	420	900	565	792
29/05/15 22:45	276	900	429	624
29/05/15 22:50	264	900	474	732
29/05/15 22:55	360	900	338	624
29/05/15 23:00	432	900	332	804
29/05/15 23:05	408	900	405	816
29/05/15 23:10	240	900	254	612

Sistema de Controle de Tráfego Urbano OPTIMUS

5 MINUTOS	INTENSIDADE			
	PM 0403004	PM 0403006	PM 0404002	PM 0404004
29/05/15 23:15	264	864	273	840
29/05/15 23:20	324	720	189	708
29/05/15 23:25	240	720	246	588
29/05/15 23:30	288	720	217	540
29/05/15 23:35	276	720	211	612
29/05/15 23:40	228	720	206	588
29/05/15 23:45	288	720	196	672
29/05/15 23:50	240	720	192	612
29/05/15 23:55	288	720	231	468
30/05/15 00:00	348	684	256	492
30/05/15 00:05	324	540	224	624
30/05/15 00:10	204	540	212	600
30/05/15 00:15	132	540	152	576
30/05/15 00:20	180	540	154	372
30/05/15 00:25	228	540	170	660
30/05/15 00:30	276	540	302	384
30/05/15 00:35	216	540	176	504
30/05/15 00:40	240	540	147	612
30/05/15 00:45	180	540	236	468
30/05/15 00:50	108	540	129	348
30/05/15 00:55	240	540	162	408
30/05/15 01:00	252	540	74	432
30/05/15 01:05	204	540	93	348
30/05/15 01:10	60	540	114	300
30/05/15 01:15	120	504	106	312
30/05/15 01:20	192	360	130	324
30/05/15 01:25	180	360	70	348
30/05/15 01:30	84	360	130	252
30/05/15 01:35	156	360	142	420
30/05/15 01:40	84	360	116	204
30/05/15 01:45	84	360	50	228
30/05/15 01:50	84	360	110	228
30/05/15 01:55	144	360	112	240
30/05/15 02:00	72	360	55	276
30/05/15 02:05	96	360	127	384
30/05/15 02:10	48	360	86	348
30/05/15 02:15	60	360	66	288
30/05/15 02:20	132	360	93	192
30/05/15 02:25	72	360	36	300
30/05/15 02:30	120	360	55	288
30/05/15 02:35	156	360	111	204
30/05/15 02:40	144	360	98	204
30/05/15 02:45	60	360	50	264
30/05/15 02:50	84	360	75	192
30/05/15 02:55	84	360	63	276
30/05/15 03:00	48	324	36	168
30/05/15 03:05	108	180	55	288
30/05/15 03:10	96	180	24	192
30/05/15 03:15	108	180	79	192
30/05/15 03:20	60	180	43	252
30/05/15 03:25	48	180	63	168
30/05/15 03:30	60	180	72	180
30/05/15 03:35	36	180	75	120
30/05/15 03:40	120	180	51	180
30/05/15 03:45	96	180	87	192
30/05/15 03:50	24	180	39	96
30/05/15 03:55	24	180	51	144
30/05/15 04:00	48	180	55	168

Sistema de Controle de Tráfego Urbano OPTIMUS

5 MINUTOS	INTENSIDADE			
	PM 0403004	PM 0403006	PM 0404002	PM 0404004
30/05/15 04:05	48	180	36	192
30/05/15 04:10	36	180	36	144
30/05/15 04:15	84	180	79	240
30/05/15 04:20	84	180	51	120
30/05/15 04:25	72	180	46	204
30/05/15 04:30	84	180	75	144
30/05/15 04:35	120	180	27	168
30/05/15 04:40	36	180	123	192
30/05/15 04:45	96	180	51	228
30/05/15 04:50	36	180	43	264
30/05/15 04:55	48	180	60	144
30/05/15 05:00	108	180	46	204
30/05/15 05:05	48	180	48	144
30/05/15 05:10	96	180	99	168
30/05/15 05:15	60	180	79	264
30/05/15 05:20	72	180	31	288
30/05/15 05:25	72	180	39	192
30/05/15 05:30	36	180	36	192
30/05/15 05:35	60	180	12	204
30/05/15 05:40	120	180	50	312
30/05/15 05:45	192	180	86	240
30/05/15 05:50	72	180	79	228
30/05/15 05:55	84	180	55	156
30/05/15 06:00	84	180	48	252
30/05/15 06:05	204	180	86	372
30/05/15 06:10	216	180	106	312
30/05/15 06:15	120	180	182	372
30/05/15 06:20	132	180	90	348
30/05/15 06:25	156	180	122	360
30/05/15 06:30	276	180	67	492
30/05/15 06:35	216	180	129	408
30/05/15 06:40	336	180	91	444
30/05/15 06:45	432	180	169	468
30/05/15 06:50	564	180	165	600
30/05/15 06:55	588	180	177	504
30/05/15 07:00	492	180	301	504
30/05/15 07:05	504	180	237	588
30/05/15 07:10	492	180	297	696
30/05/15 07:15	408	180	241	588
30/05/15 07:20	408	180	289	708
30/05/15 07:25	384	180	236	672
30/05/15 07:30	600	180	258	756
30/05/15 07:35	540	180	200	804
30/05/15 07:40	564	180	182	720
30/05/15 07:45	696	180	270	984
30/05/15 07:50	900	180	169	972
30/05/15 07:55	768	180	267	1068
30/05/15 08:00	756	180	298	876
30/05/15 08:05	744	180	349	1080
30/05/15 08:10	576	180	321	912
30/05/15 08:15	768	180	348	1116
30/05/15 08:20	600	180	326	948
30/05/15 08:25	612	180	242	984
30/05/15 08:30	708	180	404	924
30/05/15 08:35	600	180	350	924
30/05/15 08:40	984	180	396	1224
30/05/15 08:45	768	180	358	1068
30/05/15 08:50	744	180	246	984

Sistema de Controle de Tráfego Urbano OPTIMUS

5 MINUTOS	INTENSIDADE			
	PM 040300 4	PM 040300 6	PM 040400 2	PM 040400 4
30/05/15 08:55	876	180	274	1200
30/05/15 09:00	864	180	457	1068
30/05/15 09:05	768	180	357	1092
30/05/15 09:10	840	180	283	984
30/05/15 09:15	960	180	433	1248
30/05/15 09:20	756	180	321	1332
30/05/15 09:25	768	180	378	1044
30/05/15 09:30	732	216	403	1152
30/05/15 09:35	936	360	439	1128
30/05/15 09:40	720	360	405	1080
30/05/15 09:45	804	360	477	1092
30/05/15 09:50	960	360	369	1056
30/05/15 09:55	924	360	439	1308
30/05/15 10:00	780	396	458	1188
30/05/15 10:05	984	540	451	1224
30/05/15 10:10	1020	540	396	1140
30/05/15 10:15	900	540	432	1224
30/05/15 10:20	756	540	267	1236
30/05/15 10:25	840	540	466	1080
30/05/15 10:30	708	540	562	1116
30/05/15 10:35	888	540	555	1236
30/05/15 10:40	1008	540	477	1212
30/05/15 10:45	948	540	534	1188
30/05/15 10:50	1224	540	492	1200
30/05/15 10:55	936	540	470	1236
30/05/15 11:00	1008	540	324	1212
30/05/15 11:05	948	540	366	1308
30/05/15 11:10	684	540	573	1272
30/05/15 11:15	744	576	501	1320
30/05/15 11:20	864	720	363	1188
30/05/15 11:25	792	720	384	1476
30/05/15 11:30	768	720	620	1140
30/05/15 11:35	792	720	503	1284
30/05/15 11:40	936	720	580	1176
30/05/15 11:45	732	720	493	1236
30/05/15 11:50	996	720	433	1104
30/05/15 11:55	684	720	559	1344
30/05/15 12:00	996	720	436	1044
30/05/15 12:05	756	720	588	1296
30/05/15 12:10	936	720	660	1236
30/05/15 12:15	756	756	687	1236
30/05/15 12:20	684	900	609	1044
30/05/15 12:25	864	900	650	1308
30/05/15 12:45	432	540	319	948
30/05/15 12:50	708	900	370	972
30/05/15 12:55	660	900	578	1080
30/05/15 13:00	804	900	428	1116
30/05/15 13:05	600	900	603	1488
30/05/15 13:10	780	900	574	984
30/05/15 13:15	744	936	721	1188
30/05/15 13:20	600	1080	573	912
30/05/15 13:25	756	1080	552	1320
30/05/15 13:30	768	1080	470	1008
30/05/15 13:35	624	1080	777	1140
30/05/15 13:40	768	1080	519	876
30/05/15 13:45	780	1080	510	1080
30/05/15 13:50	684	1080	567	924
30/05/15 13:55	744	1080	308	1092

Sistema de Controle de Tráfego Urbano OPTIMUS

5 MINUTOS	INTENSIDADE			
	PM 040300 4	PM 040300 6	PM 040400 2	PM 040400 4
30/05/15 14:00	624	1080	512	972
30/05/15 14:05	696	1080	297	1260
30/05/15 14:10	612	1080	306	1080
30/05/15 14:15	744	1044	426	1272
30/05/15 14:20	468	900	361	1068
30/05/15 14:25	552	900	399	1368
30/05/15 14:30	564	900	322	1044
30/05/15 14:35	540	900	406	1104
30/05/15 14:40	504	900	285	840
30/05/15 14:45	552	900	474	1440
30/05/15 14:50	624	900	352	900
30/05/15 14:55	660	900	324	1212
30/05/15 15:00	468	900	403	924
30/05/15 15:05	564	900	290	1128
30/05/15 15:10	540	900	410	1008
30/05/15 15:15	360	900	346	1548
30/05/15 15:20	540	900	442	828
30/05/15 15:25	516	900	385	1296
30/05/15 15:30	696	900	390	984
30/05/15 15:35	660	900	244	960
30/05/15 15:40	540	900	396	960
30/05/15 15:45	576	900	326	1152
30/05/15 15:50	516	900	424	1044
30/05/15 15:55	588	900	315	876
30/05/15 16:00	588	900	282	888
30/05/15 16:05	516	900	405	1020
30/05/15 16:10	564	900	360	1044
30/05/15 16:15	468	900	400	1008
30/05/15 16:20	528	900	337	972
30/05/15 16:25	528	900	417	888
30/05/15 16:30	564	900	387	996
30/05/15 16:35	564	900	404	1200
30/05/15 16:40	480	900	476	1164
30/05/15 16:45	444	900	346	1080
30/05/15 16:50	528	900	405	1128
30/05/15 16:55	444	900	453	1140
30/05/15 17:00	636	900	546	1116
30/05/15 17:05	504	900	603	1080
30/05/15 17:10	528	900	334	1128
30/05/15 17:15	552	900	428	1020
30/05/15 17:20	552	900	386	684
30/05/15 17:25	540	900	434	1080
30/05/15 17:30	612	900	429	852
30/05/15 17:35	588	900	314	1056
30/05/15 17:40	576	900	387	984
30/05/15 17:45	600	900	412	984
30/05/15 17:50	612	900	541	984
30/05/15 17:55	660	900	466	936
30/05/15 18:00	552	900	442	912
30/05/15 18:05	540	900	443	984
30/05/15 18:10	600	900	398	984
30/05/15 18:15	576	900	399	1212
30/05/15 18:20	828	900	348	1152
30/05/15 18:25	588	900	382	1068
30/05/15 18:30	648	900	296	1020
30/05/15 18:35	624	900	320	864
30/05/15 18:40	636	900	361	1176
30/05/15 18:45	672	900	262	1008

Sistema de Controle de Tráfego Urbano OPTIMUS

5 MINUTOS	INTENSIDADE			
	PM 0403004	PM 0403006	PM 0404002	PM 0404004
30/05/15 18:50	672	900	337	1056
30/05/15 18:55	636	900	334	852
30/05/15 19:00	720	900	283	996
30/05/15 19:05	804	900	350	1032
30/05/15 19:10	588	900	397	960
30/05/15 19:15	552	900	320	1008
30/05/15 19:20	612	900	315	900
30/05/15 19:25	480	900	316	984
30/05/15 19:30	564	900	334	984
30/05/15 19:35	732	900	328	1260
30/05/15 19:40	504	900	342	1104
30/05/15 19:45	564	900	320	888
30/05/15 19:50	540	900	201	792
30/05/15 19:55	468	900	332	1116
30/05/15 20:00	516	900	357	1164
30/05/15 20:05	396	900	318	816
30/05/15 20:10	588	900	312	924
30/05/15 20:15	576	900	237	1248
30/05/15 20:20	612	900	345	960
30/05/15 20:25	576	900	190	900
30/05/15 20:30	600	900	244	1092
30/05/15 20:35	612	900	230	888
30/05/15 20:40	576	900	320	876
30/05/15 20:45	492	900	201	876
30/05/15 20:50	624	900	280	852
30/05/15 20:55	384	900	202	696
30/05/15 21:00	516	900	356	912
30/05/15 21:05	528	900	253	1116
30/05/15 21:10	552	900	266	1176
30/05/15 21:15	420	900	192	948
30/05/15 21:20	384	900	157	912
30/05/15 21:25	360	900	192	816
30/05/15 21:30	504	900	281	864
30/05/15 21:35	444	900	189	780
30/05/15 21:40	396	900	364	1056
30/05/15 21:45	324	900	280	912
30/05/15 21:50	528	900	308	840
30/05/15 21:55	336	900	244	840
30/05/15 22:00	444	900	199	720
30/05/15 22:05	540	900	170	828
30/05/15 22:10	408	900	291	792
30/05/15 22:15	324	900	243	960
30/05/15 22:20	504	900	199	876
30/05/15 22:25	300	900	272	972
30/05/15 22:30	372	900	195	864
30/05/15 22:35	444	900	308	972
30/05/15 22:40	324	900	625	768
30/05/15 22:45	432	900	542	780
30/05/15 22:50	324	900	388	804
30/05/15 22:55	348	900	208	876
30/05/15 23:00	288	864	224	840
30/05/15 23:05	384	720	225	792
30/05/15 23:10	288	720	360	696
30/05/15 23:15	372	720	231	888
30/05/15 23:20	168	720	228	708
30/05/15 23:25	168	720	200	684
30/05/15 23:30	384	720	158	936
30/05/15 23:35	348	720	157	852

Sistema de Controle de Tráfego Urbano OPTIMUS

5 MINUTOS	INTENSIDADE			
	PM 040300 4	PM 040300 6	PM 040400 2	PM 040400 4
30/05/15 23:40	360	720	195	576
30/05/15 23:45	264	720	109	504
30/05/15 23:50	288	720	165	660
30/05/15 23:55	156	720	152	528
31/05/15 00:00	360	720	110	600
31/05/15 00:05	276	720	228	792
31/05/15 00:10	300	720	232	540
31/05/15 00:15	228	684	160	456
31/05/15 00:20	240	540	207	636
31/05/15 00:25	240	540	168	468
31/05/15 00:30	204	540	205	576
31/05/15 00:35	192	540	244	492
31/05/15 00:40	192	540	177	516
31/05/15 00:45	240	540	213	504
31/05/15 00:50	204	540	163	504
31/05/15 00:55	276	540	246	540
31/05/15 01:00	180	540	189	660
31/05/15 01:05	228	540	162	612
31/05/15 01:10	216	540	124	492
31/05/15 01:15	204	540	126	444
31/05/15 01:20	180	540	153	480
31/05/15 01:25	192	540	175	456
31/05/15 01:30	144	540	97	480
31/05/15 01:35	276	540	158	384
31/05/15 01:40	204	540	106	648
31/05/15 01:45	108	504	151	372
31/05/15 01:50	72	360	129	408
31/05/15 01:55	120	360	150	324
31/05/15 02:00	132	360	166	360
31/05/15 02:05	84	360	98	396
31/05/15 02:10	132	360	122	372
31/05/15 02:15	144	360	190	156
31/05/15 02:20	204	360	151	372
31/05/15 02:25	96	360	139	372
31/05/15 02:30	120	360	94	336
31/05/15 02:35	108	360	67	360
31/05/15 02:40	72	360	115	192
31/05/15 02:45	84	360	174	288
31/05/15 02:50	120	360	163	300
31/05/15 02:55	60	360	75	372
31/05/15 03:00	84	324	130	348
31/05/15 03:05	120	180	127	264
31/05/15 03:10	84	180	111	300
31/05/15 03:15	72	180	46	264
31/05/15 03:20	132	180	138	288
31/05/15 03:25	108	180	98	252
31/05/15 03:30	60	180	135	228
31/05/15 03:35	144	180	134	168
31/05/15 03:40	84	180	60	192
31/05/15 03:45	144	180	115	228
31/05/15 03:50	108	180	38	240
31/05/15 03:55	84	180	122	252
31/05/15 04:00	72	180	60	180
31/05/15 04:05	84	180	67	264
31/05/15 04:10	96	180	75	192
31/05/15 04:15	72	180	115	228
31/05/15 04:20	108	180	51	192
31/05/15 04:25	108	180	150	240

Sistema de Controle de Tráfego Urbano OPTIMUS

5 MINUTOS	INTENSIDADE			
	PM 0403004	PM 0403006	PM 0404002	PM 0404004
31/05/15 04:30	132	180	117	300
31/05/15 04:35	96	180	115	240
31/05/15 04:40	24	180	43	156
31/05/15 04:45	36	180	75	204
31/05/15 04:50	12	180	36	144
31/05/15 04:55	84	180	75	156
31/05/15 05:00	36	180	110	192
31/05/15 05:05	96	180	22	192
31/05/15 05:10	108	180	79	144
31/05/15 05:15	72	180	50	252
31/05/15 05:20	36	180	84	204
31/05/15 05:25	36	180	82	144
31/05/15 05:30	84	144	67	300
31/05/15 05:35	84	0	60	204
31/05/15 05:40	48	0	60	180
31/05/15 05:45	24	0	39	276
31/05/15 05:50	60	0	87	216
31/05/15 05:55	72	0	51	216
31/05/15 06:00	60	0	79	204
31/05/15 06:05	60	0	55	180
31/05/15 06:10	84	0	115	228
31/05/15 06:15	108	0	67	216
31/05/15 06:20	156	0	139	432
31/05/15 06:25	156	0	98	216
31/05/15 06:30	120	0	94	252
31/05/15 06:35	120	0	102	240
31/05/15 06:40	180	0	103	288
31/05/15 06:45	348	0	91	324
31/05/15 06:50	204	0	117	432
31/05/15 06:55	216	0	135	408
31/05/15 07:00	216	0	201	228
31/05/15 07:05	252	0	163	456
31/05/15 07:10	348	0	246	348
31/05/15 07:15	228	0	174	408
31/05/15 07:20	300	0	226	372
31/05/15 07:25	228	0	226	528
31/05/15 07:30	240	0	151	324
31/05/15 07:35	204	0	210	432
31/05/15 07:40	264	0	202	480
31/05/15 07:45	312	0	234	480
31/05/15 07:50	276	0	146	480
31/05/15 07:55	264	0	265	612
31/05/15 08:00	288	0	198	300
31/05/15 08:05	204	0	230	360
31/05/15 08:10	300	0	164	384
31/05/15 08:15	336	0	270	444
31/05/15 08:20	372	0	282	588
31/05/15 08:25	408	0	254	504
31/05/15 08:30	384	36	103	408
31/05/15 08:35	360	180	172	660
31/05/15 08:40	540	180	248	384
31/05/15 08:45	444	180	246	852
31/05/15 08:50	372	180	270	672
31/05/15 08:55	468	180	292	852
31/05/15 09:00	468	180	172	648
31/05/15 09:05	576	180	218	528
31/05/15 09:10	312	180	277	588
31/05/15 09:15	468	180	309	540

Sistema de Controle de Tráfego Urbano OPTIMUS

5 MINUTOS	INTENSIDADE			
	PM 040300 4	PM 040300 6	PM 040400 2	PM 040400 4
31/05/15 09:20	396	180	218	552
31/05/15 09:25	528	180	181	708
31/05/15 09:30	396	216	223	780
31/05/15 09:35	444	360	250	780
31/05/15 09:40	444	360	229	648
31/05/15 09:45	396	360	237	564
31/05/15 09:50	504	360	268	780
31/05/15 09:55	288	360	220	720
31/05/15 10:00	480	360	201	792
31/05/15 10:05	408	360	157	468
31/05/15 10:10	396	360	254	696
31/05/15 10:15	396	360	277	756
31/05/15 10:20	360	360	320	804
31/05/15 10:25	372	360	94	924
31/05/15 10:30	444	360	260	672
31/05/15 10:35	396	360	289	672
31/05/15 10:40	624	360	326	936
31/05/15 10:45	624	360	442	1008
31/05/15 10:50	408	360	285	828
31/05/15 10:55	504	360	352	672
31/05/15 11:00	456	360	235	720
31/05/15 11:05	540	360	376	900
31/05/15 11:10	444	360	212	576
31/05/15 11:15	540	360	328	948
31/05/15 11:20	468	360	362	792
31/05/15 11:25	624	360	392	828
31/05/15 11:30	504	360	361	936
31/05/15 11:35	528	360	408	840
31/05/15 11:40	468	360	338	864
31/05/15 11:45	480	360	313	768
31/05/15 11:50	624	360	312	948
31/05/15 11:55	600	360	445	972
31/05/15 12:00	492	360	414	1128
31/05/15 12:05	612	360	404	1032
31/05/15 12:10	420	360	478	624
31/05/15 12:15	732	396	321	1080
31/05/15 12:20	660	540	471	876
31/05/15 12:25	576	540	412	1140
31/05/15 12:30	672	540	385	1068
31/05/15 12:35	648	540	471	780
31/05/15 12:40	612	540	314	924
31/05/15 12:45	696	576	373	816
31/05/15 12:50	708	720	368	756
31/05/15 12:55	552	720	378	780
31/05/15 13:00	540	720	434	792
31/05/15 13:05	468	720	475	732
31/05/15 13:10	672	720	369	864
31/05/15 13:15	660	720	396	816
31/05/15 13:20	876	720	414	912
31/05/15 13:25	756	720	464	840
31/05/15 13:30	684	720	261	924
31/05/15 13:35	624	720	351	828
31/05/15 13:40	672	720	414	1344
31/05/15 13:45	444	720	363	1176
31/05/15 13:50	396	720	338	888
31/05/15 13:55	600	720	403	888
31/05/15 14:00	552	720	394	648
31/05/15 14:05	516	720	301	624

Sistema de Controle de Tráfego Urbano OPTIMUS

5 MINUTOS	INTENSIDADE			
	PM 040300 4	PM 040300 6	PM 040400 2	PM 040400 4
31/05/15 14:10	492	720	400	708
31/05/15 14:15	660	720	266	828
31/05/15 14:20	456	720	318	588
31/05/15 14:25	516	720	340	864
31/05/15 14:30	360	720	333	756
31/05/15 14:35	636	720	321	960
31/05/15 14:40	516	720	284	936
31/05/15 14:45	528	720	285	756
31/05/15 14:50	624	720	156	744
31/05/15 14:55	600	720	166	948
31/05/15 15:00	696	720	206	684
31/05/15 15:05	516	720	265	924
31/05/15 15:10	528	720	276	864
31/05/15 15:15	744	720	235	948
31/05/15 15:20	576	720	141	732
31/05/15 15:25	564	720	109	804
31/05/15 15:30	648	720	211	828
31/05/15 15:35	564	720	156	984
31/05/15 15:40	552	720	152	792
31/05/15 15:45	360	720	199	1008
31/05/15 15:50	600	720	206	744
31/05/15 15:55	600	720	289	876
31/05/15 16:00	636	720	194	912
31/05/15 16:05	432	720	182	924
31/05/15 16:10	516	720	97	792
31/05/15 16:15	420	720	108	936
31/05/15 16:20	528	720	185	876
31/05/15 16:25	528	720	162	888
31/05/15 16:30	372	720	148	840
31/05/15 16:35	444	720	213	828
31/05/15 16:40	492	720	201	840
31/05/15 16:45	576	720	159	660
31/05/15 16:50	492	720	226	708
31/05/15 16:55	384	720	217	972
31/05/15 17:00	444	720	122	636
31/05/15 17:05	408	720	178	864
31/05/15 17:10	480	720	188	948
31/05/15 17:15	420	720	141	828
31/05/15 17:20	444	720	201	756
31/05/15 17:25	600	720	241	972
31/05/15 17:30	456	720	247	744
31/05/15 17:35	528	720	217	912
31/05/15 17:40	492	720	175	888
31/05/15 17:45	600	720	145	936
31/05/15 17:50	684	720	213	1272
31/05/15 17:55	840	720	165	1260
31/05/15 18:00	1308	720	266	1200
31/05/15 18:05	1104	720	199	1800
31/05/15 18:10	1080	720	267	1644
31/05/15 18:15	1224	720	136	1764
31/05/15 18:20	1044	720	249	1464
31/05/15 18:25	1068	720	142	1932
31/05/15 18:30	900	720	124	1536
31/05/15 18:35	1200	720	157	2064
31/05/15 18:40	792	720	193	1476
31/05/15 18:45	1056	720	222	1836
31/05/15 18:50	960	720	216	1404
31/05/15 18:55	636	720	219	1692

Sistema de Controle de Tráfego Urbano OPTIMUS

5 MINUTOS	INTENSIDADE			
	PM 0403004	PM 0403006	PM 0404002	PM 0404004
31/05/15 19:00	576	720	230	1128
31/05/15 19:05	516	720	149	984
31/05/15 19:10	612	720	282	1152
31/05/15 19:15	552	756	276	1044
31/05/15 19:20	708	900	236	984
31/05/15 19:25	540	900	248	1008
31/05/15 19:30	372	900	410	744
31/05/15 19:35	432	900	248	996
31/05/15 19:40	504	900	152	744
31/05/15 19:45	480	900	259	792
31/05/15 19:50	576	900	260	960
31/05/15 19:55	624	900	207	924
31/05/15 20:00	420	900	144	768
31/05/15 20:05	432	900	198	864
31/05/15 20:10	336	900	204	672
31/05/15 20:15	420	900	205	840
31/05/15 20:20	408	900	235	768
31/05/15 20:25	456	900	338	720
31/05/15 20:30	372	900	128	696
31/05/15 20:35	264	900	122	768
31/05/15 20:40	468	900	242	732
31/05/15 20:45	348	900	298	888
31/05/15 20:50	504	900	175	852
31/05/15 20:55	420	900	163	648
31/05/15 21:00	396	900	176	792
31/05/15 21:05	264	900	189	696
31/05/15 21:10	360	900	205	624
31/05/15 21:15	336	900	259	792
31/05/15 21:20	324	900	193	924
31/05/15 21:25	348	900	308	804
31/05/15 21:30	324	900	207	708
31/05/15 21:35	312	900	237	600
31/05/15 21:40	372	900	213	660
31/05/15 21:45	192	864	181	624
31/05/15 21:50	408	720	207	576
31/05/15 21:55	264	720	130	540
31/05/15 22:00	216	684	111	612
31/05/15 22:05	132	540	109	492
31/05/15 22:10	324	540	160	444
31/05/15 22:15	240	540	148	600
31/05/15 22:20	252	540	127	552
31/05/15 22:25	156	540	108	516
31/05/15 22:30	312	540	177	528
31/05/15 22:35	228	540	86	444
31/05/15 22:40	264	540	86	528
31/05/15 22:45	156	540	145	540
31/05/15 22:50	204	540	87	432
31/05/15 22:55	120	540	82	396
31/05/15 23:00	144	540	181	312
31/05/15 23:05	180	540	237	420
31/05/15 23:10	204	540	148	480
31/05/15 23:15	144	504	90	444
31/05/15 23:20	120	360	105	408
31/05/15 23:25	192	360	79	360
31/05/15 23:30	168	360	62	240
31/05/15 23:35	132	360	26	228
31/05/15 23:40	60	360	36	264
31/05/15 23:45	96	360	38	324

Sistema de Controle de Tráfego Urbano OPTIMUS

5 MINUTOS	INTENSIDADE			
	PM 0403004	PM 0403006	PM 0404002	PM 0404004
31/05/15 23:50	120	360	55	312
31/05/15 23:55	108	360	86	228
01/06/15 00:00	120	360	67	180
01/06/15 00:05	96	360	75	264
01/06/15 00:10	72	360	102	156
01/06/15 00:15	36	324	74	180
01/06/15 00:20	72	180	58	240
01/06/15 00:25	60	180	79	192
01/06/15 00:30	48	180	43	228
01/06/15 00:35	48	180	67	132
01/06/15 00:40	96	180	7	168
01/06/15 00:45	96	180	24	168
01/06/15 00:50	48	180	36	156
01/06/15 00:55	60	180	27	156
01/06/15 01:00	48	180	55	96
01/06/15 01:05	36	180	27	144
01/06/15 01:10	36	180	22	108
01/06/15 01:15	60	144	14	132
01/06/15 01:20	0	0	36	60
01/06/15 01:25	48	0	48	132
01/06/15 01:30	0	0	39	84
01/06/15 01:35	24	0	51	48
01/06/15 01:40	24	0	39	48
01/06/15 01:45	0	0	27	72
01/06/15 01:50	12	0	15	120
01/06/15 01:55	12	0	0	24
01/06/15 02:00	12	0	0	48
01/06/15 02:05	12	0	12	120
01/06/15 02:10	48	0	3	72
01/06/15 02:15	36	0	12	60
01/06/15 02:20	60	0	3	156
01/06/15 02:25	36	0	24	120
01/06/15 02:30	12	0	12	84
01/06/15 02:35	24	0	0	48
01/06/15 02:40	24	0	36	72
01/06/15 02:45	24	0	24	96
01/06/15 02:50	24	0	12	72
01/06/15 02:55	0	0	36	72
01/06/15 03:00	60	0	36	48
01/06/15 03:05	0	0	24	72
01/06/15 03:10	0	0	15	48
01/06/15 03:15	12	0	39	48
01/06/15 03:20	24	0	19	72
01/06/15 03:25	12	0	3	36
01/06/15 03:30	24	0	36	48
01/06/15 03:35	24	0	36	48
01/06/15 03:40	48	0	24	36
01/06/15 03:45	0	0	48	72
01/06/15 03:50	12	0	36	36
01/06/15 03:55	24	0	7	36
01/06/15 04:00	0	0	12	24
01/06/15 04:05	24	0	36	84
01/06/15 04:10	36	0	12	96
01/06/15 04:15	12	0	12	108
01/06/15 04:20	36	0	27	36
01/06/15 04:25	24	0	24	84
01/06/15 04:30	12	0	27	72
01/06/15 04:35	60	0	12	144

Sistema de Controle de Tráfego Urbano OPTIMUS

5 MINUTOS	INTENSIDADE			
	PM 0403004	PM 0403006	PM 0404002	PM 0404004
01/06/15 04:40	24	0	24	72
01/06/15 04:45	48	0	15	96
01/06/15 04:50	12	0	3	96
01/06/15 04:55	60	0	27	60
01/06/15 05:00	24	0	82	108
01/06/15 05:05	84	0	70	204
01/06/15 05:10	48	0	3	144
01/06/15 05:15	72	0	36	180
01/06/15 05:20	96	0	31	300
01/06/15 05:25	72	0	43	204
01/06/15 05:30	132	0	106	252
01/06/15 05:35	84	0	60	252
01/06/15 05:40	240	0	163	300
01/06/15 05:45	216	0	91	324
01/06/15 05:50	180	0	106	336
01/06/15 05:55	264	0	151	348
01/06/15 06:00	204	0	175	396
01/06/15 06:05	396	0	98	516
01/06/15 06:10	276	0	202	312
01/06/15 06:15	360	0	162	552
01/06/15 06:20	480	0	103	552
01/06/15 06:25	696	0	110	744
01/06/15 06:30	648	0	282	804
01/06/15 06:35	1116	0	238	948
01/06/15 06:40	1332	0	177	1032
01/06/15 06:45	1524	0	255	1320
01/06/15 06:50	1560	0	448	1800
01/06/15 06:55	1800	0	301	1248
01/06/15 07:00	1632	0	693	1500
01/06/15 07:05	2112	0	606	1236
01/06/15 07:10	1896	0	679	1764
01/06/15 07:15	1860	0	701	1272
01/06/15 07:20	2088	0	668	1644
01/06/15 07:25	1836	0	476	1620
01/06/15 07:30	2076	0	567	1944
01/06/15 07:35	1740	0	523	1452
01/06/15 07:40	2124	0	613	1704
01/06/15 07:45	1752	0	657	1536
01/06/15 07:50	2148	0	442	1692
01/06/15 07:55	1728	0	498	1416
01/06/15 08:00	2040	0	464	1620
01/06/15 08:05	1620	0	442	1032
01/06/15 08:10	1740	0	520	1584
01/06/15 08:15	1692	0	434	1632
01/06/15 08:20	1440	0	466	1476
01/06/15 08:25	1344	0	399	1104
01/06/15 08:30	1548	36	494	1488
01/06/15 08:35	1320	180	459	1344
01/06/15 08:40	1392	180	498	1392
01/06/15 08:45	1500	180	570	1512
01/06/15 08:50	1512	180	626	1296
01/06/15 08:55	1308	180	498	1152
01/06/15 09:00	1272	216	590	1224
01/06/15 09:05	1404	360	558	1224
01/06/15 09:10	1452	360	638	1368
01/06/15 09:15	1092	396	752	1068
01/06/15 09:20	936	540	483	1200
01/06/15 09:25	1080	540	679	1212

Sistema de Controle de Tráfego Urbano OPTIMUS

5 MINUTOS	INTENSIDADE			
	PM 040300 4	PM 040300 6	PM 040400 2	PM 040400 4
01/06/15 09:30	1092	576	652	1464
01/06/15 09:35	1032	720	504	1260
01/06/15 09:40	912	720	552	1356
01/06/15 09:45	1056	720	584	1128
01/06/15 09:50	912	720	500	1320
01/06/15 09:55	888	720	469	1380
01/06/15 10:00	828	720	556	1032
01/06/15 10:05	780	720	619	1176
01/06/15 10:10	612	720	498	1104
01/06/15 10:15	828	720	637	1044
01/06/15 10:20	732	720	520	1308
01/06/15 10:25	828	720	588	1188
01/06/15 10:30	852	720	565	1224
01/06/15 10:35	804	720	562	1368
01/06/15 10:40	720	720	634	1044
01/06/15 10:45	648	720	762	1272
01/06/15 10:50	564	720	625	960
01/06/15 10:55	708	720	597	1236
01/06/15 11:00	660	720	733	1164
01/06/15 11:05	672	720	704	1284
01/06/15 11:10	696	720	771	1128
01/06/15 11:15	636	720	979	1308
01/06/15 11:20	516	720	919	1056
01/06/15 11:25	720	720	694	1164
01/06/15 11:30	576	720	684	1044
01/06/15 11:35	804	720	686	1152
01/06/15 11:40	660	720	730	1140
01/06/15 11:45	636	720	703	984
01/06/15 11:50	624	720	733	1272
01/06/15 11:55	792	720	828	1116
01/06/15 12:00	624	720	739	720
01/06/15 12:05	720	720	757	1164
01/06/15 12:10	708	720	848	1308
01/06/15 12:15	780	720	888	1116
01/06/15 12:20	588	720	636	1008
01/06/15 12:25	624	720	609	1008
01/06/15 12:30	636	720	660	1044
01/06/15 12:35	816	720	628	972
01/06/15 12:40	852	720	721	1272
01/06/15 12:45	1020	756	802	1212
01/06/15 12:50	960	900	604	996
01/06/15 12:55	1032	900	853	1260
01/06/15 13:00	876	900	709	1032
01/06/15 13:05	1068	900	687	1128
01/06/15 13:10	1056	900	543	1296
01/06/15 13:15	1032	900	662	1224
01/06/15 13:20	1284	900	691	1476
01/06/15 13:25	1056	900	556	1344
01/06/15 13:30	984	900	603	1164
01/06/15 13:35	1056	900	856	996
01/06/15 13:40	1056	900	638	1524
01/06/15 13:45	1104	900	614	1512
01/06/15 13:50	1236	900	747	1164
01/06/15 13:55	1176	900	573	1344
01/06/15 14:00	804	900	651	1152
01/06/15 14:05	876	900	457	1344
01/06/15 14:10	948	900	643	1260
01/06/15 14:15	960	900	560	1344

Sistema de Controle de Tráfego Urbano OPTIMUS

5 MINUTOS	INTENSIDADE			
	PM 0403004	PM 0403006	PM 0404002	PM 0404004
01/06/15 14:20	840	900	678	1284
01/06/15 14:25	876	900	601	1404
01/06/15 14:30	996	900	543	1260
01/06/15 14:35	696	900	651	1332
01/06/15 14:40	1008	900	770	1308
01/06/15 14:45	1020	900	596	1272
01/06/15 14:50	984	900	626	1224
01/06/15 14:55	840	900	654	1260
01/06/15 15:00	744	900	826	1452
01/06/15 15:05	732	900	690	1260
01/06/15 15:10	900	900	724	1380
01/06/15 15:15	888	900	691	1488
01/06/15 15:20	732	900	831	1356
01/06/15 15:25	600	900	614	1164
01/06/15 15:30	996	900	745	1236
01/06/15 15:35	888	900	597	1476
01/06/15 15:40	828	900	768	1104
01/06/15 15:45	720	900	558	1320
01/06/15 15:50	924	900	763	1056
01/06/15 15:55	600	900	752	1092
01/06/15 16:00	828	900	713	1284
01/06/15 16:05	720	900	592	1164
01/06/15 16:10	552	900	814	1212
01/06/15 16:15	732	936	685	1212
01/06/15 16:20	684	1080	928	1296
01/06/15 16:25	756	1080	819	1104
