# Supplementary figures and images for: Traffic light optimization using non-dominated sorting genetic algorithm (NSGA2)
Source: Sci Rep. 2023 Sep 20;13:15550. doi: 10.1038/s41598-023-38884-2 (PMC10511403; doi:10.1038/s41598-023-38884-2)

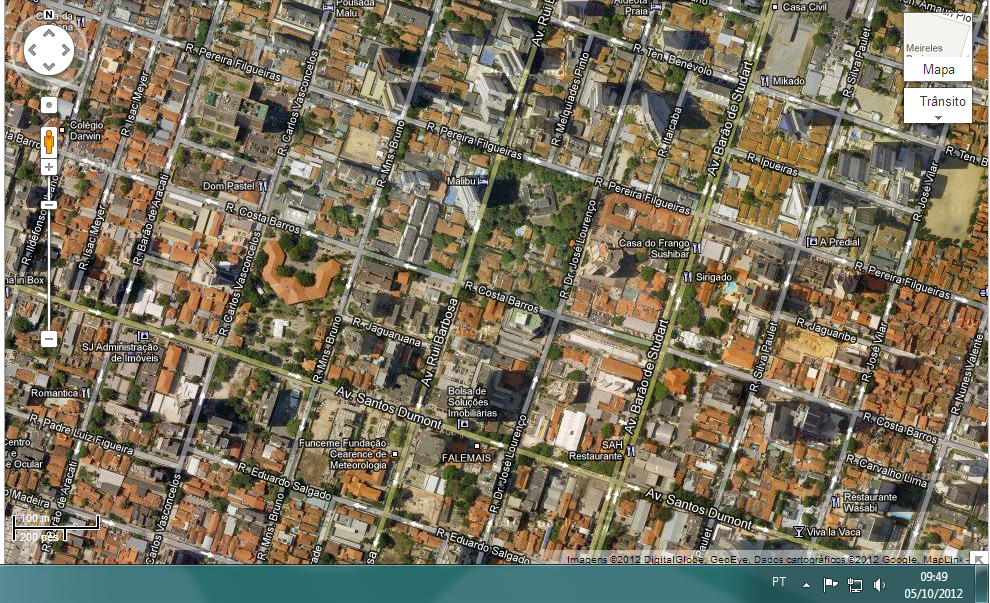

Supplement: Supplementary file 1 — Supplementary Information. [file 41598_2023_38884_MOESM1_ESM.zip › dadosBHTrans/calibrac+î-oa+î▌Æo do modelo/Arquivos/AG-Micro/AV Santos Dumont x R Pereira Filgueiras Satelite.png]

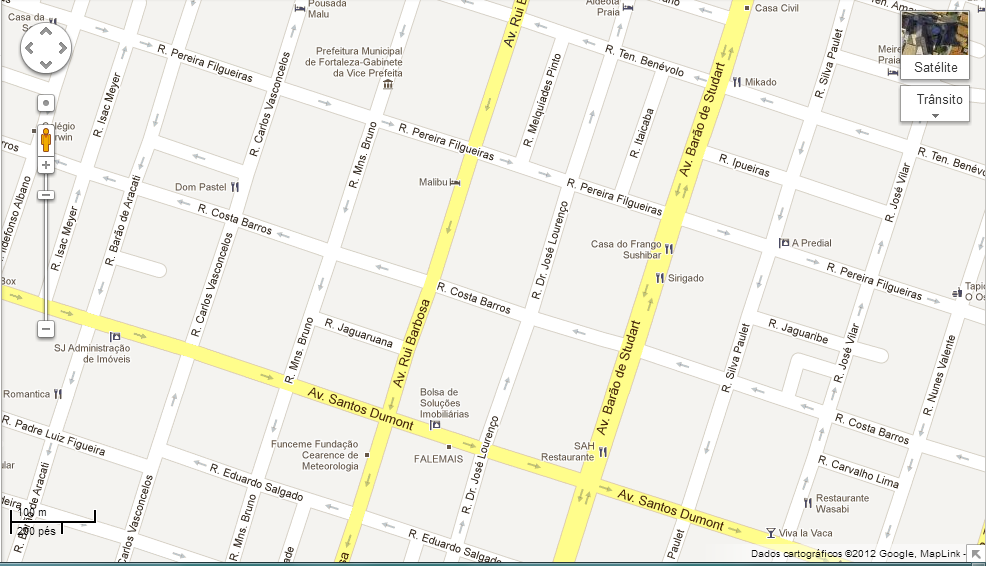

Supplement: Supplementary file 1 — Supplementary Information. [file 41598_2023_38884_MOESM1_ESM.zip › dadosBHTrans/calibrac+î-oa+î▌Æo do modelo/Arquivos/AG-Micro/AV Santos Dumont x R Pereira Filgueiras.png]

Contorno c/ Getúlio Vargas


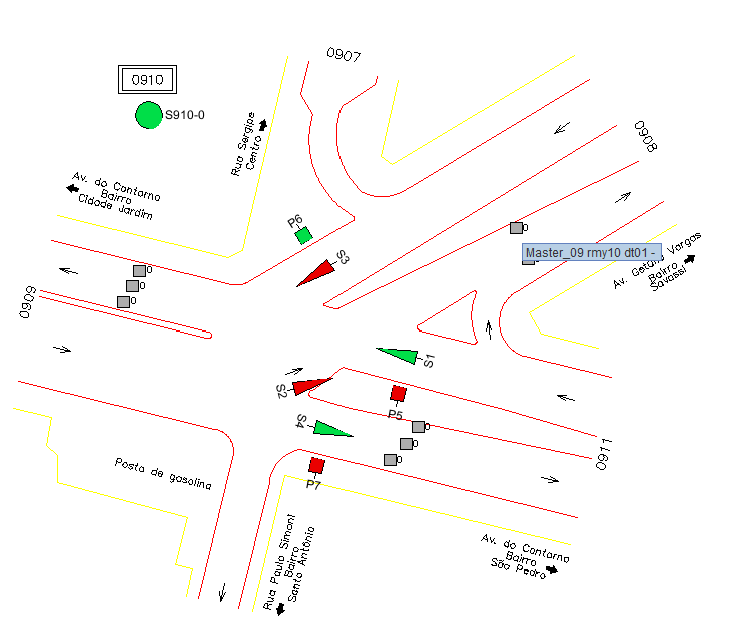


Getúlio Vargas c/ Alagoas


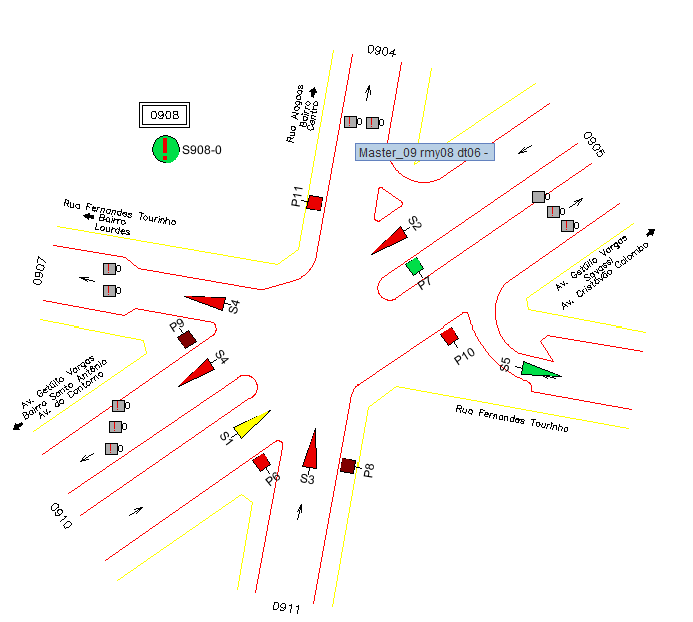


Contorno c/ Alagoas


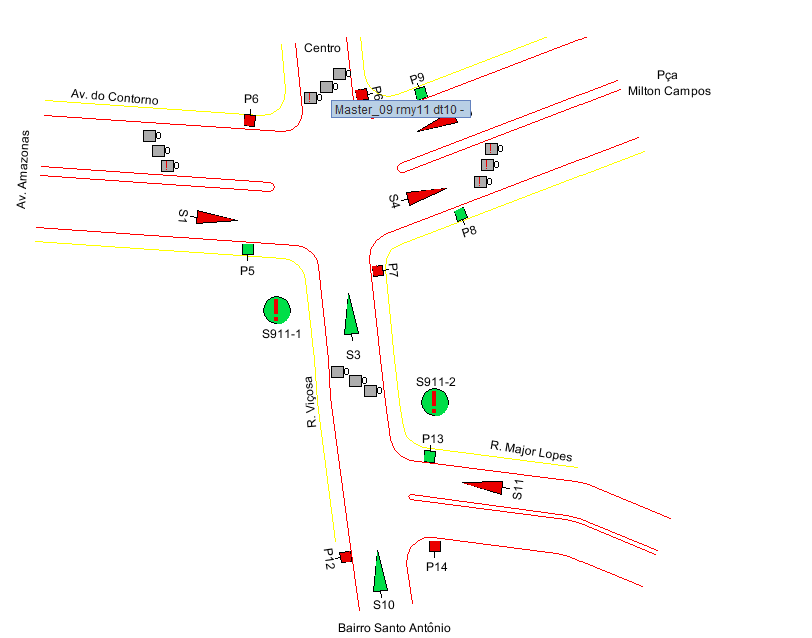

Supplement: Supplementary file 1 — Supplementary Information. [file 41598_2023_38884_MOESM1_ESM.zip › dadosBHTrans/dados BHTrans 2019/COMPILADO SAVASSI/subAreaSavassi.docx]

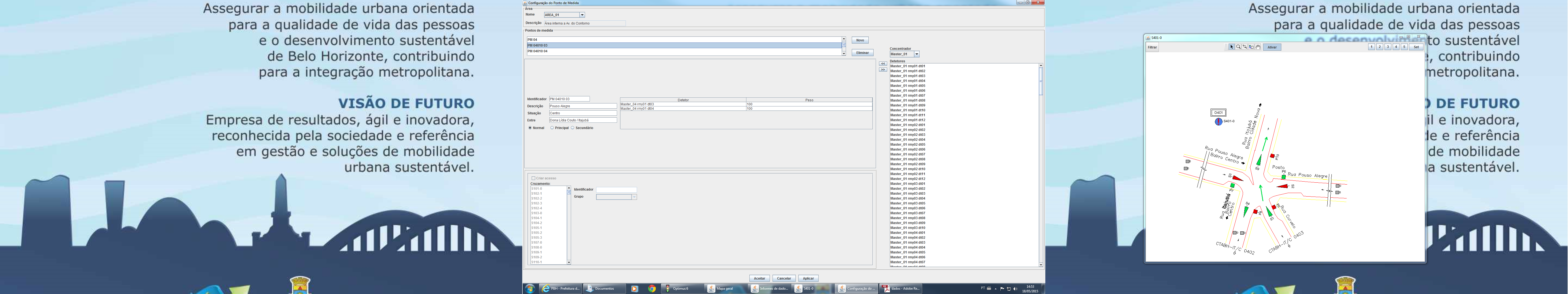


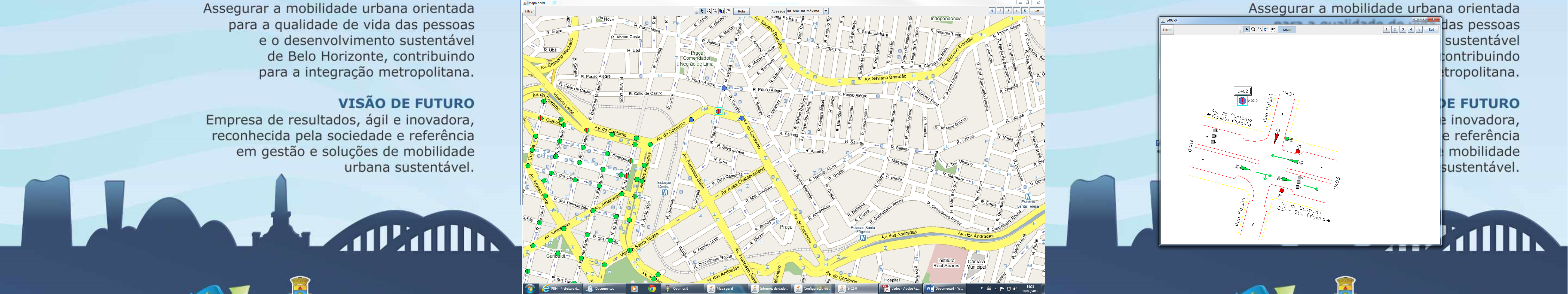


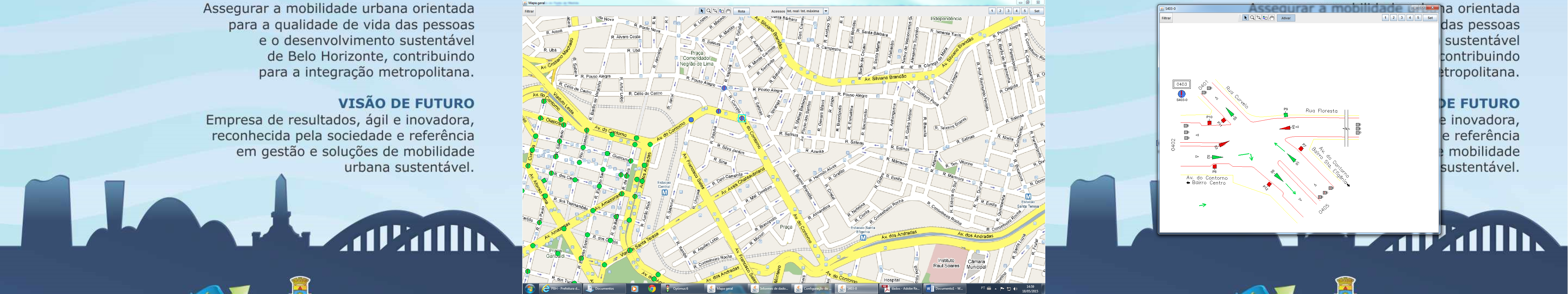


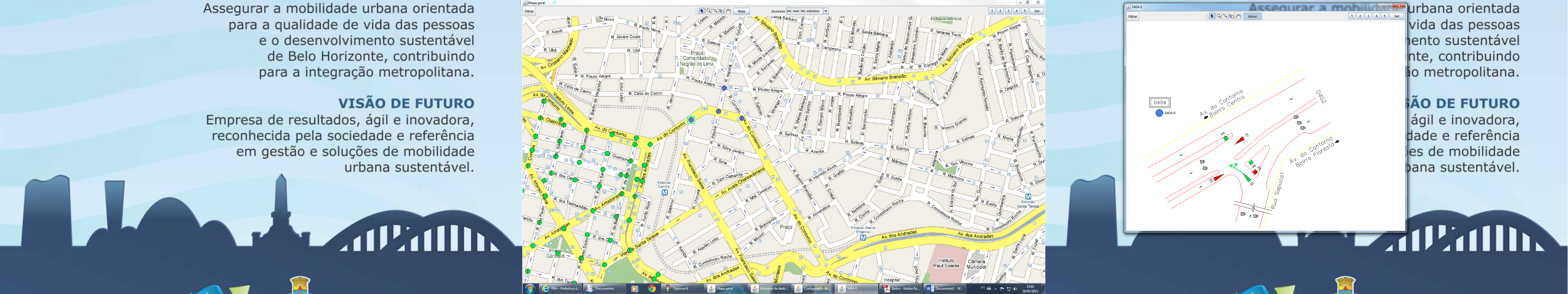


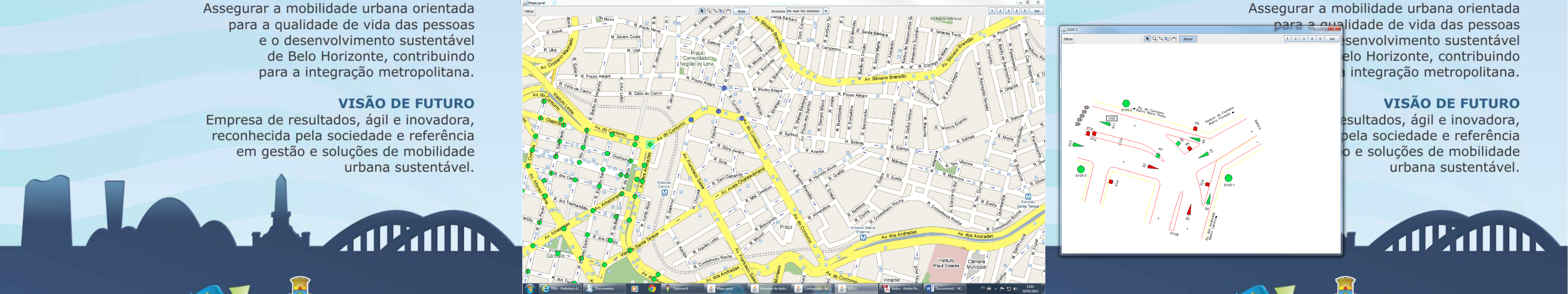

Supplement: Supplementary file 1 — Supplementary Information. [file 41598_2023_38884_MOESM1_ESM.zip › dadosBHTrans/Mapas.docx]
